# Supplementary material for: Chemogenomics for NR1 nuclear hormone receptors
Source: Nat Commun. 2024 Jun 18;15:5201. doi: 10.1038/s41467-024-49493-6 (PMC11189487; doi:10.1038/s41467-024-49493-6)
Supplement: Supplementary file 1 — Supplementary Information [file 41467_2024_49493_MOESM1_ESM.pdf]

Chemogenomics for NR1 nuclear hormone receptors

Laura Isigkeit[1,#], Espen Schallmayer[1,#], Romy Busch[2], Lorene Brunello[1,3], Amelie Menge[1,3], Lewis Elson[1,3], Susanne Müller[1,3], Stefan Knapp[1,3], Alexandra Stolz[3], Julian A. Marschner[2], Daniel Merk[1,2*]

[1] Goethe University Frankfurt, Institute of Pharmaceutical Chemistry, 60438 Frankfurt, Germany
[2] Ludwig-Maximilians-Universität (LMU) München, Department of Pharmacy, 81377 Munich, Germany
[3] Buchmann Institute for Molecular Life Sciences, Goethe University Frankfurt, 60438 Frankfurt, Germany
[#] L.I. and E.S. contributed equally to this study
* daniel.merk@cup.lmu.de

Table of Contents

Supplementary Figures and Tables 2
Supplementary References 17

Supplementary Figures

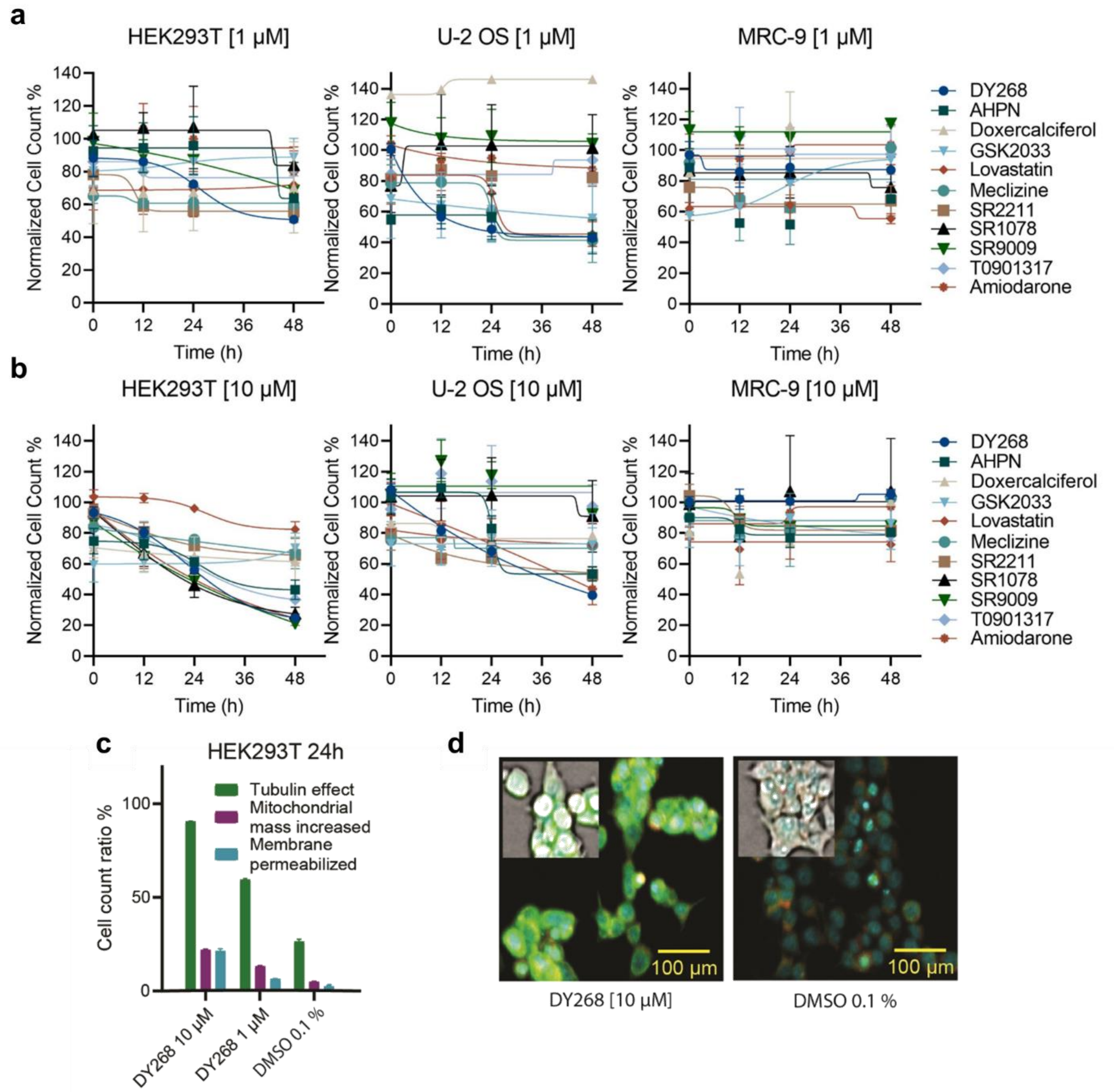

Supplementary Figure 1. Multiplex assay of HEK293T, U-2 OS and MRC-9 cells. (a, b) Cell count (%, relative to 0.1% DMSO) after 0 h, 12 h, 24 h and 48 h of HEK293T, U-2 OS and MRC-9 cells exposed to compounds at 1 µM (a) or 10 µM (b). Data are the mean ± S.E.M.; n = 2. (c) Cell count ratio of tubulin effect (green), mitochondrial mass increase (magenta) and membrane permeabilized (blue) of HEK293T cells after 24h of compound exposure (DY268 [1 µM, 10 µM]) in comparison to cells exposed to DMSO 0.1 %. Error bars show S.E.M. of biological duplicates. (d) Fluorescent image and brightfield confocal image of stained (blue: DNA/nuclei, green: microtubules, red: mitochondria, magenta: Annexin V apoptosis marker) HEK293T cells after 24 h of compound exposure (DY268 [10 µM]), in comparison to cells exposed to 0.1 % DMSO.

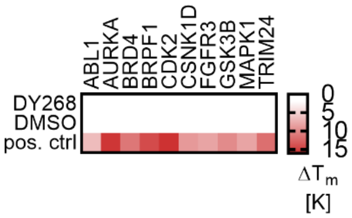

Supplementary Figure 2. Liability target screening of 1 µM DY268 by differential scanning fluorimetry. Proteins were used at 2 µM and as references (20 µM) staurosporine (ABL1, AURKA, CDK2, FGFR3 and GSK3B), (+)-JQ1 (BRD4), GSK6853 (BRPF1), PK016714a (CSNK1D), GDC-0994 (MAPK1) and IACS-9571 (TRIM24) were used. The heatmap shows the mean ΔTm calculated by the Boltzmann fit; n = 2.

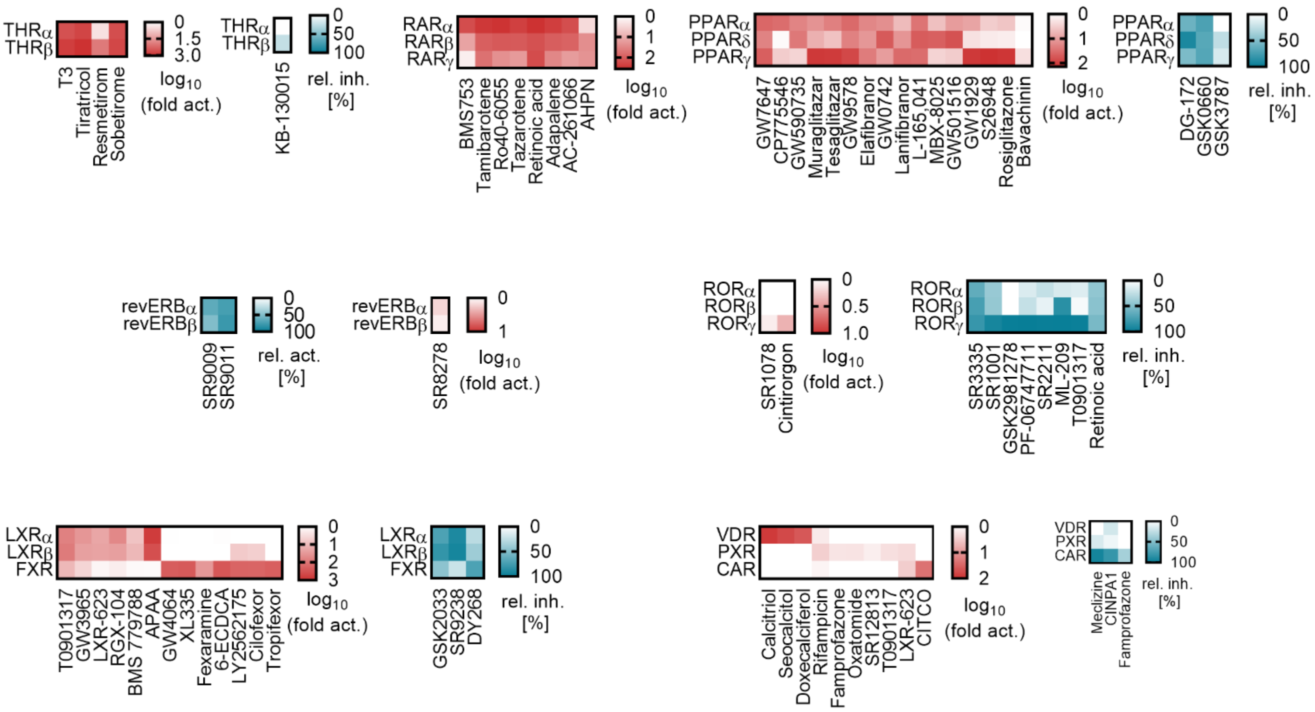

Supplementary Figure 3. Detailed in-family selectivity profiles of NR1 CG compounds at the recommended concentrations (Table 1). Red heatmaps show NR mediated activating effects expressed as mean log₁₀ fold activation; blue heatmaps show NR mediated inhibiting effects expressed as mean relative inhibition compared to the respective reference agonists (for antagonists) or compared to the receptor's basal activity (for inverse agonists); n ≥ 3.

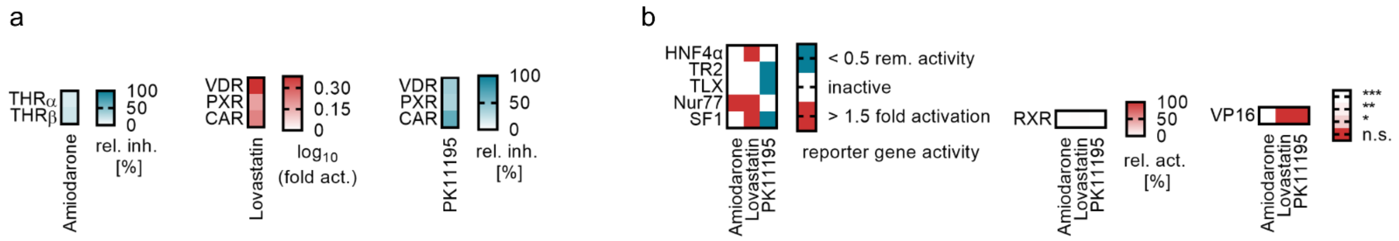

Supplementary Figure 4. (a) Detailed in-family selectivity profiles of the CG compound candidates amiodarone [10 µM], lovastatin [10 µM] and PK11195 [10 µM] (Table 1). Red heatmaps show NR mediated activating effects expressed as mean $\log_{10}$ fold activation; blue heatmaps show NR mediated inhibiting effects expressed as mean relative inhibition compared to the respective reference agonists for antagonists; n ≥ 3. (b) Selectivity profiling of amiodarone [10 µM], lovastatin [10 µM] and PK11195 [10 µM] on representative NRs outside the NR1 family, RXR and VP16. The heatmap shows NR mediated activation (red; agonists) and inhibition of reporter gene expression (blue; antagonists and inverse agonists), expressed as mean fold activation. Activities were determined in uniform hybrid reporter gene assays; n = 3.

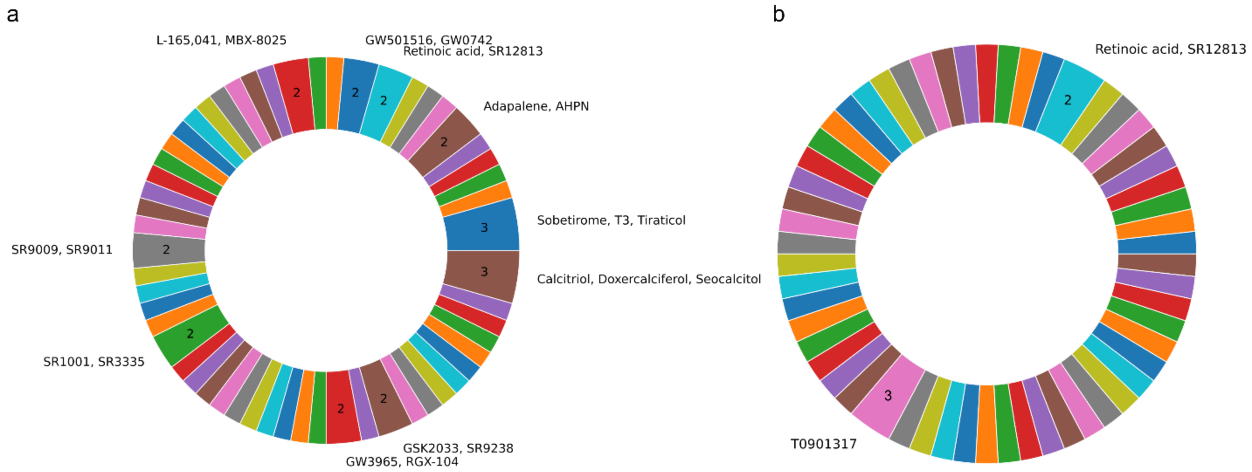

Supplementary Figure 5. Pie charts of skeleton distribution. (a) Compounds per skeleton in the CG set. (b) NR1 target subfamily per skeleton.

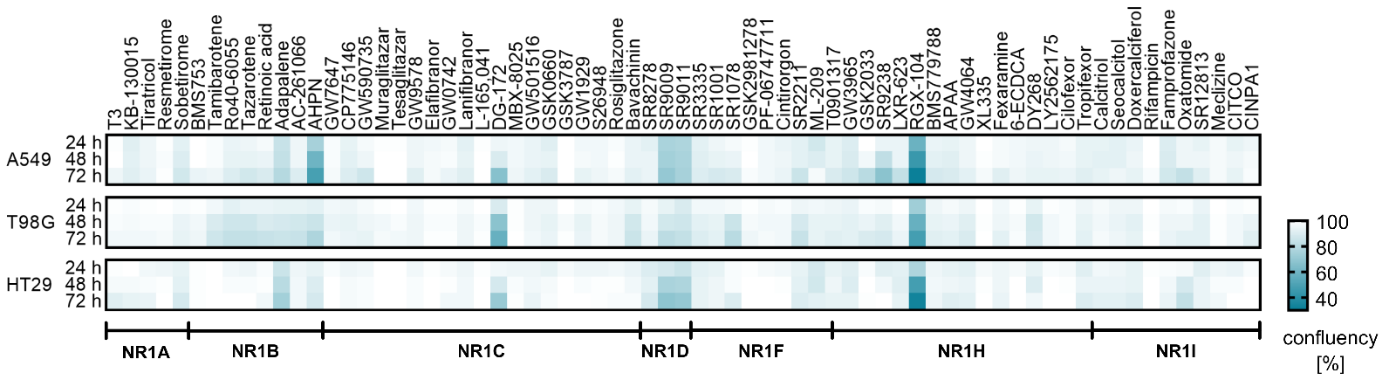

Supplementary Figure 6. Effects of the NR1 CG set on proliferation of A549, T98G and HT29 cells. The heatmaps show cell confluence over time (24 h, 48 h, 72 h) normalized to DMSO treated cells at the respective time point; n = 3.

Supplementary Table 1. Biochemical properties and recommended concentrations for phenotypic screening of the primary NR1 CG set, containing 80 chemically diverse and selective compounds.

Compound	Reference	Main NR target	Potency [µM] [rel. activity from literature]	Activity at recom. concentration in Gal4-hybrid reporter gene assay	Type	NR off-target	Recom. conc. or reason f. exclusion	NR1 set
**T3 (CAS# 6893-02-3)** 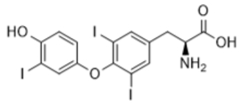	1,2	NR1A1 (THRα) NR1A2 (THRβ)	EC_{50} = 0.0024 ± 0.0005[x] (lit.) [100%] EC_{50} = 0.0024 ± 0.0005[x] (lit.) [100%]	364 ± 96[y] fold act. 704 ± 218[y] fold act.	Agonist	-	1 µM	Yes
**KB-130015 (CAS# 147030-48-6)** 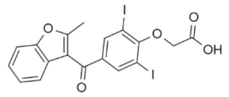	3	NR1A2 (THRβ)	IC_{50} = 2 ± 1[x]	69 ± 5%[y] rem. act.	Antagonist	-	3 µM	Yes
**Amiodarone (CAS# 19774-82-4)** 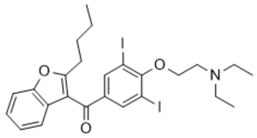	4	NR1A1 (THRα) NR1A2 (THRβ)	IC_{50} = 6 ± 4[x] IC_{50} = 11 ± 2[x]	89 ± 13%[y] rem. act. at 10 µM 86 ± 13%[y] rem. act. at 10 µM	Antagonist	-	Many off-targets outside NR family	No
**Tiratricol (CAS# 51-24-1)** 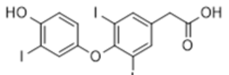	5	NR1A1 (THRα) NR1A2 (THRβ)	EC_{50} = 0.002[z] (lit.) [21% of T3] EC_{50} = 0.004[z] (lit.) [33% of T3]	557 ± 331[y] fold act. 1069 ± 632[y] fold act.	Agonist	NR5A1	1 µM	Yes
**Resmetirom (CAS# 920509-32-6)** 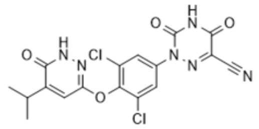	6	NR1A2 (THRβ)	EC_{50} = 0.2[z] (lit.) [84% of T3]	89 ± 14[y] fold act.	Agonist	-	10 µM	Yes
**Sobetirome (CAS# 211110-63-3)** 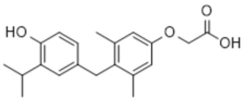	2	NR1A1 (THRα) NR1A2 (THRβ)	EC_{50} = 0.05[z] (lit.) [100% of T3] EC_{50} = 0.007[z] (lit) [100% of T3]	412 ± 33[y] fold act. 522 ± 23[y] fold act.	Agonist	-	1 µM	Yes
**BMS753 (CAS# 215307-86-1)** 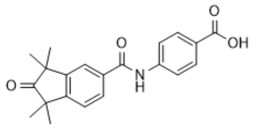	7	NR1B1 (RARα)	EC50 = 0.11 ± 0.04[x]	159 ± 55[y] fold act.	Agonist	-	1 µM	Yes
**Tamibarotene (CAS# 94497-51-5)** 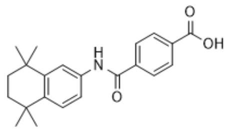	8	NR1B1 (RARα) NR1B2 (RARβ) NR1B3 (RARγ)	EC_{50} = 0.05[z] (lit.) EC_{50} = 0.2[z] (lit.) EC_{50} = 0.6[z] (lit.)	285 ± 48[y] fold act. 94 ± 9[y] fold act. 56 ± 46[y] fold act.	Agonist	-	1 µM	Yes
**Ro40-6055 (CAS# 102121-60-8)** 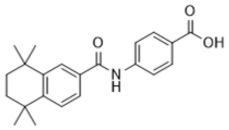	9	NR1B1 (RARα) NR1B2 (RARβ) NR1B3 (RARγ)	EC_{50} = 0.0003[z] (lit.) [43% of retinoic acid] EC_{50} = 0.009[z] (lit.) [46% of retinoic acid] EC_{50} = 0.01[z] (lit.) [43% of retinoic acid]	327 ± 42[y] fold act. 112 ± 15[y] fold act. 23 ± 5[y] fold act.	Agonist	-	1 µM	Yes

Compound	Reference	Main NR target	Potency [µM] [rel. activity from literature]	Activity at recom. concentration in Gal4-hybrid reporter gene assay	Type	NR off-target	Recom. conc. or reason f. exclusion	NR1 set
**Tazarotene (CAS# 118292-40-3)** 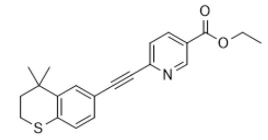	9	NR1B1 (RARα) NR1B2 (RARβ) NR1B3 (RARγ)	$EC_{50} = 0.06^{z}$ (lit.) [54% of retinoic acid] $EC_{50} = 0.0008^{z}$ (lit.) [57% of retinoic acid] $EC_{50} = 0.04^{z}$ (lit.) [72% of retinoic acid]	224 ± 30^{y} fold act. 107 ± 7^{y} fold act. 18 ± 4^{y} fold act.	Agonist	-	1 µM	Yes
**Retinoic acid (CAS# 302-79-4)** 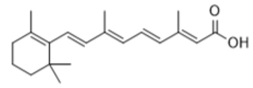	10	NR1B1 (RARα) NR1B2 (RARβ) NR1B3 (RARγ)	$EC_{50} = 0.4 \pm 0.1^{x}$ $EC_{50} = 0.36 \pm 0.05^{x}$ $EC_{50} = 0.34 \pm 0.04^{x}$	214 ± 87^{y} fold act. 140 ± 32^{y} fold act. 147 ± 20^{y} fold act.	Agonist	NR2B1 NR1F1 NR1F2 NR1F3 (lit. [11]) NR2E1 NR5A1	1 µM	Yes
**Adapalene (CAS# 106685-40-9)** 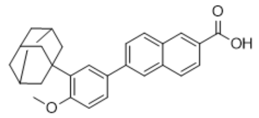	12	NR1B1 (RARα) NR1B2 (RARβ) NR1B3 (RARγ)	$EC_{50} = 0.02^{z}$ (lit.) $EC_{50} = 0.002^{z}$ (lit.) $EC_{50} = 0.003^{z}$ (lit.)	238 ± 49^{y} fold act. 97 ± 9^{y} fold act. 30 ± 8^{y} fold act.	Agonist	-	1 µM	Yes
**AC-261066 (CAS# 870773-76-5)** 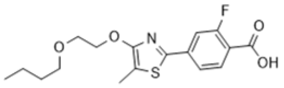	13, 14	NR1B1 (RARα) NR1B2 (RARβ) NR1B3 (RARγ)	$EC_{50} = 0.6 \pm 0.3^{y}$ (lit.) [38% of retinoic acid] $EC_{50} = 0.010 \pm 0.008^{y}$ (lit.) [76% of retinoic acid] $EC_{50} = 0.5 \pm 0.1^{y}$ (lit.) [41% of retinoic acid]	153 ± 15^{y} fold act. 84 ± 13^{y} fold act. 17 ± 5^{y} fold act.	Agonist	NR2B1	1 µM	Yes
**AHPN (CAS# 125316-60-1)** 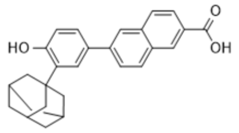	12	NR1B2 (RARβ) NR1B3 (RARγ)	$EC_{50} = 0.2^{z}$ (lit.) $EC_{50} = 0.05^{z}$ (lit.)	25 ± 6^{y} fold act. 21 ± 5^{y} fold act.	Agonist	NR2C1 NR2E1	1 µM	Yes
**GW7647 (CAS# 265129-71-3)** 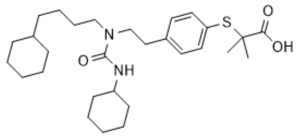	15	NR1C1 (PPARα) NR1C3 (PPARγ)	$EC_{50} = 0.006^{z}$ (lit.) $EC_{50} = 1^{z}$ (lit.)	38 ± 18^{y} fold act. 46 ± 2^{y} fold act.	Agonist	NR1C2 (lit. [15])	1 µM	Yes
**CP775146 (CAS# 702680-17-9)** 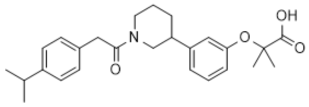	16	NR1C1 (PPARα)	$EC_{50} = 0.06 \pm 0.04^{x}$ (lit.)	33 ± 2^{y} fold act.	Agonist	-	1 µM	Yes
**GW590735 (CAS# 622402-22-6)** 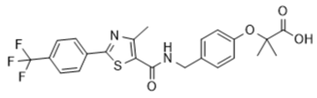	17, 18	NR1C1 (PPARα) NR1C2 (PPARδ)	$EC_{50} = 0.004 \pm 0.002^{y}$ (lit.) [100%] $EC_{50} = 0.16 \pm 0.03^{x}$ [82% of GW501516]	19 ± 4^{y} fold act. 25 ± 5^{y} fold act.	Agonist	-	1 µM	Yes
**Muraglitazar (CAS# 331741-94-7)** 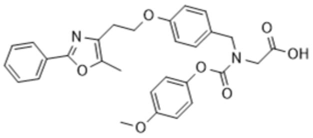	19	NR1C1 (PPARα) NR1C3 (PPARγ)	$EC_{50} = 0.05 \pm 0.03^{x}$ [70% of GW2331] $EC_{50} = 0.016 \pm 0.002^{x}$ [82% of rosiglitazone]	13 ± 7^{y} fold act. 115 ± 12^{y} fold act.	Agonist	NR1C2	1 µM	Yes
**Tesaglitazar (CAS# 251565-85-2)** 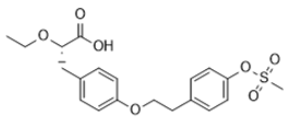	20	NR1C1 (PPARα) NR1C3 (PPARγ)	$EC_{50} = 0.29 \pm 0.07^{x}$ $EC_{50} = 0.127 \pm 0.009^{x}$	29 ± 14^{y} fold act. 109 ± 5^{y} fold act.	Agonist	-	1 µM	Yes

Compound	Reference	Main NR target	Potency [µM] [rel. activity from literature]	Activity at recom. concentration in Gal4-hybrid reporter gene assay	Type	NR off-target	Recom. conc. or reason f. exclusion	NR1 set
**GW9578 (CAS# 247923-29-1)** 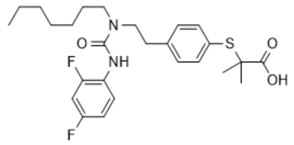	21	NR1C1 (PPARα)<br>NR1C3 (PPARγ)	EC_{50} = 0.05^{z} (lit.)<br>EC_{50} = 1^{z} (lit.)	36 ± 6^{y} fold act.<br>80 ± 8^{y} fold act.	Agonist	NR1C2 (lit. [21])	1 µM	Yes
**Oleoyl Ethanolamide (CAS# 111-58-0)** 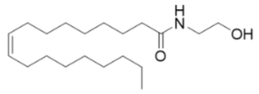	22	NR1C1 (PPARα)	EC_{50} = 0.120 ± 0.001^{x} (lit.)	1.1 ± 0.2^{y} fold act. at 30 µM	Agonist	NR1C2 (lit. [22])	No on-target activity ≤ 30 µM	No
**Elafibranor (CAS# 923978-27-2)** 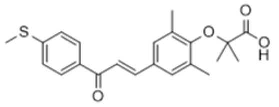	23	NR1C1 (PPARα)<br>NR1C2 (PPARδ)<br>NR1C3 (PPARγ)	EC_{50} = 0.13 ± 0.02^{x}<br>[117% of GW7647]<br>EC_{50} = 0.21 ± 0.04^{x}<br>[72% of L-165,041]<br>EC_{50} = 0.41 ± 0.07^{x}<br>[93% of troglitazone]	17.6 ± 0.8^{y} fold act.<br>12 ± 2^{y} fold act.<br>23 ± 5^{y} fold act.	Agonist	-	1 µM	Yes
**GW0742 (CAS# 317318-84-6)** 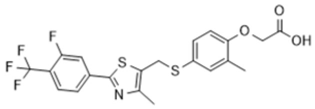	24	NR1C2 (PPARδ)	EC_{50} = 0.001 ± 0.002^{x} (lit.)	52 ± 5^{y} fold act.	Agonist	NR1C1<br>NR1C3<br>(lit. [24])	1 µM	Yes
**Lanifibranor (CAS# 927961-18-0)** 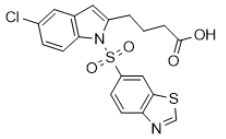	25	NR1C2 (PPARδ)<br>NR1C3 (PPARγ)	EC_{50} = 0.9 ± 0.2^{z} (lit.)<br>[105% of GW501516]<br>EC_{50} = 0.21 ± 0.07^{z} (lit.)<br>[79% of rosiglitazone]	13 ± 2^{y} fold act.<br>35 ± 11^{y} fold act.	Agonist	NR1C1 (lit. [25])	1 µM	Yes
**L-165,041 (CAS# 79558-09-1)** 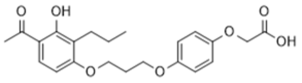	26	NR1C1 (PPARα)<br>NR1C2 (PPARδ)<br>NR1C3 (PPARγ)	EC_{50} = 1^{z} (lit.)<br>[97% of WY-14643]<br>EC_{50} = 0.015 ± 0.006^{x}<br>[51% of MWI66]<br>EC_{50} = 1.0 ± 0.1^{x}<br>[79% of rosiglitazone]	28 ± 13^{y} fold act.<br>50 ± 8^{y} fold act.<br>33 ± 6^{y} fold act.	Agonist	-	1 µM	Yes
**DG-172 (CAS# 1361504-77-9)** 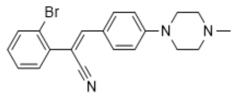	27	NR1C2 (PPARδ)	IC_{50} = 1.8 ± 0.3^{x}	8.0 ± 0.4%^{y} rem. act.	Antagonist	-	10 µM	Yes
**MBX-8025 (CAS# 851528-79-5)** 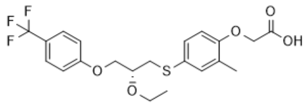	28	NR1C2 (PPARδ)	EC_{50} = 0.002^{z} (lit.)<br>[105% of GW501516]	45 ± 5^{y} fold act.	Agonist	-	1 µM	Yes
**GW501516 (CAS# 317318-70-0)** 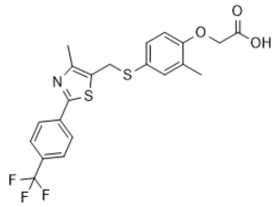	29	NR1C2 (PPARδ)	EC_{50} = 0.0012 ± 0.0001^{x} (lit.)	63 ± 27^{y} fold act.	Agonist	-	1 µM	Yes
**GSK0660 (CAS# 1014691-61-2)** 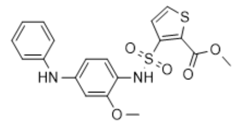	30	NR1C1 (PPARα)<br>NR1C2 (PPARδ)<br>NR1C3 (PPARγ)	IC_{50} = 6.1 ± 0.6^{x}<br>IC_{50} = 5.5 ± 0.7^{x}<br>[0% rem. act. of GW501516]<br>IC_{50} = 7.6 ± 0.3^{x}	14 ± 1%^{y} rem. act.<br>33 ± 12%^{y} rem. act.<br>31 ± 3%^{y} rem. act.	Antagonist	-	10 µM	Yes

Compound	Reference	Main NR target	Potency [µM] [rel. activity from literature]	Activity at recom. concentration in Gal4-hybrid reporter gene assay	Type	NR off-target	Recom. conc. or reason f. exclusion	NR1 set
**GSK3787 (CAS# 188591-46-0)** 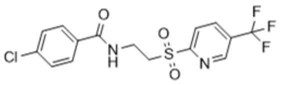	31	NR1C2 (PPARδ)	IC_{50} = 0.1[z] (lit.) [0% rem. act. of GW501516]	74 ± 14%[y] rem. act.	Antagonist	NR1C3	1 µM	Yes
**GW1929 (CAS# 196808-24-9)** 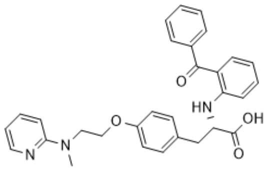	32	NR1C3 (PPARγ)	EC_{50} = 0.006[z] (lit.) [72% of rosiglitazone]	115 ± 35[y] fold act.	Agonist	-	1 µM	Yes
**S26948 (CAS# 353280-43-0)** 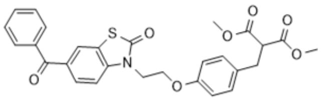	33	NR1C3 (PPARγ)	EC_{50} = 0.042 ± 0.008[x]	137 ± 5[y] fold act.	Agonist	-	1 µM	Yes
**Rosiglitazone (CAS# 122320-73-4)** 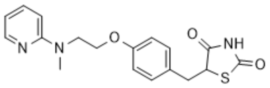	34	NR1C3 (PPARγ)	EC_{50} = 0.035 ± 0.003[z] (lit.)	124 ± 21[y] fold act.	Agonist	-	1 µM	Yes
**Bavachinin (CAS# 19879-30-2)** 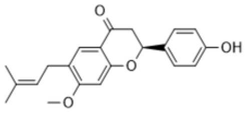	35	NR1C3 (PPARγ)	EC_{50} = 0.7[z] (lit.)	2.1 ± 0.2[y] fold act.	Agonist	-	1 µM	Yes
**LG101506 (CAS# 331248-11-4)** 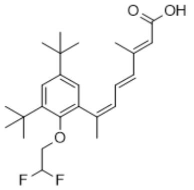	36	NR1C3 (PPARγ)	EC_{50} = 0.003 ± 0.001[z] (lit.)	1.16 ± 0.05[y] fold act. at 3 µM	Agonist	-	No on-target activity ≤ 3 µM	No
**SR8278 (CAS# 1254944-66-5)** 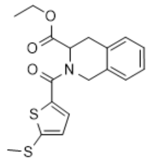	37	NR1D1 (revERBα)	IC_{50} = 0.5[z] (lit.)	161 ± 26% rel. act. to SR9011	Antagonist	NR4A1	10 µM	Yes
**GSK4112 (CAS# 1216744-19-2)** 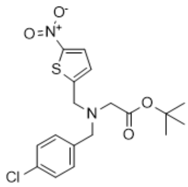	38	NR1D1 (revERBα)	EC_{50} = 30 ± 3[x] [0.82 fold act. at 10 µM]	0.6 ± 0.1[y] fold act. at 30 µM	Agonist	-	EC_{50} is above criteria (≤ 10 µM)	No
**SR9009 (CAS# 1379686-30-2)** 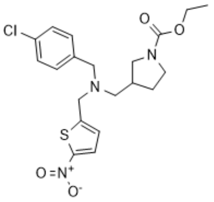	39	NR1D1 (revERBα) NR1D2 (revERBβ)	EC_{50} = 6.0 ± 0.8[x] EC_{50} = 11 ± 2[x]	0.4 ± 0.2[y] fold act. 0.5 ± 0.2[y] fold act.	Agonist	NR2C1	10 µM	Yes

Compound	Reference	Main NR target	Potency [µM] [rel. activity from literature]	Activity at recom. concentration in Gal4-hybrid reporter gene assay	Type	NR off-target	Recom. conc. or reason f. exclusion	NR1 set
**SR9011 (CAS# 1379686-29-9)**<br>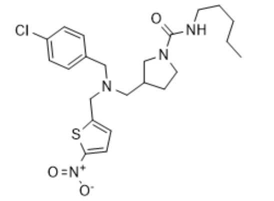	39	NR1D1 (revERBα)<br>NR1D2 (revERBβ)	EC_{50} = 2 ± 1[x]<br>EC_{50} = 3.1 ± 0.6[x]	0.3 ± 0.2[y] fold act.<br>0.2 ± 0.1[y] fold act.	Agonist	NR2C1<br>NR2E1	10 µM	Yes
**SR3335 (CAS# 293753-05-6)**<br>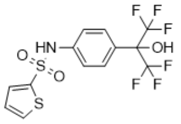	40	NR1F1 (RORα)<br>NR1F2 (RORβ)<br>NR1F3 (RORγ)	IC_{50} = 0.5[z] (lit.)<br>IC_{50} = 1.5 ± 0.3[x]<br>IC_{50} = 3.2 ± 0.6[x]	36 ± 1%[y] rem. act.<br>26 ± 12%[y] rem. act.<br>19 ± 5%[y] rem. act.	inv. Agonist	-	10 µM	Yes
**SR1001 (CAS# 1335106-03-0)**<br>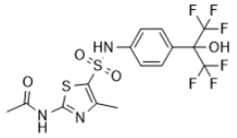	41	NR1F1 (RORα)<br>NR1F2 (RORβ)<br>NR1F3 (RORγ)	IC_{50} = 0.9 ± 0.2[x]<br>IC_{50} = 1.1 ± 0.5[x]<br>IC_{50} = 0.11 ± 0.02[x]	59 ± 12%[y] rem. act.<br>58 ± 15%[y] rem. act.<br>7 ± 4%[y] rem. act.	inv. Agonist	-	1 µM	Yes
**SR1078 (CAS# 1246525-60-9)**<br>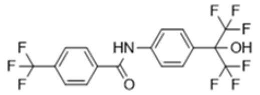	42	NR1F3 (RORγ)	EC_{50} = 2[z] (lit.)	1.2 ± 0.1[y] fold act.	Agonist	-	1 µM	Yes
**GSK2981278 (CAS# 1474110-21-8)**<br>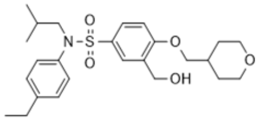	43	NR1F3 (RORγ)	IC_{50} = 0.02[z] (lit.)	1.0 ± 0.4%[y] rem. act.	inv. Agonist	-	1 µM	Yes
**PF-06747711 (CAS# 1892576-58-7)**<br>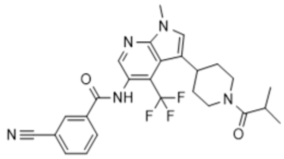	44	NR1F3 (RORγ)	IC_{50} = 0.004[z] (lit.)	0.6 ± 0.3%[y] rem. act.	inv. Agonist	-	1 µM	Yes
**Cintirorgon (CAS# 2055536-64-4)**<br>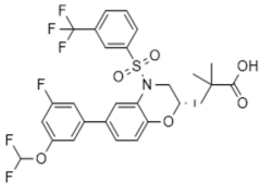	45	NR1F3 (RORγ)	EC_{50} = 0.021 ± 0.009[x]	2.4 ± 0.3[y] fold act.	Agonist	-	1 µM	Yes
**SR2211 (CAS# 1359164-11-6)**<br>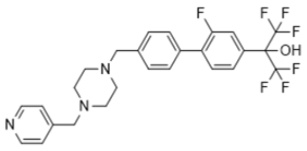	46	NR1F3 (RORγ)	IC_{50} = 0.3[z] (lit.)<br>[5% rem. act. of DMSO control at 10 µM]	1.4 ± 0.1%[y] rem. act.	inv. Agonist	-	1 µM	Yes
**XY018 (CAS# 1873358-87-2)**<br>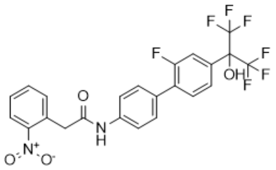	47	NR1F3 (RORγ)	IC_{50} = 0.2[z] (lit.)	89 ± 12%[y] rem. act. at 3 µM	inv. Agonist	-	No on-target activity ≤ 3 µM; toxic ≥ 10 µM	No

Compound	Reference	Main NR target	Potency [µM] [rel. activity from literature]	Activity at recom. concentration in Gal4-hybrid reporter gene assay	Type	NR off-target	Recom. conc. or reason f. exclusion	NR1 set
**ML-209 (CAS# 1334526-14-5)** 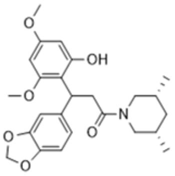	48	NR1F2 (RORβ) NR1F3 (RORγ)	IC_{50} = 1.3 ± 0.1[x] IC_{50} = 0.20 ± 0.02[x]	14.1 ± 0.4%[y] rem. act. 4.1 ± 0.3%[y] rem. act.	inv. Agonist	NR1F1 NR2C1 (@ 10µM)	10µM (NR1F2 NR1F3) 1 µM (NR1F3)	Yes
**T0901317 (CAS# 293754-55-9)** 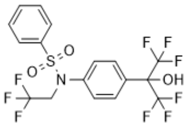	49	NR1F3 (RORγ) NR1H3 (LXRα) NR1H2 (LXRβ) NR1I2 (PXR)	IC_{50} = 0.5[z] (lit.) EC_{50} = 0.4[z] (lit.) EC_{50} = 0.2[z] (lit.) EC_{50} = 0.04[z] (lit.)	2.0 ± 0.4%[y] rem. act. 70 ± 32[y] fold act. 84 ± 35[y] fold act. 2.0 ± 0.1[y] fold act.	inv. Agonist, Agonist	NR1H4 (lit. [49])	1 µM	Yes
**GW3965 (CAS# 405911-17-3)** 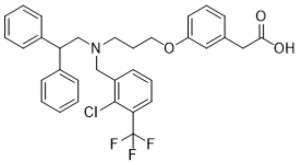	50	NR1H3 (LXRα) NR1H2 (LXRβ)	EC_{50} = 0.19 ± 0.03[x] (lit.) [170% of 24(S),25-epoxycholesterol] EC_{50} = 2.1 ± 0.5[x]	34 ± 12[y] fold act. 18 ± 6[y] fold act.	Agonist	-	3 µM	Yes
**GSK2033 (CAS# 1221277-90-2)** 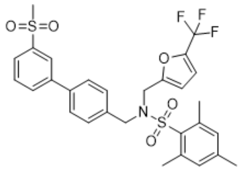	51	NR1H3 (LXRα) NR1H2 (LXRβ)	IC_{50} = 0.1[z] (lit.) IC_{50} = 0.04[z] (lit.)	28 ± 3%[y] rem. act. 18.0 ± 0.7%[y] rem. act.	Antagonist	-	3 µM	Yes
**SR9238 (CAS# 1416153-62-2)** 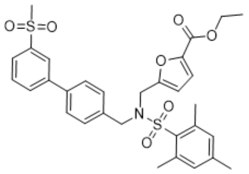	52	NR1H3 (LXRα) NR1H2 (LXRβ)	IC_{50} = 0.2[z] (lit.) IC_{50} = 0.04[z] (lit.)	9 ± 1%[y] rem. act. 6.3 ± 0.6%[y] rem. act.	Antagonist	-	1 µM	Yes
**IMB-808 (CAS# 870768-70-0)** 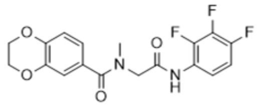	53	NR1H3 (LXRα) NR1H2 (LXRβ)	EC_{50} = 0.2[z] (lit.) EC_{50} = 0.5[z] (lit.)	0.9 ± 0.1[y] fold act. at 10 µM 0.7 ± 0.1[y] fold act. at 10 µM	Agonist	-	No on-target activity ≤ 10 µM	No
**LXR-623 (CAS# 875787-07-8)** 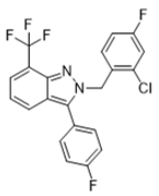	54	NR1H3 (LXRα) NR1H2 (LXRβ)	EC_{50} = 7[z] (lit.) [53% of T0901317] EC_{50} = 4[z] (lit.) [73% of T0901317]	20 ± 11[y] fold act. 22 ± 5[y] fold act.	Agonist	NR1I2 (lit. [54]) NR2E1	3 µM	Yes
**25(R)-27-hydroxy Cholesterol (CAS# 20380-11-4)** 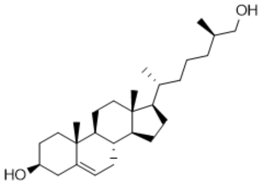	55	NR1H3 (LXRα) NR1H2 (LXRβ)	EC_{50} = 0.09[z] (lit.) EC_{50} = 0.07[z] (lit.)	0.9 ± 0.2[y] fold act. at 10 µM 0.8 ± 0.2[y] fold act. at 10 µM	Agonist	-	No on-target activity ≤ 10 µM	No

Compound	Reference	Main NR target	Potency [µM] [rel. activity from literature]	Activity at recom. concentration in Gal4-hybrid reporter gene assay	Type	NR off-target	Recom. conc. or reason f. exclusion	NR1 set
**RGX-104 (CAS# 610318-03-1)** 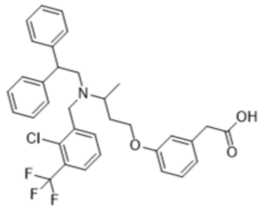	56	NR1H3 (LXRα) NR1H2 (LXRβ)	EC_{50} = 3.3 ± 0.3^{x} [91% of GSK lead 2] EC_{50} = 2.9 ± 0.6^{x} [80% of GSK lead 2]	54 ± 10^{y} fold act. 25 ± 5^{y} fold act.	Agonist	-	10 µM	Yes
**BMS779788 (CAS# 918348-67-1)** 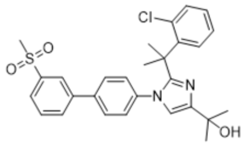	57, 58	NR1H3 (LXRα) NR1H2 (LXRβ)	EC_{50} = 0.2^{z} (lit.) [38% of lead 3b] EC_{50} = 0.3^{z} (lit.) [72% of lead 3b]	7.4 ± 0.9^{y} fold act. 11 ± 3^{y} fold act.	Agonist	-	1 µM	Yes
**APAA (CAS# 344327-48-6)** 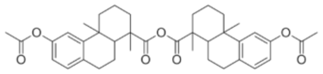	59	NR1H3 (LXRα) NR1H2 (LXRβ)	EC_{50} = 0.001^{z} (lit.) EC_{50} = 0.001^{z} (lit.)	709 ± 337^{y} fold act. 375 ± 235^{y} fold act.	Agonist	-	1 µM	Yes
**GW4064 (CAS# 278779-30-9)** 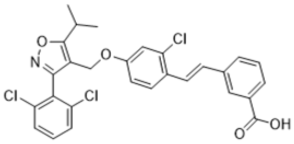	60, 61	NR1H4 (FXR)	EC_{50} = 0.37 ± 0.03^{x} [100%]	308 ± 49^{y} fold act.	Agonist	-	1 µM	Yes
**XL335 (CAS# 629664-81-9)** 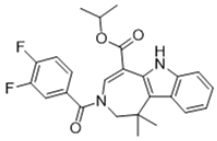	62, 63	NR1H4 (FXR)	EC_{50} = 0.099 ± 0.009^{x} [130% of GW4064]	311 ± 54^{y} fold act.	Agonist	-	1 µM	Yes
**Fexaramine (CAS# 574013-66-4)** 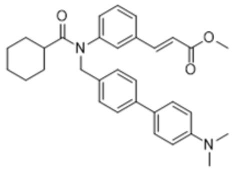	64, 65	NR1H4 (FXR)	EC_{50} = 0.96 ± 0.05^{x}	32 ± 4^{y} fold act.	Agonist	-	1 µM	Yes
**6-ECDCA (CAS# 459789-99-2)** 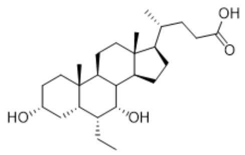	66	NR1H4 (FXR)	EC_{50} = 0.30 ± 0.02^{x}	389 ± 57^{y} fold act.	Agonist	-	1 µM	Yes
**DY268 (CAS# 1609564-75-1)** 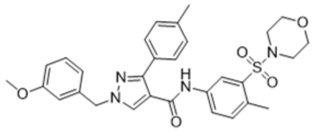	67	NR1H4 (FXR)	IC_{50} = 0.58 ± 0.03^{x} [1% rem. act. of 400 nM GW4064]	28 ± 15%^{y} rem. act.	Antagonist	NR1H2 NR1H3 NR2E1	1 µM	Yes
**LY2562175 (CAS# 1103500-20-4)** 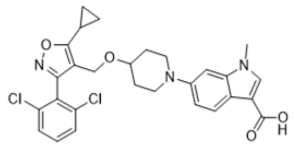	68	NR1H4 (FXR)	EC_{50} = 0.2^{z} (lit.) [41% of GW4064]	185 ± 28^{y} fold act.	Agonist	-	1 µM	Yes

Compound	Reference	Main NR target	Potency [µM] [rel. activity from literature]	Activity at recom. concentration in Gal4-hybrid reporter gene assay	Type	NR off-target	Recom. conc. or reason f. exclusion	NR1 set
**Cilofexor (CAS# 1418274-28-8)** 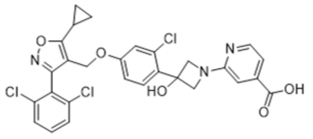	69	NR1H4 (FXR)	EC_{50} = 0.04[z] (lit.)	179 ± 47[y] fold act.	Agonist	-	1 µM	Yes
**Tropifexor (CAS# 1383816-29-2)** 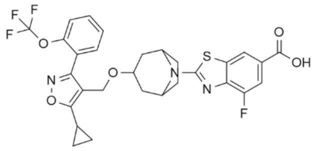	70	NR1H4 (FXR)	EC_{50} = 0.0003[z] (lit.) [89% of GW4064]	216 ± 15[y] fold act.	Agonist	-	1 µM	Yes
**Calcitriol (CAS# 32222-06-3)** 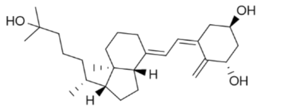	71	NR1I1 (VDR)	EC_{50} = 0.10 ± 0.03[x]	89 ± 24[y] fold act.	Agonist	-	1 µM	Yes
**Seocalcitol (CAS# 134404-52-7)** 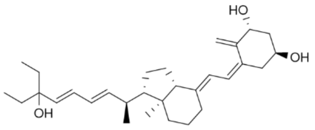	72	NR1I1 (VDR)	EC_{50} = 0.11 ± 0.02[x]	74 ± 19[y] fold act.	Agonist	-	1 µM	Yes
**Doxercalciferol (CAS# 54573-75-0)** 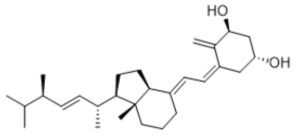	73	NR1I1 (VDR)	EC_{50} = 0.96 ± 0.07[x]	42 ± 2[y] fold act.	Agonist	-	1 µM	Yes
**Rifampicin (CAS# 13292-46-1)** 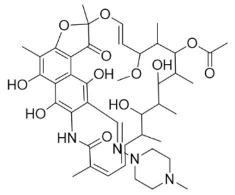	74	NR1I2 (PXR)	EC_{50} = 1[z] (lit.) [75% of T0901317]	3 ± 1[y] fold act.	Agonist	-	10 µM	Yes
**Famprofazone (CAS# 22881-35-2)** 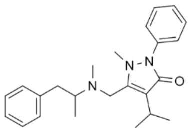	75	NR1I2 (PXR)	EC_{50} = 0.13 ± 0.4[x]	1.8 ± 0.5[y] fold act.	Agonist	-	1 µM	Yes
**Oxatomide (CAS# 60607-34-3)** 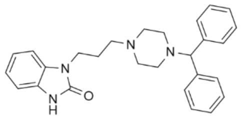	75	NR1I2 (PXR)	EC_{50} = 6 ± 3[x]	1.9 ± 0.4[y] fold act.	Agonist	-	10 µM	Yes
**Pleconaril (CAS# 153168-05-9)** 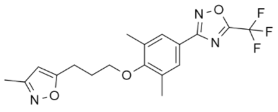	76	NR1I2 (PXR)	EC_{50} = 0.3[z] (lit.) [88% of lead]	1.1 ± 0.2[y] fold act. at 10 µM	Agonist	-	No on-target activity ≤ 10 µM	No

Compound	Reference	Main NR target	Potency [µM] [rel. activity from literature]	Activity at recom. concentration in Gal4-hybrid reporter gene assay	Type	NR off-target	Recom. conc. or reason f. exclusion	NR1 set
**Lovastatin (CAS# 75330-75-5)** 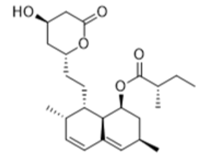	77	NR1I2 (PXR)	EC_{50} = 2^{z} (lit.)	1.5 ± 0.1^{y} fold act. at 10 µM	Agonist	-	Non-specific effects; many off-targets outside NR family	No
**SR12813 (CAS# 126411-39-0)** 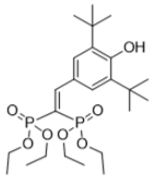	78	NR1I2 (PXR)	EC_{50} = 0.14 ± 0.05^{z} (lit.)	1.5 ± 0.4^{y} fold act.	Agonist	-	1 µM	Yes
**PK11195 (CAS# 85532-75-8)** 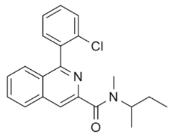	79	NR1I3 (CAR)	IC_{50} = 0.4^{z} (lit.)	36 ± 8%^{y} rem. act. at 10 µM	inv. Agonist	NR2C1 NR2E1 NR5A1	Non-specific effects	No
**Meclizine (CAS# 1104-22-9)** 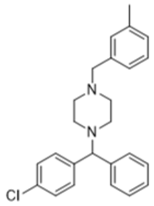	80	NR1I3 (CAR)	IC_{50} = 0.06^{z} (lit.)	5.8 ± 0.8%^{y} rem. act.	inv. Agonist	-	1 µM	Yes
**CITCO (CAS# 338404-52-7)** 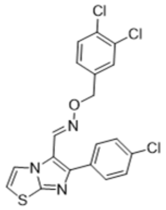	81	NR1I3 (CAR)	EC_{50} = 0.03^{z} (lit.)	23 ± 20^{y} fold act.	Agonist	-	1 µM	Yes
**Clotrimazole (CAS# 23593-75-1)** 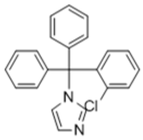	82	NR1I3 (CAR)	IC_{50} = 0.7^{z} (lit.)	1.14 ± 0.07^{y} fold act. at 1 µM	inv. Agonist	-	No on-target activity ≤ 1 µM; toxic ≥ 3 µM	No
**CINPA1 (CAS# 102636-74-8)** 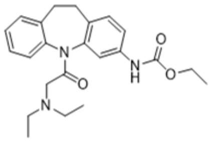	79	NR1I3 (CAR)	IC_{50} = 0.69 ± 0.07^{z} (lit.)	19 ± 2%^{y} rem. act.	Antagonist	-	10 µM	Yes

^{x} mean ± S.E.M.
^{y} mean ± SD
^{z} information on error or error type not available

Supplementary Table 2. Off-targets for amiodarone and lovastatin with annotated bioactivity values ≤ 10 µM in ChEMBL. Data from previously published dataset[83].

Compound	Off-targets ≤ 10 µM	p(Potency)	Compound	Off-targets ≤ 10 µM	p(Potency)
Amiodarone	SMN1	5.3		NFKB1	5.5
	MEN1	5.5		CYP1A2/2C8/2C9/3A4	5.2/5.1/7.7/5.2
	CHRM1/M2/M3/M4/M5	5.9/5.5/5.8/5.8/5.9		CCR2	5.2
	ERBB2	5.0		MAPK1	5.0
	HTR2A/2B/2C	5.7/6.1/6.5		SIRT3	5.5
	FYN	6.1		MC5R	5.0
	ADRA2A/2B	6.7/5.1		EBP	7.6
	TSHR	5.2	Lovastatin	HMGCR	7.7
	DRD1/D3	5.2/6.2		LMNA	5.1
	CACNA1A	6.1		SLC6A2/A3	5.1/5.0
	SLC6A2	5.6		CYP3A4	5.0
	BDKRB2	5.0			

Supplementary Table 3. Constructs and sequences of liability panel proteins[84].

Construct ID	Protein Sequence (without tag)	Construct DNA Sequence
ABL1A-c006	SMSPNYDKWEMERTDITMKHKLGGGQY<br>GEVYEGVWKKYSLTVAVKTLKEDTMEVEE<br>FLKEAAVMKEIKHPNLVQLLGVCTREPPF<br>YIITEFMTYGNLLDYLRECNRQEVNAVVL<br>LYMATQISSAMEYLEKKNFIHRDLAARNC<br>LVGENHLVKVADFGLSRLMTGDTYTAHA<br>GAKFPIKWTAPESLAYNKFSIKSDVWAFG<br>VLLWEIATYGMSPYPGIDLSQVYELLEKD<br>YRMERPEGCPEKVYELMRACWQWNPS<br>DRPSFAEIHQAFETMFQESSISDEVEKEL<br>GK	CATATGCACCATCATCATCATCATTCTTCTGGTGTAGATCTGGGTACCGAGAACCTGTACTTCCAATCCATGTCCCCCAACTACGACAAGTGG<br>GAGATGGAACGCACGGACATCACCATGAAGCACAAGCTGGGCGGGGGCCAGTACGGGGAGGTGTACGAGGGCGTGTGGAAGAAATAC<br>AGCCTGACGGTGGCCGTGAAGACCTTGAAGGAGGACACCATGGAGGTGGAAGAGTTCTTGAAAGAAGCTGCAGTCATGAAAGAGATCAA<br>ACACCCTAACCTGGTGCAGCTCCTTGGGGTCTGCACCCGGGAGCCCCCGTTCTATATCATCACTGAGTTCATGACCTACGGGAACCTCCT<br>GGACTACCTGAGGGAGTGCAACCGGCAGGAGGTGAACGCCGTGGTGCTGCTGTACATGGCCACTCAGATCTCGTCAGCCATGGAGTAC<br>CTGGAGAAGAAAAACTTCATCCACAGAGATCTTGCTGCCCGAAACTGCCTGGTAGGGGAGAACCACTTGGTGAAGGTAGCTGATTTTGGC<br>CTGAGCAGGTTGATGACAGGGGACACCTACACAGCCCATGCTGGAGCCAAGTTCCCCATCAAATGGACTGCACCCGAGAGCCTGGCCT<br>ACAACAAGTTCTCCATCAAGTCCGACGTCTGGGCATTTGGAGTATTGCTTTGGGAAATTGCTACCTATGGCATGTCCCCTTACCCGGGAATT<br>GACCTGTCCCAGGTGTATGAGCTGCTAGAGAAGGACTACCGCATGGAGCGCCCAGAAGGCTGCCCAGAGAAGGTCTATGAACTCATGCG<br>AGCATGTTGGCAGTGGAATCCCTCTGACCGGCCCTCCTTTGCTGAAATCCACCAAGCCTTTGAAACAATGTTCCAGGAATCCAGTATCTCA<br>GACGAAGTGGAAAAGGAGCTGGGGAAATGACAGTAAAGGTGGATACGGATCCGAA
CSNK1DA-c010	SMMELRVGNRYRLGRKIGSGSFGDIYLG<br>TDIAAGEEVAIKLECVKTKHPQLHIESKIYK<br>MMQGGVGIPTIRWCGAEGDYNVMVME<br>LLGPSLEDLFNFCSRKFSLKTVLLLADQM<br>ISRIEYIHSKNFIHRDVKPDNFLMGLGKK<br>GNLVYIIDFGLAKKYRDARTHQHIPYREN<br>KNLTGTARYASINTHLGIEQSRRDDLESL<br>GYVLMYFNLGSLPWQGLKAATKRQKYER<br>ISEKKMSTPIEVLCKGYPSEFATYLNFCRS<br>LRFDDKPDYSYLRQLFRNLFHRQGFSYD<br>YVFDWNMLK	CATATGCACCATCATCATCATCATTCTTCTGGTGTAGATCTGGGTACCGAGAACCTGTACTTCCAATCCATGATGGAACTGCGTGTGGGCAAC<br>CGCTATCGTCTGGGTCGTAAAATCGGCTCGGGCTCGTTTGGTGACATCTATCTGGGCACGGACATCGCGGCCGGCGAAGAAGTCGCGAT<br>CAAACTGGAATGCGTGAAAACGAAACATCCGCAGCTGCACATCGAAAGCAAAATCTACAAAATGATGCAAGGCGGTGTGGGCATTCCGAC<br>CATCCGTTGGTGCGGCGCCGAGGGTGATTACAATGTGATGGTTATGGAACTGCTGGGTCCGAGCCTGGAAGACCTGTTTAACTTCTGTTCG<br>CGCAAATTCAGCCTGAAAACCGTTCTGCTGCTGGCAGATCAGATGATTTCGCGTATCGAATACATCCATAGCAAAAACTTCATCCACCGCGA<br>TGTGAAACCGGACAATTTTCTGATGGGCCTGGGTAAAAAAGGCAACCTGGTTTATATTATCGATTTTGGTCTGGCGAAAAAATACCGTGACGC<br>CCGCACGCATCAGCACATTCCGTATCGTGAAAACAAAAATCTGACCGGCACGGCACGCTACGCTTCCATCAATACCCATCTGGGTATTGA<br>ACAATCTCGTCGCGATGACCTGGAAAGTCTGGGCTATGTCCTGATGTACTTCAACCTGGGCAGTCTGCCGTGGCAGGGTCTGAAAGCAGC<br>TACCAAACGTCAAAAATACGAACGCATCTCTGAAAAGAAAATGAGTACGCCGATTGAAGTGCTGTGCAAAGGTTATCCGTCTGAATTTGCGA<br>CCTACCTGAATTTCTGTCGTTCCCTGCGCTTTGATGACAAACCGGATTATTCATACCTGCGTCAGCTGTTTCGCAACCTGTTTCATCGTCAAG<br>GCTTCTCGTATGATTACGTGTTTGACTGGAACATGCTGAAATGACAGTAAAGGTGGATACGGATCCGAA
MAPK1A-c001	SMAAAAAAGAGPEMVRGQVFDVGPRYT<br>NLSYIGEGAYGMVCSAYDNVNKVRVAIKK<br>ISPFEHQTYCQRTLREIKILLRFRHENIIGI<br>NDIIRAPTIEQMKDVYIVQDLMETDLYKLL<br>KTQHLSNDHICYFLYQILRGLKYIHSANVL<br>HRDLKPSNLLLNTTCDLKICDFGLARVAD<br>PDHDHTGFLTEYVATRWYRAPEIMLNSK<br>GYTKSIDIWSVGCILAEMLSNRPIFPGKH<br>YLDQLNHILGILGSPSQEDLNCIINLKAR<br>NYLLSLPHKNKVPWNRLFPNADSKALDL<br>LDKMLTFNPHKRIEVEQALAHPYLEQYYD<br>PSDEPIAEAPFKFDMELDDLPKEKLKELIF<br>EETARFQPGYRS	CATATGCACCATCATCATCATCATTCTTCTGGTGTAGATCTGGGTACCGAGAACCTGTACTTCCAATCCATGGCGGCGGCGGCGGCGGCG<br>GGCGCGGGCCCGGAGATGGTCCGCGGGCAGGTGTTCGACGTGGGGCCGCGCTACACCAACCTCTCGTACATCGGCGAGGGCGCCT<br>ACGGCATGGTGTGCTCTGCTTATGATAATGTCAACAAAGTTCGAGTAGCTATCAAGAAAATCAGCCCCTTTGAGCACCAGACCTACTGCCAG<br>AGAACCCTGAGGGAGATAAAAATCTTACTGCGCTTCAGACATGAGAACATCATTGGAATCAATGACATTATTCGAGCACCAACCATCGAGCA<br>AATGAAAGATGTATATATAGTACAGGACCTCATGGAAACAGATCTTTACAAGCTCTTGAAGACACAACACCTCAGCAATGACCATATCTGCTAT<br>TTTCTCTACCAGATCCTCAGAGGGTTAAAATATATCCATTCAGCTAACGTTCTGCACCGTGACCTCAAGCCTTCCAACCTGCTGCTCAACAC<br>CACCTGTGATCTCAAGATCTGTGACTTTGGCCTGGCCCGTGTTGCAGATCCAGACCATGATCACACAGGGTTCCTGACAGAATATGTGGCC<br>ACACGTTGGTACAGGGCTCCAGAAATTATGTTGAATTCCAAGGGCTACACCAAGTCCATTGATATTTGGTCTGTAGGCTGCATTCTGGCAGAA<br>ATGCTTTCTAACAGGCCCATCTTTCCAGGGAAGCATTATCTTGACCAGCTGAACCACATTTTGGGTATTCTTGGATCCCCATCACAAGAAGAC<br>CTGAATTGTATAATAAATTTAAAAGCTAGGAACTATTTGCTTTCTCTTCCACACAAAAATAAGGTGCCATGGAACAGGCTGTTCCCAAATGCTGA<br>CTCCAAAGCTCTGGACTTATTGGACAAAATGTTGACATTCAACCCACACAAGAGGATTGAAGTAGAACAGGCTCTGGCCCACCCATATCTG<br>GAGCAGTATTACGACCCGAGTGACGAGCCCATCGCCGAAGCACCATTCAAGTTCGACATGGAATTGGATGACTTGCCTAAGGAAAAGCTC<br>AAAGAACTAATTTTTGAAGAGACTGCTAGATTCCAGCCAGGATACAGATCTTGACAGTAAAGGTGGATACGGATCCGAA
FGFR2A-cf002	SMPAVHKLTKRIPLRRQVSAESSSSMNS<br>NTPLVRITTRLSSTADTPMLAGVSEYELPE<br>DPKWEFPRDKLTLGKPLGEGCFGQVVM<br>AEAVGIDKDKPKEAVTVAVKMLKDDATEK<br>DLSDLVSEMEMMKMIGKHKNIINLLGAC<br>TQDGPLYVIVEYASKGNLREYLRARRPPG<br>MEYSYDINRVPEEQMTFKDLVSCTYQLA<br>RGMEYLASQKCIHRDLAARNVLVTENNV<br>MKIADFGLARDINNIDYYKKTTNGRLPVK<br>WMAPEALFDRVYTHQSDVWSFGVLMW<br>EIFTLGGSPYPGIPVEELFKLLKEGHRMD<br>KPANCTNELYMMMRDCWHAVPSQRPTF<br>KQLVEDLDRILTLTTNEE	CATATGCACCATCATCATCATCATTCTTCTGGTGTAGATCTGGGTACCGAGAACCTGTACTTCCAATCCATGCCGGCTGTGCACAAGCTGAC<br>CAAACGTATCCCCCTGCGGAGACAGGTTTCGGCTGAGTCCAGCTCCTCCATGAACTCCAACACCCCGCTGGTGAGGATAACAACACGC<br>CTCTCTTCAACGGCAGACACCCCCATGCTGGCAGGGGTCTCCGAGTATGAACTTCCAGAGGACCCAAAATGGGAGTTTCCAAGAGATAA<br>GCTGACACTGGGCAAGCCCCTGGGAGAAGGTTGCTTTGGGCAAGTGGTCATGGCGGAAGCAGTGGGAATTGACAAAGACAAGCCCAAG<br>GAGGCGGTCACCGTGGCCGTGAAGATGTTGAAAGATGATGCCACAGAGAAAGACCTTTCTGATCTGGTGTCAGAGATGGAGATGATGAAGA<br>TGATTGGGAAACACAAGAATATCATAAATCTTCTTGGAGCCTGCACACAGGATGGGCCTCTCTATGTCATAGTTGAGTATGCCTCTAAAGGCA<br>ACCTCCGAGAATACCTCCGAGCCCGGAGGCCACCCGGGATGGAGTACTCCTATGACATTAACCGTGTTCCTGAGGAGCAGATGACCTTC<br>AAGGACTTGGTGTCATGCACCTACCAGCTGGCCAGAGGCATGGAGTACTTGGCTTCCCAAAAATGTATTCATCGAGATTTAGCAGCCAGAA<br>ATGTTTTGGTAACAGAAAACAATGTGATGAAAATAGCAGACTTTGGACTCGCCAGAGATATCAACAATATAGACTATTACAAAAAGACCACCAA<br>TGGGCGGCTTCCAGTCAAGTGGATGGCTCCAGAAGCCCTGTTTGATAGAGTATACACTCATCAGAGTGATGTCTGGTCCTTCGGGGTGTTAA<br>TGTGGGAGATCTTCACTTTAGGGGGCTCGCCCTACCCAGGGATTCCCGTGGAGGAACTTTTTAAGCTGCTGAAGGAAGGACACAGAATGG<br>ATAAGCCAGCCAACTGCACCAACGAACTGTACATGATGATGAGGGACTGTTGGCATGCAGTGCCCTCCCAGAGACCAACGTTCAAGCAGT<br>TGGTAGAAGACTTGGATCGAATTCTCACTCTCACAACCAATGAGGAATGACAGTAAAGGTGGATACGGATCCGAA
STK6A-c001	GAMESKKRQWALEDFEIGRPLGKGKFG<br>NVYLAREKQSKFILALKVLFKAQLEKAGV<br>EHQLRREVEIQSHLRHPNILRLYGYFHDA<br>TRVYLILEYAPLGTVYRELQKLSKFDEQRT<br>ATYITELANALSYCHSKRVIHRDIKPENLL<br>LGSAGELKIADFGWSVHAPSSRRTTLCGT<br>LDYLPPEMIEGRMHDEKVDLWSLGVLCY<br>EFLVGKPPFEANTYQETYKRISRVEFTFPD<br>FVTEGARDLISRLLKHNPSQRPMLREVLE<br>HPWITANSSKPSNCQNKESASKQS	N/A
BRPF1B-c001	SMEMQLTPFLILLRKTLEQLQEKDTGNIFS<br>EPVPLSEVPDYLDHIKKPMDFFTMKQNL<br>EAYRYLNFDDFEEDFNLIVSNCLKYNAKD<br>TIFYRAAVRLREQGGAVLRQARRQAEKM<br>G	CATATGCACCATCATCATCATCATTCTTCTGGTGTAGATCTGGGTACCGAGAACCTGTACTTCCAATCCATGGAGATGCAGCTGACTCCTTTC<br>CTCATCCTCCTTCGCAAAACCTTGGAGCAGCTCCAAGAGAAGGACACAGGCAACATCTTCAGCGAGCCGGTCCCTCTGTCTGAGGTACC<br>TGACTACCTAGACCACATCAAAAAGCCCATGGACTTTTTCACCATGAAGCAGAACTTGGAGGCTTACCGCTACCTGAATTTTGATGATTTTGA<br>GGAGGACTTCAACCTCATCGTCAGCAACTGCCTCAAGTATAACGCCAAGGACACCATCTTCTACCGGGCAGCAGTGCGGCTTCGTGAGC<br>AGGGTGGTGCTGTGCTCCGCCAGGCCCGGCGCCAGGCAGAAAAAATGGGCTGACAGTAAAGGTGGATACGGATCCGAA
TIF1A-c026	SMGLVKLTPIDKRKCERLLLFLYCHEMSL<br>AFQDPVPLTVPDYYKIIKNPMDLSTIKKRL<br>QEDYSMYSKPEDFVADFRLIFQNCAEFN<br>EPDSEVANAGIKLENYFEELLKNLYPEKRF<br>PKPE	CATATGCACCATCATCATCATCATCATCATCATCATTCTTCTGGTGTAGATCTGGGTACCGAGAACCTGTACTTCCAATCCATGGGCCTTGTTA<br>AGTTAACACCTATAGATAAAAGGAAGTGTGAGCGCCTACTTTTATTTCTTTACTGCCATGAAATGAGCCTGGCTTTTCAAGACCCTGTTCCTCTA<br>ACTGTGCCTGATTATTACAAAATAATTAAAAATCCAATGGATTTGTCAACCATCAAGAAAAGACTACAAGAAGATTATTCCATGTACTCAAAACCT<br>GAAGATTTTGTAGCTGATTTTAGATTGATCTTTCAAAACTGTGCTGAATTCAATGAGCCTGATTCAGAAGTAGCCAATGCTGGTATAAAACTTGAA<br>AATTATTTTGAAGAACTTCTAAAGAACCTCTATCCAGAAAAAAGGTTTCCCAAACCAGAATGACAGTAAAGGTGGATACGGATCCGAA

GSK3BB-<br>c026	MKVSRDKDGSKVTTVVATPGQGPDRPQ<br>EVSYTDTKVIGNGSFGVVYQAKLCDSGEL<br>VAIKKVLQDKRFKNRELQIMRKLDHCNIV<br>RLRYFFYSSGEKKDEVYLNLVLDYVPETV<br>YRVARHYSRAKQTLPVIYVKLYMYQLFRSL<br>AYIHSFGICHRDIKPQNLLLDPDTAVLKLC<br>DFGSAKQLVRGEPNVSYICSRYYRAPELI<br>FGATDYTSSIDVWSAGCVLAELLLGQPIF<br>PGDSGVDQLVEIIKVLGTPTREQIREMNP<br>NYTEFKFPQIKAHPWTKVFRPRTPPEAIAL<br>CSRLLEYTPTARLTPLEACAHSFFDELRD<br>PNVKLPNGRDTPALFNFTTQELSSNPPLA<br>TILIPPHARAHHHHHH	CTTAAGAAGGAGATATACTATGAAAGTTAGCAGAGACAAGGACGGCAGCAAGGTGACAACAGTGGTGGCAACTCCTGGGCAGGGTCCAG<br>ACAGGCCACAAGAAGTCAGCTATACAGACACTAAAGTGATTGGAAATGGATCATTTGGTGTGGTATATCAAGCCAAACTTTGTGATTCAGGAG<br>AACTGGTCGCCATCAAGAAAGTATTGCAGGACAAGAGATTTAAGAATCGAGAGCTCCAGATCATGAGAAAGCTAGATCACTGTAACATAGTC<br>CGATTGCGTTATTTCTTCTACTCCAGTGGTGAGAAGAAAGATGAGGTCTATCTTAATCTGGTGCTGGACTATGTTCCGGAAACAGTATACAGAG<br>TTGCCAGACACTATAGTCGAGCCAAACAGACGCTCCCTGTGATTTATGTCAAGTTGTATATGTATCAGCTGTTCCGAAGTTTAGCCTATATCCA<br>TTCCTTTGGAATCTGCCATCGGGATATTAAACCGCAGAACCTCTTGTTGGATCCTGATACTGCTGTATTAAAACTCTGTGACTTTGGAAGTGCA<br>AAGCAGCTGGTCCGAGGAGAACCCAATGTTTCGTATATCTGTTCTCGGTACTATAGGGCACCAGAGTTGATCTTTGGAGCCACTGATTATACC<br>TCTAGTATAGATGTATGGTCTGCTGGCTGTGTGTTGGCTGAGCTGTTACTAGGACAACCAATATTTCCAGGGGATAGTGGTGTGGATCAGTTGG<br>TAGAAATAATCAAGGTCCTGGGAACTCCAACAAGGGAGCAAATCAGAGAAATGAACCCAAACTACACAGAATTTAAATTCCCTCAAATTAAG<br>GCACATCCTTGGACTAAGGTCTTCCGACCCCGAACTCCACCGGAGGCAATTGCACTGTGTAGCCGTCTGCTGGAGTATACACCAACTGC<br>CCGACTAACACCACTGGAAGCTTGTGCACATTCATTTTTGATGAATTACGGGACCCAAATGTCAAACTACCAAATGGGCGAGACACACCTG<br>CACTCTTCAACTTCACCACTCAAGAACTGTCAAGTAATCCACCTCTGGCTACCATCCTTATTCCTCCTCATGCTCGGGCGCACCATCATCAC<br>CACCATTGAGGATCC
CDK2A-<br>c002	SMENFQKVEKIGEGTYGVVYKARNKLTG<br>EVVALKKIRLDTETEGVPSTAIREISLLKEL<br>NHPNIVKLLDVIHTENKLYLVFEFLHQDL<br>KKFMDASALTGIPLPLIKSYLFQLLQGLAF<br>CHSHRVLHRDLKPQNLLINTEGAIKLADF<br>GLARAFGVPVRTYTHEVVTLWYRAPEILL<br>GCKYYSTAVDIWSLGCIFAEMVTRRALFP<br>GDSEIDQLFRIFRTLGTPDEVVWPGVTSM<br>PDYKPSFPKWARQDFSKVVPPLDEDGR<br>SLLSQMLHYDPNKRISAKAALAHPFFQD<br>VTKPVPHLRL	CATATGCACCATCATCATCATCATTCTTCTGGTGTAGATCTGGGTACCGAGAACCTGTACTTCCAATCCATGGAGAACTTCCAAAAGGTGGAA<br>AAGATCGGAGAGGGCACGTACGGAGTTGTGTACAAAGCCAGAAACAAGTTGACGGGAGAGGTGGTGGCGCTTAAGAAAATCCGCCTGGA<br>CACTGAGACTGAGGGTGTGCCCAGTACTGCCATCCGAGAGATCTCTCTGCTTAAGGAGCTTAACCATCCTAATATTGTCAAGCTGCTGGATG<br>TCATTCACACAGAAAATAAACTCTACCTGGTTTTTGAATTTCTGCACCAAGATCTCAAGAAATTCATGGATGCCTCTGCTCTCACTGGCATTCC<br>TCTTCCCCTCATCAAGAGCTATCTGTTCCAGCTGCTCCAGGGCCTAGCTTTCTGCCATTCTCATCGGGTCCTCCACCGAGACCTTAAACCT<br>CAGAATCTGCTTATTAACACAGAGGGGGCCATCAAGCTAGCAGACTTTGGACTAGCCAGAGCTTTTGGAGTCCCTGTTCGTACTTACACCC<br>ATGAGGTGGTGACCCTGTGGTACCGAGCTCCTGAAATCCTCCTGGGCTGCAAATATTATTCCACAGCTGTGGACATCTGGAGCCTGGGCTG<br>CATCTTTGCTGAGATGGTGACTCGCCGGGCCCTATTCCCTGGAGATTCTGAGATTGACCAGCTCTTCCGGATCTTTCGGACTCTGGGGACC<br>CCAGATGAGGTGGTGTGGCCAGGAGTTACTTCTATGCCTGATTACAAGCCAAGTTTCCCCAAGTGGGCCCGGCAAGATTTTAGTAAAGTTGT<br>ACCTCCCCTGGATGAAGATGGACGGAGCTTGTTATCGCAAATGCTGCACTACGACCCTAACAAGCGGATTTCGGCCAAGGCAGCCCTGG<br>CTCACCCTTTCTTCCAGGATGTGACCAAGCCAGTACCCCATCTTCGACTCTGACAGTAAAGGTGGATACGGATCCGAA
BRD4A-<br>c002	SMNPPPPETSNPNKPKRQTNQLQYLLRV<br>VLKTLWKHQFAWPFQQPVDAVKLNLPD<br>YYKIIKTPMDMGTIKKRLENNYYWNAQEC<br>IQDFNTMFTNCYIYNKPGDDIVLMAEALE<br>KLFLQKINELPTEE	CATATGCACCATCATCATCATCATTCTTCTGGTGTAGATCTGGGTACCGAGAACCTGTACTTCCAATCCATGAACCCCCCGCCCCCAGAGA<br>CCTCCAACCCTAACAAGCCCAAGAGGCAGACCAACCAACTGCAATACCTGCTCAGAGTGGTGCTCAAGACACTATGGAAACACCAGTTT<br>GCATGGCCTTTCCAGCAGCCTGTGGATGCCGTCAAGCTGAACCTCCCTGATTACTATAAGATCATTAAAACGCCTATGGATATGGGAACAAT<br>AAAGAAGCGCTTGGAAAACAACTATTACTGGAATGCTCAGGAATGTATCCAGGACTTCAACACTATGTTTACAAATTGTTACATCTACAACAAG<br>CCTGGAGATGACATAGTCTTAATGGCAGAAGCTCTGGAAAAGCTCTTCTTGCAAAAAATAAATGAGCTACCCACAGAAGAATGACAGTAAAG<br>GTGGATACGGATCCGAA

Supplementary References

1. Ocasio, C. A. & Scanlan, T. S. Characterization of thyroid hormone receptor α (TRα)-specific analogs with varying inner- and outer-ring substituents. *Bioorg Med Chem* **16**, 762–770 (2008).

2. Nguyen, N. H. *et al.* Rational design and synthesis of a novel thyroid hormone antagonist that blocks coactivator recruitment. *J Med Chem* **45**, 3310–3320 (2002).

3. Hedfors, Å. *et al.* Thyroid receptor ligands. 3. Design and synthesis of 3,5-dihalo-4-alkoxyphenylalkanoic acids as indirect antagonists of the thyroid hormone receptor. *J Med Chem* **48**, 3114–3117 (2005).

4. Carlsson, B. *et al.* Synthesis and Preliminary Characterization of a Novel Antiarrhythmic Compound (KB130015) with an Improved Toxicity Profile Compared with Amiodarone. *J Med Chem* **45**, 623–630 (2002).

5. Oka, T. *et al.* Establishment of transactivation assay systems using fish, amphibian, reptilian and human thyroid hormone receptors. *Journal of Applied Toxicology* **33**, 991–1000 (2013).

6. Kelly, M. J. *et al.* Discovery of 2-[3,5-dichloro-4-(5-isopropyl-6-oxo-1,6-dihydropyridazin-3- yloxy)phenyl]-3,5-dioxo-2,3,4,5-tetrahydro[1,2,4]triazine-6-carbonitrile (MGL-3196), a highly selective thyroid hormone receptor β agonist in clinical trials for the treatment of dyslipidemia. *J Med Chem* **57**, 3912–3923 (2014).

7. Géhin, M. *et al.* Structural basis for engineering of retinoic acid receptor isotype-selective agonists and antagonists. *Chem Biol* **6**, 519–529 (1999).

8. Vizirianakis, I. S. *et al.* Toward the development of innovative bifunctional agents to induce differentiation and to promote apoptosis in leukemia: Clinical candidates and perspectives. *Journal of Medicinal Chemistry* vol. 53 6779–6810 Preprint at https://doi.org/10.1021/jm100189a (2010).

9. Charton, J. *et al.* Novel non-carboxylic acid retinoids: 1,2,4-Oxadiazol-5-one derivatives. *Bioorg Med Chem Lett* **19**, 489–492 (2009).

10. Idres, N., Marill, J., Flexor, M. A. & Chabot, G. G. Activation of retinoic acid receptor-dependent transcription by all-trans-retinoic acid metabolites and isomers. *Journal of Biological Chemistry* **277**, 31491–31498 (2002).

11. Gege, C., Schlüter, T. & Hoffmann, T. Identification of the first inverse agonist of retinoid-related orphan receptor (ROR) with dual selectivity for RORβ and RORγt. *Bioorg Med Chem Lett* **24**, 5265–5267 (2014).

12. Thoreau, E. *et al.* Structure-based design of Trifarotene (CD5789), a potent and selective RARγ agonist for the treatment of acne. *Bioorg Med Chem Lett* **28**, 1736–1741 (2018).

13. Lund, B. W. *et al.* Discovery of a potent, orally available, and isoform-selective retinoic acid β2 receptor agonist. *J Med Chem* **48**, 7517–7519 (2005).

14. Lund, B. W. *et al.* Design, synthesis, and structure-activity analysis of isoform-selective retinoic acid receptor β ligands. *J Med Chem* **52**, 1540–1545 (2009).

15. Brown, P. J. *et al. Identification of a Subtype Selective Human PPAR Agonist Through Parallel-Array Synthesis*.

16. Kane, C. D. *et al.* Molecular characterization of novel and selective peroxisome proliferator-activated receptor α agonists with robust hypolipidemic activity in vivo. *Mol Pharmacol* **75**, 296–306 (2009).

17. Martres, P. *et al.* The discovery of equipotent PPARα/γ dual activators. *Bioorg Med Chem Lett* **18**, 6251–6254 (2008).

18. Sierra, M. L. *et al.* Substituted 2-[(4-aminomethyl)phenoxy]-2-methylpropionic acid PPARα agonists. 1. Discovery of a novel series of potent HDLc raising agents. *J Med Chem* **50**, 685–695 (2007).

19. Devasthale, P. V. *et al.* Design and synthesis of N-[(4-methoxyphenoxy)carbonyl]-N-[[4-[2-(5-methyl- 2-phenyl-4-oxazolyl)ethoxy]phenyl]methyl]glycine [muraglitazar/BMS-298585], a novel peroxisome proliferator-activated receptor α/γ dual agonist with efficacious glucose and lipid-lowering activities. *J Med Chem* **48**, 2248–2250 (2005).

20. Ebdrup, S. *et al.* Synthesis and biological and structural characterization of the dual-acting peroxisome proliferator-activated receptor α/γ agonist ragaglitazar. *J Med Chem* **46**, 1306–1317 (2003).

21. Brown, P. J. *et al.* A ureido-thioisobutyric acid (GW9578) is a subtype-selective PPARα agonist with potent lipid-lowering activity [2]. *Journal of Medicinal Chemistry* vol. 42 3785–3788 Preprint at https://doi.org/10.1021/jm9903601 (1999).

22. Fu, J. *et al. Oleylethanolamide Regulates Feeding and Body Weight through Activation of the Nuclear Receptor PPAR-a*. www.nature.com/nature (2003).

23. Jiang, Z. *et al.* Discovery of a Novel Selective Dual Peroxisome Proliferator-Activated Receptor α/δAgonist for the Treatment of Primary Biliary Cirrhosis. *ACS Med Chem Lett* **10**, 1068–1073 (2019).

24. Sznaidman, M. L. *et al.* Novel selective small molecule agonists for peroxisome proliferator-activated receptor δ (PPARδ) - Synthesis and biological activity. *Bioorg Med Chem Lett* **13**, 1517–1521 (2003).

25. Boubia, B. *et al.* Design, Synthesis, and Evaluation of a Novel Series of Indole Sulfonamide Peroxisome Proliferator Activated Receptor (PPAR) α/γ/δ Triple Activators: Discovery of Lanifibranor, a New Antifibrotic Clinical Candidate. *J Med Chem* **61**, 2246–2265 (2018).

26. Ekambomé Basséne, C. *et al.* Studies towards the conception of new selective PPARβ/δ ligands. *Bioorg Med Chem Lett* **16**, 4528–4532 (2006).

27. Lieber, S. *et al.* (Z)-2-(2-bromophenyl)-3-{[4-(1-methyl-piperazine)amino]phenyl}acrylonitrile (DG172): An orally bioavailable PPARβ/δ-selective ligand with inverse agonistic properties. *J Med Chem* **55**, 2858–2868 (2012).

28. Zhang, R. *et al.* Discovery of para-alkylthiophenoxyacetic acids as a novel series of potent and selective PPARδ agonists. *Bioorg Med Chem Lett* **17**, 3855–3859 (2007).

29. Oliver, W. R. *et al. A Selective Peroxisome Proliferator-Activated Receptor Agonist Promotes Reverse Cholesterol Transport*. www.pnas.orgcgidoi10.1073pnas.091021198.

30. Shearer, B. G. *et al.* Identification and characterization of a selective peroxisome proliferator-activated receptor β/δ (NR1C2) antagonist. *Molecular Endocrinology* **22**, 523–529 (2008).

31. Shearer, B. G. *et al.* Identification and characterization of 4-chloro-N-(2- {[5-trifluoromethyl)- 2-pyridyl] sulfonyl} ethyl)benzamide (GSK3787), a selective and irreversible peroxisome proliferator-activated receptor δ (PPARδ) antagonist. *J Med Chem* **53**, 1857–1861 (2010).

32. Lamotte, Y. *et al.* Synthesis and biological activities of novel indole derivatives as potent and selective PPARγ modulators. *Bioorg Med Chem Lett* **20**, 1399–1404 (2010).

33. Carmona, M. C. *et al.* S 26948: A new specific peroxisome proliferator-activated receptor γ modulator with potent antidiabetes and antiatherogenic effects. *Diabetes* **56**, 2797–2808 (2007).

34. Zhou, X. *et al.* (S)-3-(4-(2-(5-Methyl-2-phenyloxazol-4-yl)ethoxy)phenyl)-2-(piperazin-1-yl)propanoic acid compounds: Synthesis and biological evaluation of dual PPARα/γ agonists. *Bioorg Med Chem Lett* **20**, 2605–2608 (2010).

35. Feng, L. *et al.* Bavachinin, as a novel natural pan-PPAR agonist, exhibits unique synergistic effects with synthetic PPAR-γ and PPAR-α agonists on carbohydrate and lipid metabolism in db/db and diet-induced obese mice. *Diabetologia* **59**, 1276–1286 (2016).

36. Michellys, P. Y. *et al.* Novel (2E,4E,6Z)-7-(2-alkoxy-3,5-dialkylbenzene)-3-methylocta-2,4,6-trienoic acid retinoid X receptor modulators are active in models of type 2 diabetes. *J Med Chem* **46**, 2683–2696 (2003).

37. Kojetin, D., Wang, Y., Kamenecka, T. M. & Burris, T. P. Identification of SR8278, a synthetic antagonist of the nuclear heme receptor REV-ERB. *ACS Chem Biol* **6**, 131–134 (2011).

38. Shin, Y. *et al.* Small molecule tertiary amines as agonists of the nuclear hormone receptor Rev-erbα. *Bioorg Med Chem Lett* **22**, 4413–4417 (2012).

39. Solt, L. A. *et al.* Regulation of circadian behaviour and metabolism by synthetic REV-ERB agonists. *Nature* **485**, 62–68 (2012).

40. Kumar, N. *et al.* Identification of SR3335 (ML-176): A synthetic RORα selective inverse agonist. *ACS Chem Biol* **6**, 218–222 (2011).

41. Fauber, B. P. *et al.* Structure-based design of substituted hexafluoroisopropanol-arylsulfonamides as modulators of RORc. *Bioorg Med Chem Lett* **23**, 6604–6609 (2013).

42. Wang, Y. *et al.* Identification of SR1078, a synthetic agonist for the orphan nuclear receptors RORα and RORγ. *ACS Chem Biol* **5**, 1029–1034 (2010).

43. Ouvry, G. *et al.* Sulfoximines as potent RORγ inverse agonists. *Bioorg Med Chem Lett* **28**, 1269–1273 (2018).

44. Schnute, M. E. *et al.* Discovery of 3-Cyano- N-(3-(1-isobutyrylpiperidin-4-yl)-1-methyl-4-(trifluoromethyl)-1 H-pyrrolo[2,3- b]pyridin-5-yl)benzamide: A Potent, Selective, and Orally Bioavailable Retinoic Acid Receptor-Related Orphan Receptor C2 Inverse Agonist. *J Med Chem* **61**, 10415–10439 (2018).

45. Aicher, T. D., Taylor, C. B. & VanHuis, C. A. Aryl dihydro-2H-benzo[b][1,4]oxazine sulfonamide and related compounds for use as agonists of RORγ and the treatment of disease. (2016).

46. Kumar, N. *et al.* Identification of SR2211: A potent synthetic RORγ-selective modulator. *ACS Chemical Biology* vol. 7 672–677 Preprint at https://doi.org/10.1021/cb200496y (2012).

47. Wang, J. *et al.* ROR-γ drives androgen receptor expression and represents a therapeutic target in castration-resistant prostate cancer. *Nat Med* **22**, 488–496 (2016).

48. Huh, J. R. *et al.* Identification of potent and selective diphenylpropanamide RORγ inhibitors. *ACS Med Chem Lett* **4**, 79–84 (2013).

49. Fauber, B. P. & Magnuson, S. Modulators of the nuclear receptor retinoic acid receptor-related orphan receptor-γ (RORγ or RORc). *Journal of Medicinal Chemistry* vol. 57 5871–5892 Preprint at https://doi.org/10.1021/jm401901d (2014).

50. Collins, J. L. *et al.* Identification of a nonsteroidal liver X receptor agonist through parallel array synthesis of tertiary amines. *J Med Chem* **45**, 1963–1966 (2002).

51. Zuercher, W. J. *et al.* Discovery of tertiary sulfonamides as potent liver X receptor antagonists. *J Med Chem* **53**, 3412–3416 (2010).

52. Griffett, K., Solt, L. A., El-Gendy, B. E. D. M., Kamenecka, T. M. & Burris, T. P. A liver-selective LXR inverse agonist that suppresses hepatic steatosis. *ACS Chem Biol* **8**, 559–567 (2013).

53. El-Gendy, B. E. D. M., Goher, S. S., Hegazy, L. S., Arief, M. M. H. & Burris, T. P. Recent Advances in the Medicinal Chemistry of Liver X Receptors. *Journal of Medicinal Chemistry* vol. 61 10935–10956 Preprint at https://doi.org/10.1021/acs.jmedchem.8b00045 (2018).

54. Wrobel, J. *et al.* Indazole-based Liver X Receptor (LXR) modulators with maintained atherosclerotic lesion reduction activity but diminished stimulation of hepatic triglyceride synthesis. *J Med Chem* **51**, 7161–7168 (2008).

55. Fu, X. *et al.* 27-Hydroxycholesterol Is an Endogenous Ligand for Liver X Receptor in Cholesterol-loaded Cells. *Journal of Biological Chemistry* **276**, 38378–38387 (2001).

56. Marino, J. P. *et al.* The discovery of tertiary-amine LXR agonists with potent cholesterol efflux activity in macrophages. *Bioorg Med Chem Lett* **19**, 5617–5621 (2009).

57. Kick, E. K. *et al.* Discovery of Highly Potent Liver X Receptor β Agonists. *ACS Med Chem Lett* **7**, 1207–1212 (2016).

58. Kick, E. *et al.* Liver X Receptor (LXR) partial agonists: Biaryl pyrazoles and imidazoles displaying a preference for LXRβ. *Bioorg Med Chem Lett* **25**, 372–377 (2015).

59. Sparrow, C. P. *et al.* A potent synthetic LXR agonist is more effective than cholesterol loading at inducing ABCA1 mRNA and stimulating cholesterol efflux. *Journal of Biological Chemistry* **277**, 10021–10027 (2002).

60. Kainuma, M., Makishima, M., Hashimoto, Y. & Miyachi, H. Design, synthesis, and evaluation of non-steroidal farnesoid X receptor (FXR) antagonist. *Bioorg Med Chem* **15**, 2587–2600 (2007).

61. Akwabi-Ameyaw, A. *et al.* Conformationally constrained farnesoid X receptor (FXR) agonists: Naphthoic acid-based analogs of GW 4064. *Bioorg Med Chem Lett* **18**, 4339–4343 (2008).

62. Lundquist IV, J. T. *et al.* Improvement of physiochemical properties of the tetrahydroazepinoindole series of farnesoid X receptor (FXR) agonists: Beneficial modulation of lipids in primates. *J Med Chem* **53**, 1774–1787 (2010).

63. Mehlmann, J. F. *et al.* Pyrrole[2,3-d]azepino compounds as agonists of the farnesoid X receptor (FXR). *Bioorg Med Chem Lett* **19**, 5289–5292 (2009).

64. Downes, M. *et al.* A Chemical, Genetic, and Structural Analysis of the Nuclear Bile Acid Receptor FXR. *Mol Cell* **11**, 1079–1092 (2003).

65. Nicolaou, K. C. Joys of molecules. 2. Endeavors in chemical biology and medicinal chemistry. *Journal of Medicinal Chemistry* vol. 48 5613–5638 Preprint at https://doi.org/10.1021/jm050524f (2005).

66. Merk, D., Steinhilber, D. & Schubert-Zsilavecz, M. Characterizing ligands for farnesoid X receptor-available in vitro test systems for farnesoid X receptor modulator development. *Expert Opinion on Drug Discovery* vol. 9 27–37 Preprint at https://doi.org/10.1517/17460441.2014.860129 (2014).

67. Yu, D. D., Lin, W., Forman, B. M. & Chen, T. Identification of trisubstituted-pyrazol carboxamide analogs as novel and potent antagonists of farnesoid X receptor. *Bioorg Med Chem* **22**, 2919–2938 (2014).

68. Genin, M. J. *et al.* Discovery of 6-(4-{[5-Cyclopropyl-3-(2,6-dichlorophenyl)isoxazol-4-yl]methoxy}piperidin-1-yl)-1-methyl-1H-indole-3-carboxylic Acid: A Novel FXR Agonist for the Treatment of Dyslipidemia. *J Med Chem* **58**, 9768–9772 (2015).

69. Romero, F. A., Jones, C. T., Xu, Y., Fenaux, M. & Halcomb, R. L. The Race to Bash NASH: Emerging Targets and Drug Development in a Complex Liver Disease. *Journal of Medicinal Chemistry* vol. 63 5031–5073 Preprint at https://doi.org/10.1021/acs.jmedchem.9b01701 (2020).

70. Tully, D. C. *et al.* Discovery of Tropifexor (LJN452), a Highly Potent Non-bile Acid FXR Agonist for the Treatment of Cholestatic Liver Diseases and Nonalcoholic Steatohepatitis (NASH). *J Med Chem* **60**, 9960–9973 (2017).

71. Lin, Z. *et al.* Synthesis and Biological Evaluation of Vitamin D3 Metabolite 20S,23S-Dihydroxyvitamin D3 and Its 23R Epimer. *J Med Chem* **59**, 5102–5108 (2016).

72. Tocchini-Valentini, G., Rochel, N., Wurtz, J. M. & Moras, D. Crystal Structures of the Vitamin D Nuclear Receptor Liganded with the Vitamin D Side Chain Analogues Calcipotriol and Seocalcitol, Receptor Agonists of Clinical Importance. Insights into a Structural Basis for the Switching of Calcipotriol to a Receptor Antagonist by Further Side Chain Modification. *J Med Chem* **47**, 1956–1961 (2004).

73. Wang, X. X. *et al.* Vitamin D receptor agonist doxercalciferol modulates dietary fat-induced renal disease and renal lipid metabolism. *Am J Physiol Renal Physiol* **300**, 801–810 (2011).

74. Toyama, H. *et al.* Altered activity profile of a tertiary silanol analog of multi-targeting nuclear receptor modulator T0901317. *Bioorg Med Chem Lett* **26**, 1817–1820 (2016).

75. Shukla, S. J. *et al.* Identification of clinically used drugs that activate pregnane X receptors. *Drug Metabolism and Disposition* **39**, 151–159 (2011).

76. Morley, A. *et al.* Effect of lipophilicity modulation on inhibition of human rhinovirus capsid binders. *Bioorg Med Chem Lett* **21**, 6031–6035 (2011).

77. Lehmann, J. M. *et al.* The human orphan nuclear receptor PXR is activated by compounds that regulate CYP3A4 gene expression and cause drug interactions. *Journal of Clinical Investigation* **102**, 1016–1023 (1998).

78. Lemaire, G. *et al.* Discovery of a highly active ligand of human pregnane X receptor: A case study from pharmacophore modeling and virtual screening to 'in vivo' biological activity. *Mol Pharmacol* **72**, 572–581 (2007).

79. Lin, W., Yang, L., Chai, S. C., Lu, Y. & Chen, T. Development of CINPA1 analogs as novel and potent inverse agonists of constitutive androstane receptor. *Eur J Med Chem* **108**, 505–528 (2016).

80. Huang, W., Zhang, J., Wei, P., Schrader, W. T. & Moore, D. D. Meclizine is an agonist ligand for mouse constitutive androstane receptor (CAR) and an inverse agonist for human CAR. *Molecular Endocrinology* **18**, 2402–2408 (2004).

81. Maglich, J. M. *et al.* Identification of a novel human constitutive androstane receptor (CAR) agonist and its use in the identification of CAR target genes. *Journal of Biological Chemistry* **278**, 17277–17283 (2003).

82. Moore, L. B. *et al.* Orphan nuclear receptors constitutive androstane receptor and pregnane X receptor share xenobiotic and steroid ligands. *Journal of Biological Chemistry* **275**, 15122–15127 (2000).

83. Isigkeit, L., Chaikuad, A. & Merk, D. A Consensus Compound/Bioactivity Dataset for Data-Driven Drug Design and Chemogenomics. *Molecules* **27**, (2022).

84. Rak, M. *et al.* Development of Selective Pyrido[2,3-d]pyrimidin-7(8H)-one-Based Mammalian STE20-Like (MST3/4) Kinase Inhibitors. *J Med Chem* **67**, 3813–3842 (2024).
