# Supplementary material for: Chemogenomics for NR1 nuclear hormone receptors
Source: Nat Commun. 2024 Jun 18;15:5201. doi: 10.1038/s41467-024-49493-6 (PMC11189487; doi:10.1038/s41467-024-49493-6)
Supplement: Supplementary file 3 — Description of Additional Supplementary Files [file 41467_2024_49493_MOESM3_ESM.docx]

**Description of Additional Supplementary Files**

**Supplementary Data 1:**
The CG compound sheets contains bioactivity annotation and analytical data of the NR1 CG compounds in human-readable format (individual pdf files and one xlsx file with assay data).
