# Supplementary material for: Chemogenomics for NR1 nuclear hormone receptors
Source: Nat Commun. 2024 Jun 18;15:5201. doi: 10.1038/s41467-024-49493-6 (PMC11189487; doi:10.1038/s41467-024-49493-6)

COMPOUND INFORMATION

6-ECDCA (Obeticholic acid)

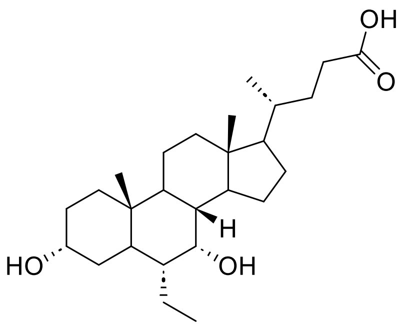

**CAS Registry No.:**	459789-99-2
**Formal Name:**	(R)-4-((3R,5S,6R,7R,8S,9S,10S,13R,14S,17R)-6-ethyl-3,7-dihydroxy-10,13-dimethylhexadecahydro-1H-cyclopenta[a]phenanthren-17-yl)pentanoic acid
**EUBOPEN ID:**	EUB0000081c
**Molecular Formula:**	$C_{26}H_{44}O_4$
**Molecular Weight:**	420.63 g/mol
**Smiles:**	O[C@@H]1CC[C@]2([C@@]([H])(C1)[C@H]([C@H]([C@@]3([C@@]2(CC[C@]4([C@]3(CC[C@@]4([C@H](C)CCC(O)=O)[H])[H])C)[H])[H])O)CC)C
**Recommended concentration:**	1 µM

Biological activity

		Type	IC_{50}/EC_{50} [µM]	Reference
Main NR target:	NR1H4 (FXR)	Agonist	0.30	inhouse
NR off-target:				

COMPOUND INFORMATION

Identity

^{1}H NMR

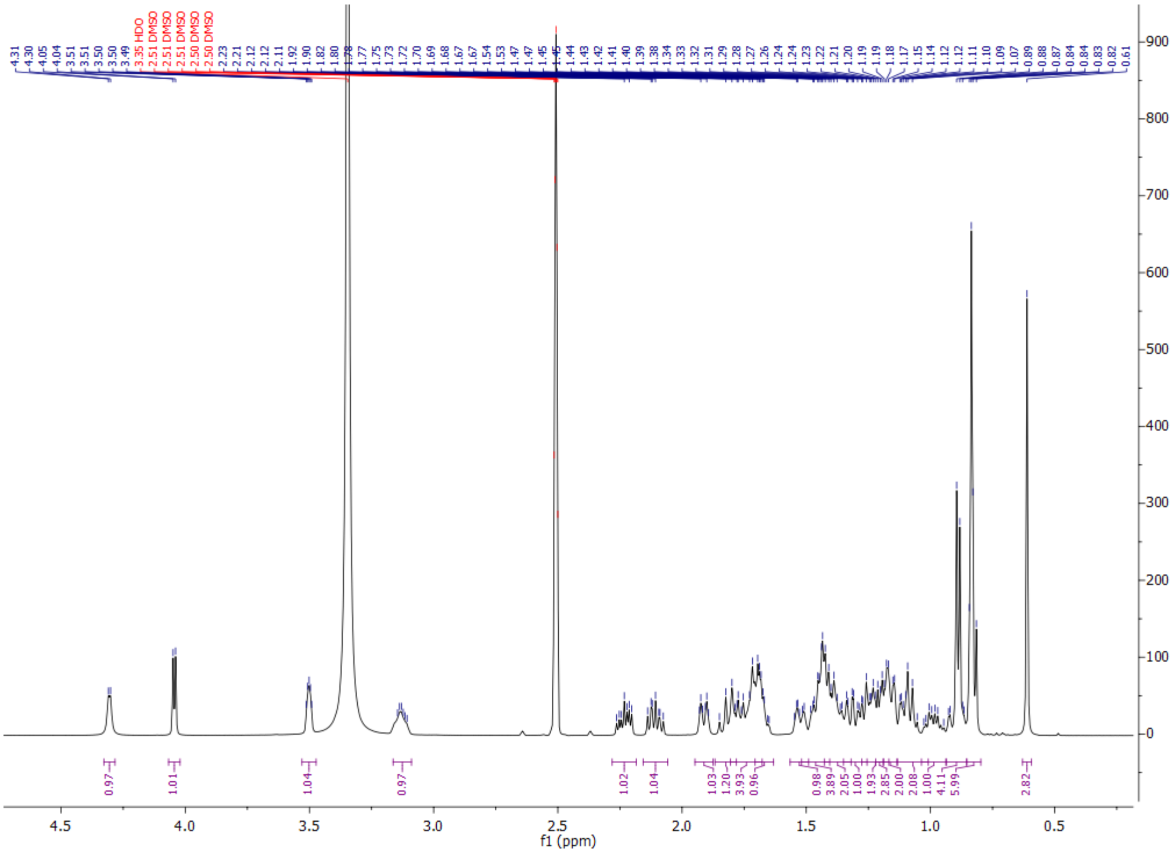

^{13}C NMR

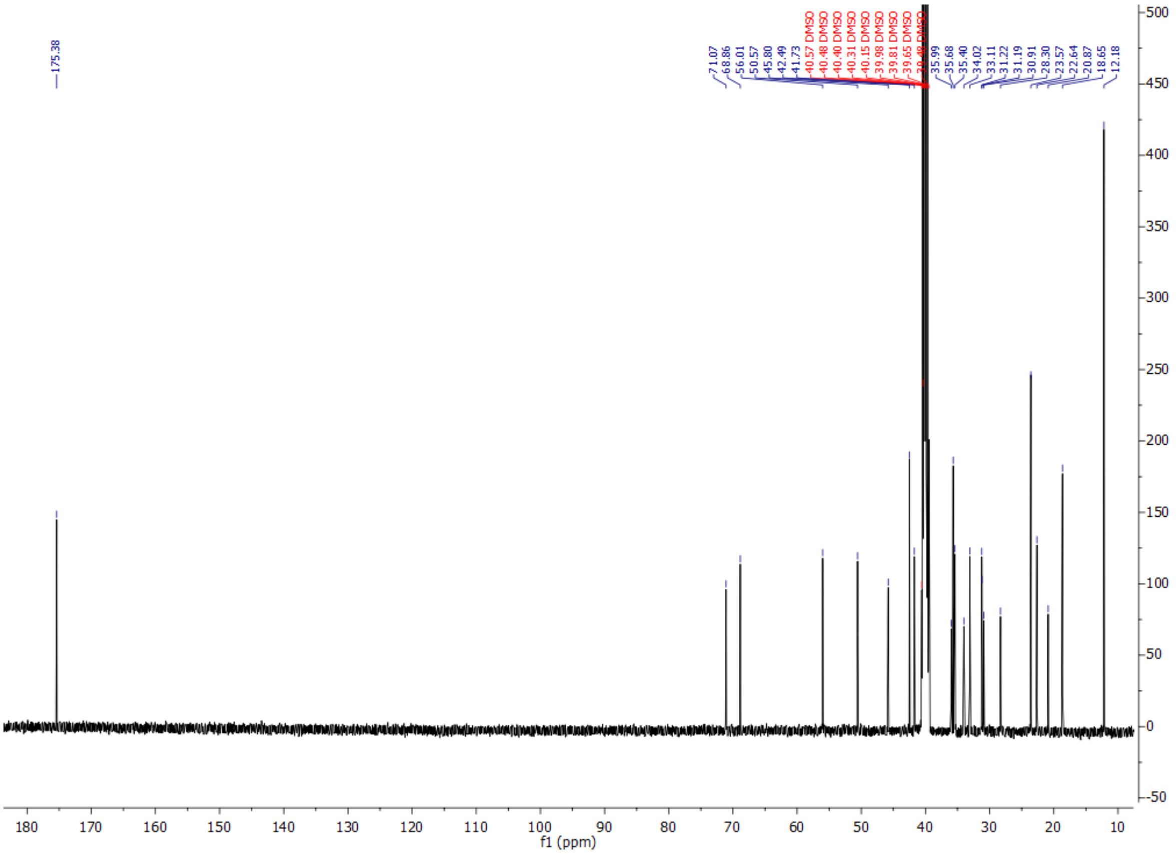

COMPOUND INFORMATION

Purity

LC-MS

*M*r 420.63

MS: ESI-negative, m/z 419/401

LC: 0.1% HCOOH/ACN (30/70)

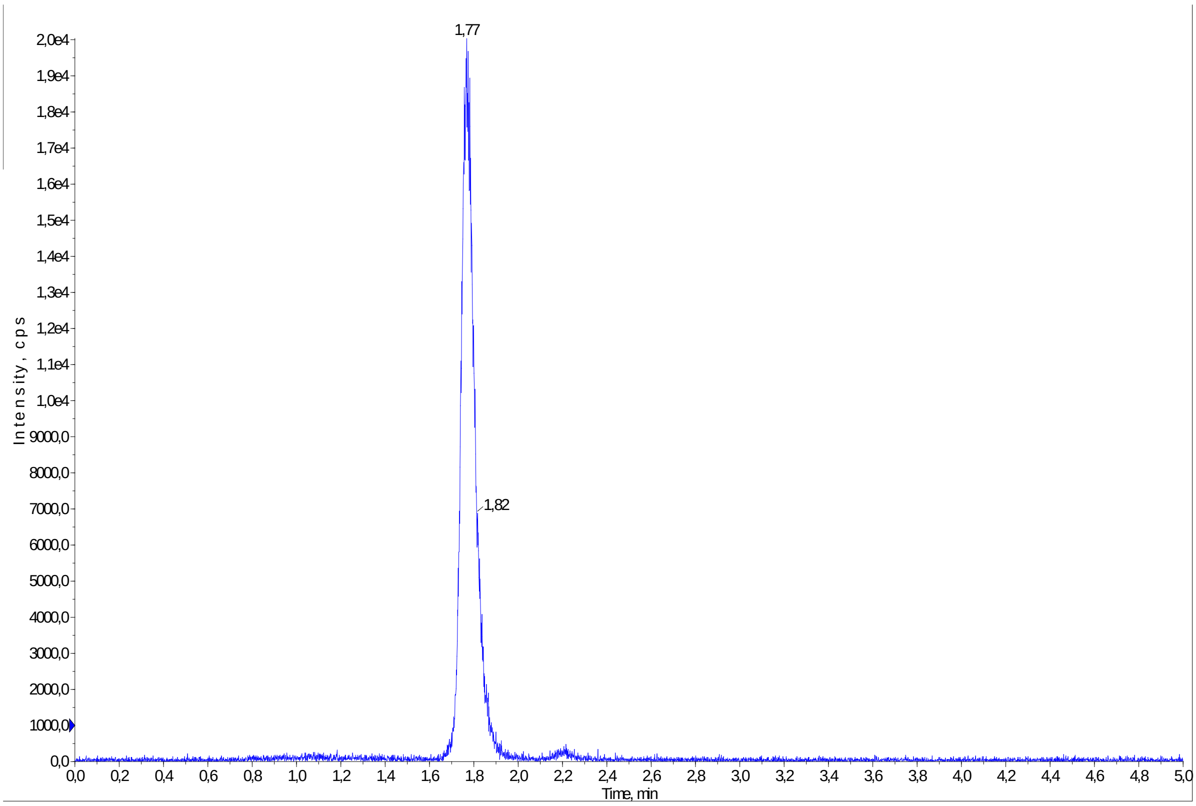

LC-ELSD

LC: 0.1% HCOOH/ACN (30/70), sample concentration 100 µM

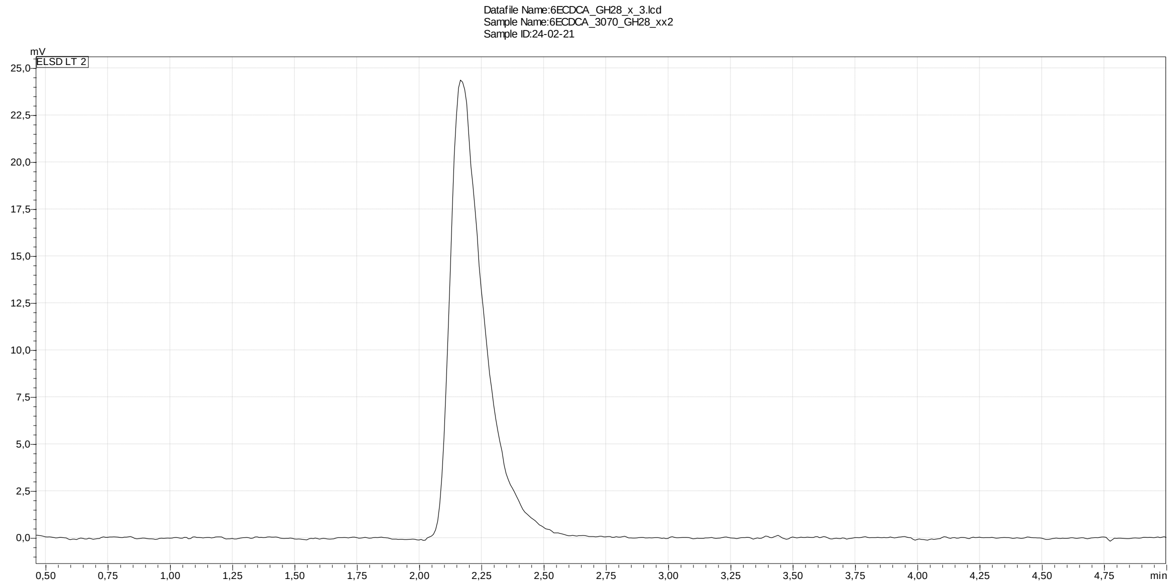

COMPOUND INFORMATION

Biological activity

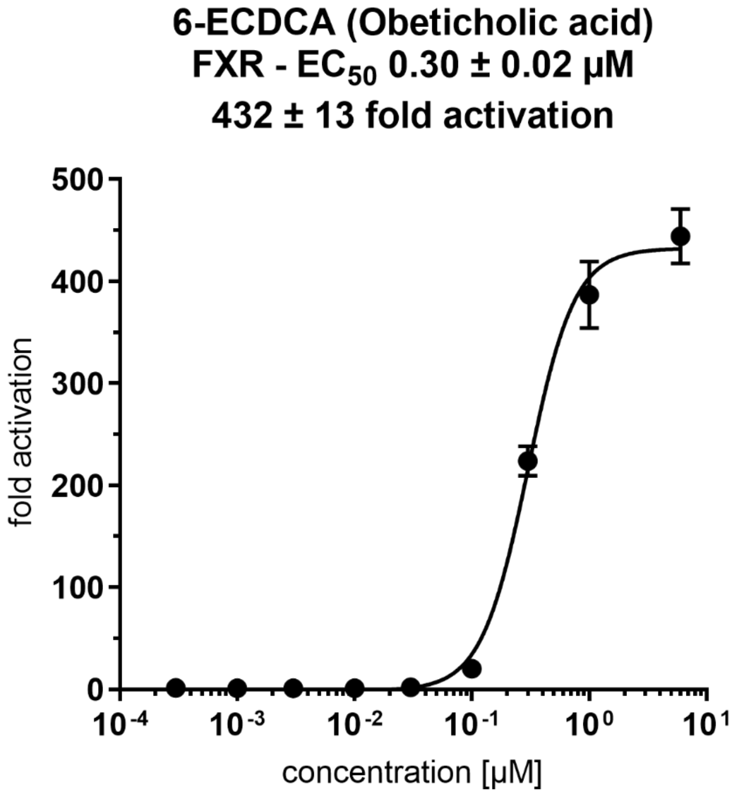

Supplement: Supplementary file 4 — Supplementary Data 1 [file 41467_2024_49493_MOESM4_ESM.zip › 6-ECDCA.pdf]
