# Supplementary material for: Chemogenomics for NR1 nuclear hormone receptors
Source: Nat Commun. 2024 Jun 18;15:5201. doi: 10.1038/s41467-024-49493-6 (PMC11189487; doi:10.1038/s41467-024-49493-6)

COMPOUND INFORMATION

AC-261066

**CAS Registry No.:**	870773-76-5
**Formal Name:**	4-(4-(2-butoxyethoxy)-5-methylthiazol-2-yl)-2-fluorobenzoic acid
**EUbOPEN ID:**	EUB0000835a
**Molecular Formula:**	$C_{17}H_{20}FNO_4S$
**Molecular Weight:**	353.41 g/mol
**Smiles:**	CCCCOCCOC1=C(SC(=N1)C2=CC(=C(C=C2)C(=O)O)F)C
**Purity:**	100 %
**Recommended concentration:**	1 µM

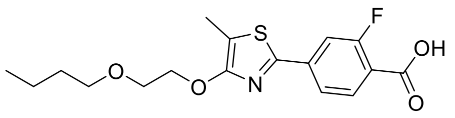

Biological activity

		Type	IC_{50}/EC_{50} [µM]	Reference
Main NR target:	NR1B1 (RARα)	Agonist	0.6	https://doi.org/10.1021/jm801532e, https://doi.org/10.1021/jm050891r
	NR1B2 (RARβ)	Agonist	0.010	
	NR1B3 (RARγ)	Agonist	0.5	
NR off-target:	NR2B1 (RXRα)	Agonist	26-fold act.	inhouse

COMPOUND INFORMATION

Identity

^{1}H NMR

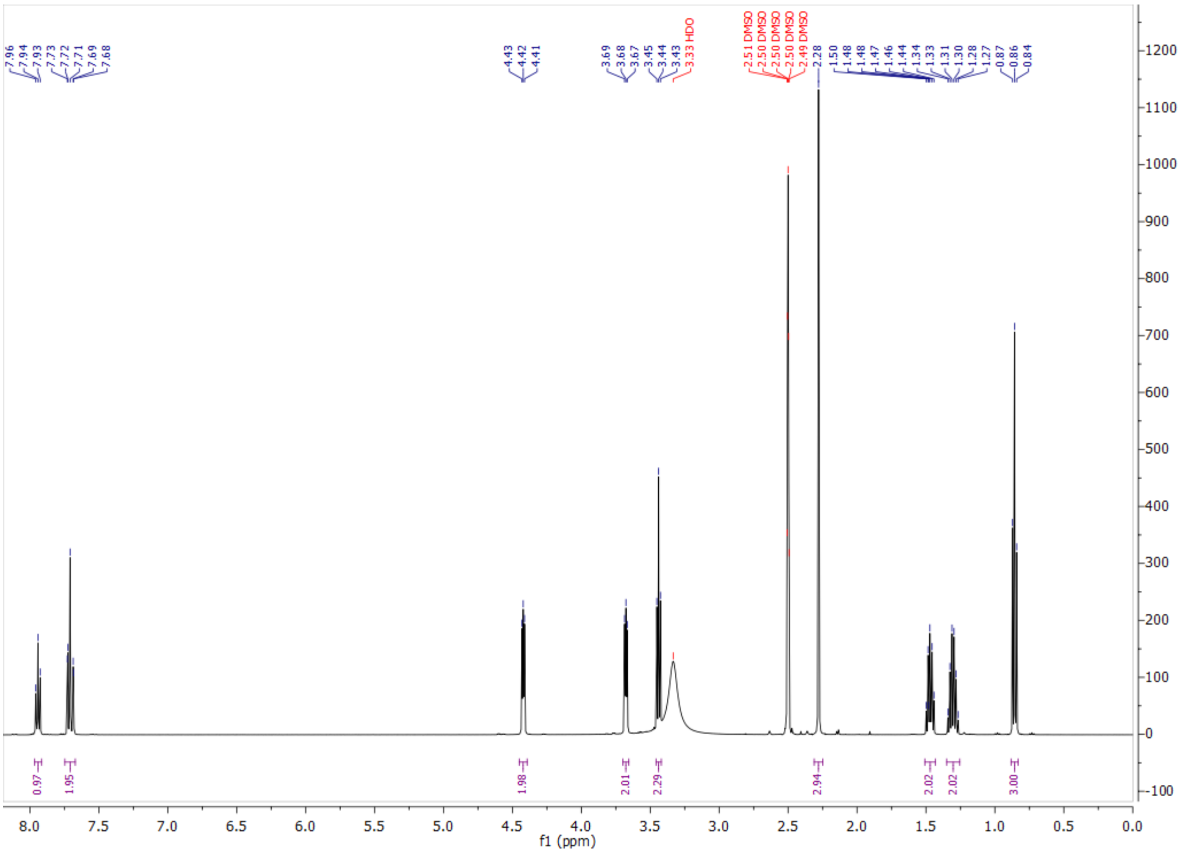

^{13}C NMR

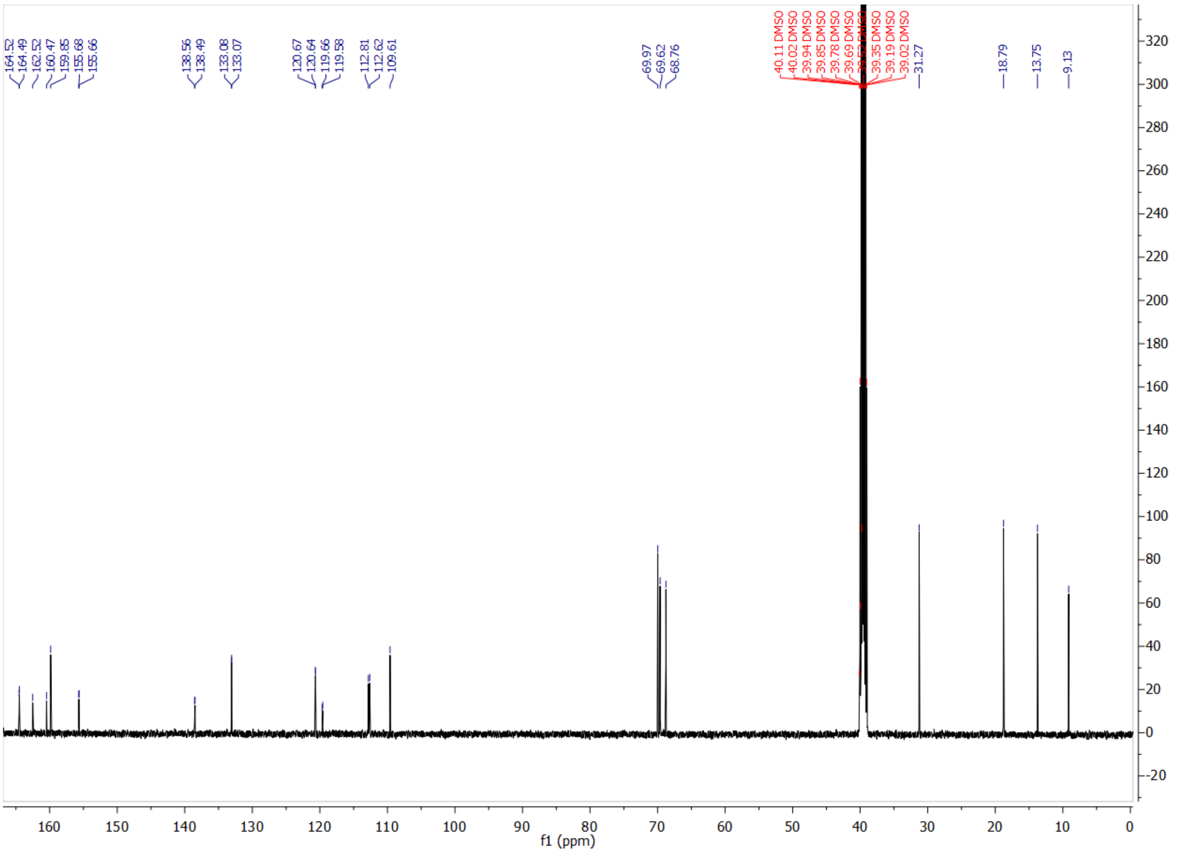

COMPOUND INFORMATION

Purity

Data File W:\analyti...\CGC_wave2_4_FirstPassB 2022-02-18 17-58-18\024-D2F-B12-AC-261066.D
Sample Name: AC-261066

```
=====================================================================
Acq. Operator   : SYSTEM                        Seq. Line :  24
Sample Operator : SYSTEM
Acq. Instrument : LCMS test                      Location :   D2F-B12
Injection Date  : 2/18/2022 10:15:29 PM               Inj :   1
                                               Inj Volume : Inj prog
Sequence File   : W:\analytical_LCMS_DATA\EUbOPEN\CGC_wave2_4_FirstPassB 2022-02-18 17-58-18
                  \CGC_wave2_4_FirstPassB.S
Method          : W:\analytical_LCMS_DATA\EUbOPEN\CGC_wave2_4_FirstPassB 2022-02-18 17-58-18
                  \CGL_FIRSTPASS_GENERALMETHOD_VIAL1+2_20210319.M (Sequence Method)
Last changed    : 1/25/2022 4:36:18 PM by SYSTEM
Method Info     : CGL wellplate, 0.5 uL of 10 mM DMSO, general method
```

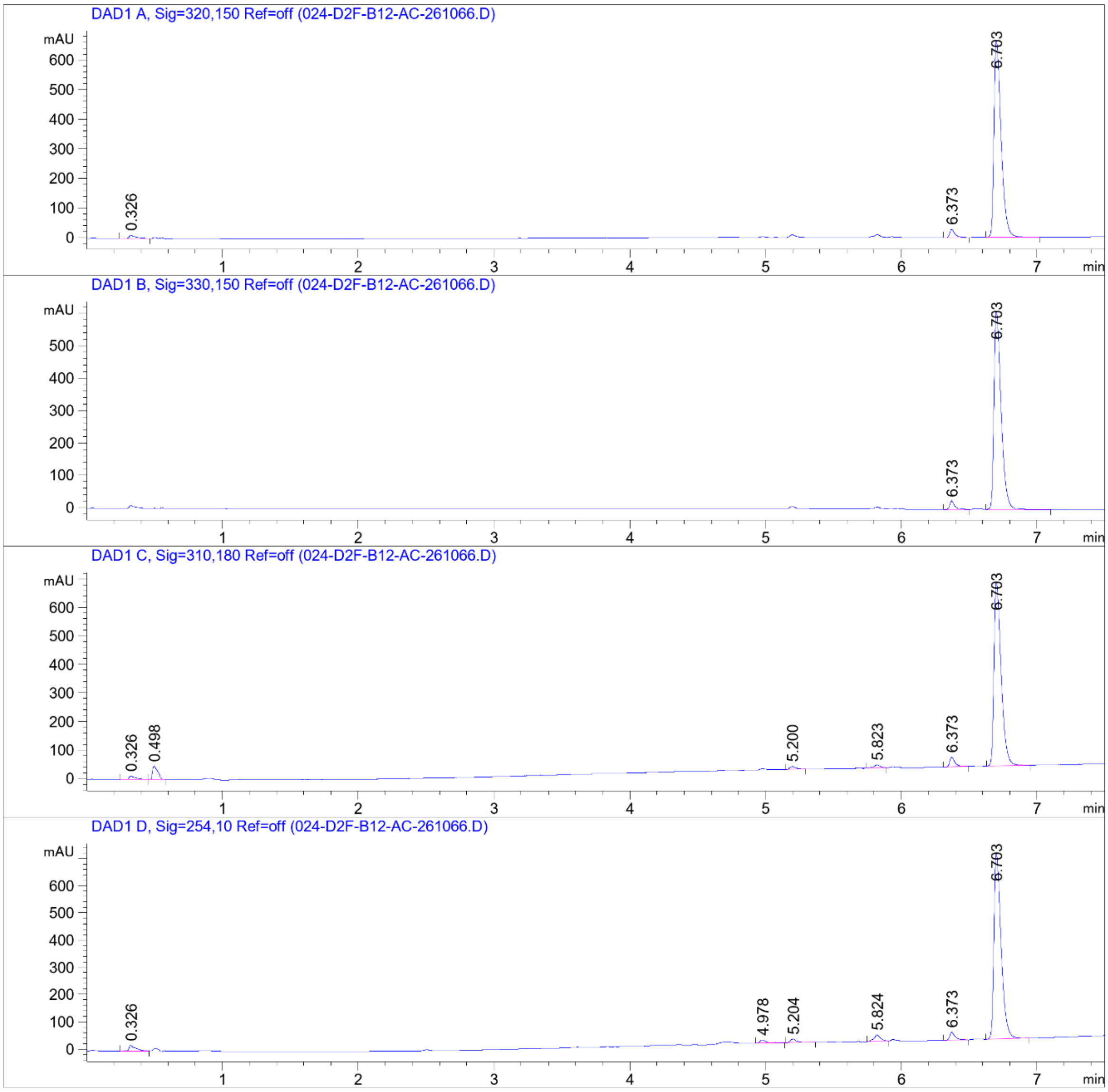

COMPOUND INFORMATION

Data File W:\analyti...\CGC_wave2_4_FirstPassB 2022-02-18 17-58-18\024-D2F-B12-AC-261066.D
Sample Name: AC-261066

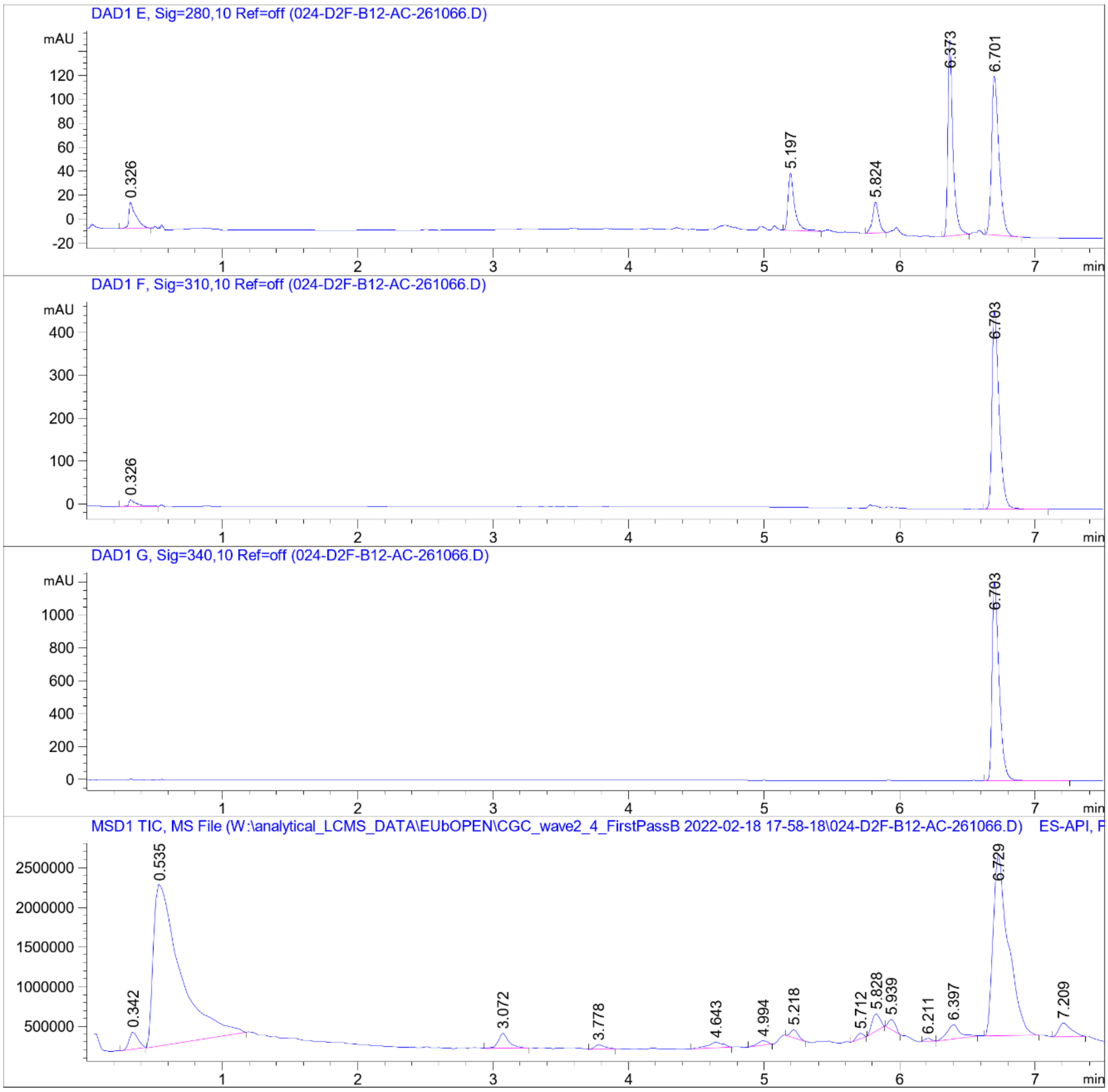

COMPOUND INFORMATION

Data File W:\analyti...\CGC_wave2_4_FirstPassB 2022-02-18 17-58-18\024-D2F-B12-AC-261066.D
Sample Name: AC-261066

MS Signal: MSD1 TIC, MS File, ES-API, Pos, Scan, Frag: 70, "POS Scan"
Spectra from peak tops.
Noise Cutoff: 1000 counts.
Reportable Ion Abundance: > 50%.
LC Signal: DAD1 A, Sig=320,150 Ref=off
Peak matching window: 0.1 min

Retention Time (LC)	LC Area	Retention Time (MS)	MS Area	Mol. Weight or Ion
0.326	38	0.342	972044	183.00 I
				158.90 I
				141.90 I
-	-	0.535	28038024	157.00 I
-	-	3.072	1038603	239.00 I
				217.00 I
-	-	3.778	295049	274.20 I
-	-	4.643	553328	332.20 I
-	-	4.994	278035	350.00 I
				328.10 I
-	-	5.218	395665	316.20 I
				298.20 I
-	-	5.712	208873	280.20 I
-	-	5.828	887903	543.20 I
				318.20 I
				296.20 I
-	-	5.939	467711	318.20 I
				296.20 I
				282.20 I
				280.20 I
-	-	6.211	132407	228.10 I
6.373	72	6.397	1223767	350.20 I
				282.20 I
				254.20 I
				243.00 I
6.703	2412	6.729	18031048	354.10 I
-	-	7.209	1092900	284.20 I
				282.20 I

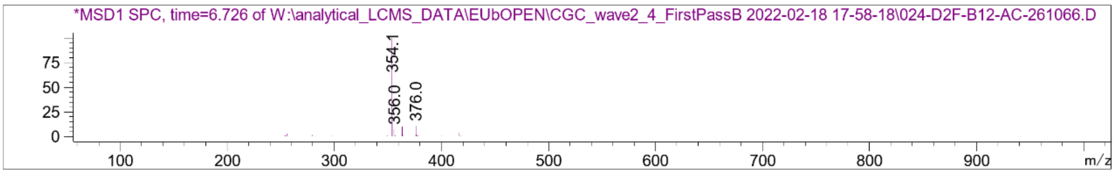

Supplement: Supplementary file 4 — Supplementary Data 1 [file 41467_2024_49493_MOESM4_ESM.zip › AC-261066.pdf]
