# Supplementary material for: Chemogenomics for NR1 nuclear hormone receptors
Source: Nat Commun. 2024 Jun 18;15:5201. doi: 10.1038/s41467-024-49493-6 (PMC11189487; doi:10.1038/s41467-024-49493-6)

COMPOUND INFORMATION

Adapalene

**CAS Registry No.:**	106685-40-9
**Formal Name:**	6-(3-(adamantan-1-yl)-4-methoxyphenyl)-2-naphthoic acid
**EUbOPEN ID:**	EUB0000564a
**Molecular Formula:**	$C_{28}H_{28}O_3$
**Molecular Weight:**	412.53 g/mol
**Smiles:**	COC1=C(C=C(C=C1)C2=CC3=C(C=C2)C=C(C=C3)C(=O)O)C45CC6CC(C4)CC(C6)C5
**Recommended concentration:**	1 µM

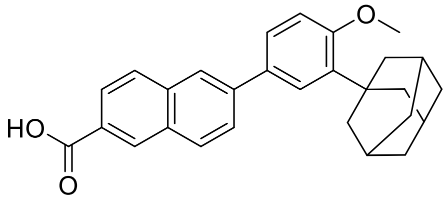

Biological activity

		Type	IC_{50}/EC_{50} [µM]	Reference
Main NR target:	NR1B1 (RARα)	Agonist	0.02	https://doi.org/10.1016/j.bmcl.2018.04.036
	NR1B2 (RARβ)	Agonist	0.002	
	NR1B3 (RARγ)	Agonist	0.003	
NR off-target:				

COMPOUND INFORMATION

Identity

^{1}H NMR

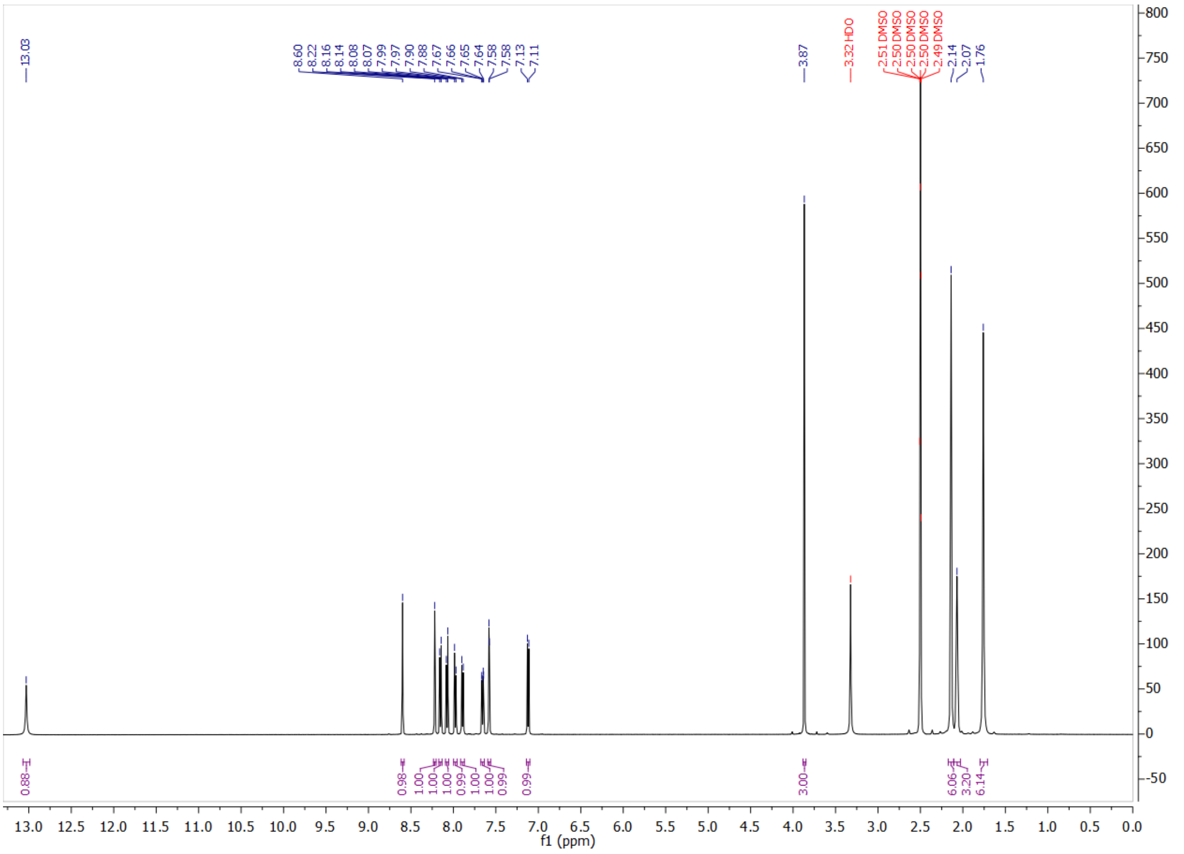

^{13}C NMR

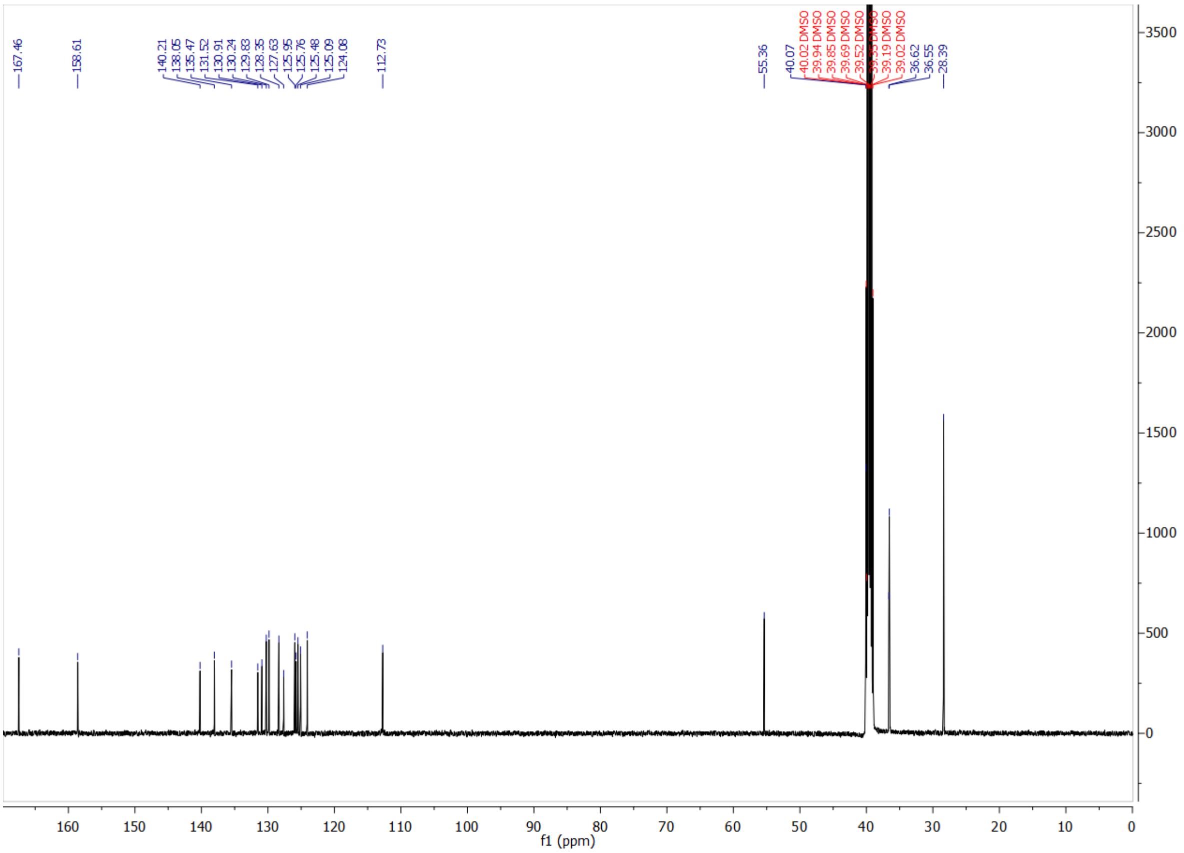

COMPOUND INFORMATION

Purity

M_r 412.53

MS: ESI-negative, *m/z* 411/367

LC: 0.1% HCOOH/ACN (20/80)

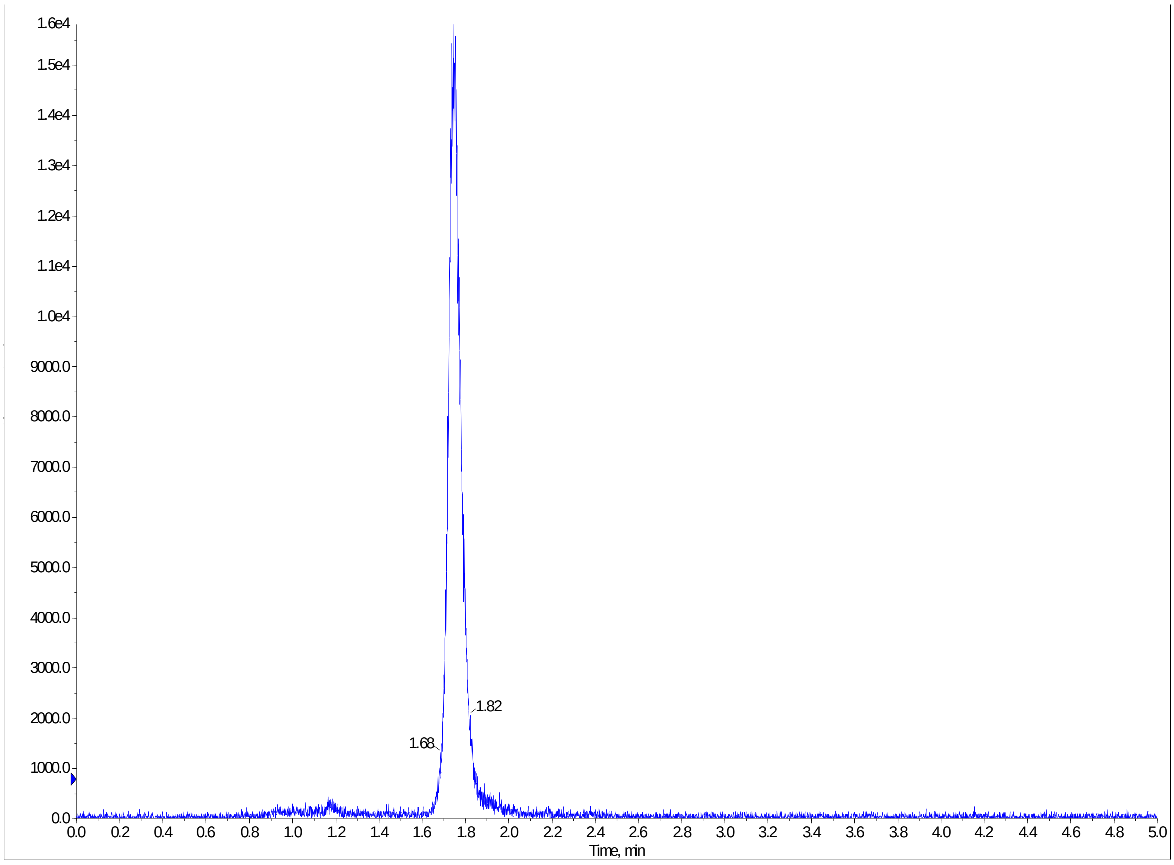

COMPOUND INFORMATION

LC-UV

LC: 0.1% HCOOH/ACN (30/70)

DAD: 210, 230, 240, 254 (XWC), 280 nm

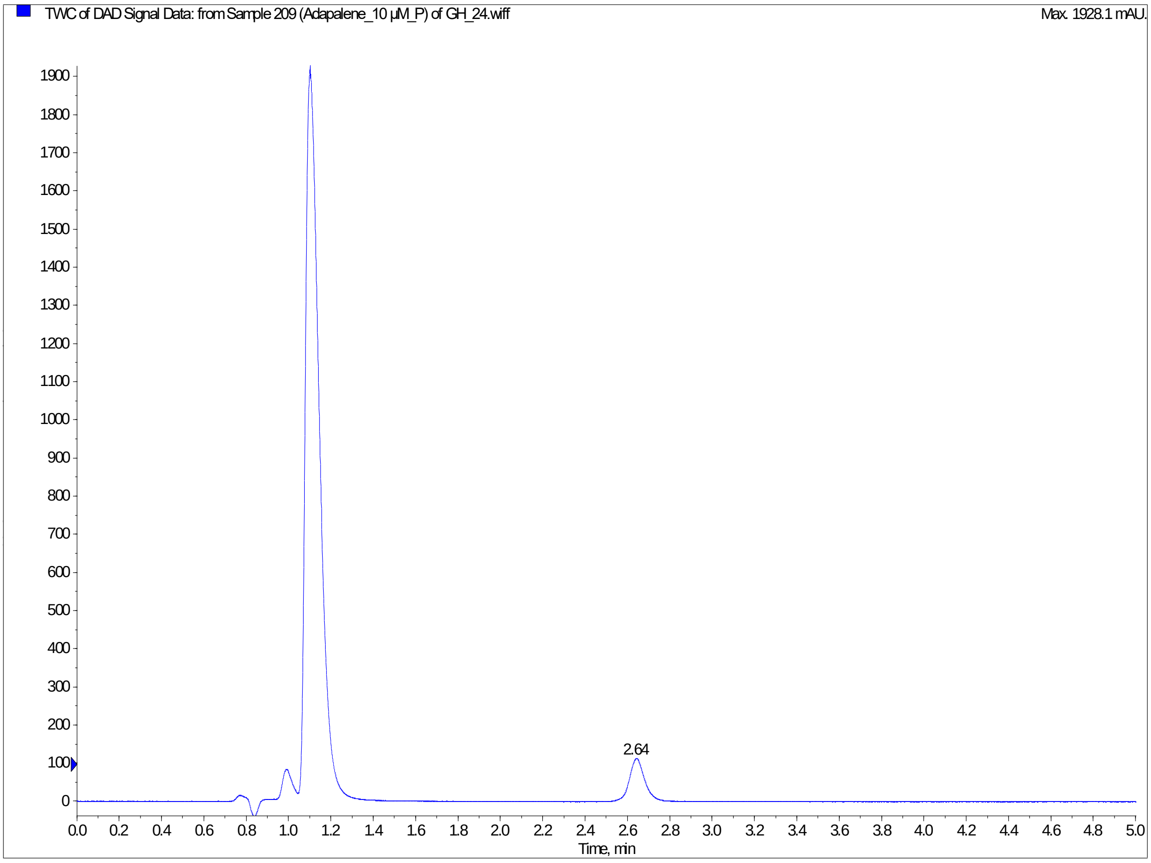

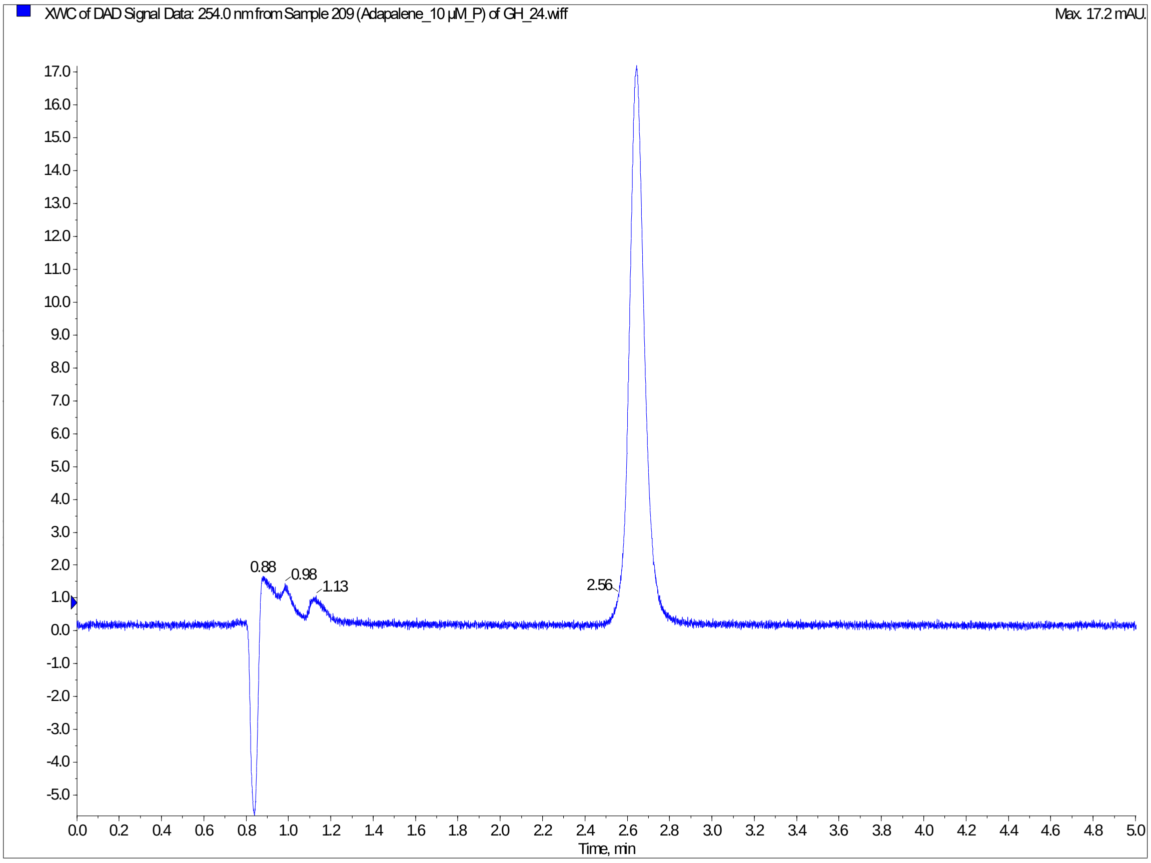

Supplement: Supplementary file 4 — Supplementary Data 1 [file 41467_2024_49493_MOESM4_ESM.zip › Adapalene.pdf]
