# Supplementary material for: Chemogenomics for NR1 nuclear hormone receptors
Source: Nat Commun. 2024 Jun 18;15:5201. doi: 10.1038/s41467-024-49493-6 (PMC11189487; doi:10.1038/s41467-024-49493-6)

COMPOUND INFORMATION

AHPN (CD437)

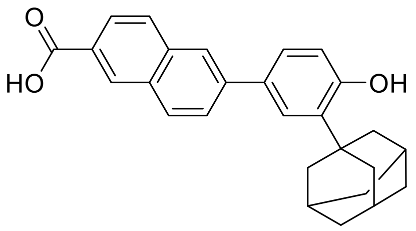

**CAS Registry No.:**	125316-60-1
**Formal Name:**	6-(3-(adamantan-1-yl)-4-hydroxyphenyl)-2-naphthoic acid
**EUbOPEN ID:**	EUB0000568a
**Molecular Formula:**	$C_{27}H_{26}O_3$
**Molecular Weight:**	398.50 g/mol
**Smiles:**	C1C2CC3CC1CC(C2)(C3)C4=C(C=CC(=C4)C5=CC6=C(C=C5)C=C(C=C6)C(=O)O)O
**Recommended concentration:**	1 µM

Biological activity

		Type	IC_{50}/EC_{50} [µM]	Reference
Main NR target:	NR1B2 (RARβ)	Agonist	0.2	https://doi.org/10.1016/j.bmcl.2018.04.036
	NR1B3 (RARγ)	Agonist	0.05	
NR off-target:				

COMPOUND INFORMATION

Identity

^{1}H NMR

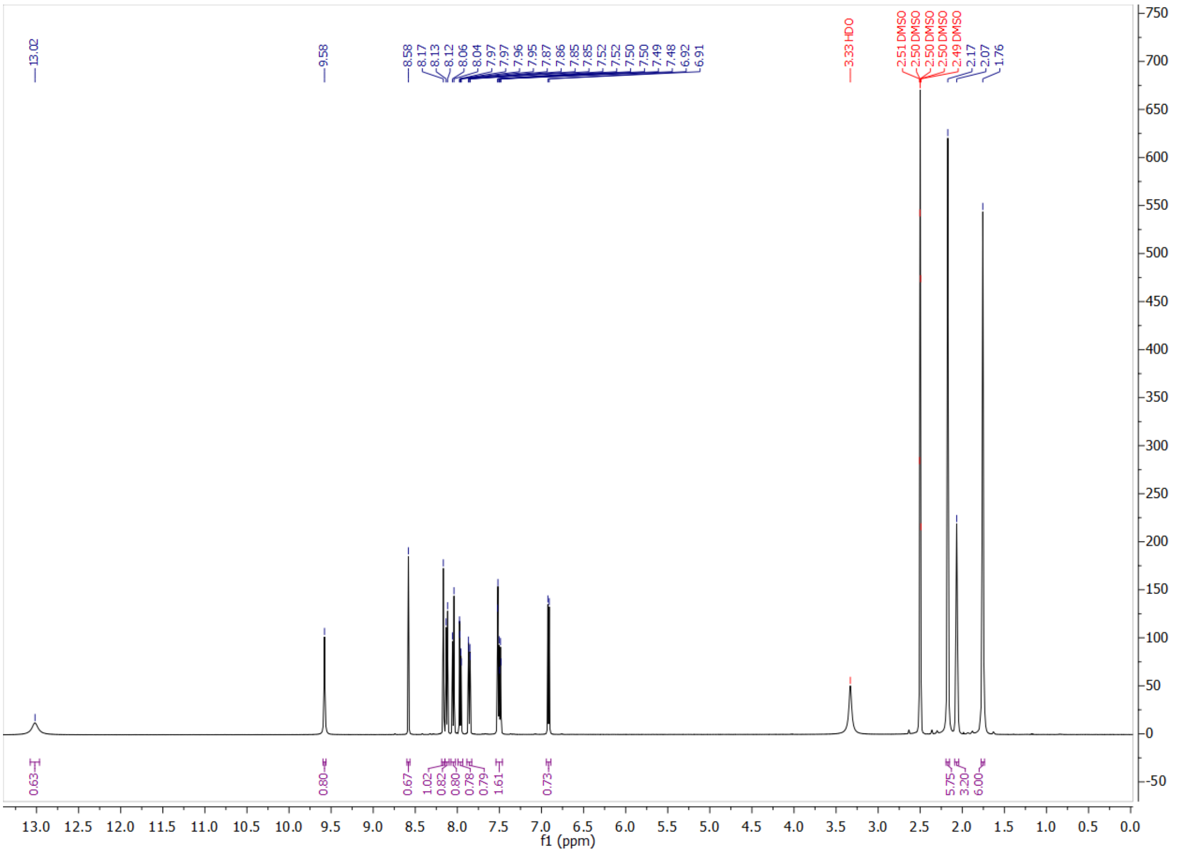

^{13}C NMR

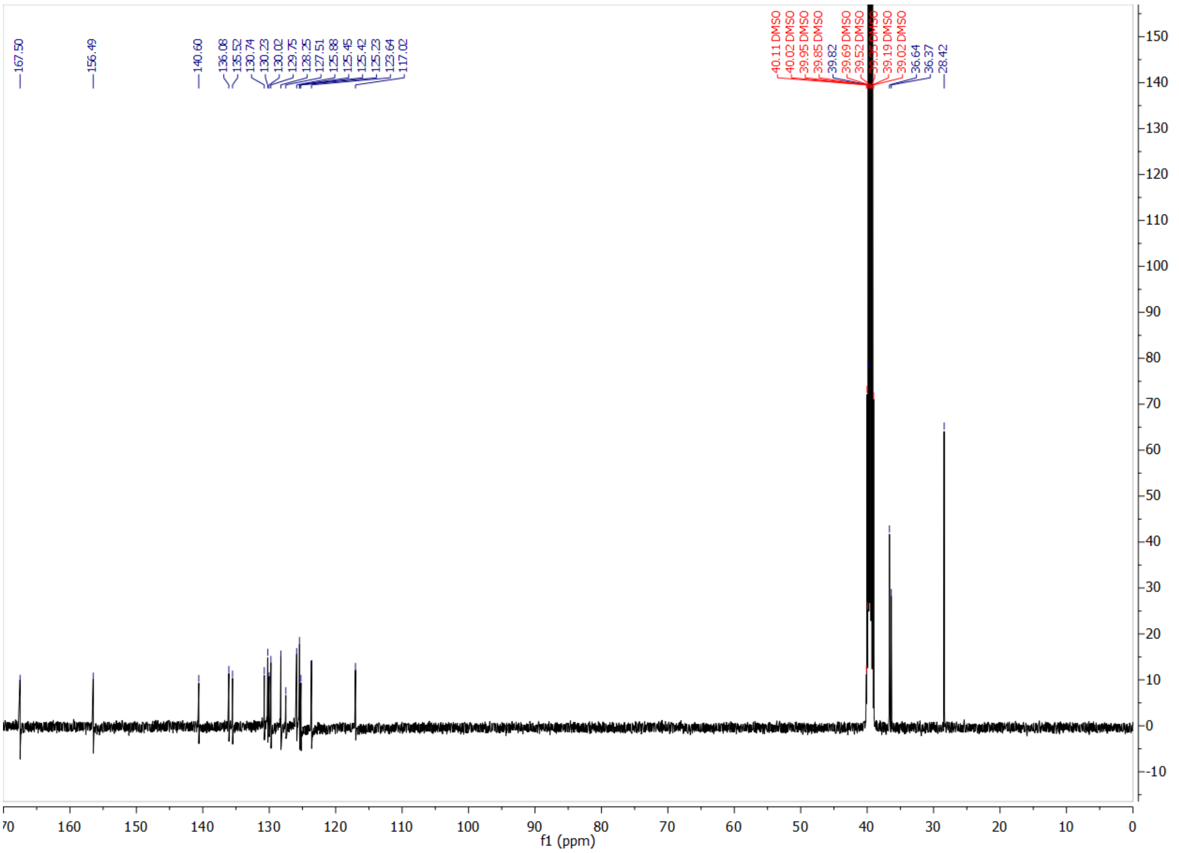

COMPOUND INFORMATION

Purity

M_r 398.50

MS: ESI-negative, *m/z* 397/353

LC: 0.1% HCOOH/ACN (30/70)

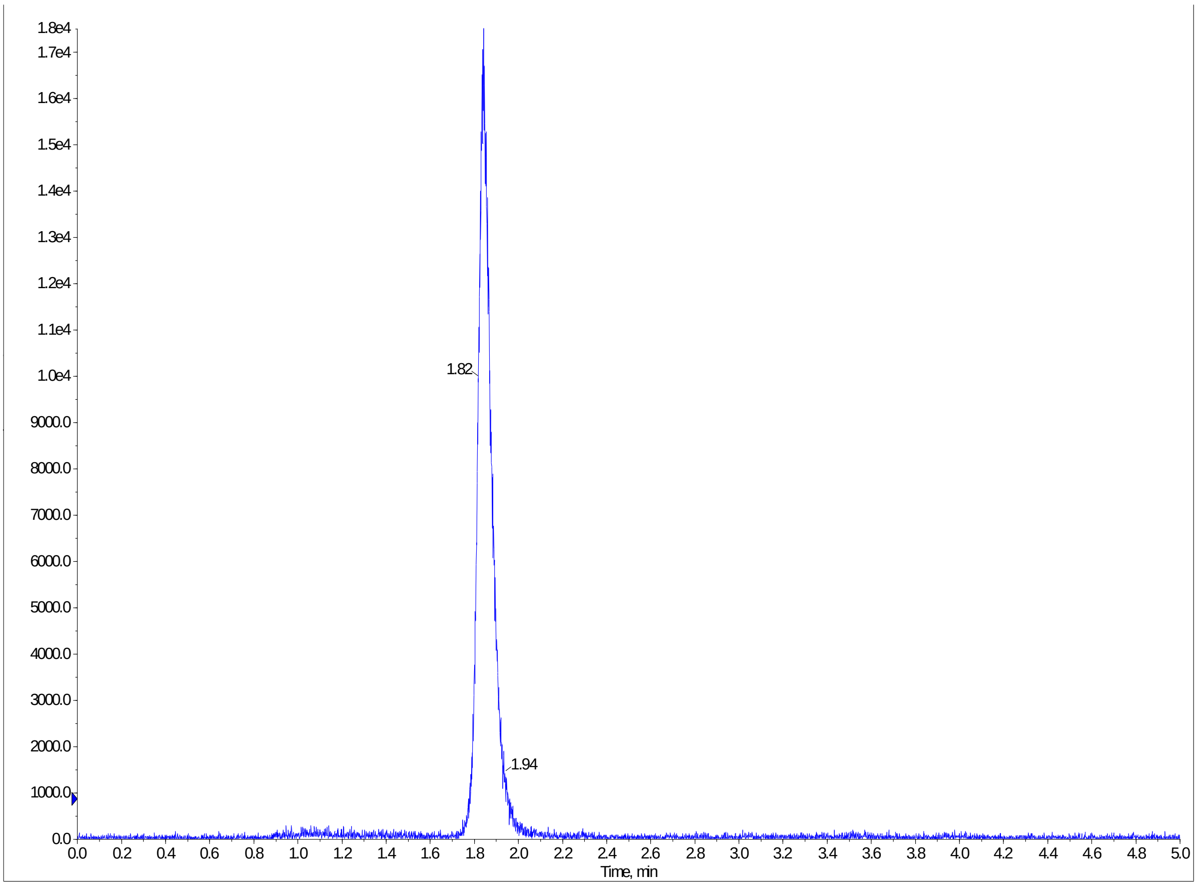

COMPOUND INFORMATION

LC-UV

LC: 0.1% HCOOH/ACN (30/70)

DAD: 230, 254, 270 (XWC), 280, 300 nm

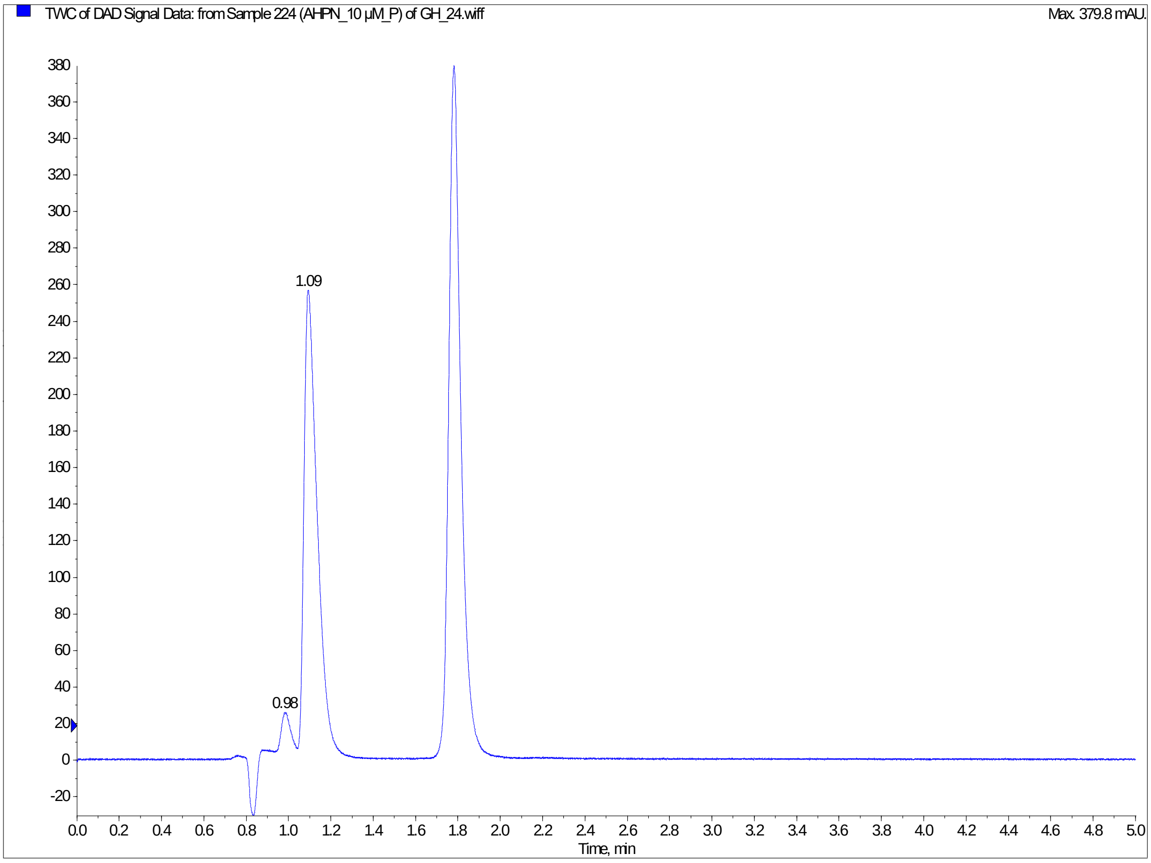

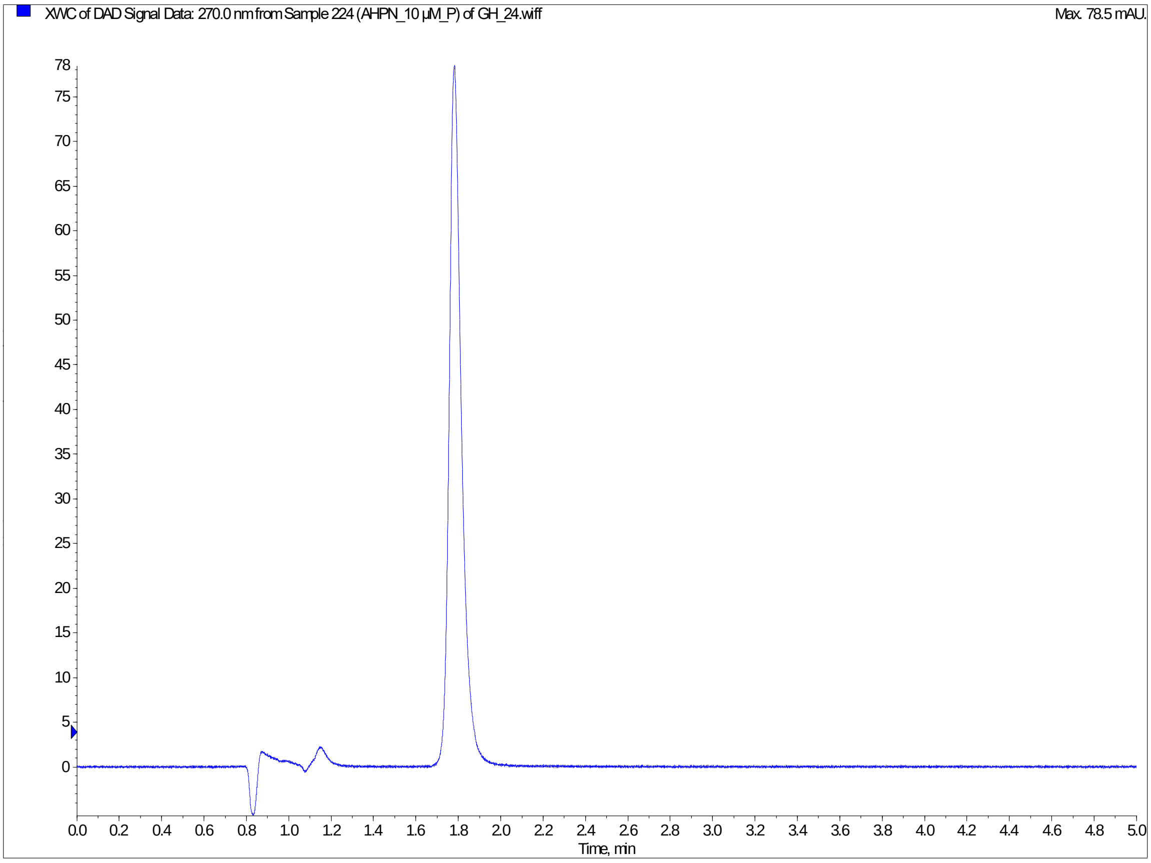

Supplement: Supplementary file 4 — Supplementary Data 1 [file 41467_2024_49493_MOESM4_ESM.zip › AHPN.pdf]
