# Supplementary material for: Chemogenomics for NR1 nuclear hormone receptors
Source: Nat Commun. 2024 Jun 18;15:5201. doi: 10.1038/s41467-024-49493-6 (PMC11189487; doi:10.1038/s41467-024-49493-6)

COMPOUND INFORMATION

APAA (Acetyl podocarpic acid anhydride)

**CAS Registry No.:**	344327-48-6
**Formal Name:**	6-acetoxy-1,4a-dimethyl-1,2,3,4,4a,9,10,10a-octahydrophenanthrene-1-carboxylic anhydride
**EUBOPEN ID:**	EUB0001167a
**Molecular Formula:**	$C_{38}H_{46}O_7$
**Molecular Weight:**	614.78 g/mol
**Smiles:**	CC(=O)OC1=CC2=C(CCC3C2(CCCC3(C)C(=O)OC(=O)C4(CCCC5(C4CCC6=C5C=C(C=C6)OC(=O)C)C)C)C)C=C1
**Recommended concentration:**	1 µM

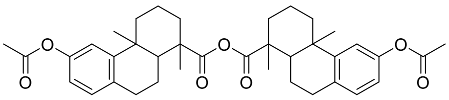

Biological activity

		Type	IC_{50}/EC_{50} [µM]	Reference
Main NR target:	NR1H3 (LXRα)	Agonist	0.001	https://doi.org/10.1074/jbc.M108225200
	NR1H2 (LXRβ)	Agonist	0.001	
NR off-target:				

COMPOUND INFORMATION

Identity

1H NMR

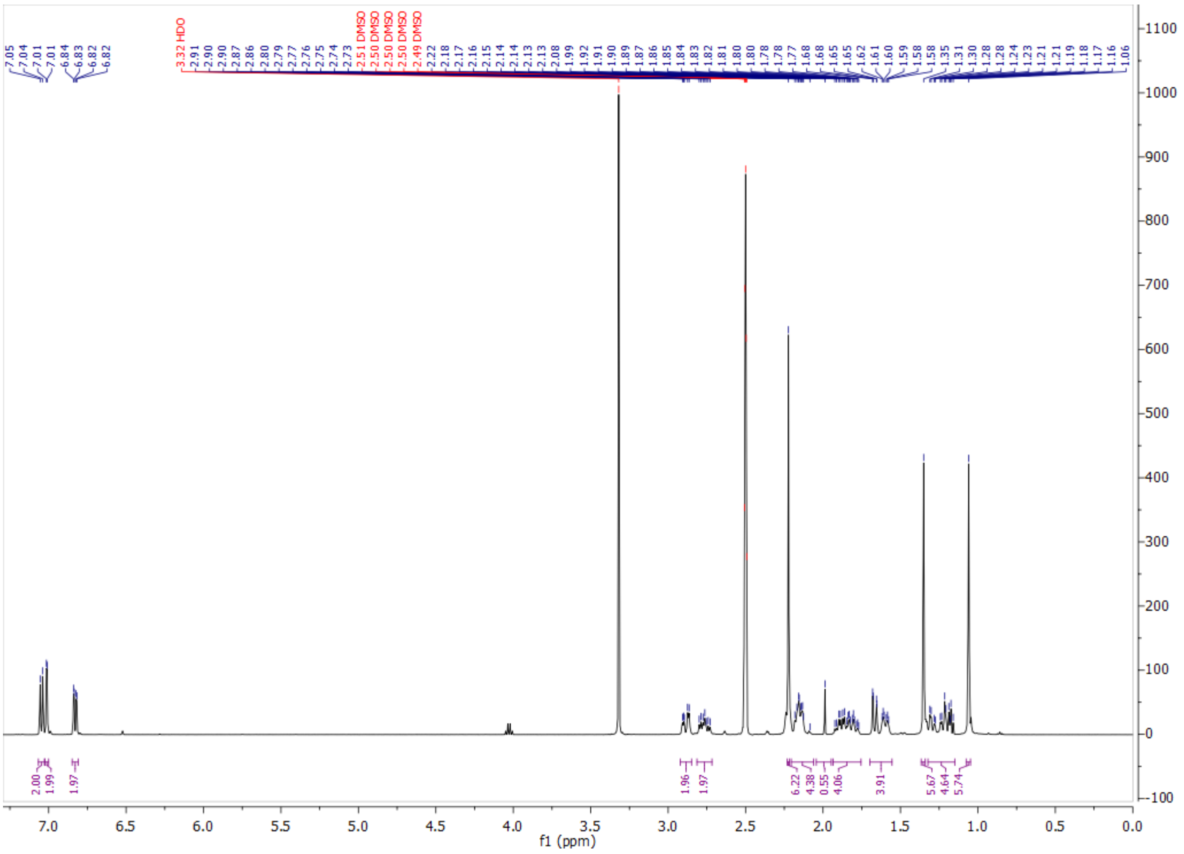

13C NMR

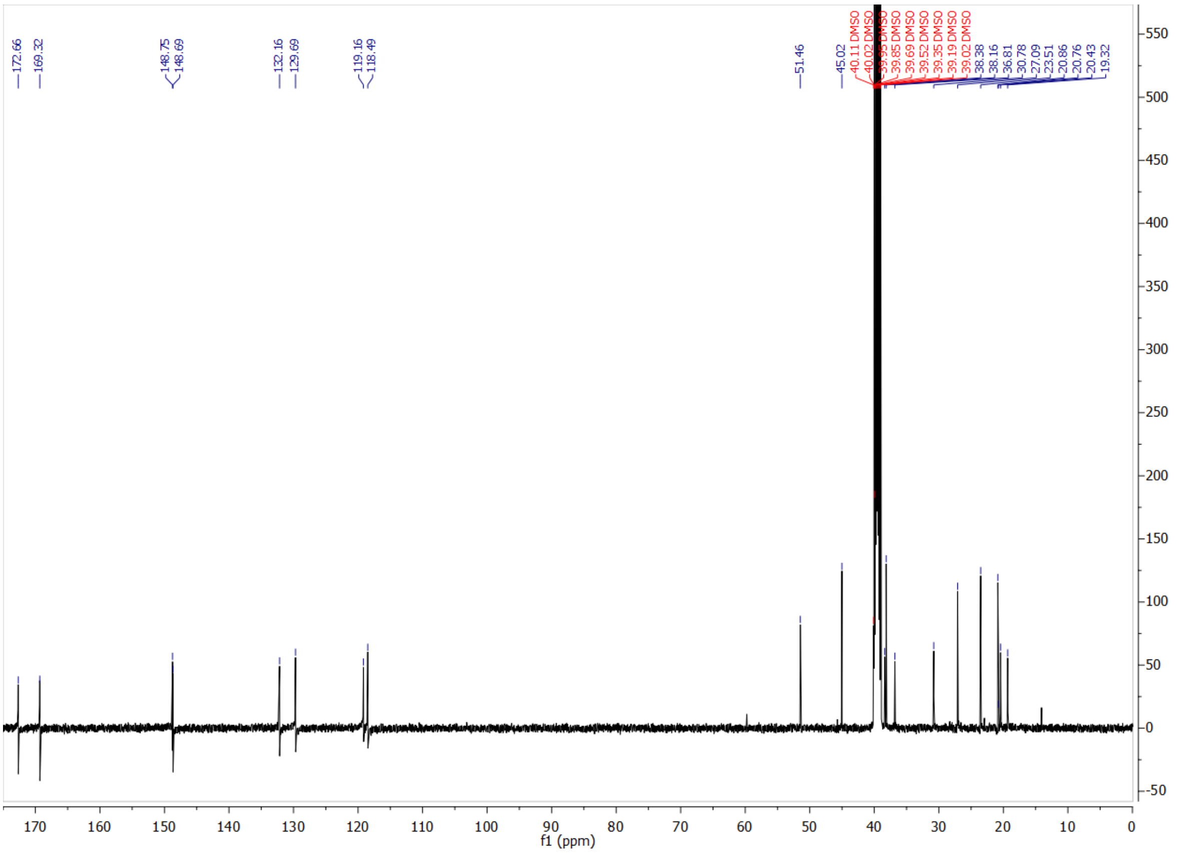

COMPOUND INFORMATION

Purity

M_r 614.8

MS: ESI-positive, m/z 615/271 (disolved and diluted in acetonitrile)

LC: 0.1% HCOOH/ACN (15/85)

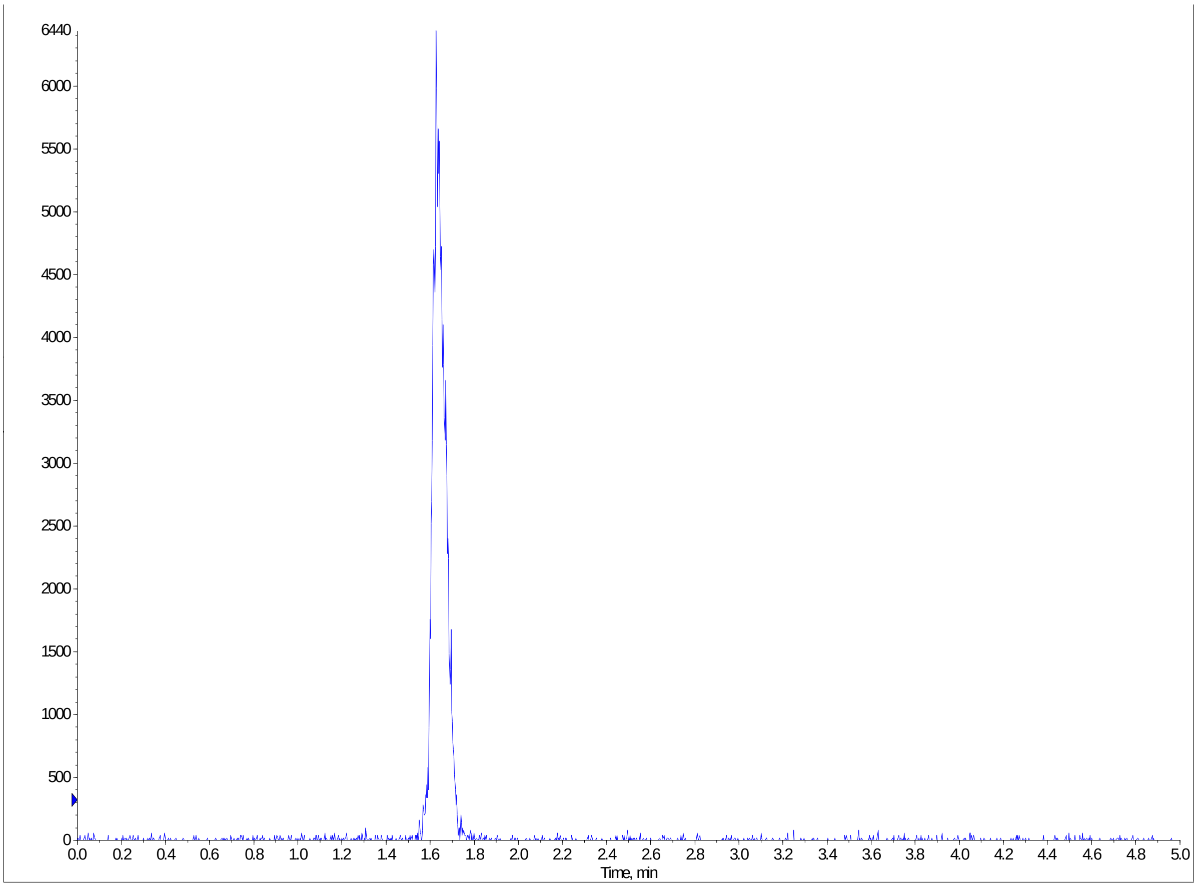

COMPOUND INFORMATION

LC-UV

LC: 0.1% HCOOH/ACN (10/90), stock solution in acetonitrile

DAD: 210, 230, 240, 254, 268 (XWC) nm

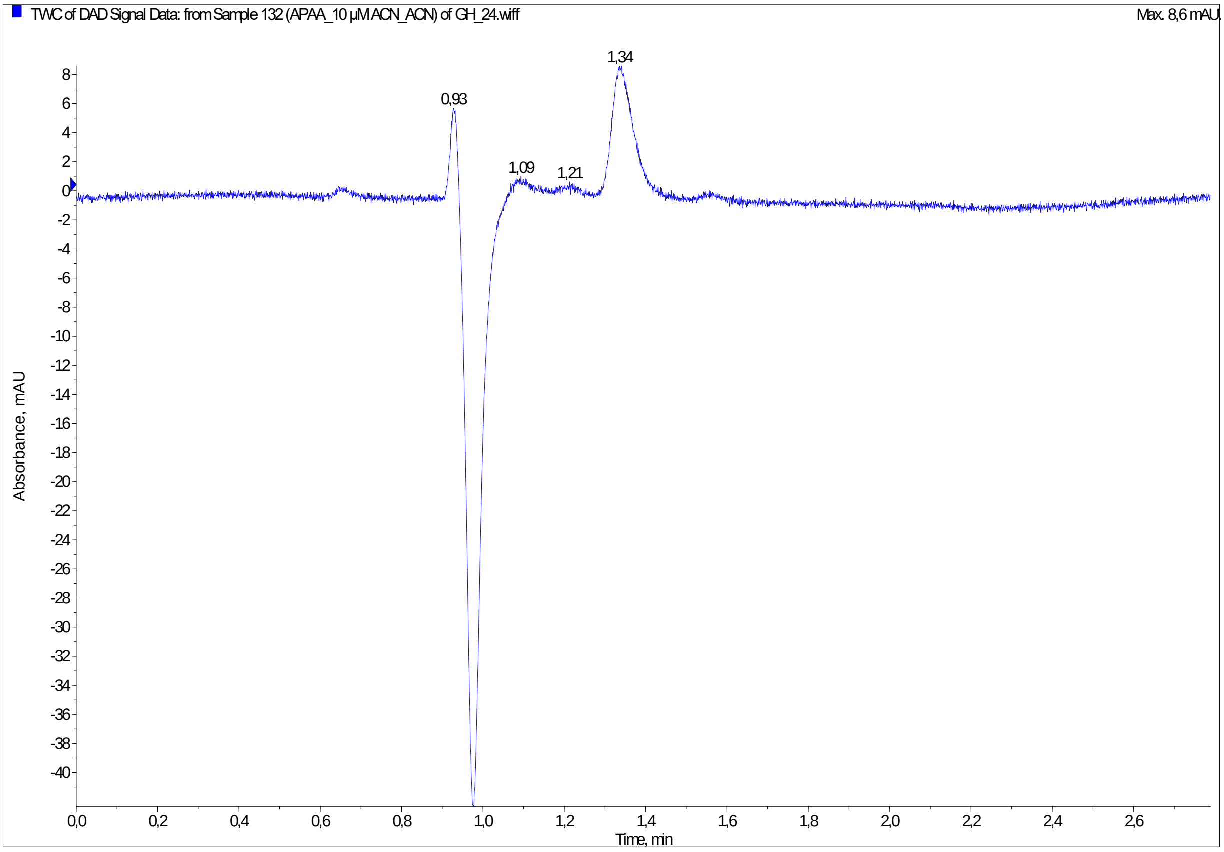

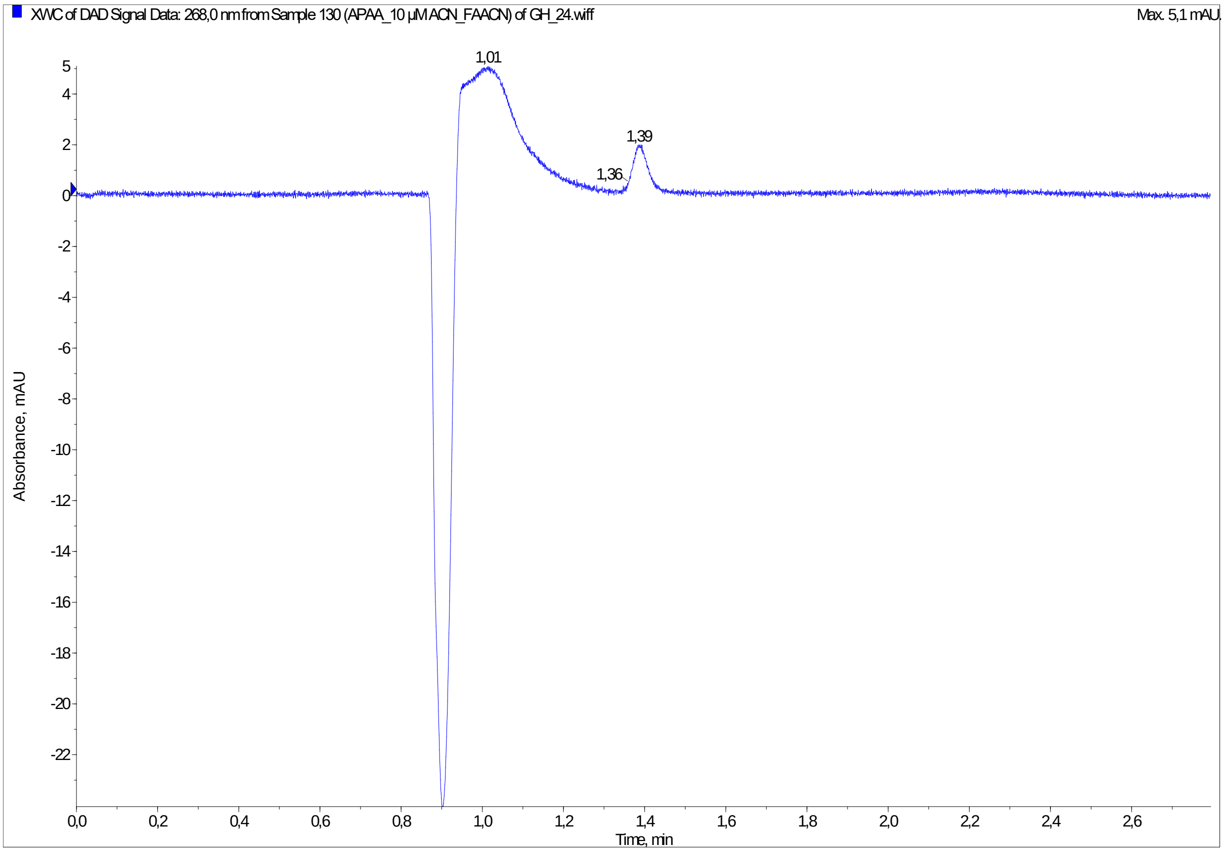

COMPOUND INFORMATION

LC-ELSD

LC: 0.1% HCOOH/ACN (20/80), sample concentration 100 µM

DAD: 268 nm

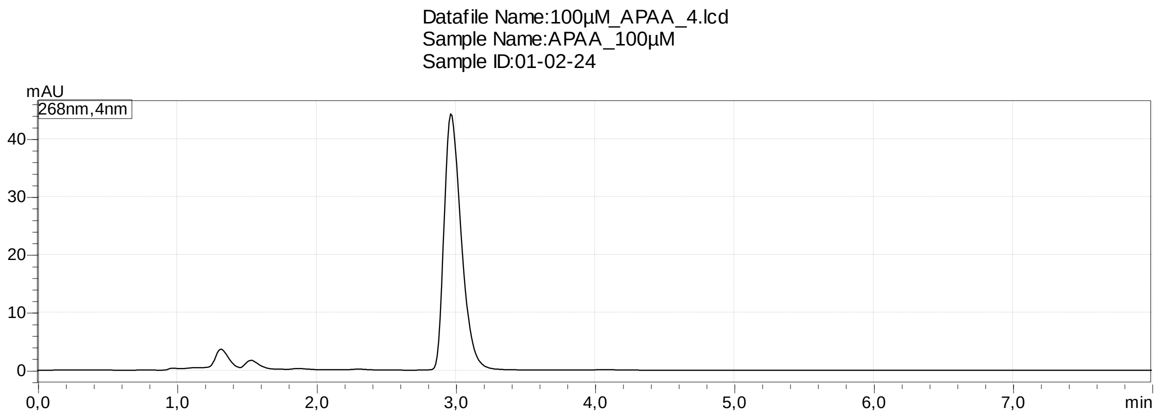

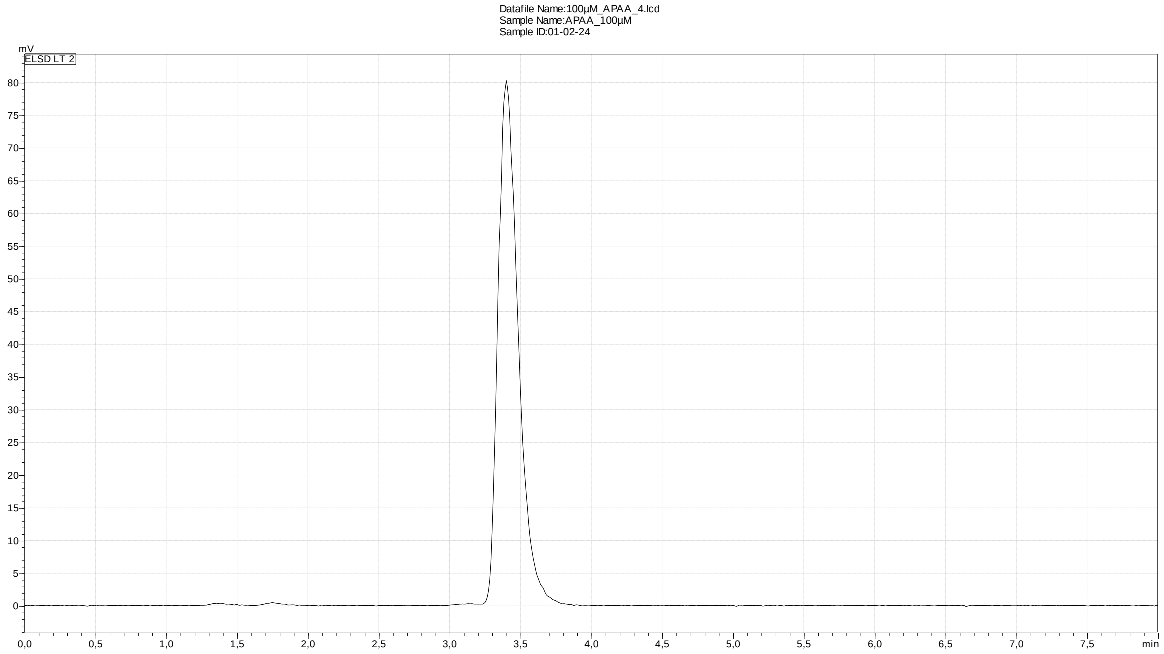

Supplement: Supplementary file 4 — Supplementary Data 1 [file 41467_2024_49493_MOESM4_ESM.zip › APAA.pdf]
