# Supplementary material for: Chemogenomics for NR1 nuclear hormone receptors
Source: Nat Commun. 2024 Jun 18;15:5201. doi: 10.1038/s41467-024-49493-6 (PMC11189487; doi:10.1038/s41467-024-49493-6)

COMPOUND INFORMATION

Bavachinin

**CAS Registry No.:**	19879-30-2
**Formal Name:**	(S)-2-(4-hydroxyphenyl)-7-methoxy-6-(3-methylbut-2-en-1-yl)chroman-4-one
**EUbOPEN ID:**	EUB0001143a
**Molecular Formula:**	$C_{21}H_{22}O_4$
**Molecular Weight:**	338.40 g/mol
**Smiles:**	CC(=CCC1=CC2=C(C=C1OC)OC(CC2=O)C3=CC=C(C=C3)O)C
**Recommended concentration:**	1 µM

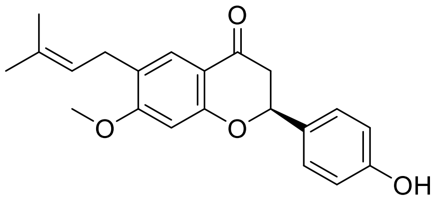

Biological activity

		Type	IC_{50}/EC_{50} [µM]	Reference
Main NR target:	NR1C3 (PPARγ)	Agonist	0.7	https://doi.org/10.1007/s00125-016-3912-9
NR off-target:				

COMPOUND INFORMATION

Identity

^{1}H NMR

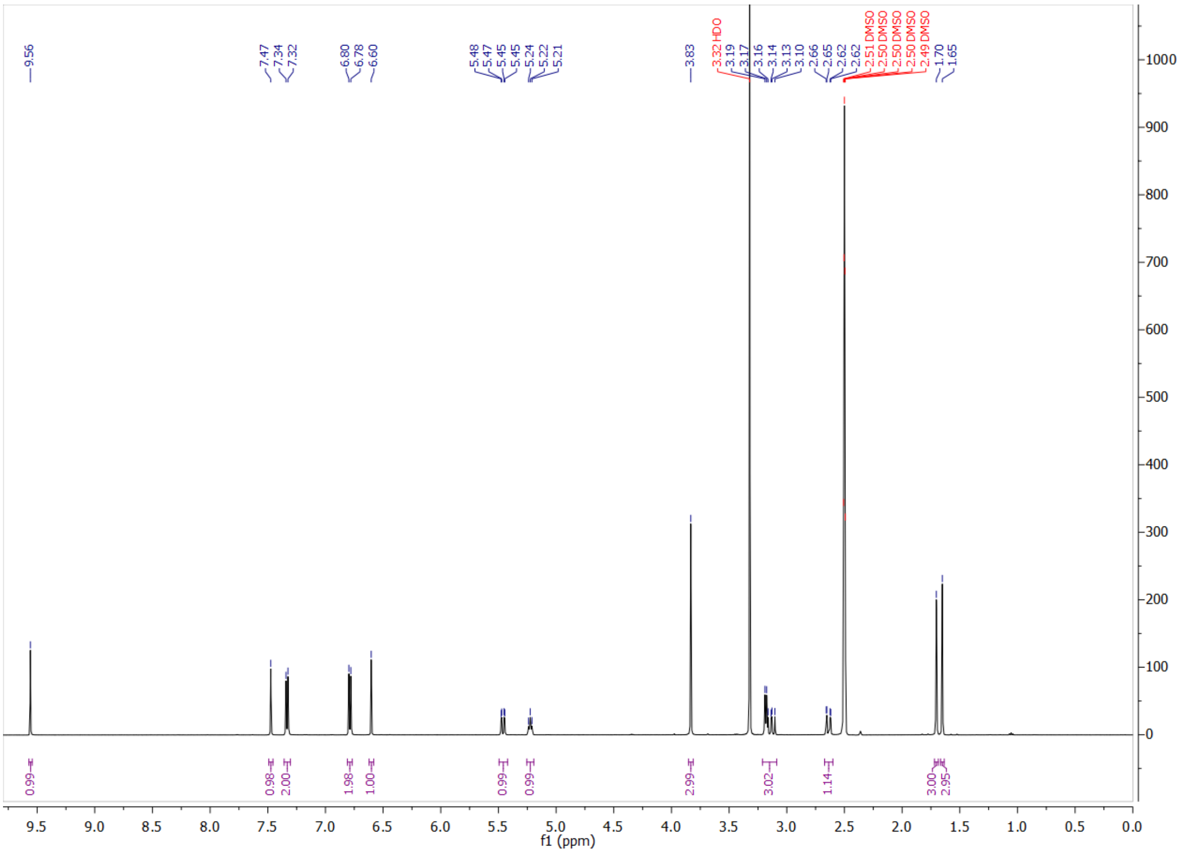

^{13}C NMR

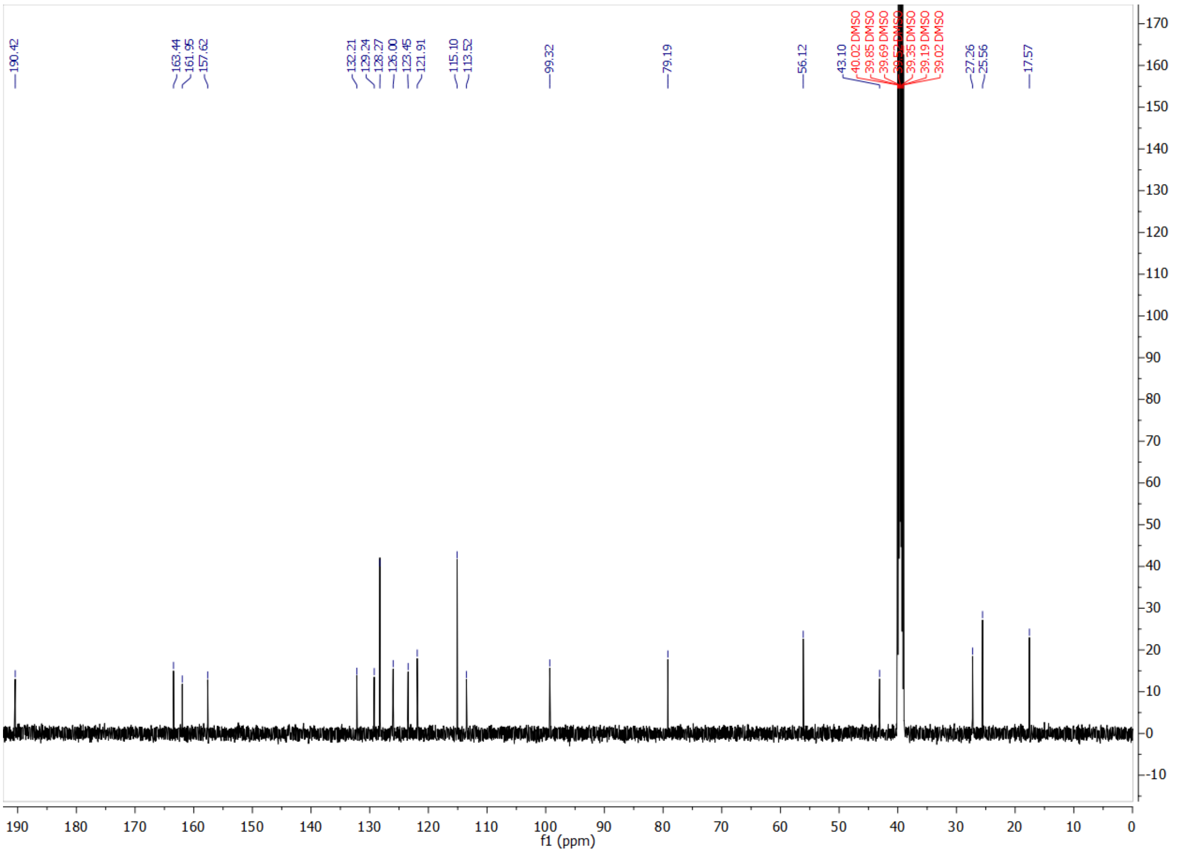

COMPOUND INFORMATION

Purity

Data File W:\analyti...\CGC_wave3_1_FirstPassB 2023-01-04 18-28-02\061-D2F-F1-Bavachinin.D
Sample Name: Bavachinin

```
=====================================================================
Acq. Operator   : SYSTEM                          Seq. Line :  61
Sample Operator : SYSTEM
Acq. Instrument : LCMS test                        Location :  D2F-F1
Injection Date  : 1/5/2023 5:34:18 AM                   Inj :  1
                                                Inj Volume : Inj prog
Sequence File   : W:\analytical_LCMS_DATA\EUbOPEN\CGC_wave3_1_FirstPassB 2023-01-04 18-28-02
                  \CGC_wave3_1_FirstPassB.S
Method          : W:\analytical_LCMS_DATA\EUbOPEN\CGC_wave3_1_FirstPassB 2023-01-04 18-28-02
                  \CGL_FIRSTPASS_GENERALMETHOD_VIAL1+2_20210319.M (Sequence Method)
Last changed    : 1/25/2022 4:36:18 PM by SYSTEM
Method Info     : CGL wellplate, 0.5 uL of 10 mM DMSO, general method
```

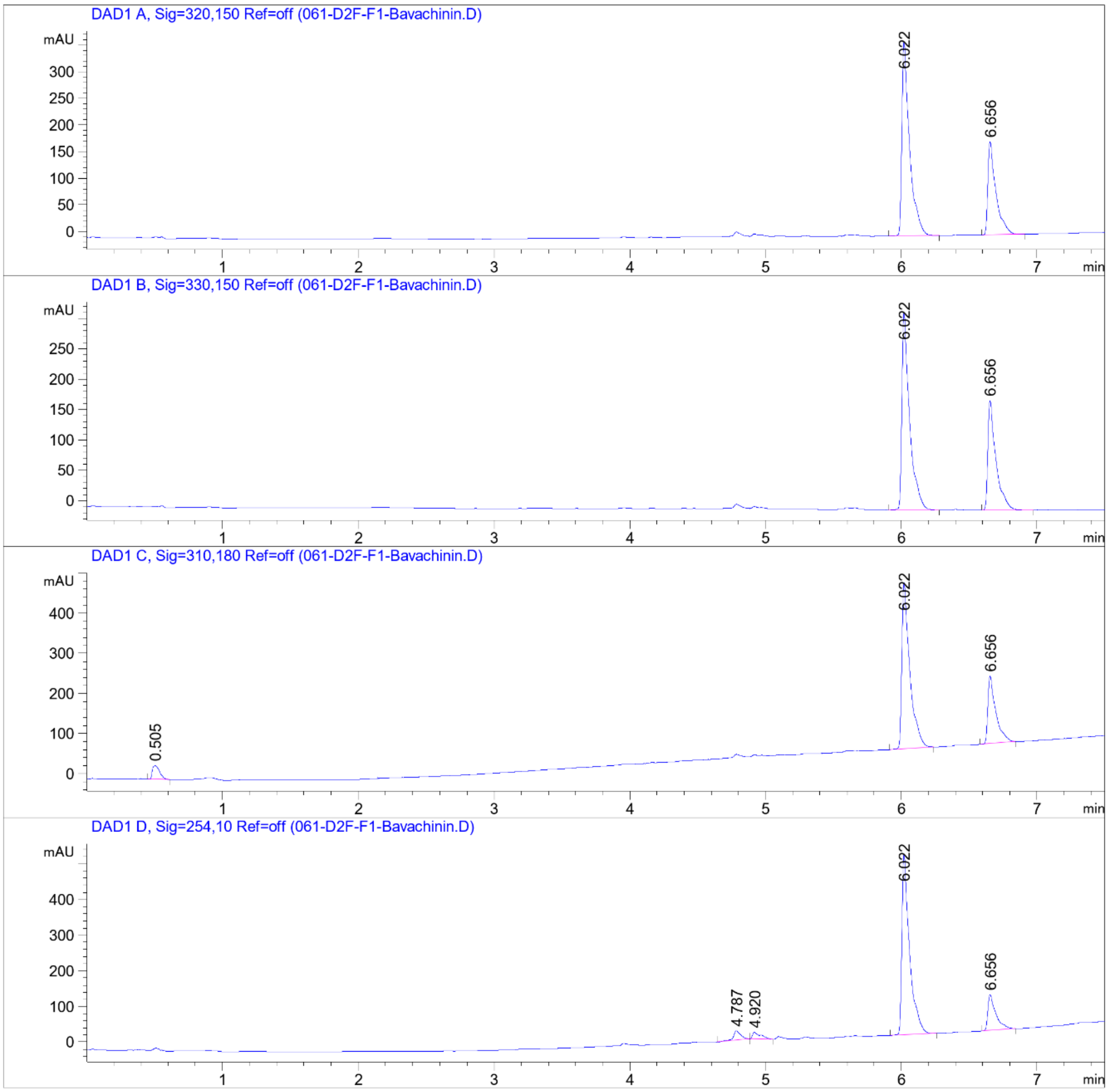

COMPOUND INFORMATION

Data File W:\analyti...\CGC_wave3_1_FirstPassB 2023-01-04 18-28-02\061-D2F-F1-Bavachinin.D
Sample Name: Bavachinin

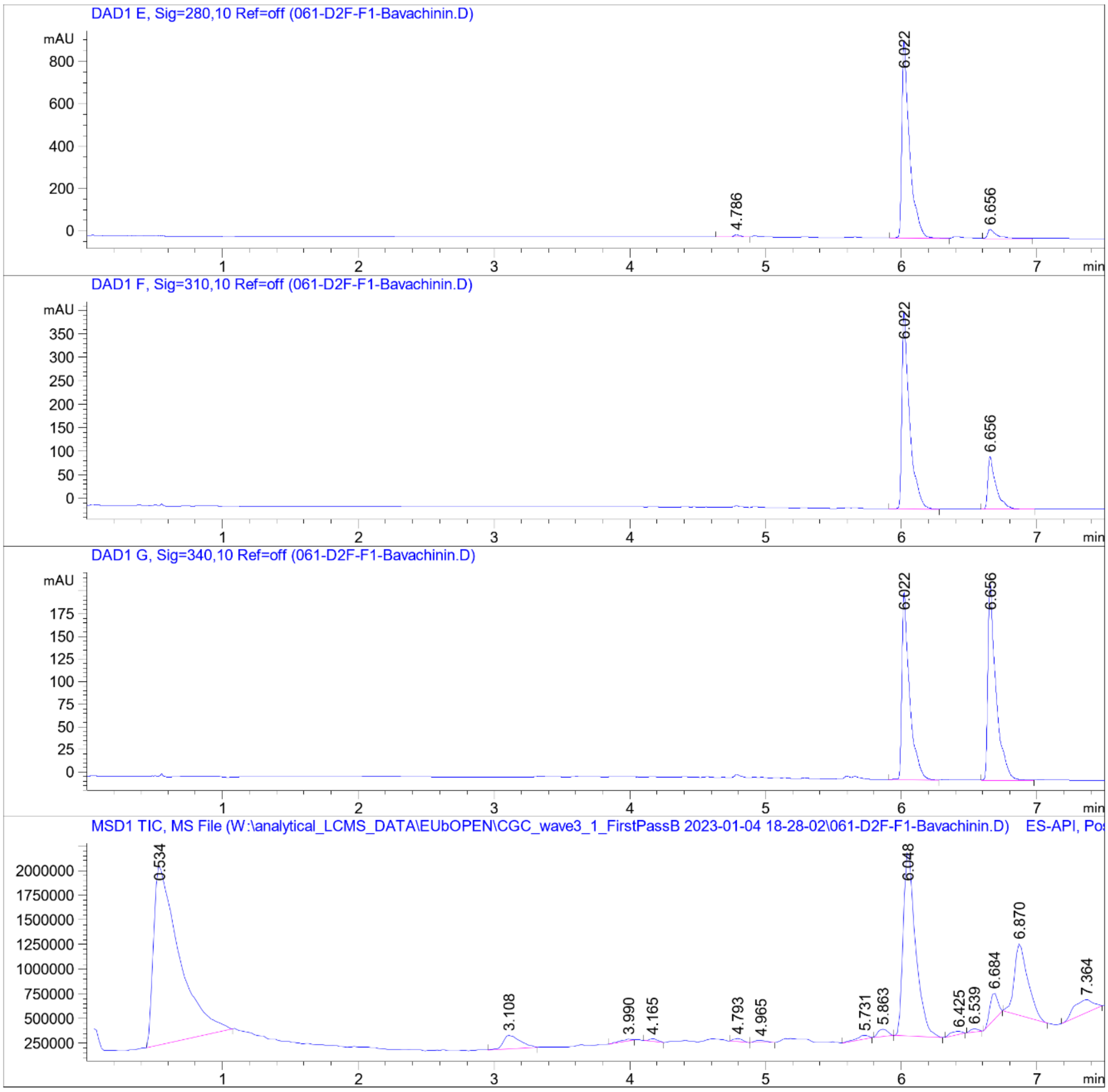

COMPOUND INFORMATION

Data File W:\analyti...\CGC_wave3_1_FirstPassB 2023-01-04 18-28-02\061-D2F-F1-Bavachinin.D
Sample Name: Bavachinin

MS Signal: MSD1 TIC, MS File, ES-API, Pos, Scan, Frag: 70, "POS Scan"
Spectra from peak tops.
Noise Cutoff: 1000 counts.
Reportable Ion Abundance: > 50%.
LC Signal: DAD1 A, Sig=320,150 Ref=off
Peak matching window: 0.1 min

Retention Time (LC)	LC Area	Retention Time (MS)	MS Area	Mol. Weight or Ion
-	-	0.534	23765002	157.00 I
-	-	3.108	1162701	239.10 I<br>217.10 I
-	-	3.990	116212	170.80 I
-	-	4.165	116257	170.80 I
-	-	4.793	145928	510.30 I<br>170.90 I
-	-	4.965	128429	510.30 I<br>170.90 I
-	-	5.731	224163	280.20 I
-	-	5.863	354805	318.20 I<br>296.20 I
6.022	1425	6.048	11734022	339.10 I
-	-	6.425	208327	350.20 I<br>282.20 I<br>254.20 I<br>137.10 I
-	-	6.539	132791	280.20 I
6.656	716	6.684	1056838	339.10 I
-	-	6.870	5085602	282.20 I
-	-	7.364	1380681	400.30 I<br>282.30 I

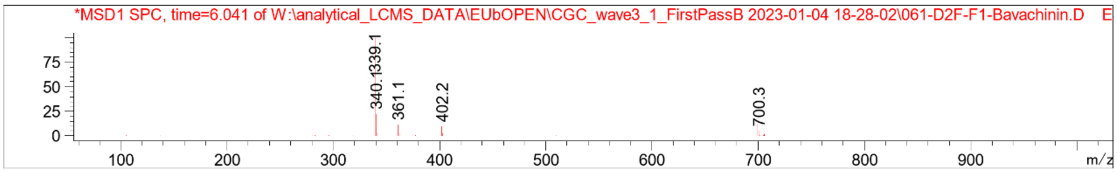

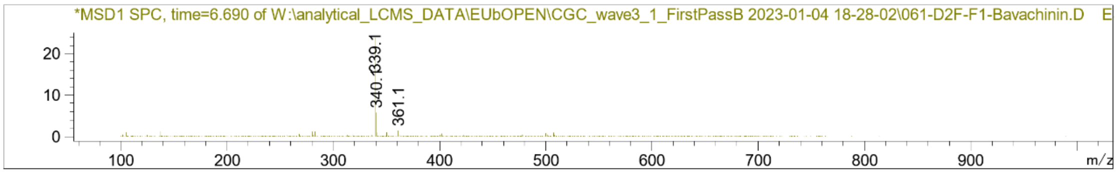

Supplement: Supplementary file 4 — Supplementary Data 1 [file 41467_2024_49493_MOESM4_ESM.zip › Bavachinin.pdf]
