# Supplementary material for: Chemogenomics for NR1 nuclear hormone receptors
Source: Nat Commun. 2024 Jun 18;15:5201. doi: 10.1038/s41467-024-49493-6 (PMC11189487; doi:10.1038/s41467-024-49493-6)

COMPOUND INFORMATION

BMS753

**CAS Registry No.:**	215307-86-1
**Formal Name:**	4-(1,1,3,3-tetramethyl-2-oxo-2,3-dihydro-1H-indene-5-carboxamido)benzoic acid
**EUbOPEN ID:**	EUB0000565a
**Molecular Formula:**	$C_{21}H_{21}NO_4$
**Molecular Weight:**	351.40 g/mol
**Smiles:**	CC1(C2=C(C=C(C=C2)C(=O)NC3=CC=C(C=C3)C(=O)O)C(C1=O)(C)C)C
**Recommended concentration:**	1 µM

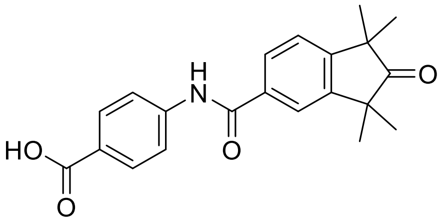

Biological activity

		Type	IC_{50}/EC_{50} [µM]	Reference
Main target:	NR1B1 (RARα)	Agonist	0.11	inhouse
Off-targets:				

COMPOUND INFORMATION

Identity

^{1}H NMR

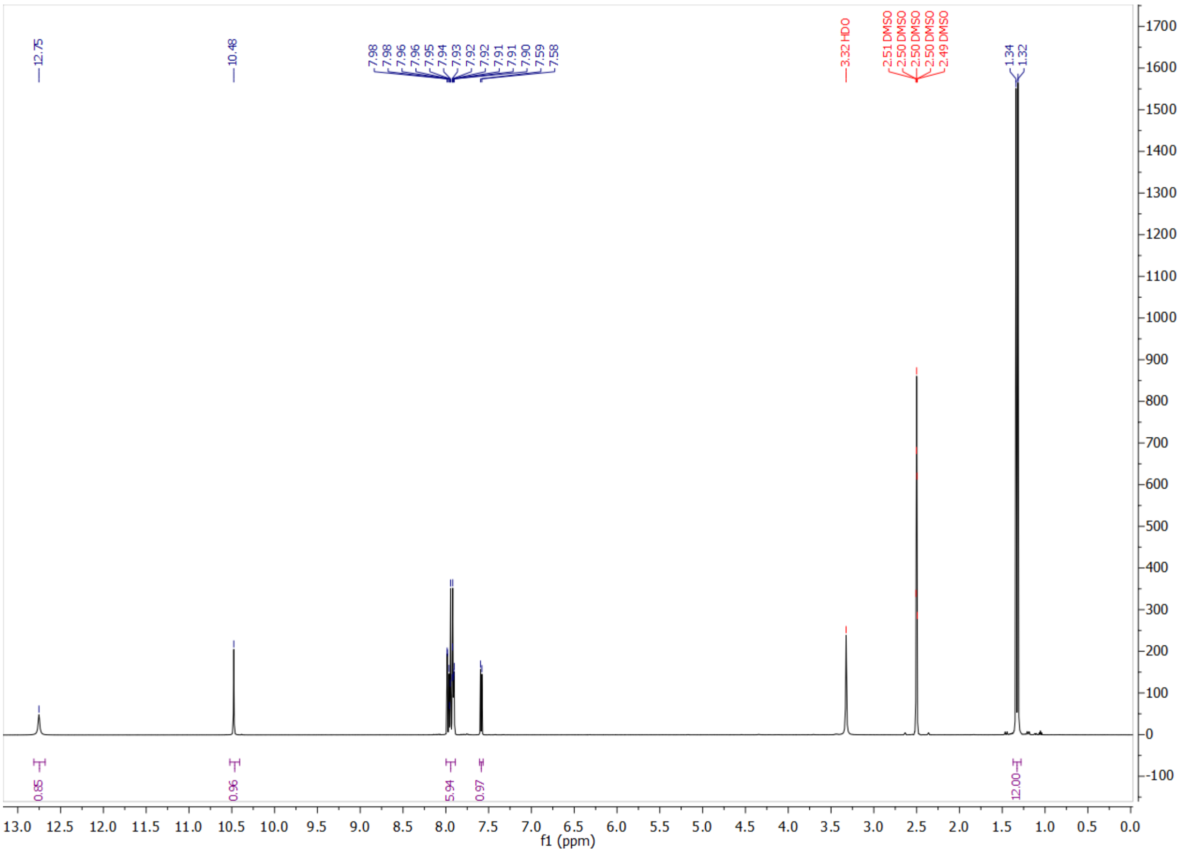

^{13}C NMR

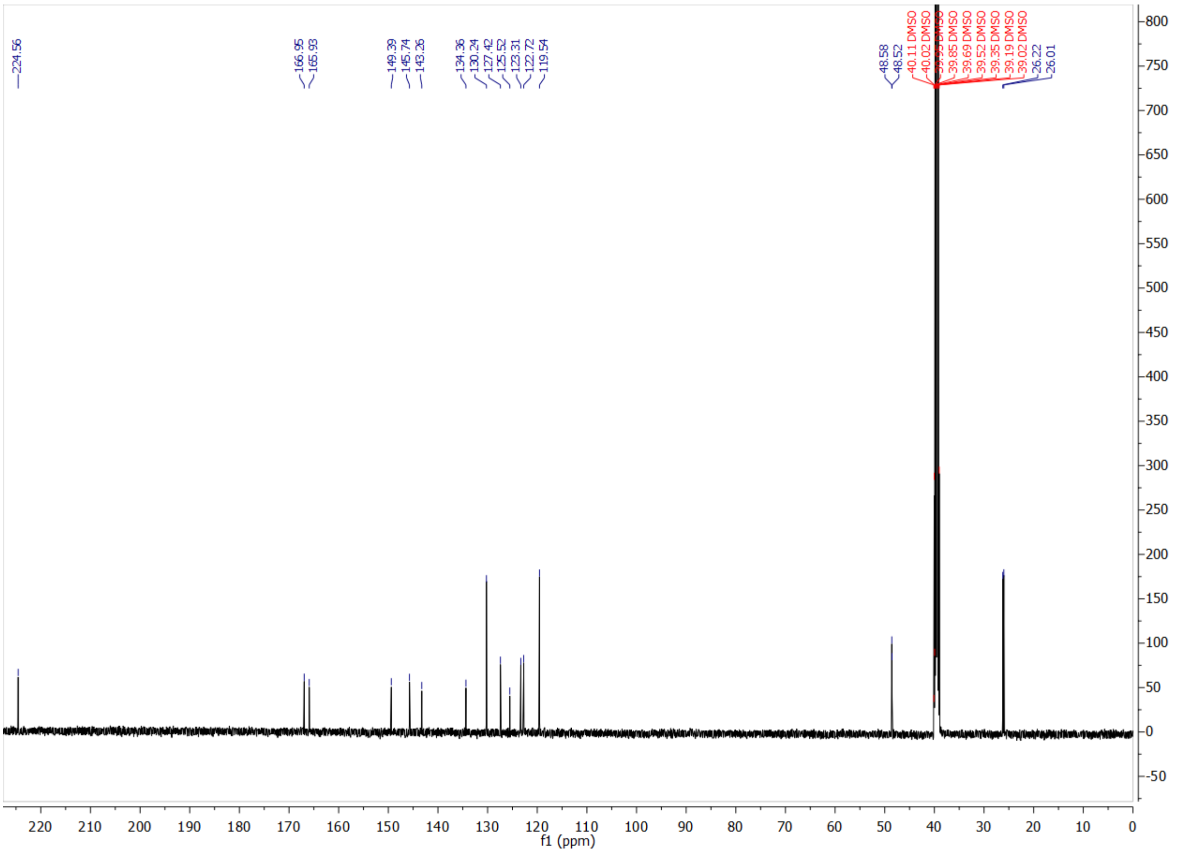

COMPOUND INFORMATION

Purity

```
Data File W:\analyti...OPEN\CGC_ECHO1-3_FirstPass 2021-03-20 13-21-54\004-D2B-A12-BMS753.D
Sample Name: BMS753

    =====================================================================
    Acq. Operator   : SYSTEM                          Seq. Line :    4
    Sample Operator : SYSTEM
    Acq. Instrument : LCMS test                        Location :    D2B-A12
    Injection Date  : 3/20/2021 1:58:50 PM                  Inj :    1
                                                    Inj Volume : Inj prog
    Sequence File   : W:\analytical_LCMS_DATA\EUbOPEN\CGC_ECHO1-3_FirstPass 2021-03-20 13-21-54
                      \CGC_ECHO1-3_FirstPass.S
    Method          : W:\analytical_LCMS_DATA\EUbOPEN\CGC_ECHO1-3_FirstPass 2021-03-20 13-21-54
                      \CGL_FIRSTPASS_GENERALMETHOD_VIAL3+4_20210319.M (Sequence Method)
    Last changed    : 3/19/2021 5:35:24 PM by SYSTEM
    Method Info     : CGL wellplate, 0.5 uL of 10 mM DMSO, general method
```

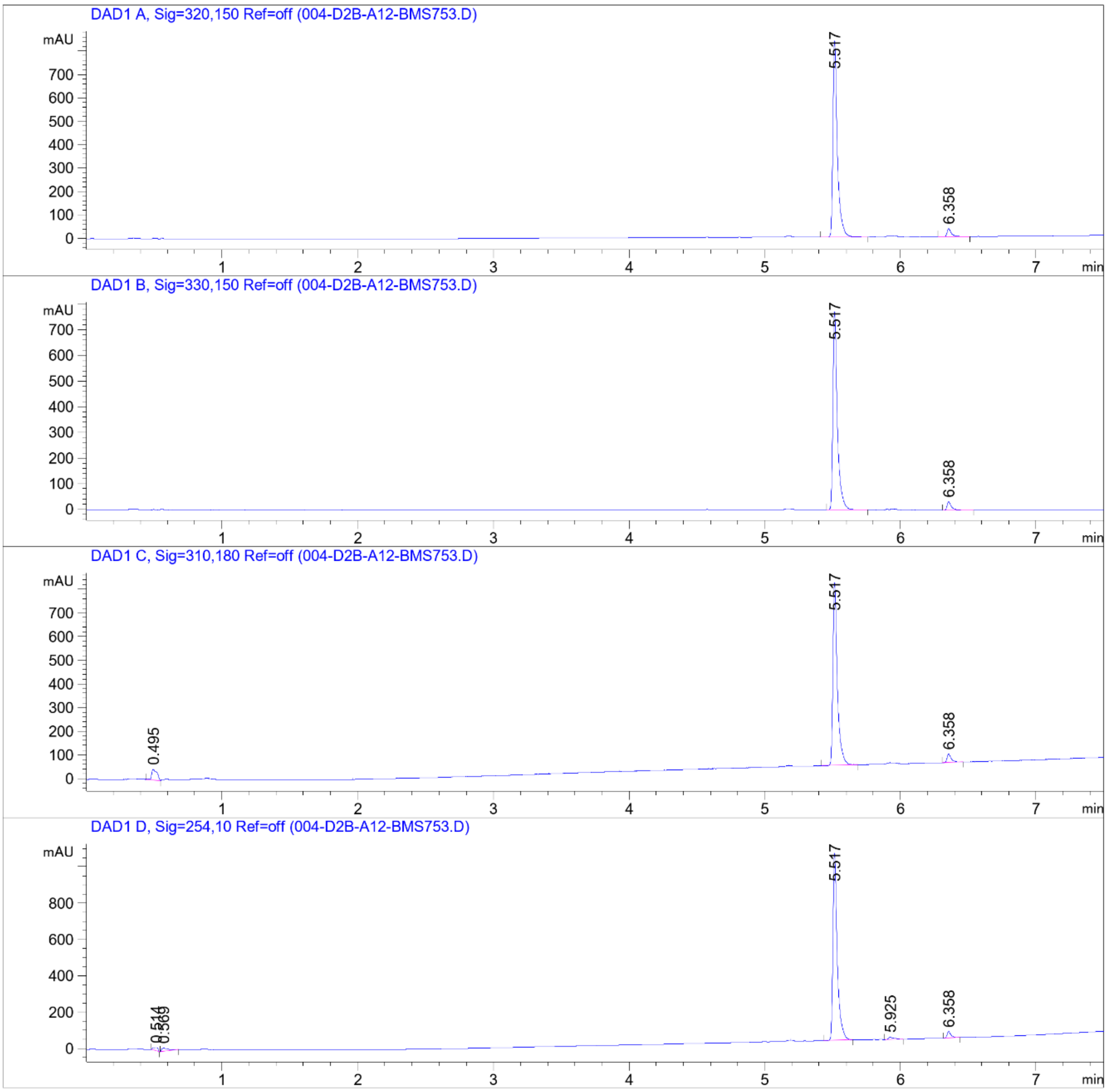

COMPOUND INFORMATION

```
Data File W:\analyti...OPEN\CGC_ECHO1-3_FirstPass 2021-03-20 13-21-54\004-D2B-A12-BMS753.D
Sample Name: BMS753
```

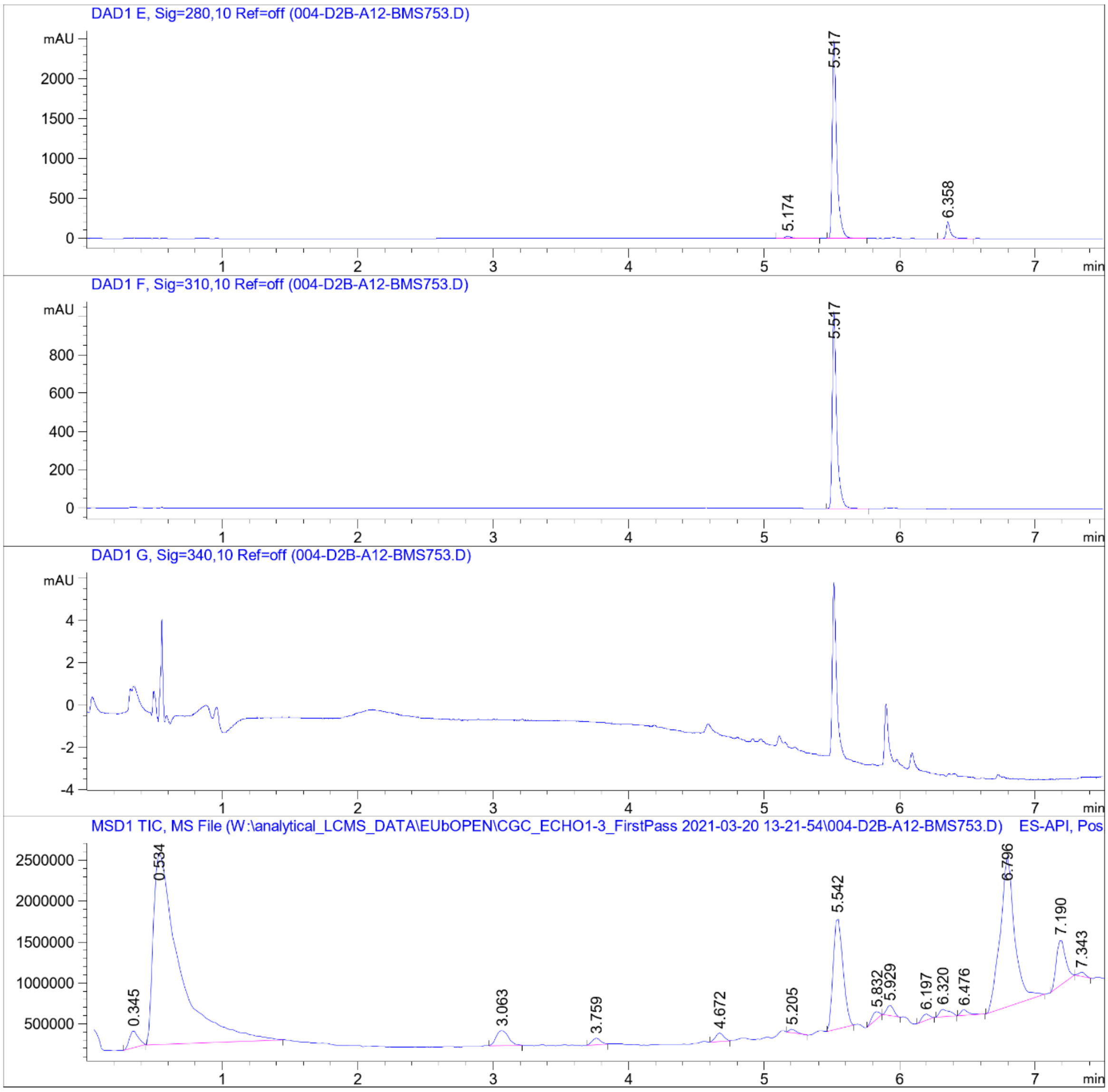

COMPOUND INFORMATION

Data File W:\analyti...OPEN\CGC_ECHO1-3_FirstPass 2021-03-20 13-21-54\004-D2B-A12-BMS753.D
Sample Name: BMS753

```
MS Signal: MSD1 TIC, MS File,  ES-API, Pos, Scan, Frag: 70, "POS Scan"
    Spectra from peak tops.
    Noise Cutoff: 1000 counts.
    Reportable Ion Abundance: > 50%.
LC Signal: DAD1 A, Sig=320,150 Ref=off
    Peak matching window: 0.1  min
```

Retention Time (LC)	LC Area	Retention Time (MS)	MS Area	Mol. Weight or Ion
-	-	0.345	938060	158.00 I
-	-	0.534	31501578	157.00 I
-	-	3.063	1183681	217.10 I
-	-	3.759	363428	274.20 I
-	-	4.672	435477	326.30 I
-	-	5.205	153765	316.20 I
				298.20 I
				199.90 I
				158.90 I
				111.10 I
				102.10 I
5.517	1895	5.542	6701950	352.10 I
-	-	5.832	353985	296.20 I
-	-	5.929	457770	296.20 I
				294.20 I
				280.30 I
-	-	6.197	303301	280.20 I
				228.20 I
6.358	76	6.320	476307	254.20 I
-	-	6.476	301237	280.20 I
-	-	6.796	13585264	282.30 I
-	-	7.190	2593089	284.20 I
				282.20 I
-	-	7.343	184609	400.30 I
				282.20 I

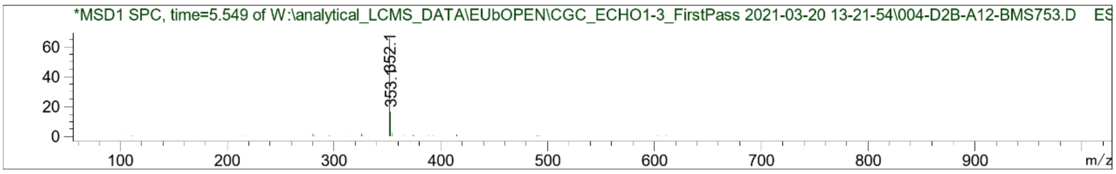

COMPOUND INFORMATION

Biological activity

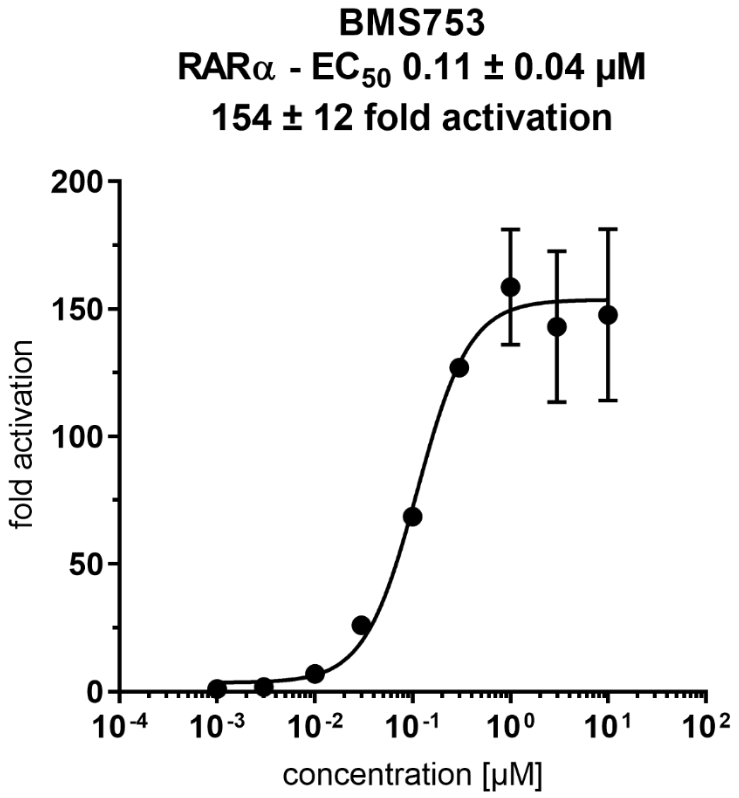

Supplement: Supplementary file 4 — Supplementary Data 1 [file 41467_2024_49493_MOESM4_ESM.zip › BMS753.pdf]
