# Supplementary material for: Chemogenomics for NR1 nuclear hormone receptors
Source: Nat Commun. 2024 Jun 18;15:5201. doi: 10.1038/s41467-024-49493-6 (PMC11189487; doi:10.1038/s41467-024-49493-6)

COMPOUND INFORMATION

BMS779788

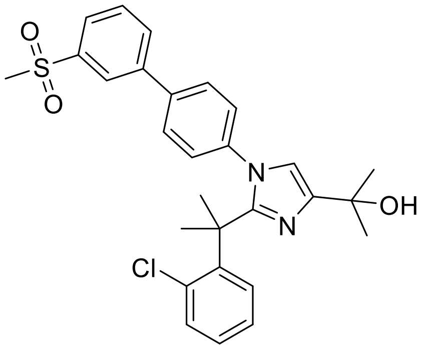

**CAS Registry No.:**	918348-67-1
**Formal Name:**	2-(2-(2-(2-chlorophenyl)propan-2-yl)-1-(3'-(methylsulfonyl)-[1,1'-biphenyl]-4-yl)-1H-imidazol-4-yl)propan-2-ol
**EUBOPEN ID:**	EUB0001166a
**Molecular Formula:**	$C_{28}H_{29}ClN_2O_3S$
**Molecular Weight:**	509.06 g/mol
**Smiles:**	CS(C1=CC(C2=CC=C(C=C2)N3C(C(C)(C4=CC=CC=C4Cl)C)=NC(C(C)(C)O)=C3)=CC=C1)(=O)=O
**Recommended concentration:**	1 µM

Biological activity

		Type	IC_{50}/EC_{50} [µM]	Reference
Main NR target:	NR1H3 (LXRα)	Agonist	0.2	https://doi.org/10.1016/j.bmcl.2014.11.029, https://doi.org/10.1021/acsmedchemlett.6b00234
	NR1H2 (LXRβ)	Agonist	0.3	
NR off-target:				

COMPOUND INFORMATION

Identity

^{1}H NMR

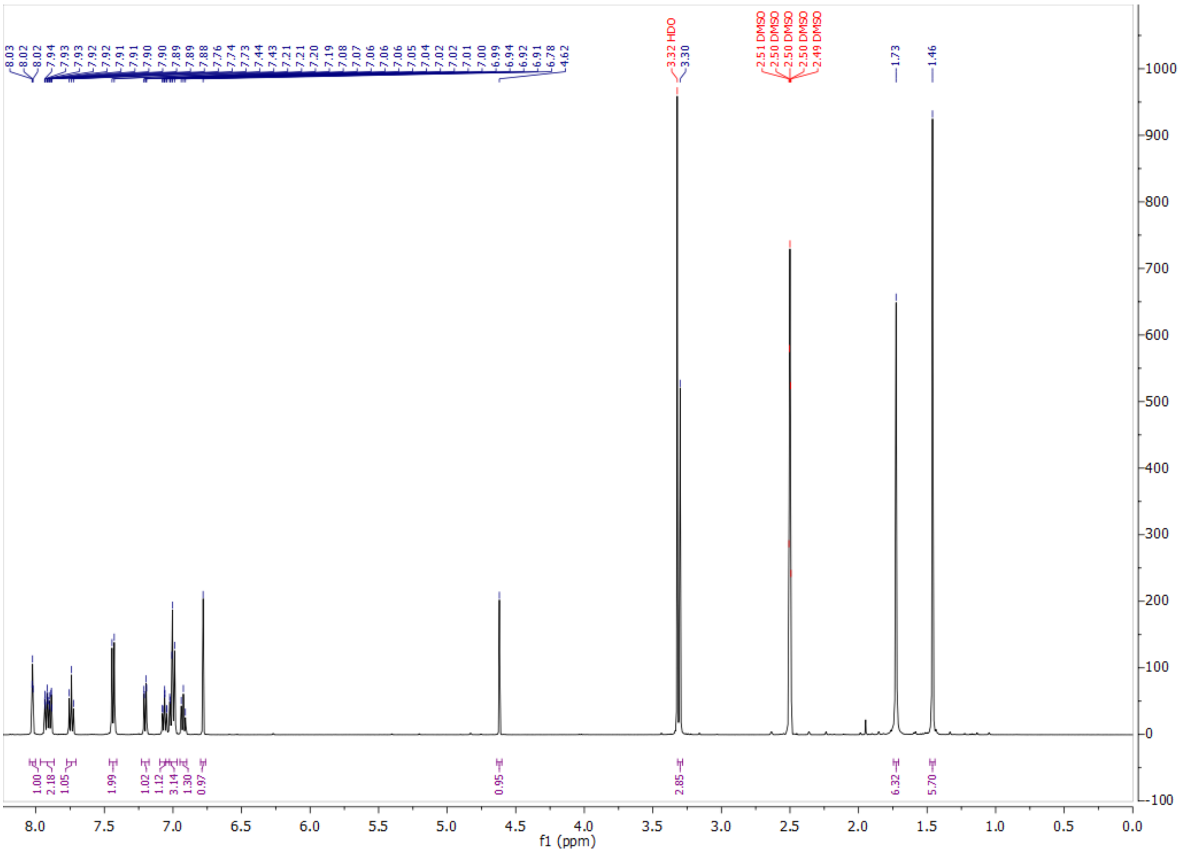

^{13}C NMR

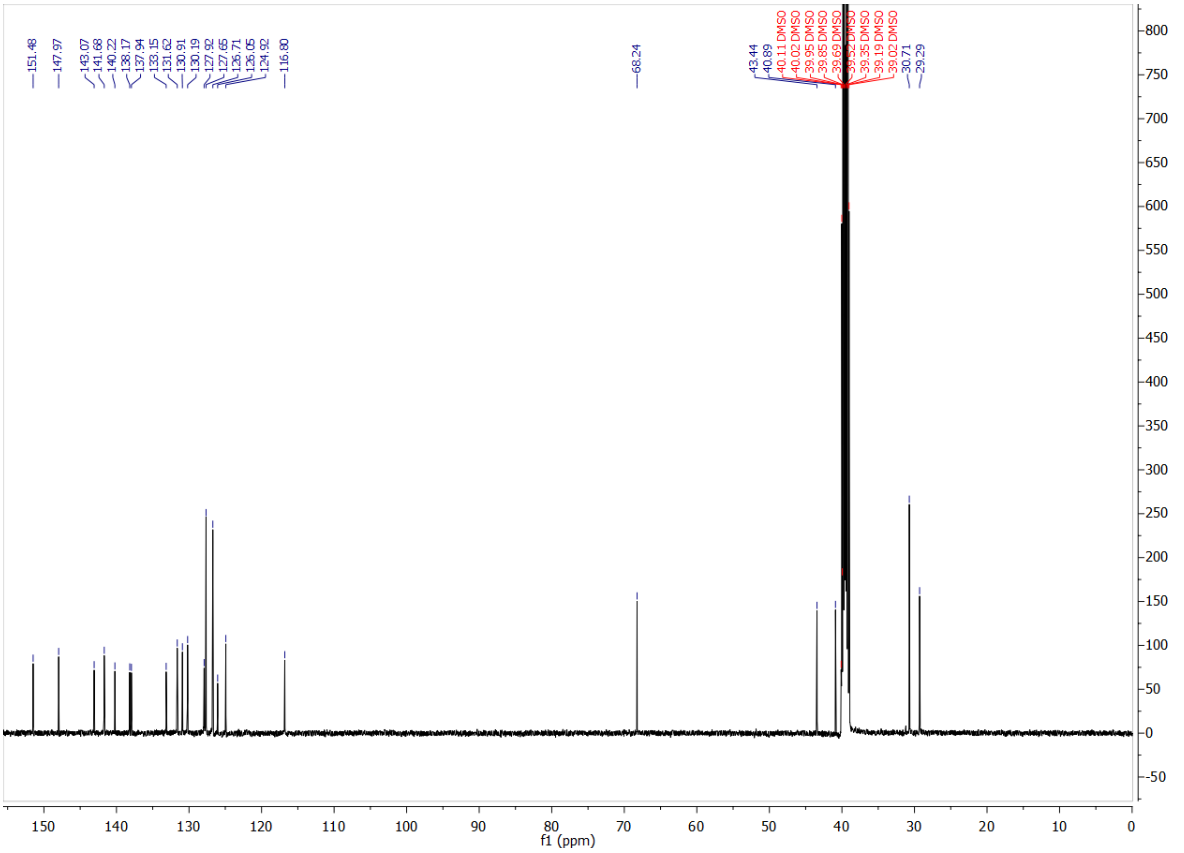

COMPOUND INFORMATION

Purity

```
Data File W:\analyti...\CGC_wave3_1_SecondPass 2023-01-19 20-16-18\031-D2F-H1-BMS 779788.D
Sample Name: BMS 779788

    =====================================================================
    Acq. Operator   : SYSTEM                           Seq. Line :  31
    Sample Operator : SYSTEM
    Acq. Instrument : LCMS test                         Location :  D2F-H1
    Injection Date  : 1/20/2023 1:56:39 AM                   Inj :  1
                                                   Inj Volume : Inj prog
    Sequence File   : W:\analytical_LCMS_DATA\EUbOPEN\CGC_wave3_1_SecondPass 2023-01-19 20-16-18
                      \CGC_wave3_1_SecondPass.S
    Method          : W:\analytical_LCMS_DATA\EUbOPEN\CGC_wave3_1_SecondPass 2023-01-19 20-16-18
                      \CGL_SECONDPASS_NONPOLCOMP_VIAL2+4_20210323.M (Sequence Method)
    Last changed    : 7/18/2022 10:07:35 AM by SYSTEM
    Method Info     : CGL wellplate, 0.5 uL of 10 mM DMSO. Dilution with MeCN only (9+9 uL)
```

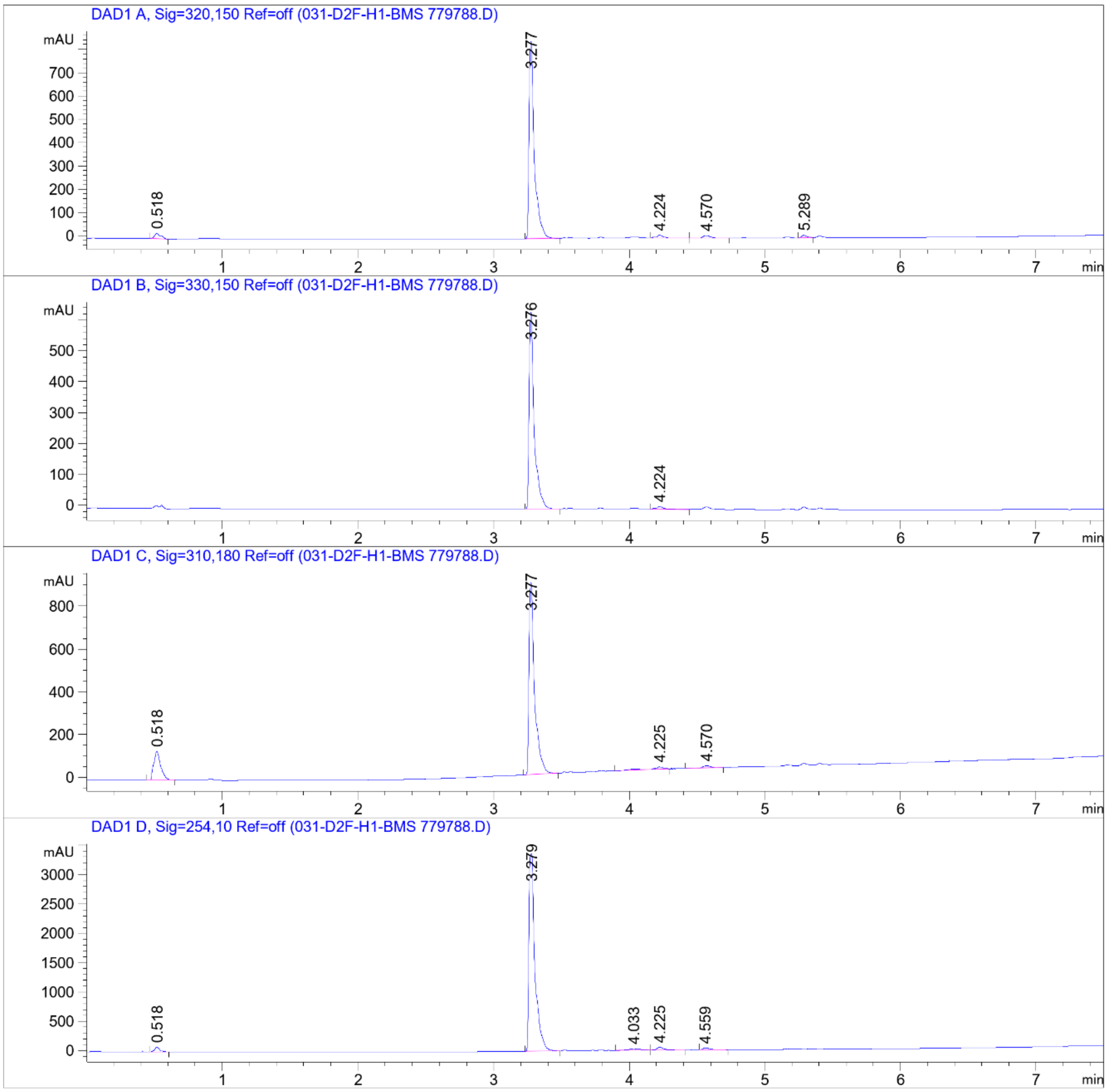

COMPOUND INFORMATION

```
Data File W:\analyti...\CGC_wave3_1_SecondPass 2023-01-19 20-16-18\031-D2F-H1-BMS 779788.D
Sample Name: BMS 779788
```

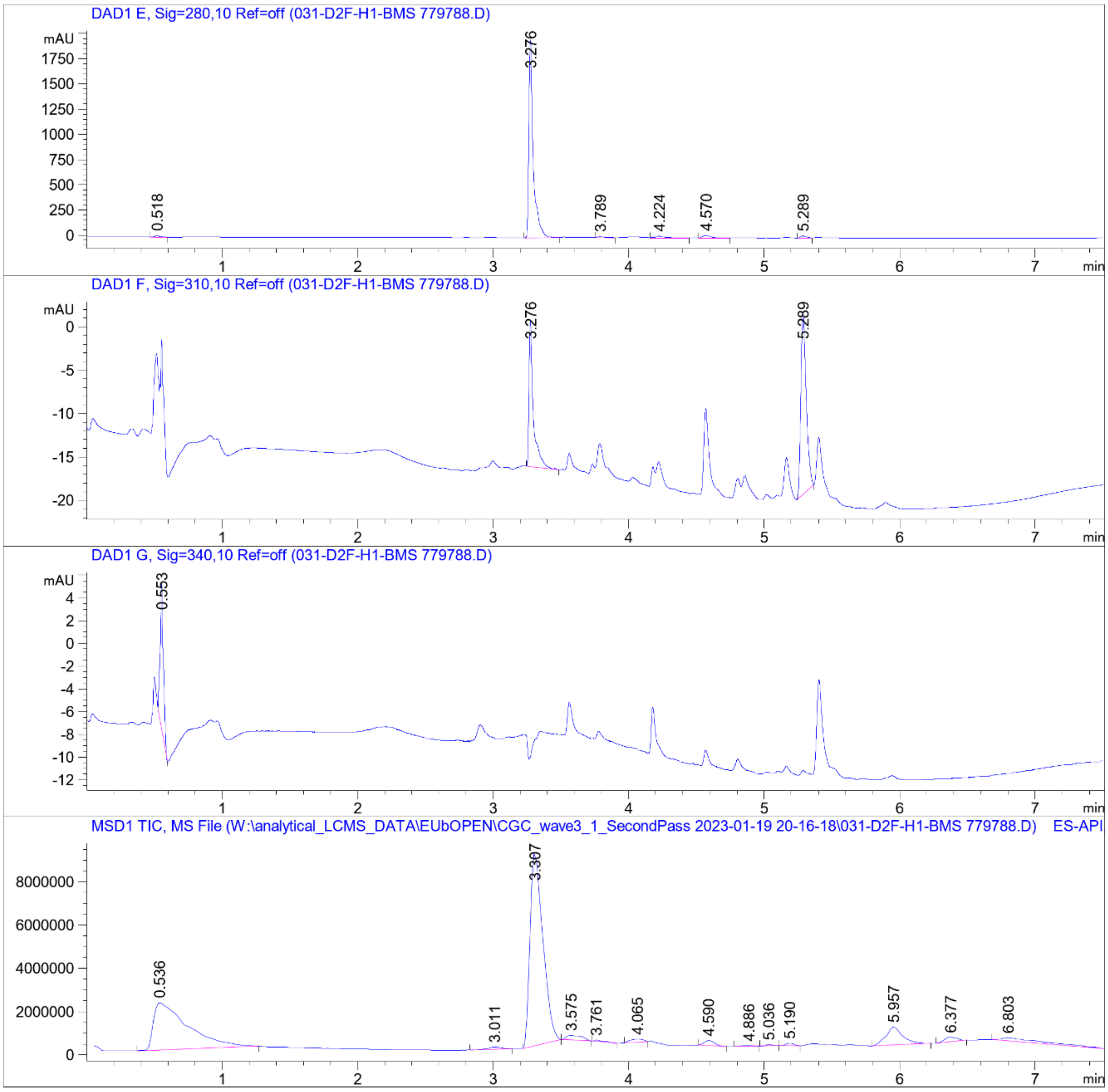

COMPOUND INFORMATION

Data File W:\analyti...\CGC_wave3_1_SecondPass 2023-01-19 20-16-18\031-D2F-H1-BMS 779788.D
Sample Name: BMS 779788

MS Signal: MSD1 TIC, MS File, ES-API, Pos, Scan, Frag: 70, "POS Scan"
Spectra from peak tops.
Noise Cutoff: 1000 counts.
Reportable Ion Abundance: > 50%.
LC Signal: DAD1 A, Sig=320,150 Ref=off
Peak matching window: 0.1 min

Retention Time (LC)	LC Area	Retention Time (MS)	MS Area	Mol. Weight or Ion
0.518	77	0.536	38466428	157.00 I
-	-	3.011	755706	239.00 I
				217.00 I
3.277	2305	3.307	58446176	509.20 I
-	-	3.575	1689027	549.20 I
-	-	3.761	288427	558.00 I
				510.30 I
				491.10 I
				258.10 I
				170.80 I
-	-	4.065	932863	502.00 I
				501.30 I
4.224	48	-	-	
4.570	49	4.590	1154513	493.10 I
-	-	4.886	235594	523.10 I
				510.30 I
				280.20 I
				137.10 I
-	-	5.036	267537	318.20 I
				296.20 I
-	-	5.190	486618	507.10 I
5.289	28	-	-	
-	-	5.957	7208726	282.20 I
-	-	6.377	1274694	400.30 I
				284.20 I
				282.20 I
				137.10 I
-	-	6.803	3892052	282.20 I
				137.10 I

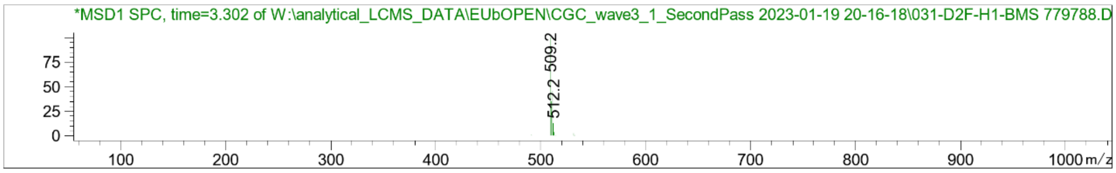

Supplement: Supplementary file 4 — Supplementary Data 1 [file 41467_2024_49493_MOESM4_ESM.zip › BMS779788.pdf]
