# Supplementary material for: Chemogenomics for NR1 nuclear hormone receptors
Source: Nat Commun. 2024 Jun 18;15:5201. doi: 10.1038/s41467-024-49493-6 (PMC11189487; doi:10.1038/s41467-024-49493-6)

# **COMPOUND** INFORMATION

# Cilofexor

| | |
|---|---|
| **CAS Registry No.:** | 1418274-28-8 |
| **Formal Name:** | 2-(3-(2-chloro-4-((5-cyclopropyl-3-(2,6-dichlorophenyl)isoxazol-4-yl)methoxy)phenyl)-3-hydroxyazetidin-1-yl)isonicotinic acid |
| **EUBOPEN ID:** | EUB0001168a |
| **Molecular Formula:** | $C_{28}H_{22}Cl_3N_3O_5$ |
| **Molecular Weight:** | 586.85 g/mol |
| **Smiles:** | OC(C1=CC=NC(N2CC(O)(C2)C3=C(C=C(C=C3)OCC4=C(ON=C4C5=C(C=CC=C5Cl)Cl)C6CC6)Cl)=C1)=O |
| **Recommended concentration:** | 1 μM |

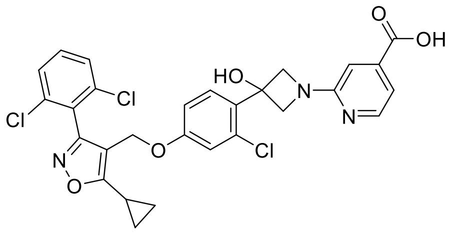


### Biological activity

| | | Type | $IC_{50}/EC_{50}$ [μM] | Reference |
|---|---|---|---|---|
| Main NR target: | NR1H4 (FXR) | Agonist | 0.04 | https://doi.org/10.1021/acs.jmedchem.9b01701 |
| NR off-target: | | | | |

# **COMPOUND** INFORMATION

## Identity

### $^{1}$H NMR

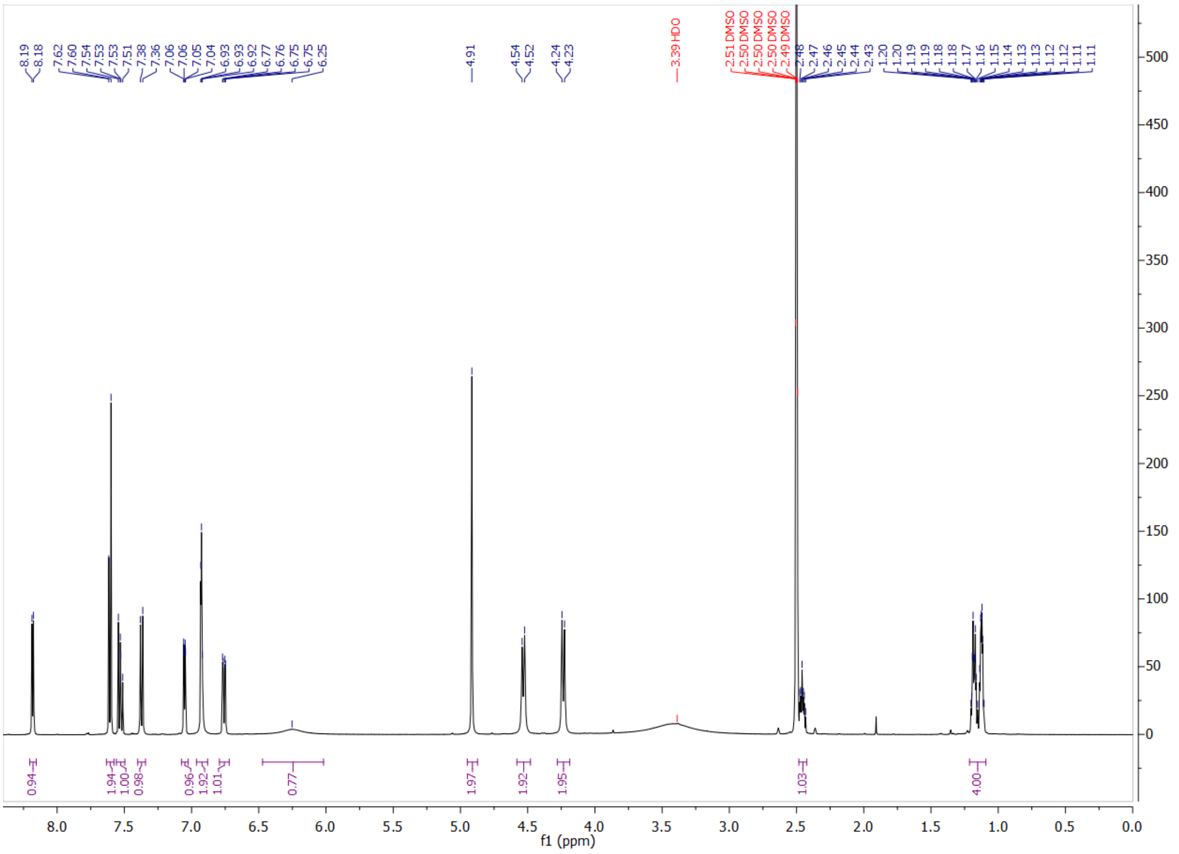

### $^{13}$C NMR

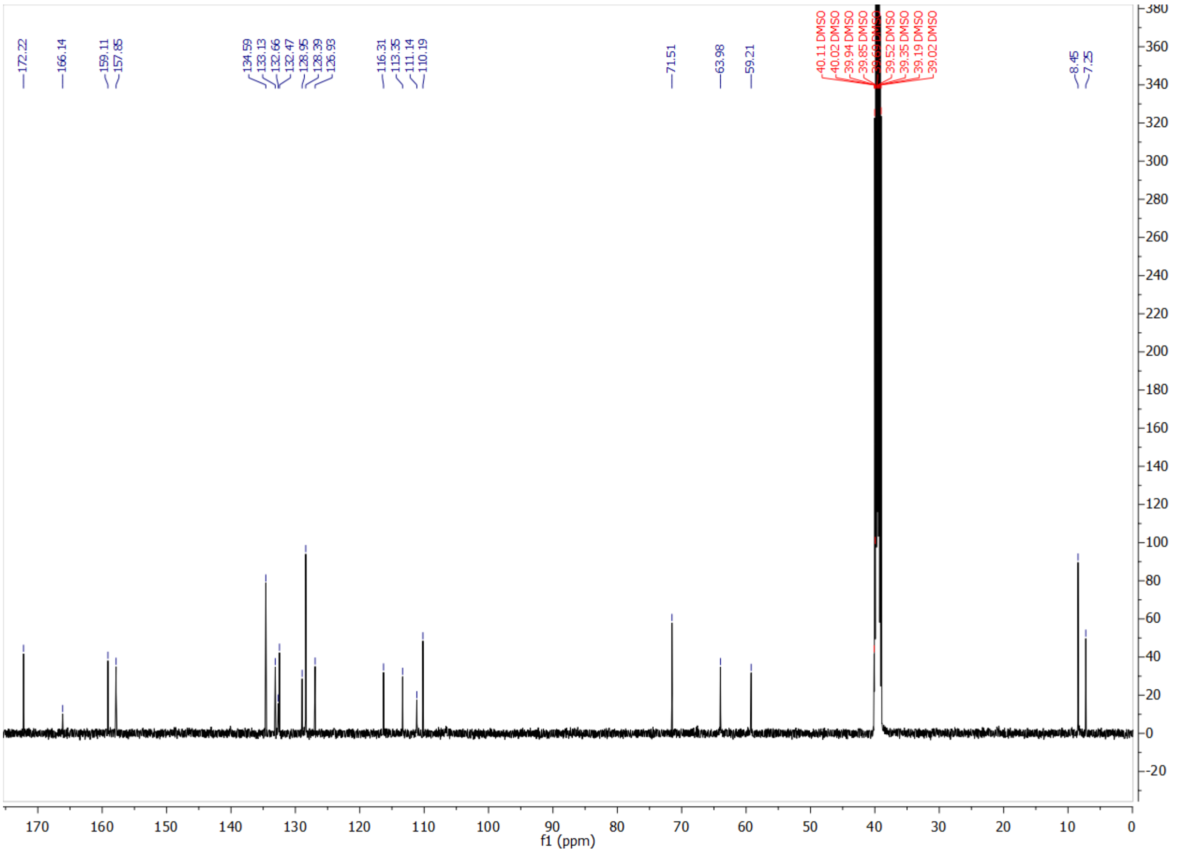

# **COMPOUND** INFORMATION

## Purity

Data File W:\analyti...N\CGC_wave3_1_SecondPass 2023-01-19 20-16-18\034-D2F-H4-Cilofexor.D
Sample Name: Cilofexor

```
=====================================================================
Acq. Operator   : SYSTEM                        Seq. Line :  34
Sample Operator : SYSTEM
Acq. Instrument : LCMS test                      Location :   D2F-H4
Injection Date  : 1/20/2023 2:30:23 AM                Inj :   1
                                               Inj Volume : Inj prog
Sequence File   : W:\analytical_LCMS_DATA\EUbOPEN\CGC_wave3_1_SecondPass 2023-01-19 20-16-18
                  \CGC_wave3_1_SecondPass.S
Method          : W:\analytical_LCMS_DATA\EUbOPEN\CGC_wave3_1_SecondPass 2023-01-19 20-16-18
                  \CGL_SECONDPASS_NONPOLCOMP_VIAL2+4_20210323.M (Sequence Method)
Last changed    : 7/18/2022 10:07:35 AM by SYSTEM
Method Info     : CGL wellplate, 0.5 uL of 10 mM DMSO. Dilution with MeCN only (9+9 uL)
```

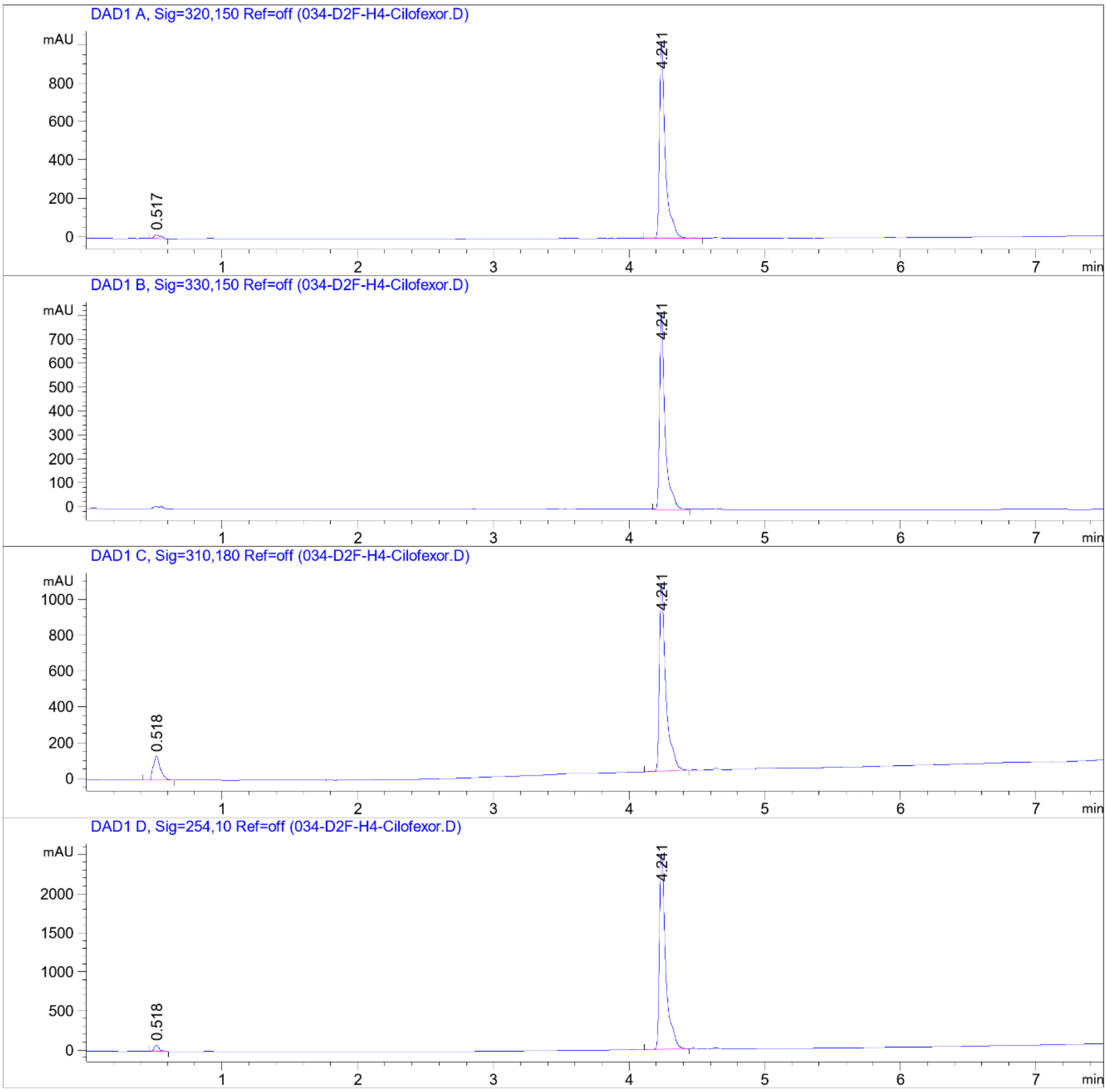

# COMPOUND INFORMATION

Data File W:\analyti...N\CGC_wave3_1_SecondPass 2023-01-19 20-16-18\034-D2F-H4-Cilofexor.D
Sample Name: Cilofexor

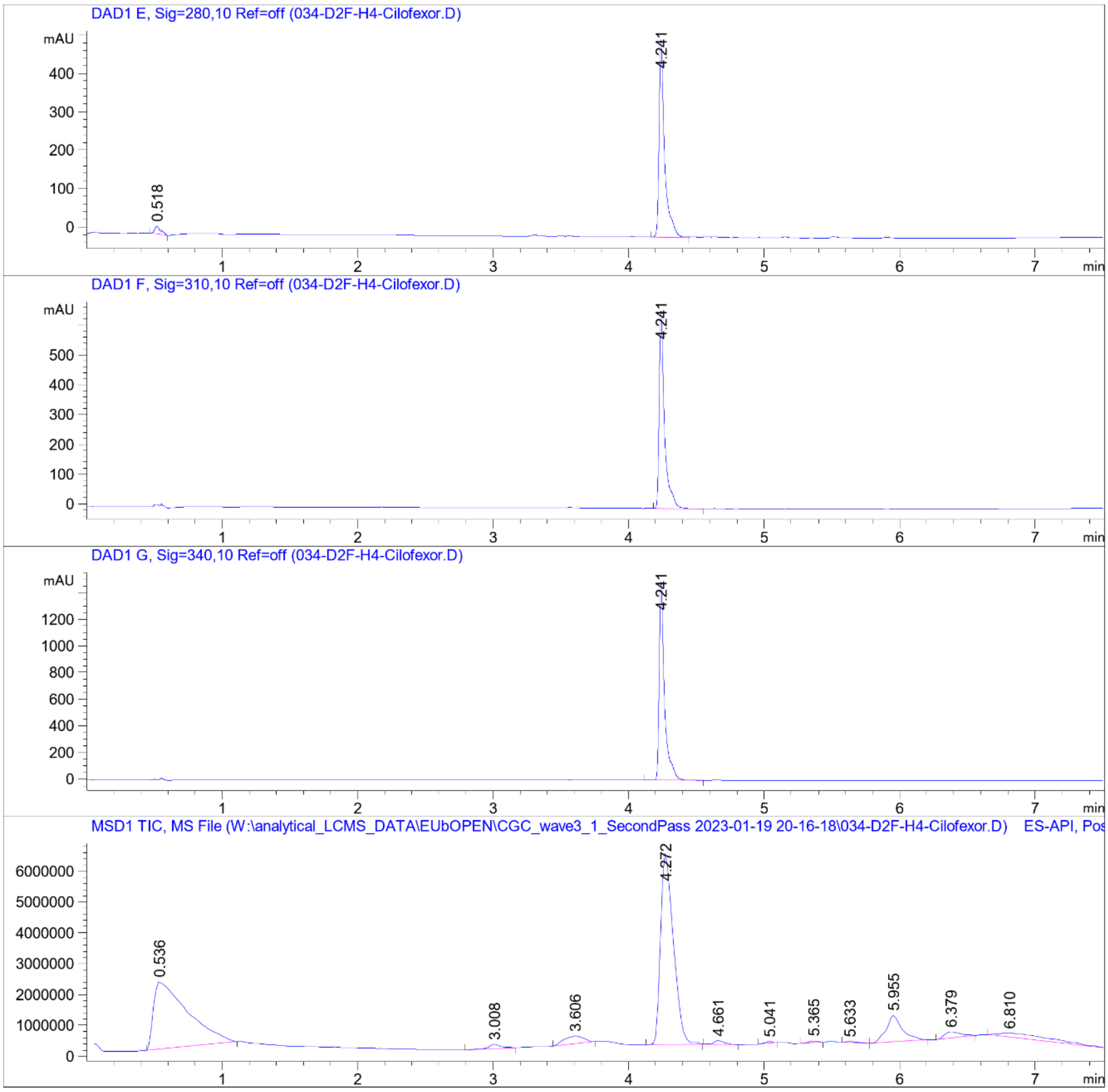

# **COMPOUND** INFORMATION

Data File W:\analyti...N\CGC_wave3_1_SecondPass 2023-01-19 20-16-18\034-D2F-H4-Cilofexor.D
Sample Name: Cilofexor

MS Signal: MSD1 TIC, MS File, ES-API, Pos, Scan, Frag: 70, "POS Scan"
Spectra from peak tops.
Noise Cutoff: 1000 counts.
Reportable Ion Abundance: > 50%.
LC Signal: DAD1 A, Sig=320,150 Ref=off
Peak matching window: 0.1 min

| Retention Time (LC) | LC Area | Retention Time (MS) | MS Area | Mol. Weight or Ion |
|---|---|---|---|---|
| 0.517 | 77 | 0.536 | 37654432 | 157.00 I |
| - | - | 3.008 | 860643 | 239.00 I |
| | | | | 217.00 I |
| - | - | 3.606 | 2444670 | 234.00 I |
| | | | | 170.80 I |
| 4.241 | 3045 | 4.272 | 40616108 | 588.10 I |
| | | | | 586.10 I |
| - | - | 4.661 | 609146 | 602.10 I |
| | | | | 600.10 I |
| - | - | 5.041 | 195912 | 318.20 I |
| | | | | 296.20 I |
| - | - | 5.365 | 301437 | 348.20 I |
| | | | | 280.20 I |
| | | | | 228.20 I |
| | | | | 137.10 I |
| - | - | 5.633 | 316641 | 507.30 I |
| | | | | 280.20 I |
| | | | | 137.10 I |
| - | - | 5.955 | 7029393 | 282.20 I |
| - | - | 6.379 | 1609329 | 400.30 I |
| | | | | 284.20 I |
| | | | | 282.20 I |
| | | | | 137.10 I |
| - | - | 6.810 | 4163593 | 282.20 I |
| | | | | 137.10 I |

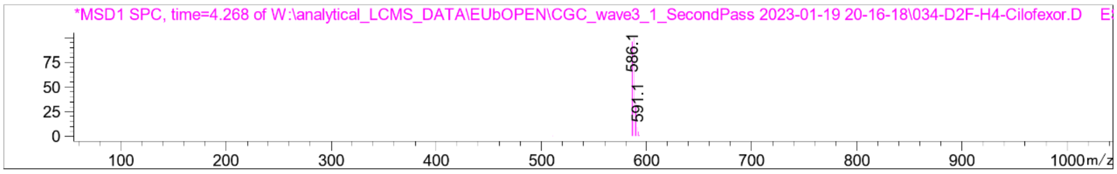

Supplement: Supplementary file 4 — Supplementary Data 1 [file 41467_2024_49493_MOESM4_ESM.zip › Cilofexor.pdf]
