# Supplementary material for: Chemogenomics for NR1 nuclear hormone receptors
Source: Nat Commun. 2024 Jun 18;15:5201. doi: 10.1038/s41467-024-49493-6 (PMC11189487; doi:10.1038/s41467-024-49493-6)

COMPOUND INFORMATION

CINPA1

**CAS Registry No.:**	102636-74-8
**Formal Name:**	Ethyl (5-(diethylglycyl)-10,11-dihydro-5H-dibenzo[b,f]azepin-3-yl)carbamate
**EUbOPEN ID:**	EUB0001163a
**Molecular Formula:**	$C_{23}H_{29}N_3O_3$
**Molecular Weight:**	395.50 g/mol
**Smiles:**	O=C(N1C2=C(CCC3=C1C=C(C=C3)NC(OCC)=O)C=CC=C2)CN(CC)CC
**Recommended concentration:**	10 µM

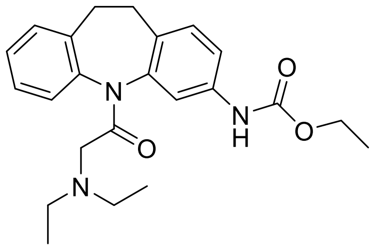

Biological activity

		Type	IC_{50}/EC_{50} [µM]	Reference
Main NR target:	NR1I3 (CAR)	Antagonist	0.69	https://doi.org/10.1016/j.ejmech.2015.12.018
NR off-target:				

COMPOUND INFORMATION

Identity

^{1}H NMR

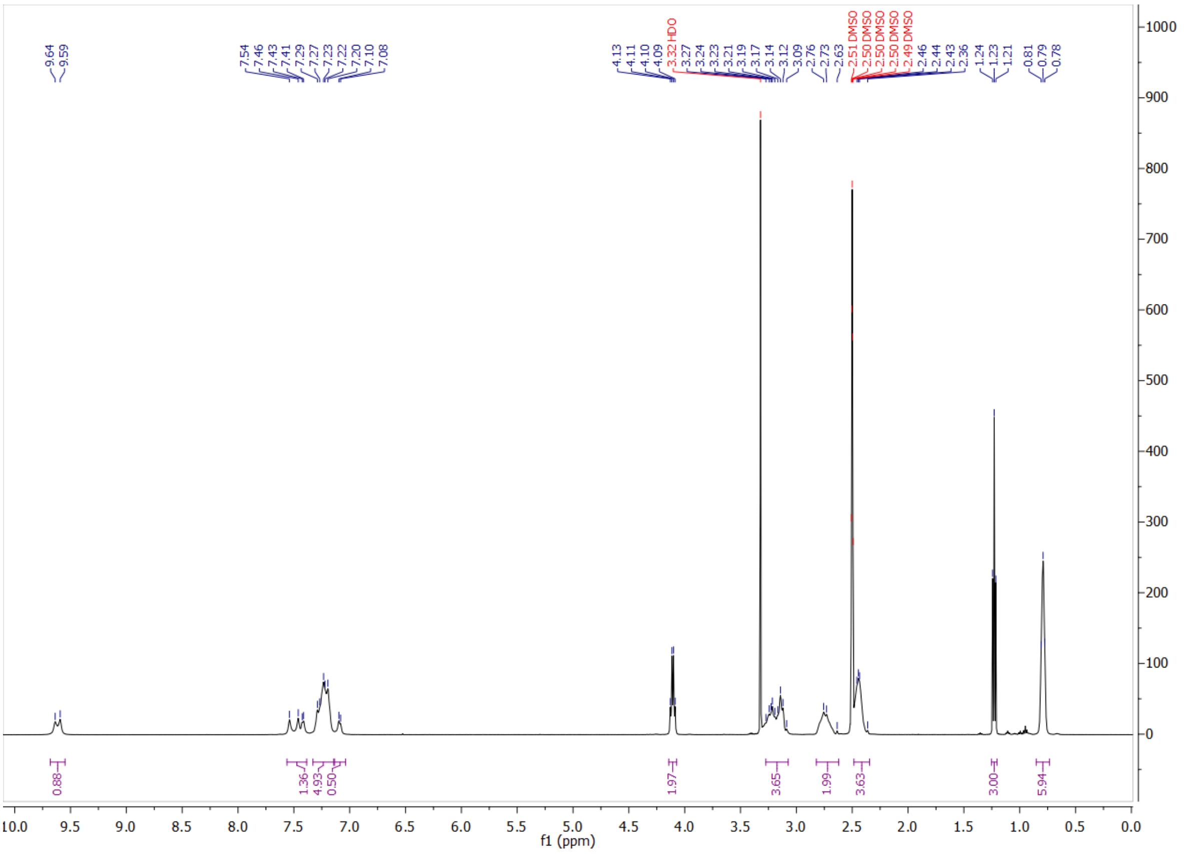

^{13}C NMR

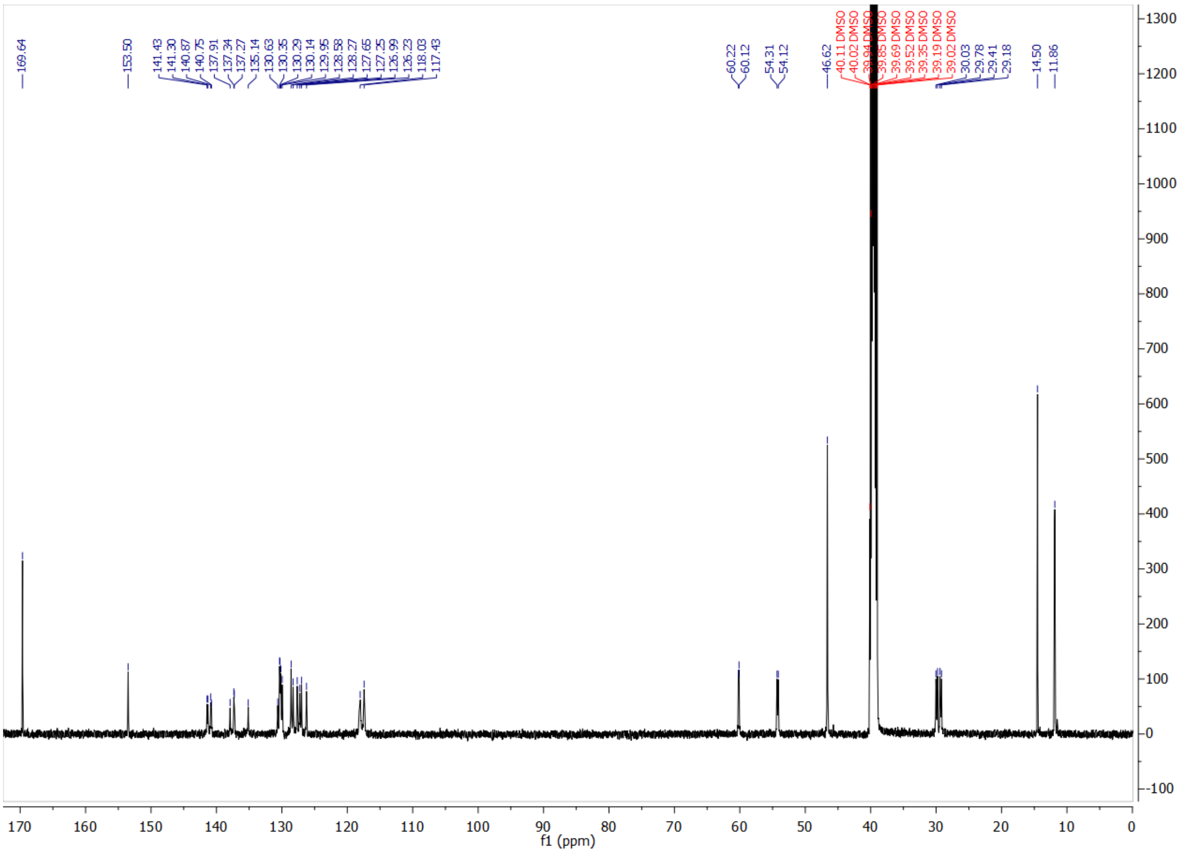

COMPOUND INFORMATION

Purity

```
Data File W:\analyti...OPEN\CGC_wave3_1_FirstPassB 2023-01-04 18-28-02\084-D2F-G9-CINPA1.D
Sample Name: CINPA1

    ======================================================================
    Acq. Operator   : SYSTEM                          Seq. Line :  84
    Sample Operator : SYSTEM
    Acq. Instrument : LCMS test                        Location :   D2F-G9
    Injection Date  : 1/5/2023 9:48:40 AM                   Inj :   1
                                                     Inj Volume : Inj prog
    Sequence File   : W:\analytical_LCMS_DATA\EUbOPEN\CGC_wave3_1_FirstPassB 2023-01-04 18-28-02
                      \CGC_wave3_1_FirstPassB.S
    Method          : W:\analytical_LCMS_DATA\EUbOPEN\CGC_wave3_1_FirstPassB 2023-01-04 18-28-02
                      \CGL_FIRSTPASS_GENERALMETHOD_VIAL1+2_20210319.M (Sequence Method)
    Last changed    : 1/25/2022 4:36:18 PM by SYSTEM
    Method Info     : CGL wellplate, 0.5 uL of 10 mM DMSO, general method
```

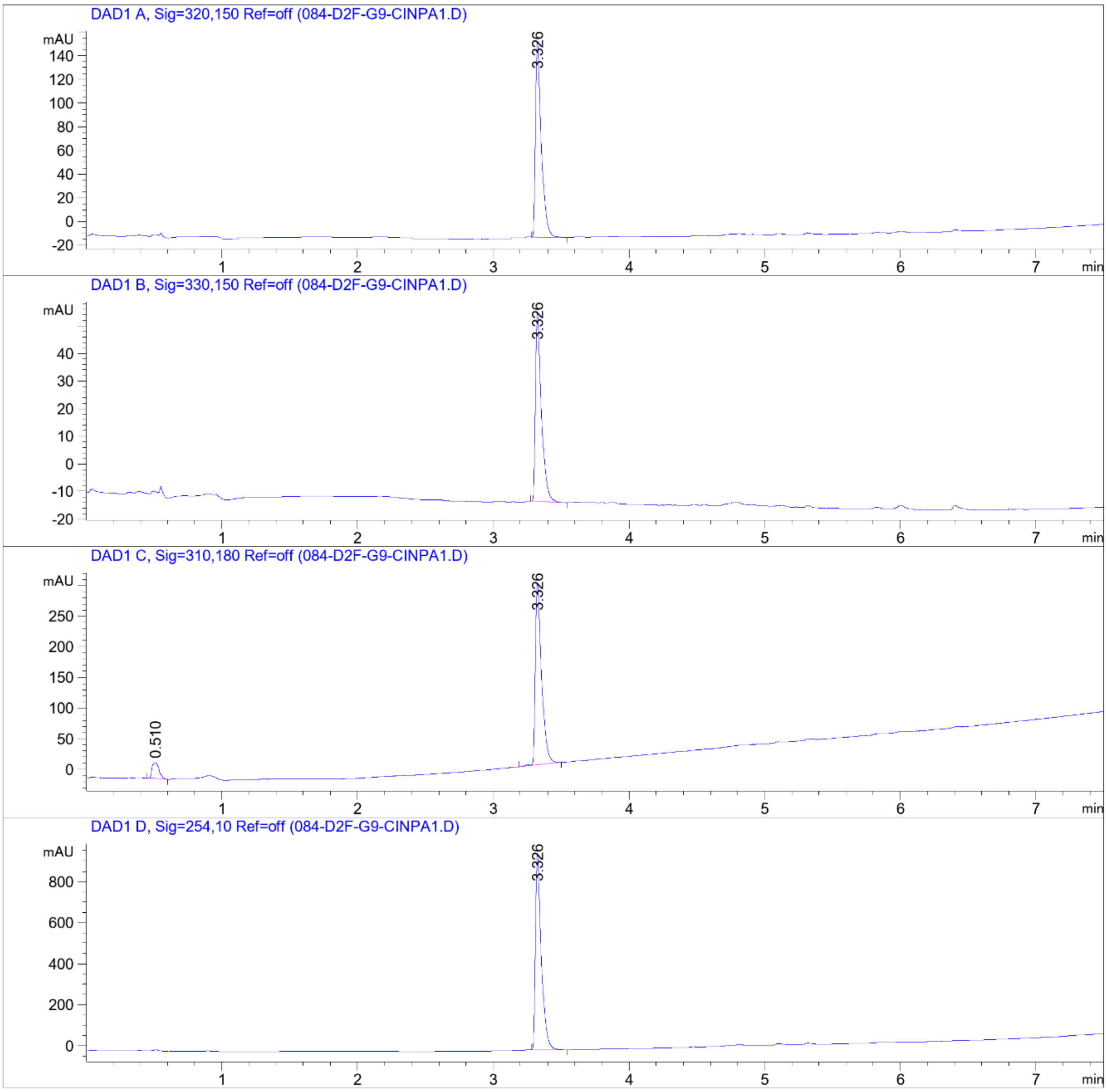

COMPOUND INFORMATION

```
Data File W:\analyti...OPEN\CGC_wave3_1_FirstPassB 2023-01-04 18-28-02\084-D2F-G9-CINPA1.D
Sample Name: CINPA1
```

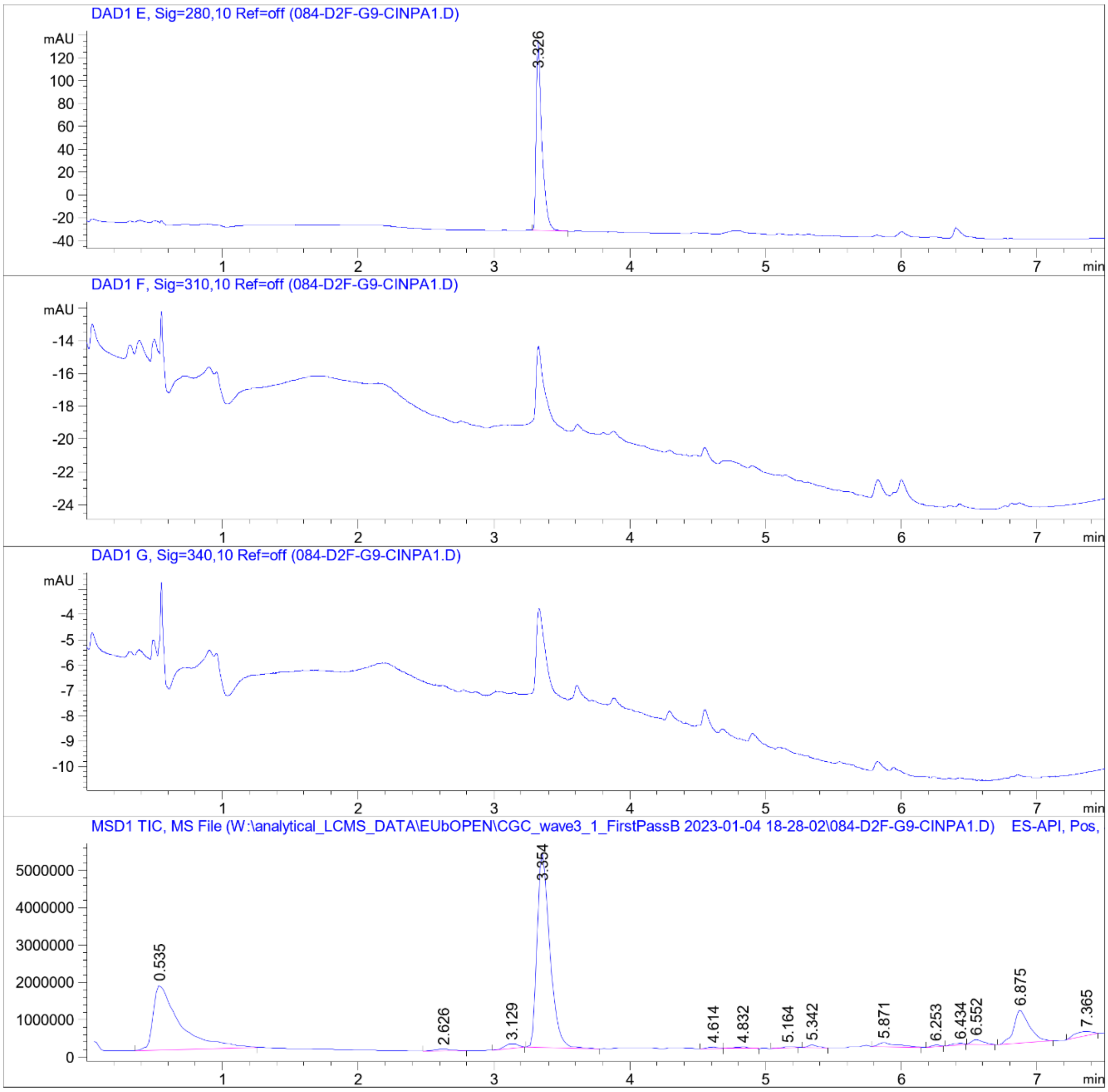

COMPOUND INFORMATION

Data File W:\analyti...OPEN\CGC_wave3_1_FirstPassB 2023-01-04 18-28-02\084-D2F-G9-CINPA1.D
Sample Name: CINPA1

MS Signal: MSD1 TIC, MS File, ES-API, Pos, Scan, Frag: 70, "POS Scan"
Spectra from peak tops.
Noise Cutoff: 1000 counts.
Reportable Ion Abundance: > 50%.
LC Signal: DAD1 A, Sig=320,150 Ref=off
Peak matching window: 0.1 min

Retention Time (LC)	LC Area	Retention Time (MS)	MS Area	Mol. Weight or Ion
-	-	0.535	23416552	157.00 I
-	-	2.626	439920	219.10 I
-	-	3.129	753895	396.20 I
				239.10 I
				217.10 I
				188.10 I
3.326	498	3.354	33809932	396.20 I
-	-	4.614	195320	510.30 I
				170.80 I
				158.20 I
				137.20 I
-	-	4.832	275218	377.10 I
				355.20 I
				170.80 I
				137.10 I
-	-	5.164	144733	510.40 I
				338.20 I
				316.20 I
				298.30 I
				170.90 I
				137.10 I
				111.10 I
				105.10 I
-	-	5.342	339033	381.00 I
				359.10 I
-	-	5.871	953999	318.20 I
				296.20 I
-	-	6.253	167474	228.10 I
				137.10 I
-	-	6.434	194943	350.30 I
				282.30 I
				254.20 I
				137.10 I
-	-	6.552	775593	507.30 I
				485.20 I
				280.20 I
-	-	6.875	6974802	282.20 I
-	-	7.365	1117944	400.30 I
				282.20 I

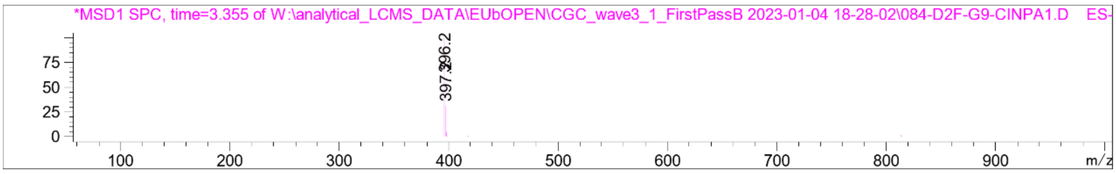

Supplement: Supplementary file 4 — Supplementary Data 1 [file 41467_2024_49493_MOESM4_ESM.zip › CINPA1.pdf]
