# Supplementary material for: Chemogenomics for NR1 nuclear hormone receptors
Source: Nat Commun. 2024 Jun 18;15:5201. doi: 10.1038/s41467-024-49493-6 (PMC11189487; doi:10.1038/s41467-024-49493-6)

COMPOUND INFORMATION

Cintirorgon

**CAS Registry No.:**	2055536-64-4
**Formal Name:**	(S)-3-(6-(3-(difluoromethoxy)-5-fluorophenyl)-4-((3-(trifluoromethyl)phenyl)sulfonyl)-3,4-dihydro-2H-benzo[b][1,4]oxazin-2-yl)-2,2-dimethylpropanoic acid
**EUBOPEN ID:**	EUB0001172
**Molecular Formula:**	$C_{27}H_{23}F_6NO_6S$
**Molecular Weight:**	603.53 g/mol
**Smiles:**	O=S(N1C2=C(O[C@H](C1)CC(C)(C(O)=O)C)C=CC(C3=CC(F)=CC(OC(F)F)=C3)=C2)(C4=CC(C(F)(F)F)=CC=C4)=O
**Recommended concentration:**	1 µM

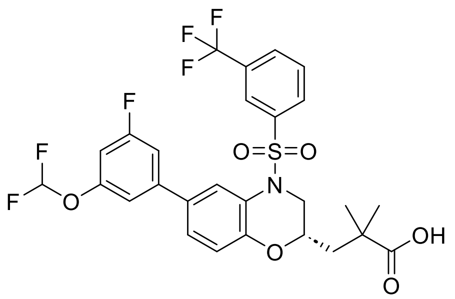

Biological activity

		Type	IC_{50}/EC_{50} [µM]	Reference
Main NR target:	NR1F3 (RORγ)	Agonist	0.021	inhouse
NR off-target:				

COMPOUND INFORMATION

Identity

^{1}H NMR

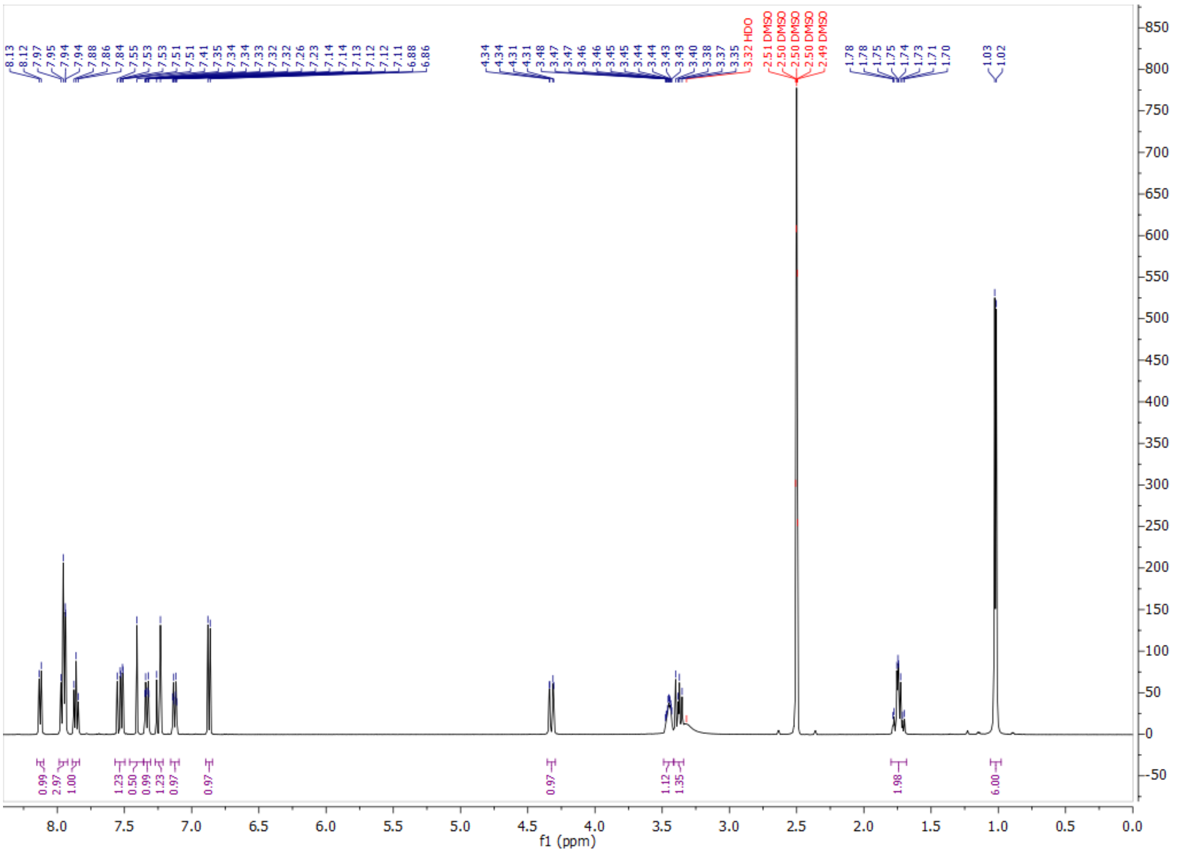

^{13}C NMR

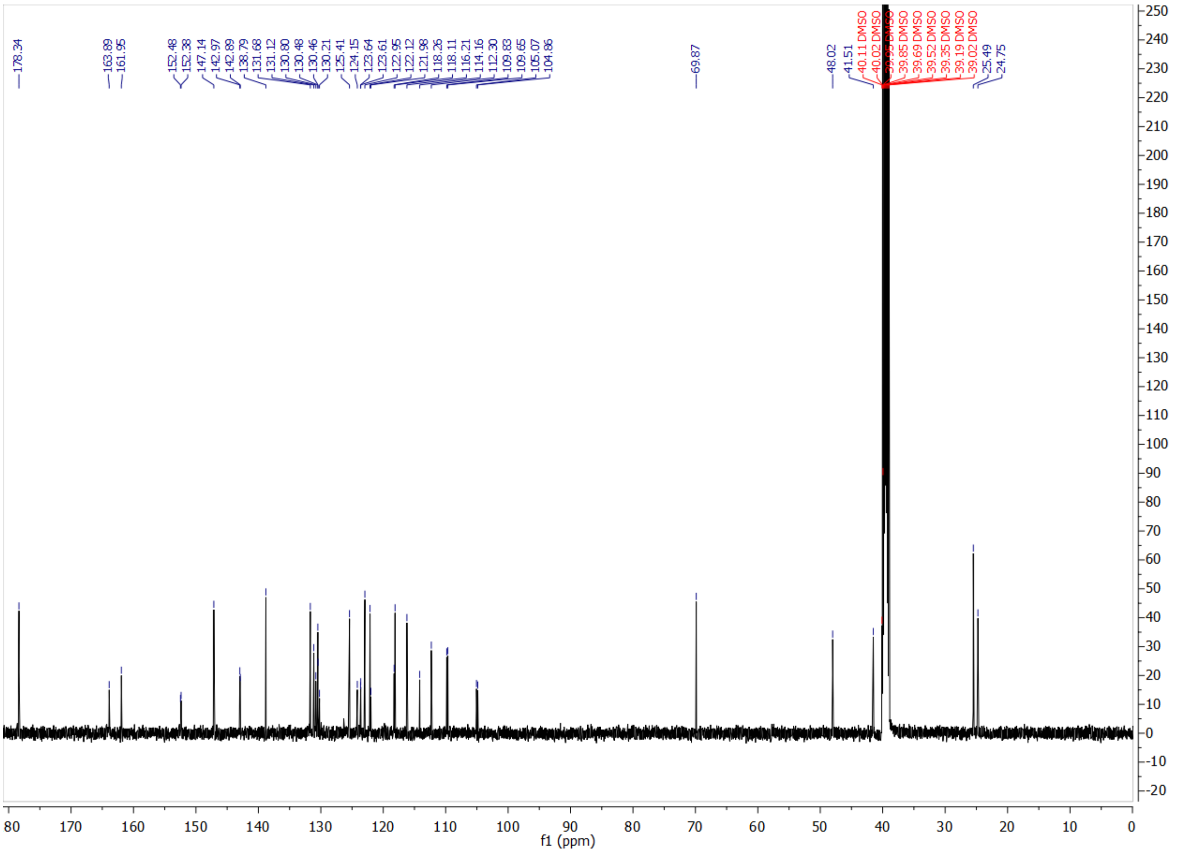

COMPOUND INFORMATION

Purity

Data File W:\analyti...CGC_wave3_1_FirstPassB 2023-01-04 18-28-02\095-D2F-H8-Cintirorgon.D
Sample Name: Cintirorgon

===

Acq. Operator : SYSTEM Seq. Line : 95
Sample Operator : SYSTEM
Acq. Instrument : LCMS test Location : D2F-H8
Injection Date : 1/5/2023 11:50:22 AM Inj : 1
Inj Volume : Inj prog
Sequence File : W:\analytical_LCMS_DATA\EUbOPEN\CGC_wave3_1_FirstPassB 2023-01-04 18-28-02\CGC_wave3_1_FirstPassB.S
Method : W:\analytical_LCMS_DATA\EUbOPEN\CGC_wave3_1_FirstPassB 2023-01-04 18-28-02\CGL_FIRSTPASS_GENERALMETHOD_VIAL1+2_20210319.M (Sequence Method)
Last changed : 1/25/2022 4:36:18 PM by SYSTEM
Method Info : CGL wellplate, 0.5 uL of 10 mM DMSO, general method

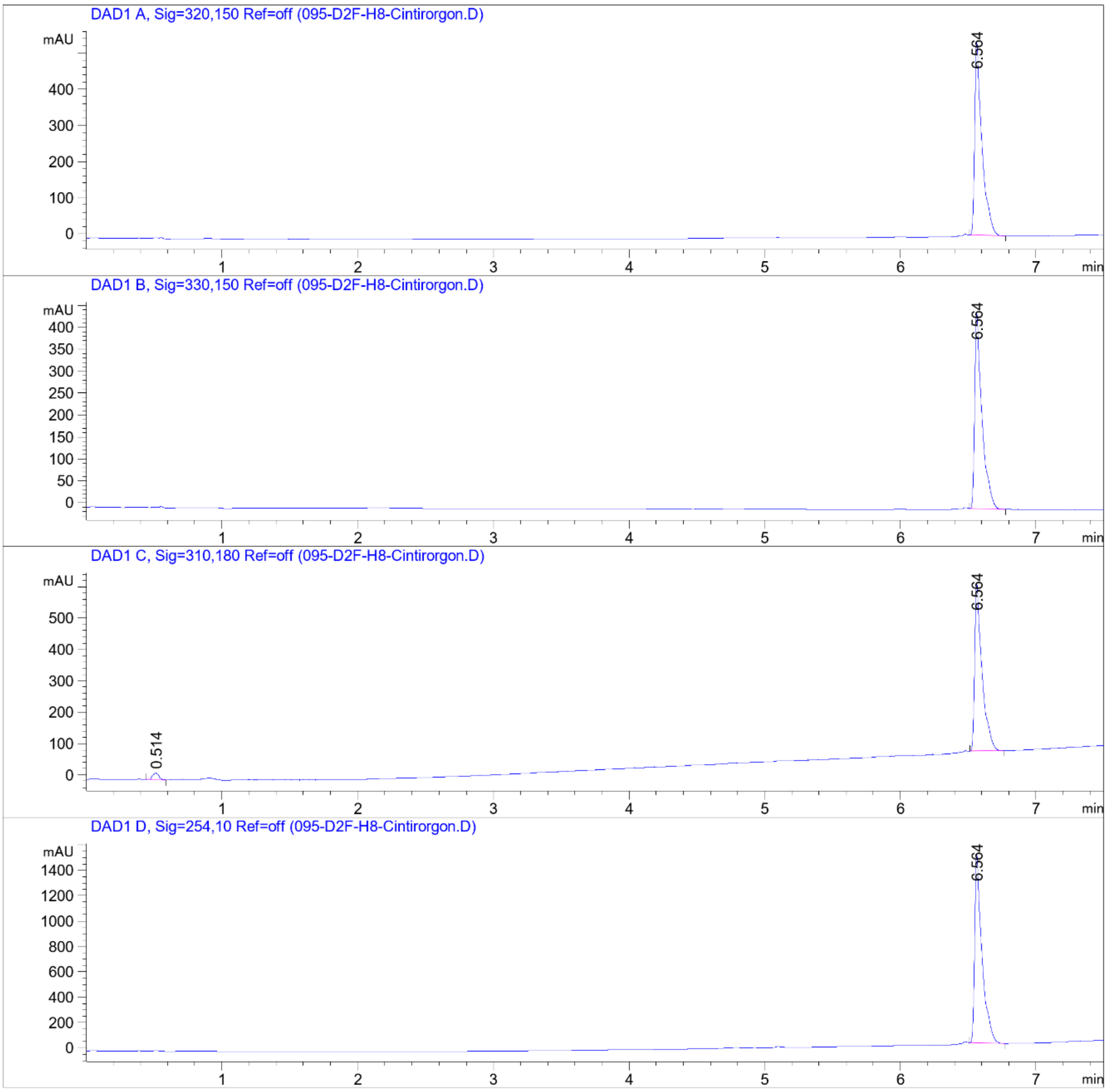

COMPOUND INFORMATION

Data File W:\analyti...CGC_wave3_1_FirstPassB 2023-01-04 18-28-02\095-D2F-H8-Cintirorgon.D
Sample Name: Cintirorgon

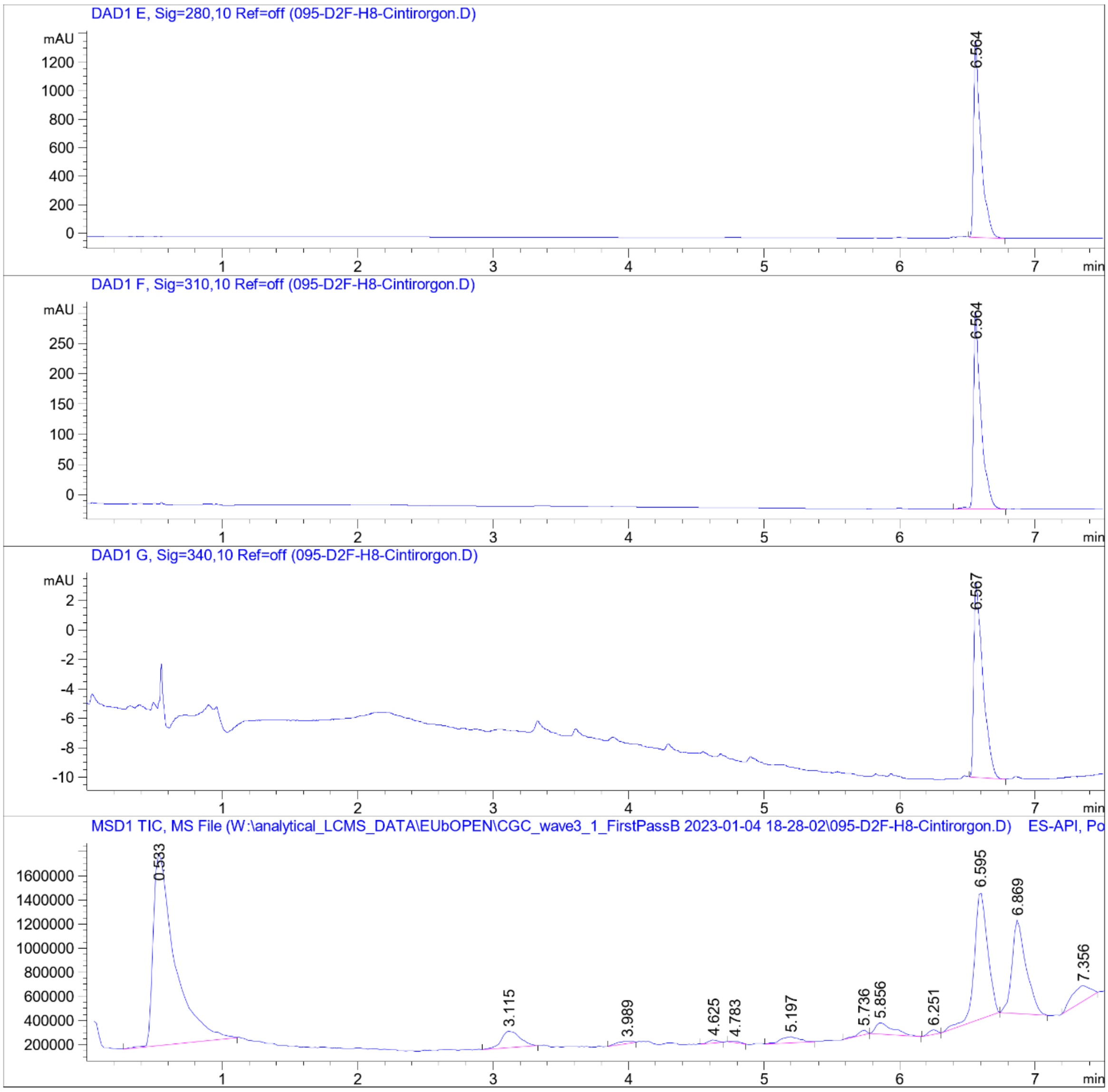

COMPOUND INFORMATION

Data File W:\analyti...CGC_wave3_1_FirstPassB 2023-01-04 18-28-02\095-D2F-H8-Cintirorgon.D
Sample Name: Cintirorgon

MS Signal: MSD1 TIC, MS File, ES-API, Pos, Scan, Frag: 70, "POS Scan"
Spectra from peak tops.
Noise Cutoff: 1000 counts.
Reportable Ion Abundance: > 50%.
LC Signal: DAD1 A, Sig=320,150 Ref=off
Peak matching window: 0.1 min

Retention Time (LC)	LC Area	Retention Time (MS)	MS Area	Mol. Weight or Ion
-	-	0.533	17619570	157.00 I
-	-	3.115	1310425	239.10 I
				217.00 I
-	-	3.989	162561	216.10 I
				200.00 I
				170.90 I
				159.00 I
				137.10 I
-	-	4.625	118658	170.80 I
				158.10 I
				137.10 I
-	-	4.783	74052	279.10 I
				137.10 I
-	-	5.197	490456	510.40 I
				338.20 I
				336.20 I
				316.20 I
				298.20 I
				252.10 I
				137.10 I
				105.10 I
-	-	5.736	167582	280.20 I
-	-	5.856	852424	318.20 I
				296.20 I
-	-	6.251	176272	228.20 I
				137.10 I
6.564	1980	6.595	7219776	626.10 I
				395.10 I
-	-	6.869	5583937	282.20 I
-	-	7.356	1240152	400.30 I
				282.20 I

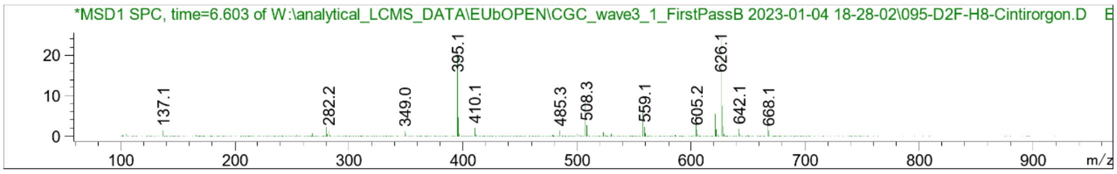

COMPOUND INFORMATION

Biological activity

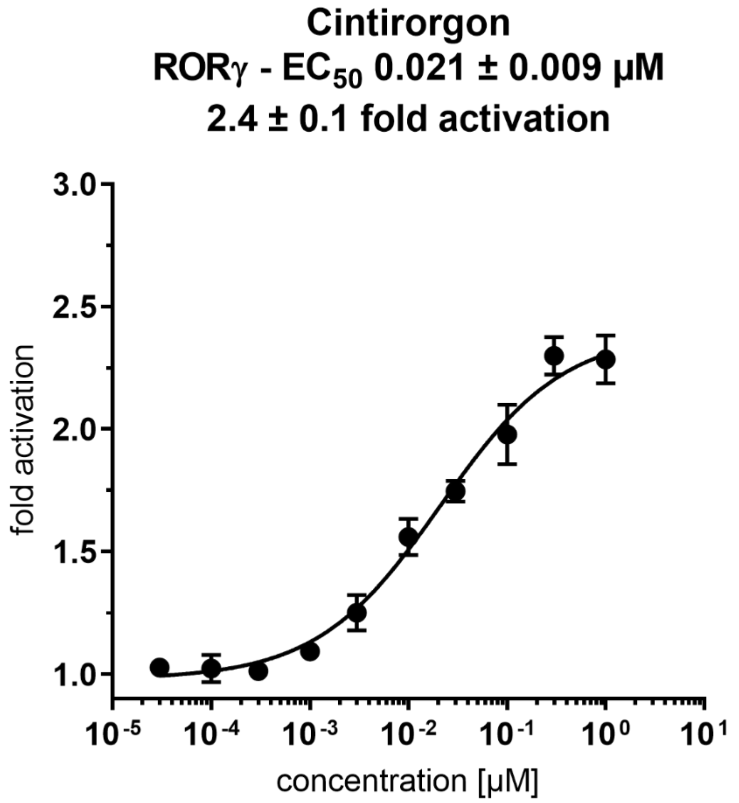

Supplement: Supplementary file 4 — Supplementary Data 1 [file 41467_2024_49493_MOESM4_ESM.zip › Cintirorgon.pdf]
