# Supplementary material for: Chemogenomics for NR1 nuclear hormone receptors
Source: Nat Commun. 2024 Jun 18;15:5201. doi: 10.1038/s41467-024-49493-6 (PMC11189487; doi:10.1038/s41467-024-49493-6)

COMPOUND INFORMATION

CITCO

**CAS Registry No.:**	338404-52-7
**Formal Name:**	(E)-6-(4-chlorophenyl)imidazo[2,1-b]thiazole-5-carbaldehyde O-(3,4-dichlorobenzyl) oxime
**EUbOPEN ID**	EUB0001477a
**Molecular Formula:**	$C_{19}H_{12}Cl_3N_3OS$
**Molecular Weight:**	436.74 g/mol
**Smiles:**	ClC1=CC=C(C=C1)C2=C(N3C(SC=C3)=N2)/C=N/OCC4=CC(Cl)=C(C=C4)Cl
**Recommended concentration:**	1 µM

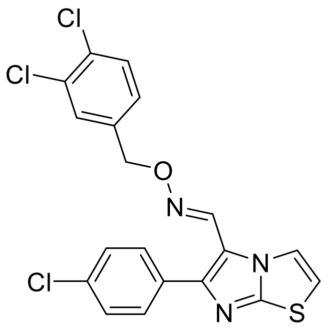

Biological activity

		Type	IC_{50}/EC_{50} [µM]	Reference
Main NR target:	NR1I3 (CAR)	Agonist	0.03	https://doi.org/10.1074/jbc.M300138200
NR off-targets:				

COMPOUND INFORMATION

Identity

^{1}H NMR

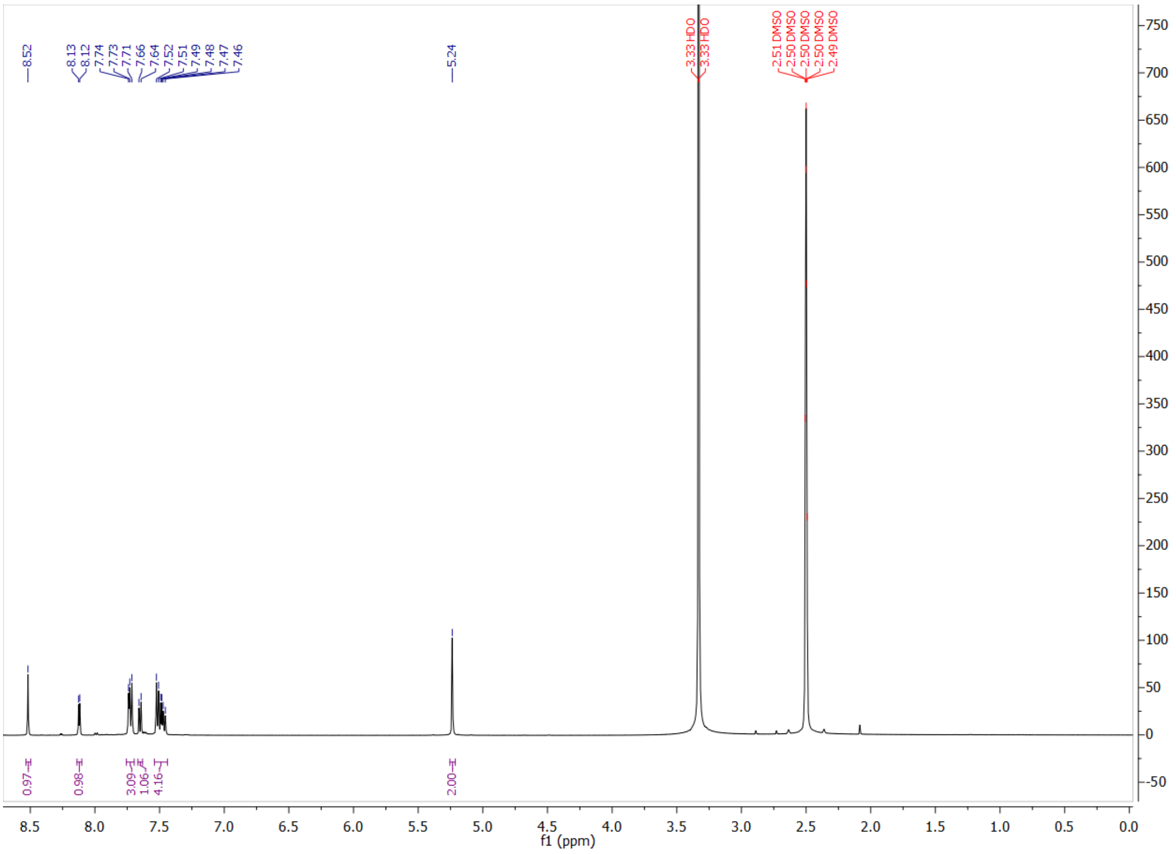

^{13}C NMR

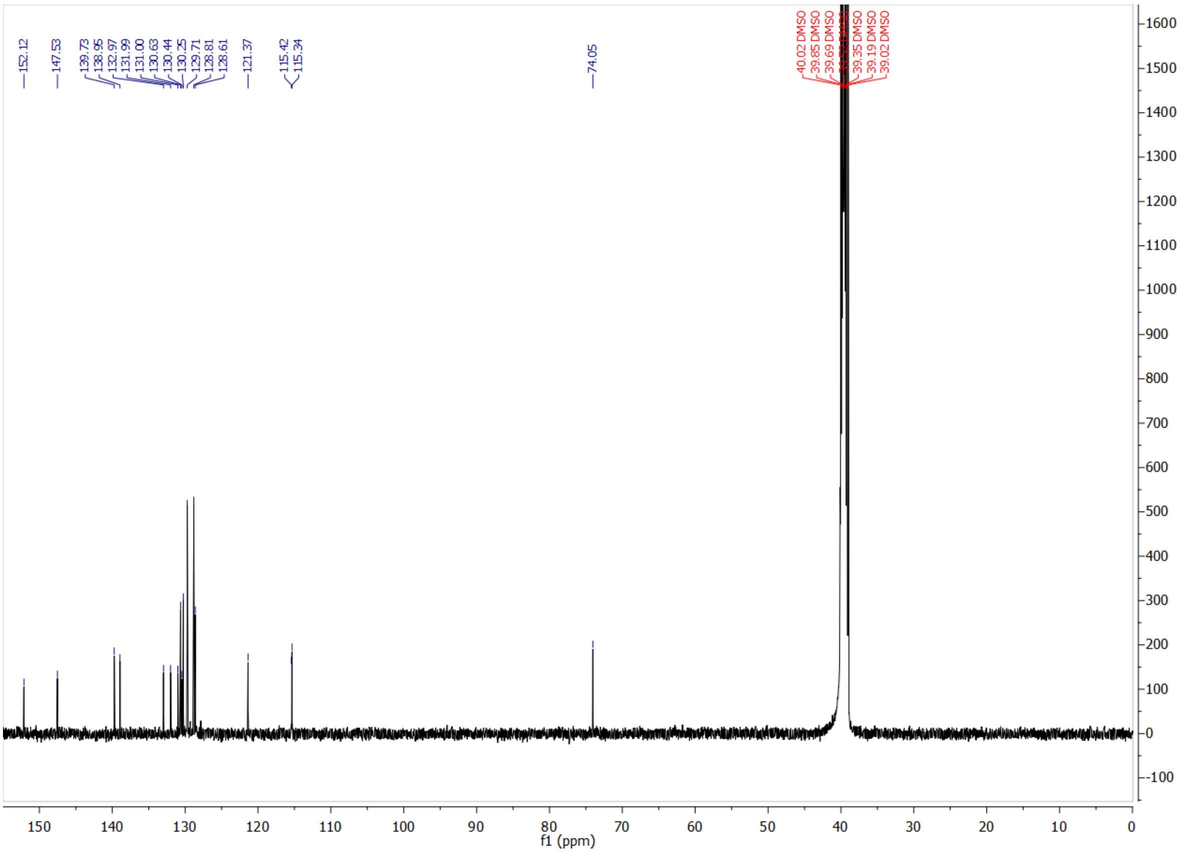

COMPOUND INFORMATION

Purity

Data File W:\analyti...UbOPEN\CGC_wave3_2_FirstPass 2023-01-05 22-10-41\034-D2F-C6-CITCO.D
Sample Name: CITCO

```
=====================================================================
Acq. Operator   : SYSTEM                          Seq. Line :  34
Sample Operator : SYSTEM
Acq. Instrument : LCMS test                        Location :   D2F-C6
Injection Date  : 1/6/2023 4:18:40 AM                   Inj :   1
                                                 Inj Volume : Inj prog
Sequence File   : W:\analytical_LCMS_DATA\EUbOPEN\CGC_wave3_2_FirstPass 2023-01-05 22-10-41
                  \CGC_wave3_2_FirstPass.S
Method          : W:\analytical_LCMS_DATA\EUbOPEN\CGC_wave3_2_FirstPass 2023-01-05 22-10-41
                  \CGL_FIRSTPASS_GENERALMETHOD_VIAL1+2_20210319.M (Sequence Method)
Last changed    : 1/25/2022 4:36:18 PM by SYSTEM
Method Info     : CGL wellplate, 0.5 uL of 10 mM DMSO, general method
```

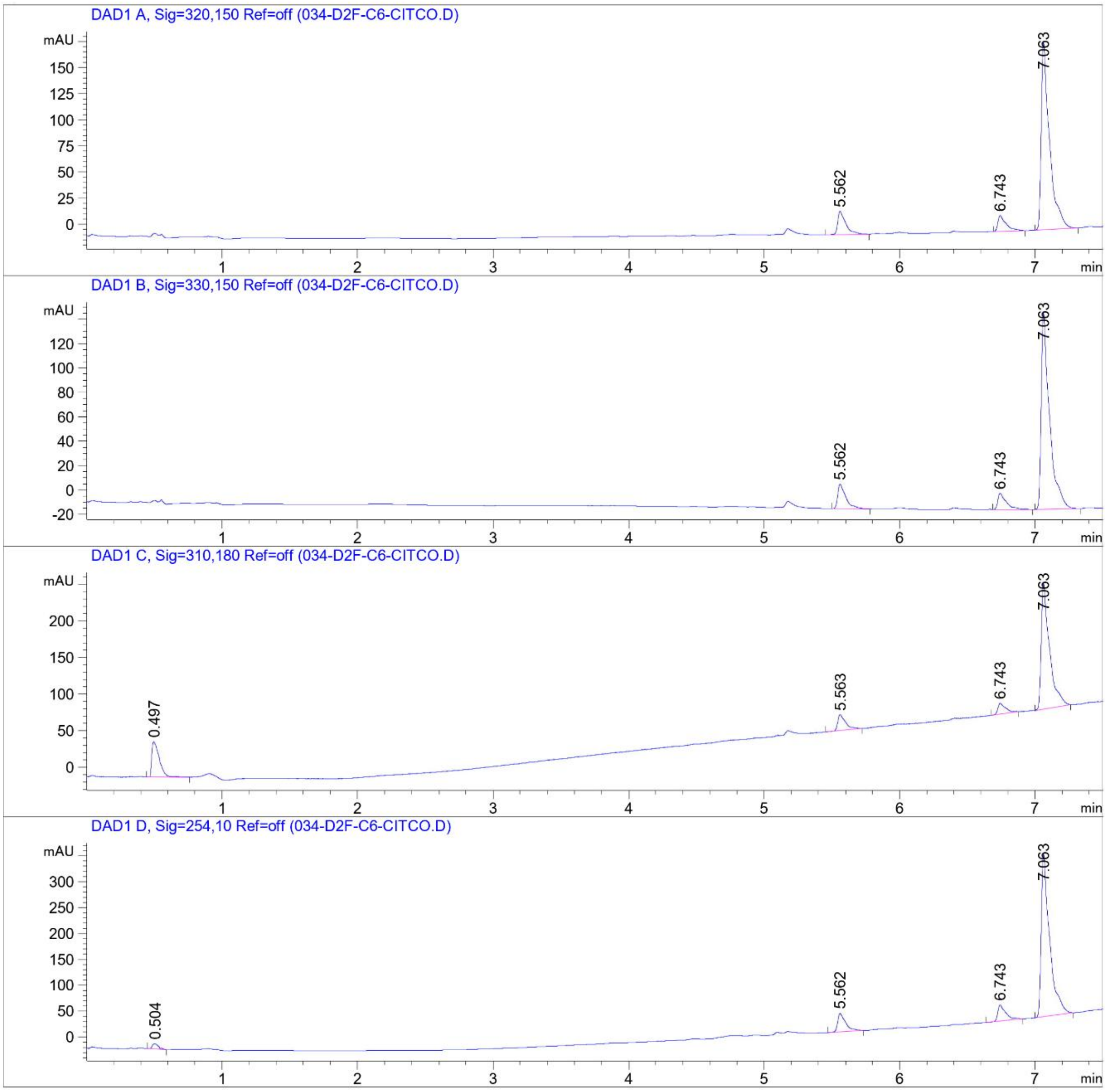

COMPOUND INFORMATION

EUbOPEN
Enabling & Unlocking Biology in the OPEN

Data File W:\analyti...UbOPEN\CGC_wave3_2_FirstPass 2023-01-05 22-10-41\034-D2F-C6-CITCO.D
Sample Name: CITCO

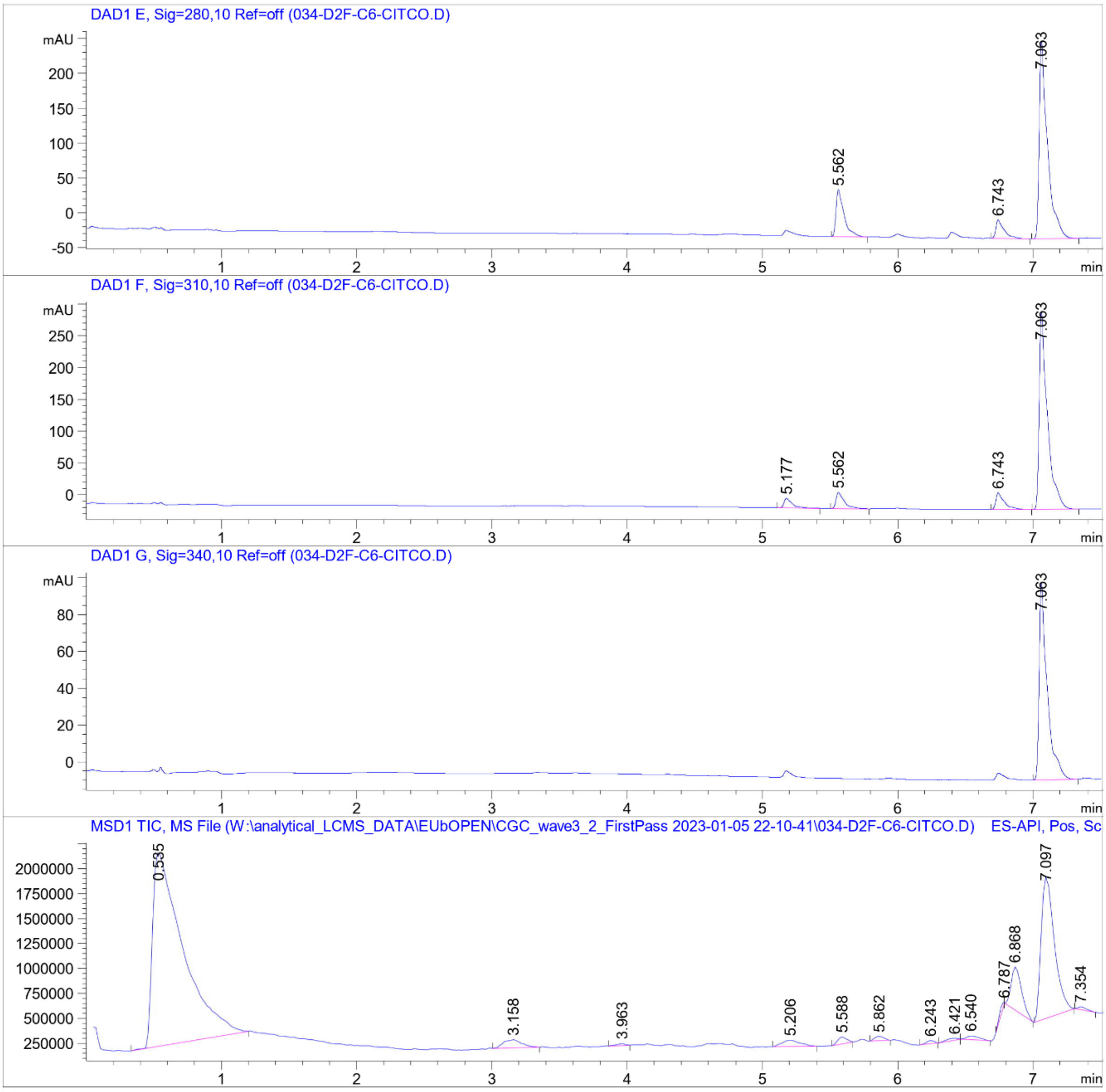

COMPOUND INFORMATION

Data File W:\analyti...UbOPEN\CGC_wave3_2_FirstPass 2023-01-05 22-10-41\034-D2F-C6-CITCO.D
Sample Name: CITCO

MS Signal: MSD1 TIC, MS File, ES-API, Pos, Scan, Frag: 70, "POS Scan"
Spectra from peak tops.
Noise Cutoff: 1000 counts.
Reportable Ion Abundance: > 50%.
LC Signal: DAD1 A, Sig=320,150 Ref=off
Peak matching window: 0.1 min

Retention Time (LC)	LC Area	Retention Time (MS)	MS Area	Mol. Weight or Ion
-	-	0.535	30099000	157.00 I
-	-	3.158	785364	188.10 I
				170.10 I
-	-	3.963	93181	420.10 I
				216.10 I
				210.60 I
-	-	5.206	603772	262.90 I
5.562	91	5.588	320900	260.00 I
-	-	5.862	207368	318.20 I
				296.20 I
-	-	6.243	130524	228.20 I
				137.10 I
-	-	6.421	135579	350.20 I
				282.20 I
				254.20 I
				137.10 I
-	-	6.540	264372	280.20 I
6.743	63	6.787	164896	438.00 I
				435.90 I
				256.20 I
-	-	6.868	2328094	282.20 I
7.063	760	7.097	9609414	437.90 I
				435.90 I
-	-	7.354	125122	400.30 I
				282.20 I

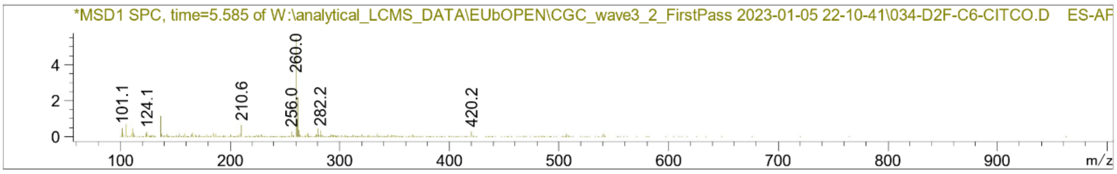

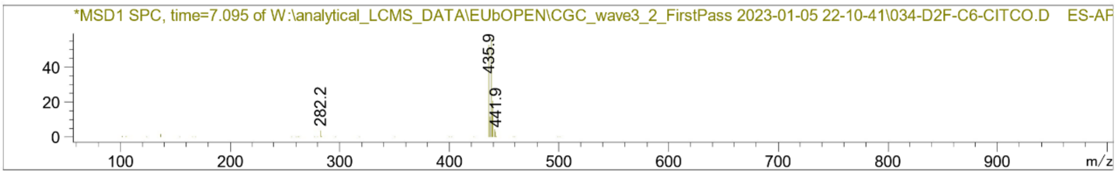

Supplement: Supplementary file 4 — Supplementary Data 1 [file 41467_2024_49493_MOESM4_ESM.zip › CITCO.pdf]
