# Supplementary material for: Chemogenomics for NR1 nuclear hormone receptors
Source: Nat Commun. 2024 Jun 18;15:5201. doi: 10.1038/s41467-024-49493-6 (PMC11189487; doi:10.1038/s41467-024-49493-6)

# COMPOUND INFORMATION

# CP775146

| | |
|---|---|
| **CAS Registry No.:** | 702680-17-9 |
| **Formal Name:** | 2-(3-(1-(2-(4-isopropylphenyl)acetyl)piperidin-3-yl)phenoxy)-2-methylpropanoic acid |
| **EUbOPEN ID:** | EUB0000581a |
| **Molecular Formula:** | $C_{26}H_{33}NO_4$ |
| **Molecular Weight:** | 423.55 g/mol |
| **Smiles:** | CC(C)C1=CC=C(C=C1)CC(=O)N2CCCC(C2)C3=CC(=CC=C3)OC(C)(C)C(=O)O |
| **Recommended concentration:** | 1 µM |

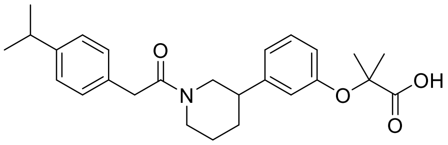


**Biological activity**

| | | Type | $IC_{50}$/$EC_{50}$ [µM] | Reference |
|---|---|---|---|---|
| Main NR target: | NR1C1 (PPARα) | Agonist | 0.06 | https://doi.org/10.1124/mol.108.051656 |
| NR off-target: | | | | |

# **COMPOUND** INFORMATION

## Identity

### $^1$H NMR

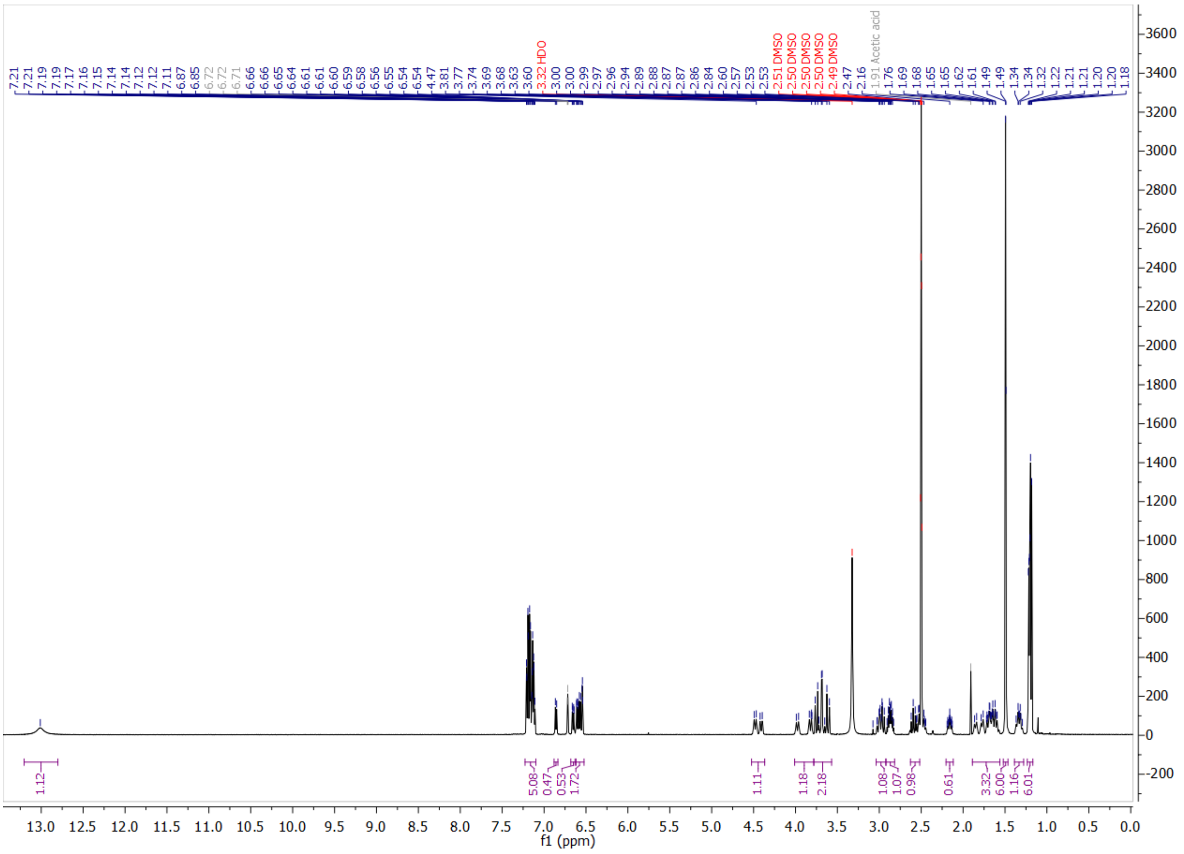


### $^{13}$C NMR

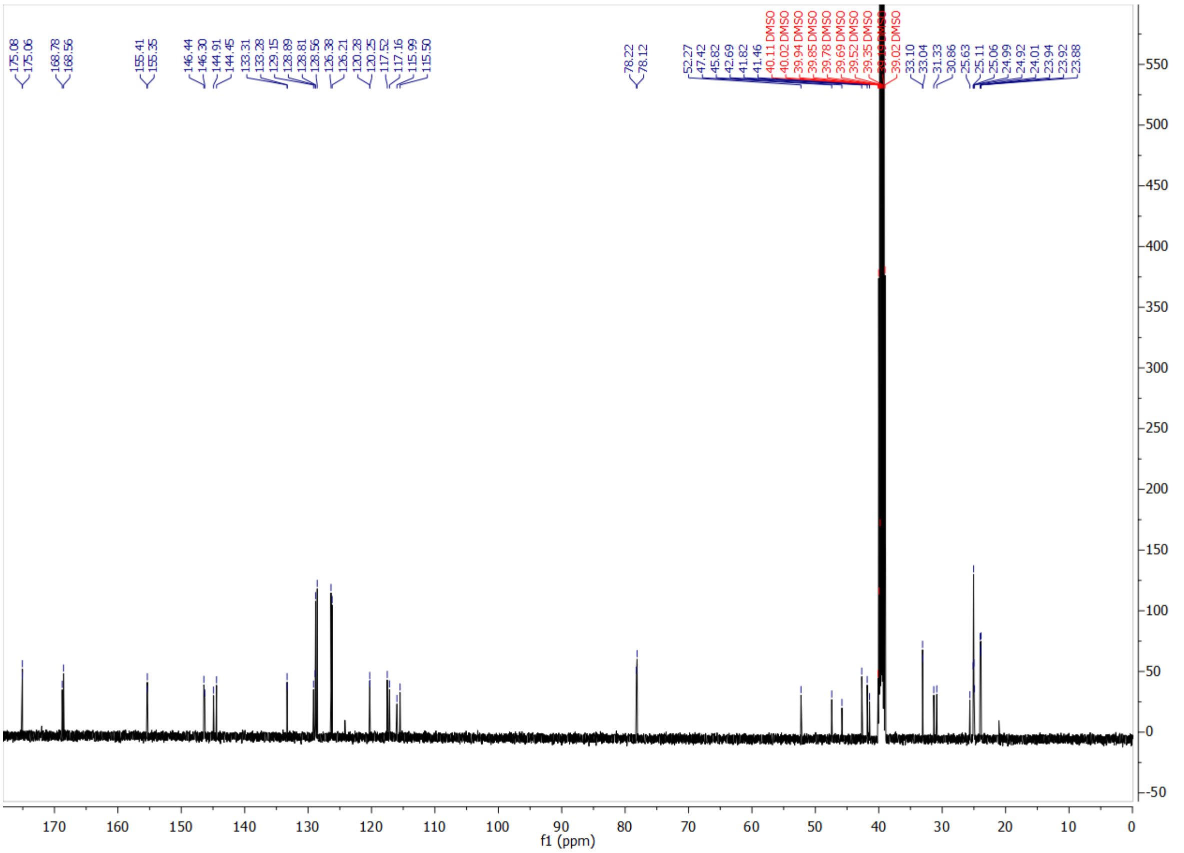

# **COMPOUND** INFORMATION

## Purity

```
Data File W:\analyti...N\CGC_ECHO1-3_SecondPass 2021-03-25 18-09-21\018-D2F-H4-CP 775146.D
Sample Name: CP 775146

    ====================================================================
    Acq. Operator   : SYSTEM                        Seq. Line :  18
    Sample Operator : SYSTEM
    Acq. Instrument : LCMS test                      Location :   D2F-H4
    Injection Date  : 3/25/2021 9:19:43 PM                Inj :   1
                                                   Inj Volume : Inj prog
    Sequence File   : W:\analytical_LCMS_DATA\EUbOPEN\CGC_ECHO1-3_SecondPass 2021-03-25 18-09-21
                      \CGC_ECHO1-3_SecondPass.S
    Method          : W:\analytical_LCMS_DATA\EUbOPEN\CGC_ECHO1-3_SecondPass 2021-03-25 18-09-21
                      \CGL_SECONDPASS_NONPOLCOMP_VIAL2+4_20210323.M (Sequence Method)
    Last changed    : 3/25/2021 4:32:02 PM by SYSTEM
    Method Info     : CGL wellplate, 0.5 uL of 10 mM DMSO. Dilution with MeCN only (9+9 uL)
```

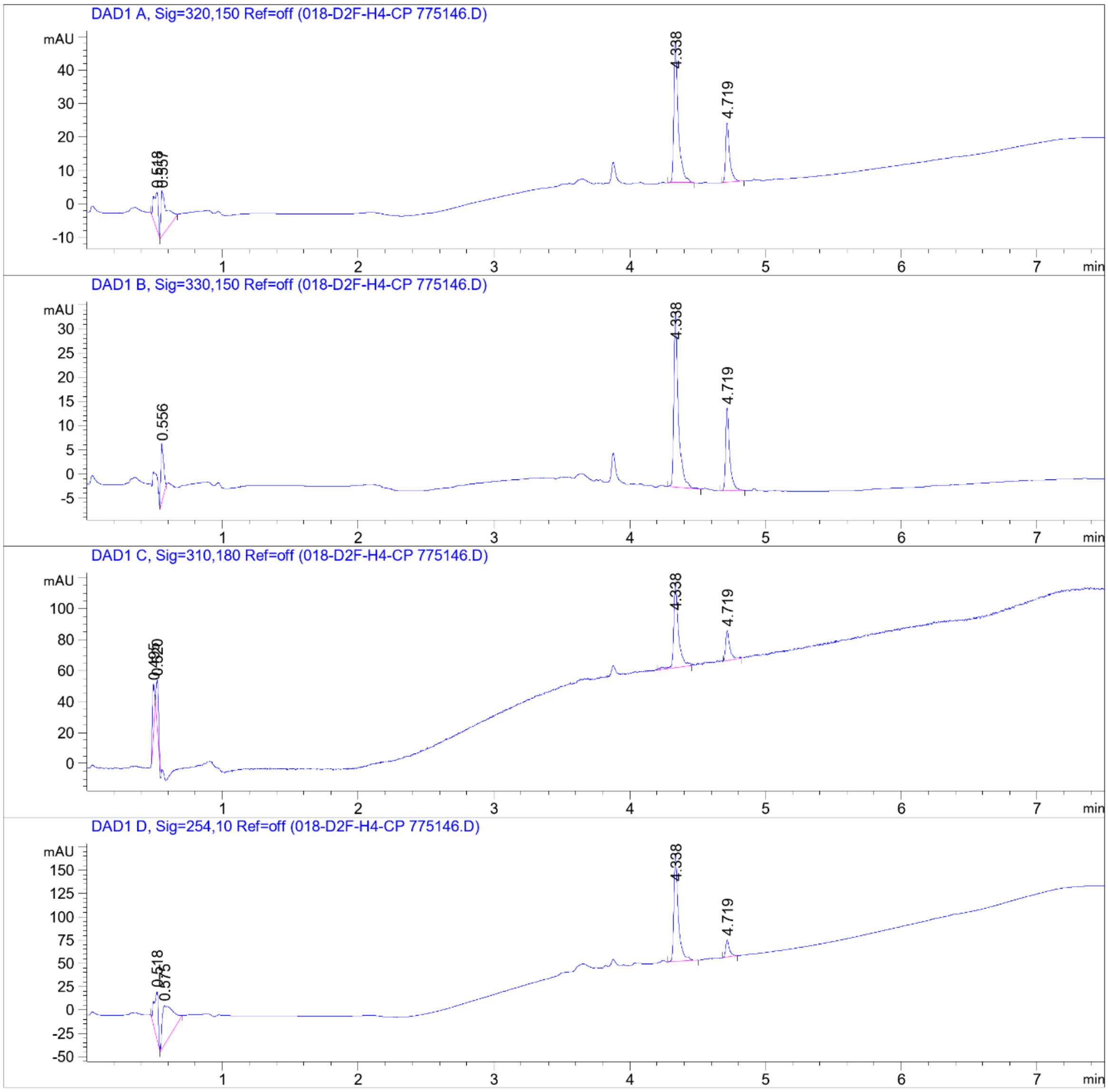

# **COMPOUND** INFORMATION

Data File W:\analyti...N\CGC_ECHO1-3_SecondPass 2021-03-25 18-09-21\018-D2F-H4-CP 775146.D
Sample Name: CP 775146

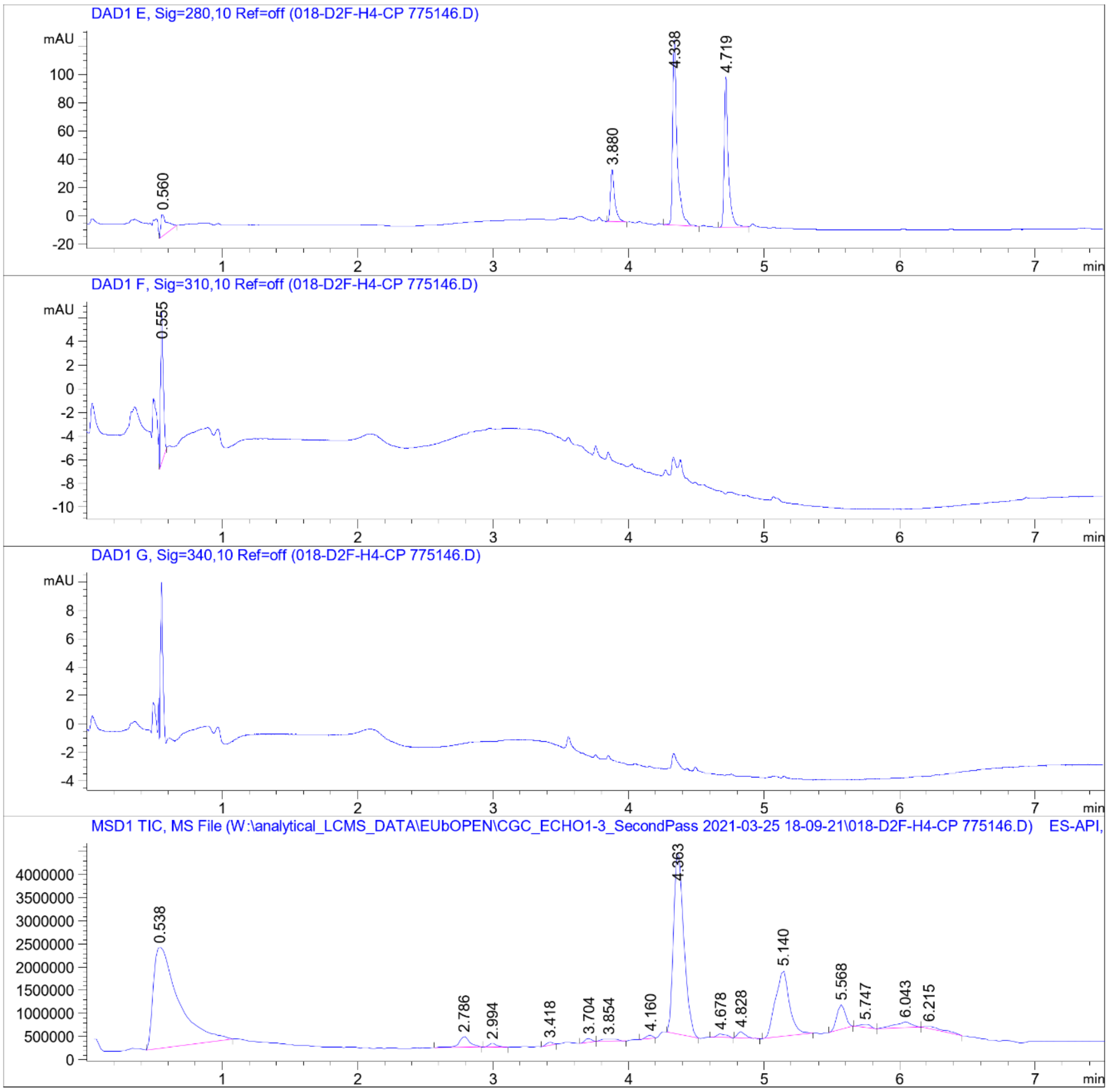

# **COMPOUND** INFORMATION

Data File W:\analyti...N\CGC_ECHO1-3_SecondPass 2021-03-25 18-09-21\018-D2F-H4-CP 775146.D
Sample Name: CP 775146

MS Signal: MSD1 TIC, MS File, ES-API, Pos, Scan, Frag: 70, "POS Scan"
Spectra from peak tops.
Noise Cutoff: 1000 counts.
Reportable Ion Abundance: > 50%.
LC Signal: DAD1 A, Sig=320,150 Ref=off
Peak matching window: 0.1 min

| Retention Time (LC) | LC Area | Retention Time (MS) | MS Area | Mol. Weight or Ion |
|---|---|---|---|---|
| 0.518 | 25 | 0.538 | 28108878 | 157.00 I |
| 0.557 | 40 | - | - | |
| - | - | 2.786 | 1241209 | 217.10 I |
| - | - | 2.994 | 292907 | 274.30 I |
| - | - | 3.418 | 212291 | 326.40 I |
| - | - | 3.704 | 296369 | 214.10 I |
| - | - | 3.854 | 412384 | 316.30 I<br>298.30 I<br>282.30 I<br>225.10 I<br>111.00 I |
| - | - | 4.160 | 219673 | 280.20 I |
| 4.338 | 97 | 4.363 | 21073622 | 424.30 I |
| 4.719 | 37 | 4.678 | 356022 | 254.20 I |
| - | - | 4.828 | 517532 | 280.20 I |
| - | - | 5.140 | 10159769 | 282.30 I |
| - | - | 5.568 | 2274933 | 381.30 I<br>359.30 I<br>341.30 I<br>284.30 I<br>282.30 I |
| - | - | 5.747 | 369979 | 400.40 I<br>282.30 I |
| - | - | 6.043 | 1025184 | 282.30 I |
| - | - | 6.215 | 546708 | 282.30 I |

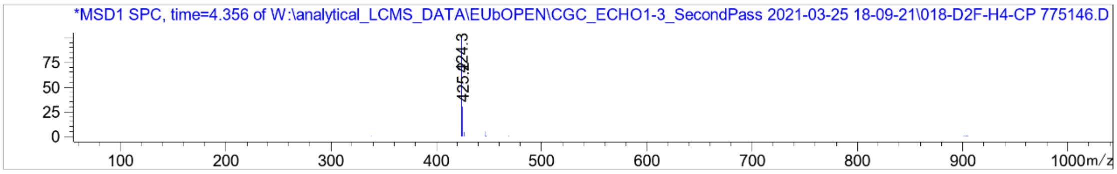

Supplement: Supplementary file 4 — Supplementary Data 1 [file 41467_2024_49493_MOESM4_ESM.zip › CP775146.pdf]
