# Supplementary material for: Chemogenomics for NR1 nuclear hormone receptors
Source: Nat Commun. 2024 Jun 18;15:5201. doi: 10.1038/s41467-024-49493-6 (PMC11189487; doi:10.1038/s41467-024-49493-6)

COMPOUND INFORMATION

DG-172

**CAS Registry No.:**	1361504-77-9
**Formal Name:**	(Z)-2-(2-bromophenyl)-3-(4-(4-methylpiperazin-1-yl)phenyl)acrylonitrile dihydrochloride
**EUbOPEN ID:**	EUB0001483aCl
**Molecular Formula:**	$C_{20}H_{22}BrCl_2N_3$
**Molecular Weight:**	455.22 g/mol
**Smiles:**	CN1CCN(CC1)C2=CC=C(C=C2)C=C(C#N)C3=CC=CC=C3Br
**Recommended concentration:**	10 µM

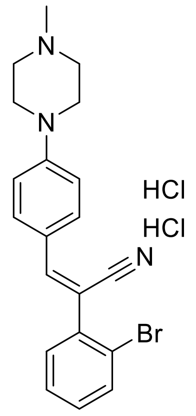

Biological activity

		Type	IC_{50}/EC_{50} [µM]	Reference
Main NR target:	NR1C2 (PPARδ)	Antagonist	1.8	inhouse
NR off-target:				

COMPOUND INFORMATION

Identity

^{1}H NMR

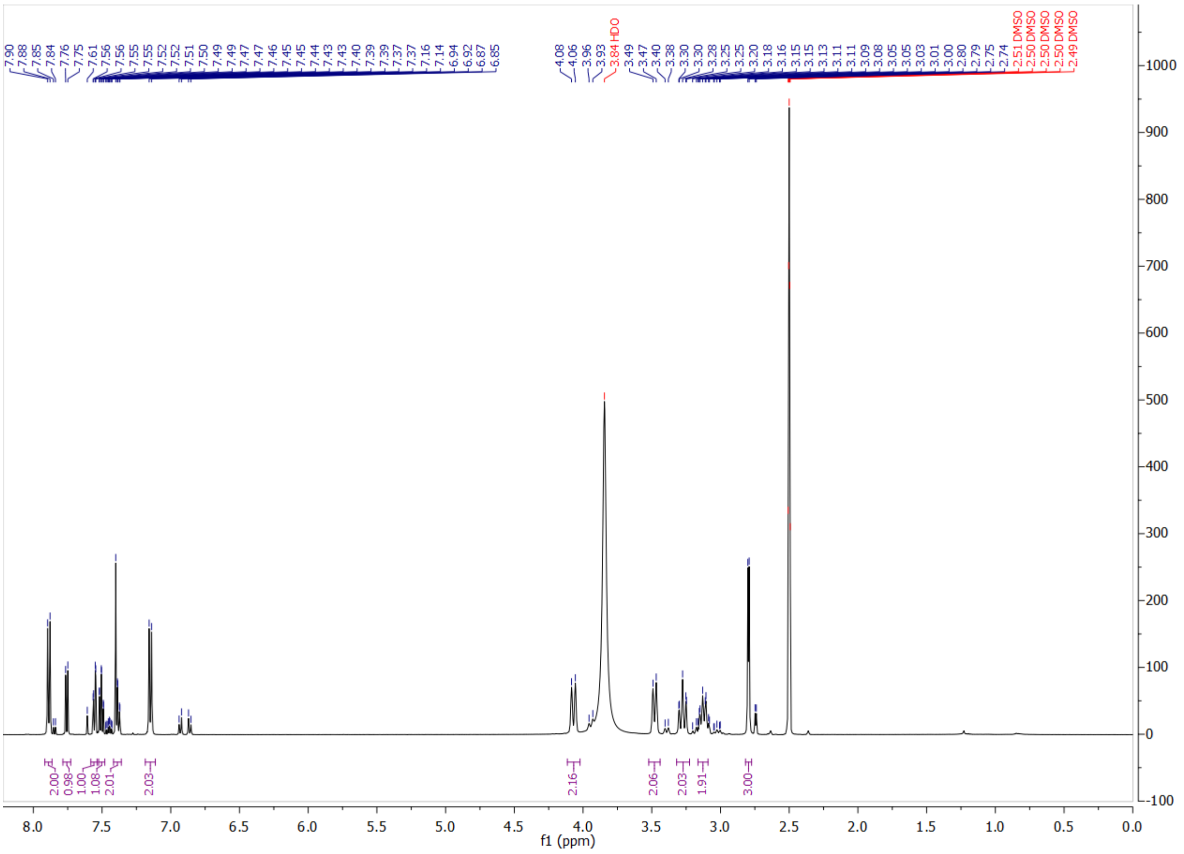

^{13}C NMR

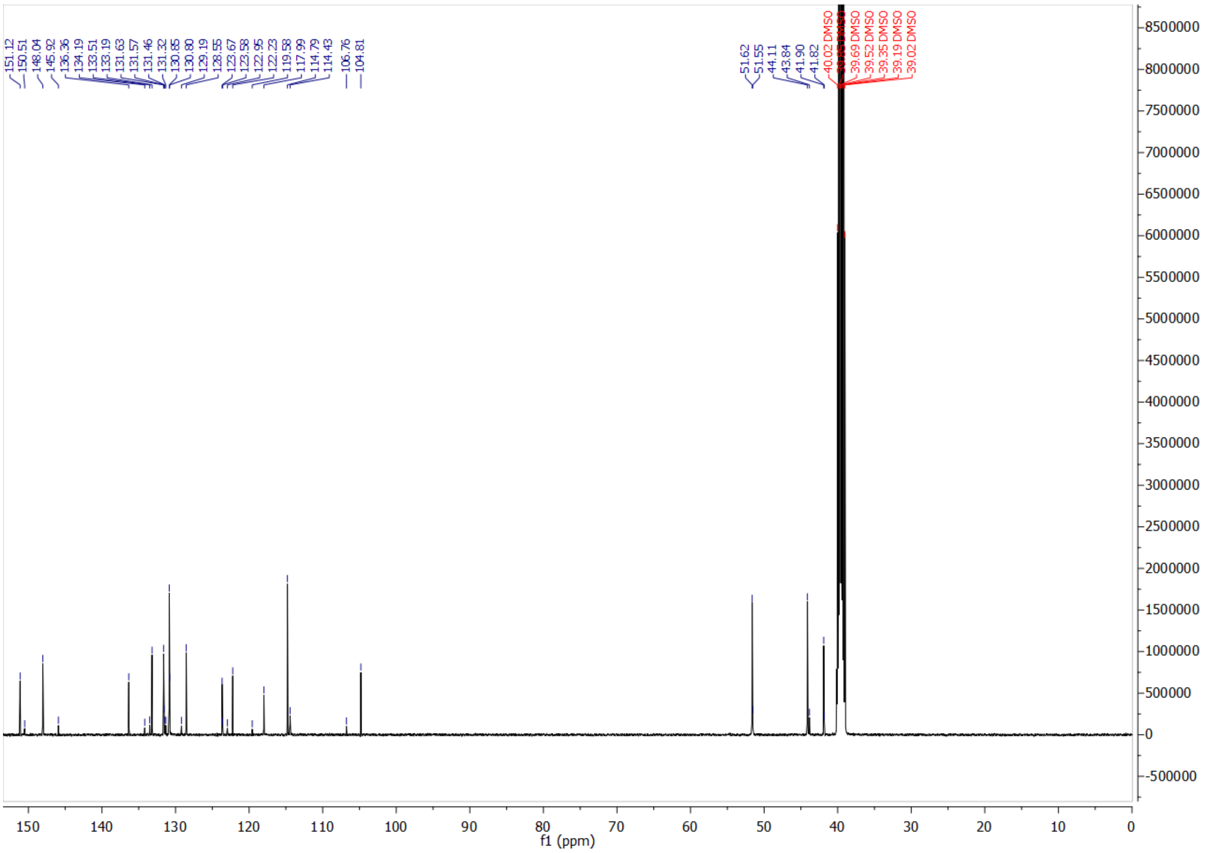

COMPOUND INFORMATION

Purity

Data File W:\analyti...bOPEN\CGC_wave3_2_FirstPass 2023-01-05 22-10-41\038-D2F-D1-DG-172.D
Sample Name: DG-172

```
=====================================================================
Acq. Operator   : SYSTEM                         Seq. Line :  38
Sample Operator : SYSTEM
Acq. Instrument : LCMS test                       Location :  D2F-D1
Injection Date  : 1/6/2023 5:02:53 AM                  Inj :  1
                                                Inj Volume : Inj prog
Sequence File   : W:\analytical_LCMS_DATA\EUbOPEN\CGC_wave3_2_FirstPass 2023-01-05 22-10-41
                  \CGC_wave3_2_FirstPass.S
Method          : W:\analytical_LCMS_DATA\EUbOPEN\CGC_wave3_2_FirstPass 2023-01-05 22-10-41
                  \CGL_FIRSTPASS_GENERALMETHOD_VIAL1+2_20210319.M (Sequence Method)
Last changed    : 1/25/2022 4:36:18 PM by SYSTEM
Method Info     : CGL wellplate, 0.5 uL of 10 mM DMSO, general method
```

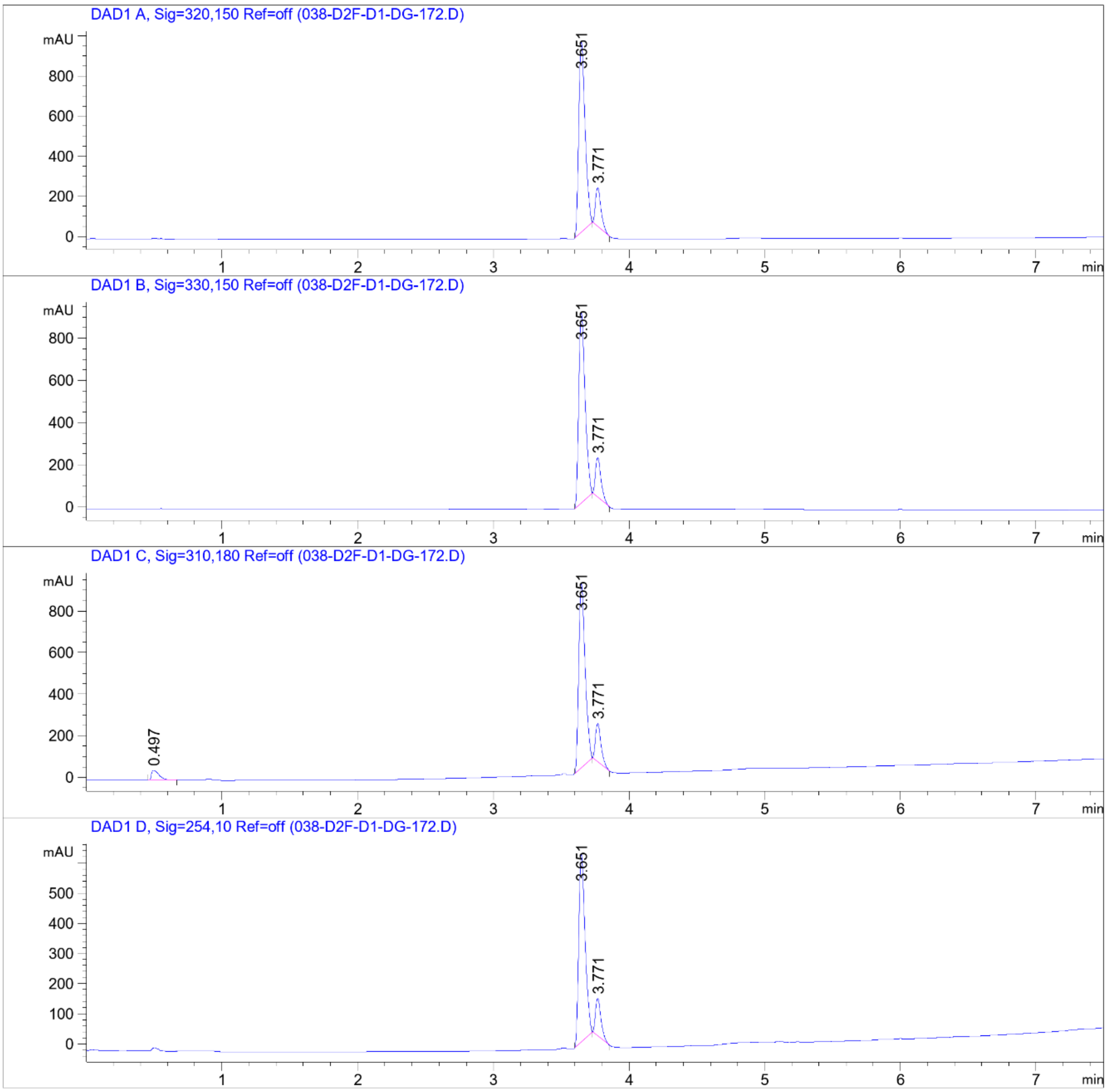

COMPOUND INFORMATION

Data File W:\analyti...bOPEN\CGC_wave3_2_FirstPass 2023-01-05 22-10-41\038-D2F-D1-DG-172.D
Sample Name: DG-172

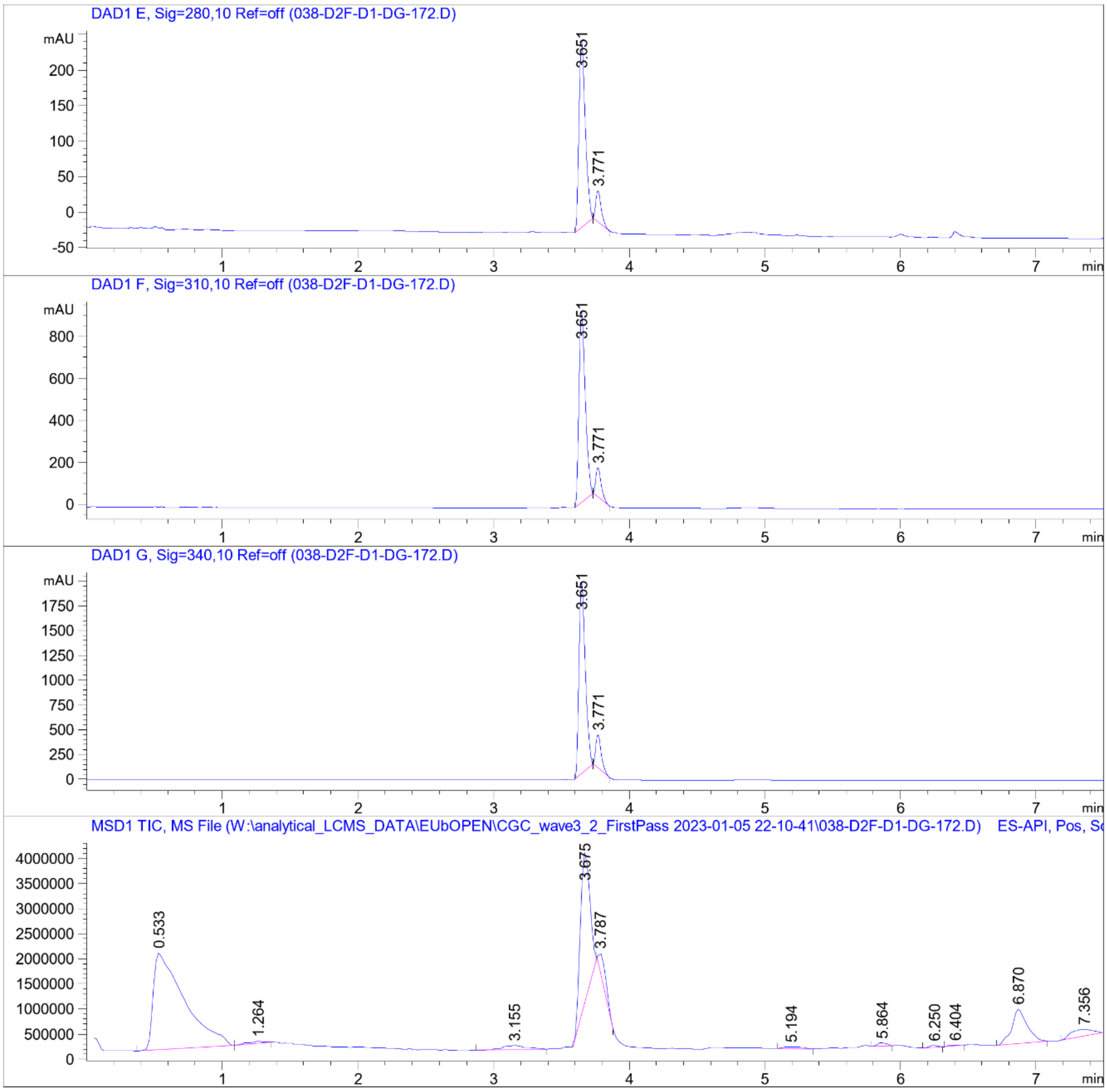

COMPOUND INFORMATION

Data File W:\analyti...bOPEN\CGC_wave3_2_FirstPass 2023-01-05 22-10-41\038-D2F-D1-DG-172.D
Sample Name: DG-172

MS Signal: MSD1 TIC, MS File, ES-API, Pos, Scan, Frag: 70, "POS Scan"
Spectra from peak tops.
Noise Cutoff: 1000 counts.
Reportable Ion Abundance: > 50%.
LC Signal: DAD1 A, Sig=320,150 Ref=off
Peak matching window: 0.1 min

Retention Time (LC)	LC Area	Retention Time (MS)	MS Area	Mol. Weight or Ion
-	-	0.533	30619936	157.10 I
-	-	1.264	319703	157.00 I
-	-	3.155	1029673	188.10 I
				170.10 I
3.651	3092	3.675	14391690	384.10 I
				382.10 I
3.771	552	3.787	1586792	384.10 I
				382.00 I
-	-	5.194	344387	338.20 I
				336.20 I
				316.30 I
				298.20 I
				137.10 I
-	-	5.864	281108	318.20 I
				296.20 I
-	-	6.250	171081	228.20 I
				137.10 I
-	-	6.404	129809	282.20 I
				254.20 I
				137.10 I
-	-	6.870	5441228	282.20 I
-	-	7.356	1543379	400.30 I
				282.20 I

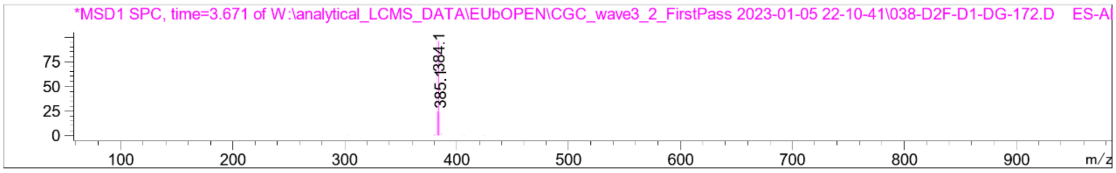

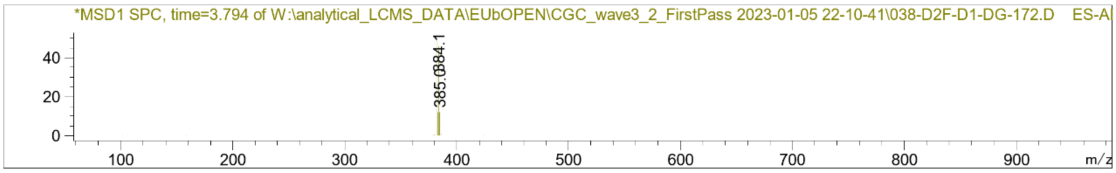

COMPOUND INFORMATION

Biological activity

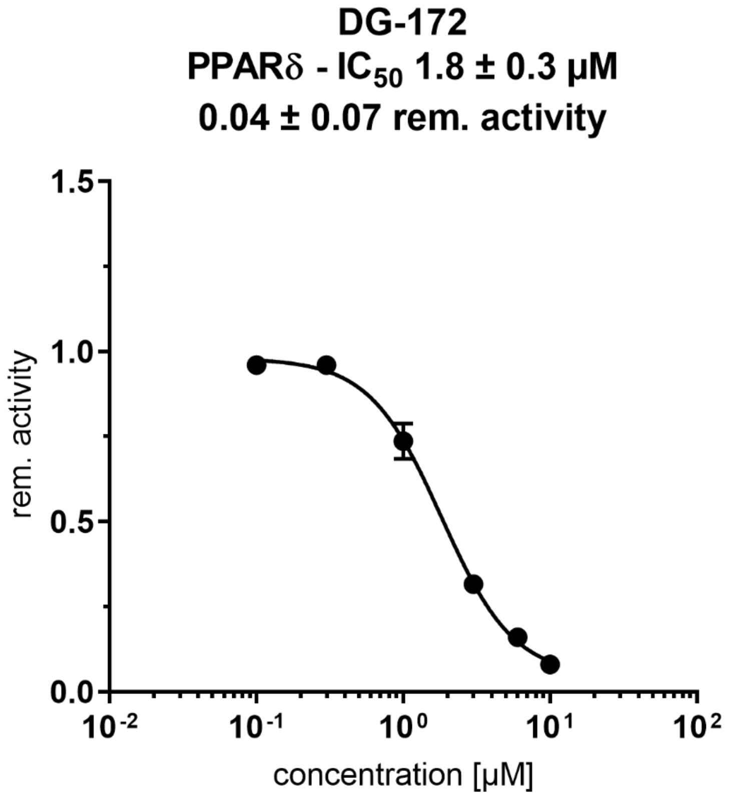

Supplement: Supplementary file 4 — Supplementary Data 1 [file 41467_2024_49493_MOESM4_ESM.zip › DG-172.pdf]
