# Supplementary material for: Chemogenomics for NR1 nuclear hormone receptors
Source: Nat Commun. 2024 Jun 18;15:5201. doi: 10.1038/s41467-024-49493-6 (PMC11189487; doi:10.1038/s41467-024-49493-6)

COMPOUND INFORMATION

Doxercalciferol

**CAS Registry No.:**	54573-75-0
**Formal Name:**	(1R,3S,Z)-5-(2-((1R,3aS,7aR,E)-1-((2R,5R,E)-5,6-dimethylhept-3-en-2-yl)-7a-methyloctahydro-4H-inden-4-ylidene)ethylidene)-4-methylenecyclohexane-1,3-diol
**EUBOPEN ID:**	EUB0000583a
**Molecular Formula:**	$C_{28}H_{44}O_2$
**Molecular Weight:**	412.66 g/mol
**Smiles:**	C[C@@H]([C@H]1CC[C@@H]2[C@]1(C)CCC/C2=C\C=C3C[C@@H](O)C[C@H](O)C\3=C)/C=C/[C@@H](C(C)C)C
**Recommended concentration:**	1 µM

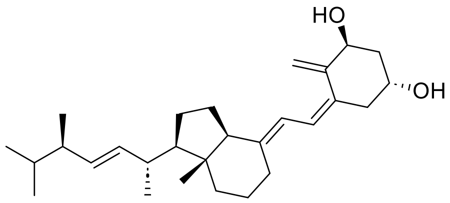

Biological activity

		Type	IC_{50}/EC_{50} [µM]	Reference
Main NR target:	NR1I1 (VDR)	Agonist	0.96	inhouse
NR off-target:				

COMPOUND INFORMATION

Identity

^{1}H NMR

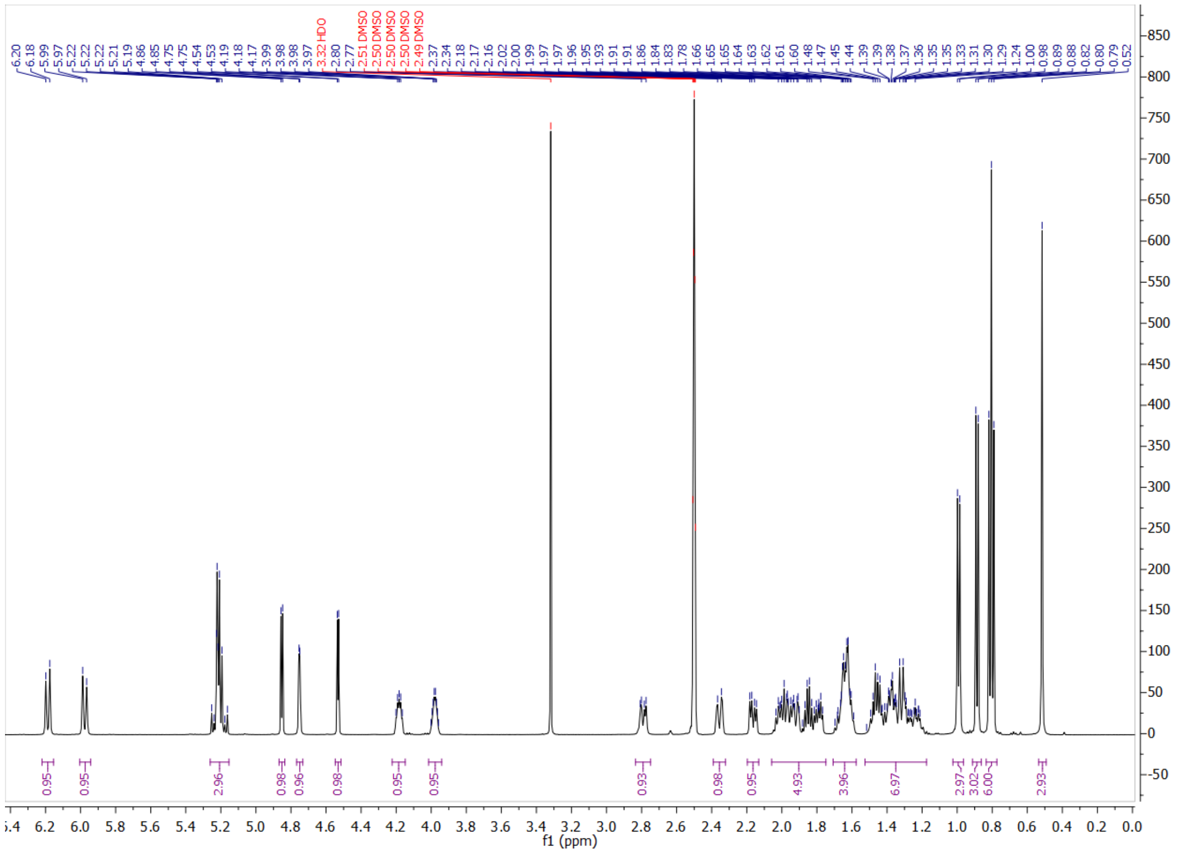

^{13}C NMR

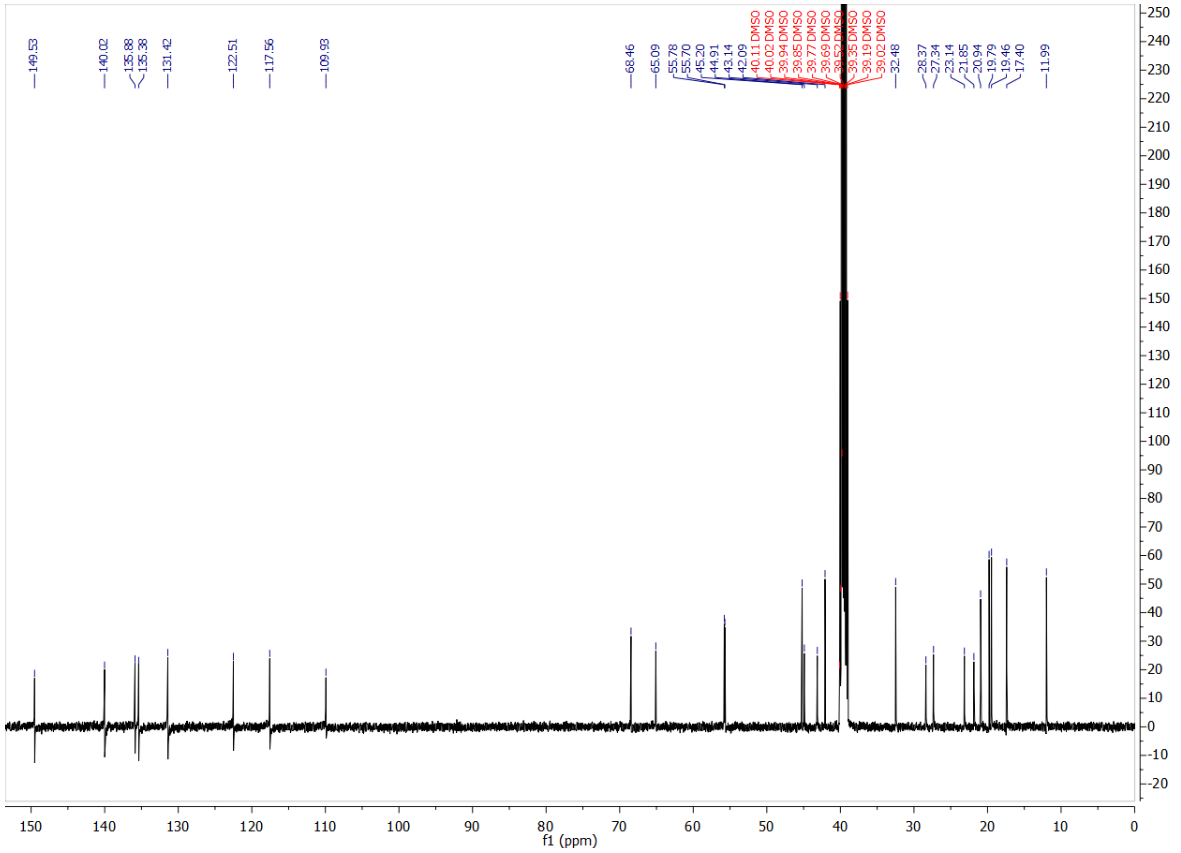

Purity

```
Data File W:\analyti...CHO1-3_SecondPass 2021-03-25 18-09-21\035-D2F-G10-doxercalciferol.D
Sample Name: doxercalciferol

    =====================================================================
    Acq. Operator   : SYSTEM                          Seq. Line :  35
    Sample Operator : SYSTEM
    Acq. Instrument : LCMS test                        Location :   D2F-G10
    Injection Date  : 3/26/2021 12:27:14 AM                 Inj :   1
                                                     Inj Volume : Inj prog
    Sequence File   : W:\analytical_LCMS_DATA\EUbOPEN\CGC_ECHO1-3_SecondPass 2021-03-25 18-09-21
                      \CGC_ECHO1-3_SecondPass.S
    Method          : W:\analytical_LCMS_DATA\EUbOPEN\CGC_ECHO1-3_SecondPass 2021-03-25 18-09-21
                      \CGL_SECONDPASS_NONPOLCOMP_VIAL2+4_20210323.M (Sequence Method)
    Last changed    : 3/25/2021 4:32:02 PM by SYSTEM
    Method Info     : CGL wellplate, 0.5 uL of 10 mM DMSO. Dilution with MeCN only (9+9 uL)
```

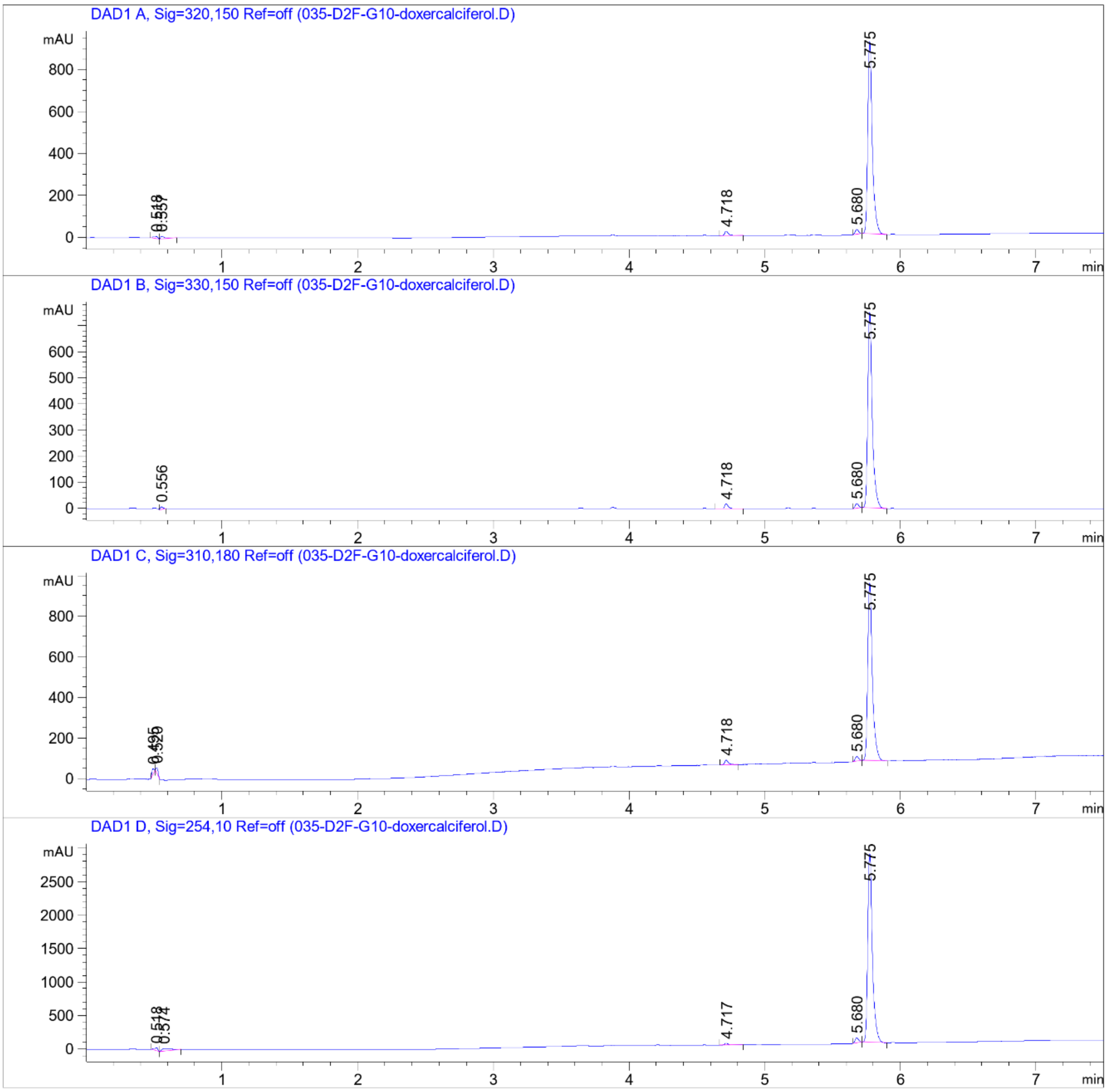

COMPOUND INFORMATION

Data File W:\analyti...CHO1-3_SecondPass 2021-03-25 18-09-21\035-D2F-G10-doxercalciferol.D
Sample Name: doxercalciferol

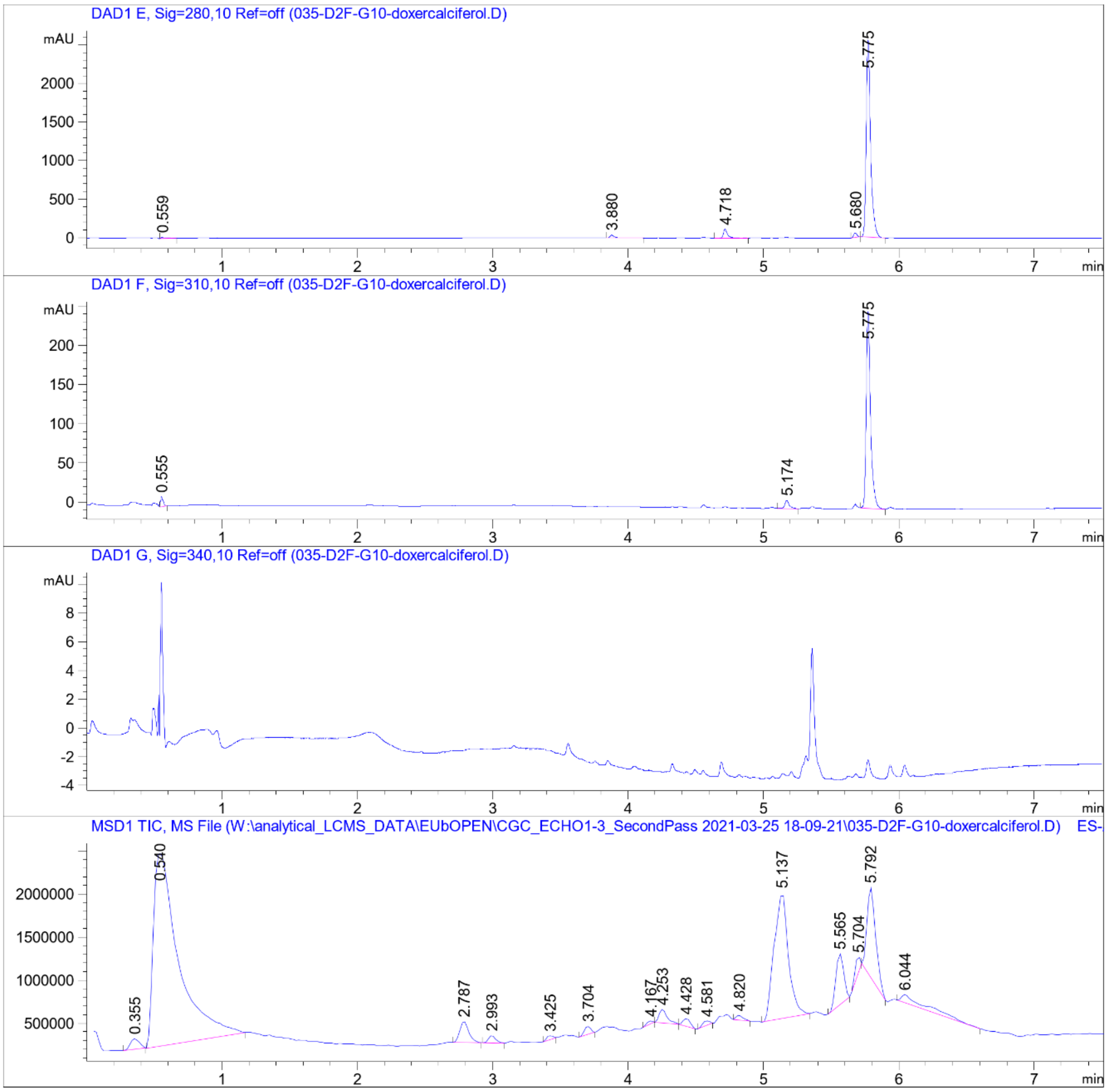

COMPOUND INFORMATION

Data File W:\analyti...CHO1-3_SecondPass 2021-03-25 18-09-21\035-D2F-G10-doxercalciferol.D
Sample Name: doxercalciferol

MS Signal: MSD1 TIC, MS File, ES-API, Pos, Scan, Frag: 70, "POS Scan"
Spectra from peak tops.
Noise Cutoff: 1000 counts.
Reportable Ion Abundance: > 50%.
LC Signal: DAD1 A, Sig=320,150 Ref=off
Peak matching window: 0.1 min

Retention Time (LC)	LC Area	Retention Time (MS)	MS Area	Mol. Weight or Ion
-	-	0.355	559757	200.00 I
				183.00 I
				159.00 I
				142.00 I
				110.10 I
0.518	23	0.540	28999792	157.10 I
0.557	37	-	-	
-	-	2.787	1152758	217.10 I
-	-	2.993	337384	274.30 I
-	-	3.425	149487	326.30 I
-	-	3.704	303418	312.20 I
				214.10 I
-	-	4.167	108858	280.20 I
-	-	4.253	658168	296.20 I
-	-	4.428	322461	298.20 I
				282.20 I
				280.20 I
-	-	4.581	178372	228.10 I
4.718	45	-	-	
-	-	4.820	211631	280.20 I
-	-	5.137	10566713	282.20 I
-	-	5.565	2348025	381.30 I
				359.20 I
				341.30 I
				284.20 I
				282.20 I
5.680	40	5.704	482055	400.30 I
				395.30 I
				282.20 I
5.775	2136	5.792	4661668	413.30 I
				395.30 I
-	-	6.044	1293452	338.30 I
				282.20 I

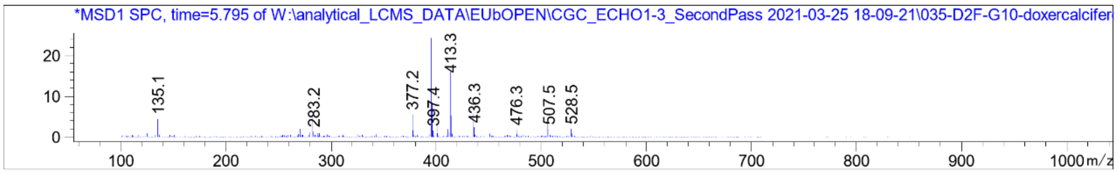

COMPOUND INFORMATION

Biological activity

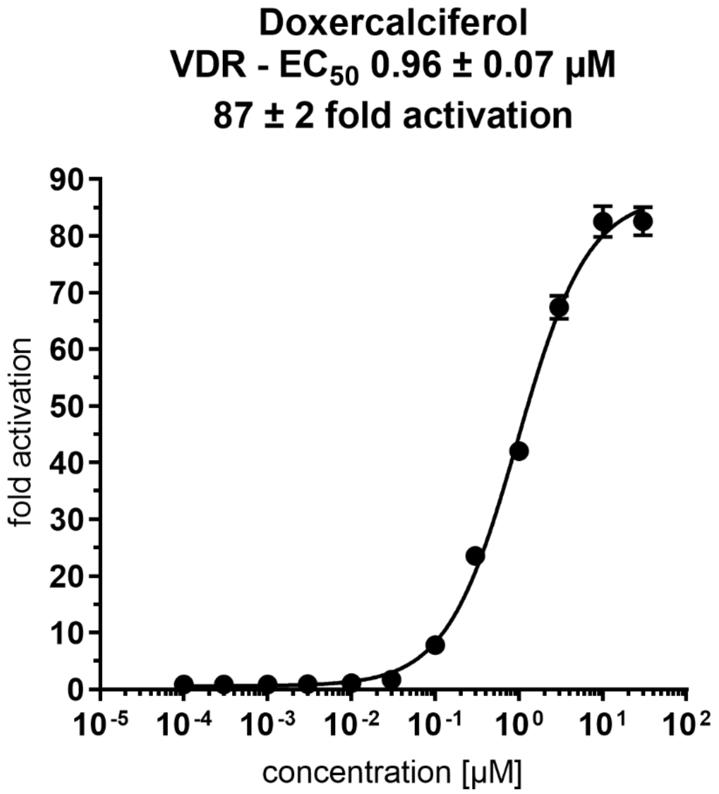

Supplement: Supplementary file 4 — Supplementary Data 1 [file 41467_2024_49493_MOESM4_ESM.zip › Doxercalciferol.pdf]
