# Supplementary material for: Chemogenomics for NR1 nuclear hormone receptors
Source: Nat Commun. 2024 Jun 18;15:5201. doi: 10.1038/s41467-024-49493-6 (PMC11189487; doi:10.1038/s41467-024-49493-6)

COMPOUND INFORMATION

DY268

**CAS Registry No.:**	1609564-75-1
**Formal Name:**	1-(3-methoxybenzyl)-N-(4-methyl-3-(morpholinosulfonyl)phenyl)-3-(p-tolyl)-1H-pyrazole-4-carboxamide
**EUBOPEN ID:**	EUB0001174a
**Molecular Formula:**	$C_{30}H_{32}N_4O_5S$
**Molecular Weight:**	560.67 g/mol
**Smiles:**	CC1=CC=C(C2=NN(C=C2C(NC3=CC=C(C(S(N4CCOCC4)(=O)=O)=C3)C)=O)CC5=CC=CC(OC)=C5)C=C1
**Recommended concentration:**	1 µM

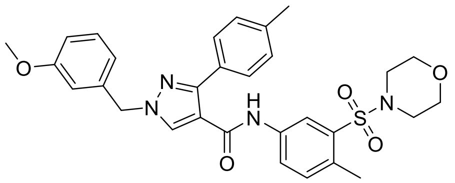

Biological activity

		Type	IC_{50}/EC_{50} [µM]	Reference
Main NR target:	NR1H4 (FXR)	Antagonist	0.58	inhouse
NR off-target:	NR1H3 (LXRα)	Antagonist	1.6	inhouse
	NR1H2 (LXRβ)	Antagonist	1.3	

COMPOUND INFORMATION

Identity

^{1}H NMR

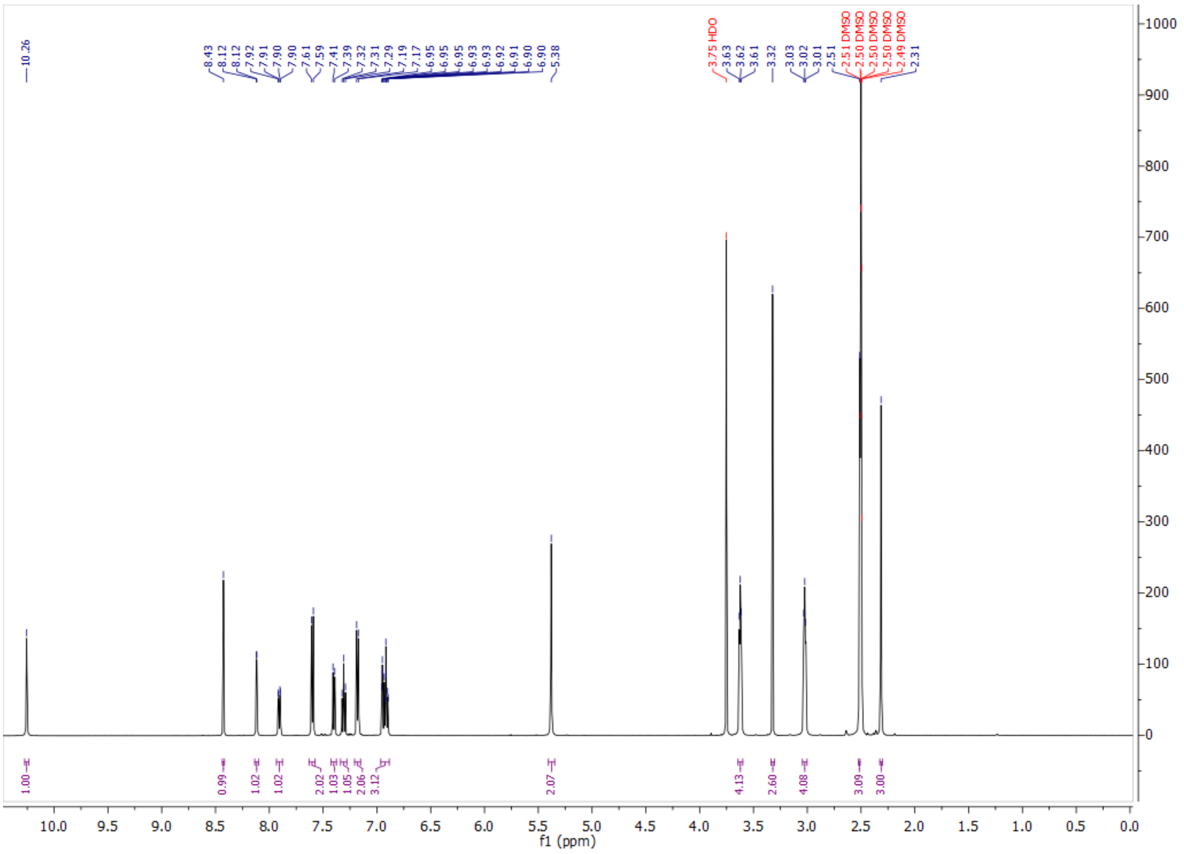

^{13}C NMR

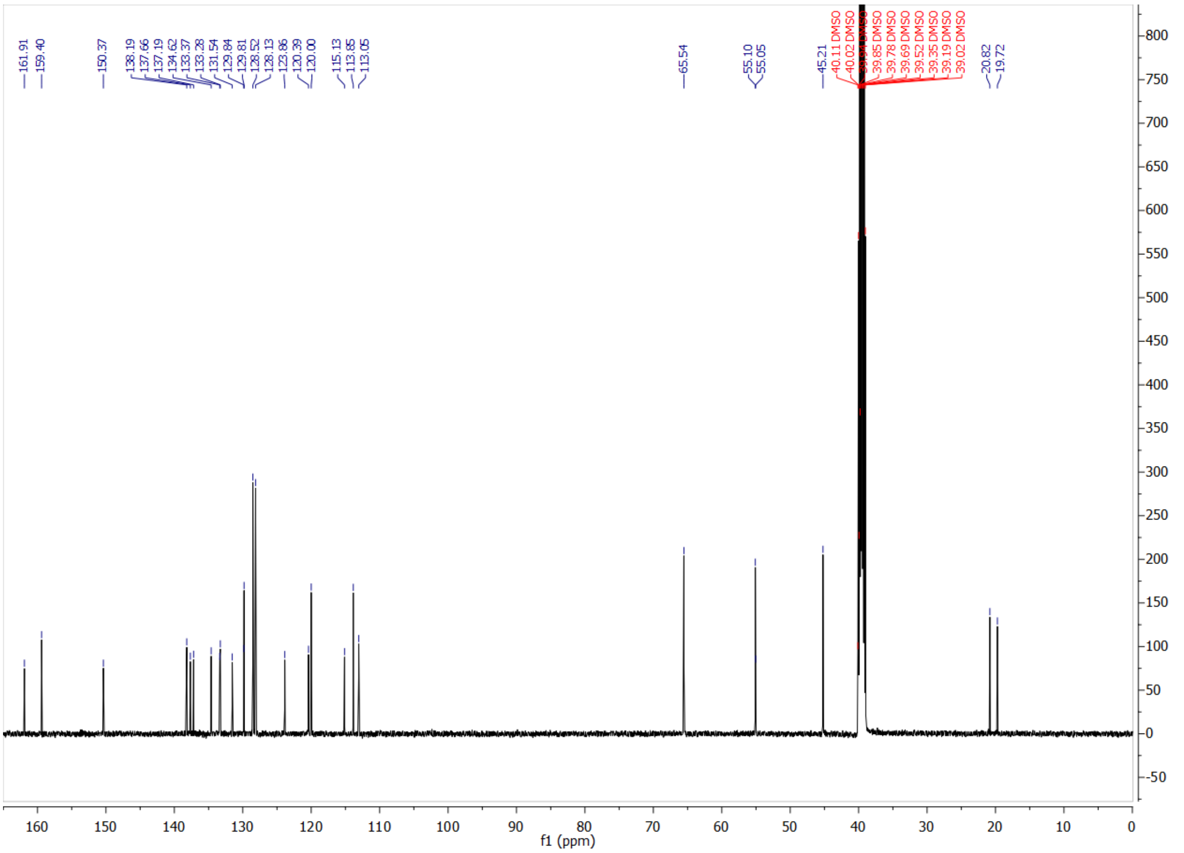

COMPOUND INFORMATION

Purity

Data File W:\analyti...OPEN\CGC_wave3_1_FirstPassB 2023-01-04 18-28-02\087-D2F-H11-DY268.D
Sample Name: DY268

```
=====================================================================
Acq. Operator   : SYSTEM                          Seq. Line :  87
Sample Operator : SYSTEM
Acq. Instrument : LCMS test                        Location :   D2F-H11
Injection Date  : 1/5/2023 10:21:51 AM                  Inj :   1
                                                Inj Volume : Inj prog
Sequence File   : W:\analytical_LCMS_DATA\EUbOPEN\CGC_wave3_1_FirstPassB 2023-01-04 18-28-02
                  \CGC_wave3_1_FirstPassB.S
Method          : W:\analytical_LCMS_DATA\EUbOPEN\CGC_wave3_1_FirstPassB 2023-01-04 18-28-02
                  \CGL_FIRSTPASS_GENERALMETHOD_VIAL1+2_20210319.M (Sequence Method)
Last changed    : 1/25/2022 4:36:18 PM by SYSTEM
Method Info     : CGL wellplate, 0.5 uL of 10 mM DMSO, general method
```

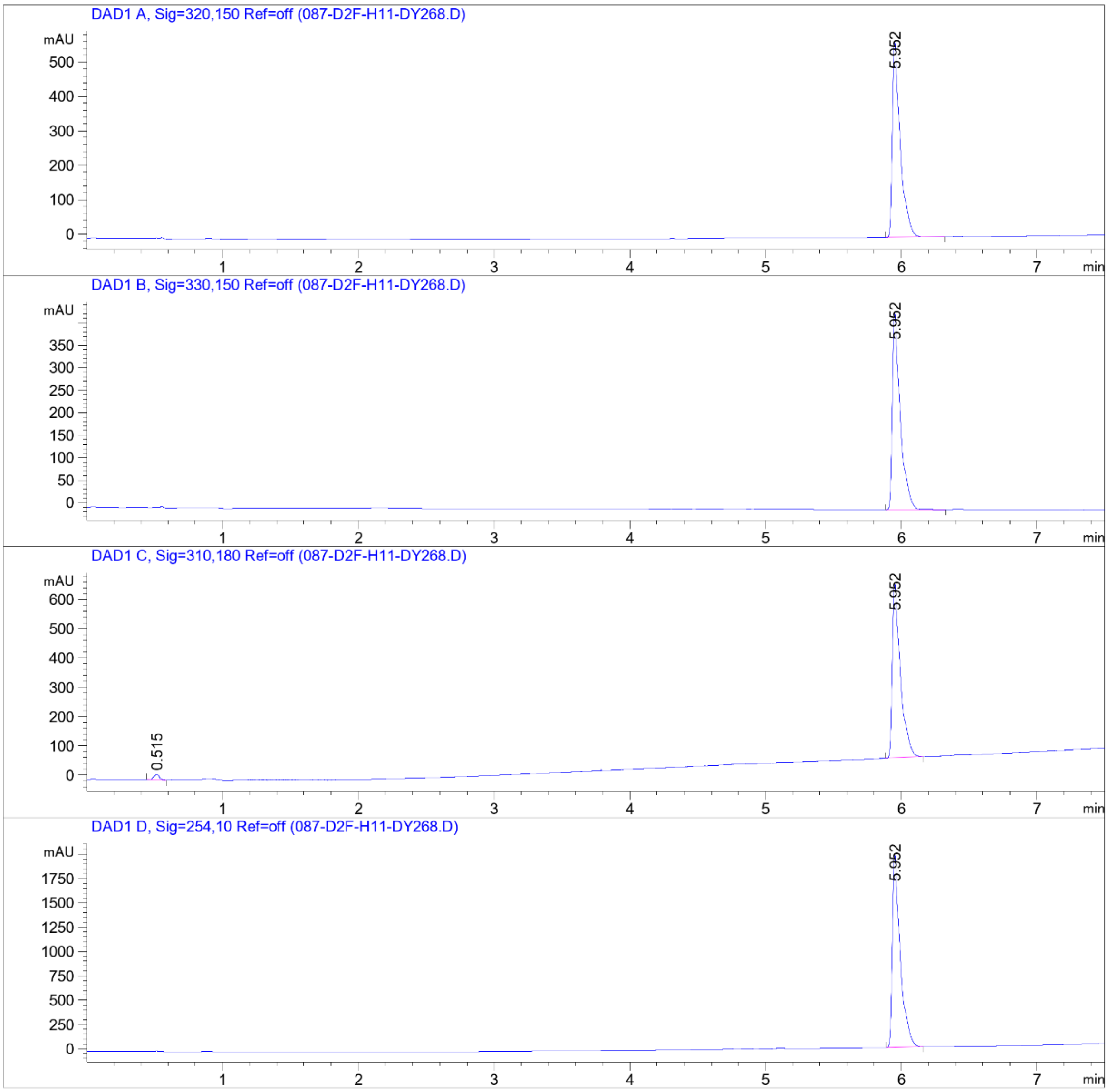

COMPOUND INFORMATION

Data File W:\analyti...OPEN\CGC_wave3_1_FirstPassB 2023-01-04 18-28-02\087-D2F-H11-DY268.D
Sample Name: DY268

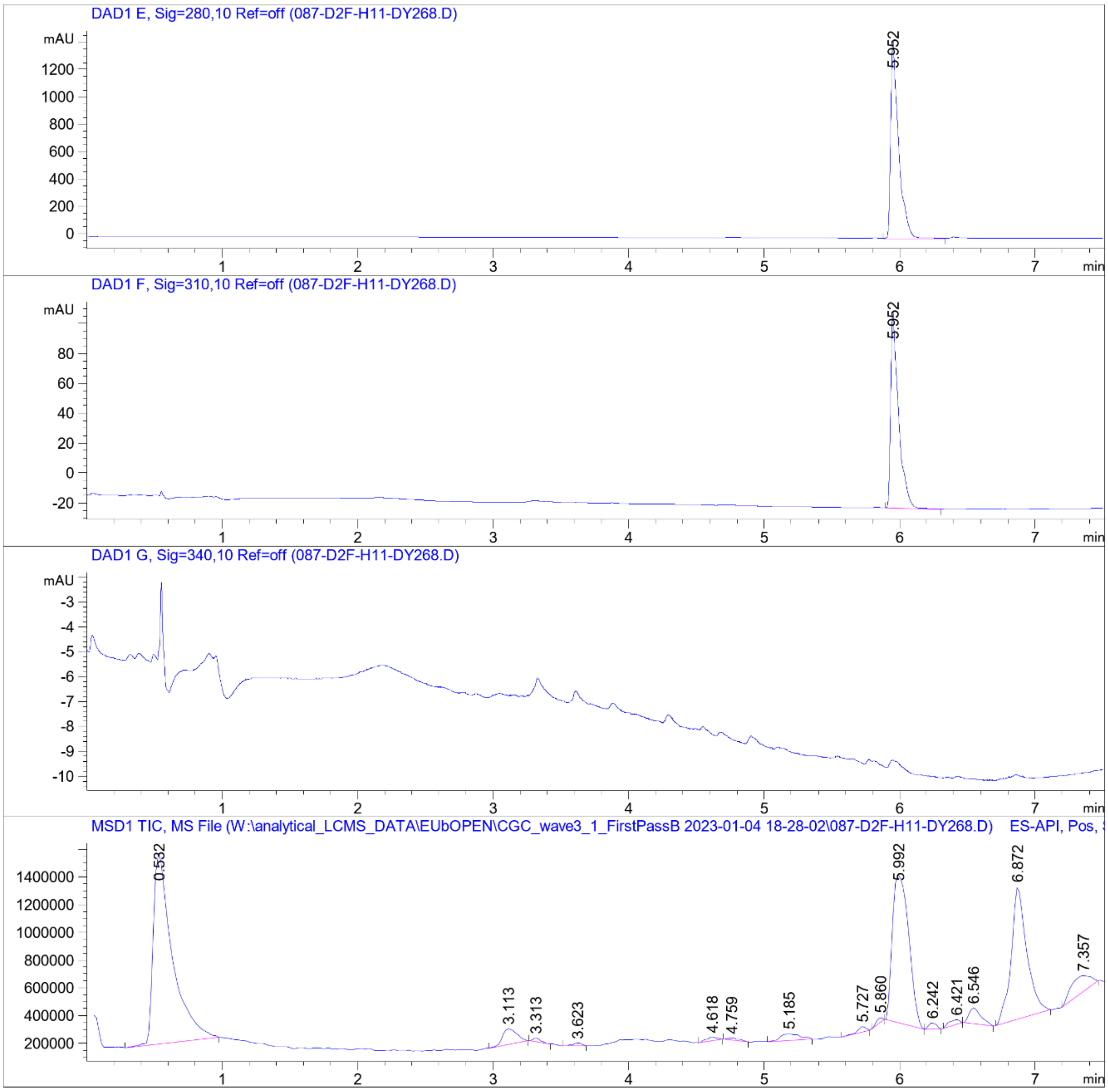

COMPOUND INFORMATION

Data File W:\analyti...OPEN\CGC_wave3_1_FirstPassB 2023-01-04 18-28-02\087-D2F-H11-DY268.D
Sample Name: DY268

MS Signal: MSD1 TIC, MS File, ES-API, Pos, Scan, Frag: 70, "POS Scan"
Spectra from peak tops.
Noise Cutoff: 1000 counts.
Reportable Ion Abundance: > 50%.
LC Signal: DAD1 A, Sig=320,150 Ref=off
Peak matching window: 0.1 min

Retention Time (LC)	LC Area	Retention Time (MS)	MS Area	Mol. Weight or Ion
-	-	0.532	13487662	157.10 I
-	-	3.113	821931	239.00 I
				217.10 I
-	-	3.313	102802	257.10 I
-	-	3.623	86049	199.90 I
				170.80 I
				159.00 I
				137.10 I
				111.10 I
-	-	4.618	149395	510.30 I
				170.90 I
				158.20 I
				137.10 I
-	-	4.759	104362	510.40 I
				279.00 I
				170.90 I
				137.10 I
-	-	5.185	496536	510.40 I
				338.20 I
				316.20 I
				298.20 I
				170.90 I
				137.10 I
				105.10 I
-	-	5.727	161107	280.20 I
-	-	5.860	107521	318.20 I
				296.20 I
5.952	2288	5.992	8951491	561.20 I
-	-	6.242	180650	228.10 I
				137.10 I
-	-	6.421	209454	350.20 I
				282.30 I
				254.20 I
				137.10 I
-	-	6.546	711251	507.20 I
				485.30 I
				280.20 I
-	-	6.872	7675830	282.20 I
-	-	7.357	1253355	400.30 I
				282.20 I

COMPOUND INFORMATION

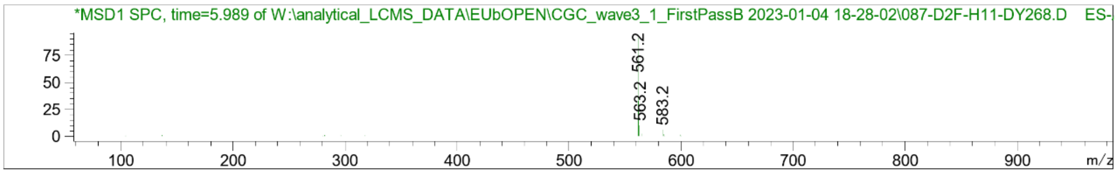

Biological activity

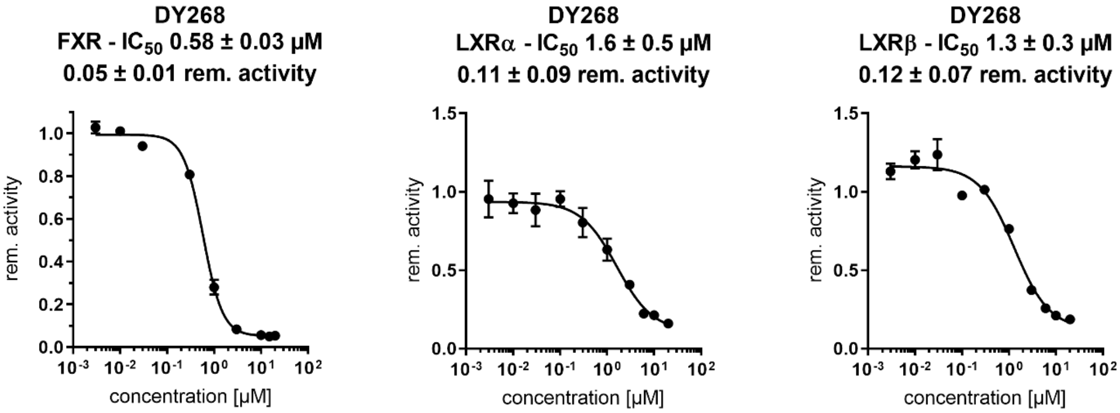

Supplement: Supplementary file 4 — Supplementary Data 1 [file 41467_2024_49493_MOESM4_ESM.zip › DY268.pdf]
