# Supplementary material for: Chemogenomics for NR1 nuclear hormone receptors
Source: Nat Commun. 2024 Jun 18;15:5201. doi: 10.1038/s41467-024-49493-6 (PMC11189487; doi:10.1038/s41467-024-49493-6)

COMPOUND INFORMATION

Elafibranor

**CAS Registry No.:**	923978-27-2
**Formal Name:**	(E)-2-(2,6-dimethyl-4-(3-(4-(methylthio)phenyl)-3-oxoprop-1-en-1-yl)phenoxy)-2-methylpropanoic acid
**EUbOPEN ID:**	EUB0001147a
**Molecular Formula:**	$C_{22}H_{24}O_4S$
**Molecular Weight:**	384.49 g/mol
**Smiles:**	CC1=CC(=CC(=C1OC(C)(C)C(=O)O)C)C=CC(=O)C2=CC=C(C=C2)SC
**Recommended concentration:**	1 µM

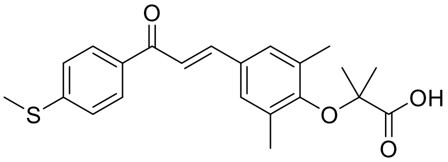

Biological activity

		Type	IC_{50}/EC_{50} [µM]	Reference
Main NR target:	NR1C1 (PPARα)	Agonist	0.13	inhouse
	NR1C2 (PPARδ)	Agonist	0.21	
	NR1C3 (PPARγ)	Agonist	0.41	
NR off-targets:				

COMPOUND INFORMATION

Identity

^{1}H NMR

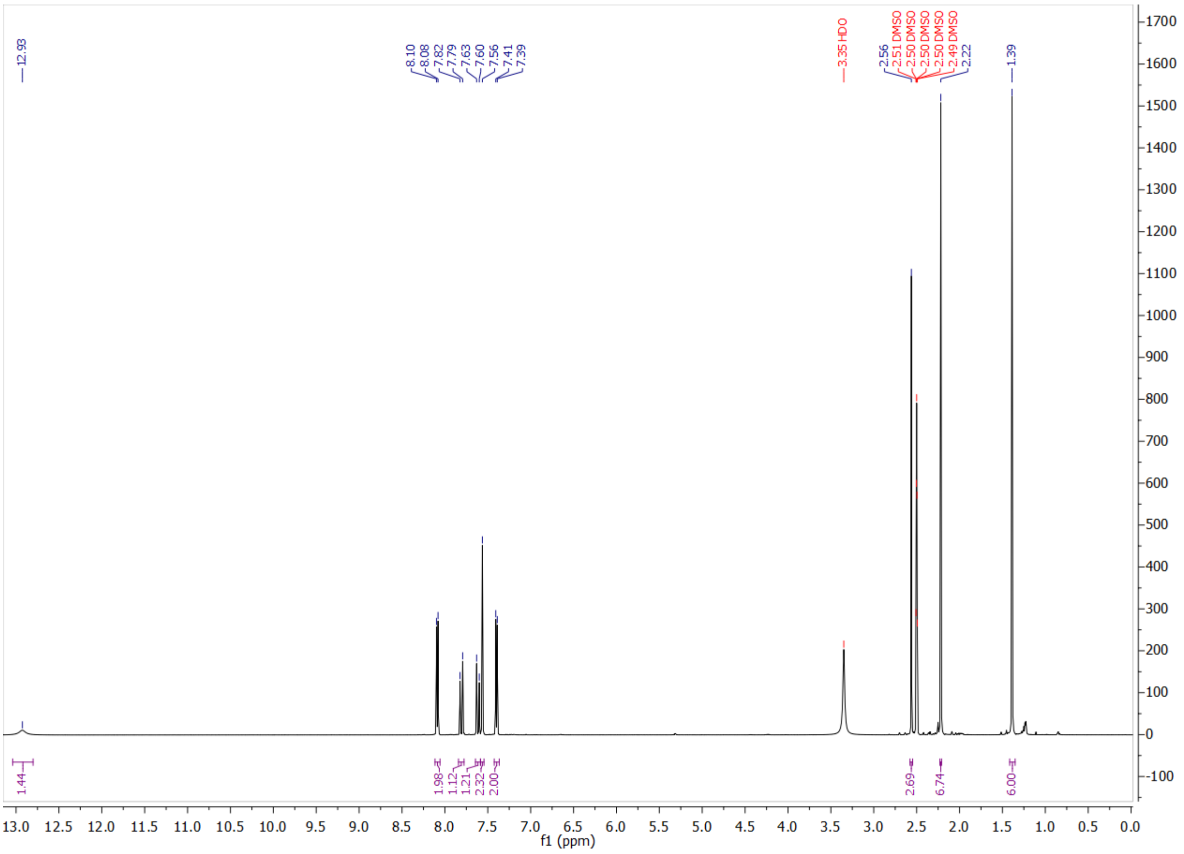

^{13}C NMR

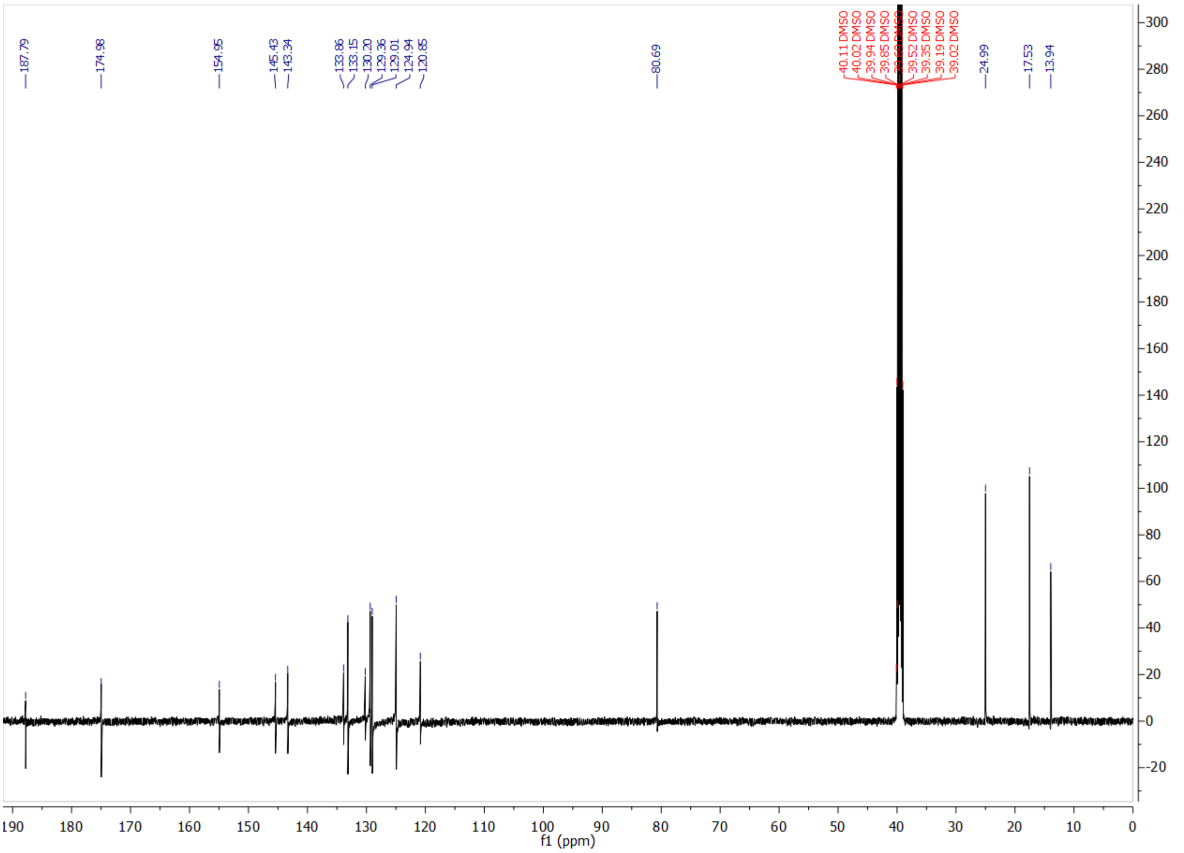

COMPOUND INFORMATION

EUbOPEN
Enabling & Unlocking Biology in the OPEN

Purity

```
Data File W:\analyti..._wave3_3rdRun_1stPassM 2023-05-05 17-35-57\014-D2F-B3-Elafibranor.D
Sample Name: Elafibranor

=====================================================================
Acq. Operator   : SYSTEM                        Seq. Line :  14
Sample Operator : SYSTEM
Acq. Instrument : LCMS test                      Location :   D2F-B3
Injection Date  : 5/5/2023 8:06:07 PM                 Inj :   1
                                               Inj Volume : Inj prog
Sequence File   : W:\analytical_LCMS_DATA\EUbOPEN\CGC_wave3_3rdRun_1stPassM 2023-05-05 17-35-
                  57\CGC_wave3_3rdRun_1stPassM.S
Method          : W:\analytical_LCMS_DATA\EUbOPEN\CGC_wave3_3rdRun_1stPassM 2023-05-05 17-35-
                  57\CGL_FIRSTPASS_GENERALMETHOD_VIAL1+2_20210319.M (Sequence Method)
Last changed    : 1/25/2022 4:36:18 PM by SYSTEM
Method Info     : CGL wellplate, 0.5 uL of 10 mM DMSO, general method
```

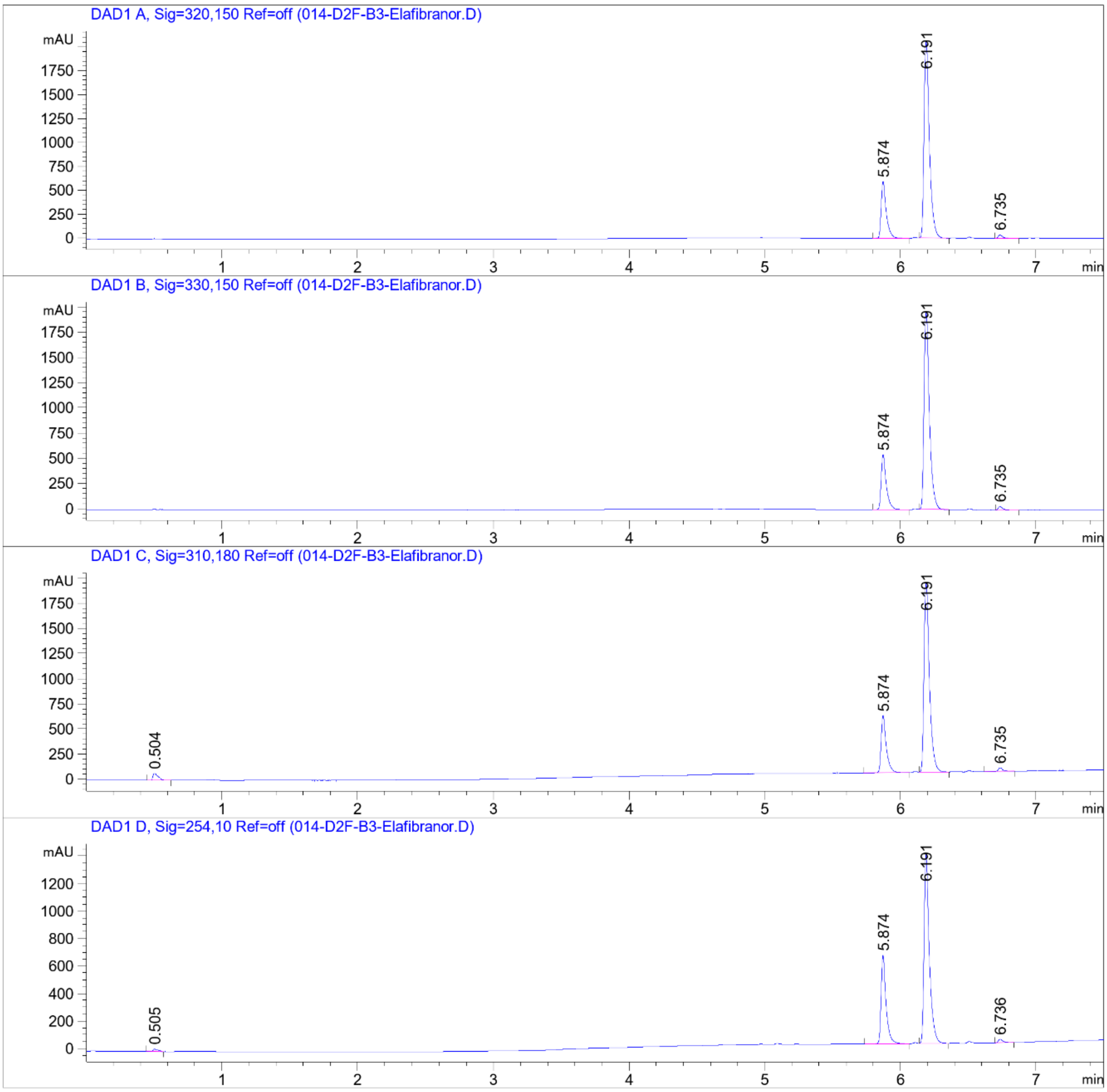

COMPOUND INFORMATION

Data File W:\analyti..._wave3_3rdRun_1stPassM 2023-05-05 17-35-57\014-D2F-B3-Elafibranor.D
Sample Name: Elafibranor

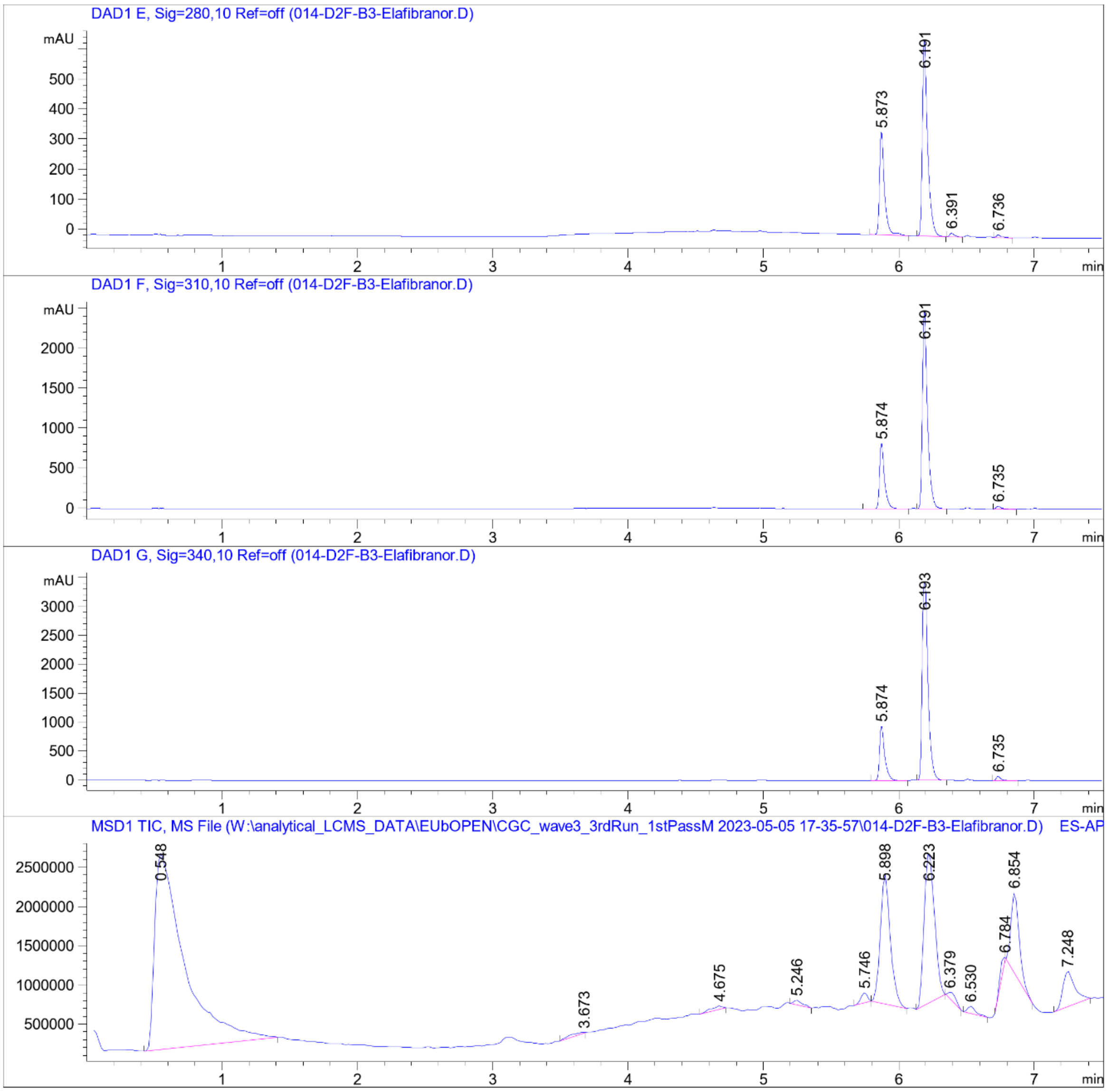

COMPOUND INFORMATION

Data File W:\analyti..._wave3_3rdRun_1stPassM 2023-05-05 17-35-57\014-D2F-B3-Elafibranor.D
Sample Name: Elafibranor

MS Signal: MSD1 TIC, MS File, ES-API, Pos, Scan, Frag: 70, "POS Scan"
Spectra from peak tops.
Noise Cutoff: 1000 counts.
Reportable Ion Abundance: > 50%.
LC Signal: DAD1 A, Sig=320,150 Ref=off
Peak matching window: 0.1 min

Retention Time (LC)	LC Area	Retention Time (MS)	MS Area	Mol. Weight or Ion
-	-	0.548	38118588	157.00 I
-	-	3.673	236383	232.60 I
-	-	4.675	290988	232.70 I
-	-	5.246	273169	232.60 I
-	-	5.746	437644	280.20 I<br>232.60 I
5.874	1535	5.898	8512759	385.10 I
6.191	5651	6.223	10189516	385.10 I
-	-	6.379	362086	254.20 I<br>232.60 I<br>172.10 I
-	-	6.530	357444	341.10 I<br>280.20 I<br>232.70 I<br>137.10 I
6.735	87	6.784	415121	256.20 I
-	-	6.854	4355882	282.20 I
-	-	7.248	2698250	284.20 I

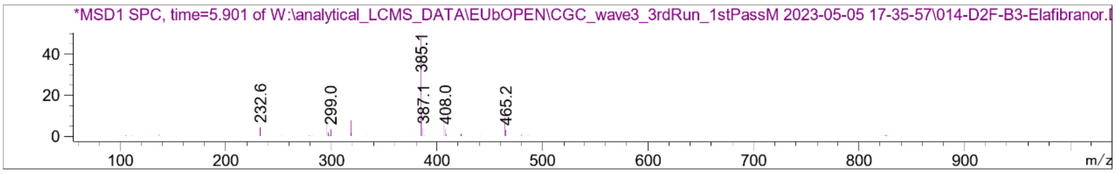

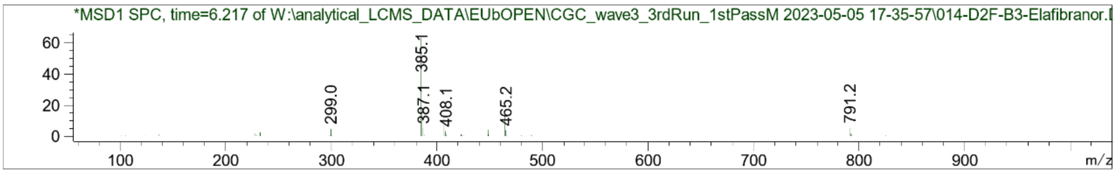

COMPOUND INFORMATION

Biological activity

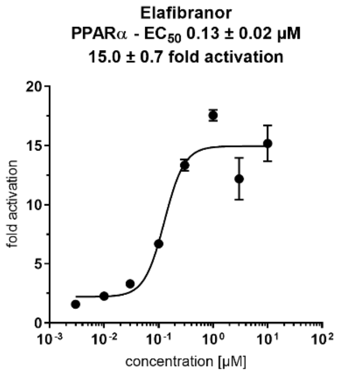

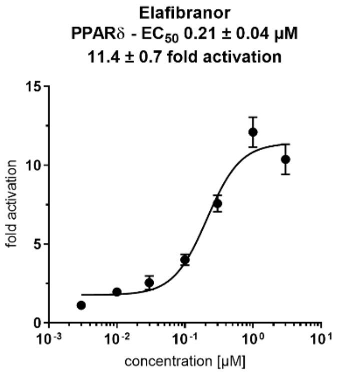

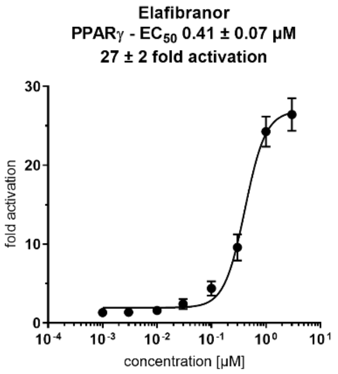

Supplement: Supplementary file 4 — Supplementary Data 1 [file 41467_2024_49493_MOESM4_ESM.zip › Elafibranor.pdf]
