# Supplementary material for: Chemogenomics for NR1 nuclear hormone receptors
Source: Nat Commun. 2024 Jun 18;15:5201. doi: 10.1038/s41467-024-49493-6 (PMC11189487; doi:10.1038/s41467-024-49493-6)

COMPOUND INFORMATION

Famprofazone

**CAS Registry No.:**	22881-35-2
**Formal Name:**	4-isopropyl-1-methyl-5-((methyl(1-phenylpropan-2-yl)amino)methyl)-2-phenyl-1,2-dihydro-3H-pyrazol-3-one
**EUbOPEN ID:**	EUB0002359a
**Molecular Formula:**	$C_{24}H_{31}N_3O$
**Molecular Weight:**	377.53 g/mol
**Smiles:**	CC(C)C1=C(N(N(C1=O)C2=CC=CC=C2)C)CN(C)C(C)CC3=CC=CC=C3
**Recommended concentration:**	1 µM

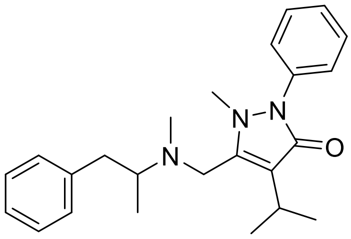

Biological activity

		Type	IC_{50}/EC_{50} [µM]	Reference
Main NR target:	NR1I2 (PXR)	Agonist	0.13	inhouse
NR off-target:				

COMPOUND INFORMATION

Identity

^{1}H NMR

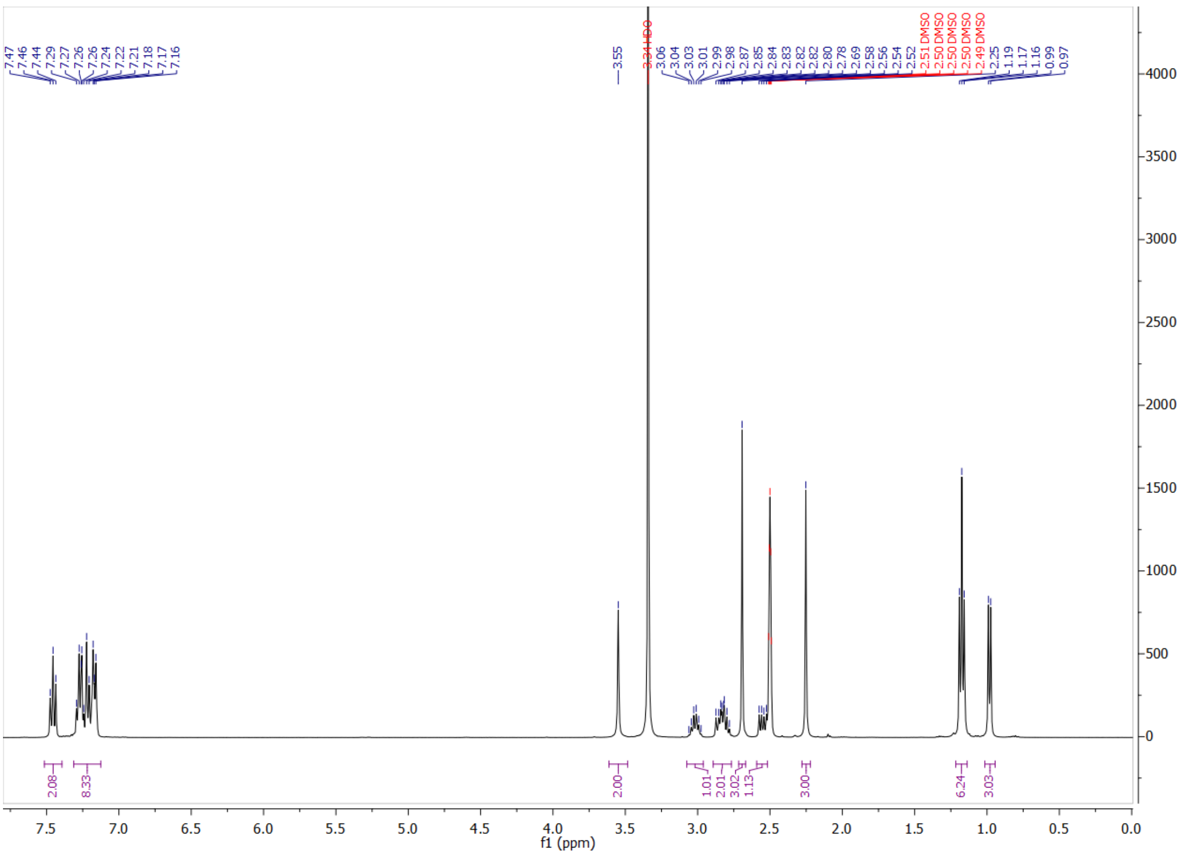

^{13}C NMR

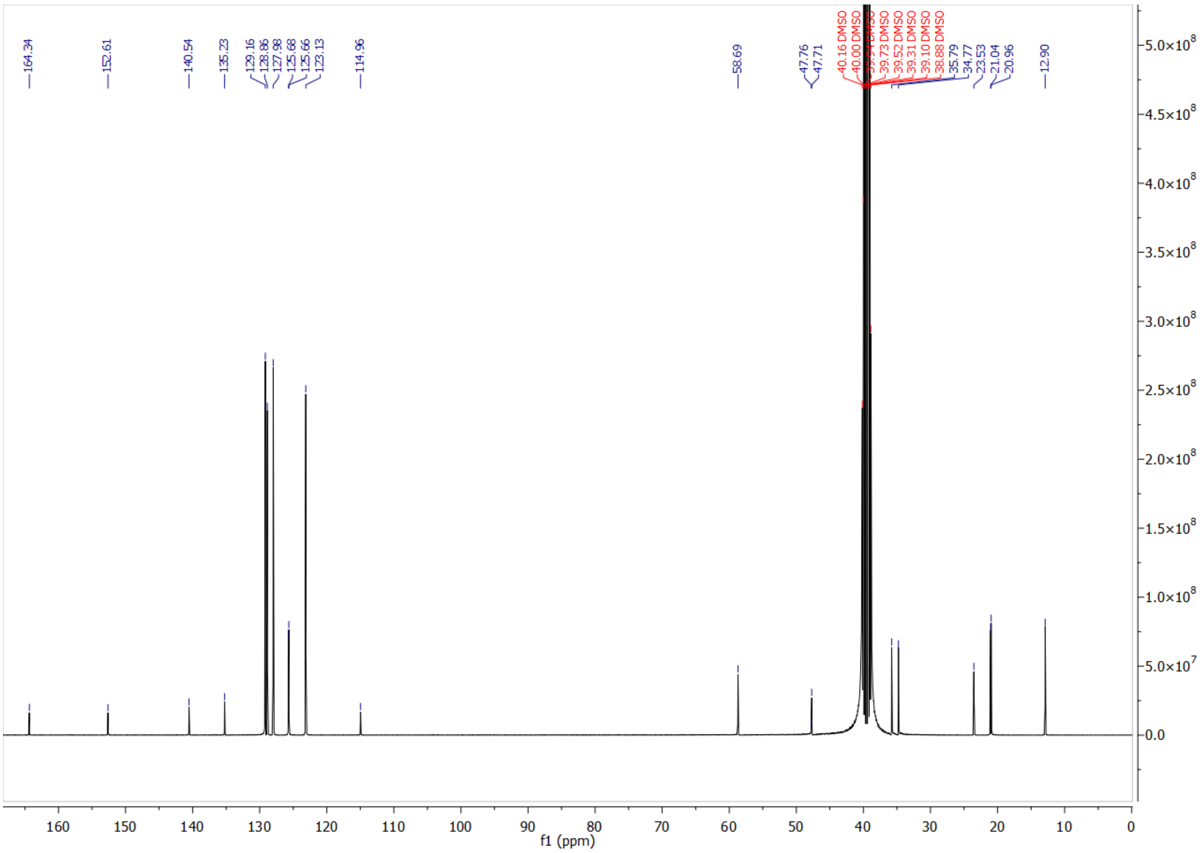

COMPOUND INFORMATION

Purity

**Data file:**	W:\analytical_LCMS_DATA\Sandra\Famprofazone (1) 2024-02-14 16-06-42.D
**Sample name:**	Famprofazone
**Description:**	

**Sample amount:**	0.000	**Sample type:**	Sample
**Instrument:**	LCMS test	**Location:**	D2F-D1
**Injection date:**	2024-02-14 16:09:22+01:00	**Injection:**	1 of 1
		**Injection volume:**	6.000

**Acq. method:**	FAST_Nonpolar_General_Method_MS-PosScan-100-1000.M
**Analysis method:**	SR_03_25_RP-0,5ml_7.5µL_GRAD - 7min_284_280 nm_INJPROG_low.M

**Module**	**Type**	**Part. No.**	**Serial No.**	**Firmware**
Agilent G6125B MSD	Detector	G6125B	<undef>	3.02.50
Quat. Pump	Pump	G7104C	DEAGZ01778	B.07.35 [0002]
Column Comp.	Column compartment	G7116A	DEAEM06531	D.07.35 [0002]
DAD	Detector	G7117C	DEAEK07274	D.07.35 [0002]
Multisampler	Auto sampler	G7167A	DEAGY01736	D.07.38 [0003]

Results

Sample Name Famprofazone

Chromatograms

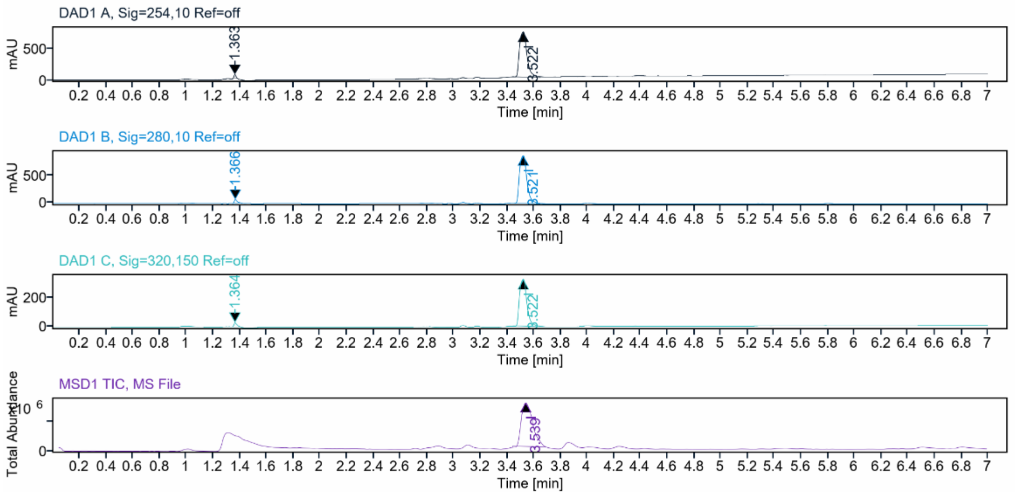

Sample Purity

Signal Description DAD1 A, Sig=254,10 Ref=off

Sample Name	Name	RT	Width	Area	Area%	Height
Famprofazone		1.363	0.016	62.6505	2.14	64.5794
Famprofazone		3.522	0.060	2868.1563	97.86	738.2081

Max Area% 97.862

UV Signal Purity>95% Pass

Signal Description DAD1 B, Sig=280,10 Ref=off

Sample Name	Name	RT	Width	Area	Area%	Height
Famprofazone		1.366	0.021	90.9742	2.65	63.8089
Famprofazone		3.521	0.059	3347.0217	97.35	875.5484

Max Area% 97.354

UV Signal Purity>95% Pass

COMPOUND INFORMATION

Signal Description DAD1 C, Sig=320,150 Ref=off

Sample Name	Name	RT	Width	Area	Area%	Height
Famprofazone		1.364	0.022	40.3872	3.12	29.0652
Famprofazone		3.522	0.060	1252.8397	96.88	329.1352

Max Area% 96.877

UV Signal Purity>95% Pass

UV Apex Spectra

RT:

3.522

Sample Name:

Famprofazone

Signal Name:

DAD1A

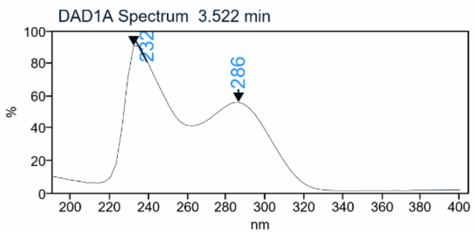

MSD Apex Spectra

RT:

3.539

Sample Name:

Famprofazone

Signal Name:

MSD1TIC

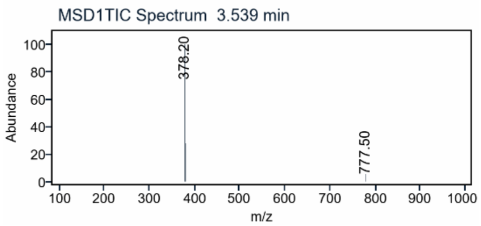

Biological activity

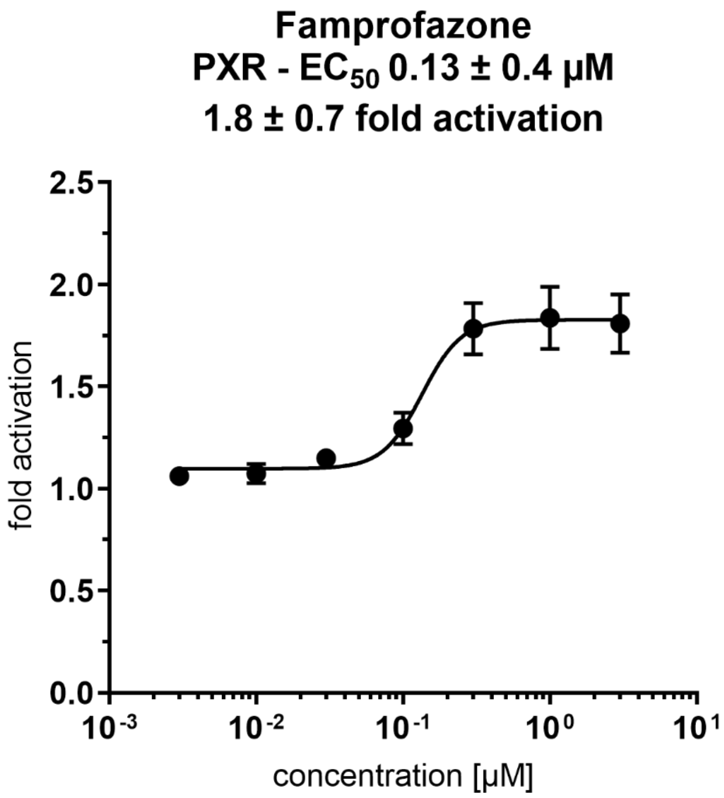

Supplement: Supplementary file 4 — Supplementary Data 1 [file 41467_2024_49493_MOESM4_ESM.zip › Famprofazone.pdf]
