# Supplementary material for: Chemogenomics for NR1 nuclear hormone receptors
Source: Nat Commun. 2024 Jun 18;15:5201. doi: 10.1038/s41467-024-49493-6 (PMC11189487; doi:10.1038/s41467-024-49493-6)

COMPOUND INFORMATION

Fexaramine

**CAS Registry No.:**	574013-66-4
**Formal Name:**	Methyl (E)-3-(3-(N-((4'-(dimethylamino)-[1,1'-biphenyl]-4-yl)methyl)cyclohexanecarboxamido)phenyl)acrylate
**EUBOPEN ID:**	EUB0000182b
**Molecular Formula:**	$C_{32}H_{36}N_2O_3$
**Molecular Weight:**	496.65 g/mol
**Smiles:**	CN(C1=CC=C(C2=CC=C(CN(C(C3CCCCC3)=O)C4=CC=CC(/C=C/C(OC)=O)=C4)C=C2)C=C1)C
**Recommended concentration:**	1 µM

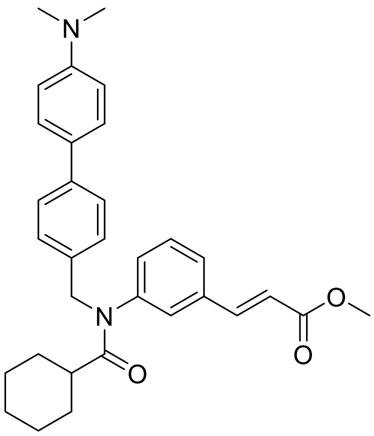

Biological activity

		Type	IC_{50}/EC_{50} [µM]	Reference
Main NR target:	NR1H4 (FXR)	Agonist	0.96	inhouse
NR off-target:				

COMPOUND INFORMATION

Identity

^{1}H NMR

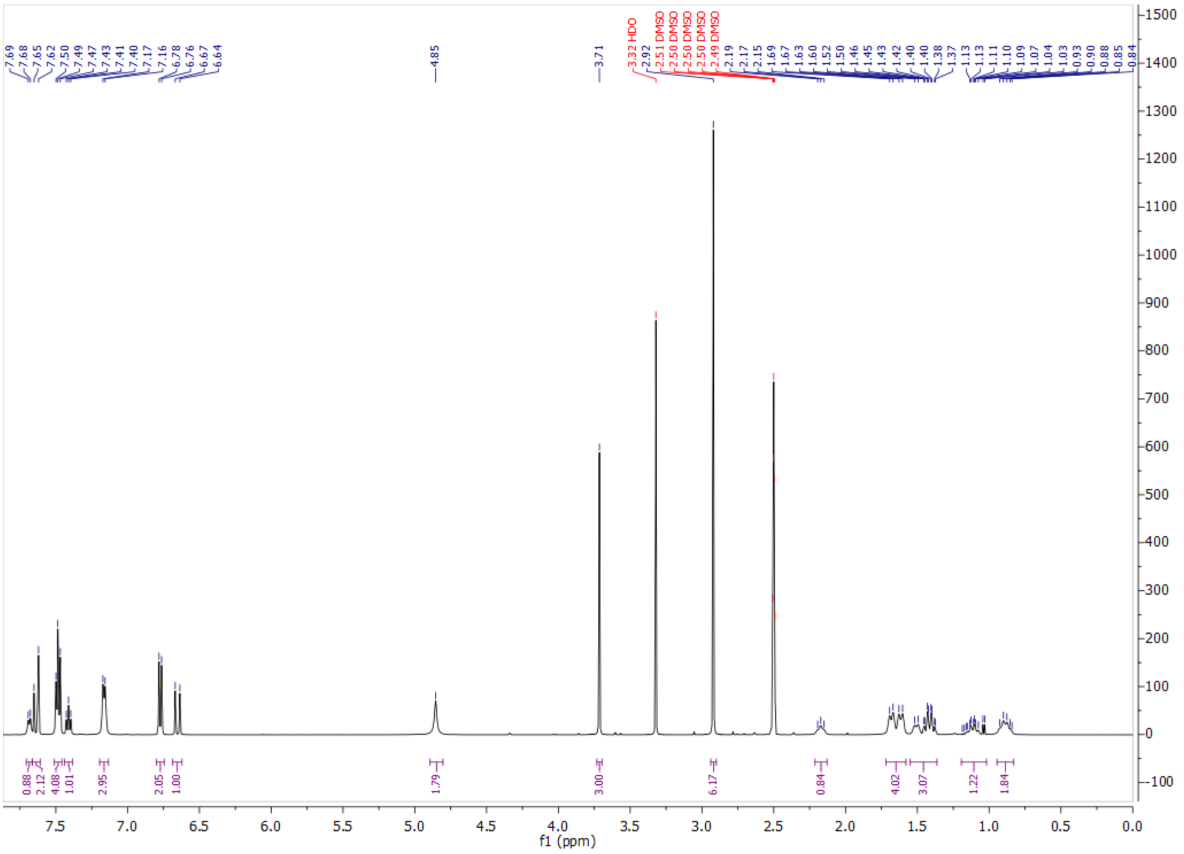

^{13}C NMR

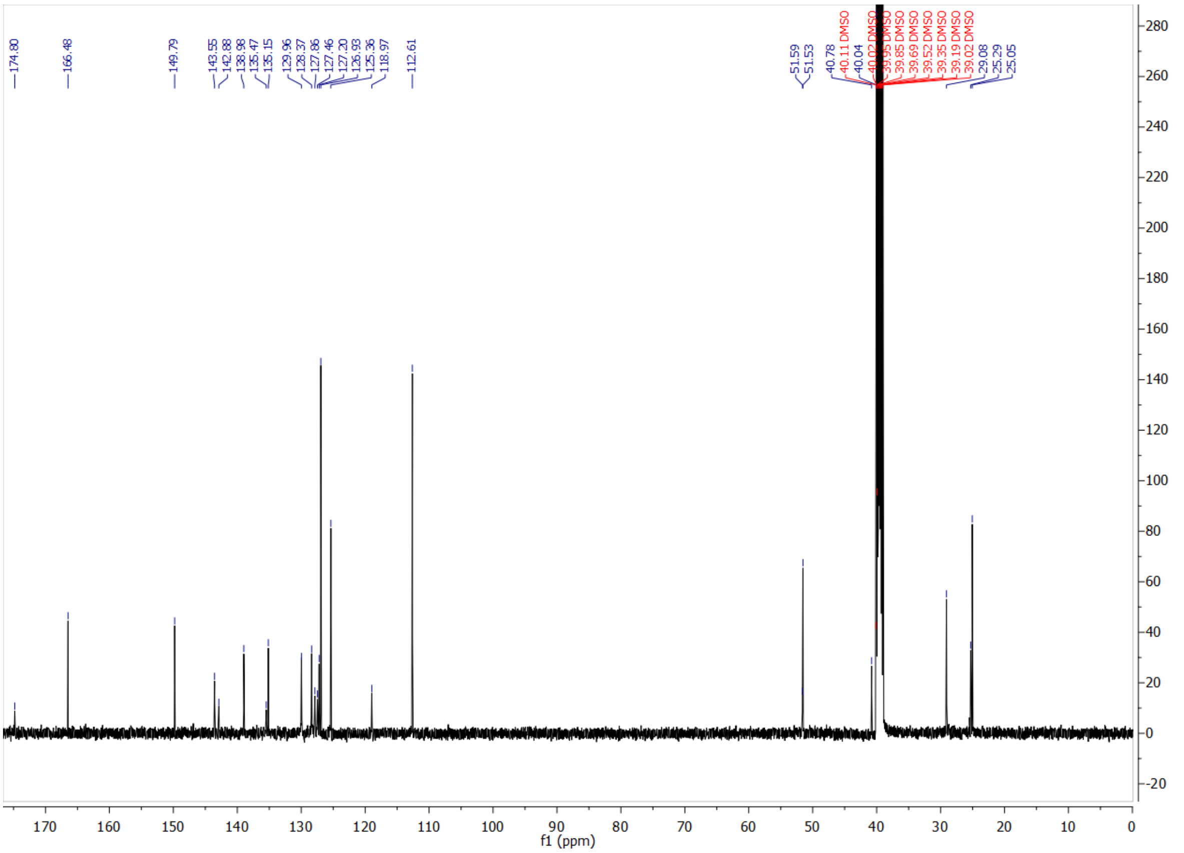

COMPOUND INFORMATION

EUbOPEN
Enabling & Unlocking Biology in the OPEN

Purity

```
Data File W:\analyti...N\CGC_ECHO1-3_FirstPass 2021-03-20 13-21-54\083-D2B-G8-fexaramine.D
Sample Name: fexaramine

    =====================================================================
    Acq. Operator   : SYSTEM                        Seq. Line :  83
    Sample Operator : SYSTEM
    Acq. Instrument : LCMS test                      Location :  D2B-G8
    Injection Date  : 3/21/2021 4:34:23 AM                Inj :  1
                                                Inj Volume : Inj prog
    Sequence File   : W:\analytical_LCMS_DATA\EUbOPEN\CGC_ECHO1-3_FirstPass 2021-03-20 13-21-54
                      \CGC_ECHO1-3_FirstPass.S
    Method          : W:\analytical_LCMS_DATA\EUbOPEN\CGC_ECHO1-3_FirstPass 2021-03-20 13-21-54
                      \CGL_FIRSTPASS_GENERALMETHOD_VIAL3+4_20210319.M (Sequence Method)
    Last changed    : 3/19/2021 5:35:24 PM by SYSTEM
    Method Info     : CGL wellplate, 0.5 uL of 10 mM DMSO, general method
```

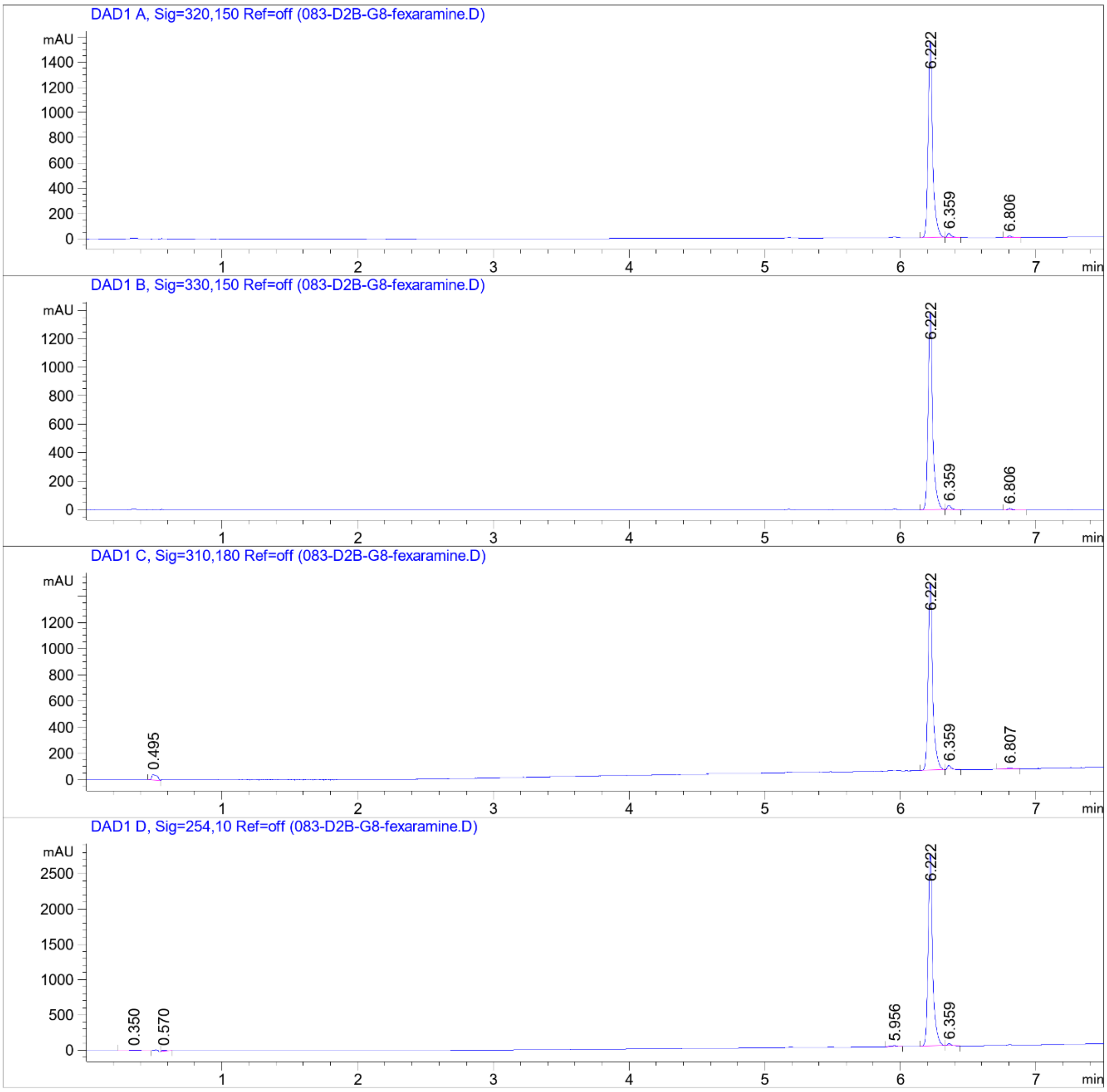

COMPOUND INFORMATION

Data File W:\analyti...N\CGC_ECHO1-3_FirstPass 2021-03-20 13-21-54\083-D2B-G8-fexaramine.D
Sample Name: fexaramine

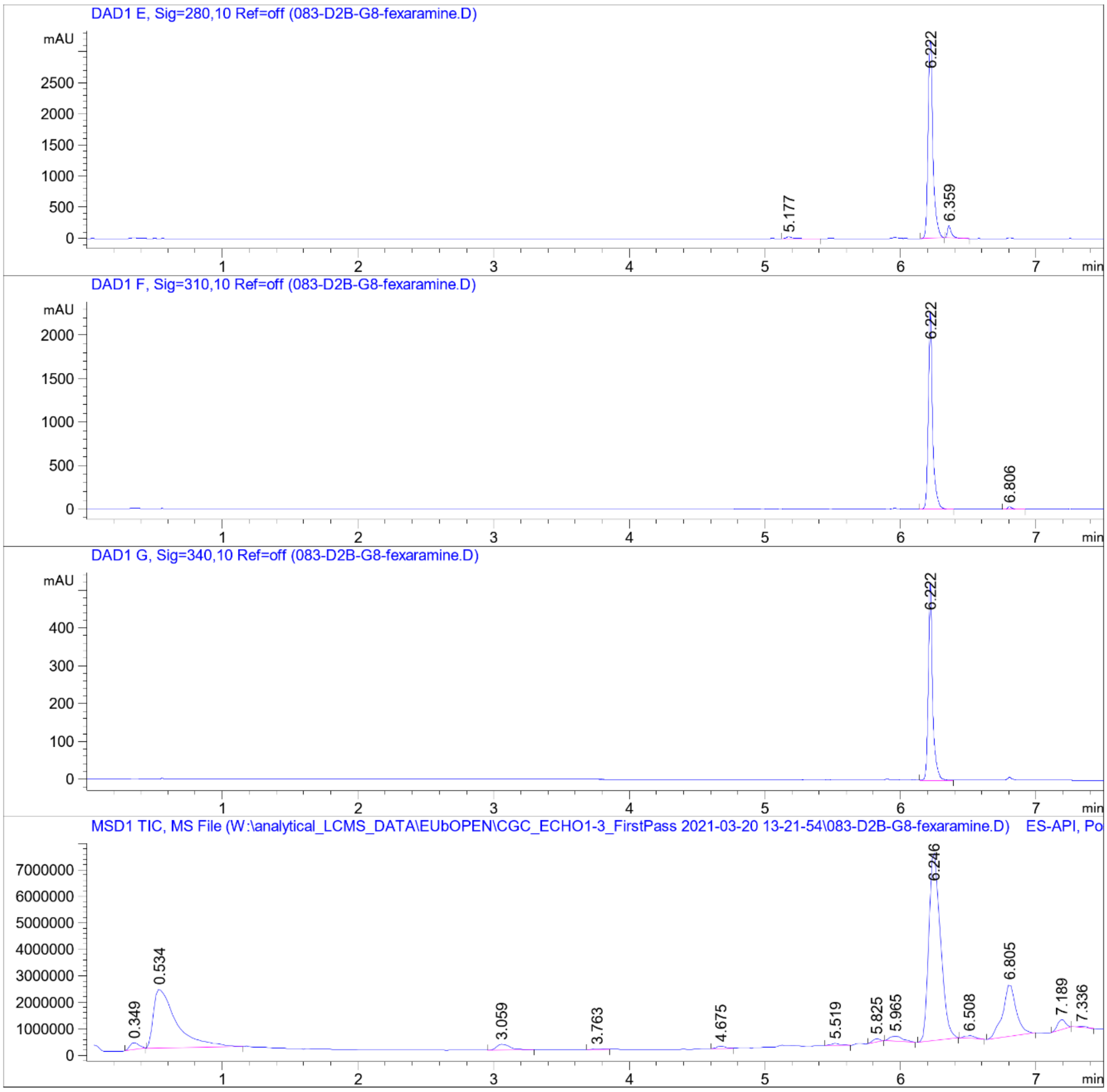

COMPOUND INFORMATION

Data File W:\analyti...N\CGC_ECHO1-3_FirstPass 2021-03-20 13-21-54\083-D2B-G8-fexaramine.D
Sample Name: fexaramine

MS Signal: MSD1 TIC, MS File, ES-API, Pos, Scan, Frag: 70, "POS Scan"
Spectra from peak tops.
Noise Cutoff: 1000 counts.
Reportable Ion Abundance: > 50%.
LC Signal: DAD1 A, Sig=320,150 Ref=off
Peak matching window: 0.1 min

Retention Time (LC)	LC Area	Retention Time (MS)	MS Area	Mol. Weight or Ion
-	-	0.349	1175882	182.90 I<br>158.00 I
-	-	0.534	26096508	157.00 I
-	-	3.059	1444823	217.10 I
-	-	3.763	181934	274.20 I
-	-	4.675	412686	326.30 I
-	-	5.519	379627	469.20 I
-	-	5.825	494745	296.20 I
-	-	5.965	1336074	483.30 I
6.222	3603	6.246	42136496	497.30 I
6.359	61	-	-	
-	-	6.508	583749	485.30 I<br>280.20 I
6.806	26	6.805	13365873	282.20 I
-	-	7.189	1514970	284.30 I<br>282.20 I
-	-	7.336	232825	400.30 I<br>282.20 I

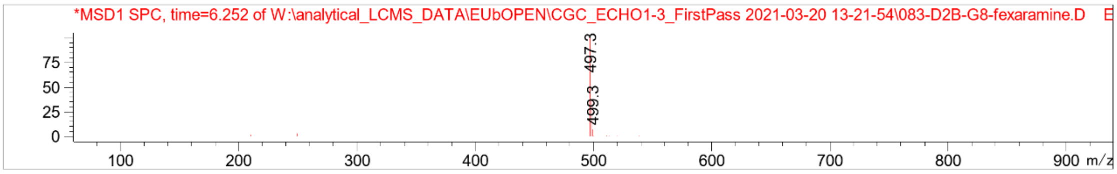

COMPOUND INFORMATION

Biological activity

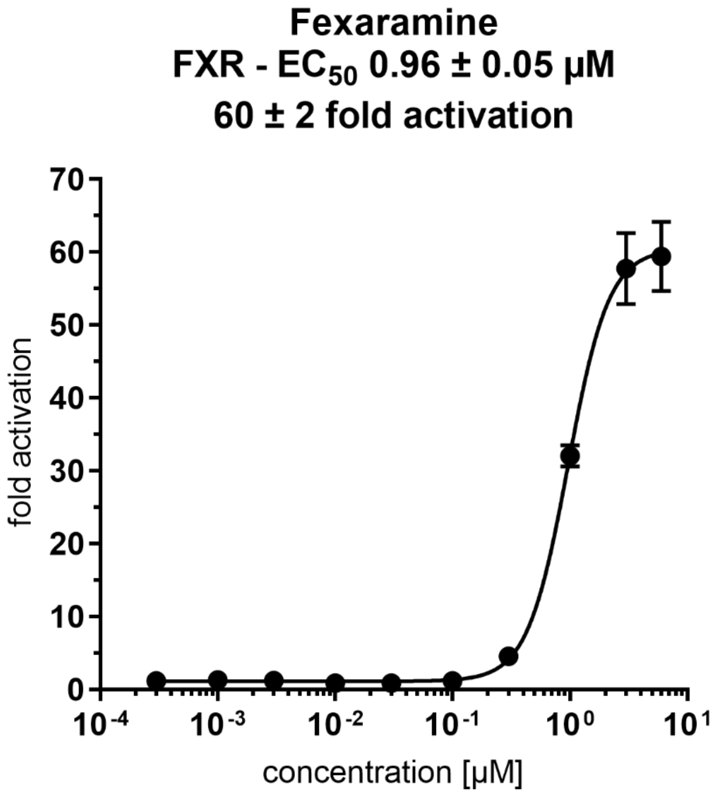

Supplement: Supplementary file 4 — Supplementary Data 1 [file 41467_2024_49493_MOESM4_ESM.zip › Fexaramine.pdf]
