# Supplementary material for: Chemogenomics for NR1 nuclear hormone receptors
Source: Nat Commun. 2024 Jun 18;15:5201. doi: 10.1038/s41467-024-49493-6 (PMC11189487; doi:10.1038/s41467-024-49493-6)

COMPOUND INFORMATION

GSK0660

**CAS Registry No.:**	1014691-61-2
**Formal Name:**	Methyl 3-(N-(2-methoxy-4-(phenylamino)phenyl)sulfamoyl)thiophene-2-carboxylate
**EUbOPEN ID:**	EUB0001150a
**Molecular Formula:**	$C_{19}H_{18}N_2O_5S_2$
**Molecular Weight:**	418.48 g/mol
**Smiles:**	COC1=C(C=CC(=C1)NC2=CC=CC=C2)NS(=O)(=O)C3=C(SC=C3)C(=O)OC
**Recommended concentration:**	10 µM

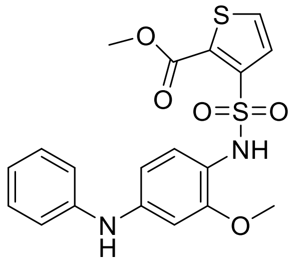

Biological activity

		Type	IC50/EC50 [µM]	Reference
Main NR target:	NR1C1 (PPARα)	Antagonist	6.1	inhouse
	NR1C2 (PPARδ)	Antagonist	5.5	
	NR1C3 (PPARγ)	Antagonist	7.6	
NR off-target:				

COMPOUND INFORMATION

Identity

^{1}H NMR

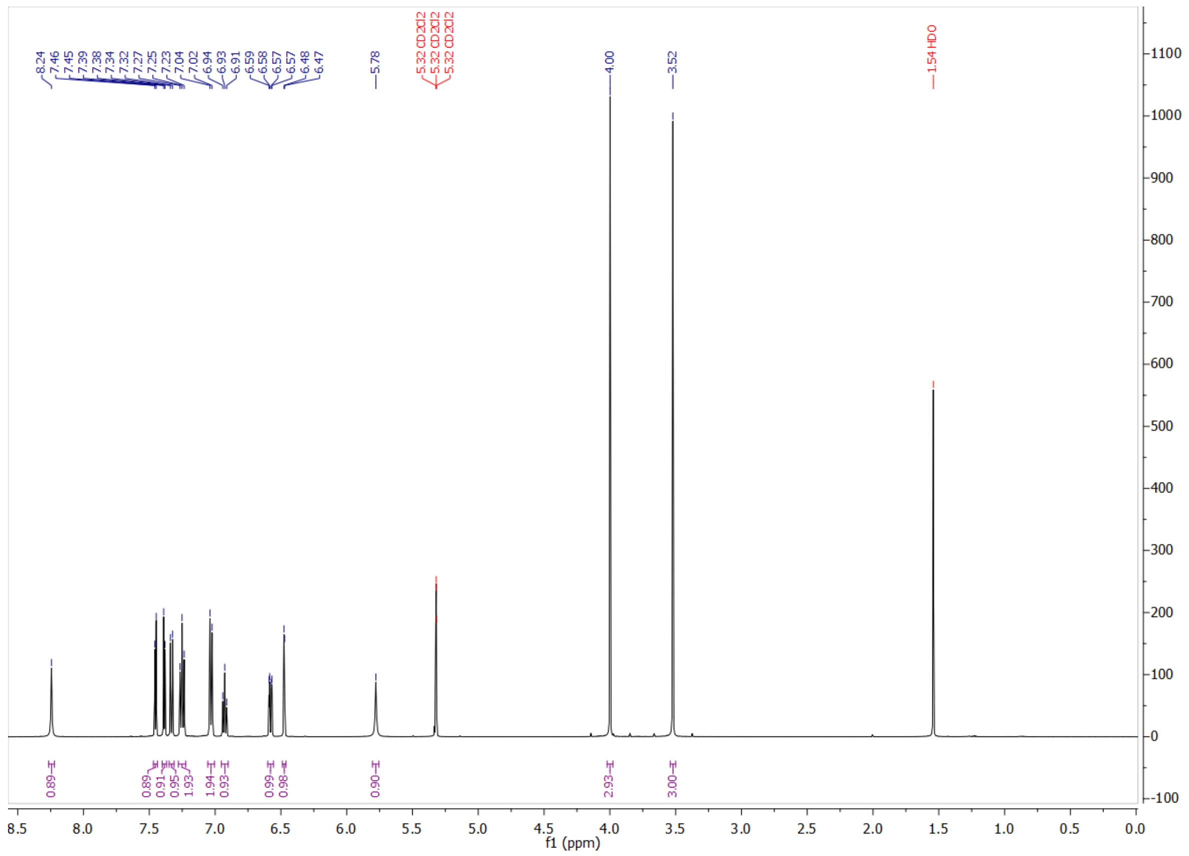

^{13}C NMR

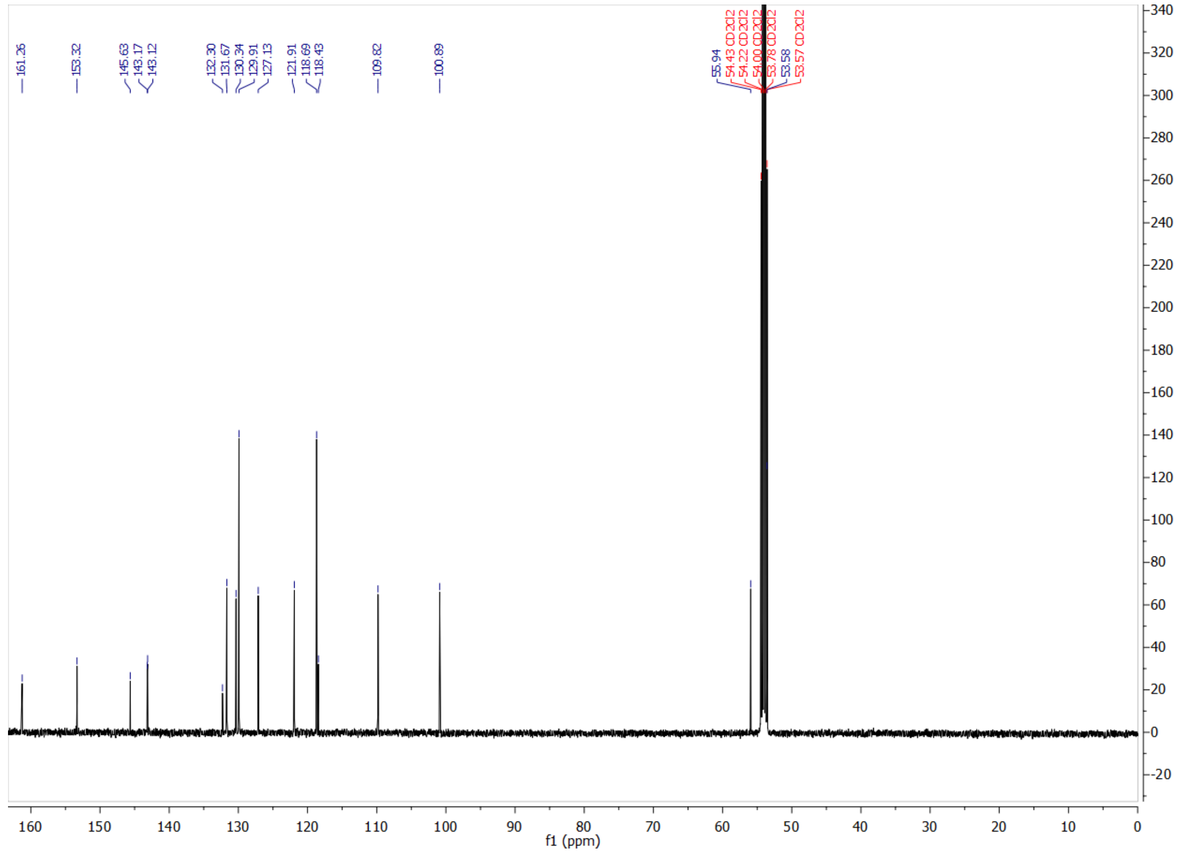

COMPOUND INFORMATION

Purity

```
Data File W:\analyti...PEN\CGC_wave3_1_FirstPassB 2023-01-04 18-28-02\071-D2F-F8-GSK0660.D
Sample Name: GSK0660

    =====================================================================
    Acq. Operator   : SYSTEM                        Seq. Line :   71
    Sample Operator : SYSTEM
    Acq. Instrument : LCMS test                      Location :   D2F-F8
    Injection Date  : 1/5/2023 7:24:53 AM                 Inj :   1
                                               Inj Volume : Inj prog
    Sequence File   : W:\analytical_LCMS_DATA\EUbOPEN\CGC_wave3_1_FirstPassB 2023-01-04 18-28-02
                      \CGC_wave3_1_FirstPassB.S
    Method          : W:\analytical_LCMS_DATA\EUbOPEN\CGC_wave3_1_FirstPassB 2023-01-04 18-28-02
                      \CGL_FIRSTPASS_GENERALMETHOD_VIAL1+2_20210319.M (Sequence Method)
    Last changed    : 1/25/2022 4:36:18 PM by SYSTEM
    Method Info     : CGL wellplate, 0.5 uL of 10 mM DMSO, general method
```

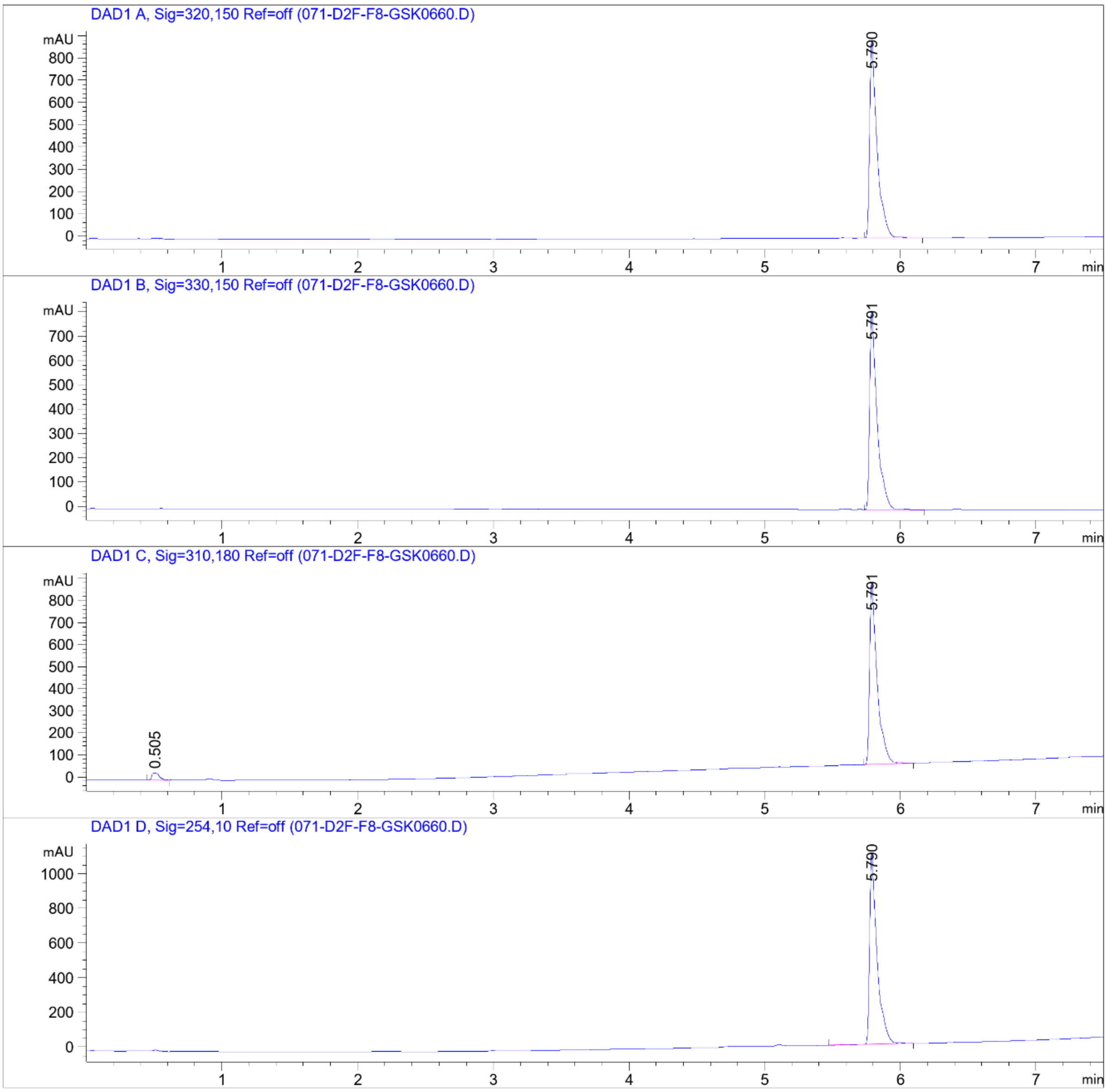

COMPOUND INFORMATION

Data File W:\analyti...PEN\CGC_wave3_1_FirstPassB 2023-01-04 18-28-02\071-D2F-F8-GSK0660.D
Sample Name: GSK0660

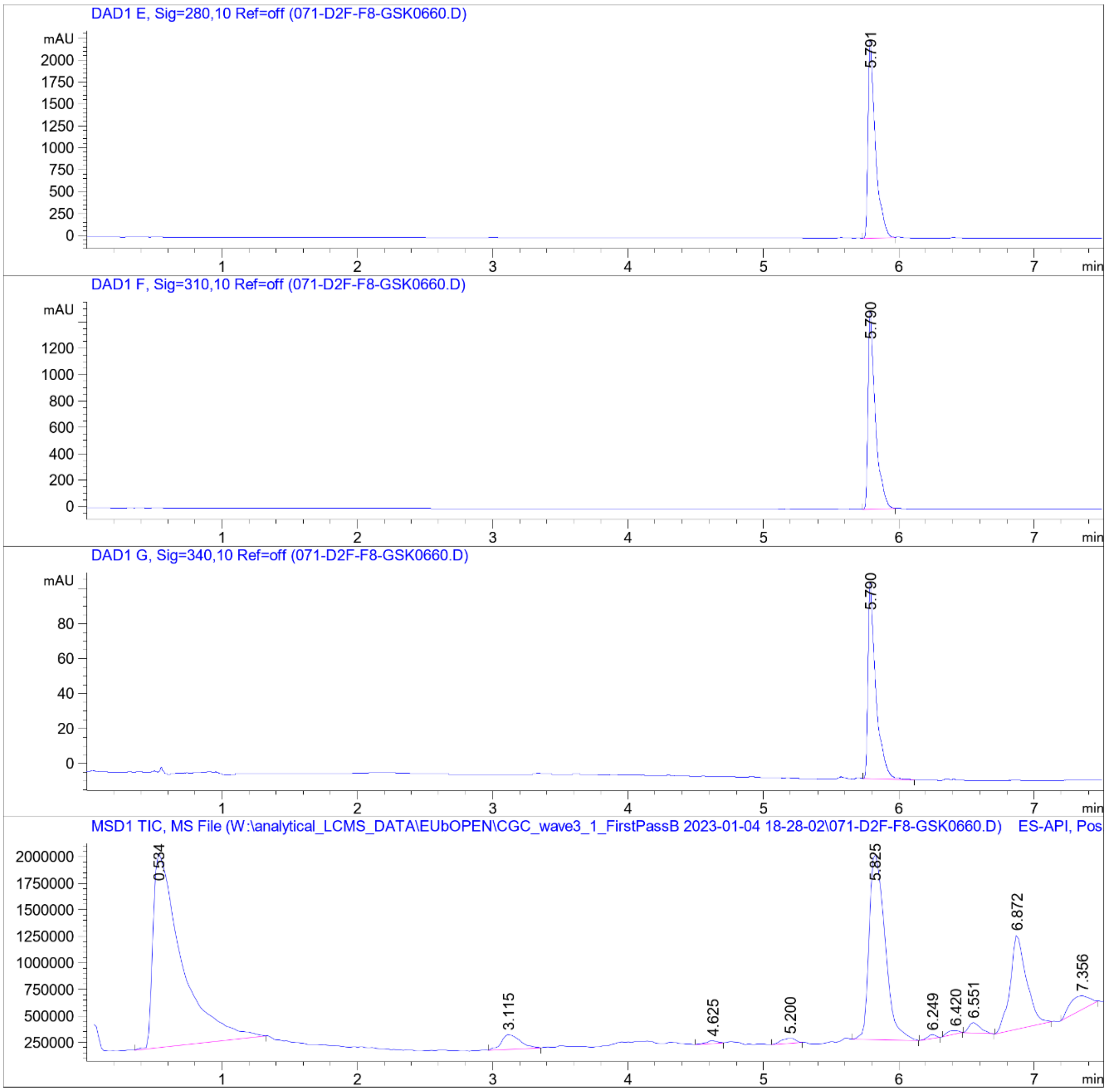

COMPOUND INFORMATION

Data File W:\analyti...PEN\CGC_wave3_1_FirstPassB 2023-01-04 18-28-02\071-D2F-F8-GSK0660.D
Sample Name: GSK0660

```
    MS Signal: MSD1 TIC, MS File,  ES-API, Pos, Scan, Frag: 70, "POS Scan"
        Spectra from peak tops.
        Noise Cutoff: 1000 counts.
        Reportable Ion Abundance: > 50%.
    LC Signal: DAD1 A, Sig=320,150 Ref=off
        Peak matching window: 0.1  min
```

Retention Time (LC)	LC Area	Retention Time (MS)	MS Area	Mol. Weight or Ion
-	-	0.534	26793372	157.00 I
-	-	3.115	1309027	239.10 I
				217.10 I
-	-	4.625	145648	510.40 I
				170.80 I
				158.10 I
-	-	5.200	366719	510.40 I
				170.80 I
				137.10 I
5.790	3372	5.825	14282155	419.00 I
				214.10 I
-	-	6.249	154232	228.20 I
				137.10 I
-	-	6.420	214934	350.30 I
				282.30 I
				254.20 I
-	-	6.551	568204	507.30 I
				280.20 I
-	-	6.872	7399131	282.30 I
-	-	7.356	1385209	400.30 I
				282.30 I

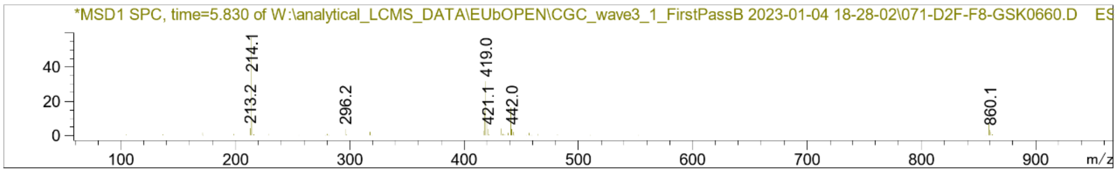

Biological activity

GSK0660
PPARα - IC_{50} 6.1 ± 0.6 µM
0.0 ± 0.1 rem. activity

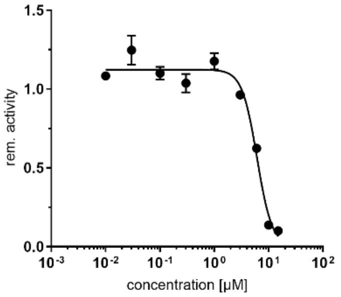

GSK0660
PPARδ - IC_{50} 5.5 ± 0.7 µM
0.0 ± 0.1 rem. activity

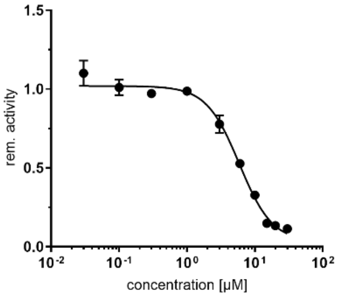

GSK0660
PPARγ - IC_{50} 7.6 ± 0.3 µM
0.02 ± 0.03 rem. activity

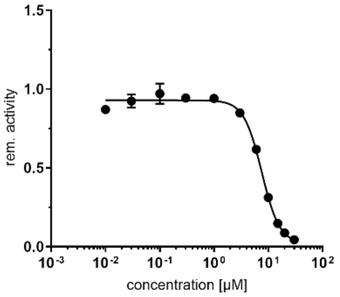

Supplement: Supplementary file 4 — Supplementary Data 1 [file 41467_2024_49493_MOESM4_ESM.zip › GSK0660.pdf]
