# Supplementary material for: Chemogenomics for NR1 nuclear hormone receptors
Source: Nat Commun. 2024 Jun 18;15:5201. doi: 10.1038/s41467-024-49493-6 (PMC11189487; doi:10.1038/s41467-024-49493-6)

COMPOUND INFORMATION

GSK2033

**CAS Registry No.:**	1221277-90-2
**Formal Name:**	2,4,6-trimethyl-N-((3'-(methylsulfonyl)-[1,1'-biphenyl]-4-yl)methyl)-N-((5-(trifluoromethyl)furan-2-yl)methyl)benzenesulfonamide
**EUBOPEN ID:**	EUB0000183b
**Molecular Formula:**	$C_{29}H_{28}F_3NO_5S_2$
**Molecular Weight:**	591.66 g/mol
**Smiles:**	CC1=CC(C)=C(S(=O)(N(CC2=CC=C(C(F)(F)F)O2)CC3=CC=C(C4=CC(S(=O)(C)=O)=CC=C4)C=C3)=O)C(C)=C1
**Recommended concentration:**	3 µM

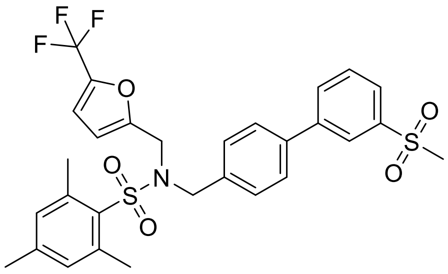

Biological activity

		Type	IC_{50}/EC_{50} [µM]	Reference
Main NR target:	NR1H3 (LXRα)	Antagonist	0.1	https://doi.org/10.1021/jm901797p
	NR1H2 (LXRβ)	Antagonist	0.04	
NR off-target:				

COMPOUND INFORMATION

Identity

1H NMR

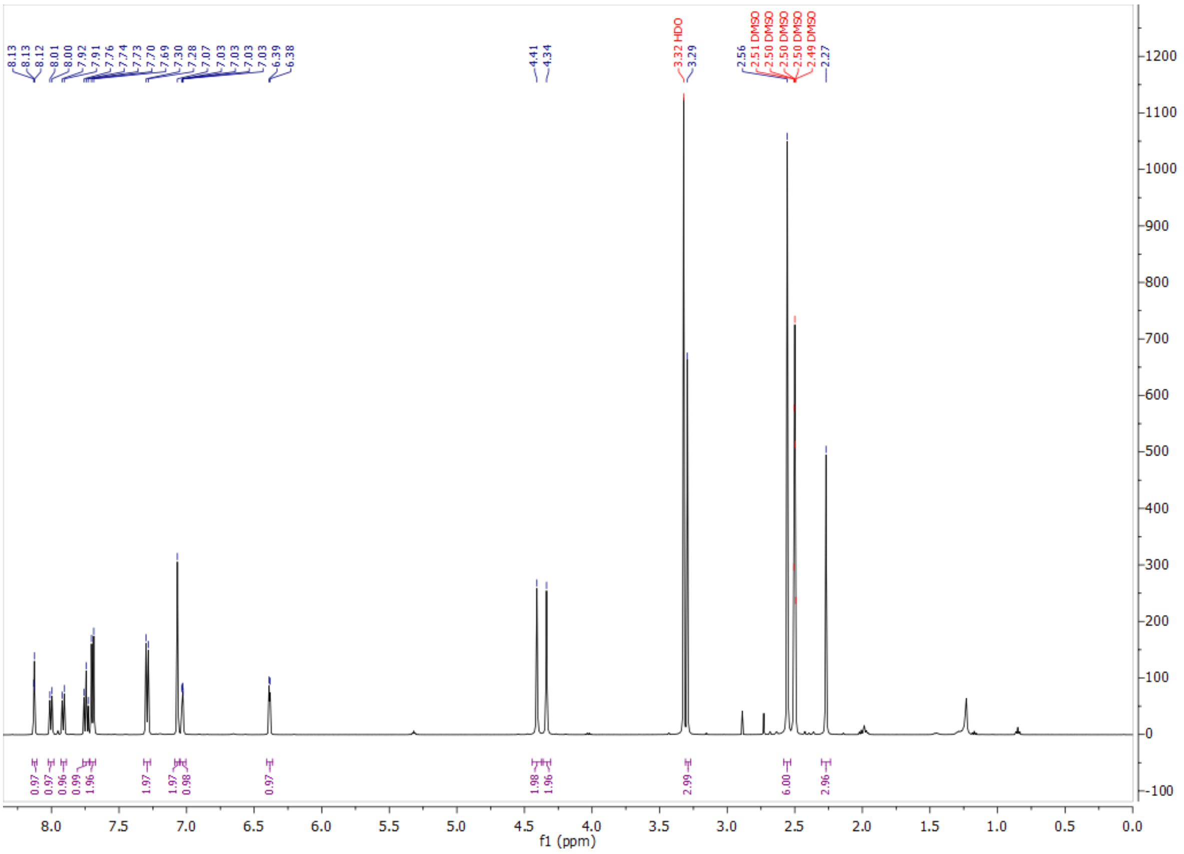

13C NMR

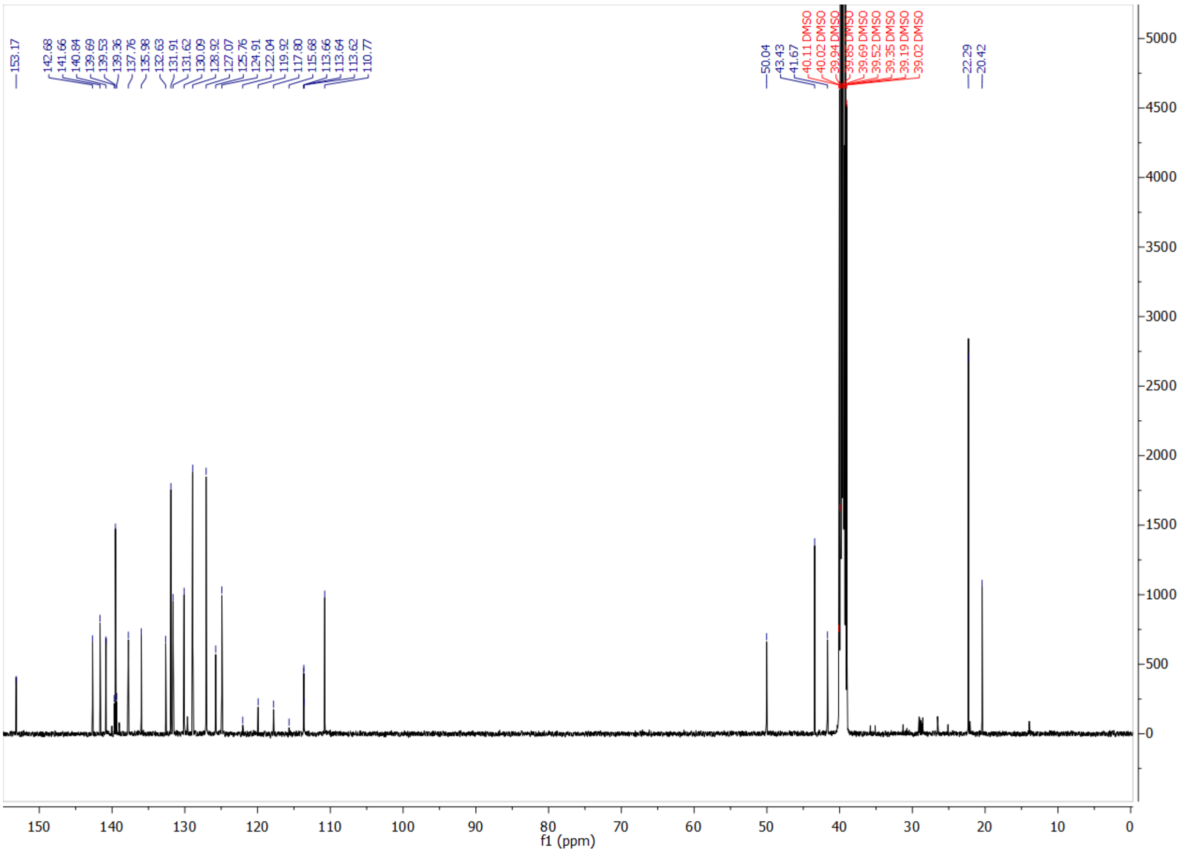

COMPOUND INFORMATION

Purity

LC-MS

M_r 591.66

MS: ESI-positive, m/z 592/245

LC: 0.1% HCOOH/ACN (20/80)

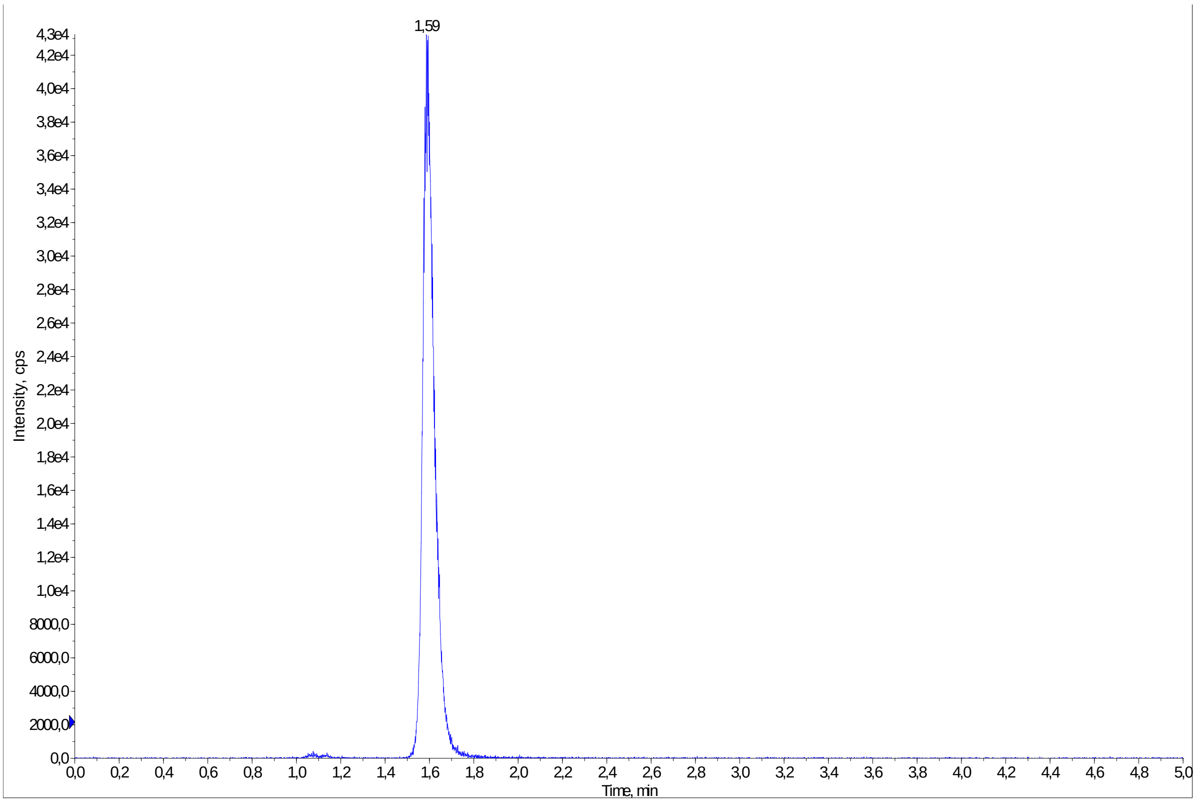

COMPOUND INFORMATION

LC-UV

LC: 0.1% HCOOH/ACN (30/70)

DAD: 210, 230, 240, 254 (XWC), 280 nm

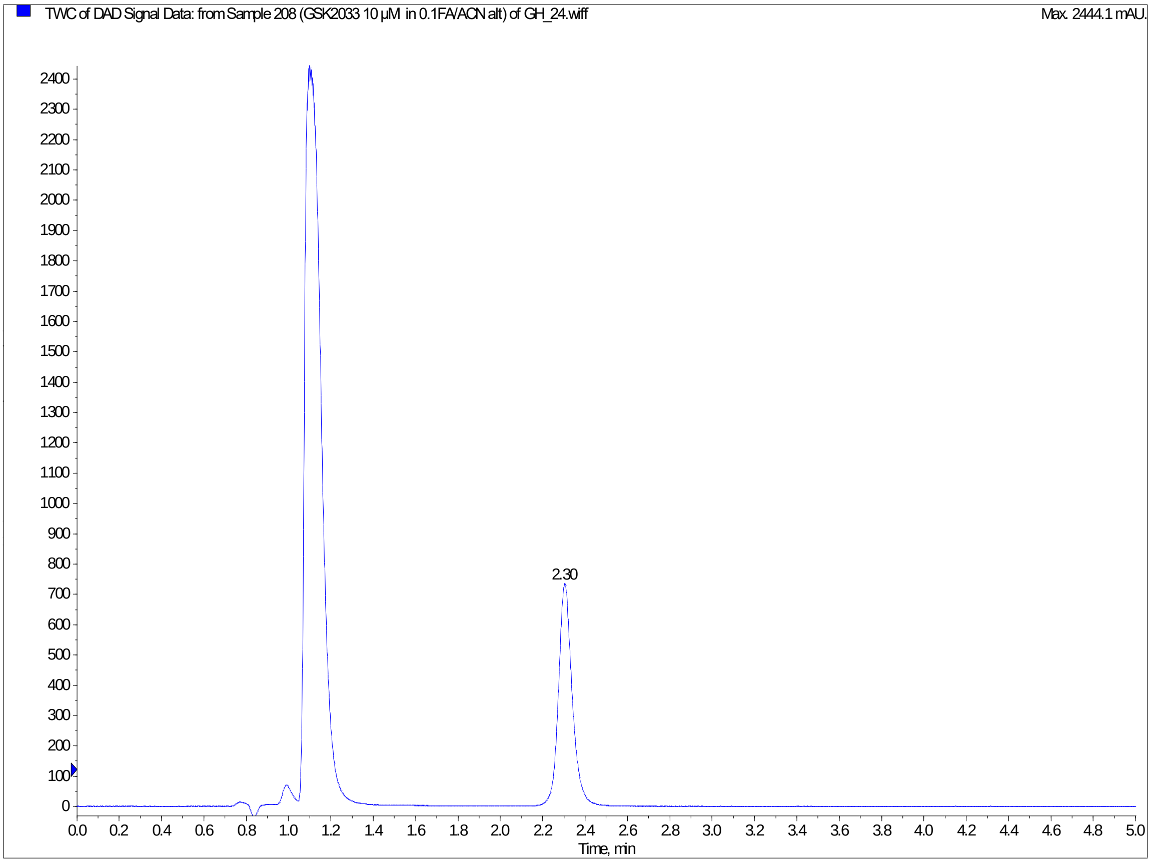

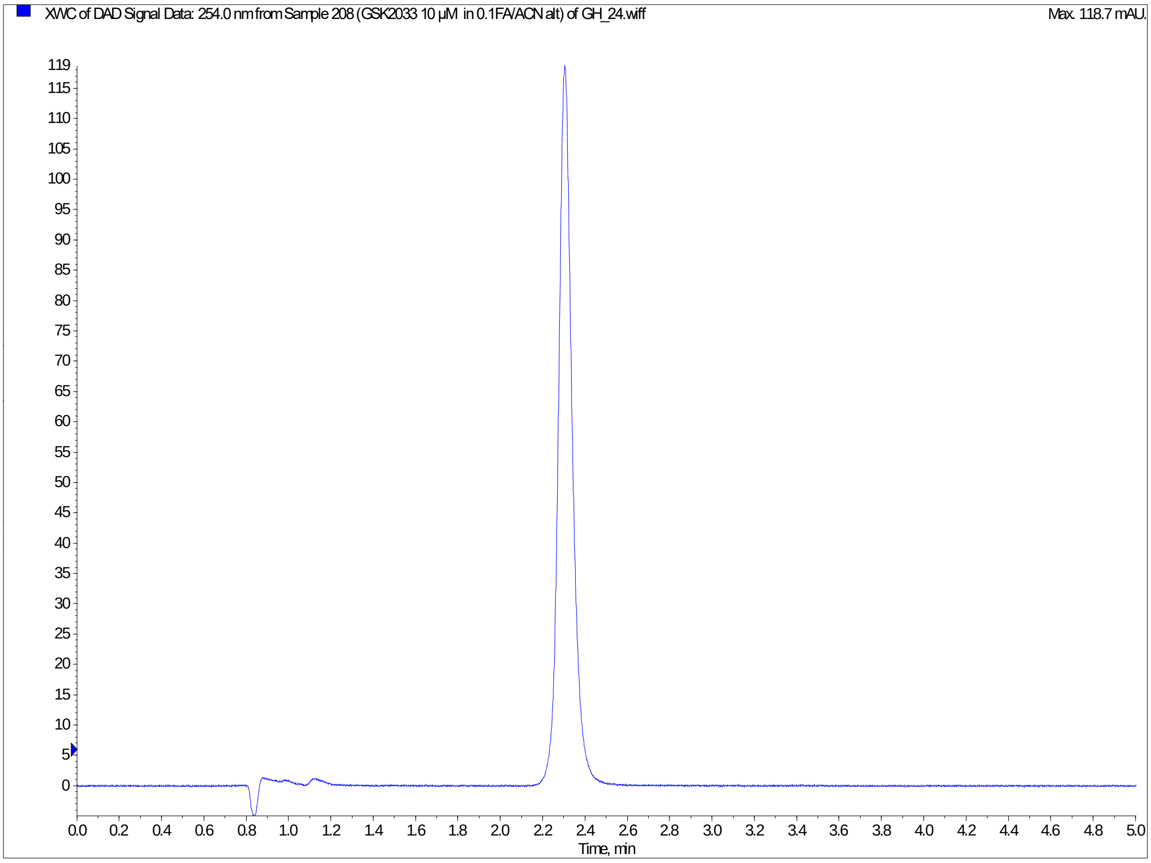

Supplement: Supplementary file 4 — Supplementary Data 1 [file 41467_2024_49493_MOESM4_ESM.zip › GSK2033.pdf]
