# Supplementary material for: Chemogenomics for NR1 nuclear hormone receptors
Source: Nat Commun. 2024 Jun 18;15:5201. doi: 10.1038/s41467-024-49493-6 (PMC11189487; doi:10.1038/s41467-024-49493-6)

COMPOUND INFORMATION

GSK2981278

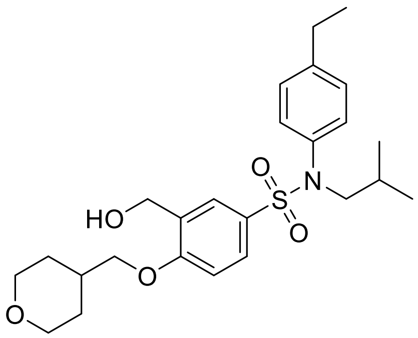

**CAS Registry No.:**	1474110-21-8
**Formal Name:**	N-(4-ethylphenyl)-3-(hydroxymethyl)-N-isobutyl-4-((tetrahydro-2H-pyran-4-yl)methoxy)benzenesulfonamide
**EUBOPEN ID:**	EUB0001159a
**Molecular Formula:**	$C_{25}H_{35}NO_5S$
**Molecular Weight:**	461.62 g/mol
**Smiles:**	OCC1=CC(S(N(C2=CC=C(C=C2)CC)CC(C)C)(=O)=O)=CC=C1OCC3CCOCC3
**Recommended concentration:**	1 µM

Biological activity

		Type	IC_{50}/EC_{50} [µM]	Reference
Main NR target:	NR1F3 (RORγ)	inv. Agonist	0.02	https://doi.org/10.1016/j.bmcl.2018.03.041
NR off-target:				

COMPOUND INFORMATION

Identity

^{1}H NMR

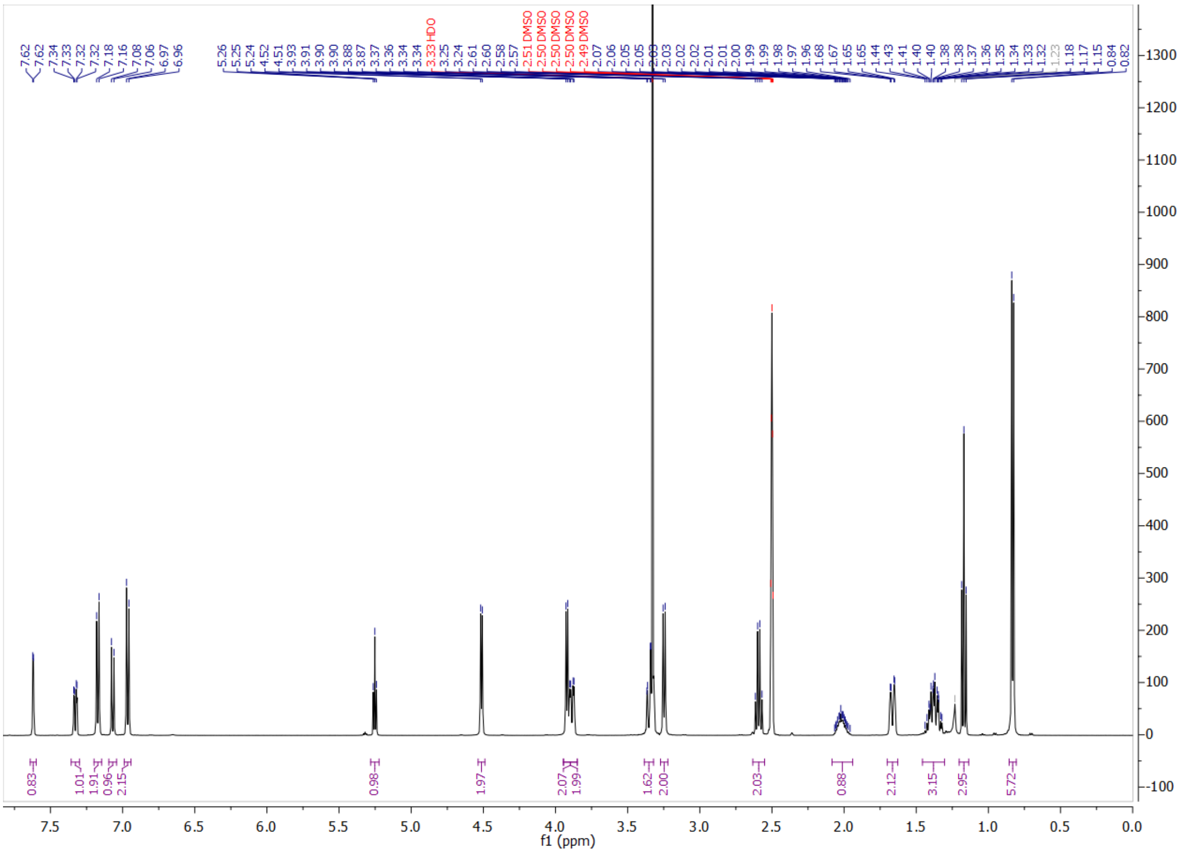

^{13}C NMR

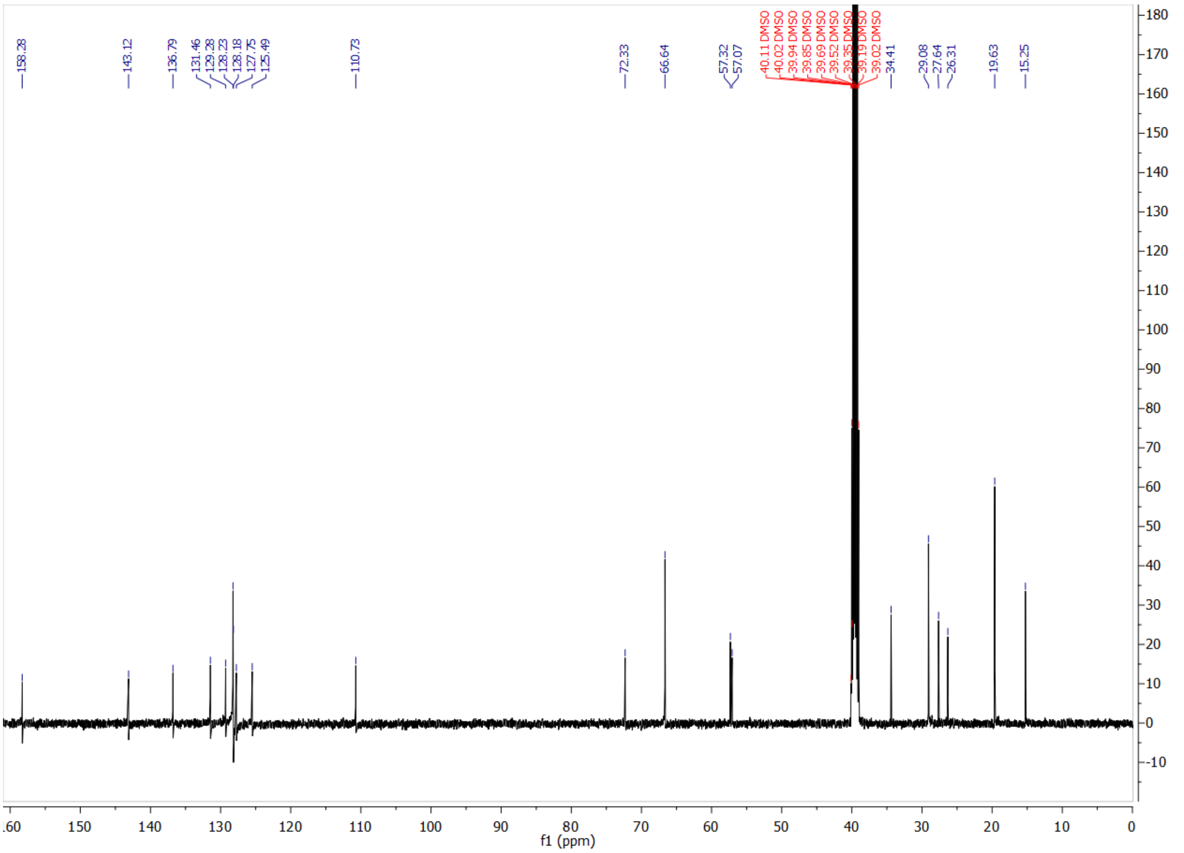

COMPOUND INFORMATION

Purity

Data File W:\analyti...\CGC_wave3_1_FirstPassB 2023-01-04 18-28-02\080-D2F-G5-GSK2981278.D
Sample Name: GSK2981278

```
=====================================================================
Acq. Operator   : SYSTEM                        Seq. Line :  80
Sample Operator : SYSTEM
Acq. Instrument : LCMS test                      Location :   D2F-G5
Injection Date  : 1/5/2023 9:04:24 AM                 Inj :   1
                                               Inj Volume : Inj prog
Sequence File   : W:\analytical_LCMS_DATA\EUbOPEN\CGC_wave3_1_FirstPassB 2023-01-04 18-28-02
                  \CGC_wave3_1_FirstPassB.S
Method          : W:\analytical_LCMS_DATA\EUbOPEN\CGC_wave3_1_FirstPassB 2023-01-04 18-28-02
                  \CGL_FIRSTPASS_GENERALMETHOD_VIAL1+2_20210319.M (Sequence Method)
Last changed    : 1/25/2022 4:36:18 PM by SYSTEM
Method Info     : CGL wellplate, 0.5 uL of 10 mM DMSO, general method
```

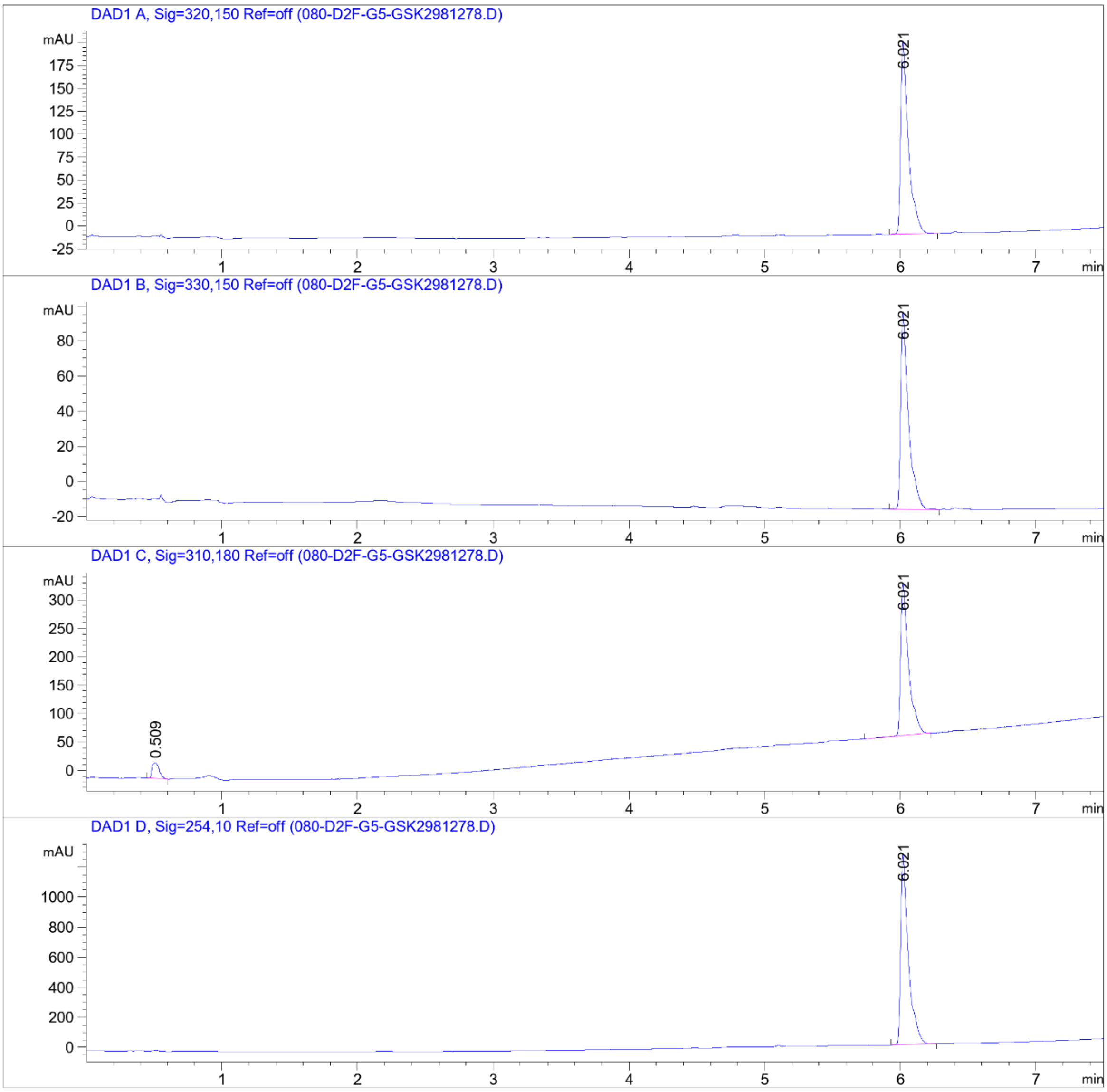

COMPOUND INFORMATION

Data File W:\analyti...\CGC_wave3_1_FirstPassB 2023-01-04 18-28-02\080-D2F-G5-GSK2981278.D
Sample Name: GSK2981278

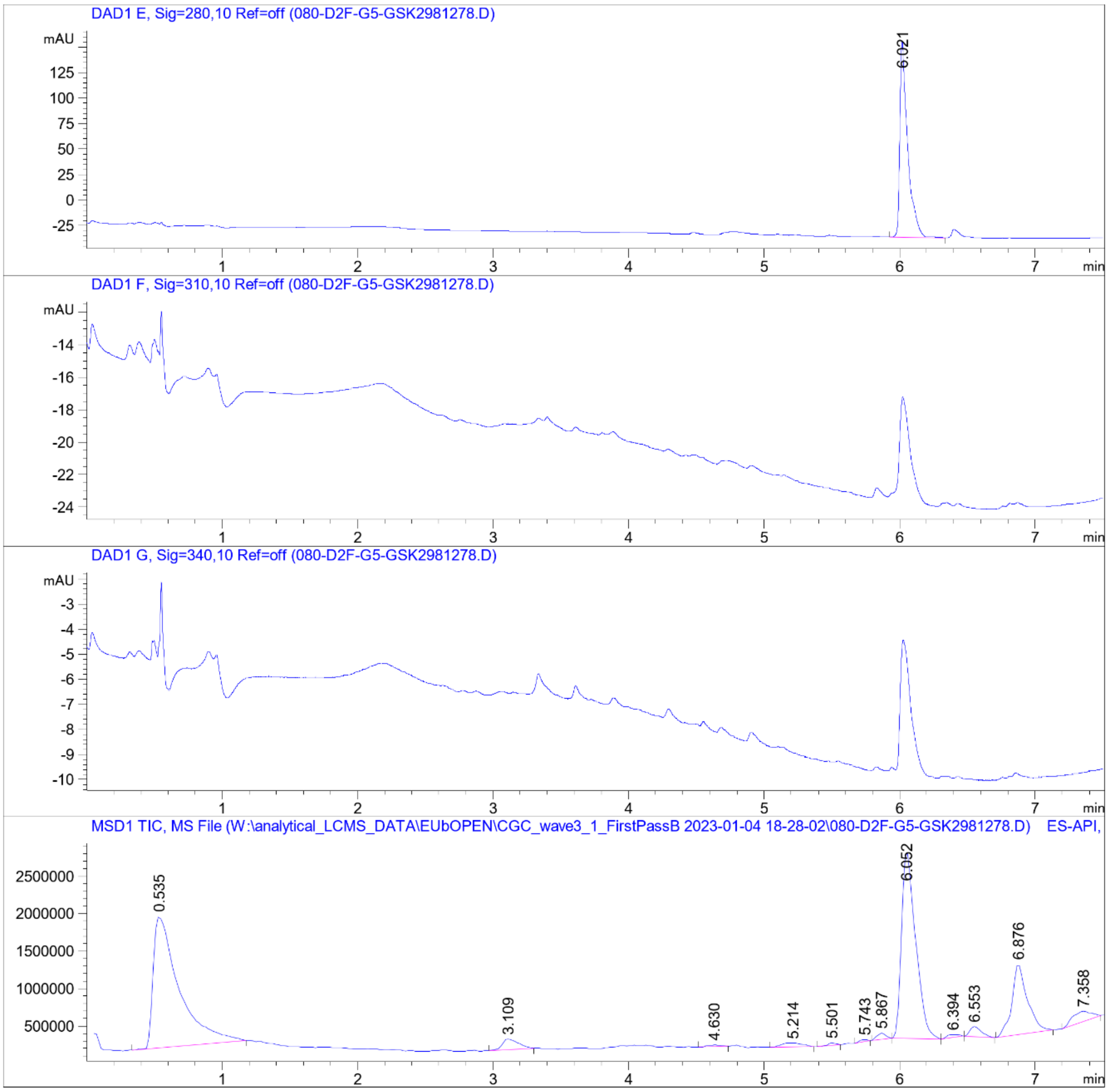

COMPOUND INFORMATION

Data File W:\analyti...\CGC_wave3_1_FirstPassB 2023-01-04 18-28-02\080-D2F-G5-GSK2981278.D
Sample Name: GSK2981278

MS Signal: MSD1 TIC, MS File, ES-API, Pos, Scan, Frag: 70, "POS Scan"
Spectra from peak tops.
Noise Cutoff: 1000 counts.
Reportable Ion Abundance: > 50%.
LC Signal: DAD1 A, Sig=320,150 Ref=off
Peak matching window: 0.1 min

Retention Time (LC)	LC Area	Retention Time (MS)	MS Area	Mol. Weight or Ion
-	-	0.535	23267944	157.00 I
-	-	3.109	1163551	239.10 I
				217.10 I
-	-	4.630	149446	510.30 I
				255.80 I
				170.90 I
				158.20 I
				137.10 I
-	-	5.214	545907	510.40 I
				338.20 I
				336.20 I
				316.30 I
				170.90 I
				137.10 I
				105.10 I
-	-	5.501	113315	510.30 I
				504.20 I
				137.10 I
				105.10 I
-	-	5.743	127128	280.20 I
-	-	5.867	365227	318.20 I
				296.20 I
6.021	821	6.052	18098704	462.20 I
-	-	6.394	237599	254.20 I
-	-	6.553	784433	507.20 I
				485.30 I
				280.20 I
-	-	6.876	7492533	282.20 I
-	-	7.358	1335475	400.30 I
				282.20 I

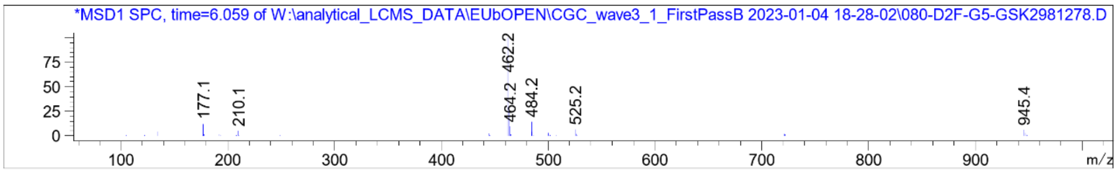

Supplement: Supplementary file 4 — Supplementary Data 1 [file 41467_2024_49493_MOESM4_ESM.zip › GSK2981278.pdf]
