# Supplementary material for: Chemogenomics for NR1 nuclear hormone receptors
Source: Nat Commun. 2024 Jun 18;15:5201. doi: 10.1038/s41467-024-49493-6 (PMC11189487; doi:10.1038/s41467-024-49493-6)

COMPOUND INFORMATION

GSK3787

**CAS Registry No.:**	188591-46-0
**Formal Name:**	4-chloro-N-(2-((5-(trifluoromethyl)pyridin-2-yl)sulfonyl)ethyl)benzamide
**EUbOPEN ID:**	EUB0001151a
**Molecular Formula:**	$C_{15}H_{12}ClF_3N_2O_3S$
**Molecular Weight:**	392.78 g/mol
**Smiles:**	C1=CC(=CC=C1C(=O)NCCS(=O)(=O)C2=NC=C(C=C2)C(F)(F)F)Cl
**Recommended concentration:**	1 µM

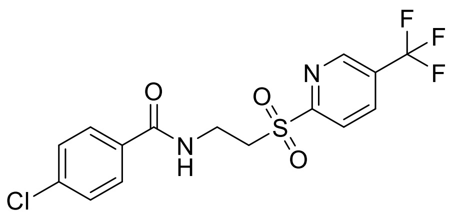

Biological activity

		Type	IC_{50}/EC_{50} [µM]	Reference
Main NR target:	NR1C2 (PPARδ)	Antagonist	0.13	https://doi.org/10.1021/jm900464j
NR off-target:	NR1C3 (PPARγ)	Antagonist	5	inhouse

COMPOUND INFORMATION

Identity

¹H NMR

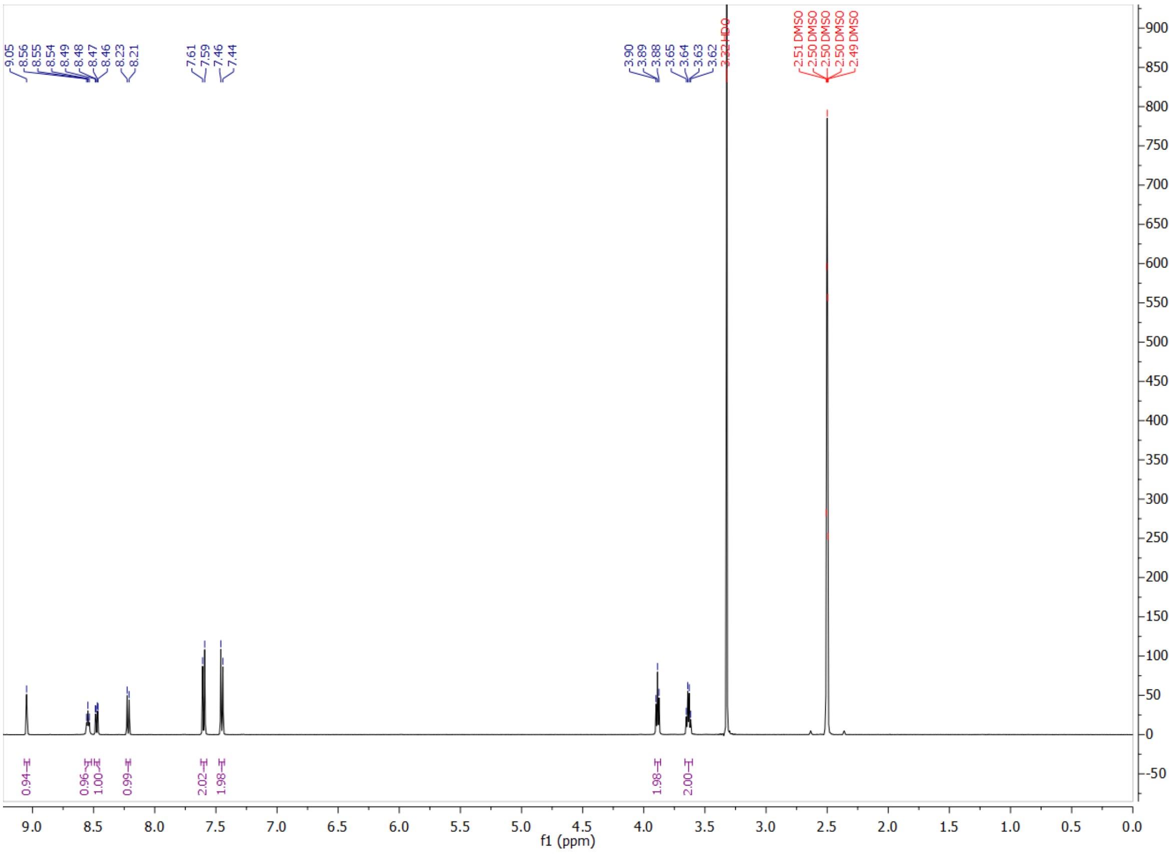

¹³C NMR

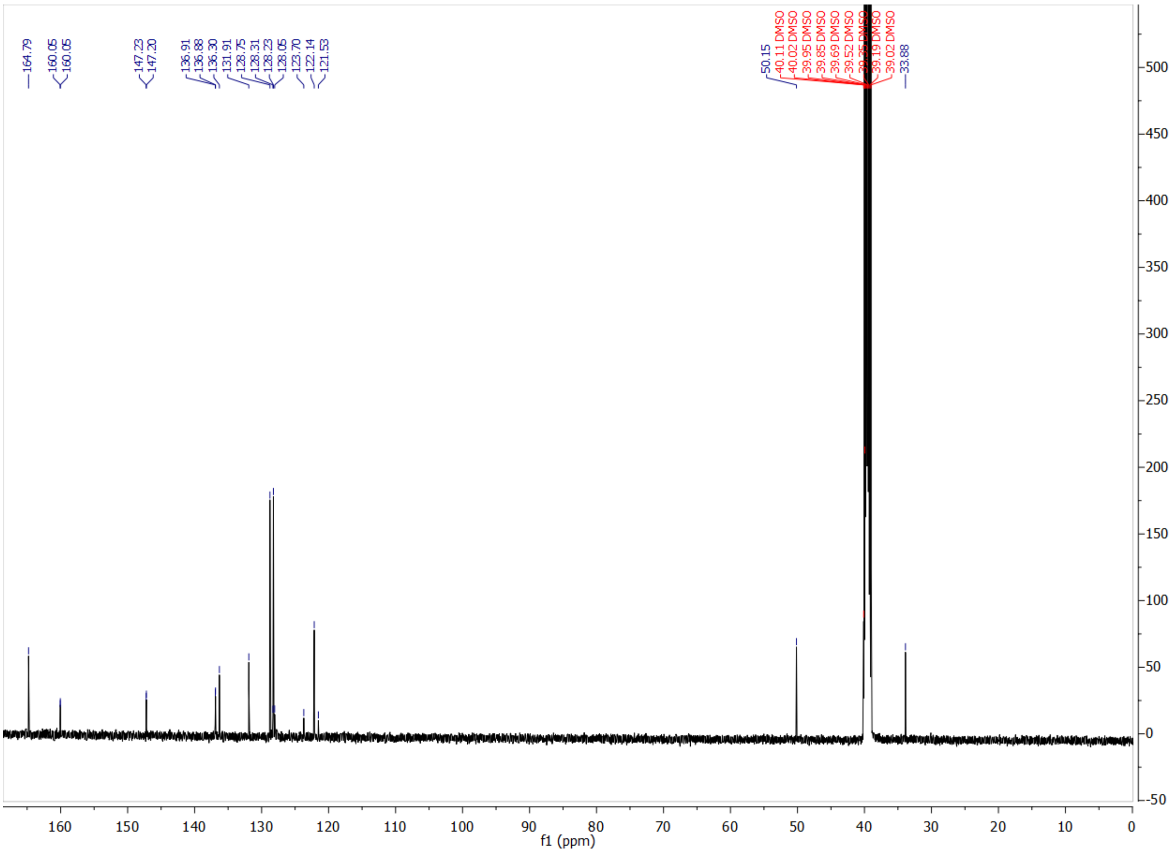

COMPOUND INFORMATION

EUbOPEN
Enabling & Unlocking Biology in the OPEN

Purity

```
Data File W:\analyti...PEN\CGC_wave3_1_FirstPassB 2023-01-04 18-28-02\072-D2F-F9-GSK3787.D
Sample Name: GSK3787

    =====================================================================
    Acq. Operator   : SYSTEM                          Seq. Line :   72
    Sample Operator : SYSTEM
    Acq. Instrument : LCMS test                        Location :   D2F-F9
    Injection Date  : 1/5/2023 7:35:56 AM                   Inj :   1
                                                 Inj Volume : Inj prog
    Sequence File   : W:\analytical_LCMS_DATA\EUbOPEN\CGC_wave3_1_FirstPassB 2023-01-04 18-28-02
                      \CGC_wave3_1_FirstPassB.S
    Method          : W:\analytical_LCMS_DATA\EUbOPEN\CGC_wave3_1_FirstPassB 2023-01-04 18-28-02
                      \CGL_FIRSTPASS_GENERALMETHOD_VIAL1+2_20210319.M (Sequence Method)
    Last changed    : 1/25/2022 4:36:18 PM by SYSTEM
    Method Info     : CGL wellplate, 0.5 uL of 10 mM DMSO, general method
```

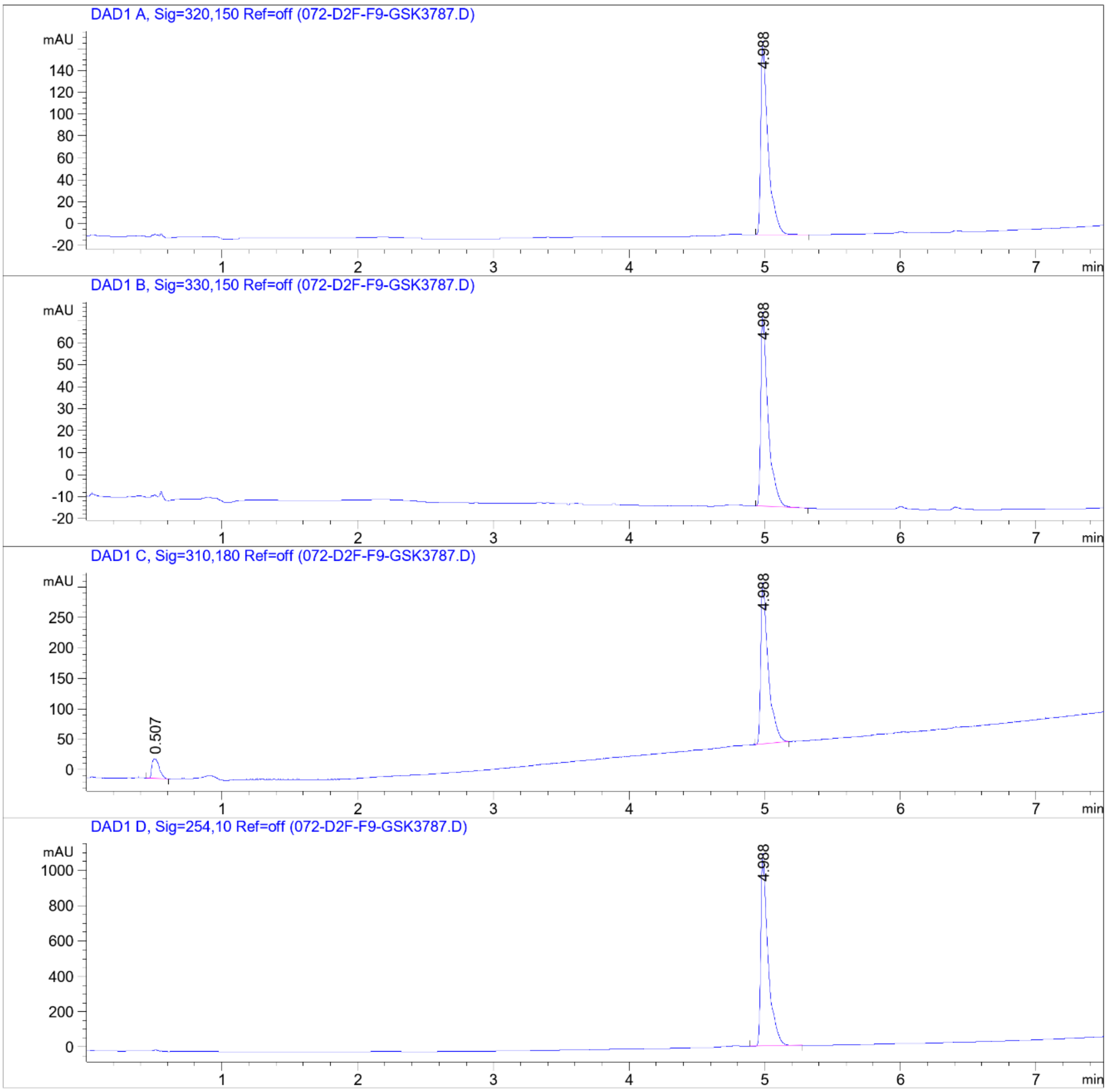

COMPOUND INFORMATION

Data File W:\analyti...PEN\CGC_wave3_1_FirstPassB 2023-01-04 18-28-02\072-D2F-F9-GSK3787.D
Sample Name: GSK3787

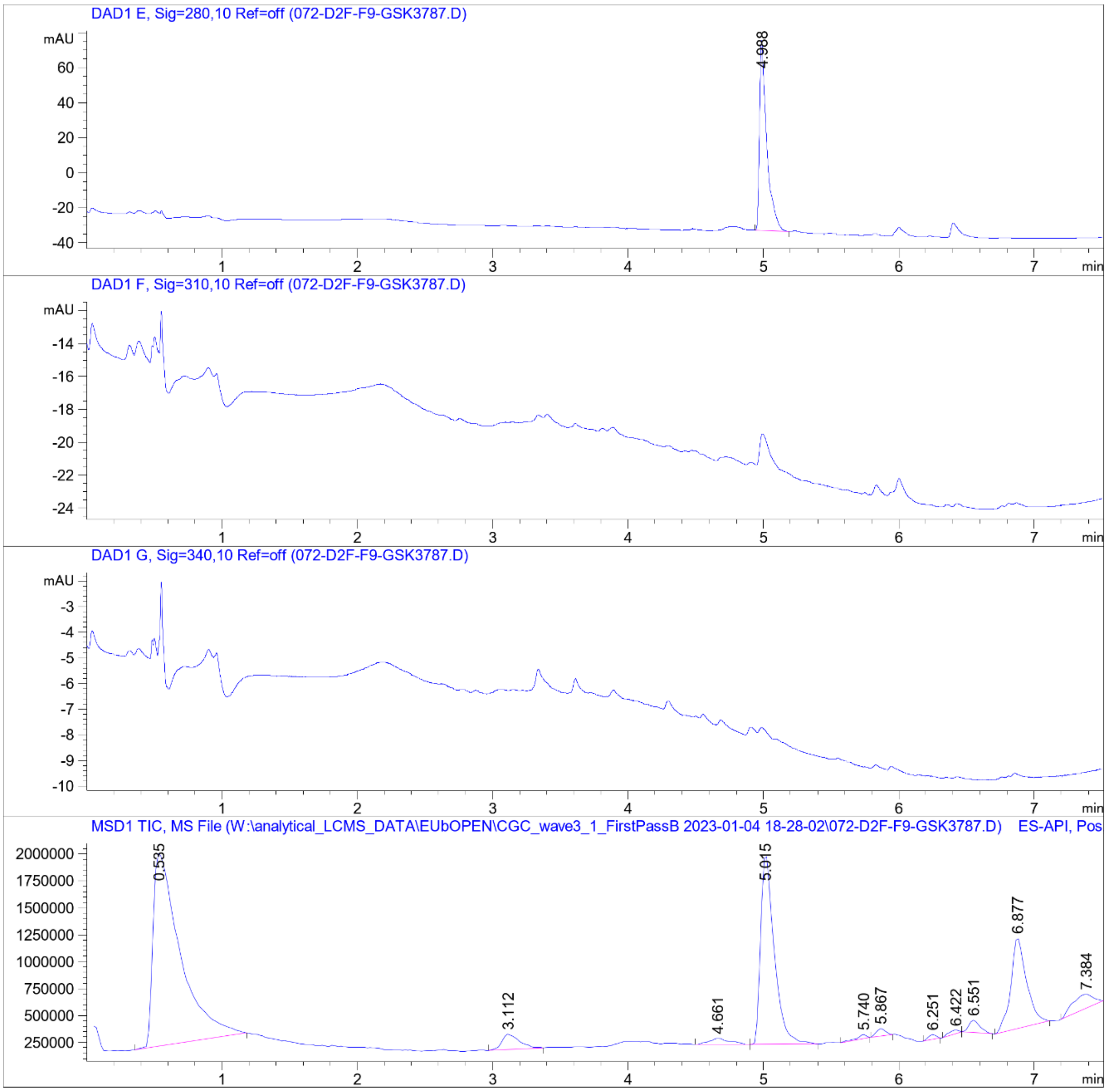

COMPOUND INFORMATION

Data File W:\analyti...PEN\CGC_wave3_1_FirstPassB 2023-01-04 18-28-02\072-D2F-F9-GSK3787.D
Sample Name: GSK3787

MS Signal: MSD1 TIC, MS File, ES-API, Pos, Scan, Frag: 70, "POS Scan"
Spectra from peak tops.
Noise Cutoff: 1000 counts.
Reportable Ion Abundance: > 50%.
LC Signal: DAD1 A, Sig=320,150 Ref=off
Peak matching window: 0.1 min

Retention Time (LC)	LC Area	Retention Time (MS)	MS Area	Mol. Weight or Ion
-	-	0.535	24993354	157.10 I
-	-	3.112	1282369	239.00 I
				217.10 I
-	-	4.661	595512	510.40 I
				170.90 I
				158.10 I
4.988	657	5.015	12271467	393.00 I
-	-	5.740	177366	280.20 I
-	-	5.867	320988	318.20 I
				296.20 I
-	-	6.251	181166	228.20 I
				137.10 I
-	-	6.422	175819	350.20 I
				282.20 I
				254.20 I
				137.10 I
-	-	6.551	635696	507.20 I
				485.30 I
				280.20 I
-	-	6.877	6791576	282.20 I
-	-	7.384	1494212	400.30 I
				282.20 I

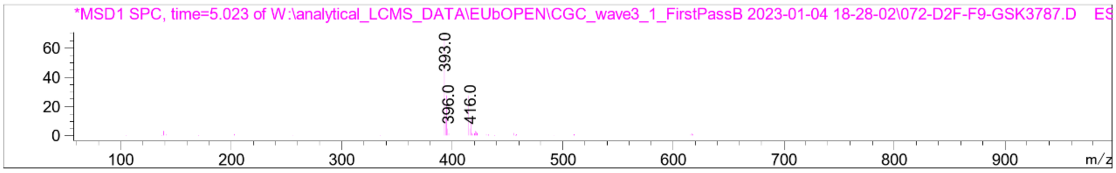

COMPOUND INFORMATION

Biological activity

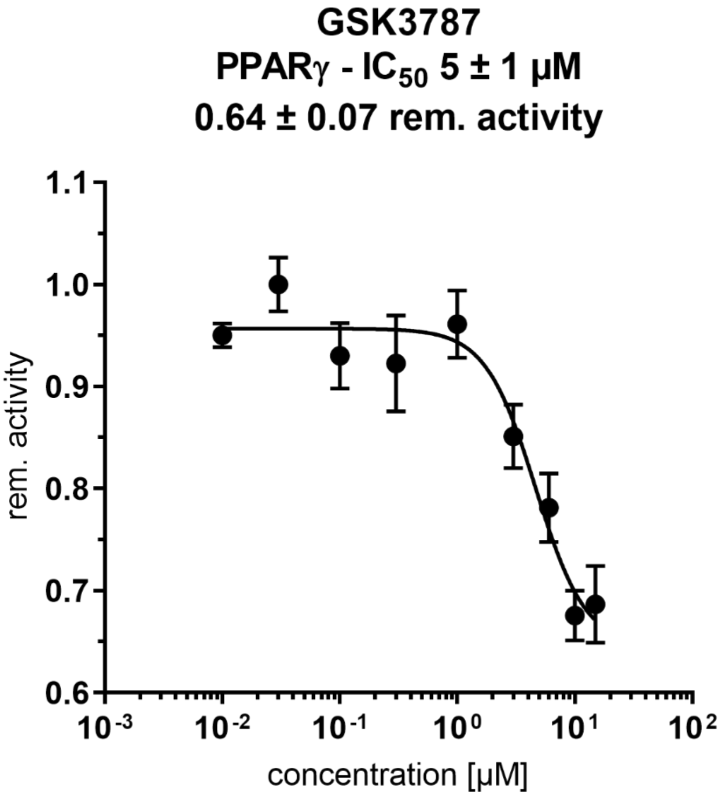

Supplement: Supplementary file 4 — Supplementary Data 1 [file 41467_2024_49493_MOESM4_ESM.zip › GSK3787.pdf]
