# Supplementary material for: Chemogenomics for NR1 nuclear hormone receptors
Source: Nat Commun. 2024 Jun 18;15:5201. doi: 10.1038/s41467-024-49493-6 (PMC11189487; doi:10.1038/s41467-024-49493-6)

COMPOUND INFORMATION

GW0742

**CAS Registry No.:**	317318-84-6
**Formal Name:**	2-(4-(((2-(3-fluoro-4-(trifluoromethyl)phenyl)-4-methylthiazol-5-yl)methyl)thio)-2-methylphenoxy)acetic acid
**EUbOPEN ID:**	EUB0000569a
**Molecular Formula:**	$C_{21}H_{17}F_4NO_3S_2$
**Molecular Weight:**	471.48 g/mol
**Smiles:**	CC1=C(C=CC(=C1)SCC2=C(N=C(S2)C3=CC(=C(C=C3)C(F)(F)F)F)C)OCC(=O)O
**Recommended concentration:**	1 µM

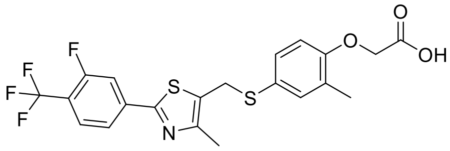

Biological activity

		Type	IC_{50}/EC_{50} [µM]	Reference
Main NR target:	NR1C2 (PPARδ)	Agonist	0.001	https://doi.org/10.1016/S0960-894X(03)00207-5
NR off-target:	NR1C1 (PPARα)	Agonist	1.1	https://doi.org/10.1016/S0960-894X(03)00207-5
	NR1C3 (PPARγ)	Agonist	2	

COMPOUND INFORMATION

Identity

¹H NMR

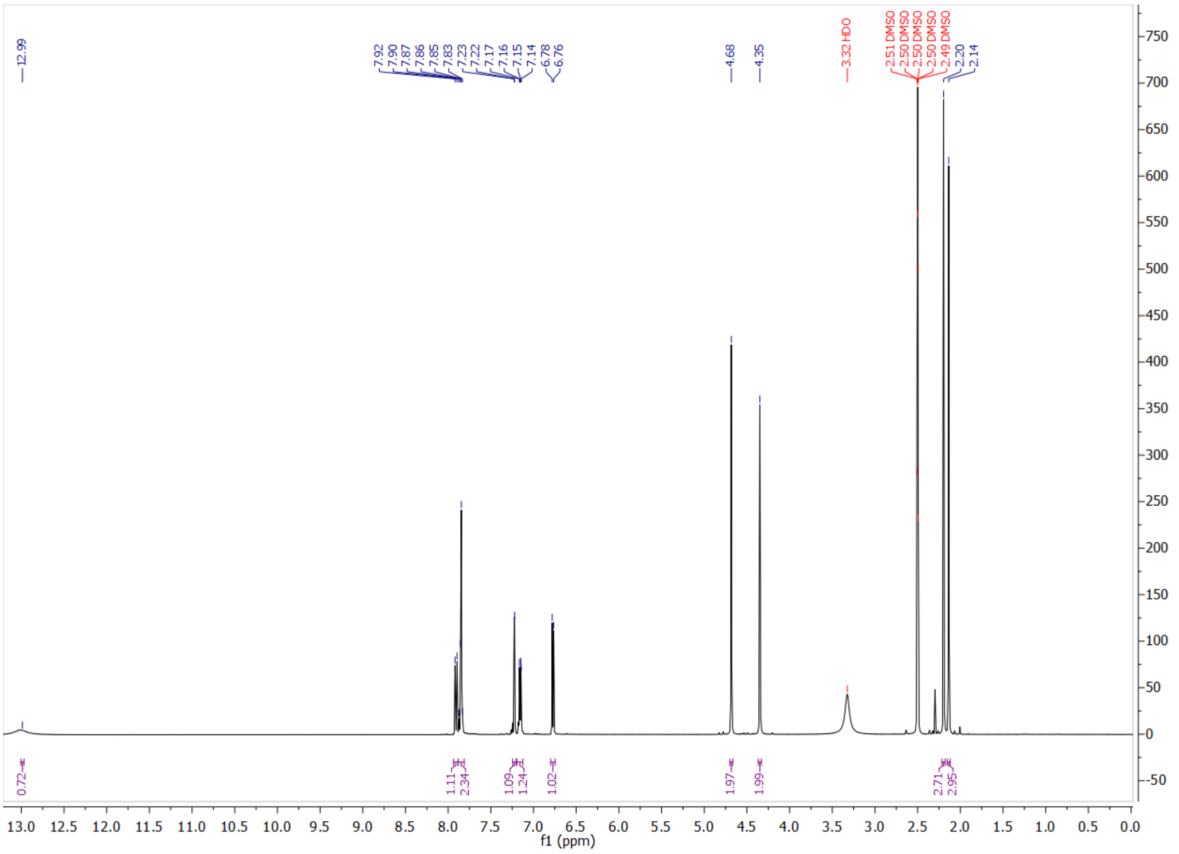

¹³C NMR

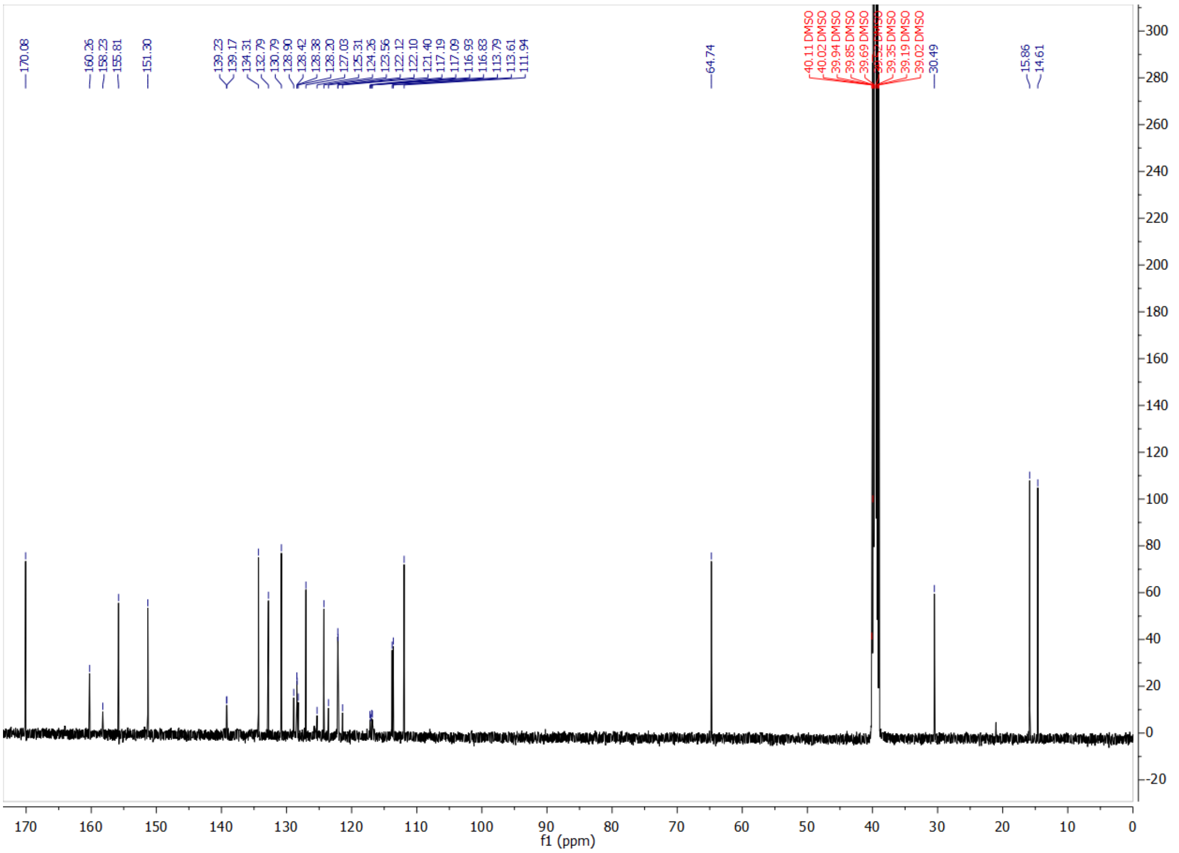

COMPOUND INFORMATION

Purity

```
Data File W:\analyti...bOPEN\CGC_ECHO1-3_FirstPass 2021-03-20 13-21-54\048-D2B-D9-GW0742.D
Sample Name: GW0742

      =====================================================================
      Acq. Operator    : SYSTEM                           Seq. Line :  48
      Sample Operator  : SYSTEM
      Acq. Instrument  : LCMS test                         Location :   D2B-D9
      Injection Date   : 3/20/2021 10:06:29 PM                  Inj :   1
                                                        Inj Volume : Inj prog
      Sequence File    : W:\analytical_LCMS_DATA\EUbOPEN\CGC_ECHO1-3_FirstPass 2021-03-20 13-21-54
                         \CGC_ECHO1-3_FirstPass.S
      Method           : W:\analytical_LCMS_DATA\EUbOPEN\CGC_ECHO1-3_FirstPass 2021-03-20 13-21-54
                         \CGL_FIRSTPASS_GENERALMETHOD_VIAL3+4_20210319.M (Sequence Method)
      Last changed     : 3/19/2021 5:35:24 PM by SYSTEM
      Method Info      : CGL wellplate, 0.5 uL of 10 mM DMSO, general method
```

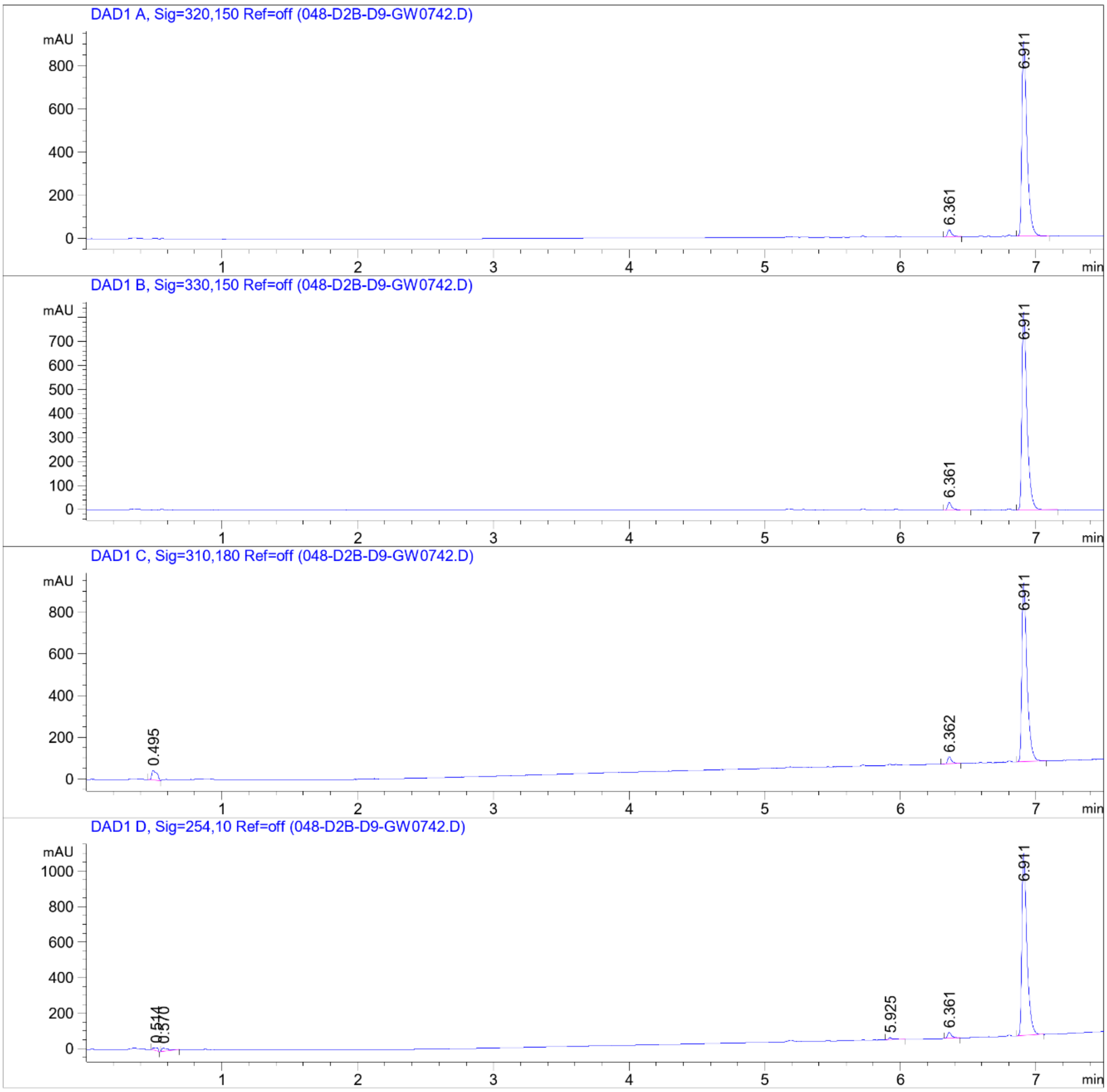

COMPOUND INFORMATION

Data File W:\analyti...bOPEN\CGC_ECHO1-3_FirstPass 2021-03-20 13-21-54\048-D2B-D9-GW0742.D
Sample Name: GW0742

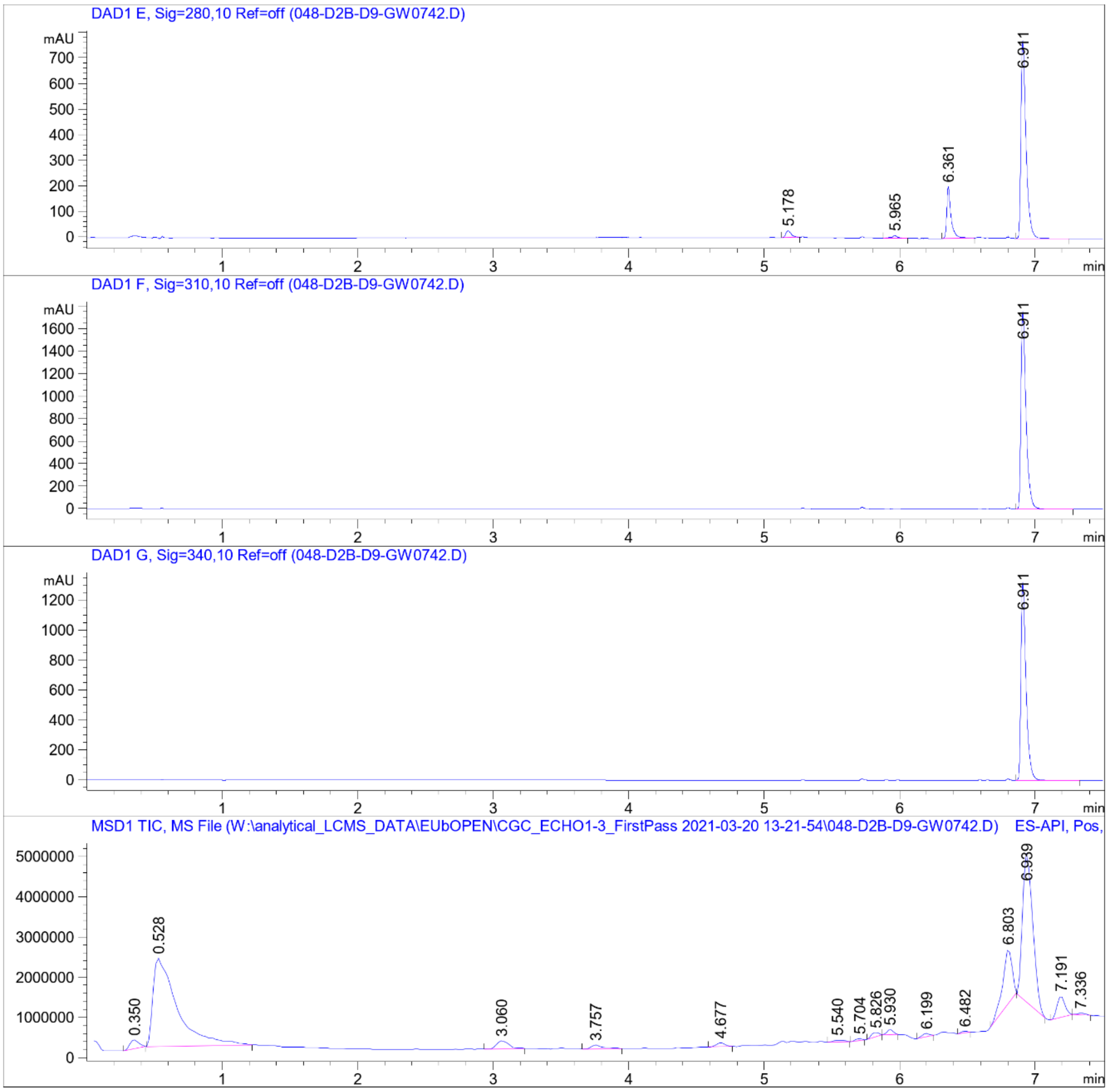

COMPOUND INFORMATION

Data File W:\analyti...bOPEN\CGC_ECHO1-3_FirstPass 2021-03-20 13-21-54\048-D2B-D9-GW0742.D
Sample Name: GW0742

MS Signal: MSD1 TIC, MS File, ES-API, Pos, Scan, Frag: 70, "POS Scan"
Spectra from peak tops.
Noise Cutoff: 1000 counts.
Reportable Ion Abundance: > 50%.
LC Signal: DAD1 A, Sig=320,150 Ref=off
Peak matching window: 0.1 min

Retention Time (LC)	LC Area	Retention Time (MS)	MS Area	Mol. Weight or Ion
-	-	0.350	980700	182.90 I
				158.00 I
				130.00 I
-	-	0.528	27156862	157.00 I
-	-	3.060	1290951	217.00 I
-	-	3.757	597148	274.20 I
-	-	4.677	412555	326.40 I
-	-	5.540	251422	326.20 I
				295.10 I
				282.20 I
				280.20 I
				102.20 I
-	-	5.704	145791	280.20 I
-	-	5.826	403374	296.20 I
-	-	5.930	391858	296.20 I
				294.10 I
				280.20 I
-	-	6.199	286367	280.20 I
				228.20 I
6.361	69	-	-	
-	-	6.482	158819	280.20 I
-	-	6.803	6534369	282.20 I
6.911	2347	6.939	19302544	472.10 I
-	-	7.191	2157972	284.30 I
				282.20 I
-	-	7.336	192554	400.30 I
				282.20 I

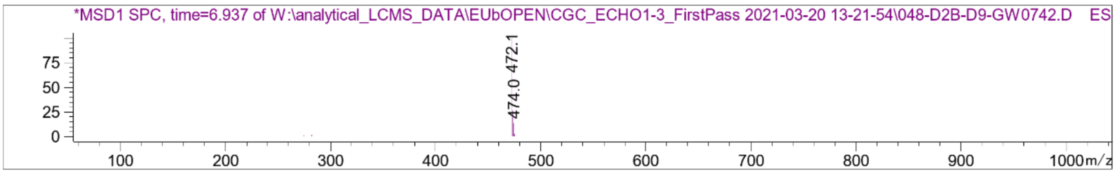

Supplement: Supplementary file 4 — Supplementary Data 1 [file 41467_2024_49493_MOESM4_ESM.zip › GW0742.pdf]
