# Supplementary material for: Chemogenomics for NR1 nuclear hormone receptors
Source: Nat Commun. 2024 Jun 18;15:5201. doi: 10.1038/s41467-024-49493-6 (PMC11189487; doi:10.1038/s41467-024-49493-6)

COMPOUND INFORMATION

GW1929

**CAS Registry No.:**	196808-24-9
**Formal Name:**	2-((2-benzoylphenyl)amino)-3-(4-(2-(methyl(pyridin-2-yl)amino)ethoxy)phenyl)propanoic acid
**EUbOPEN ID:**	EUB0000570a
**Molecular Formula:**	$C_{30}H_{29}N_3O_4$
**Molecular Weight:**	495.58 g/mol
**Smiles:**	CN(CCOC1=CC=C(C=C1)CC(C(=O)O)NC2=CC=CC=C2C(=O)C3=CC=CC=C3)C4=CC=CC=N4
**Recommended concentration:**	1 µM

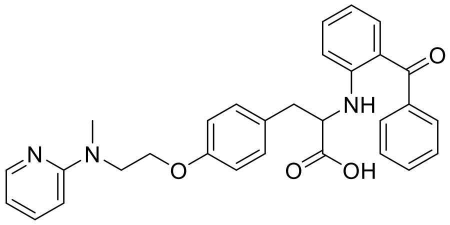

Biological activity

		Type	IC_{50}/EC_{50} [µM]	Reference
Main NR target:	NR1C3 (PPARγ)	Agonist	0.006	https://doi.org/10.1016/j.bmcl.2009.12.107
NR off-target:				

COMPOUND INFORMATION

Identity

^{1}H NMR

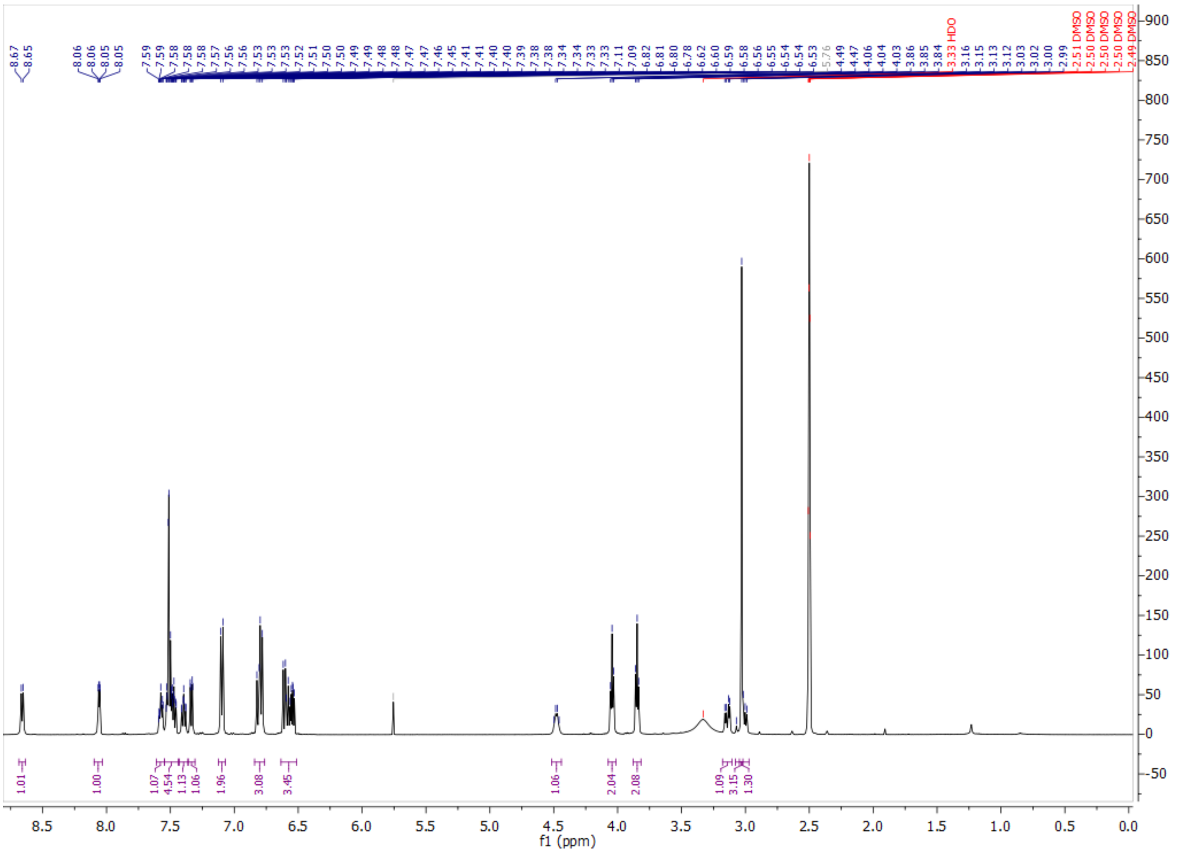

^{13}C NMR

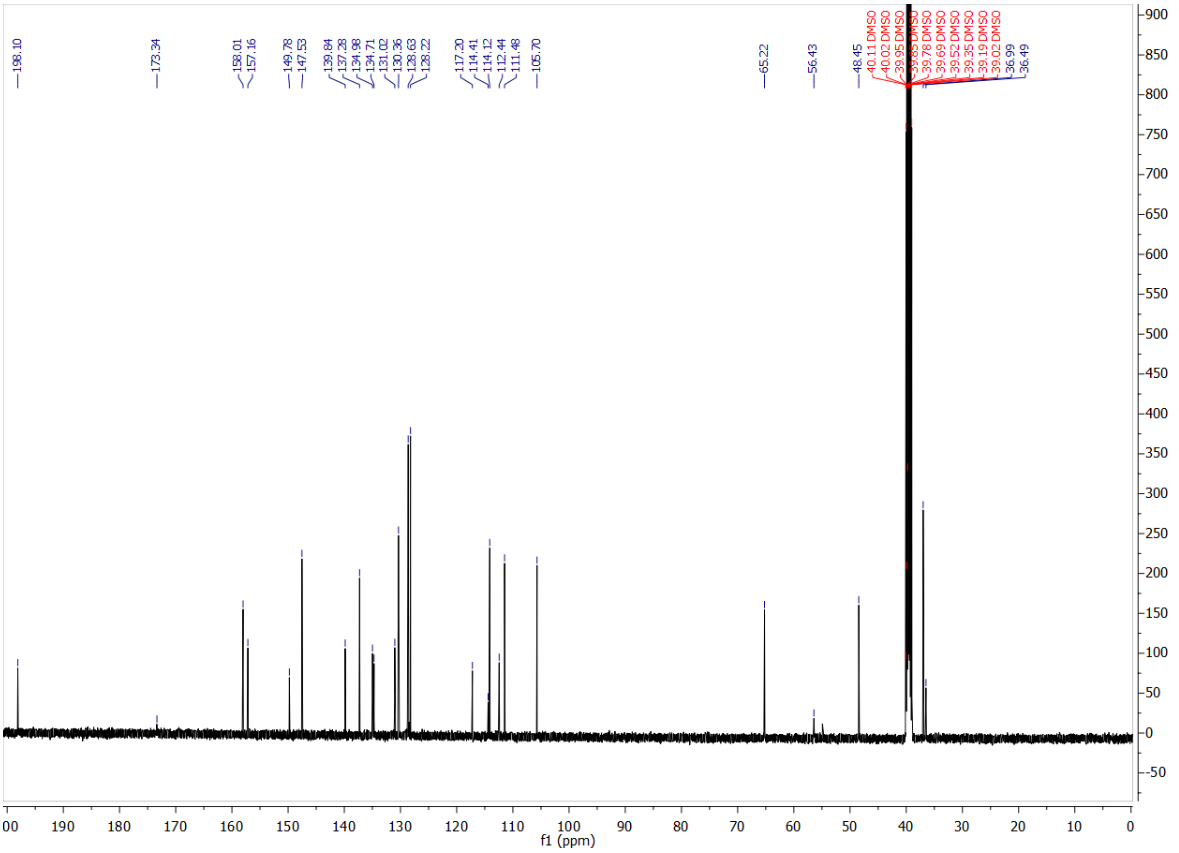

COMPOUND INFORMATION

Purity

Data File W:\analyti...C_ECHO1-3_FirstPass 2021-03-20 13-21-54\056-D2B-E5-GW1929 hydrate.D
Sample Name: GW1929 hydrate

```
=====================================================================
Acq. Operator   : SYSTEM                        Seq. Line :  56
Sample Operator : SYSTEM
Acq. Instrument : LCMS test                      Location :   D2B-E5
Injection Date  : 3/20/2021 11:35:07 PM               Inj :   1
                                                Inj Volume : Inj prog
Sequence File   : W:\analytical_LCMS_DATA\EUbOPEN\CGC_ECHO1-3_FirstPass 2021-03-20 13-21-54
                  \CGC_ECHO1-3_FirstPass.S
Method          : W:\analytical_LCMS_DATA\EUbOPEN\CGC_ECHO1-3_FirstPass 2021-03-20 13-21-54
                  \CGL_FIRSTPASS_GENERALMETHOD_VIAL3+4_20210319.M (Sequence Method)
Last changed    : 3/19/2021 5:35:24 PM by SYSTEM
Method Info     : CGL wellplate, 0.5 uL of 10 mM DMSO, general method
```

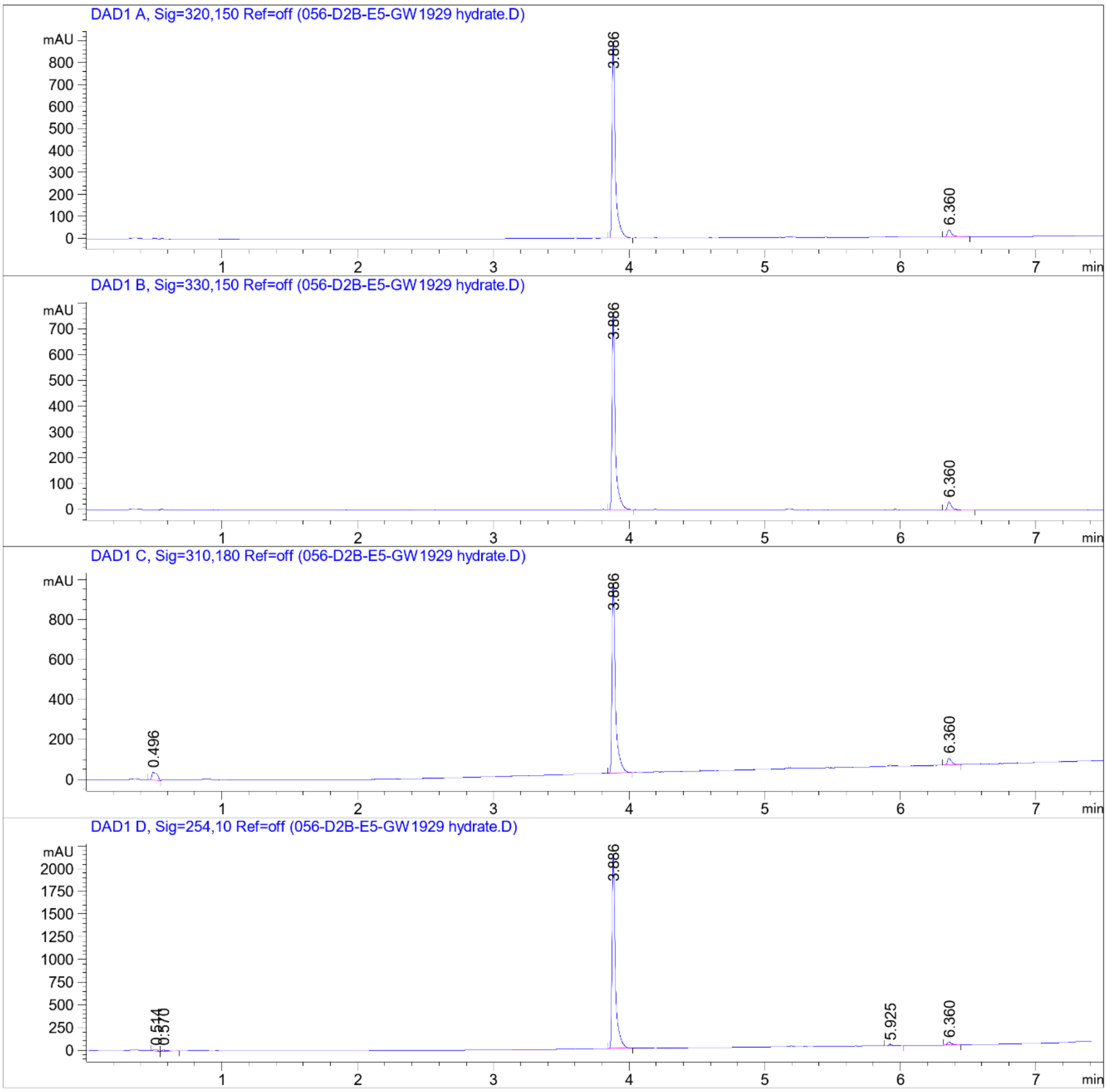

COMPOUND INFORMATION

Data File W:\analyti...C_ECHO1-3_FirstPass 2021-03-20 13-21-54\056-D2B-E5-GW1929 hydrate.D
Sample Name: GW1929 hydrate

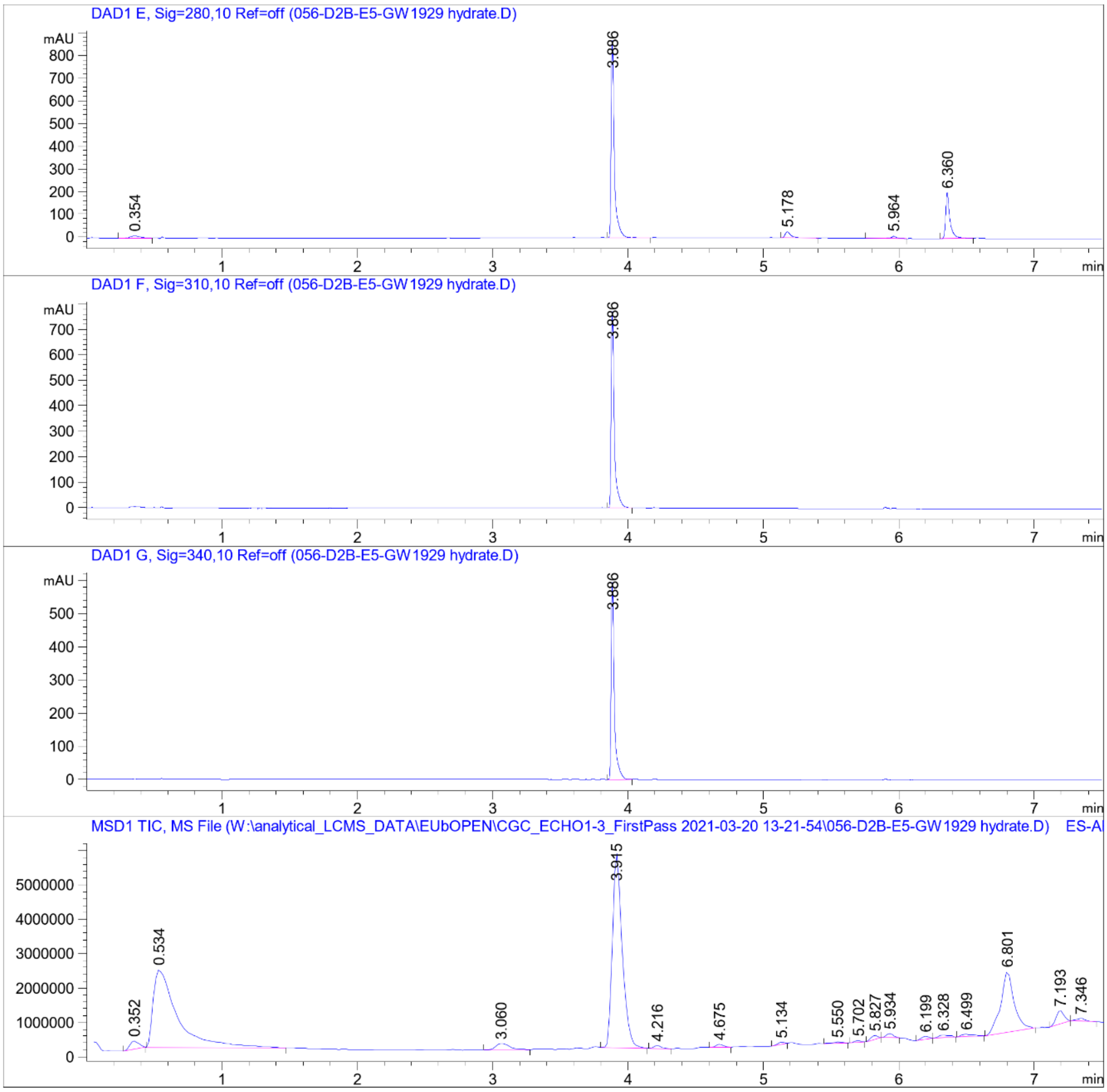

COMPOUND INFORMATION

Data File W:\analyti...C_ECHO1-3_FirstPass 2021-03-20 13-21-54\056-D2B-E5-GW1929 hydrate.D
Sample Name: GW1929 hydrate

MS Signal: MSD1 TIC, MS File, ES-API, Pos, Scan, Frag: 70, "POS Scan"
Spectra from peak tops.
Noise Cutoff: 1000 counts.
Reportable Ion Abundance: > 50%.
LC Signal: DAD1 A, Sig=320,150 Ref=off
Peak matching window: 0.1 min

Retention Time (LC)	LC Area	Retention Time (MS)	MS Area	Mol. Weight or Ion
-	-	0.352	1091682	182.90 I
				158.00 I
				130.10 I
-	-	0.534	28161148	157.00 I
-	-	3.060	1212548	217.00 I
3.886	1476	3.915	28685804	496.20 I
-	-	4.216	324718	452.20 I
				226.60 I
-	-	4.675	394699	326.30 I
-	-	5.134	201380	295.10 I
				225.10 I
				198.10 I
-	-	5.550	182296	326.20 I
				280.20 I
				102.20 I
-	-	5.702	172448	280.20 I
-	-	5.827	410675	296.20 I
-	-	5.934	403189	296.20 I
				294.10 I
				280.20 I
-	-	6.199	334305	280.20 I
				228.20 I
6.360	73	6.328	447837	254.20 I
-	-	6.499	487698	280.20 I
-	-	6.801	12089915	282.20 I
-	-	7.193	1578756	284.20 I
				282.20 I
-	-	7.346	296846	400.30 I
				282.20 I

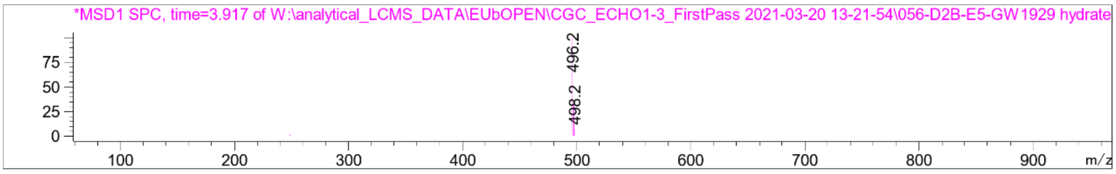

Supplement: Supplementary file 4 — Supplementary Data 1 [file 41467_2024_49493_MOESM4_ESM.zip › GW1929.pdf]
