# Supplementary material for: Chemogenomics for NR1 nuclear hormone receptors
Source: Nat Commun. 2024 Jun 18;15:5201. doi: 10.1038/s41467-024-49493-6 (PMC11189487; doi:10.1038/s41467-024-49493-6)

COMPOUND INFORMATION

GW3965 (hydrochloride)

**CAS Registry No.:**	405911-17-3
**Formal Name:**	2-(3-(3-((2-chloro-3-(trifluoromethyl)benzyl)(2,2-diphenylethyl)amino)propoxy)phenyl)acetic acid hydrochloride
**EUBOPEN ID:**	EUB0000572aCl
**Molecular Formula:**	$C_{33}H_{32}Cl_2F_3NO_3$
**Molecular Weight:**	618.52 g/mol
**Smiles:**	O=C(O)CC1=CC(OCCCN(CC2=C(Cl)C(C(F)(F)F)=CC=C2)CC(C3=CC=CC=C3)C4=CC=CC=C4)=CC=C1.Cl
**Recommended concentration:**	3 µM

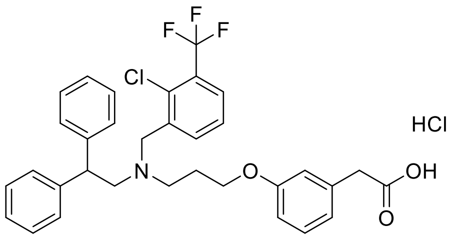

Biological activity

		Type	IC_{50}/EC_{50} [µM]	Reference
Main NR target:	NR1H3 (LXRα)	Agonist	0.19	https://doi.org/10.1021/jm0255116
	NR1H2 (LXRβ)	Agonist	2.1	inhouse
NR off-target:				

COMPOUND INFORMATION

Identity

^{1}H NMR

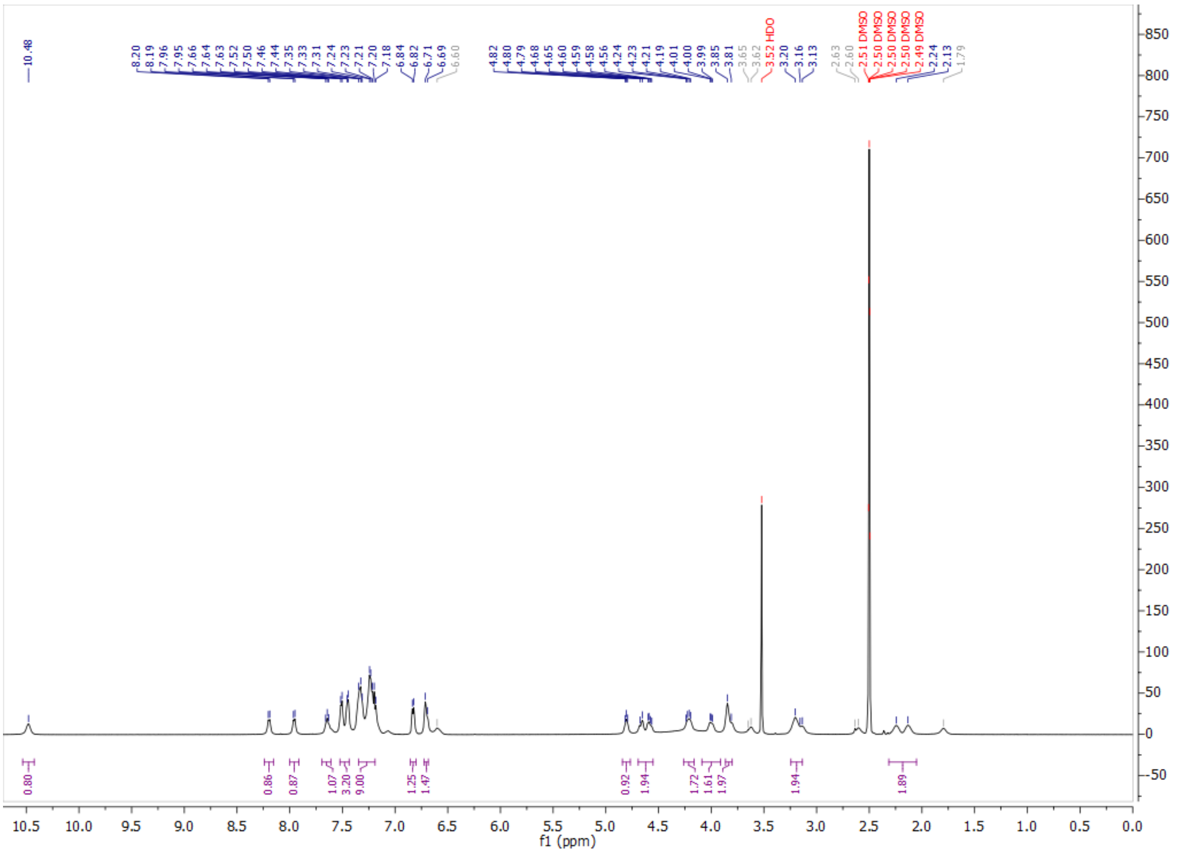

^{13}C NMR

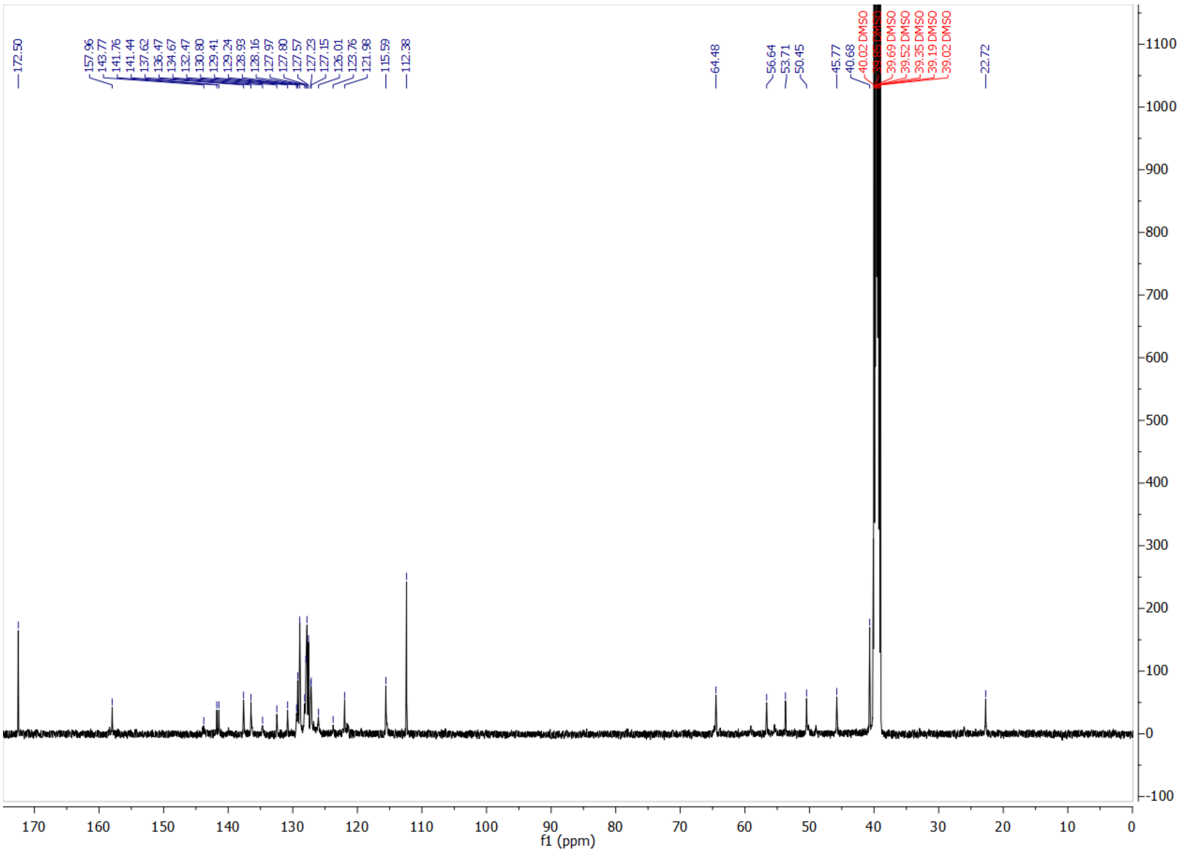

COMPOUND INFORMATION

Purity

Data File W:\analyti...OPEN\CGC_ECHO1-3_SecondPass 2021-03-25 18-09-21\011-D2F-E7-GW3965.D
Sample Name: GW3965

```
=====================================================================
Acq. Operator   : SYSTEM                          Seq. Line :  11
Sample Operator : SYSTEM
Acq. Instrument : LCMS test                        Location :  D2F-E7
Injection Date  : 3/25/2021 8:02:31 PM                  Inj :  1
                                                Inj Volume : Inj prog
Sequence File   : W:\analytical_LCMS_DATA\EUbOPEN\CGC_ECHO1-3_SecondPass 2021-03-25 18-09-21
                  \CGC_ECHO1-3_SecondPass.S
Method          : W:\analytical_LCMS_DATA\EUbOPEN\CGC_ECHO1-3_SecondPass 2021-03-25 18-09-21
                  \CGL_SECONDPASS_NONPOLCOMP_VIAL2+4_20210323.M (Sequence Method)
Last changed    : 3/25/2021 4:32:02 PM by SYSTEM
Method Info     : CGL wellplate, 0.5 uL of 10 mM DMSO. Dilution with MeCN only (9+9 uL)
```

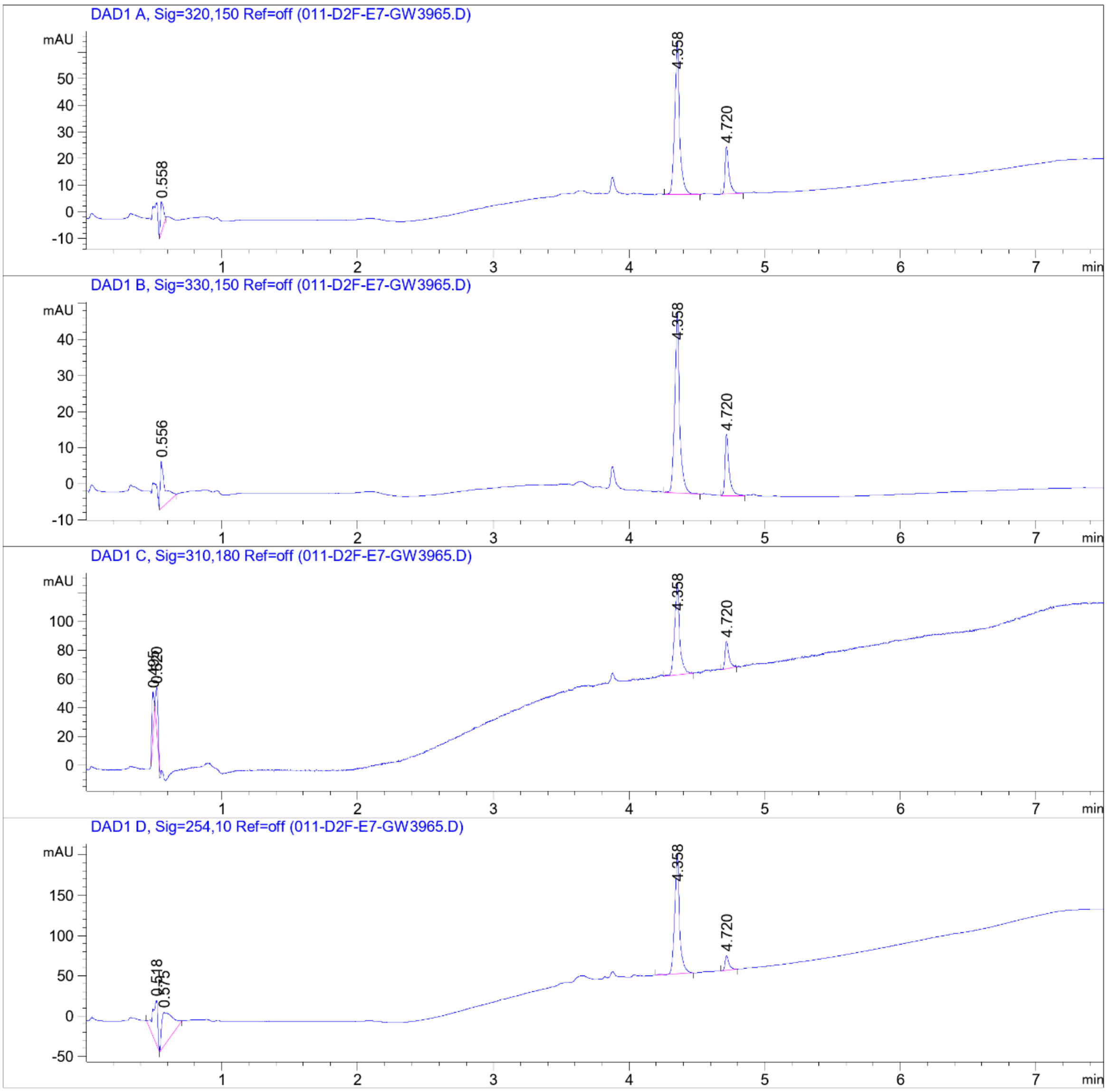

COMPOUND INFORMATION

Data File W:\analyti...OPEN\CGC_ECHO1-3_SecondPass 2021-03-25 18-09-21\011-D2F-E7-GW3965.D
Sample Name: GW3965

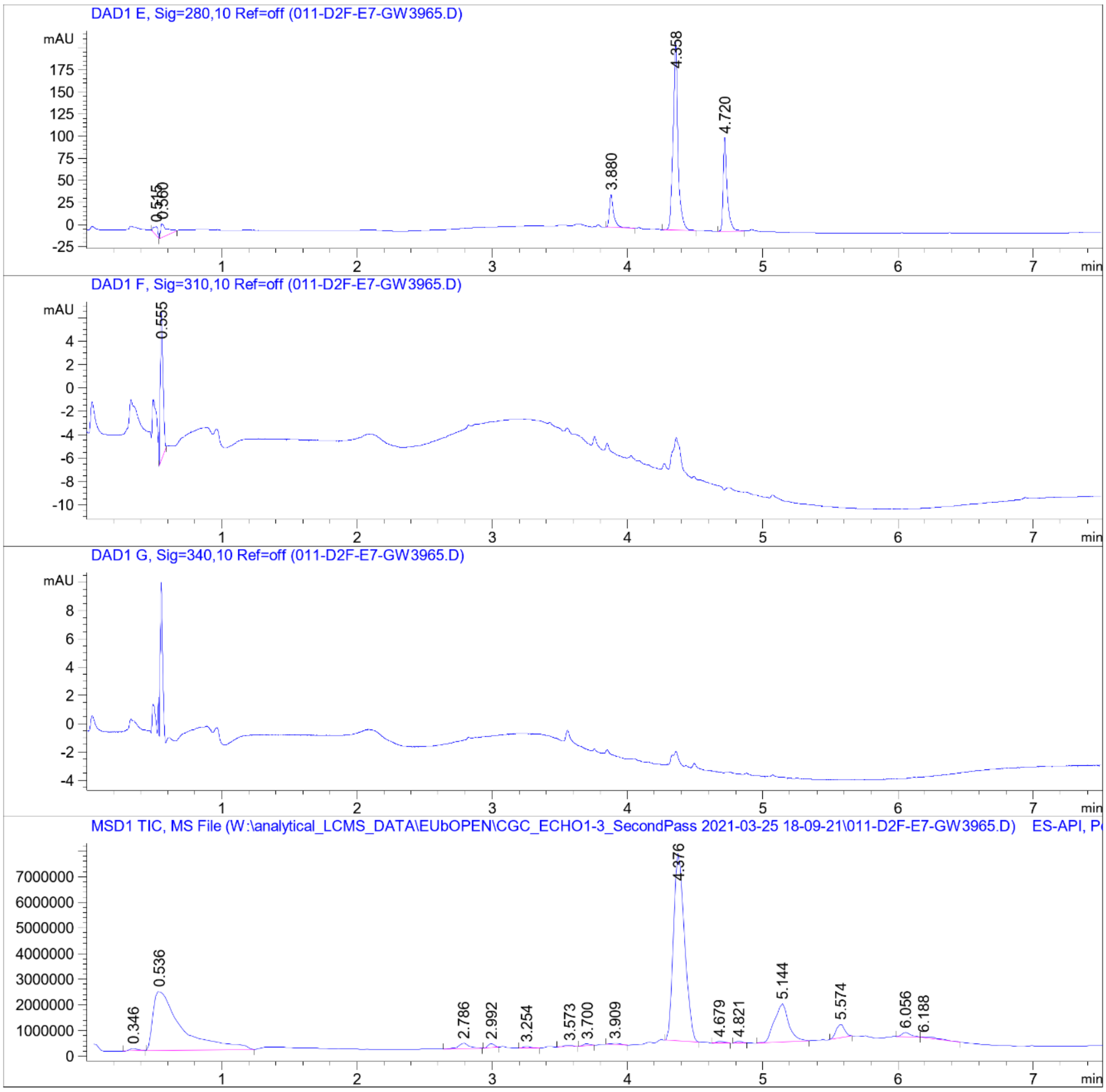

COMPOUND INFORMATION

Data File W:\analyti...OPEN\CGC_ECHO1-3_SecondPass 2021-03-25 18-09-21\011-D2F-E7-GW3965.D
Sample Name: GW3965

MS Signal: MSD1 TIC, MS File, ES-API, Pos, Scan, Frag: 70, "POS Scan"
Spectra from peak tops.
Noise Cutoff: 1000 counts.
Reportable Ion Abundance: > 50%.
LC Signal: DAD1 A, Sig=320,150 Ref=off
Peak matching window: 0.1 min

Retention Time (LC)	LC Area	Retention Time (MS)	MS Area	Mol. Weight or Ion
-	-	0.346	376675	200.00 I
				183.00 I
				159.00 I
0.558	17	0.536	33732896	157.10 I
-	-	2.786	1132223	217.10 I
-	-	2.992	468838	274.30 I
-	-	3.254	289581	448.20 I
-	-	3.573	271154	224.10 I
				111.10 I
-	-	3.700	304169	312.20 I
				214.10 I
-	-	3.909	247316	512.50 I
4.358	145	4.376	42142564	582.30 I
4.720	36	4.679	302282	254.30 I
-	-	4.821	248968	280.20 I
-	-	5.144	10709928	282.30 I
-	-	5.574	2316434	381.30 I
				359.30 I
				341.30 I
				284.30 I
				282.30 I
-	-	6.056	715062	338.30 I
				282.30 I
-	-	6.188	388252	282.30 I

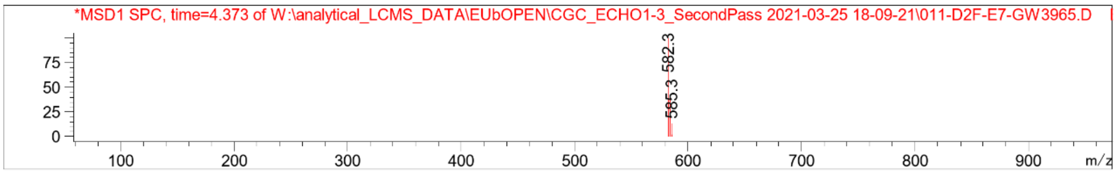

COMPOUND INFORMATION

Biological activity

GW3965
LXRβ - EC_{50} 2.1 ± 0.5 μM
28 ± 3 fold activation

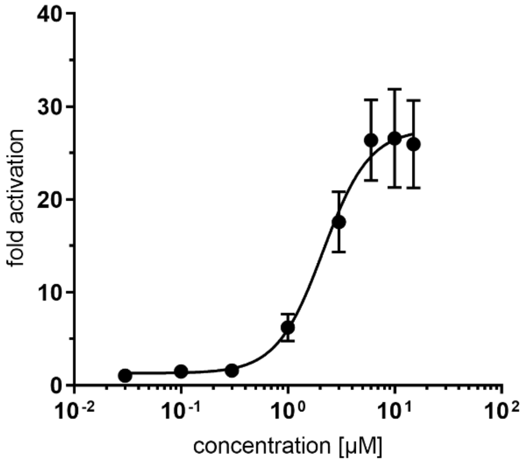

Supplement: Supplementary file 4 — Supplementary Data 1 [file 41467_2024_49493_MOESM4_ESM.zip › GW3965.pdf]
