# Supplementary material for: Chemogenomics for NR1 nuclear hormone receptors
Source: Nat Commun. 2024 Jun 18;15:5201. doi: 10.1038/s41467-024-49493-6 (PMC11189487; doi:10.1038/s41467-024-49493-6)

COMPOUND INFORMATION

EUbOPEN Enabling & Unlocking Biology in the OPEN

GW4064

CAS Registry No.: 278779-30-9

Formal Name: (E)-3-(2-chloro-4-((3-(2,6-dichlorophenyl)-5-isopropylisoxazol-4-yl)methoxy)styryl)benzoic acid

EUBOPEN ID: EUB0000184b

Molecular Formula: $C_{28}H_{22}Cl_3NO_4$

Molecular Weight: 542.84 g/mol

Smiles: O=C(C1=CC=CC(/C=C/C2=CC=C(C=C2Cl)OCC3=C(ON=C3C4=C(C=CC=C4Cl)Cl)C(C)C)=C1)O

Recommended concentration: 1 µM

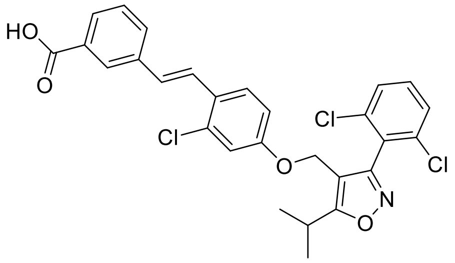

Biological activity

		Type	IC_{50}/EC_{50} [µM]	Reference
Main NR target:	NR1H4 (FXR)	Agonist	0.37	inhouse
NR off-target:				

COMPOUND INFORMATION

Identity

^{1}H NMR

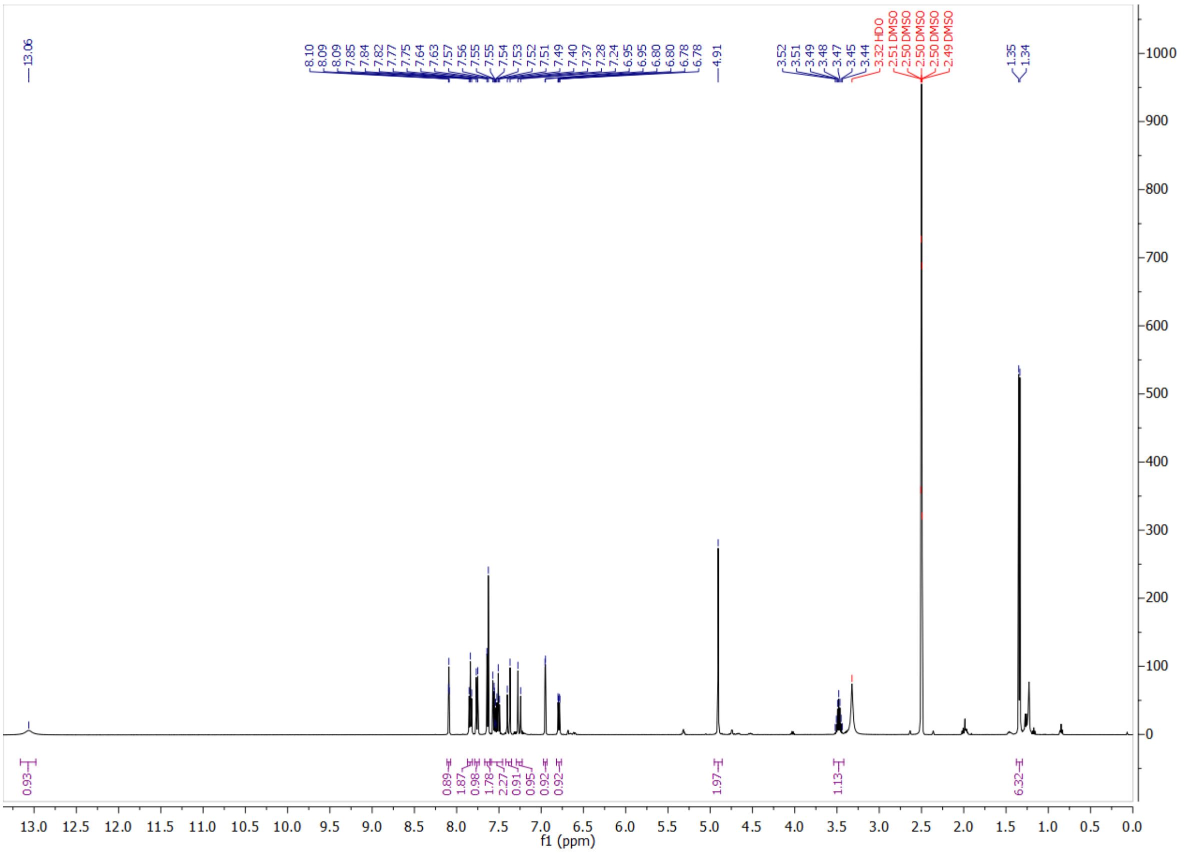

^{13}C NMR

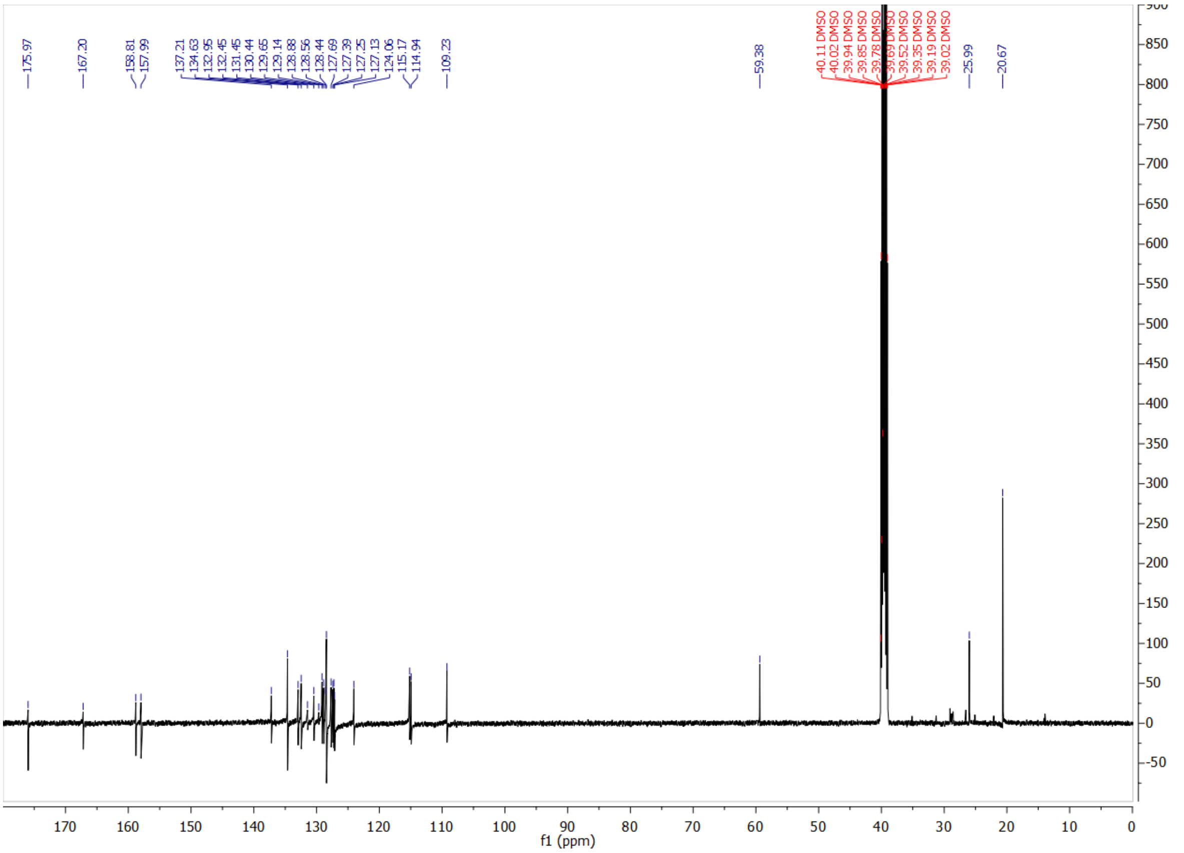

COMPOUND INFORMATION

Purity

Data File W:\analyti...OPEN\CGC_ECHO1-3_FirstPass 2021-03-20 13-21-54\038-D2B-D10-GW4064.D
Sample Name: GW4064

```
=====================================================================
Acq. Operator   : SYSTEM                        Seq. Line :  38
Sample Operator : SYSTEM
Acq. Instrument : LCMS test                      Location :   D2B-D10
Injection Date  : 3/20/2021 8:15:40 PM                Inj :   1
                                               Inj Volume : Inj prog
Sequence File   : W:\analytical_LCMS_DATA\EUbOPEN\CGC_ECHO1-3_FirstPass 2021-03-20 13-21-54
                  \CGC_ECHO1-3_FirstPass.S
Method          : W:\analytical_LCMS_DATA\EUbOPEN\CGC_ECHO1-3_FirstPass 2021-03-20 13-21-54
                  \CGL_FIRSTPASS_GENERALMETHOD_VIAL3+4_20210319.M (Sequence Method)
Last changed    : 3/19/2021 5:35:24 PM by SYSTEM
Method Info     : CGL wellplate, 0.5 uL of 10 mM DMSO, general method
```

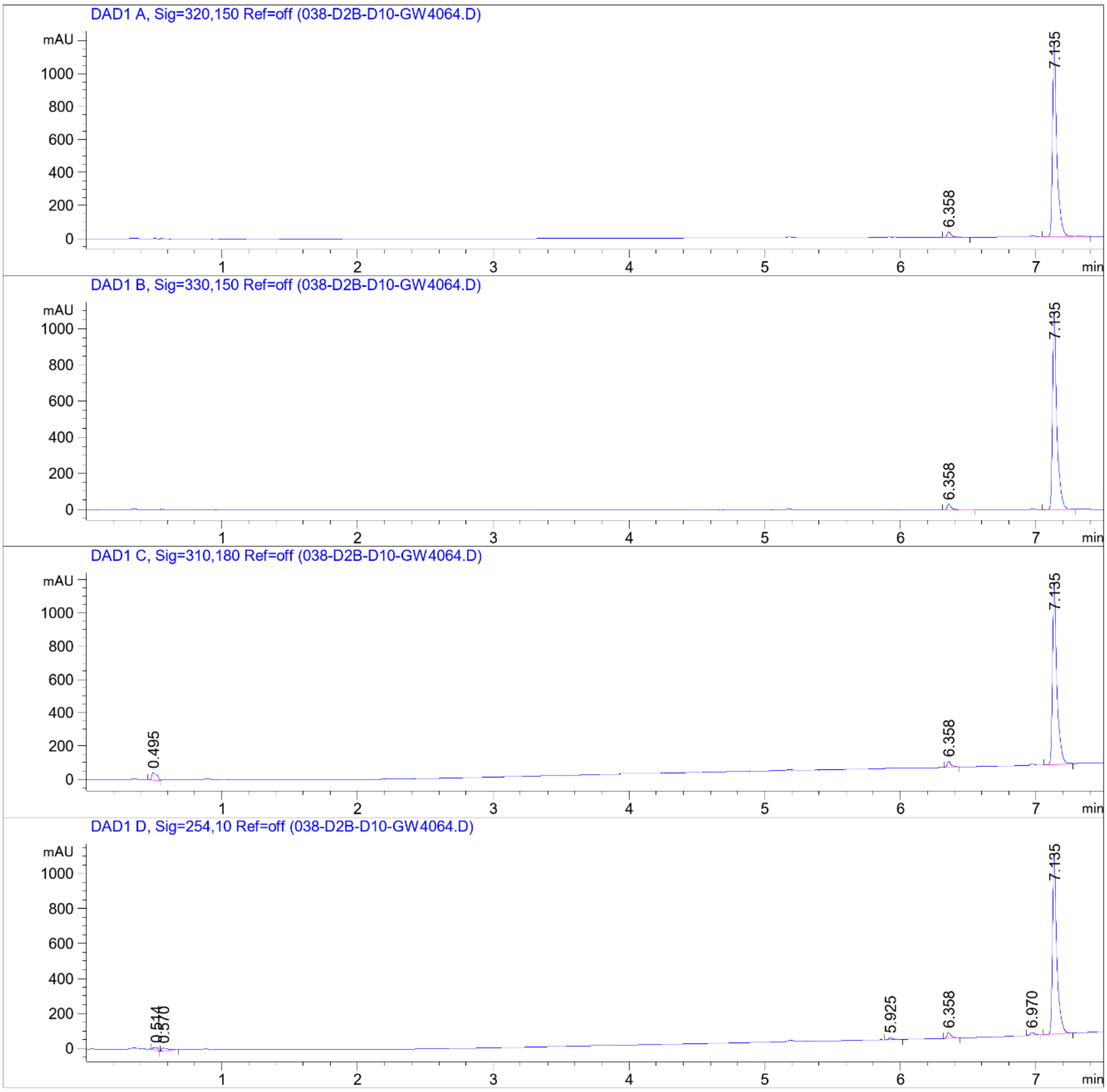

COMPOUND INFORMATION

Data File W:\analyti...OPEN\CGC_ECHO1-3_FirstPass 2021-03-20 13-21-54\038-D2B-D10-GW4064.D
Sample Name: GW4064

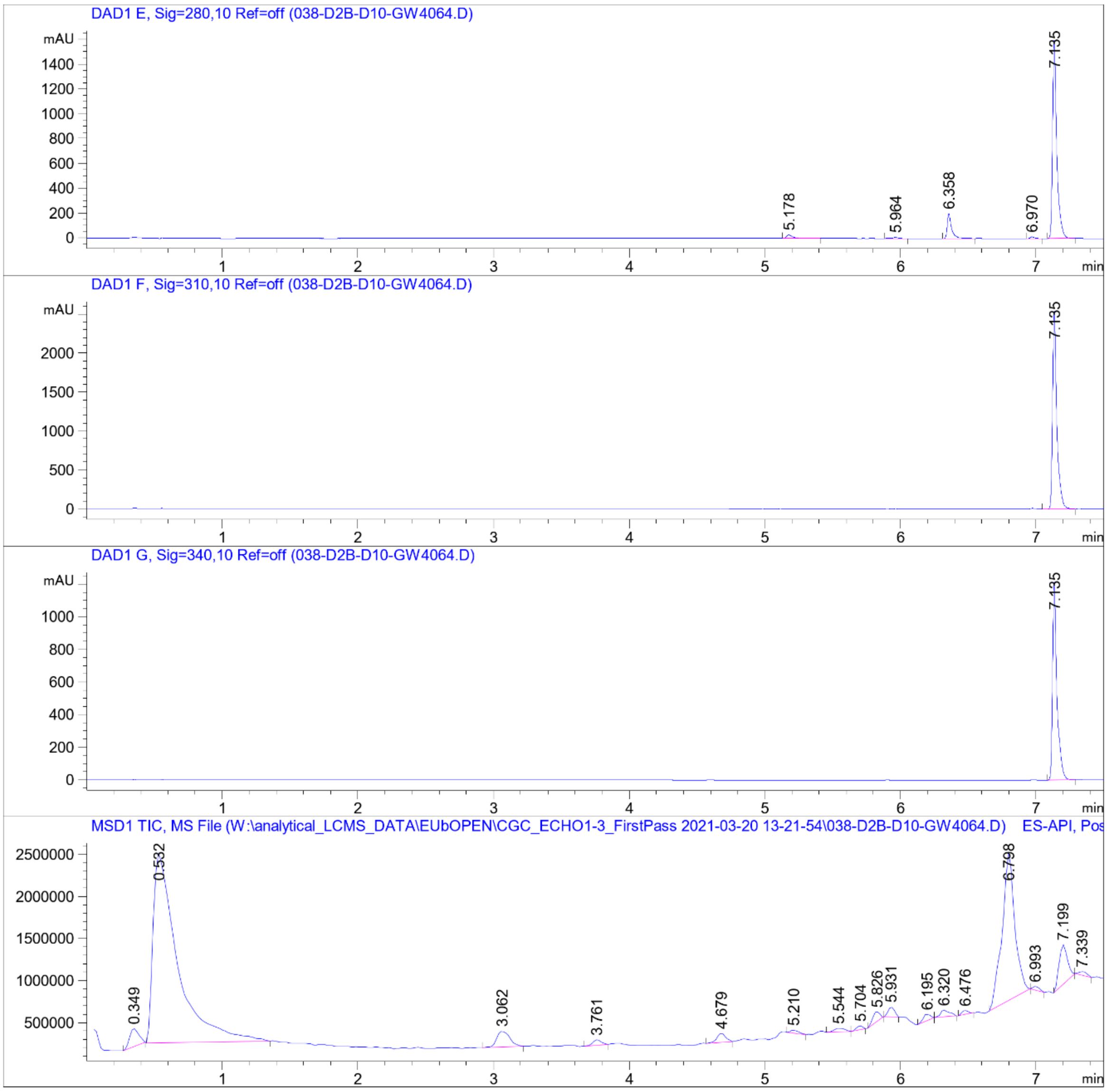

COMPOUND INFORMATION

Data File W:\analyti...OPEN\CGC_ECHO1-3_FirstPass 2021-03-20 13-21-54\038-D2B-D10-GW4064.D
Sample Name: GW4064

MS Signal: MSD1 TIC, MS File, ES-API, Pos, Scan, Frag: 70, "POS Scan"
Spectra from peak tops.
Noise Cutoff: 1000 counts.
Reportable Ion Abundance: > 50%.
LC Signal: DAD1 A, Sig=320,150 Ref=off
Peak matching window: 0.1 min

Retention Time (LC)	LC Area	Retention Time (MS)	MS Area	Mol. Weight or Ion
-	-	0.349	1009973	157.90 I
				130.00 I
-	-	0.532	28215774	157.00 I
-	-	3.062	1250510	217.10 I
-	-	3.761	285437	274.20 I
-	-	4.679	482301	326.30 I
-	-	5.210	153077	316.20 I
				102.20 I
-	-	5.544	237946	326.20 I
				280.20 I
				102.20 I
-	-	5.704	144632	280.20 I
-	-	5.826	437411	296.20 I
-	-	5.931	393297	296.20 I
				294.20 I
				280.20 I
-	-	6.195	295054	280.20 I
				228.20 I
6.358	75	6.320	387414	254.20 I
-	-	6.476	165237	280.20 I
-	-	6.798	11393991	282.20 I
-	-	6.993	153786	282.20 I
7.135	2622	7.199	2005478	544.10 I
				542.10 I
				284.20 I
				282.20 I
-	-	7.339	184484	400.30 I
				282.20 I

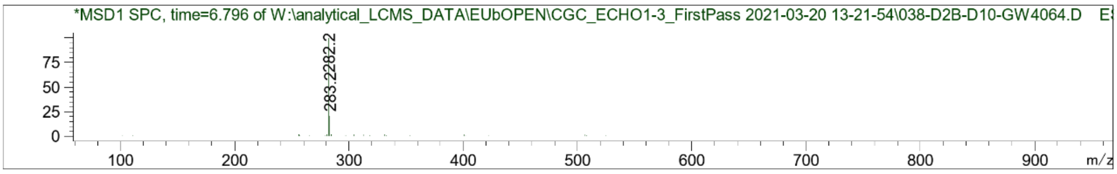

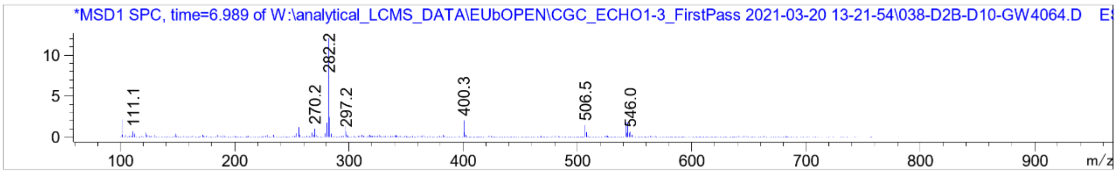

COMPOUND INFORMATION

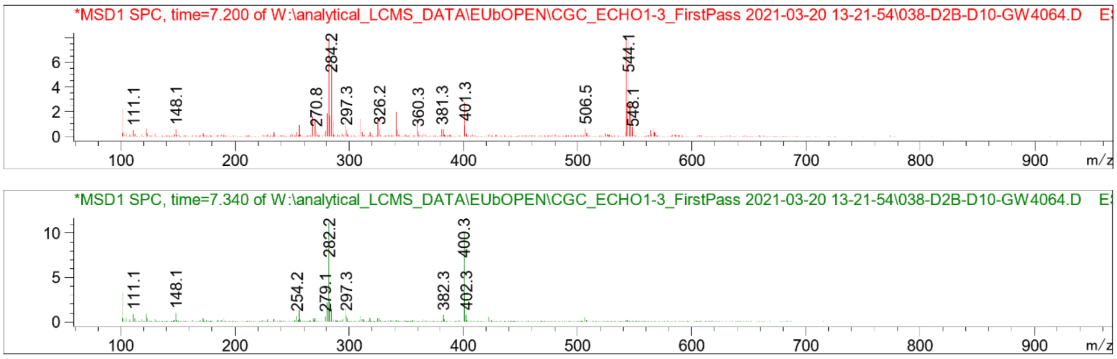

Biological activity

GW4064
FXR - EC_{50} 0.37 ± 0.03 μM
366 ± 11 fold activation

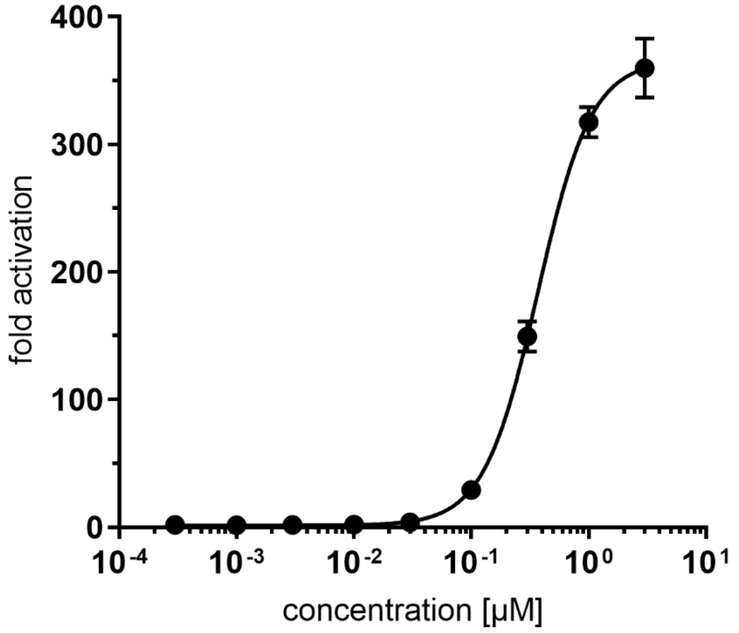

Supplement: Supplementary file 4 — Supplementary Data 1 [file 41467_2024_49493_MOESM4_ESM.zip › GW4064.pdf]
