# Supplementary material for: Chemogenomics for NR1 nuclear hormone receptors
Source: Nat Commun. 2024 Jun 18;15:5201. doi: 10.1038/s41467-024-49493-6 (PMC11189487; doi:10.1038/s41467-024-49493-6)

COMPOUND INFORMATION

GW501516

**CAS Registry No.:**	317318-70-0
**Formal Name:**	2-(2-methyl-4-(((4-methyl-2-(4-(trifluoromethyl)phenyl)thiazol-5-yl)methyl)thio)phenoxy)acetic acid
**EUbOPEN ID:**	EUB0001148a
**Molecular Formula:**	$C_{21}H_{18}F_3NO_3S_2$
**Molecular Weight:**	453.49 g/mol
**Smiles:**	CC1=C(C=CC(=C1)SCC2=C(N=C(S2)C3=CC=C(C=C3)C(F)(F)F)C)OCC(=O)O
**Recommended concentration:**	1 µM

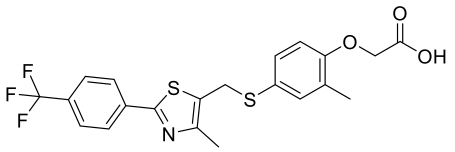

Biological activity

		Type	IC_{50}/EC_{50} [µM]	Reference
Main NR target:	NR1C2 (PPARδ)	Agonist	0.001	https://doi.org/10.1073/pnas.091021198
NR off-target:				

COMPOUND INFORMATION

Identity

¹H NMR

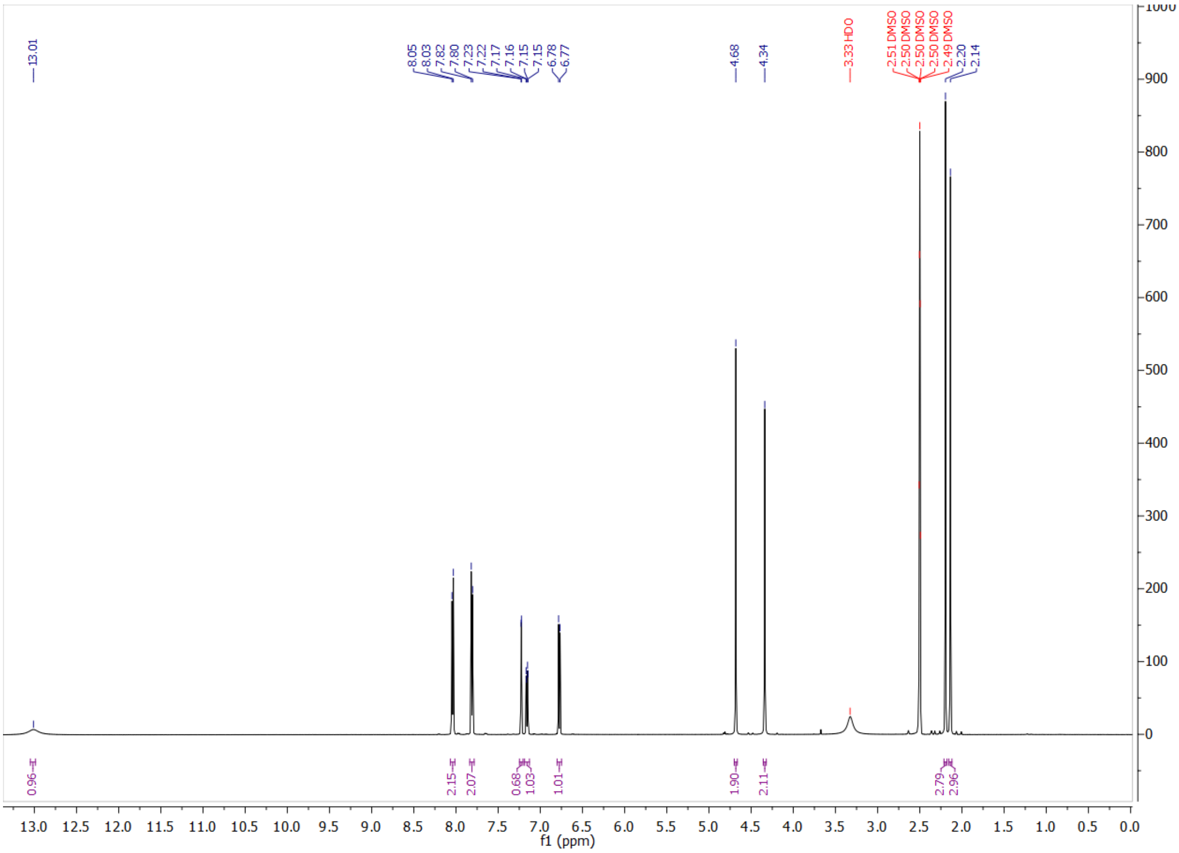

¹³C NMR

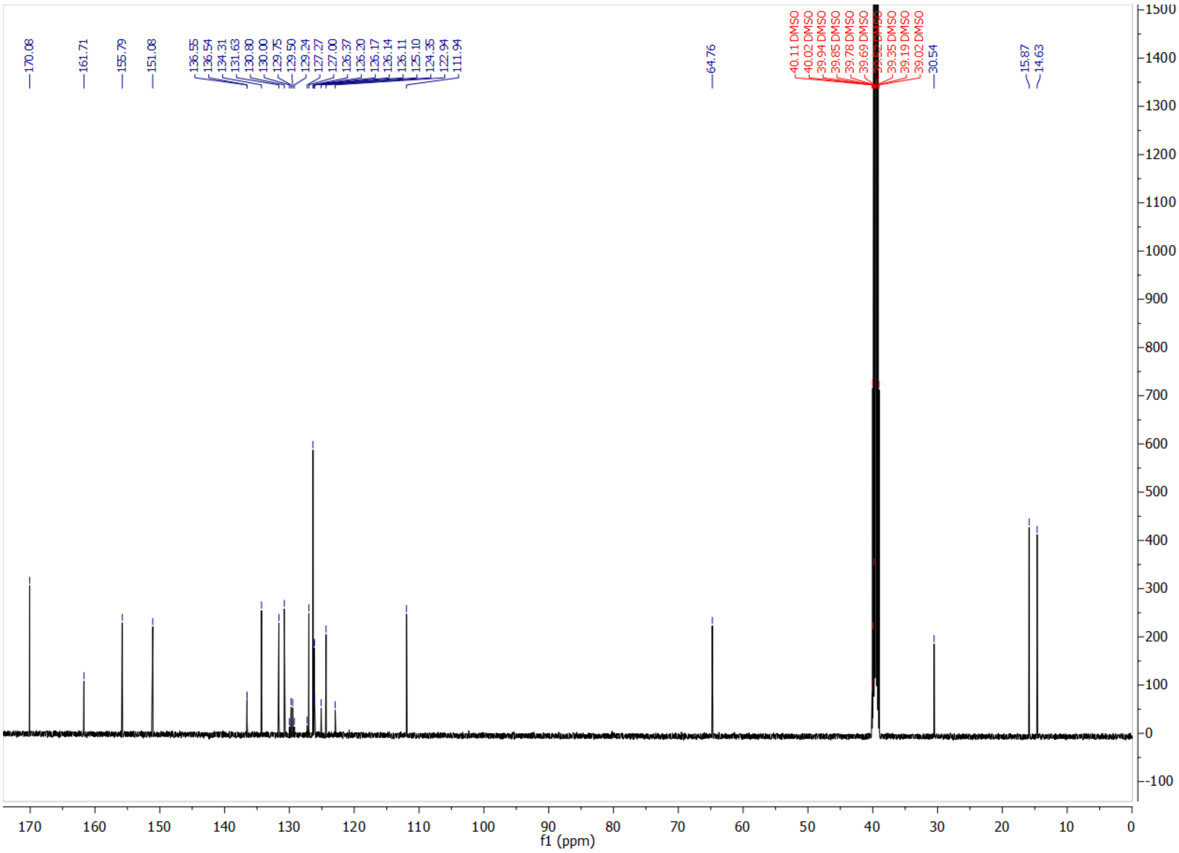

COMPOUND INFORMATION

Purity

Data File W:\analyti...N\CGC_wave3_1_FirstPassB 2023-01-04 18-28-02\069-D2F-F6-GW 501516.D
Sample Name: GW 501516

```
=====================================================================
Acq. Operator   : SYSTEM                          Seq. Line :  69
Sample Operator : SYSTEM
Acq. Instrument : LCMS test                        Location :  D2F-F6
Injection Date  : 1/5/2023 7:02:46 AM                   Inj :  1
                                                Inj Volume : Inj prog
Sequence File   : W:\analytical_LCMS_DATA\EUbOPEN\CGC_wave3_1_FirstPassB 2023-01-04 18-28-02
                  \CGC_wave3_1_FirstPassB.S
Method          : W:\analytical_LCMS_DATA\EUbOPEN\CGC_wave3_1_FirstPassB 2023-01-04 18-28-02
                  \CGL_FIRSTPASS_GENERALMETHOD_VIAL1+2_20210319.M (Sequence Method)
Last changed    : 1/25/2022 4:36:18 PM by SYSTEM
Method Info     : CGL wellplate, 0.5 uL of 10 mM DMSO, general method
```

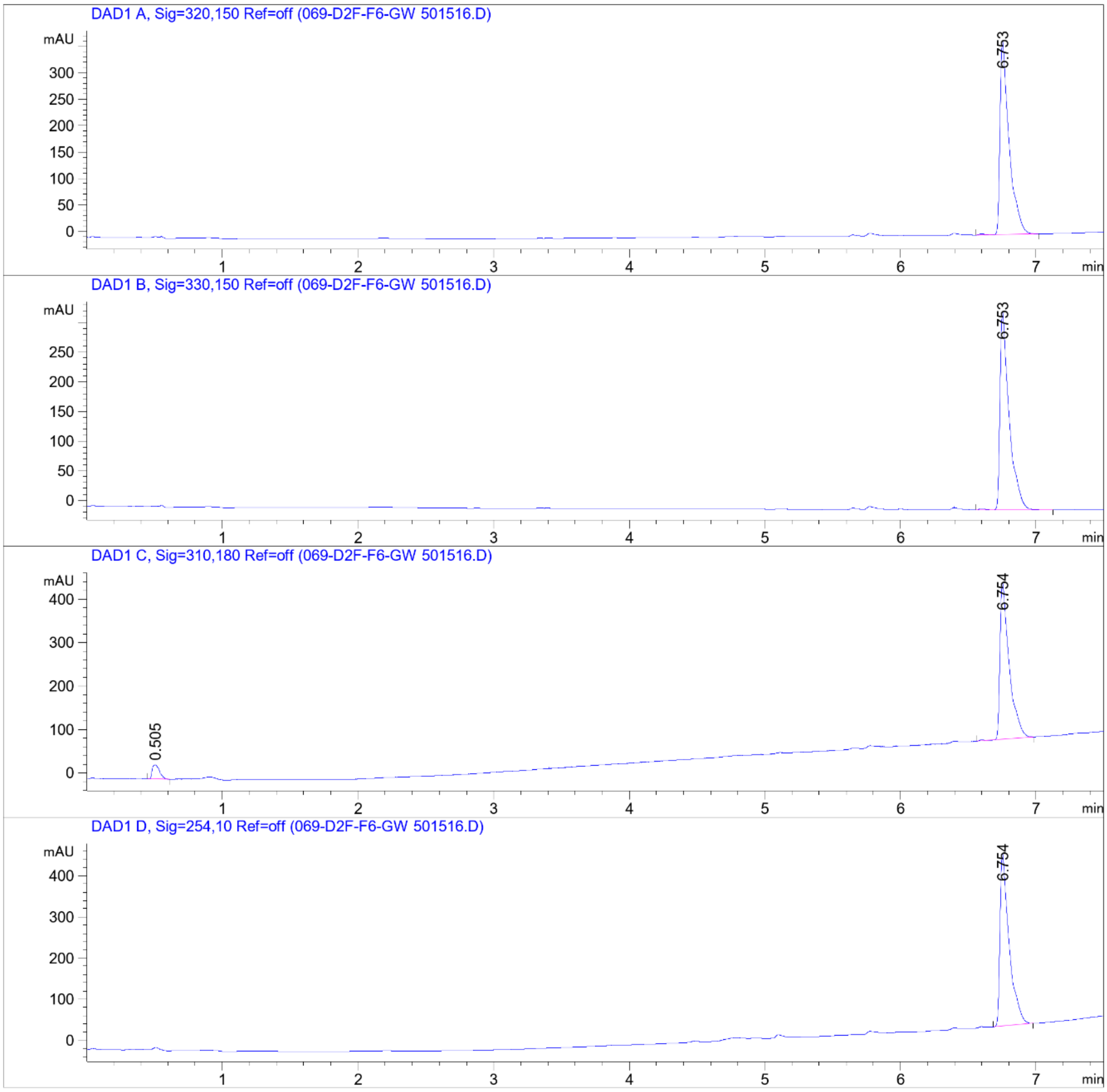

COMPOUND INFORMATION

Data File W:\analyti...N\CGC_wave3_1_FirstPassB 2023-01-04 18-28-02\069-D2F-F6-GW 501516.D
Sample Name: GW 501516

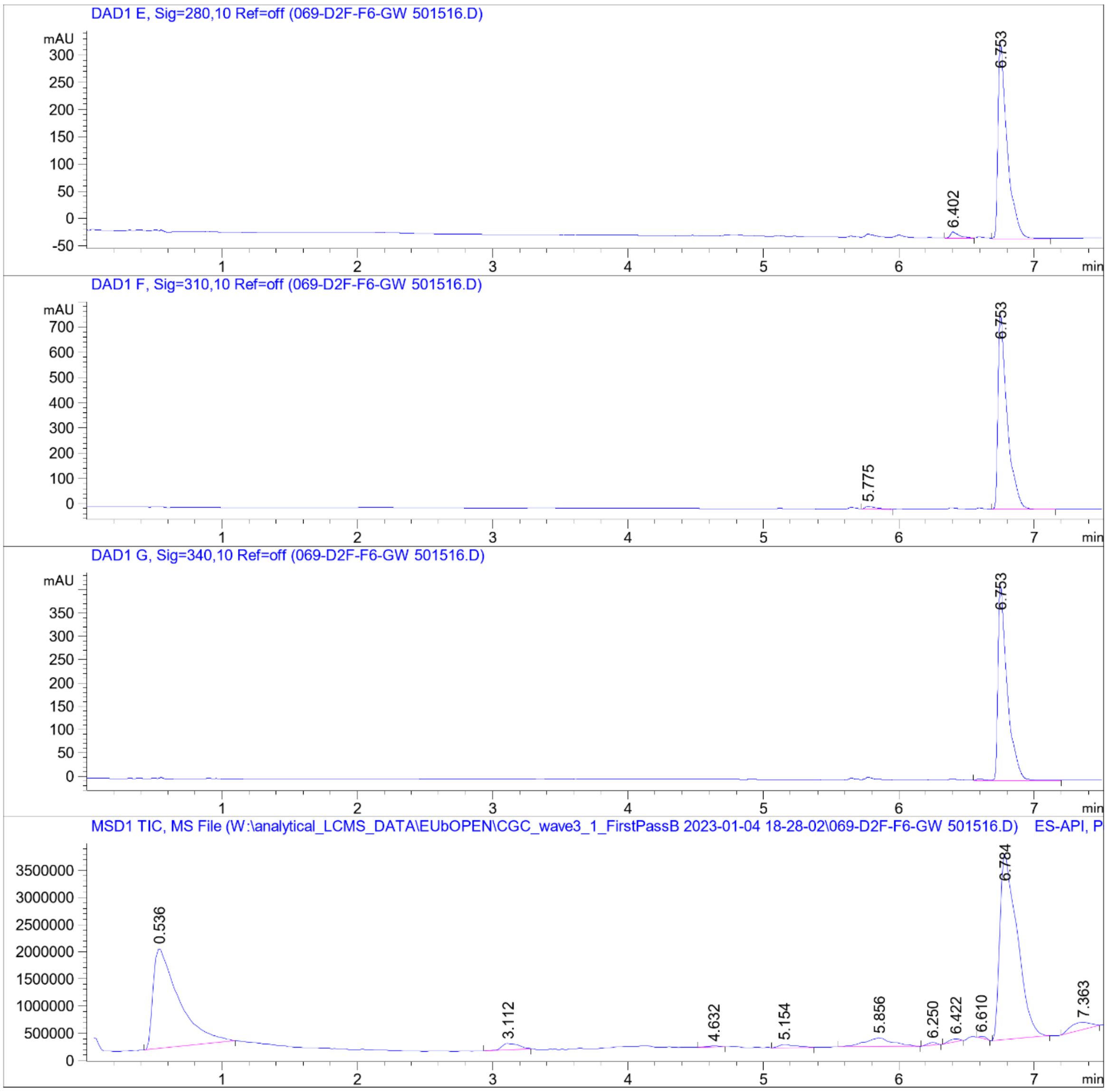

COMPOUND INFORMATION

Data File W:\analyti...N\CGC_wave3_1_FirstPassB 2023-01-04 18-28-02\069-D2F-F6-GW 501516.D
Sample Name: GW 501516

MS Signal: MSD1 TIC, MS File, ES-API, Pos, Scan, Frag: 70, "POS Scan"
Spectra from peak tops.
Noise Cutoff: 1000 counts.
Reportable Ion Abundance: > 50%.
LC Signal: DAD1 A, Sig=320,150 Ref=off
Peak matching window: 0.1 min

Retention Time (LC)	LC Area	Retention Time (MS)	MS Area	Mol. Weight or Ion
-	-	0.536	24070416	157.00 I
-	-	3.112	1019263	239.00 I
				217.10 I
-	-	4.632	164508	510.40 I
				170.80 I
				158.10 I
-	-	5.154	578058	510.30 I
				274.00 I
				170.90 I
				137.10 I
-	-	5.856	2261523	318.20 I
				296.20 I
-	-	6.250	216604	228.20 I
				137.10 I
-	-	6.422	326089	436.10 I
				350.20 I
				282.30 I
				254.20 I
				137.20 I
-	-	6.610	133779	507.30 I
				468.00 I
				280.20 I
6.753	1709	6.784	30612550	454.10 I
-	-	7.363	1397508	400.30 I
				282.30 I

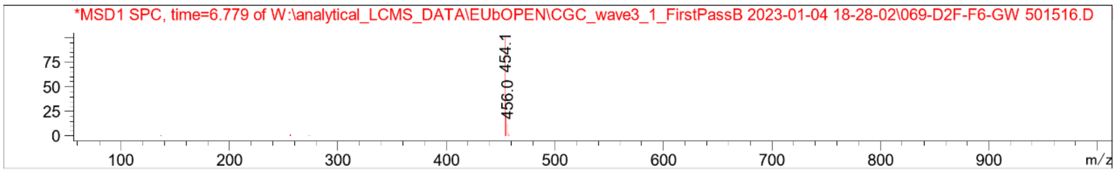

Supplement: Supplementary file 4 — Supplementary Data 1 [file 41467_2024_49493_MOESM4_ESM.zip › GW501516.pdf]
