# Supplementary material for: Chemogenomics for NR1 nuclear hormone receptors
Source: Nat Commun. 2024 Jun 18;15:5201. doi: 10.1038/s41467-024-49493-6 (PMC11189487; doi:10.1038/s41467-024-49493-6)

COMPOUND INFORMATION

GW590735

**CAS Registry No.:**	622402-22-6
**Formal Name:**	2-methyl-2-(4-((4-methyl-2-(4-(trifluoromethyl)phenyl)thiazole-5-carboxamido)methyl)phenoxy)propanoic acid
**EUbOPEN ID:**	EUB0001141a
**Molecular Formula:**	$C_{23}H_{21}F_3N_2O_4S$
**Molecular Weight:**	478.49 g/mol
**Smiles:**	CC1=C(SC(=N1)C2=CC=C(C=C2)C(F)(F)F)C(=O)NCC3=CC=C(C=C3)OC(C)(C)C(=O)O
**Recommended concentration:**	1 µM

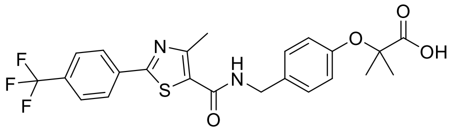

Biological activity

		Type	IC_{50}/EC_{50} [µM]	Reference
Main NR target:	NR1C1 (PPARα)	Agonist	0.004	https://doi.org/10.1021/jm058056x, https://doi.org/10.1016/j.bmcl.2008.09.094
	NR1C2 (PPARδ)	Agonist	0.15	inhouse
NR off-target:				

COMPOUND INFORMATION

Identity

^{1}H NMR

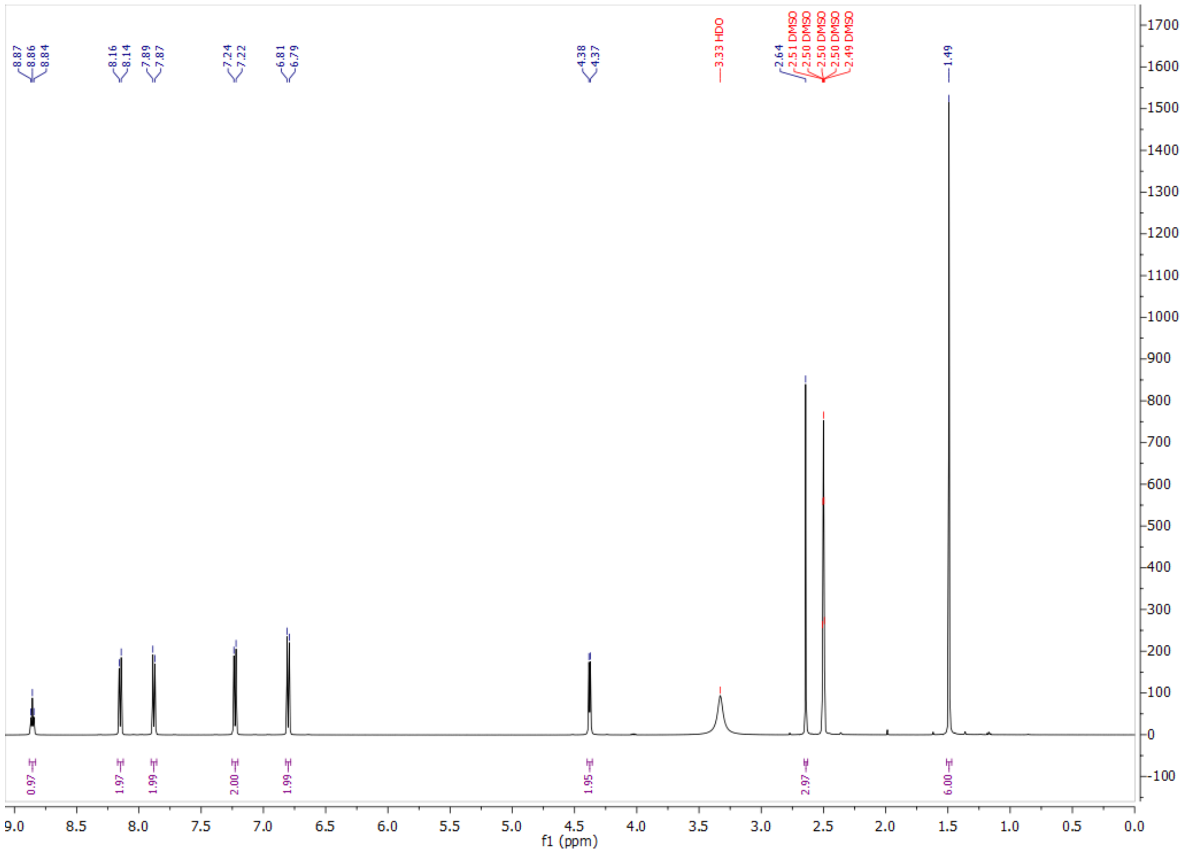

^{13}C NMR

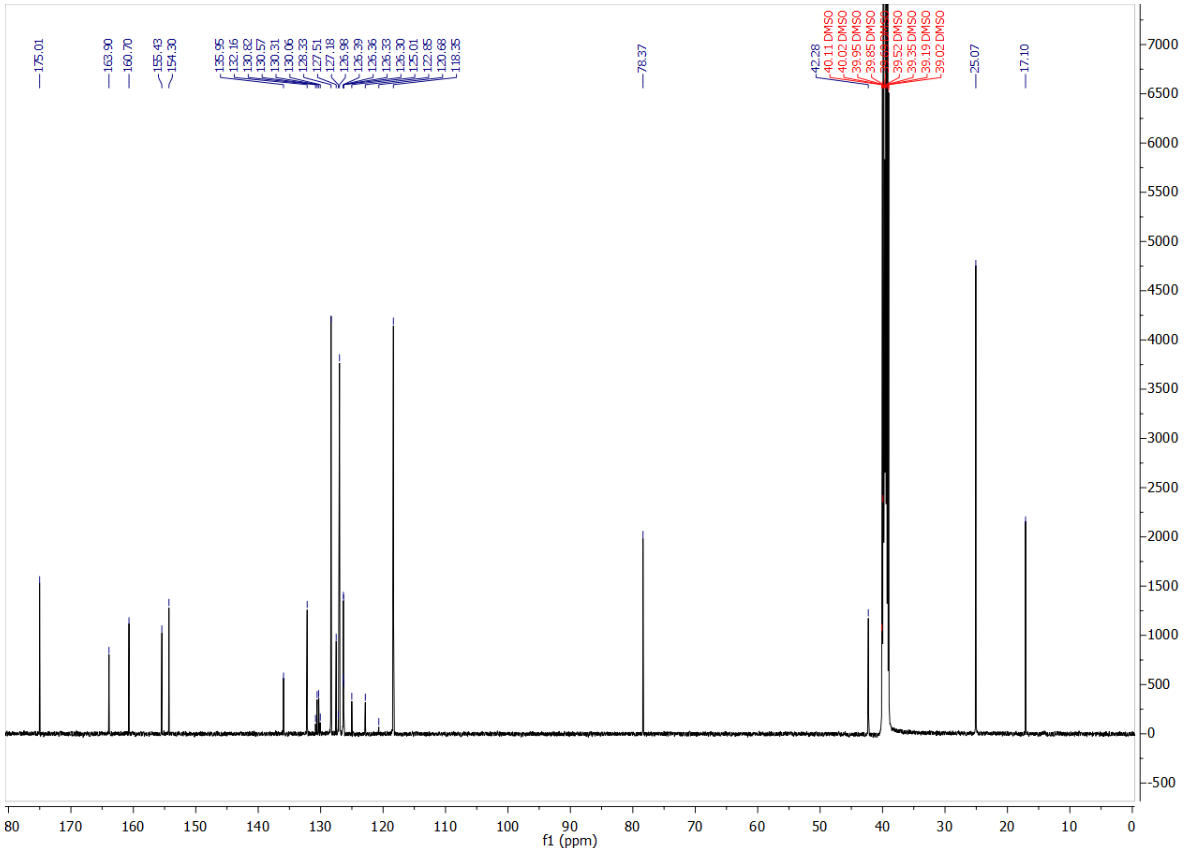

COMPOUND INFORMATION

Purity

Data File W:\analyti...N\CGC_wave3_1_FirstPassB 2023-01-04 18-28-02\051-D2F-E11-GW590735.D
Sample Name: GW590735

```
=====================================================================
Acq. Operator   : SYSTEM                          Seq. Line :  51
Sample Operator : SYSTEM
Acq. Instrument : LCMS test                        Location :  D2F-E11
Injection Date  : 1/5/2023 3:43:44 AM                   Inj :  1
                                                Inj Volume : Inj prog
Sequence File   : W:\analytical_LCMS_DATA\EUbOPEN\CGC_wave3_1_FirstPassB 2023-01-04 18-28-02
                  \CGC_wave3_1_FirstPassB.S
Method          : W:\analytical_LCMS_DATA\EUbOPEN\CGC_wave3_1_FirstPassB 2023-01-04 18-28-02
                  \CGL_FIRSTPASS_GENERALMETHOD_VIAL1+2_20210319.M (Sequence Method)
Last changed    : 1/25/2022 4:36:18 PM by SYSTEM
Method Info     : CGL wellplate, 0.5 uL of 10 mM DMSO, general method
```

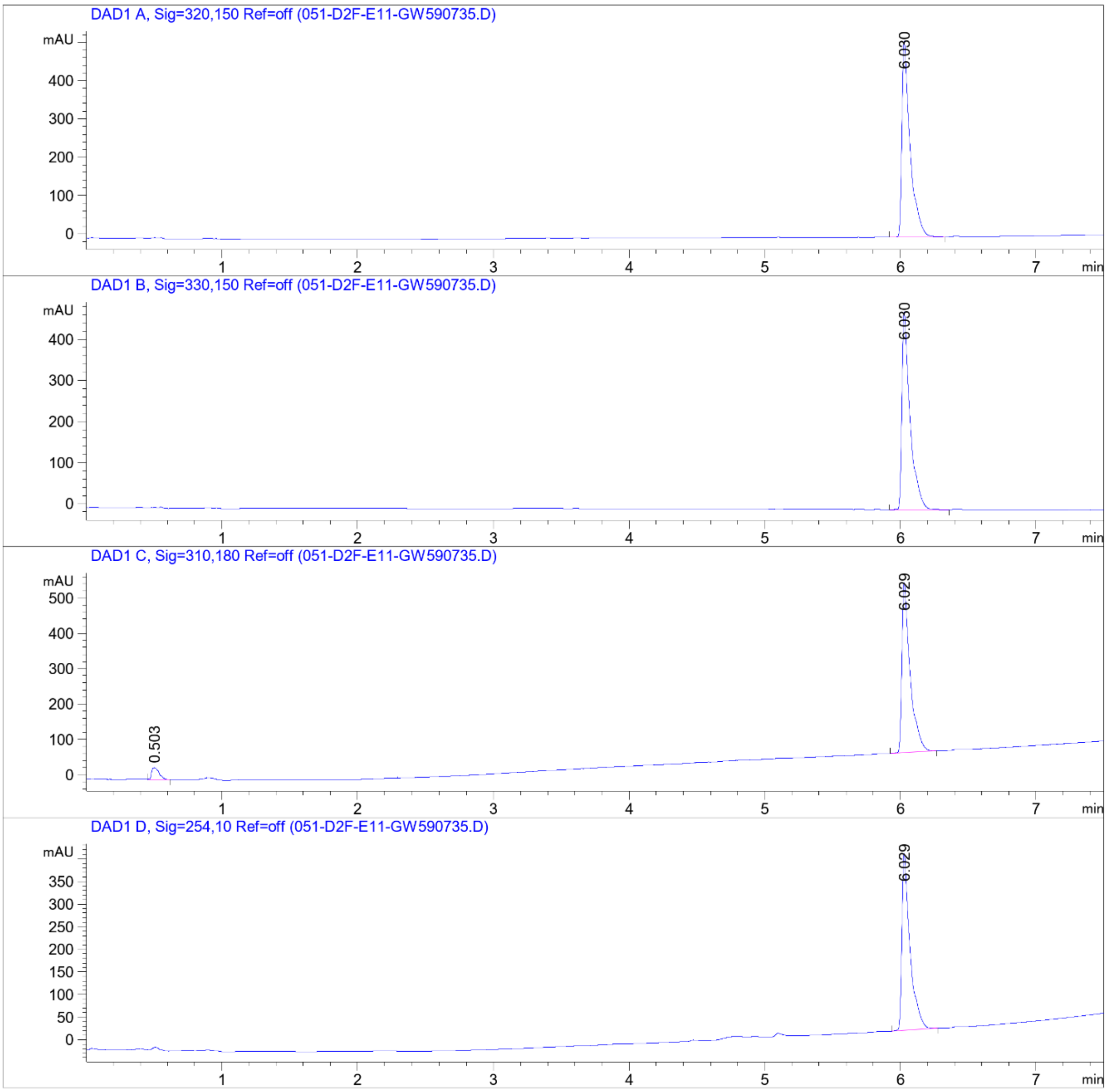

COMPOUND INFORMATION

Data File W:\analyti...N\CGC_wave3_1_FirstPassB 2023-01-04 18-28-02\051-D2F-E11-GW590735.D
Sample Name: GW590735

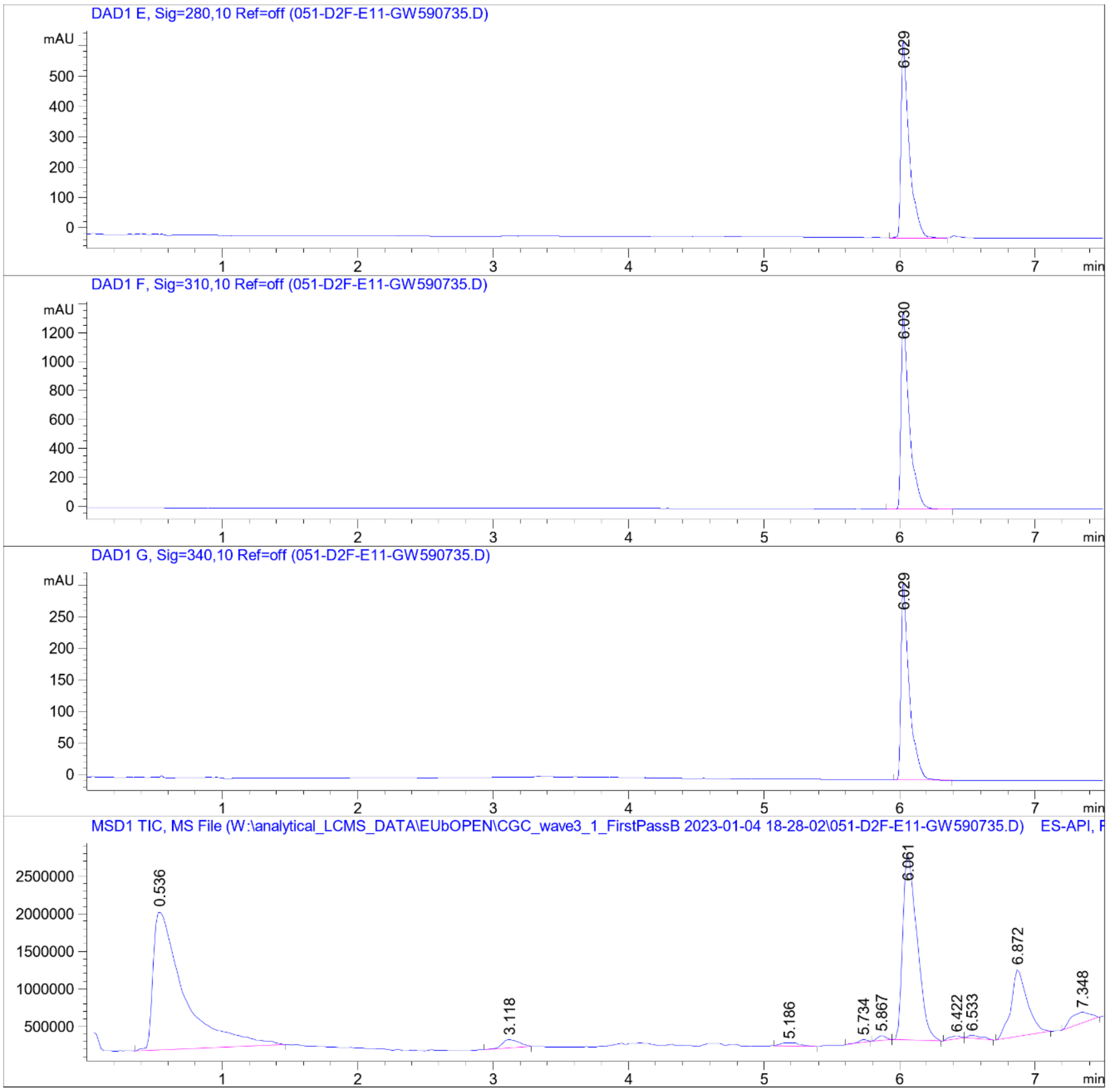

COMPOUND INFORMATION

Data File W:\analyti...N\CGC_wave3_1_FirstPassB 2023-01-04 18-28-02\051-D2F-E11-GW590735.D
Sample Name: GW590735

MS Signal: MSD1 TIC, MS File, ES-API, Pos, Scan, Frag: 70, "POS Scan"
Spectra from peak tops.
Noise Cutoff: 1000 counts.
Reportable Ion Abundance: > 50%.
LC Signal: DAD1 A, Sig=320,150 Ref=off
Peak matching window: 0.1 min

Retention Time (LC)	LC Area	Retention Time (MS)	MS Area	Mol. Weight or Ion
-	-	0.536	29029588	157.00 I
-	-	3.118	968117	239.10 I
				217.10 I
-	-	5.186	435414	510.40 I
				170.90 I
				137.10 I
-	-	5.734	149111	280.20 I
-	-	5.867	285303	318.20 I
				296.20 I
6.030	2127	6.061	18284772	479.10 I
-	-	6.422	227439	350.20 I
				282.20 I
				254.20 I
				137.10 I
-	-	6.533	278988	280.20 I
-	-	6.872	7389712	282.20 I
-	-	7.348	1466862	400.30 I
				282.30 I

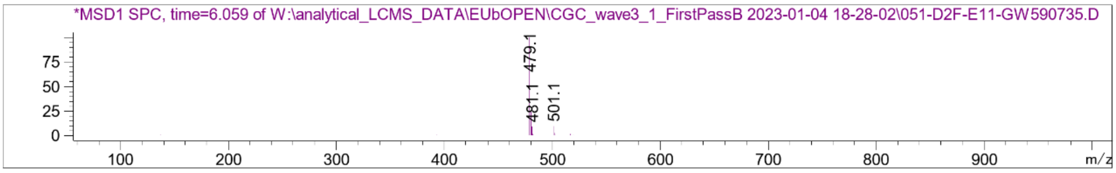

Biological activity

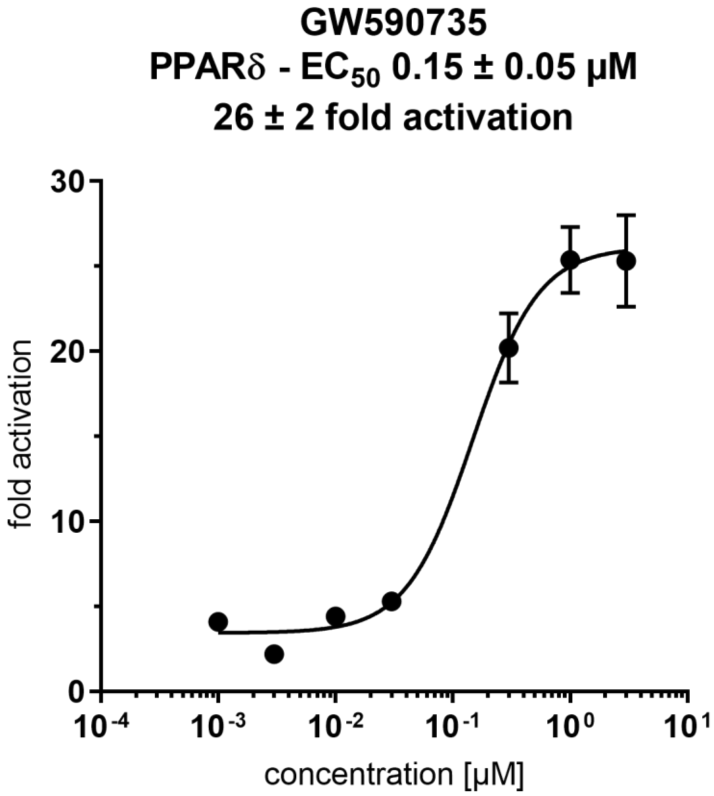

Supplement: Supplementary file 4 — Supplementary Data 1 [file 41467_2024_49493_MOESM4_ESM.zip › GW590735.pdf]
