# Supplementary material for: Chemogenomics for NR1 nuclear hormone receptors
Source: Nat Commun. 2024 Jun 18;15:5201. doi: 10.1038/s41467-024-49493-6 (PMC11189487; doi:10.1038/s41467-024-49493-6)

COMPOUND INFORMATION

GW7647

**CAS Registry No.:**	265129-71-3
**Formal Name:**	2-((4-(2-(3-cyclohexyl-1-(4-cyclohexylbutyl)ureido)ethyl)phenyl)thio)-2-methylpropanoic acid
**EUbOPEN ID:**	EUB0000185b
**Molecular Formula:**	$C_{29}H_{46}N_2O_3S$
**Molecular Weight:**	502.76 g/mol
**Smiles:**	CC(C)(C(=O)O)SC1=CC=C(C=C1)CCN(CCCCC2CCCCC2)C(=O)NC3CCCCC3
**Recommended concentration:**	1 µM

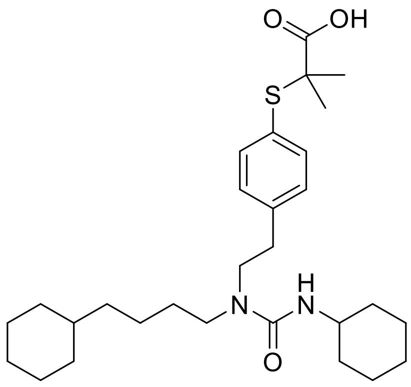

Biological activity

		Type	IC_{50}/EC_{50} [µM]	Reference
Main NR target:	NR1C1 (PPARα)	Agonist	0.006	https://doi.org/10.1016/S0960-894X(01)00188-3
	NR1C3 (PPARγ)	Agonist	1	
NR off-target:	NR1C2 (PPARδ)	Agonist	6	https://doi.org/10.1016/S0960-894X(01)00188-3

COMPOUND INFORMATION

Identity

^{1}H NMR

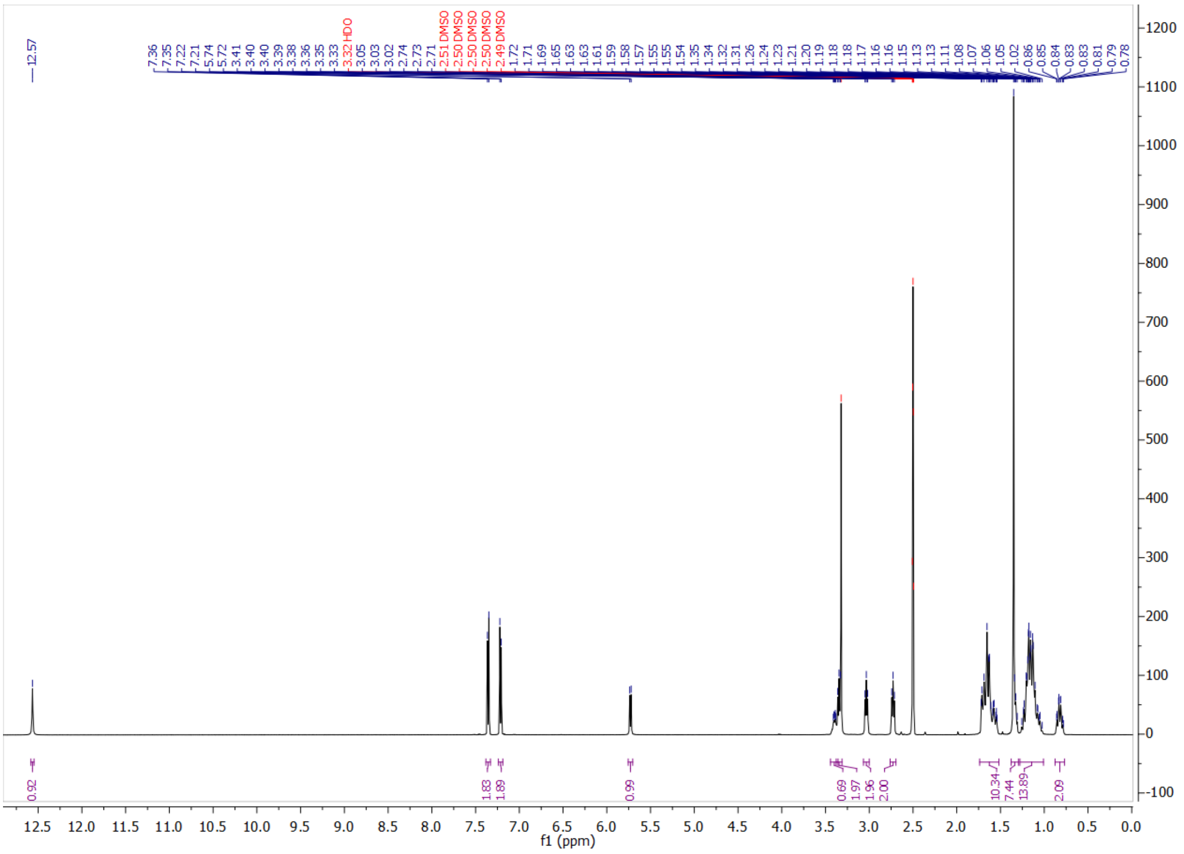

^{13}C NMR

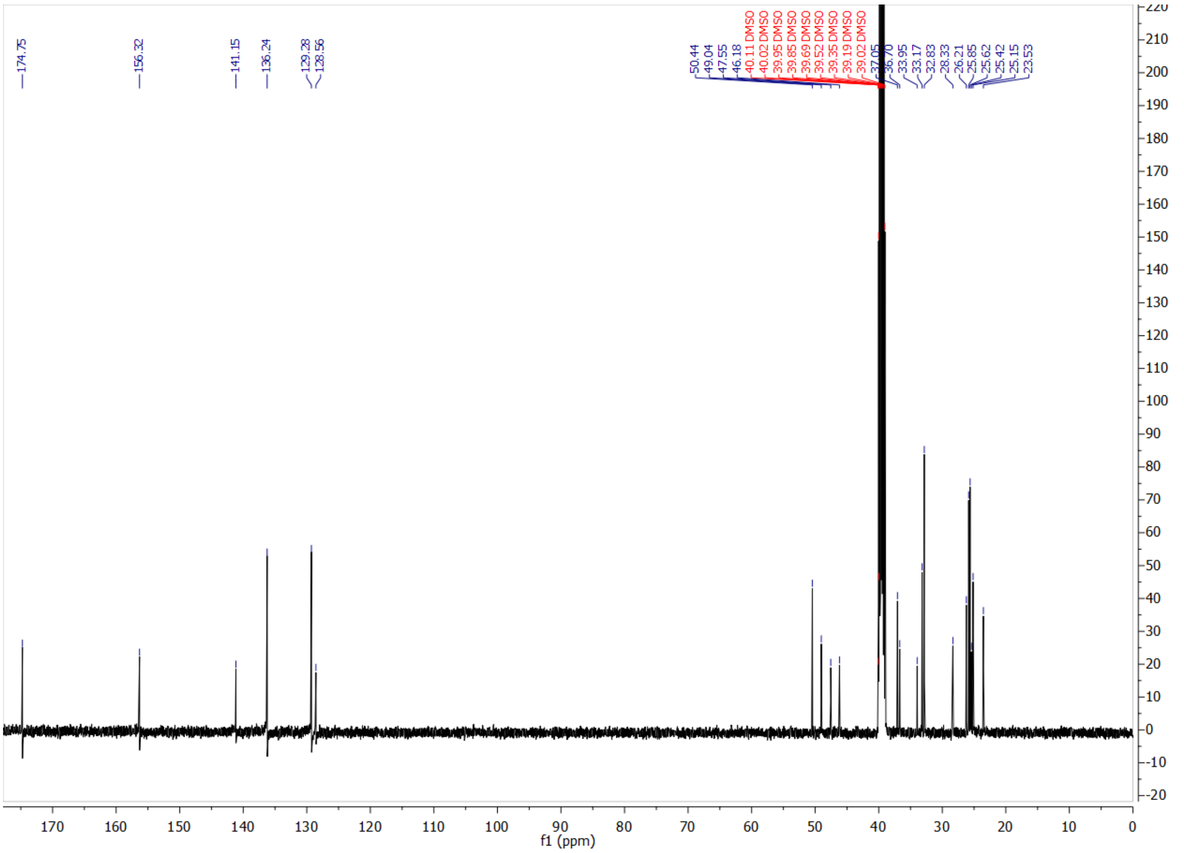

COMPOUND INFORMATION

Purity

```
Data File W:\analyti...OPEN\CGC_ECHO1-3_SecondPass 2021-03-25 18-09-21\004-D2F-B4-GW7647.D
Sample Name: GW7647

    =====================================================================
    Acq. Operator   : SYSTEM                          Seq. Line :    4
    Sample Operator : SYSTEM
    Acq. Instrument : LCMS test                        Location :   D2F-B4
    Injection Date  : 3/25/2021 6:45:18 PM                  Inj :    1
                                                     Inj Volume : Inj prog
    Sequence File   : W:\analytical_LCMS_DATA\EUbOPEN\CGC_ECHO1-3_SecondPass 2021-03-25 18-09-21
                      \CGC_ECHO1-3_SecondPass.S
    Method          : W:\analytical_LCMS_DATA\EUbOPEN\CGC_ECHO1-3_SecondPass 2021-03-25 18-09-21
                      \CGL_SECONDPASS_NONPOLCOMP_VIAL2+4_20210323.M (Sequence Method)
    Last changed    : 3/25/2021 4:32:02 PM by SYSTEM
    Method Info     : CGL wellplate, 0.5 uL of 10 mM DMSO. Dilution with MeCN only (9+9 uL)
```

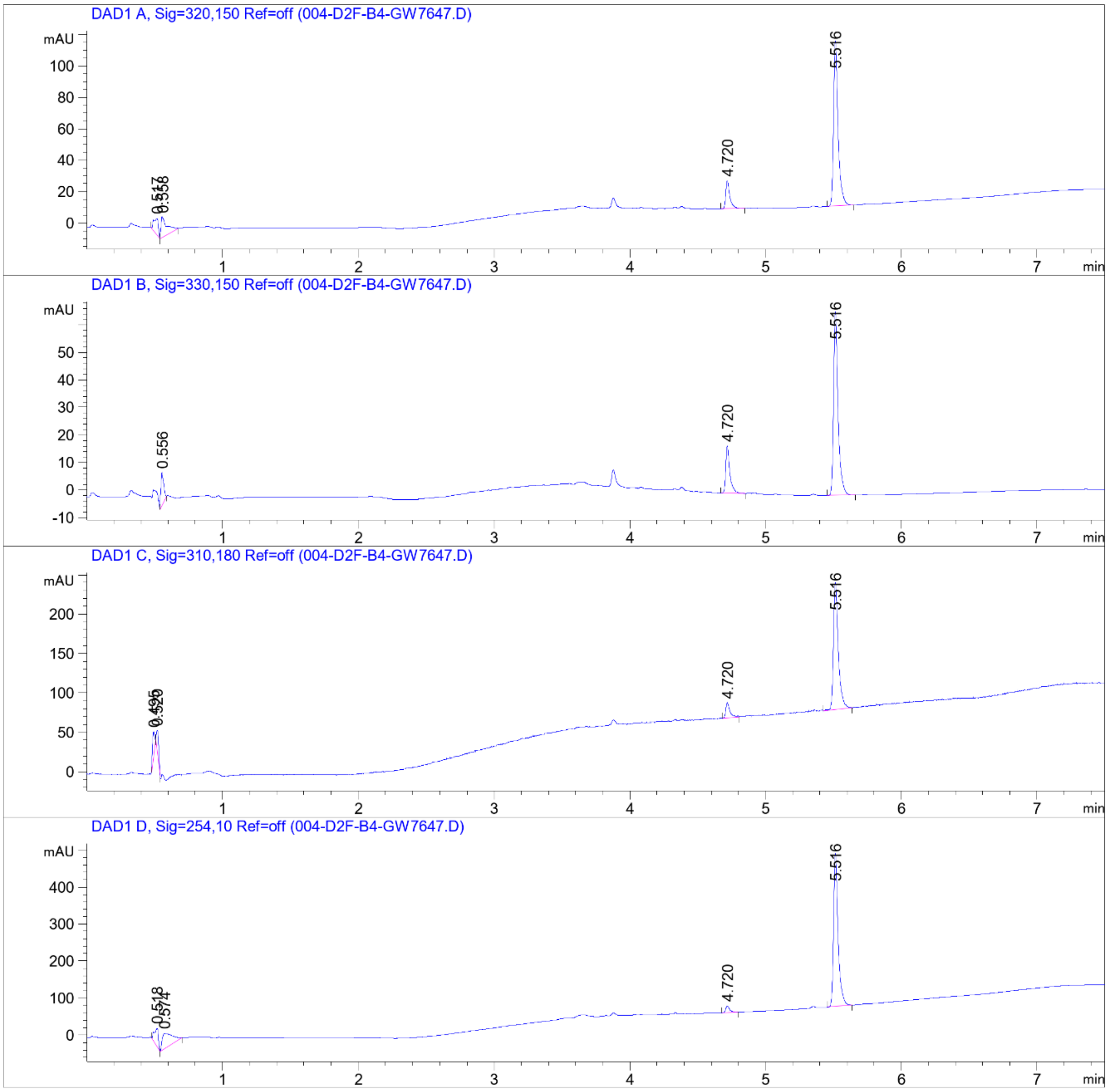

COMPOUND INFORMATION

Data File W:\analyti...OPEN\CGC_ECHO1-3_SecondPass 2021-03-25 18-09-21\004-D2F-B4-GW7647.D
Sample Name: GW7647

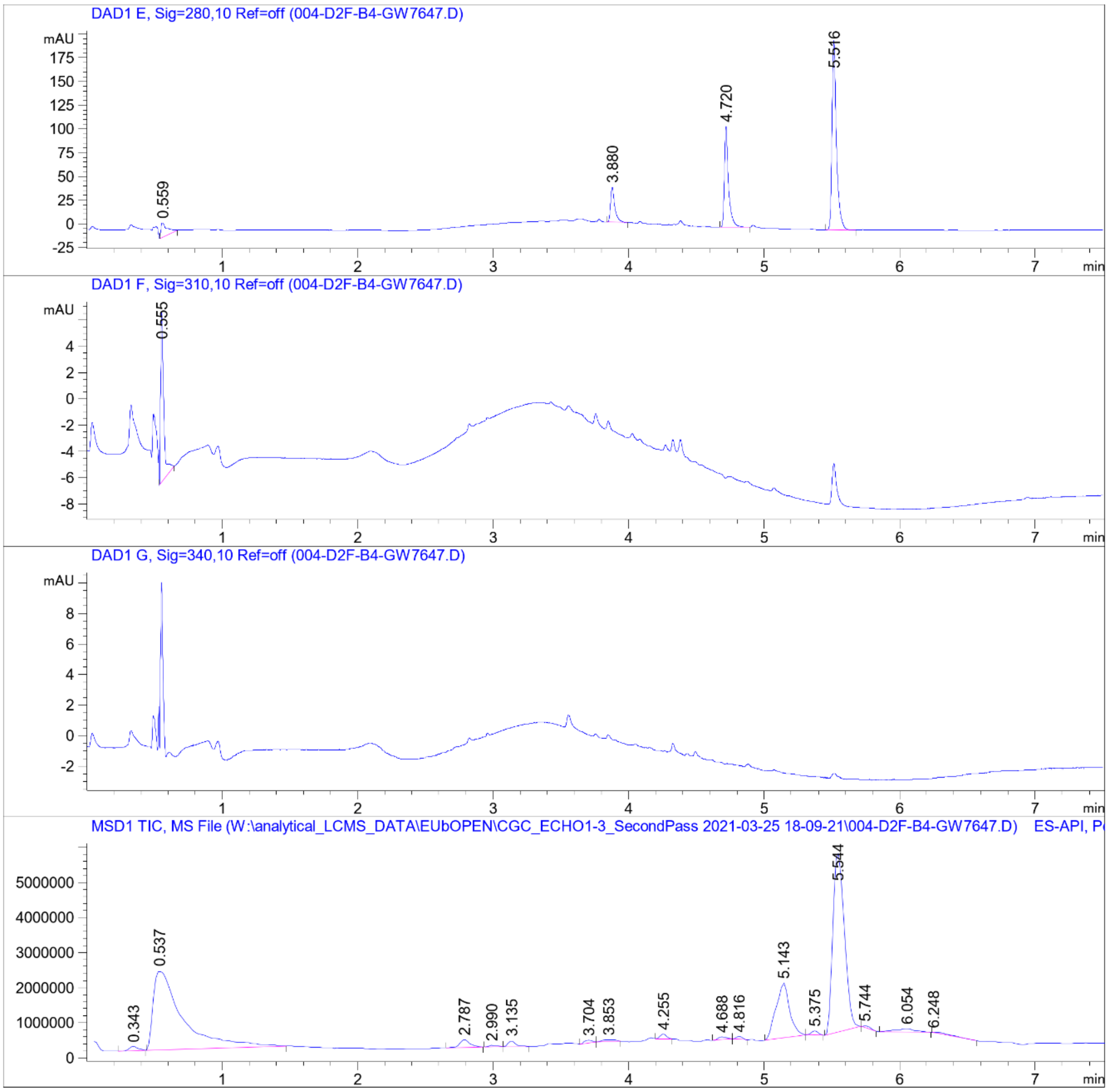

COMPOUND INFORMATION


```
Data File W:\analyti...OPEN\CGC_ECHO1-3_SecondPass 2021-03-25 18-09-21\004-D2F-B4-GW7647.D
Sample Name: GW7647

    MS Signal: MSD1 TIC, MS File,  ES-API, Pos, Scan, Frag: 70, "POS Scan"
        Spectra from peak tops.
        Noise Cutoff: 1000 counts.
        Reportable Ion Abundance: > 50%.
    LC Signal: DAD1 A, Sig=320,150 Ref=off
        Peak matching window: 0.1  min
```

Retention Time (LC)	LC Area	Retention Time (MS)	MS Area	Mol. Weight or Ion
-	-	0.343	531811	200.00 I
				182.90 I
				159.00 I
				142.00 I
0.517	24	0.537	34150540	157.00 I
0.558	39	-	-	
-	-	2.787	1203455	217.10 I
-	-	2.990	187435	274.20 I
-	-	3.135	689244	378.30 I
-	-	3.704	311274	312.20 I
				214.10 I
				111.10 I
-	-	3.853	356953	316.30 I
				298.20 I
				288.20 I
				282.30 I
				252.20 I
				225.20 I
				200.00 I
				159.00 I
				111.10 I
				110.10 I
-	-	4.255	445303	296.30 I
4.720	37	4.688	338247	254.30 I
-	-	4.816	230855	280.30 I
-	-	5.143	10786898	282.30 I
-	-	5.375	423401	489.40 I
				282.30 I
5.516	241	5.544	29970410	503.40 I
-	-	5.744	218435	400.40 I
				282.20 I
-	-	6.054	1001885	282.30 I

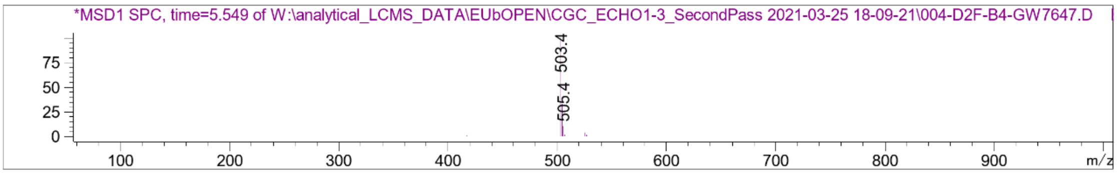

Supplement: Supplementary file 4 — Supplementary Data 1 [file 41467_2024_49493_MOESM4_ESM.zip › GW7647.pdf]
