# Supplementary material for: Chemogenomics for NR1 nuclear hormone receptors
Source: Nat Commun. 2024 Jun 18;15:5201. doi: 10.1038/s41467-024-49493-6 (PMC11189487; doi:10.1038/s41467-024-49493-6)

COMPOUND INFORMATION

GW9578

**CAS Registry No.:**	247923-29-1
**Formal Name:**	2-((4-(2-(3-(2,4-difluorophenyl)-1-heptylureido)ethyl)phenyl)thio)-2-methylpropanoic acid
**EUbOPEN ID:**	EUB0001142a
**Molecular Formula:**	$C_{26}H_{34}F_2N_2O_3S$
**Molecular Weight:**	492.63 g/mol
**Smiles:**	CCCCCCCN(CCC1=CC=C(C=C1)SC(C)(C)C(=O)O)C(=O)NC2=C(C=C(C=C2)F)F
**Recommended concentration:**	1 µM

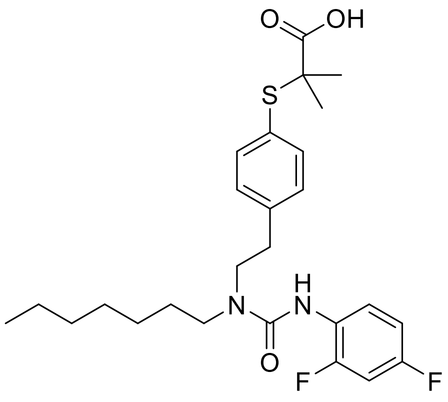

Biological activity

		Type	IC_{50}/EC_{50} [µM]	Reference
Main NR target:	NR1C1 (PPARα)	Agonist	0.05	https://doi.org/10.1021/jm9903601
	NR1C3 (PPARγ)	Agonist	1	
NR off-target:	NR1C2 (PPARδ)	Agonist	1	https://doi.org/10.1021/jm9903601

COMPOUND INFORMATION

Identity

¹H NMR

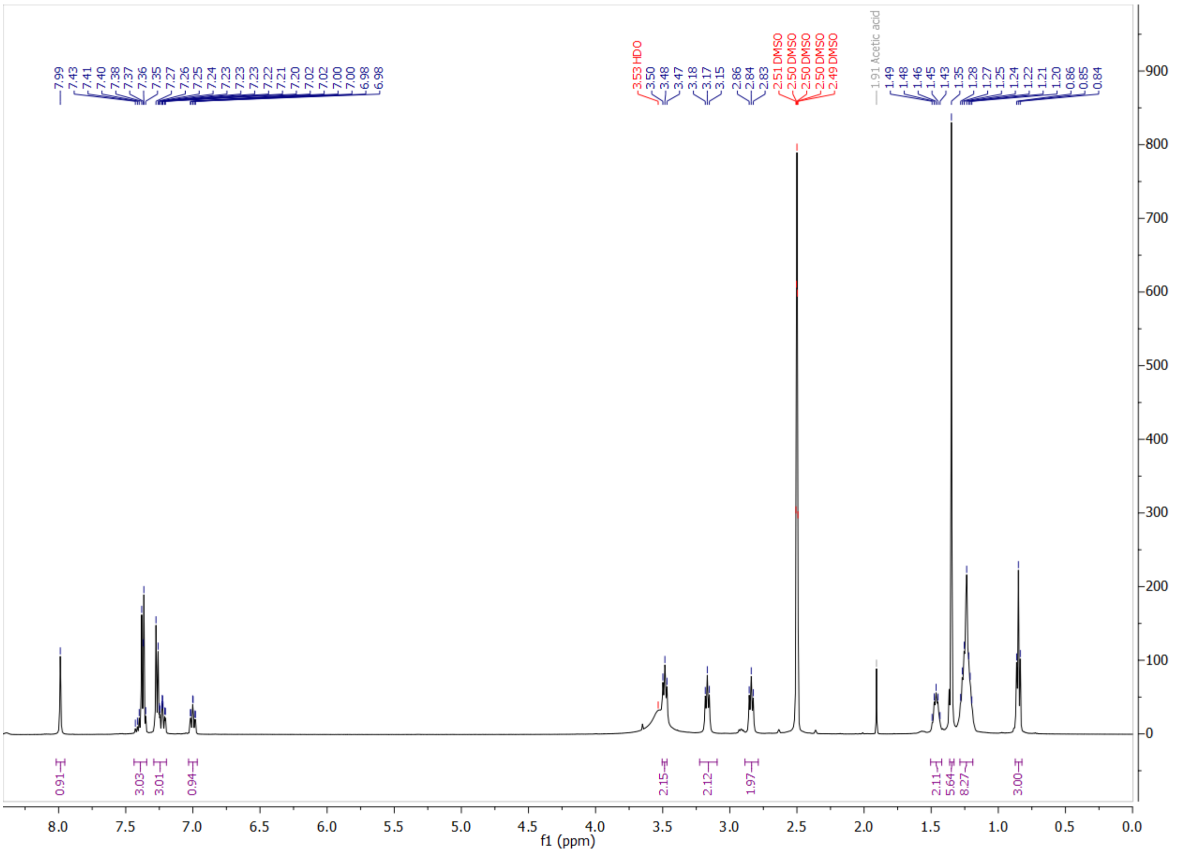

¹³C NMR

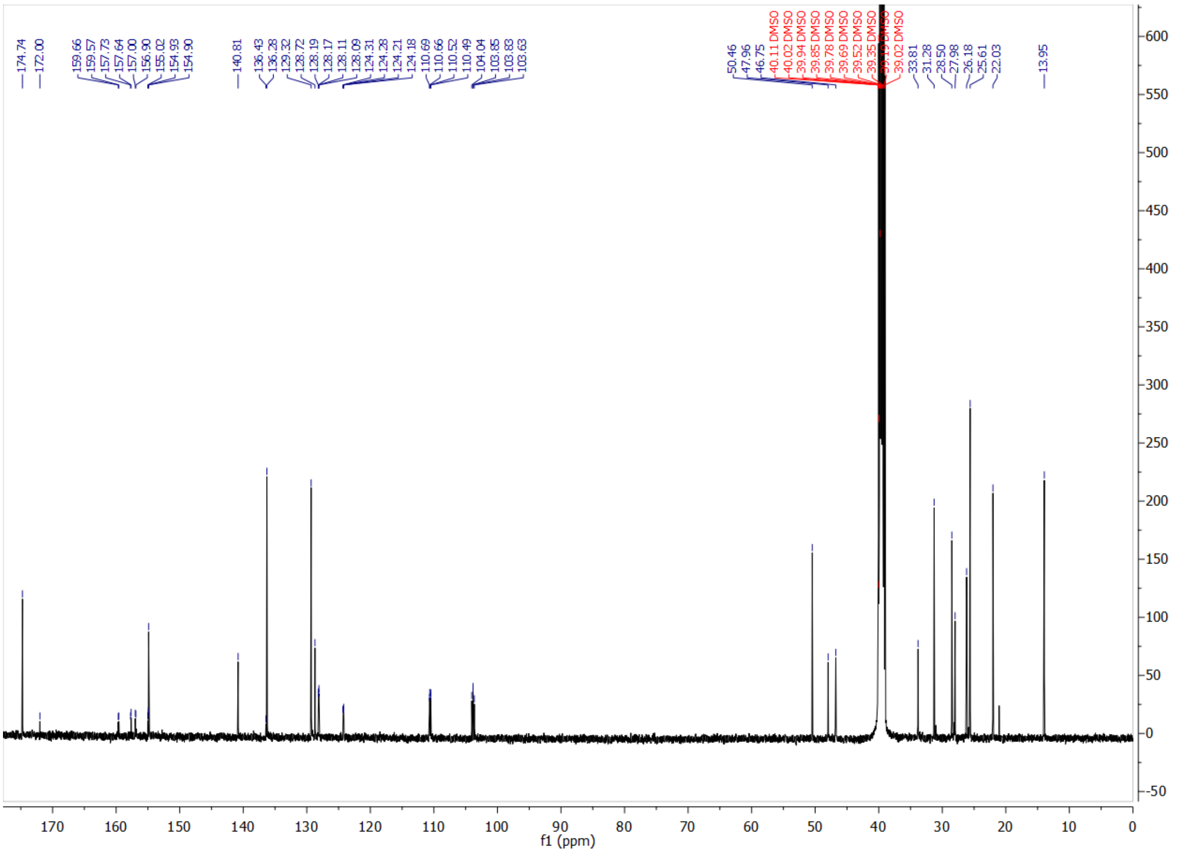

COMPOUND INFORMATION

Purity

```
Data File W:\analyti...PEN\CGC_wave3_1_SecondPass 2023-01-19 20-16-18\021-D2F-E12-GW9578.D
Sample Name: GW9578

    =====================================================================
    Acq. Operator   : SYSTEM                          Seq. Line :  21
    Sample Operator : SYSTEM
    Acq. Instrument : LCMS test                        Location :   D2F-E12
    Injection Date  : 1/20/2023 12:04:33 AM                 Inj :    1
                                                     Inj Volume : Inj prog
    Sequence File   : W:\analytical_LCMS_DATA\EUbOPEN\CGC_wave3_1_SecondPass 2023-01-19 20-16-18
                      \CGC_wave3_1_SecondPass.S
    Method          : W:\analytical_LCMS_DATA\EUbOPEN\CGC_wave3_1_SecondPass 2023-01-19 20-16-18
                      \CGL_SECONDPASS_NONPOLCOMP_VIAL2+4_20210323.M (Sequence Method)
    Last changed    : 7/18/2022 10:07:35 AM by SYSTEM
    Method Info     : CGL wellplate, 0.5 uL of 10 mM DMSO. Dilution with MeCN only (9+9 uL)
```

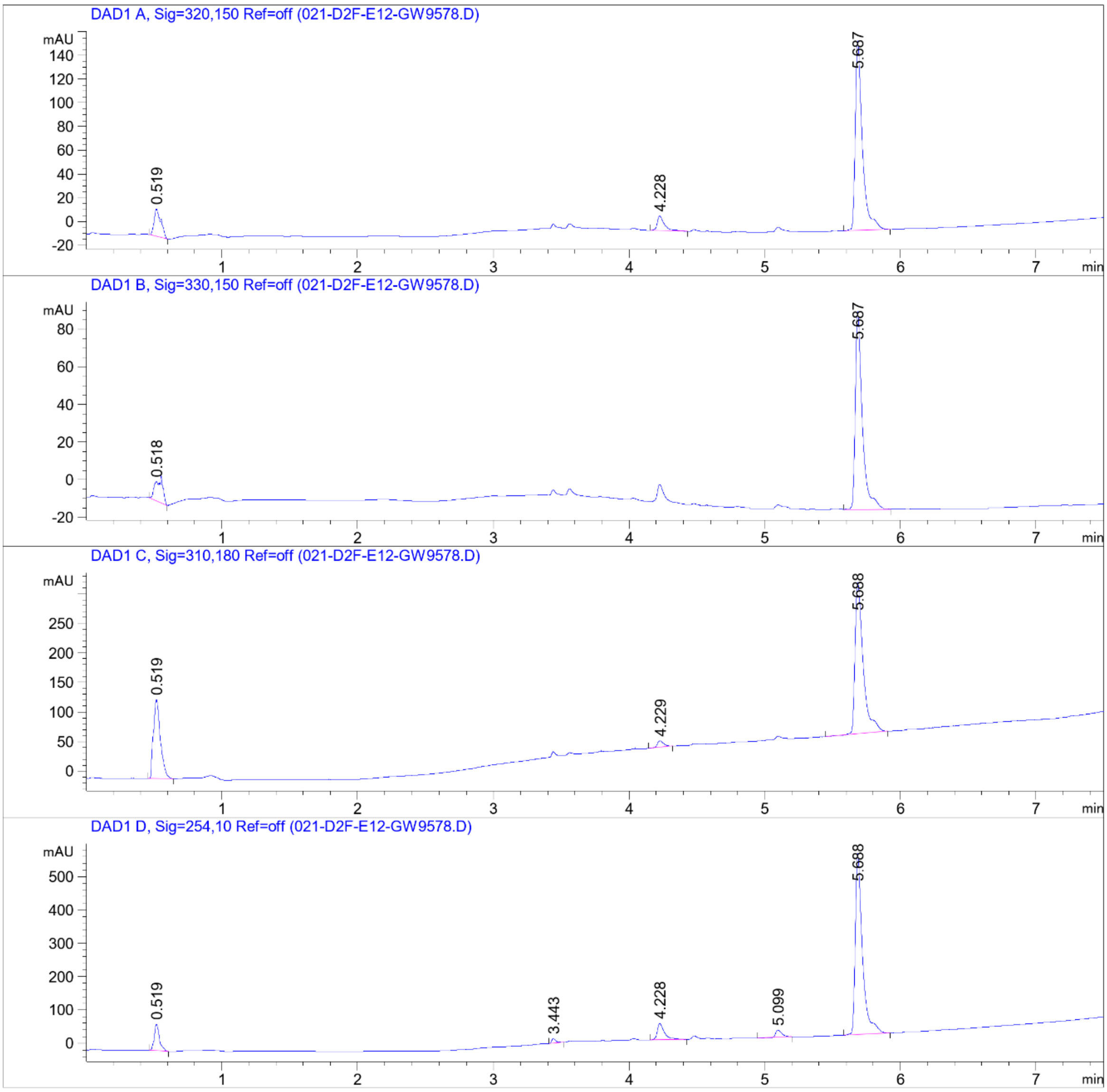

COMPOUND INFORMATION

Data File W:\analyti...PEN\CGC_wave3_1_SecondPass 2023-01-19 20-16-18\021-D2F-E12-GW9578.D
Sample Name: GW9578

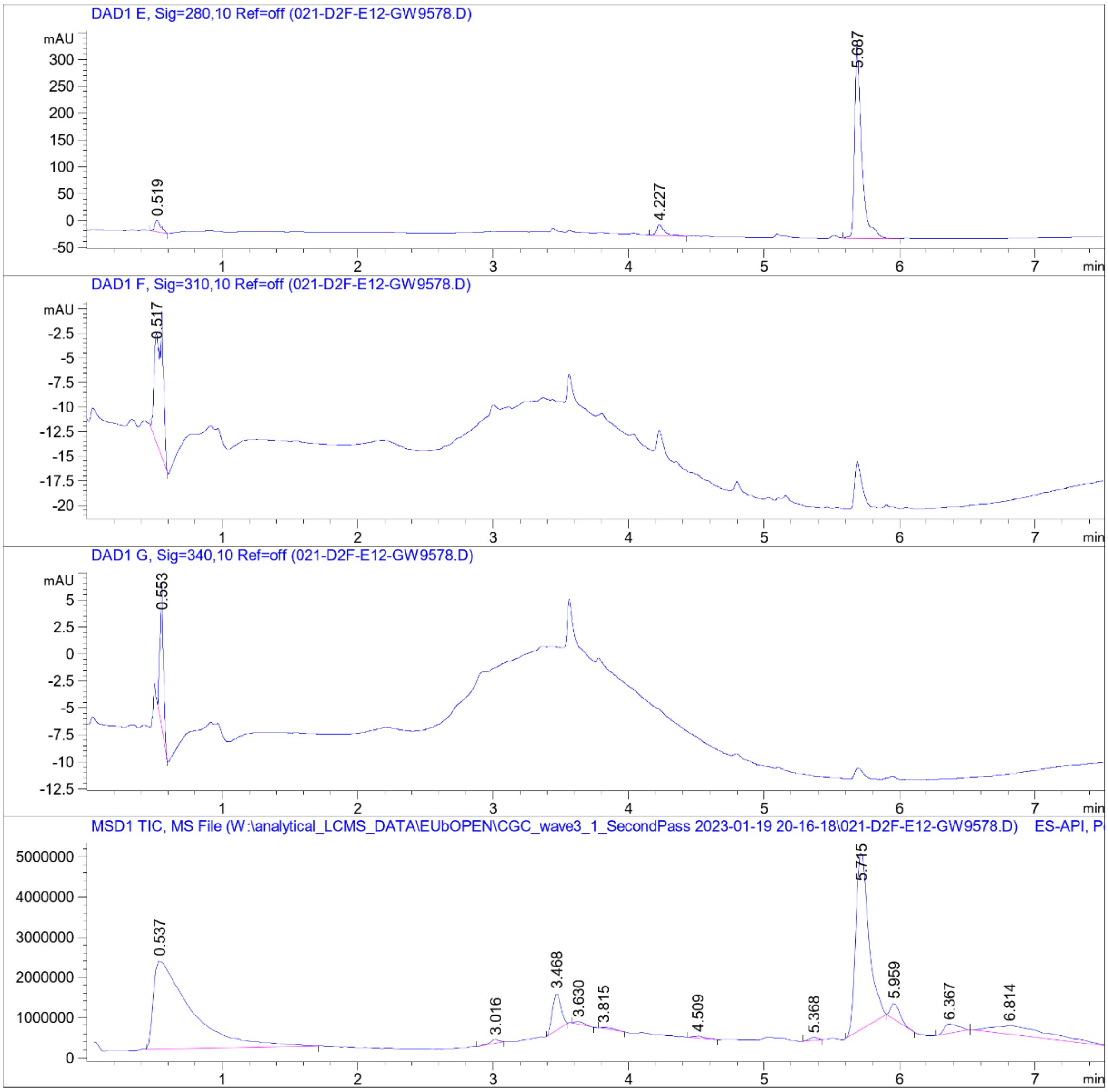

COMPOUND INFORMATION

Data File W:\analyti...PEN\CGC_wave3_1_SecondPass 2023-01-19 20-16-18\021-D2F-E12-GW9578.D
Sample Name: GW9578

MS Signal: MSD1 TIC, MS File, ES-API, Pos, Scan, Frag: 70, "POS Scan"
Spectra from peak tops.
Noise Cutoff: 1000 counts.
Reportable Ion Abundance: > 50%.
LC Signal: DAD1 A, Sig=320,150 Ref=off
Peak matching window: 0.1 min

Retention Time (LC)	LC Area	Retention Time (MS)	MS Area	Mol. Weight or Ion
0.519	80	0.537	42307164	157.00 I
-	-	3.016	375098	239.00 I<br>217.00 I<br>170.80 I
-	-	3.468	3880490	338.20 I
-	-	3.630	335096	170.80 I
-	-	3.815	304643	170.80 I
4.228	45	-	-	
-	-	4.509	313730	510.30 I<br>170.80 I
-	-	5.368	263254	510.30 I<br>348.20 I<br>280.20 I<br>228.20 I<br>137.10 I
5.687	584	5.715	29392894	515.20 I<br>493.20 I
-	-	5.959	1827537	282.20 I
-	-	6.367	1661544	284.20 I<br>282.20 I<br>137.10 I
-	-	6.814	6881839	282.20 I<br>137.10 I

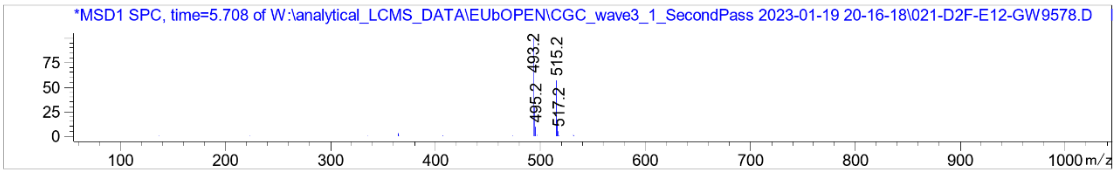

Supplement: Supplementary file 4 — Supplementary Data 1 [file 41467_2024_49493_MOESM4_ESM.zip › GW9578.pdf]
