# Supplementary material for: Chemogenomics for NR1 nuclear hormone receptors
Source: Nat Commun. 2024 Jun 18;15:5201. doi: 10.1038/s41467-024-49493-6 (PMC11189487; doi:10.1038/s41467-024-49493-6)

COMPOUND INFORMATION

KB-130015

**CAS Registry No.:**	147030-48-6
**Formal Name:**	2-[2,6-diiodo-4-[(2-methyl-1-benzofuran-3-yl)methyl]phenoxy]acetic acid
**EUbOPEN ID:**	EUB0002355a
**Molecular Formula:**	$C_{18}H_{14}I_2O_4$
**Molecular Weight:**	548.1 g/mol
**Smiles:**	CC1=C(C2=CC=CC=C2O1)CC3=CC(=C(C(=C3)I)OCC(=O)O)I
**Recommended concentration:**	3 µM

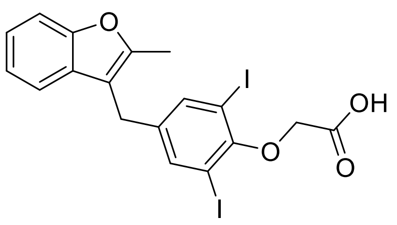

Biological activity

		Type	IC_{50}/EC_{50} [µM]	Reference
Main NR target:	NR1A2 (THRβ)	Antagonist	2.0	inhouse
NR off-target:				

COMPOUND INFORMATION

Identity

^{1}H NMR

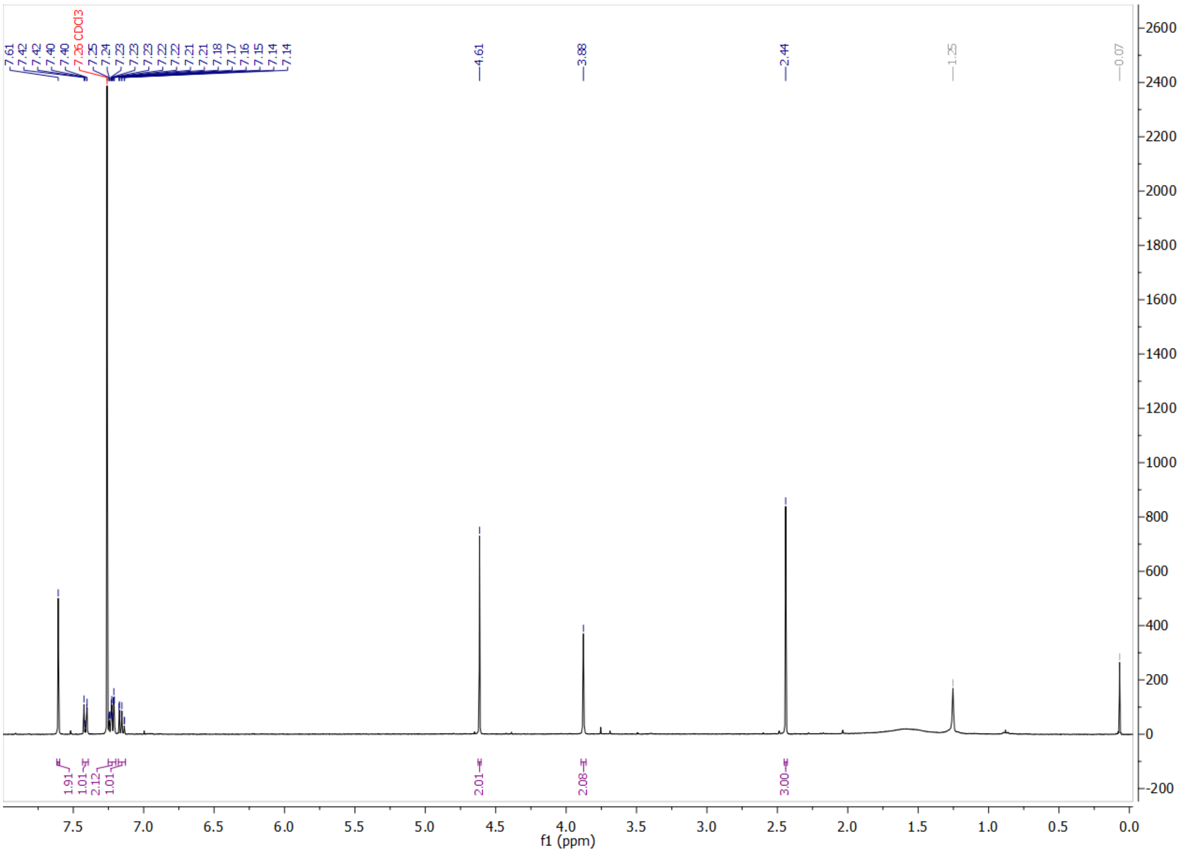

^{13}C NMR

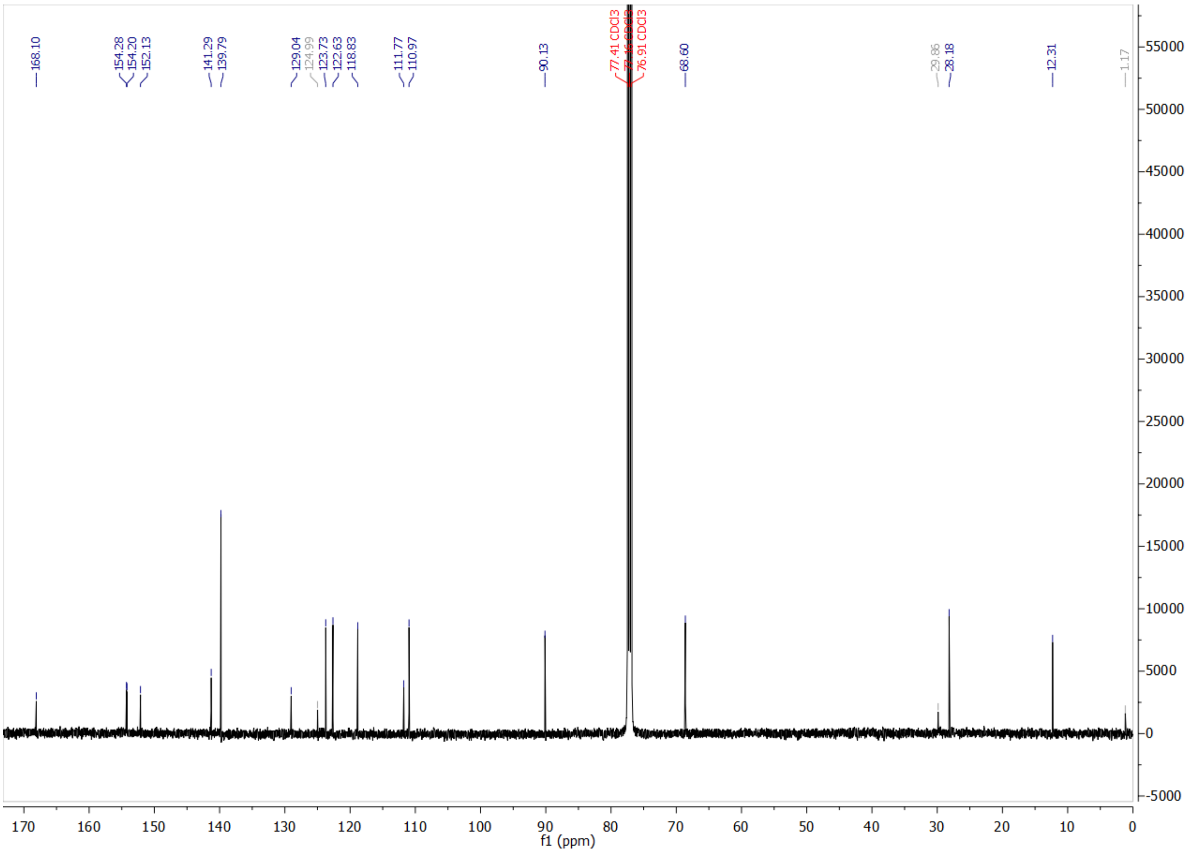

COMPOUND INFORMATION

Purity

LC-MS

M_r 548.11

MS: ESI-negative, *m/z* 547/127 (blue), *m/z* 547/489 (red)

LC: 0.1% HCOOH/ACN (40/60)

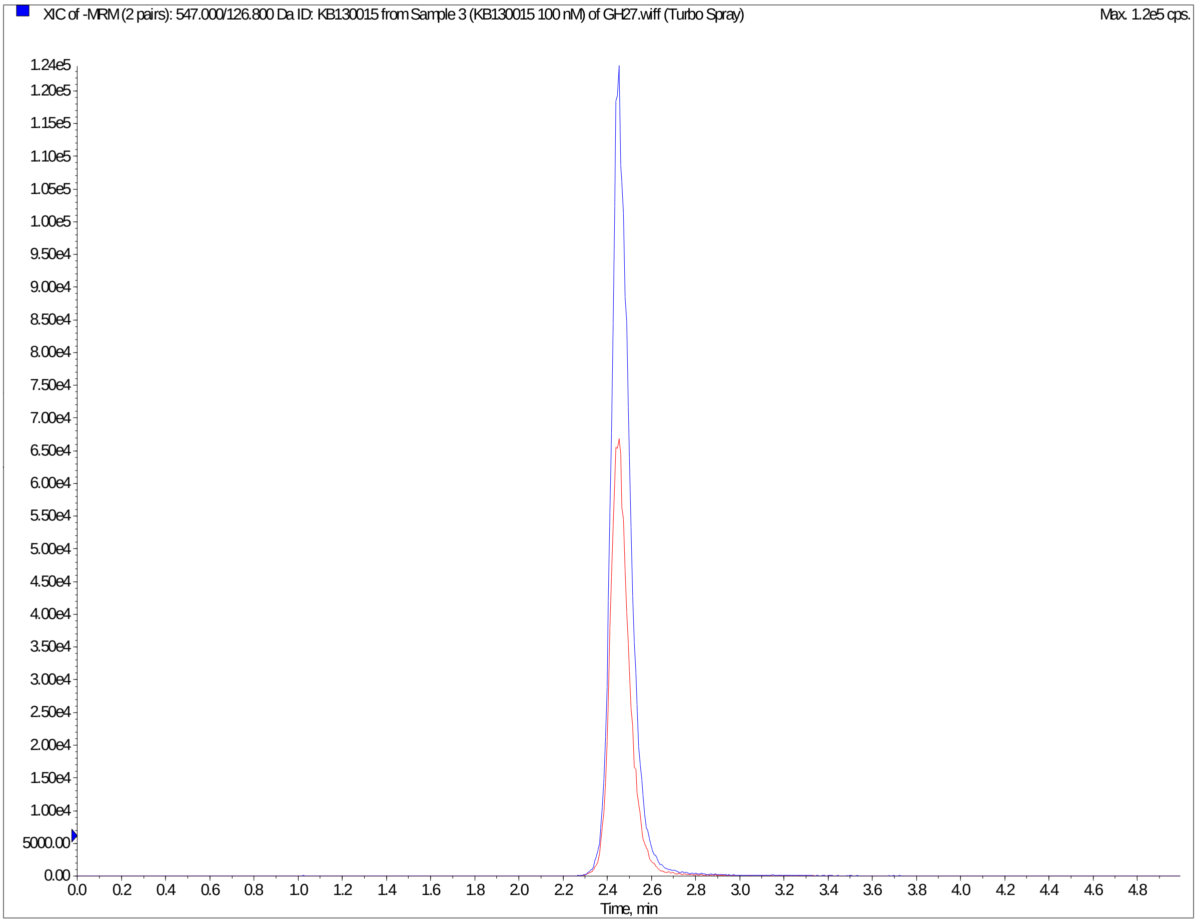

COMPOUND INFORMATION

LC-UV

LC: 0.1% HCOOH/ACN (40/60)

DAD: 215, 230, 240, 254 (XWC), 260 nm

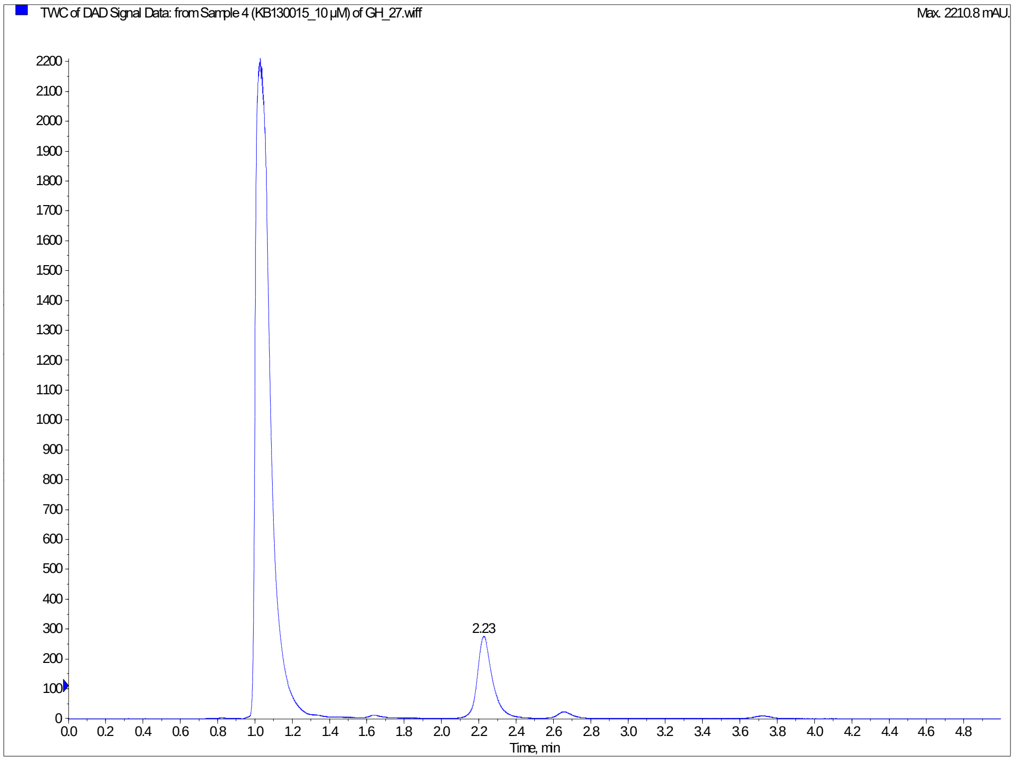

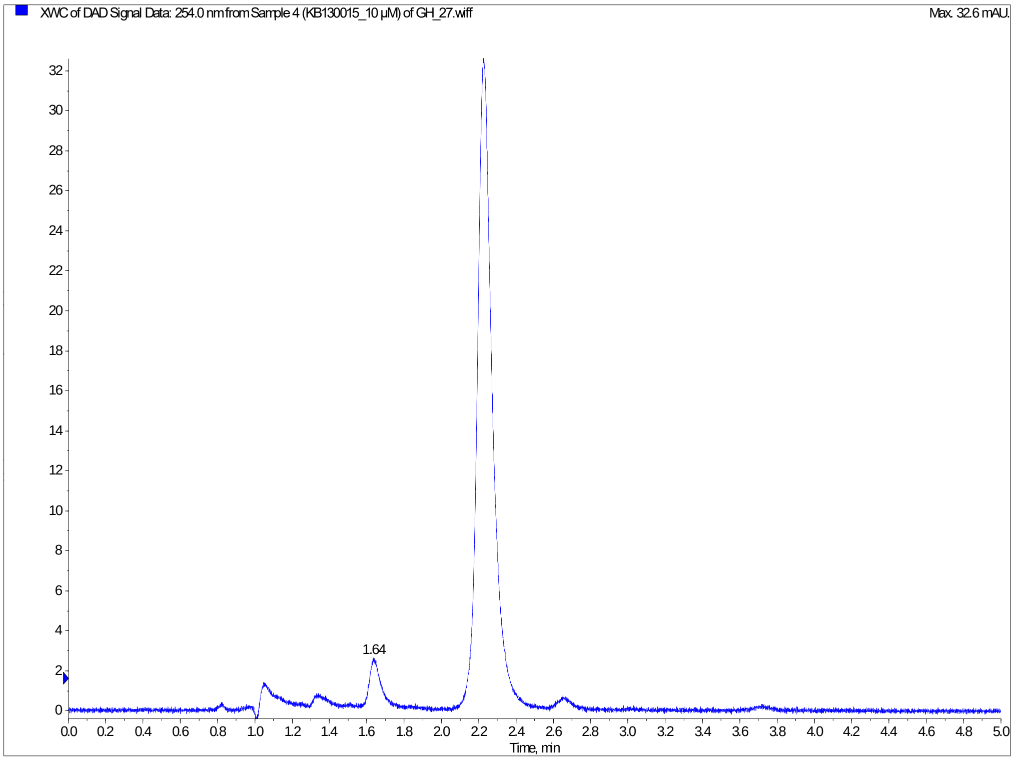

COMPOUND INFORMATION

Biological activity

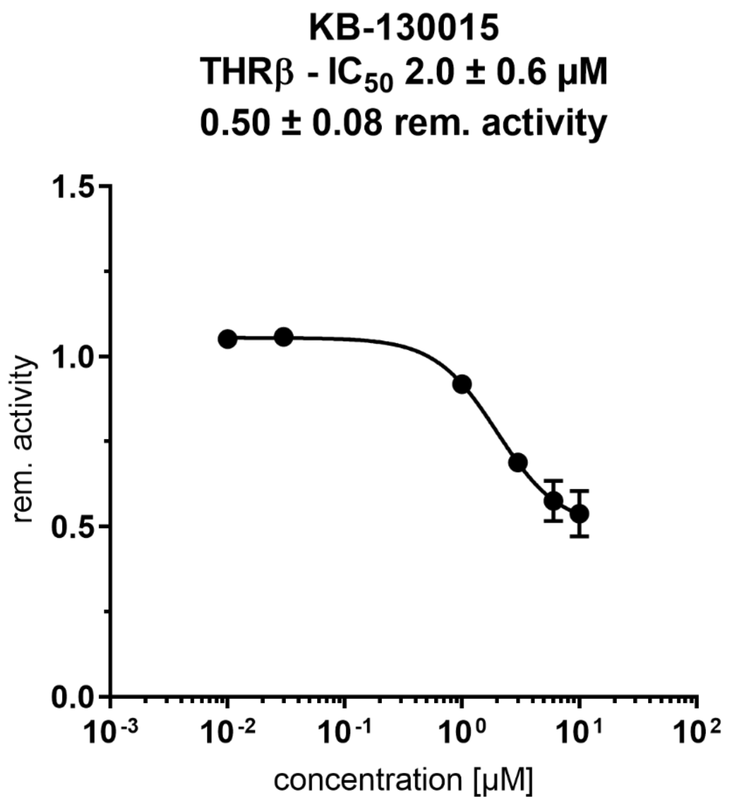

Supplement: Supplementary file 4 — Supplementary Data 1 [file 41467_2024_49493_MOESM4_ESM.zip › KB-130015.pdf]
