# Supplementary material for: Chemogenomics for NR1 nuclear hormone receptors
Source: Nat Commun. 2024 Jun 18;15:5201. doi: 10.1038/s41467-024-49493-6 (PMC11189487; doi:10.1038/s41467-024-49493-6)

COMPOUND INFORMATION

L-165,041

**CAS Registry No.:**	79558-09-1
**Formal Name:**	2-(4-(3-(4-acetyl-3-hydroxy-2-propylphenoxy)propoxy)phenoxy)acetic acid
**EUbOPEN ID:**	EUB0000188b
**Molecular Formula:**	$C_{22}H_{26}O_7$
**Molecular Weight:**	402.44 g/mol
**Smiles:**	CCCC1=C(C=CC(=C1O)C(=O)C)OCCCOC2=CC=C(C=C2)OCC(=O)O
**Recommended concentration:**	1 µM

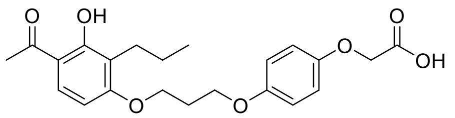

Biological activity

		Type	IC_{50}/EC_{50} [µM]	Reference
Main NR target:	NR1C1 (PPARα)	Agonist	1	https://doi.org/10.1016/j.bmcl.2006.06.028
	NR1C2 (PPARδ)	Agonist	0.015	inhouse
	NR1C3 (PPARγ)	Agonist	1.0	inhouse
NR off-target:				

COMPOUND INFORMATION

Identity

^{1}H NMR

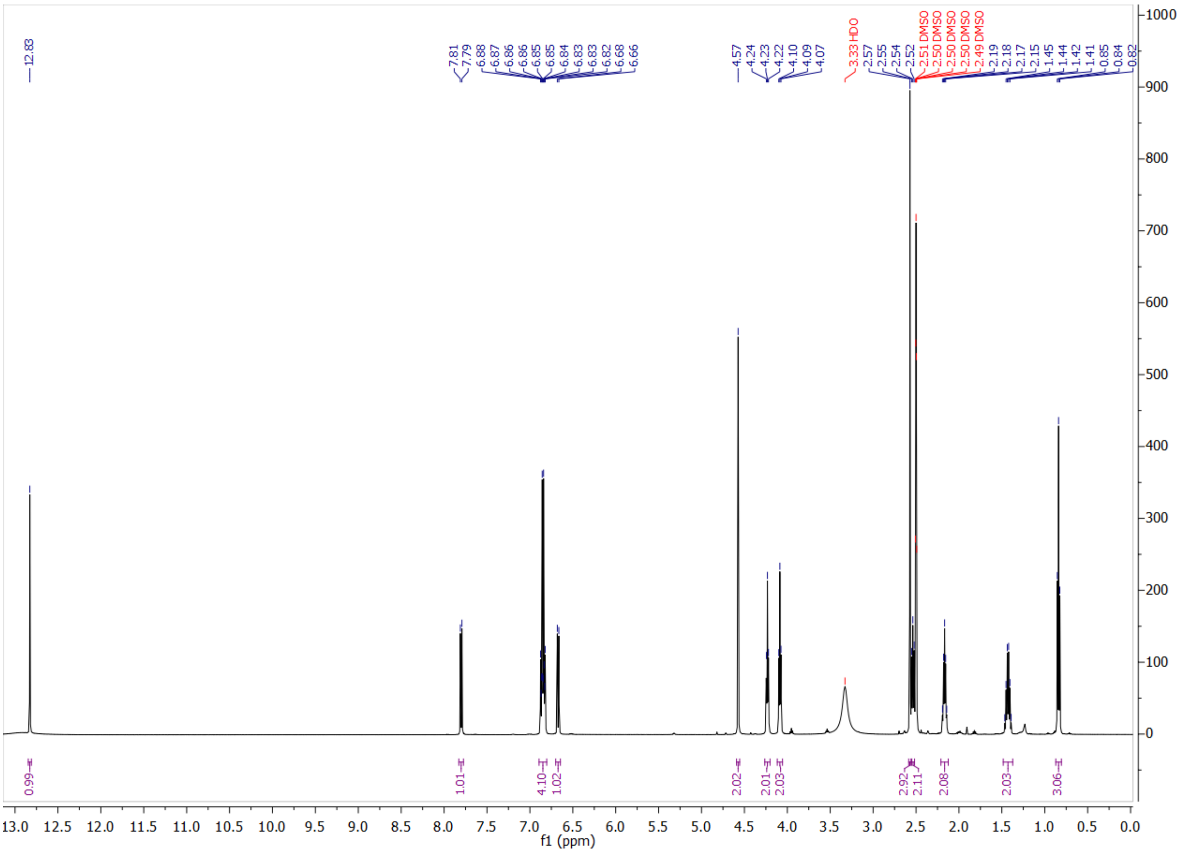

^{13}C NMR

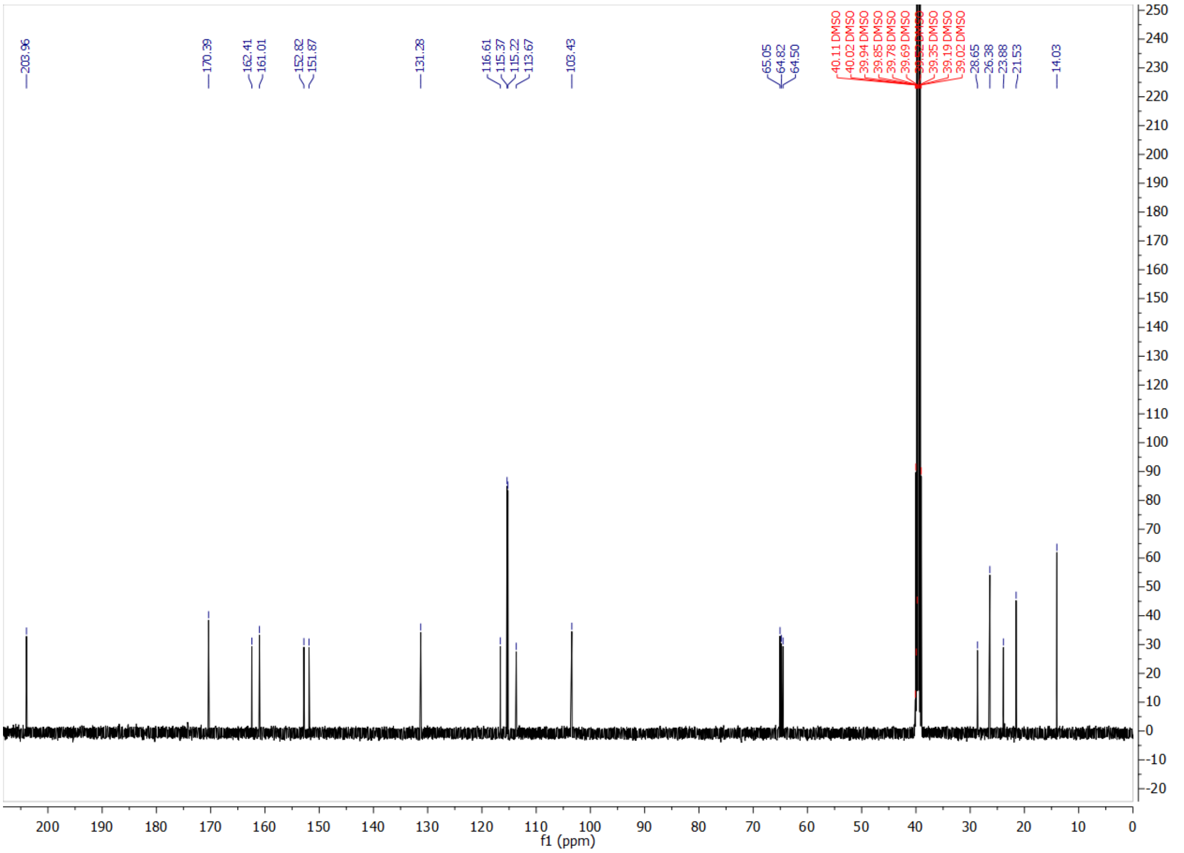

COMPOUND INFORMATION

Purity

```
Data File W:\analyti...OPEN\CGC_ECHO1-3_FirstPass 2021-03-20 13-21-54\055-D2B-E4-L165401.D
Sample Name: L165401

     =====================================================================
     Acq. Operator   : SYSTEM                          Seq. Line :  55
     Sample Operator : SYSTEM
     Acq. Instrument : LCMS test                        Location :  D2B-E4
     Injection Date  : 3/20/2021 11:24:03 PM                 Inj :  1
                                                      Inj Volume : Inj prog
     Sequence File   : W:\analytical_LCMS_DATA\EUbOPEN\CGC_ECHO1-3_FirstPass 2021-03-20 13-21-54
                        \CGC_ECHO1-3_FirstPass.S
     Method          : W:\analytical_LCMS_DATA\EUbOPEN\CGC_ECHO1-3_FirstPass 2021-03-20 13-21-54
                        \CGL_FIRSTPASS_GENERALMETHOD_VIAL3+4_20210319.M (Sequence Method)
     Last changed    : 3/19/2021 5:35:24 PM by SYSTEM
     Method Info     : CGL wellplate, 0.5 uL of 10 mM DMSO, general method
```

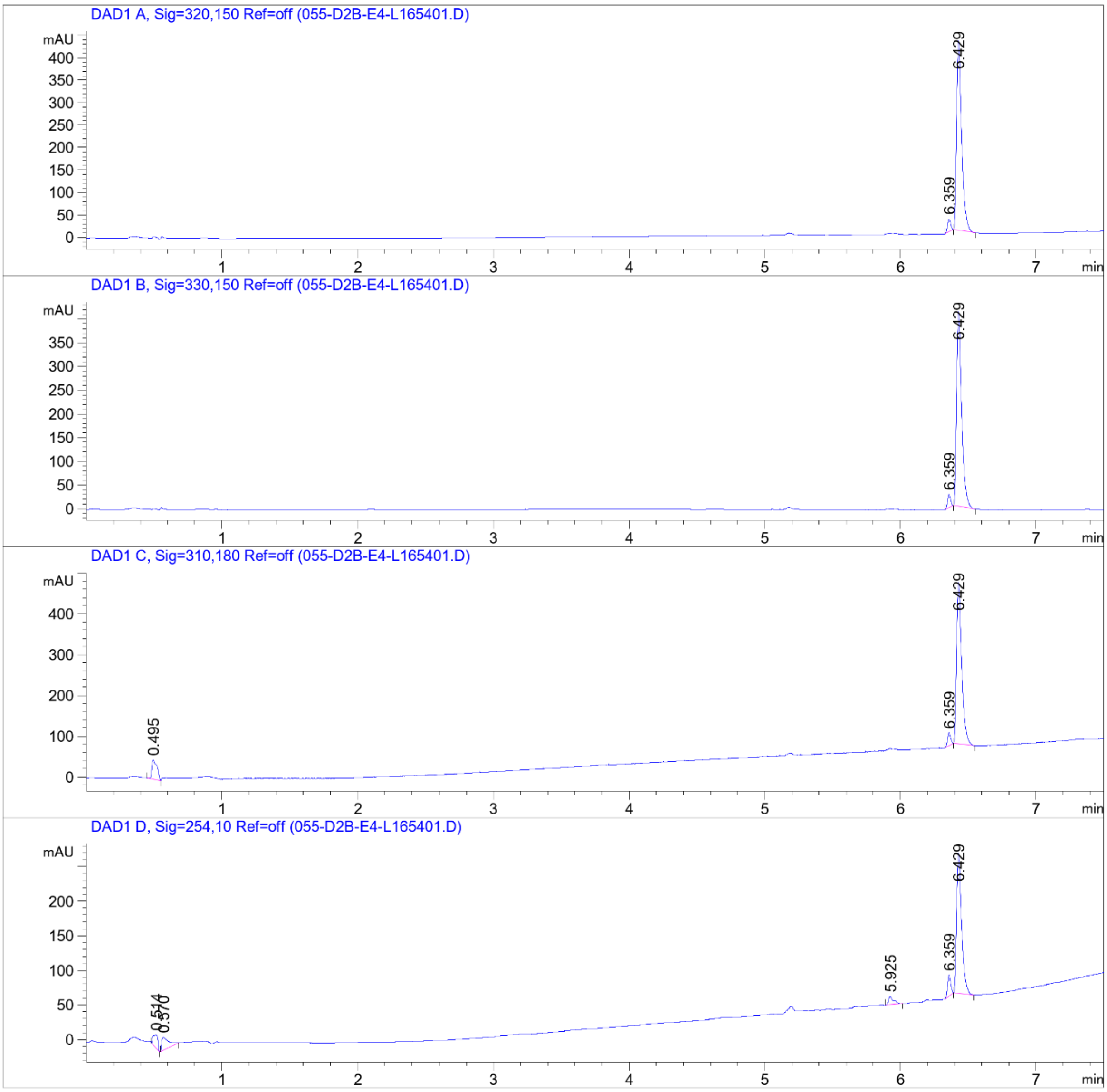

COMPOUND INFORMATION

Data File W:\analyti...OPEN\CGC_ECHO1-3_FirstPass 2021-03-20 13-21-54\055-D2B-E4-L165401.D
Sample Name: L165401

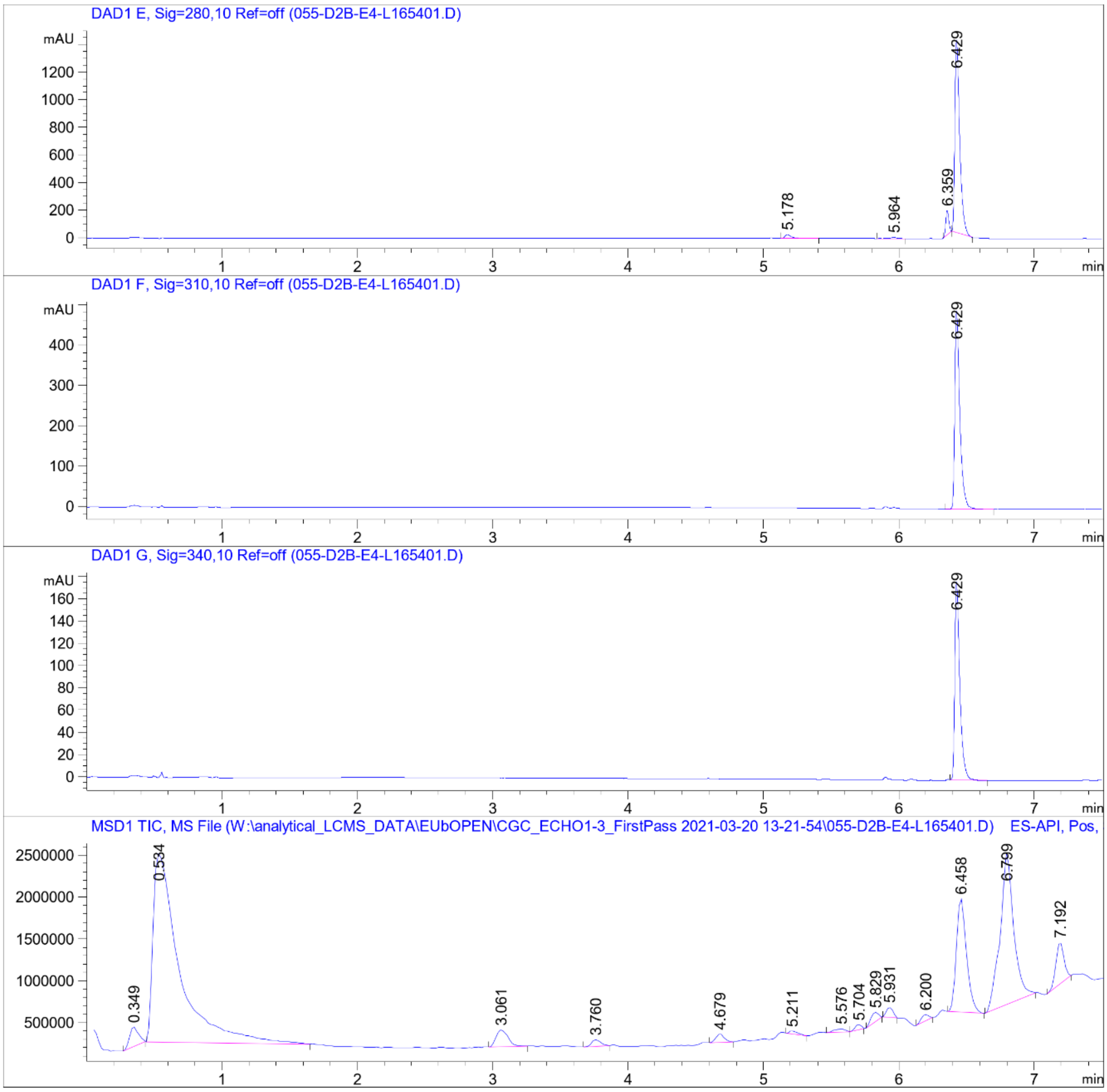

COMPOUND INFORMATION

Data File W:\analyti...OPEN\CGC_ECHO1-3_FirstPass 2021-03-20 13-21-54\055-D2B-E4-L165401.D
Sample Name: L165401

MS Signal: MSD1 TIC, MS File, ES-API, Pos, Scan, Frag: 70, "POS Scan"
Spectra from peak tops.
Noise Cutoff: 1000 counts.
Reportable Ion Abundance: > 50%.
LC Signal: DAD1 A, Sig=320,150 Ref=off
Peak matching window: 0.1 min

Retention Time (LC)	LC Area	Retention Time (MS)	MS Area	Mol. Weight or Ion
-	-	0.349	1043208	183.00 I
				158.00 I
-	-	0.534	30499918	157.00 I
-	-	3.061	1234436	217.10 I
-	-	3.760	359144	274.30 I
-	-	4.679	421777	326.40 I
-	-	5.211	146733	316.20 I
				298.20 I
				296.20 I
				282.20 I
				111.10 I
				102.10 I
-	-	5.576	223078	280.20 I
-	-	5.704	190759	280.20 I
-	-	5.829	409321	296.20 I
-	-	5.931	410022	296.20 I
				294.20 I
				280.30 I
-	-	6.200	296762	280.20 I
				228.20 I
6.359	45	-	-	
6.429	1071	6.458	7134623	403.10 I
-	-	6.799	12539839	282.20 I
-	-	7.192	2091914	284.30 I
				282.20 I

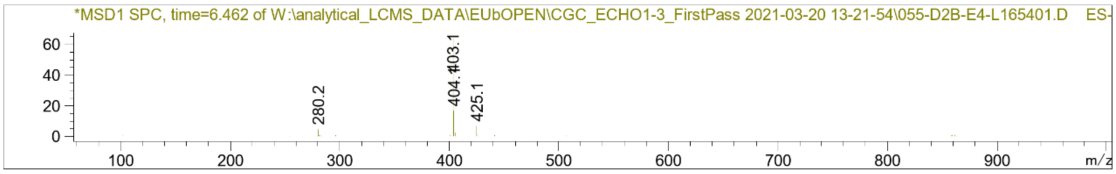

Biological activity

L-165,041
PPARδ - EC_{50} 0.015 ± 0.006 µM
52 ± 4 fold activation

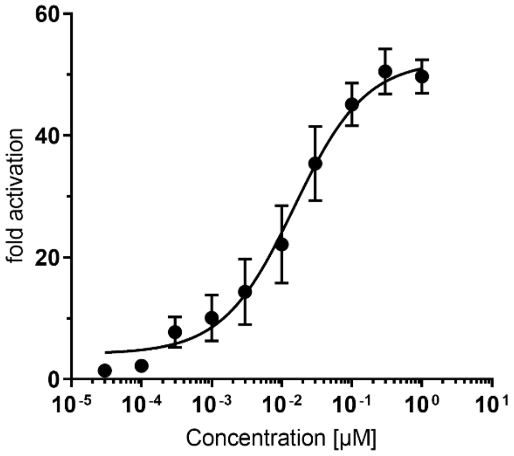

L-165,041
PPARγ - EC_{50} 1.0 ± 0.1 µM
63 ± 4 fold activation

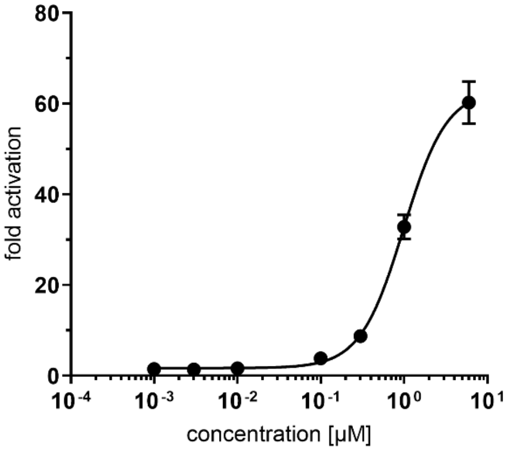

Supplement: Supplementary file 4 — Supplementary Data 1 [file 41467_2024_49493_MOESM4_ESM.zip › L-165,041.pdf]
