# Supplementary material for: Chemogenomics for NR1 nuclear hormone receptors
Source: Nat Commun. 2024 Jun 18;15:5201. doi: 10.1038/s41467-024-49493-6 (PMC11189487; doi:10.1038/s41467-024-49493-6)

COMPOUND INFORMATION

Lanifibranor

**CAS Registry No.:**	927961-18-0
**Formal Name:**	4-(1-(benzo[d]thiazol-6-ylsulfonyl)-5-chloro-1H-indol-2-yl)butanoic acid
**EUbOPEN ID:**	EUB0001149a
**Molecular Formula:**	$C_{19}H_{15}ClN_2O_4S_2$
**Molecular Weight:**	434.91 g/mol
**Smiles:**	C1=CC2=C(C=C1S(=O)(=O)N3C4=C(C=C(C=C4)Cl)C=C3CCCC(=O)O)SC=N2
**Recommended concentration:**	1 µM

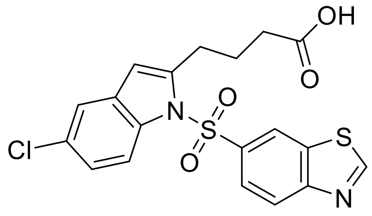

Biological activity

		Type	IC50/EC50 [µM]	Reference
Main NR target:	NR1C2 (PPARδ)	Agonist	0.9	https://doi.org/10.1021/acs.jmedchem.7b01285
	NR1C3 (PPARγ)	Agonist	0.21	
NR off-target:	NR1C1 (PPARα)	Agonist	1.5.	https://doi.org/10.1021/acs.jmedchem.7b01285

COMPOUND INFORMATION

Identity

^{1}H NMR

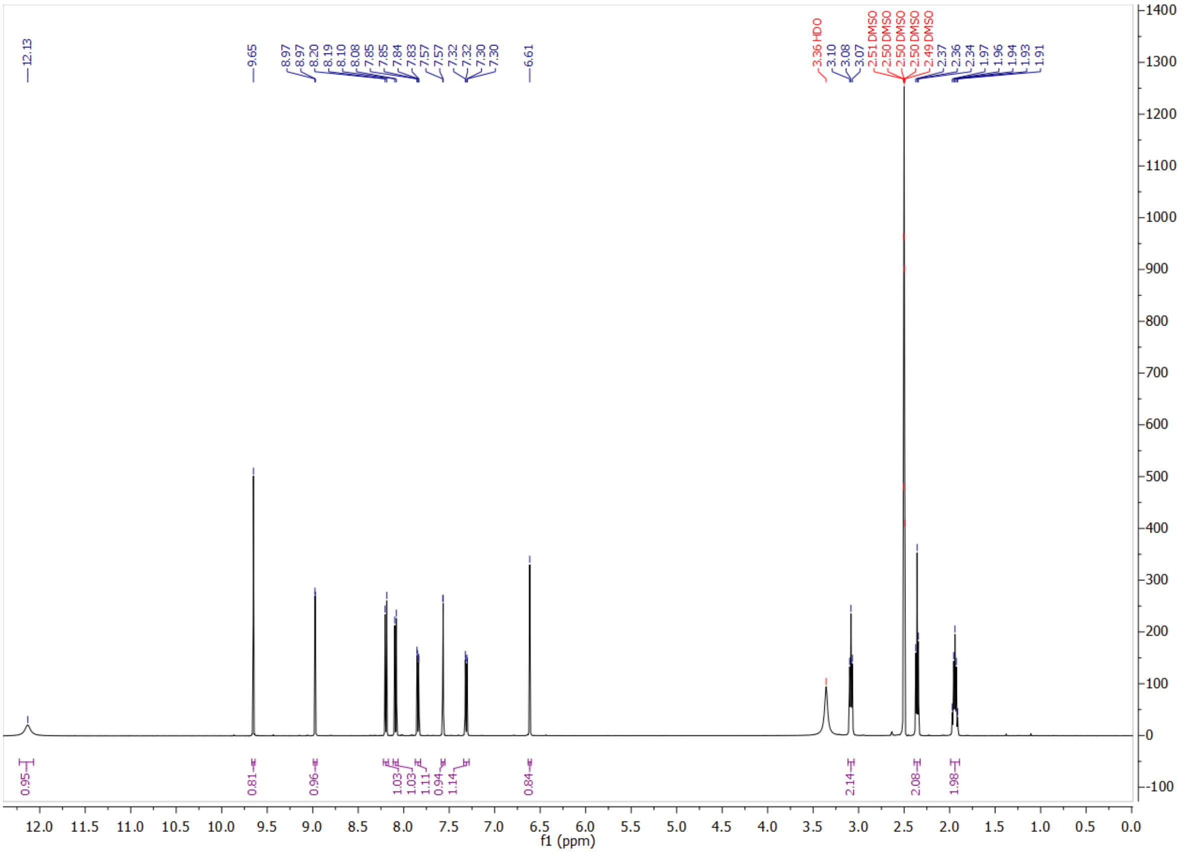

^{13}C NMR

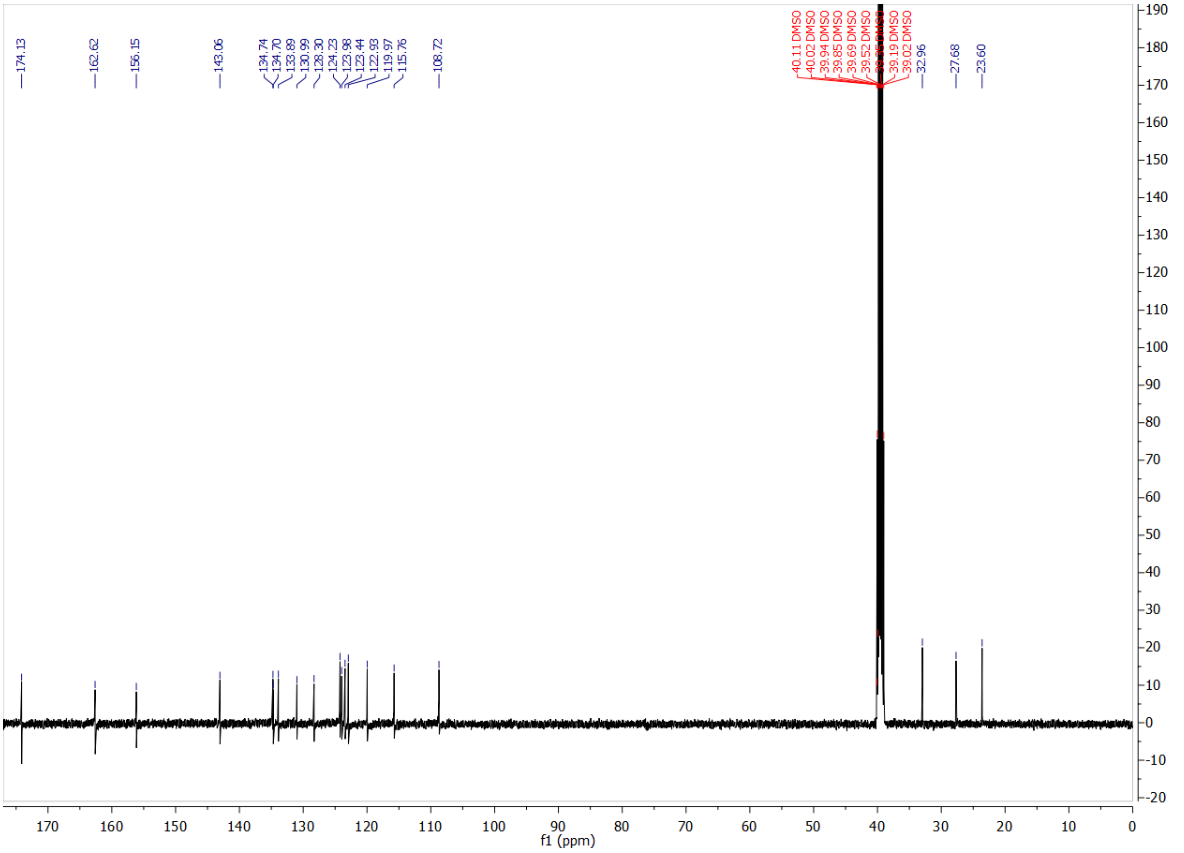

COMPOUND INFORMATION

Purity

Data File W:\analyti...GC_wave3_1_FirstPassB 2023-01-04 18-28-02\070-D2F-F7-Lanifibranor.D
Sample Name: Lanifibranor

```
=====================================================================
Acq. Operator   : SYSTEM                        Seq. Line :  70
Sample Operator : SYSTEM
Acq. Instrument : LCMS test                      Location :   D2F-F7
Injection Date  : 1/5/2023 7:13:50 AM                 Inj :   1
                                                Inj Volume : Inj prog
Sequence File   : W:\analytical_LCMS_DATA\EUbOPEN\CGC_wave3_1_FirstPassB 2023-01-04 18-28-02
                  \CGC_wave3_1_FirstPassB.S
Method          : W:\analytical_LCMS_DATA\EUbOPEN\CGC_wave3_1_FirstPassB 2023-01-04 18-28-02
                  \CGL_FIRSTPASS_GENERALMETHOD_VIAL1+2_20210319.M (Sequence Method)
Last changed    : 1/25/2022 4:36:18 PM by SYSTEM
Method Info     : CGL wellplate, 0.5 uL of 10 mM DMSO, general method
```

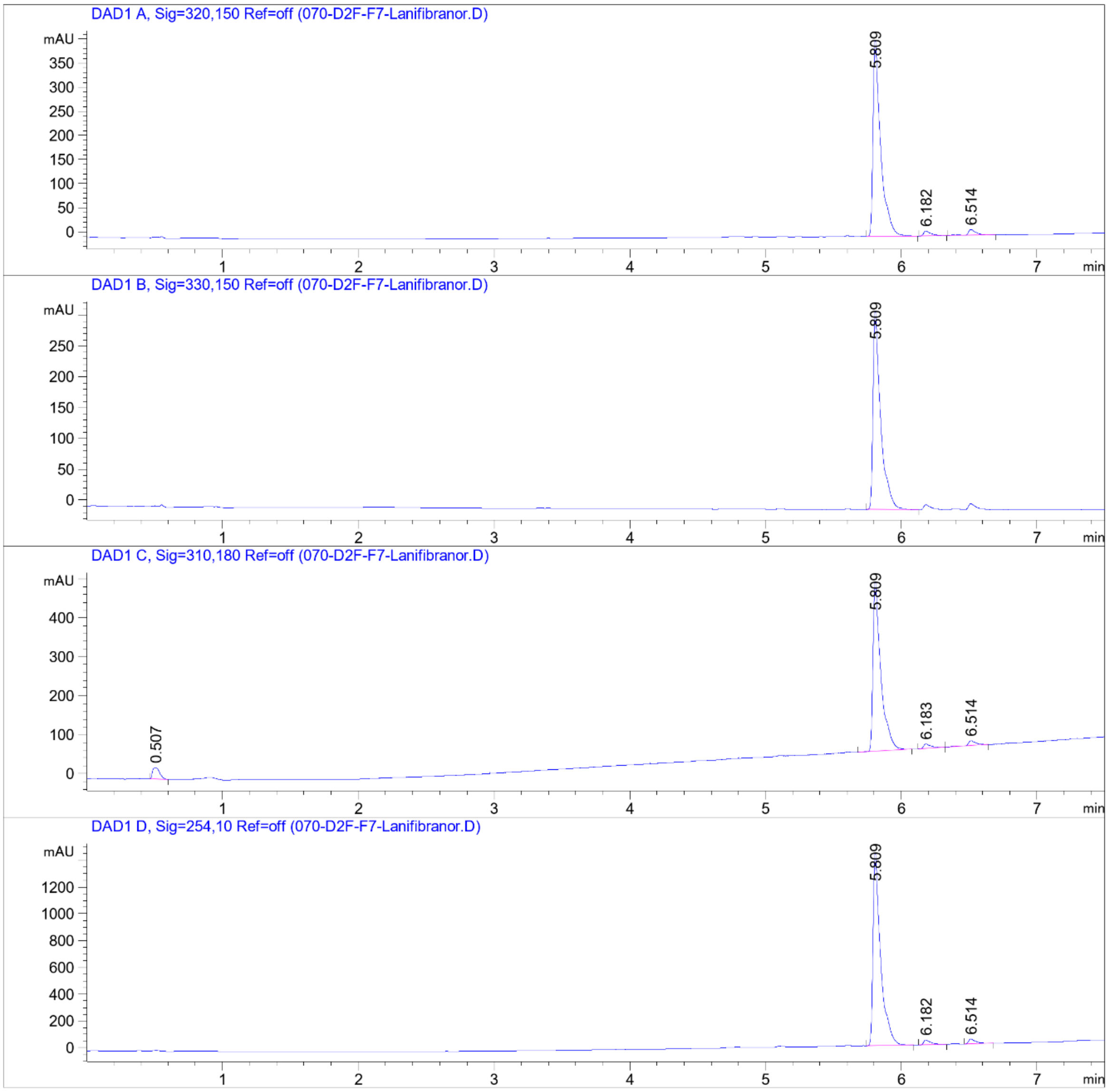

COMPOUND INFORMATION

Data File W:\analyti...GC_wave3_1_FirstPassB 2023-01-04 18-28-02\070-D2F-F7-Lanifibranor.D
Sample Name: Lanifibranor

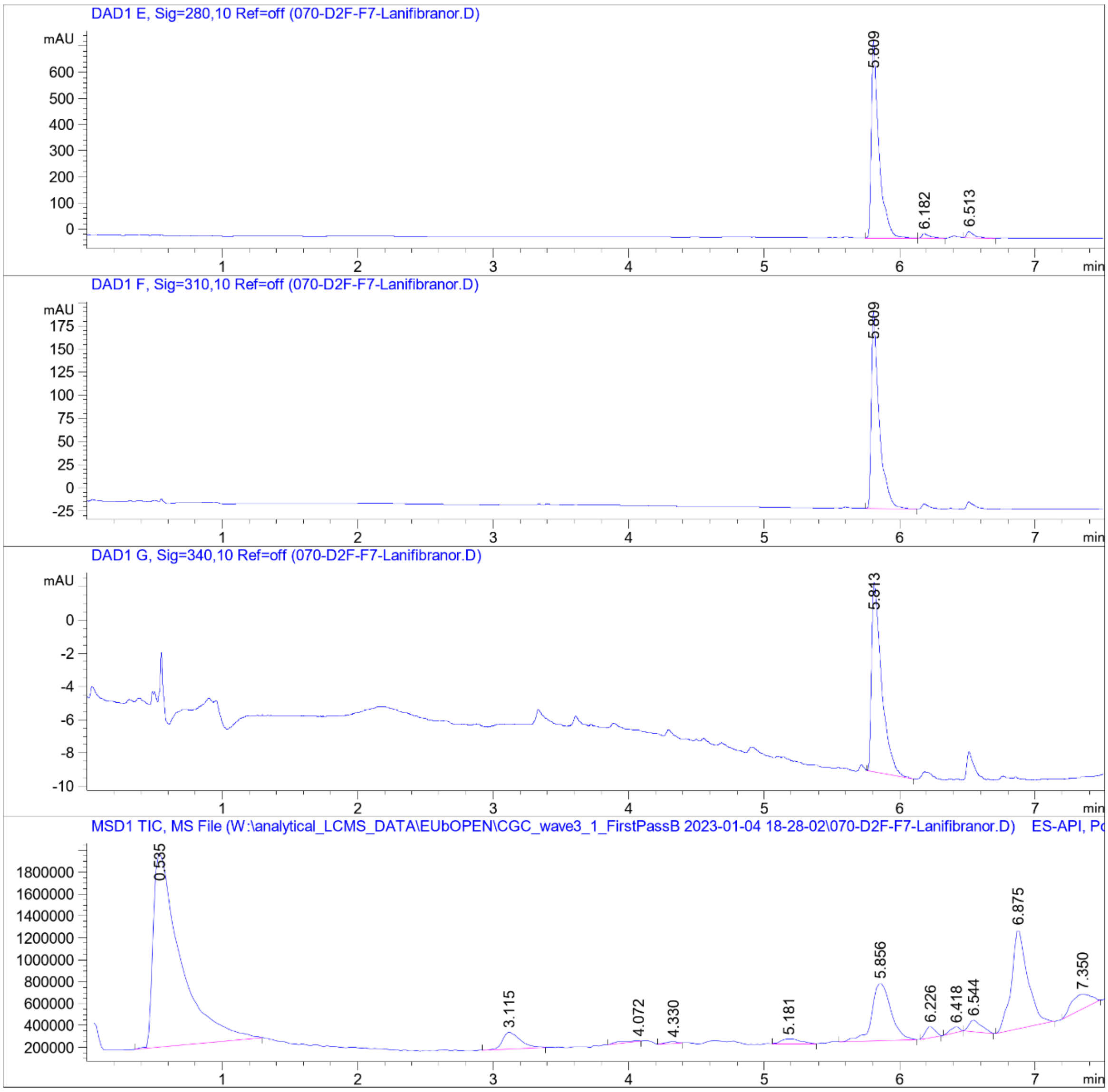

COMPOUND INFORMATION

Data File W:\analyti...GC_wave3_1_FirstPassB 2023-01-04 18-28-02\070-D2F-F7-Lanifibranor.D
Sample Name: Lanifibranor

MS Signal: MSD1 TIC, MS File, ES-API, Pos, Scan, Frag: 70, "POS Scan"
Spectra from peak tops.
Noise Cutoff: 1000 counts.
Reportable Ion Abundance: > 50%.
LC Signal: DAD1 A, Sig=320,150 Ref=off
Peak matching window: 0.1 min

Retention Time (LC)	LC Area	Retention Time (MS)	MS Area	Mol. Weight or Ion
-	-	0.535	24951508	157.00 I
-	-	3.115	1434967	239.00 I
				217.00 I
-	-	4.072	151780	170.90 I
-	-	4.330	99141	510.40 I
				170.80 I
-	-	5.181	436414	510.40 I
				170.90 I
				137.10 I
5.809	1583	5.856	5196727	435.00 I
6.182	40	6.226	496043	449.00 I
				228.20 I
				137.10 I
-	-	6.418	222562	282.20 I
				254.20 I
6.514	58	6.544	668935	507.20 I
				280.20 I
-	-	6.875	7607204	282.30 I
-	-	7.350	1427781	400.30 I
				282.20 I

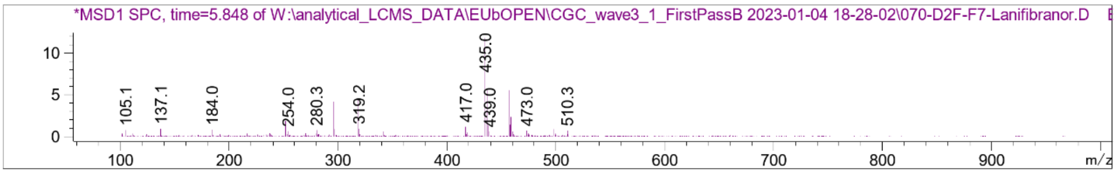

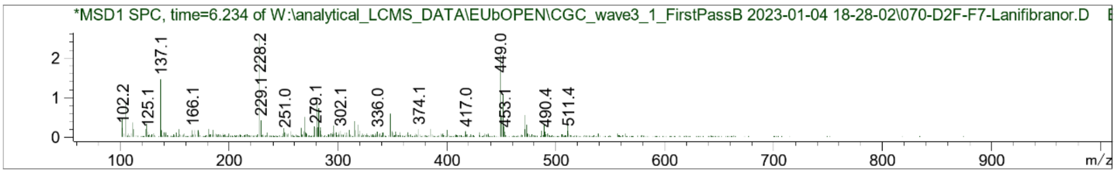

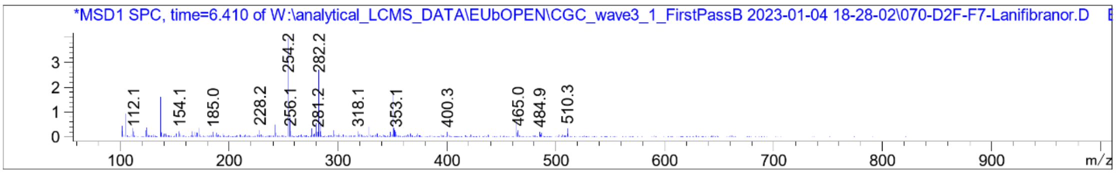

Supplement: Supplementary file 4 — Supplementary Data 1 [file 41467_2024_49493_MOESM4_ESM.zip › Lanifibranor.pdf]
