# Supplementary material for: Chemogenomics for NR1 nuclear hormone receptors
Source: Nat Commun. 2024 Jun 18;15:5201. doi: 10.1038/s41467-024-49493-6 (PMC11189487; doi:10.1038/s41467-024-49493-6)

COMPOUND INFORMATION

LXR-623

**CAS Registry No.:**	875787-07-8
**Formal Name:**	2-(2-chloro-4-fluorobenzyl)-3-(4-fluorophenyl)-7-(trifluoromethyl)-2H-indazole
**EUBOPEN ID:**	EUB0000082b
**Molecular Formula:**	$C_{21}H_{12}ClF_5N_2$
**Molecular Weight:**	422.78 g/mol
**Smiles:**	FC1=CC=C(C2=C3C(C(C(F)(F)F)=CC=C3)=NN2CC4=CC=C(C=C4Cl)F)C=C1
**Recommended concentration:**	3 µM

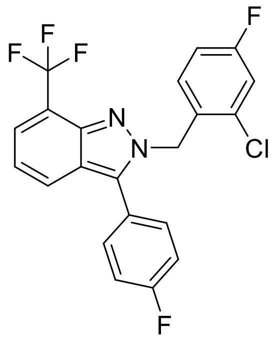

Biological activity

		Type	IC_{50}/EC_{50} [µM]	Reference
Main NR target:	NR1H3 (LXRα)	Agonist	7	https://doi.org/10.1021/jm800799q
	NR1H2 (LXRβ)	Agonist	4	
NR off-target:	NR1I2 (PXR)	Agonist	2	https://doi.org/10.1021/jm800799q

COMPOUND INFORMATION

Identity

^{1}H NMR

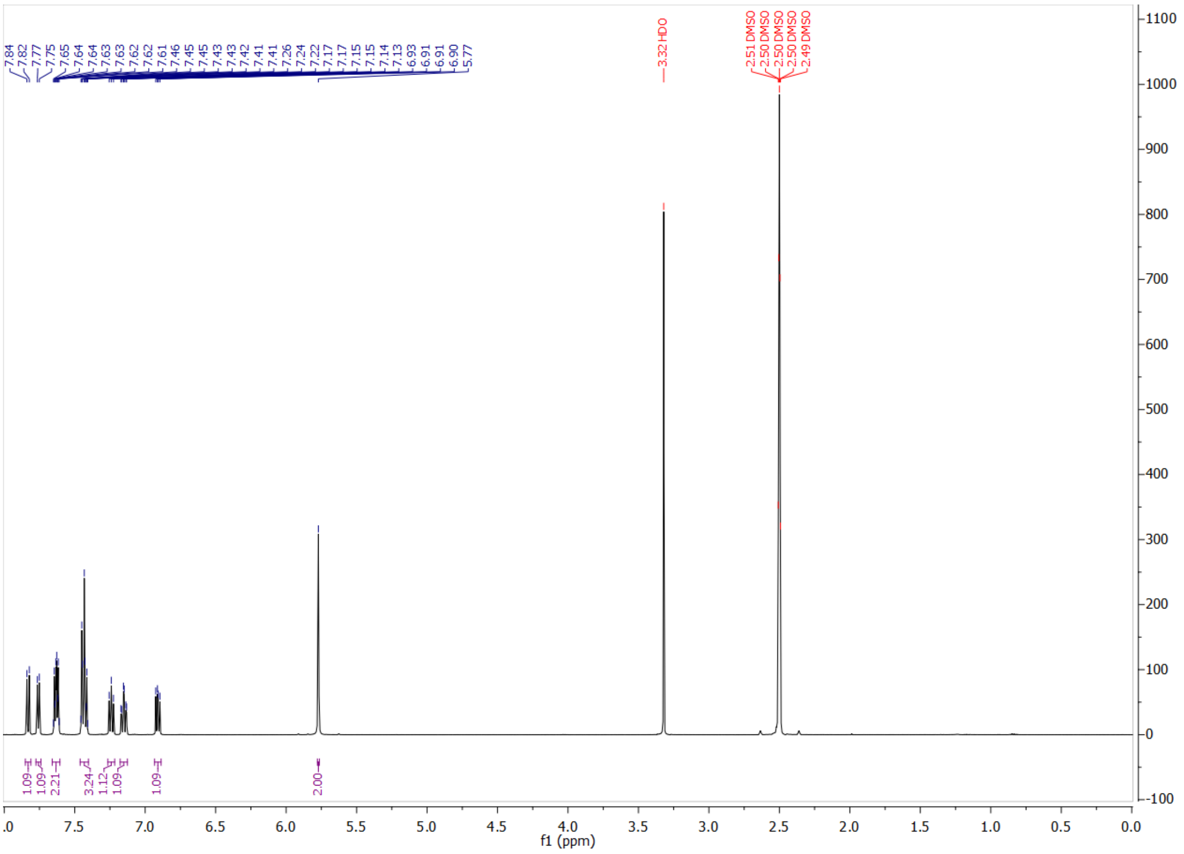

^{13}C NMR

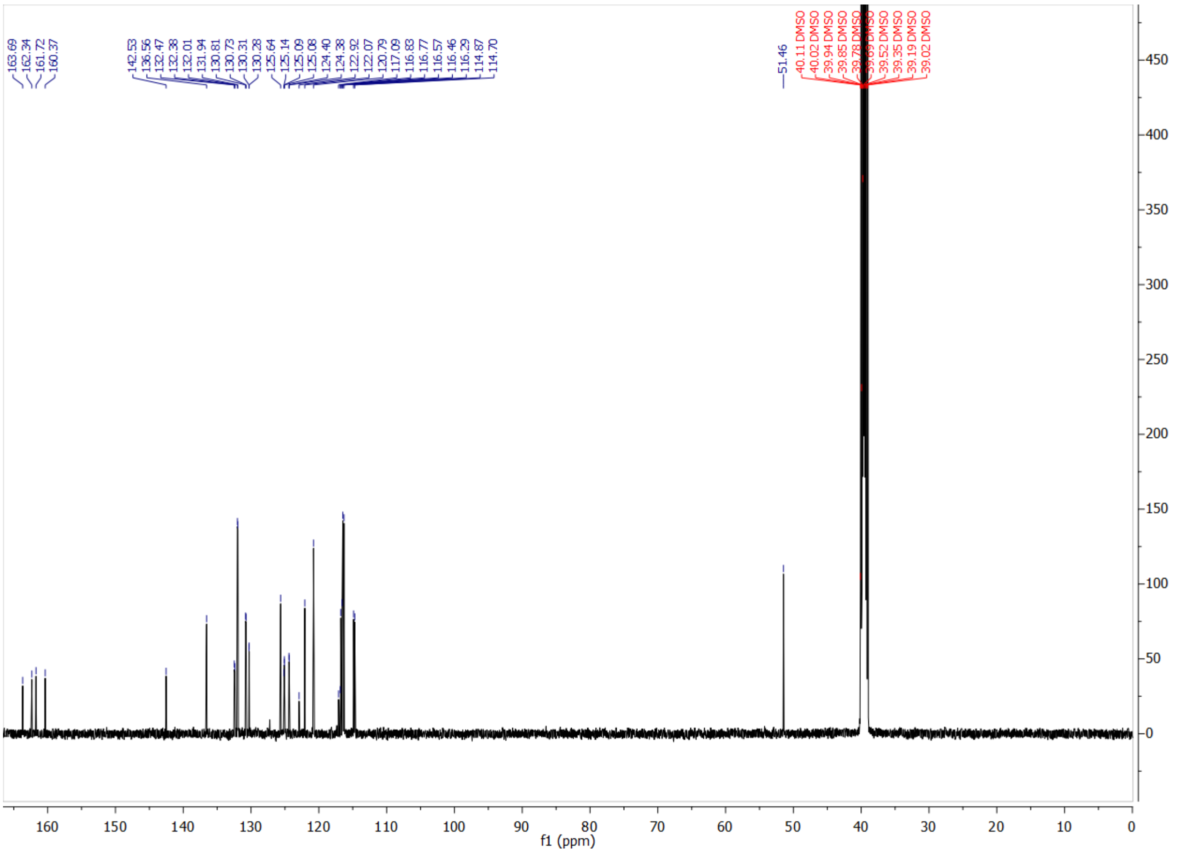

COMPOUND INFORMATION

Purity

Data File W:\analyti...EN\CGC_wave3_1_FirstPassB 2023-01-04 18-28-02\075-D2F-G11-LXR-623.D
Sample Name: LXR-623

```
=====================================================================
Acq. Operator   : SYSTEM                          Seq. Line :  75
Sample Operator : SYSTEM
Acq. Instrument : LCMS test                        Location :   D2F-G11
Injection Date  : 1/5/2023 8:09:07 AM                   Inj :   1
                                                 Inj Volume : Inj prog
Sequence File   : W:\analytical_LCMS_DATA\EUbOPEN\CGC_wave3_1_FirstPassB 2023-01-04 18-28-02
                  \CGC_wave3_1_FirstPassB.S
Method          : W:\analytical_LCMS_DATA\EUbOPEN\CGC_wave3_1_FirstPassB 2023-01-04 18-28-02
                  \CGL_FIRSTPASS_GENERALMETHOD_VIAL1+2_20210319.M (Sequence Method)
Last changed    : 1/25/2022 4:36:18 PM by SYSTEM
Method Info     : CGL wellplate, 0.5 uL of 10 mM DMSO, general method
```

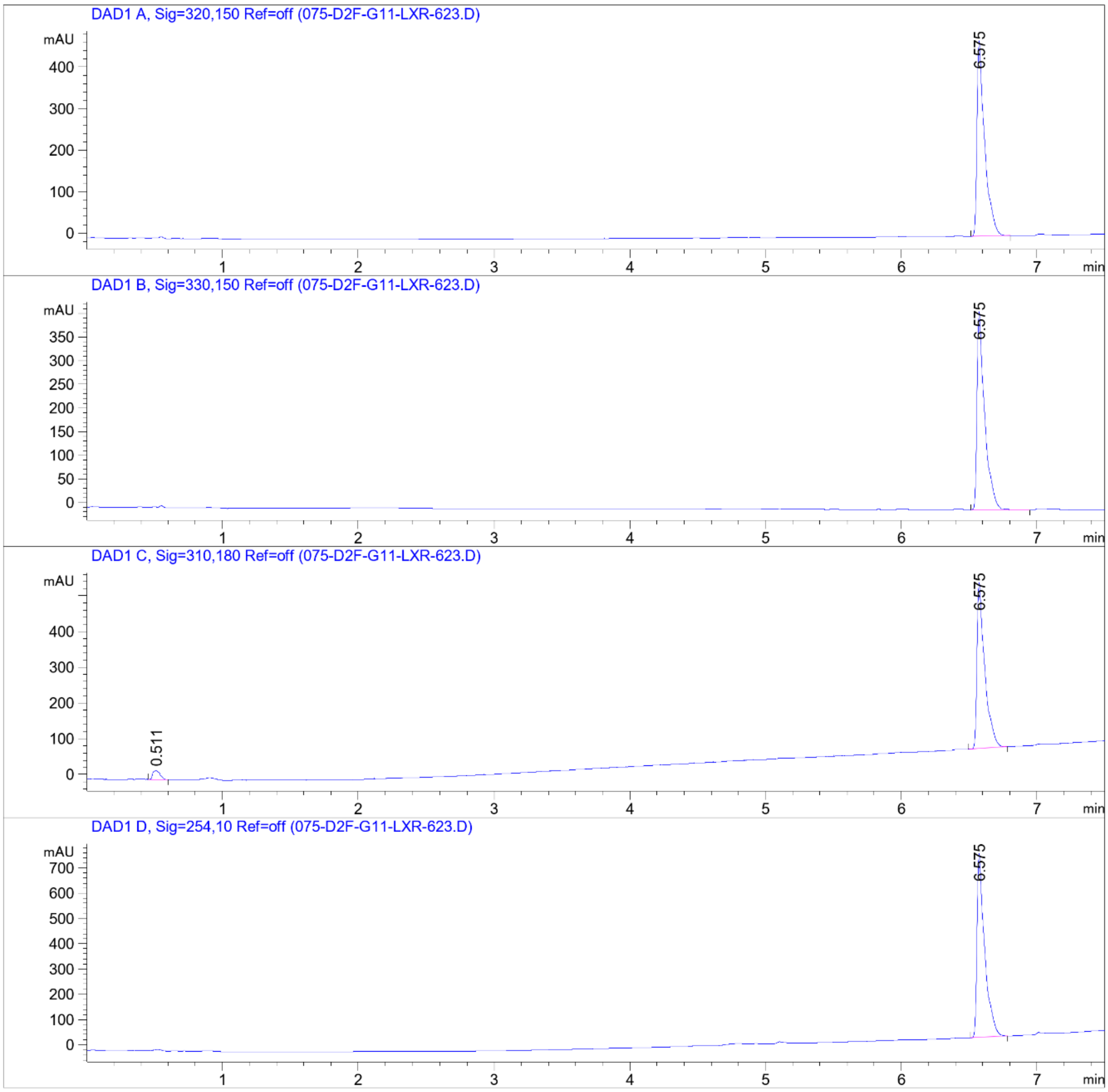

COMPOUND INFORMATION

Data File W:\analyti...EN\CGC_wave3_1_FirstPassB 2023-01-04 18-28-02\075-D2F-G11-LXR-623.D
Sample Name: LXR-623

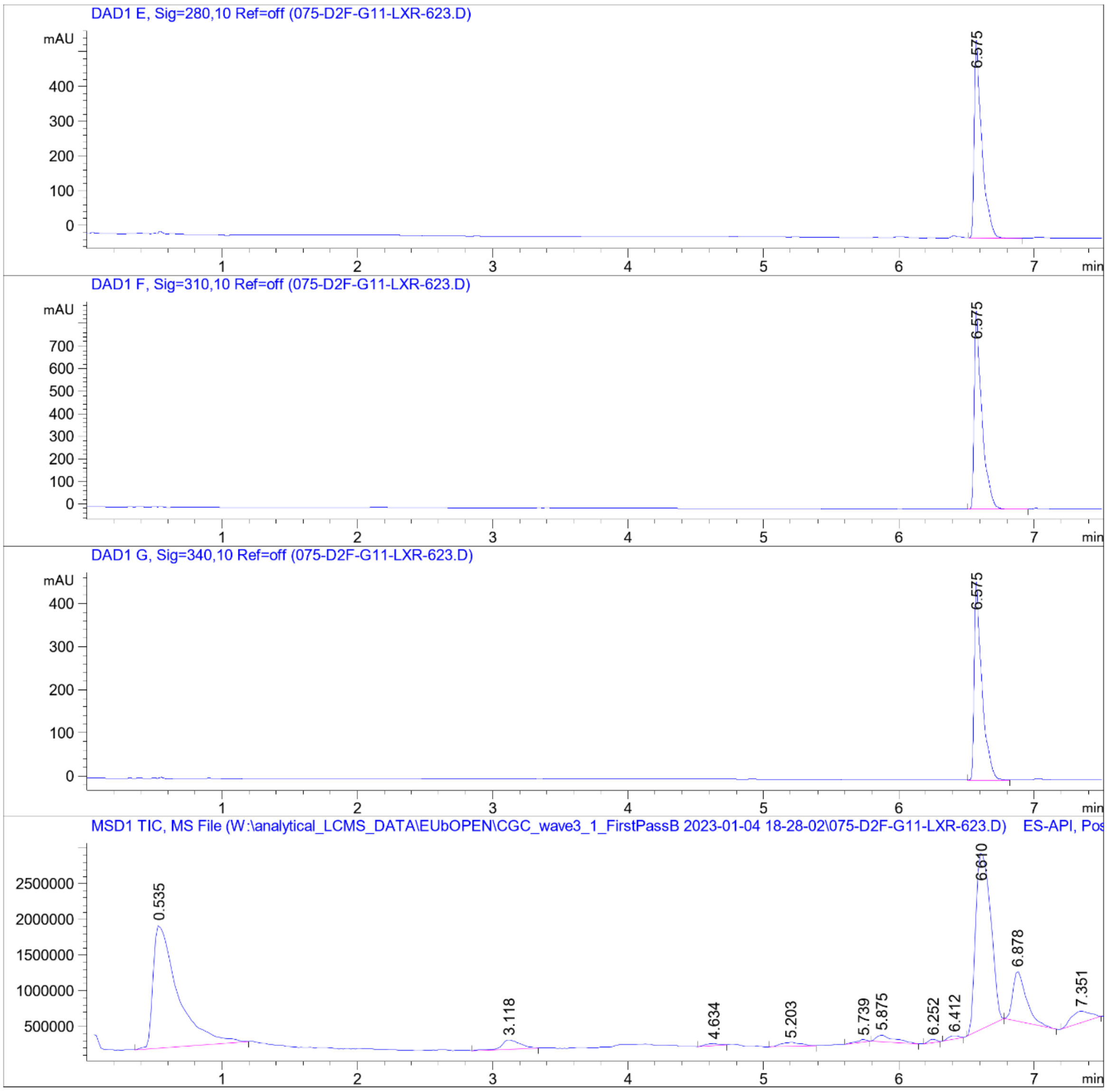

COMPOUND INFORMATION

Data File W:\analyti...EN\CGC_wave3_1_FirstPassB 2023-01-04 18-28-02\075-D2F-G11-LXR-623.D
Sample Name: LXR-623

MS Signal: MSD1 TIC, MS File, ES-API, Pos, Scan, Frag: 70, "POS Scan"
Spectra from peak tops.
Noise Cutoff: 1000 counts.
Reportable Ion Abundance: > 50%.
LC Signal: DAD1 A, Sig=320,150 Ref=off
Peak matching window: 0.1 min

Retention Time (LC)	LC Area	Retention Time (MS)	MS Area	Mol. Weight or Ion
-	-	0.535	21933610	157.00 I
-	-	3.118	1253992	239.10 I
				217.10 I
-	-	4.634	179693	510.40 I
				170.90 I
				158.10 I
-	-	5.203	516207	510.30 I
				170.90 I
				137.10 I
-	-	5.739	188284	280.20 I
-	-	5.875	845959	318.20 I
				296.20 I
-	-	6.252	168962	228.20 I
				137.10 I
-	-	6.412	253319	282.20 I
				254.20 I
6.575	1832	6.610	19212684	423.00 I
-	-	6.878	4852625	282.20 I
-	-	7.351	1644809	400.30 I
				282.20 I

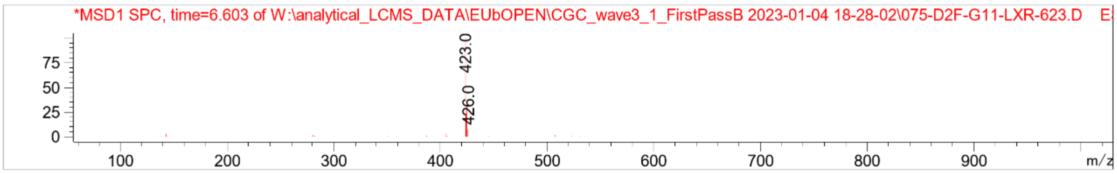

Supplement: Supplementary file 4 — Supplementary Data 1 [file 41467_2024_49493_MOESM4_ESM.zip › LXR-623.pdf]
