# Supplementary material for: Chemogenomics for NR1 nuclear hormone receptors
Source: Nat Commun. 2024 Jun 18;15:5201. doi: 10.1038/s41467-024-49493-6 (PMC11189487; doi:10.1038/s41467-024-49493-6)

COMPOUND INFORMATION

LY2562175

**CAS Registry No.:**	1103500-20-4
**Formal Name:**	6-(4-((5-cyclopropyl-3-(2,6-dichlorophenyl)isoxazol-4-yl)methoxy)piperidin-1-yl)-1-methyl-1H-indole-3-carboxylic acid
**EUBOPEN ID:**	EUB0001173a
**Molecular Formula:**	$C_{28}H_{27}Cl_2N_3O_4$
**Molecular Weight:**	540.44 g/mol
**Smiles:**	CN1C=C(C2=CC=C(C=C21)N3CCC(CC3)OCC4=C(ON=C4C5=C(C=CC=C5Cl)Cl)C6CC6)C(O)=O
**Recommended concentration:**	1 μM

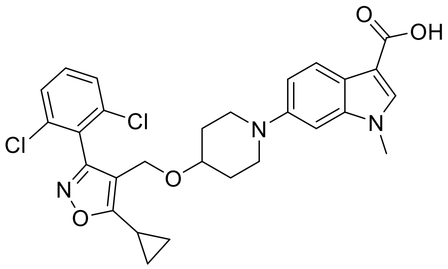

Biological activity

		Type	IC_{50}/EC_{50} [μM]	Reference
Main NR target:	NR1H4 (FXR)	Agonist	0.2	https://doi.org/10.1021/acs.jmedchem.5b01161
NR off-target:				

COMPOUND INFORMATION

Identity

^{1}H NMR

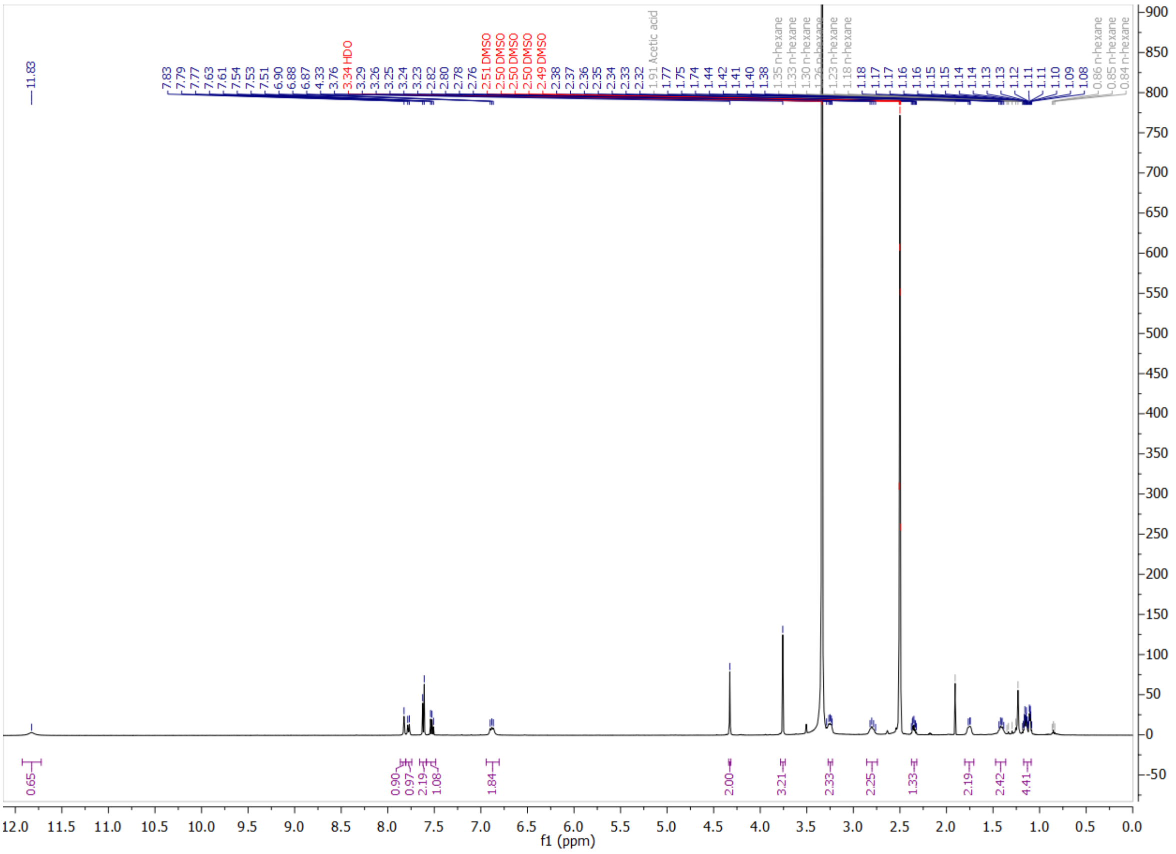

^{13}C NMR

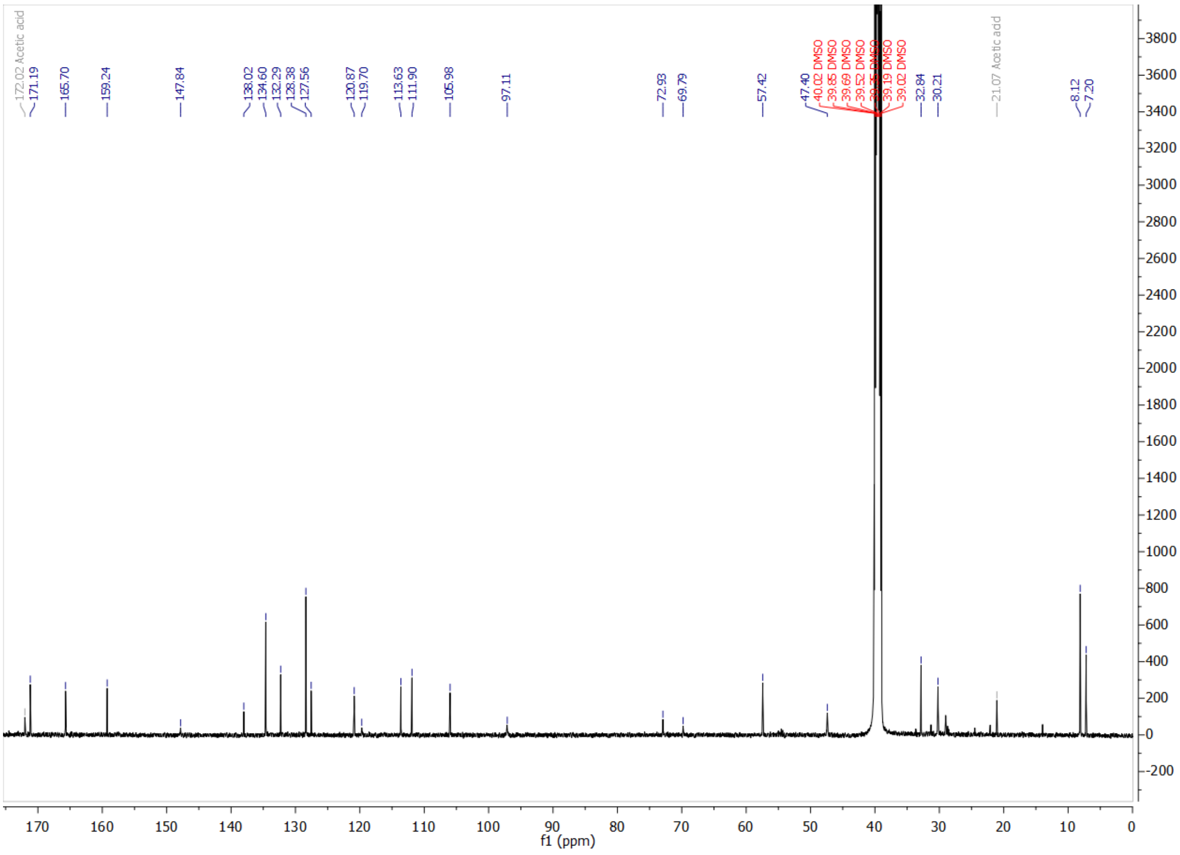

COMPOUND INFORMATION

Purity

Data File W:\analyti...\CGC_wave3_1_FirstPassB 2023-01-04 18-28-02\086-D2F-H10-LY2562175.D
Sample Name: LY2562175

```
=====================================================================
Acq. Operator   : SYSTEM                          Seq. Line :  86
Sample Operator : SYSTEM
Acq. Instrument : LCMS test                        Location :   D2F-H10
Injection Date  : 1/5/2023 10:10:47 AM                  Inj :   1
                                                 Inj Volume : Inj prog
Sequence File   : W:\analytical_LCMS_DATA\EUbOPEN\CGC_wave3_1_FirstPassB 2023-01-04 18-28-02
                  \CGC_wave3_1_FirstPassB.S
Method          : W:\analytical_LCMS_DATA\EUbOPEN\CGC_wave3_1_FirstPassB 2023-01-04 18-28-02
                  \CGL_FIRSTPASS_GENERALMETHOD_VIAL1+2_20210319.M (Sequence Method)
Last changed    : 1/25/2022 4:36:18 PM by SYSTEM
Method Info     : CGL wellplate, 0.5 uL of 10 mM DMSO, general method
```

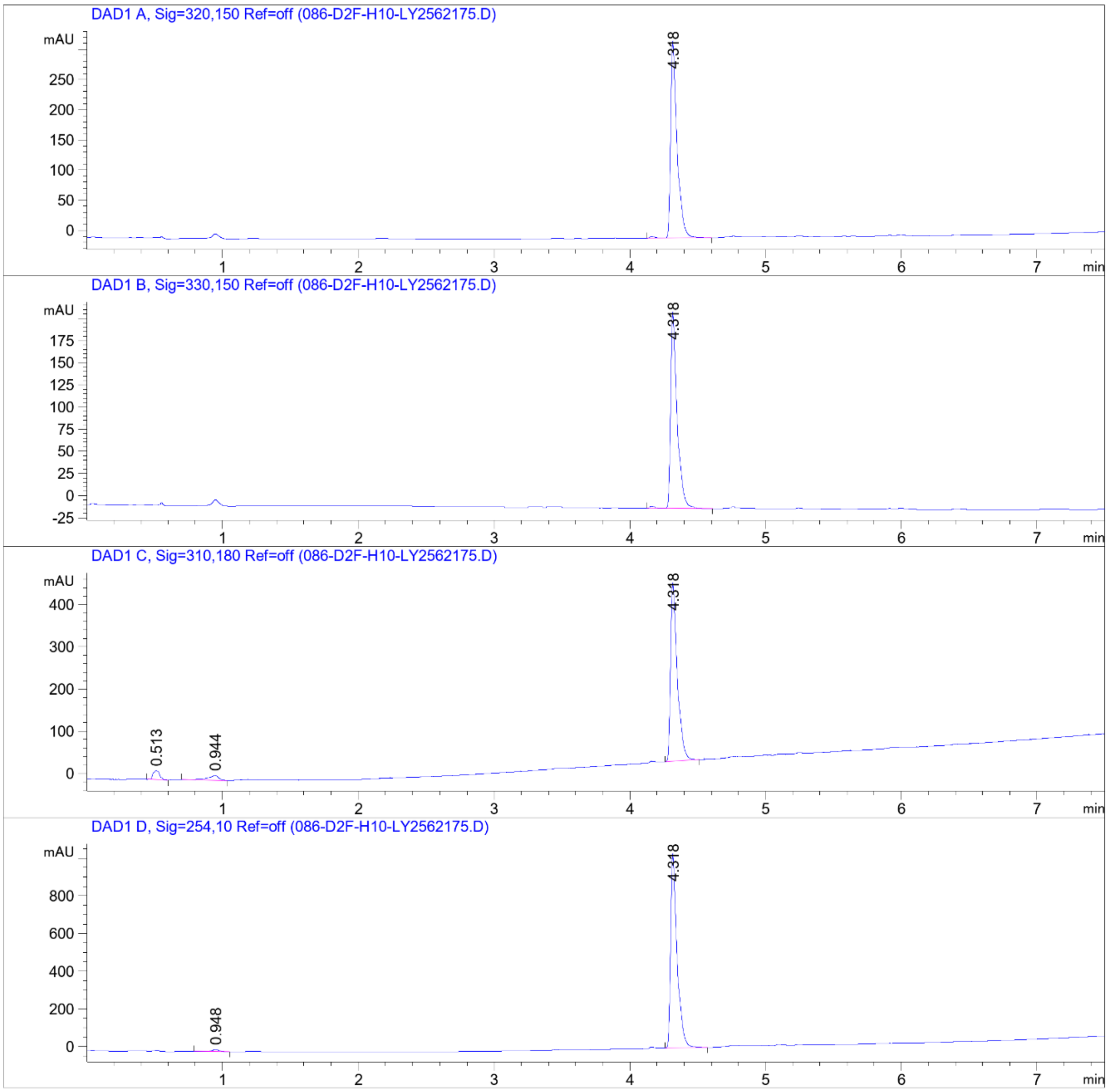

COMPOUND INFORMATION

Data File W:\analyti...\CGC_wave3_1_FirstPassB 2023-01-04 18-28-02\086-D2F-H10-LY2562175.D
Sample Name: LY2562175

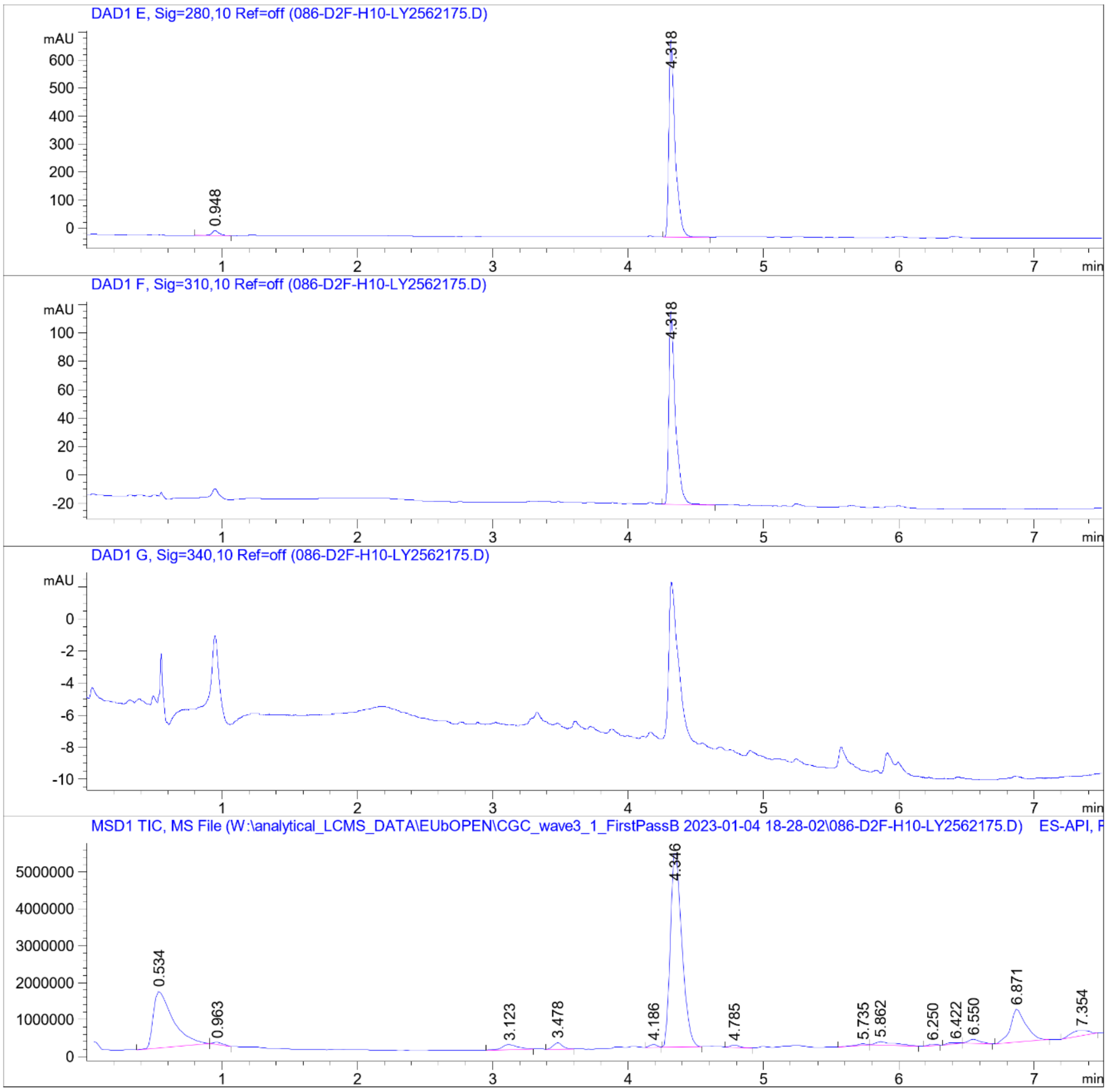

COMPOUND INFORMATION

Data File W:\analyti...\CGC_wave3_1_FirstPassB 2023-01-04 18-28-02\086-D2F-H10-LY2562175.D
Sample Name: LY2562175

MS Signal: MSD1 TIC, MS File, ES-API, Pos, Scan, Frag: 70, "POS Scan"
Spectra from peak tops.
Noise Cutoff: 1000 counts.
Reportable Ion Abundance: > 50%.
LC Signal: DAD1 A, Sig=320,150 Ref=off
Peak matching window: 0.1 min

Retention Time (LC)	LC Area	Retention Time (MS)	MS Area	Mol. Weight or Ion
-	-	0.534	15474488	157.00 I
-	-	0.963	282193	253.10 I<br>157.10 I
-	-	3.123	1123486	239.00 I<br>217.10 I<br>188.10 I
-	-	3.478	805334	369.10 I<br>367.00 I
-	-	4.186	265977	506.20 I
4.318	1077	4.346	31588666	542.10 I<br>540.10 I
-	-	4.785	285264	554.10 I<br>510.30 I<br>279.10 I<br>250.10 I<br>170.80 I<br>137.10 I
-	-	5.735	159735	280.20 I
-	-	5.862	867011	318.20 I<br>296.20 I
-	-	6.250	141678	228.20 I<br>137.10 I
-	-	6.422	182619	350.20 I<br>282.20 I<br>254.20 I<br>137.10 I
-	-	6.550	716134	507.30 I<br>485.20 I<br>280.20 I
-	-	6.871	7088273	282.20 I
-	-	7.354	1310393	400.30 I<br>282.20 I

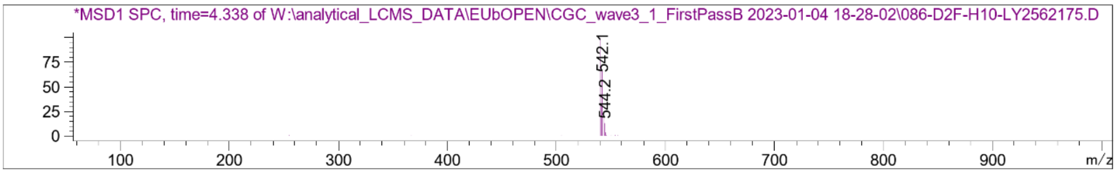

Supplement: Supplementary file 4 — Supplementary Data 1 [file 41467_2024_49493_MOESM4_ESM.zip › LY2562175.pdf]
