# Supplementary material for: Chemogenomics for NR1 nuclear hormone receptors
Source: Nat Commun. 2024 Jun 18;15:5201. doi: 10.1038/s41467-024-49493-6 (PMC11189487; doi:10.1038/s41467-024-49493-6)

COMPOUND INFORMATION

MBX-8025 sodium salt (Seladelpar)

**CAS Registry No.:**	851528-79-5
**Formal Name:**	Sodium (R)-2-(4-((2-ethoxy-3-(4-(trifluoromethyl)phenoxy)propyl)thio)-2-methylphenoxy)acetate
**EUbOPEN ID:**	EUB0001145aNa
**Molecular Formula:**	$C_{21}H_{22}F_3NaO_5S$
**Molecular Weight:**	466.45 g/mol
**Smiles:**	CCOC(COC1=CC=C(C=C1)C(F)(F)F)CSC2=CC(=C(C=C2)OCC(=O)[O-])C.[Na+]
**Recommended concentration:**	1 µM

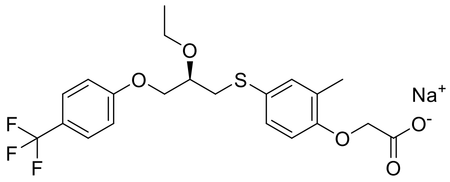

Biological activity

		Type	IC_{50}/EC_{50} [µM]	Reference
Main NR target:	NR1C2 (PPARδ)	Agonist	0.002	https://doi.org/10.1016/j.bmcl.2007.05.007
NR off-target:				

COMPOUND INFORMATION

Identity

^{1}H NMR

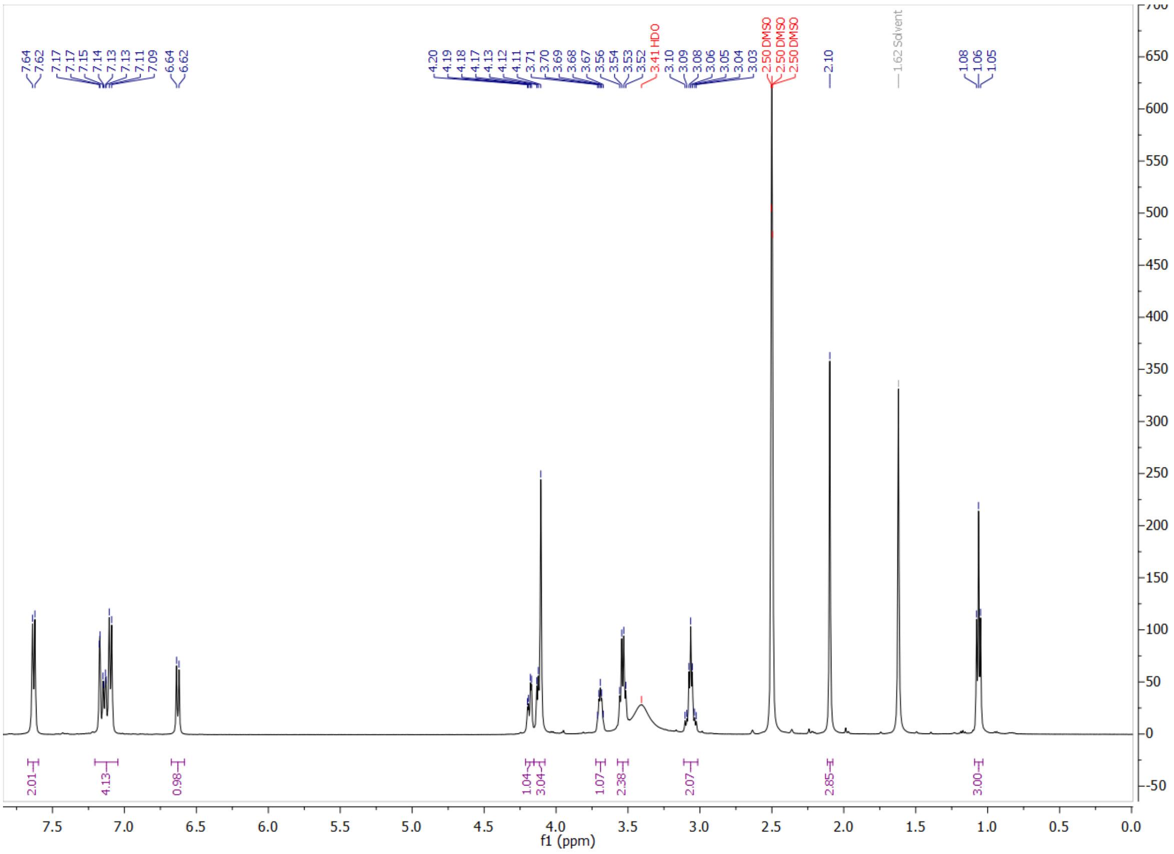

^{13}C NMR

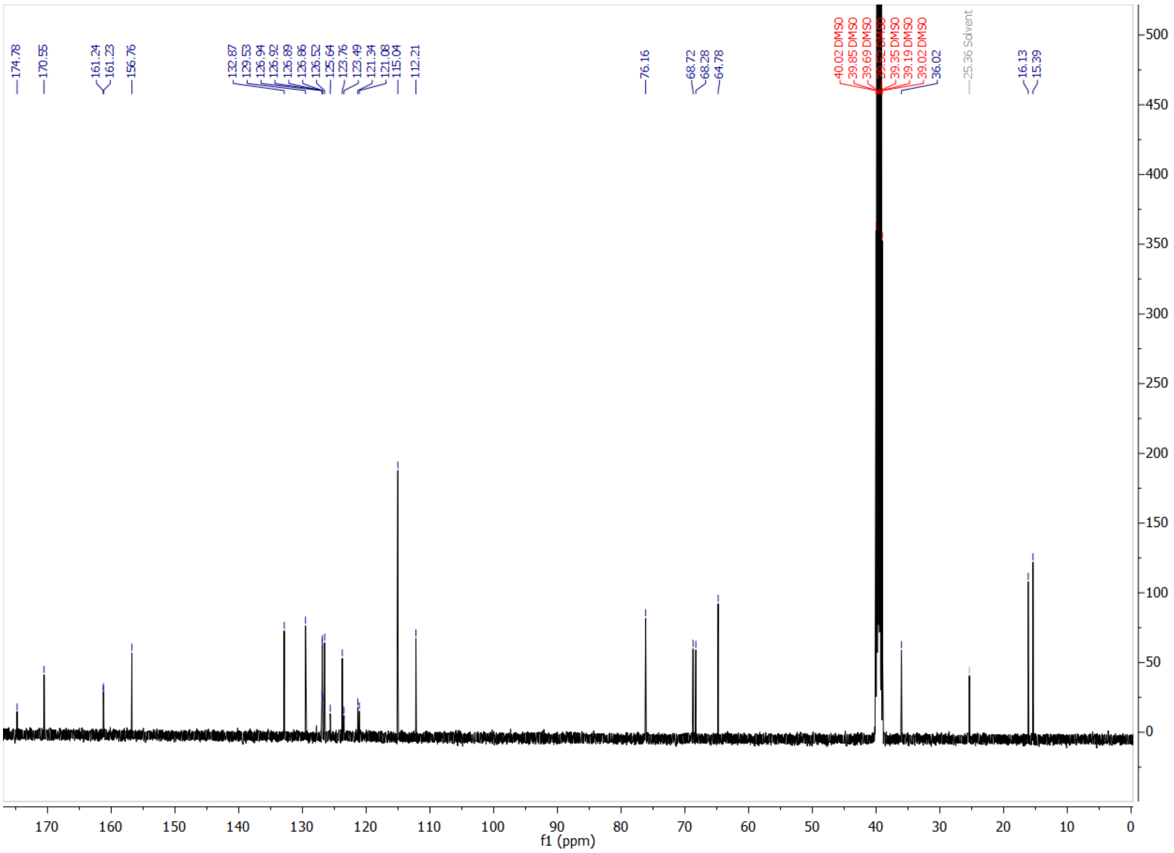

COMPOUND INFORMATION

Purity

Data File W:\analyti...EN\CGC_wave3_1_FirstPassB 2023-01-04 18-28-02\066-D2F-F3-MBX-8025.D
Sample Name: MBX-8025

```
=====================================================================
Acq. Operator   : SYSTEM                        Seq. Line :  66
Sample Operator : SYSTEM
Acq. Instrument : LCMS test                      Location :  D2F-F3
Injection Date  : 1/5/2023 6:29:36 AM                 Inj :  1
                                                Inj Volume : Inj prog
Sequence File   : W:\analytical_LCMS_DATA\EUbOPEN\CGC_wave3_1_FirstPassB 2023-01-04 18-28-02
                  \CGC_wave3_1_FirstPassB.S
Method          : W:\analytical_LCMS_DATA\EUbOPEN\CGC_wave3_1_FirstPassB 2023-01-04 18-28-02
                  \CGL_FIRSTPASS_GENERALMETHOD_VIAL1+2_20210319.M (Sequence Method)
Last changed    : 1/25/2022 4:36:18 PM by SYSTEM
Method Info     : CGL wellplate, 0.5 uL of 10 mM DMSO, general method
```

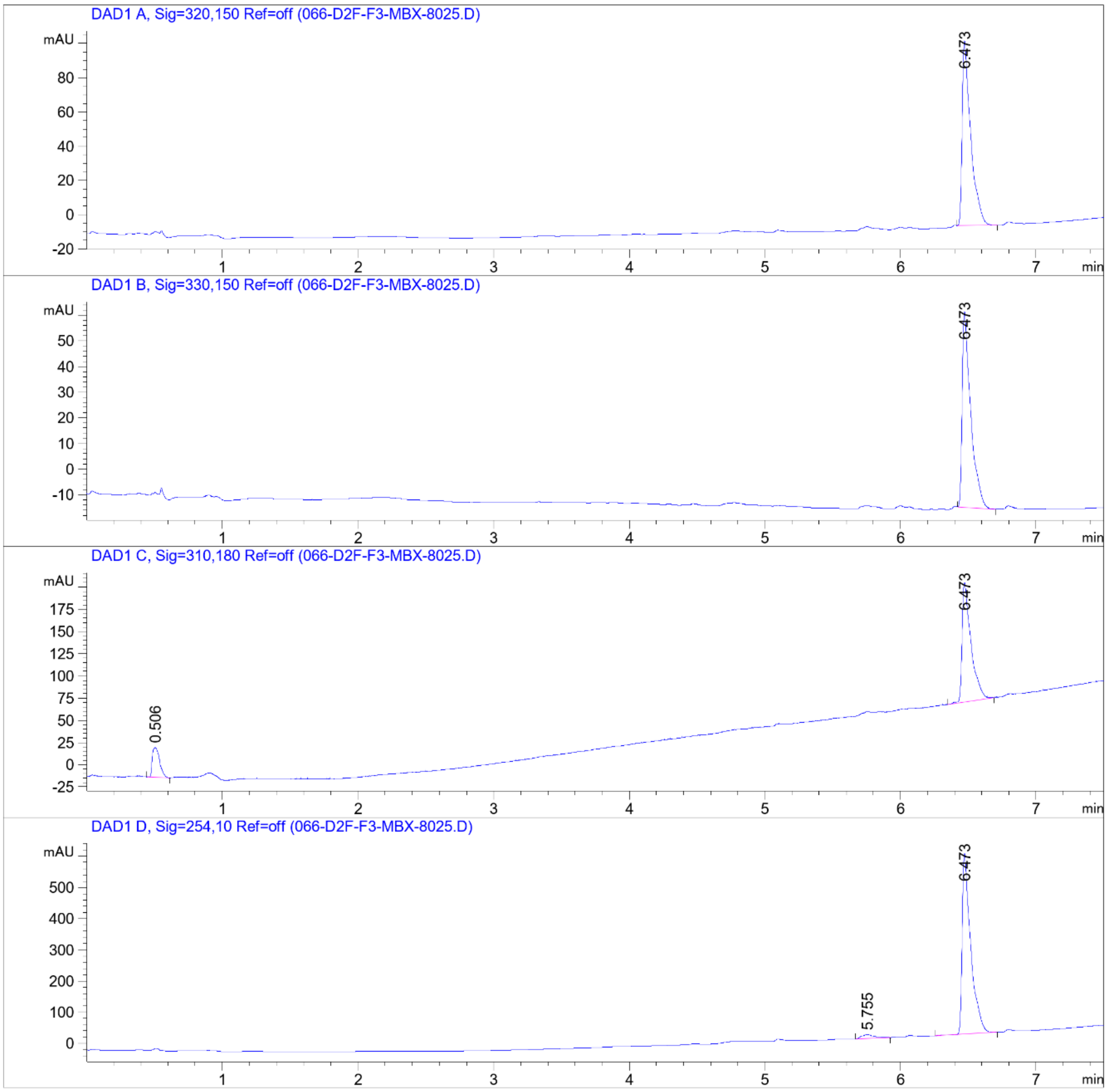

COMPOUND INFORMATION

Data File W:\analyti...EN\CGC_wave3_1_FirstPassB 2023-01-04 18-28-02\066-D2F-F3-MBX-8025.D
Sample Name: MBX-8025

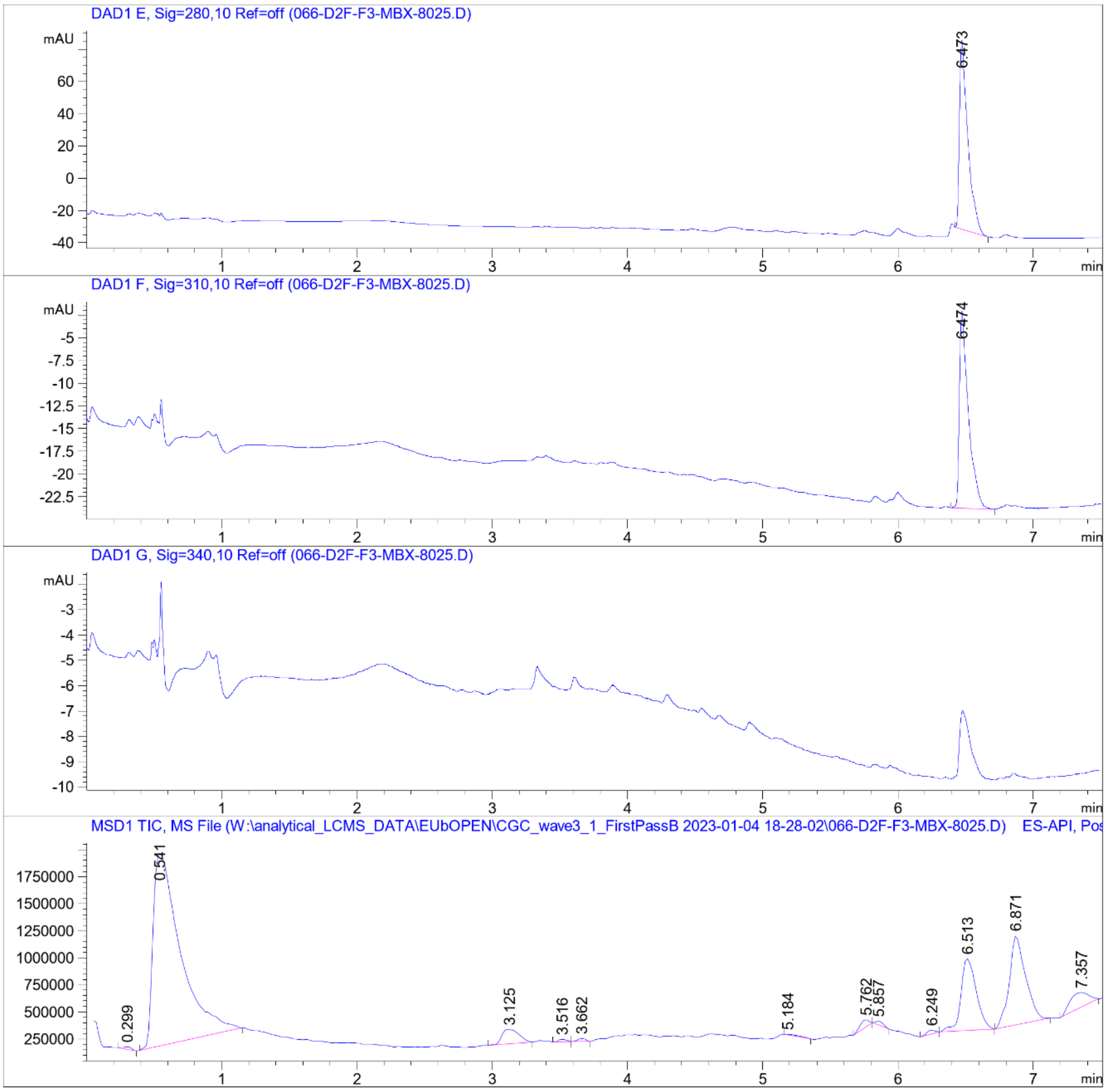

COMPOUND INFORMATION

Data File W:\analyti...EN\CGC_wave3_1_FirstPassB 2023-01-04 18-28-02\066-D2F-F3-MBX-8025.D
Sample Name: MBX-8025

MS Signal: MSD1 TIC, MS File, ES-API, Pos, Scan, Frag: 70, "POS Scan"
Spectra from peak tops.
Noise Cutoff: 1000 counts.
Reportable Ion Abundance: > 50%.
LC Signal: DAD1 A, Sig=320,150 Ref=off
Peak matching window: 0.1 min

Retention Time (LC)	LC Area	Retention Time (MS)	MS Area	Mol. Weight or Ion
-	-	0.299	103602	200.00 I
				159.00 I
-	-	0.541	25080690	157.10 I
-	-	3.125	1179300	239.10 I
				217.10 I
				188.10 I
-	-	3.516	103407	598.40 I
				554.40 I
				200.00 I
				170.90 I
				159.00 I
-	-	3.662	129922	170.80 I
				158.90 I
-	-	5.184	87712	510.30 I
				170.90 I
				137.10 I
-	-	5.762	335804	461.10 I
				280.30 I
-	-	5.857	146554	318.20 I
				296.20 I
-	-	6.249	142514	228.20 I
				137.10 I
6.473	472	6.513	5270303	507.20 I
				467.10 I
				445.10 I
				399.00 I
				379.10 I
				280.20 I
-	-	6.871	6958635	282.20 I
-	-	7.357	1366345	400.30 I
				282.30 I

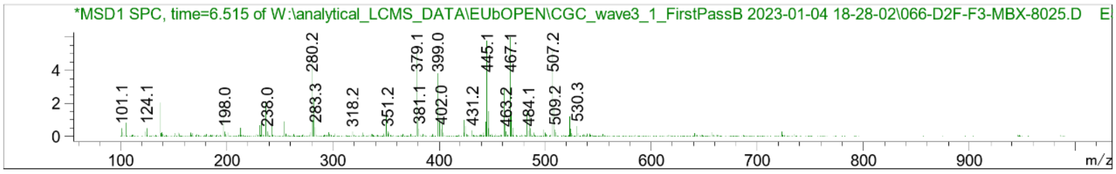

COMPOUND INFORMATION

LC-MS

M_r 444.47

MS: ESI-negative, *m/z* 443/138 (blue), *m/z* 443/161 (red), *m/z* 443/385 (green)

LC: 0.1% HCOOH/ACN (30/70)

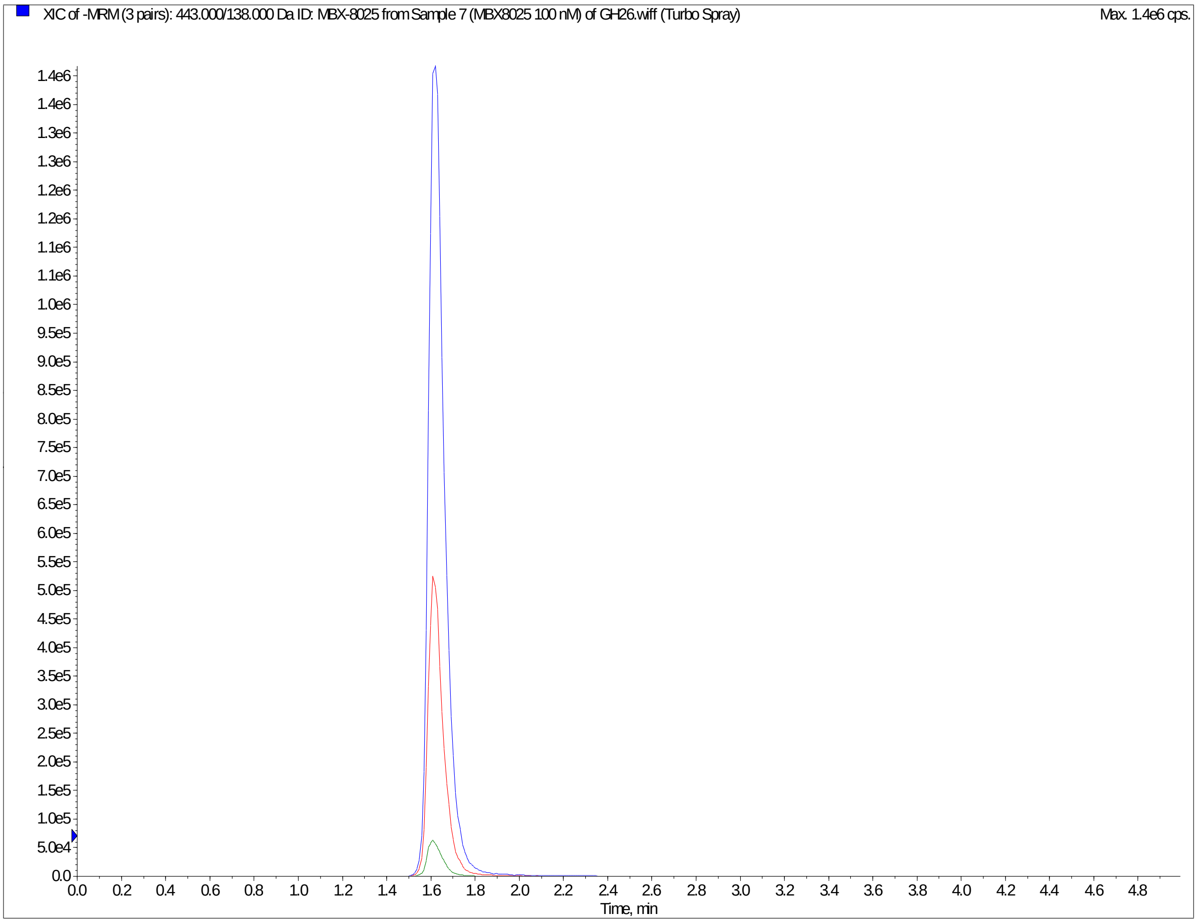

Supplement: Supplementary file 4 — Supplementary Data 1 [file 41467_2024_49493_MOESM4_ESM.zip › MBX-8025.pdf]
