# Supplementary material for: Chemogenomics for NR1 nuclear hormone receptors
Source: Nat Commun. 2024 Jun 18;15:5201. doi: 10.1038/s41467-024-49493-6 (PMC11189487; doi:10.1038/s41467-024-49493-6)

COMPOUND INFORMATION

Meclizine (dihydrochloride)

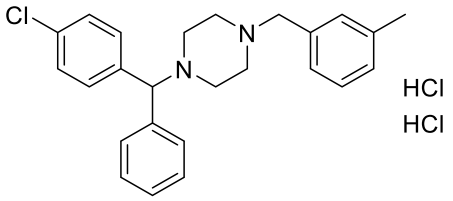

**CAS Registry No.:**	1104-22-9
**Formal Name:**	1-((4-chlorophenyl)(phenyl)methyl)-4-(3-methylbenzyl)piperazine dihydrochloride
**EUbOPEN ID**	EUB0000551aCl
**Molecular Formula:**	$C_{25}H_{29}Cl_3N_2$
**Molecular Weight:**	462.14 g/mol
**Smiles:**	CC1=CC(CN2CCN(C(C3=CC=C(Cl)C=C3)C4=CC=CC=C4)CC2)=CC=C1.Cl.Cl
**Recommended concentration:**	1 µM

Biological activity

		Type	IC_{50}/EC_{50} [µM]	Reference
Main NR target:	NR1I3 (CAR)	inv. Agonist	0.06	https://doi.org/10.1210/me.2004-0046
NR off-target:				

COMPOUND INFORMATION

Identity

^{1}H NMR

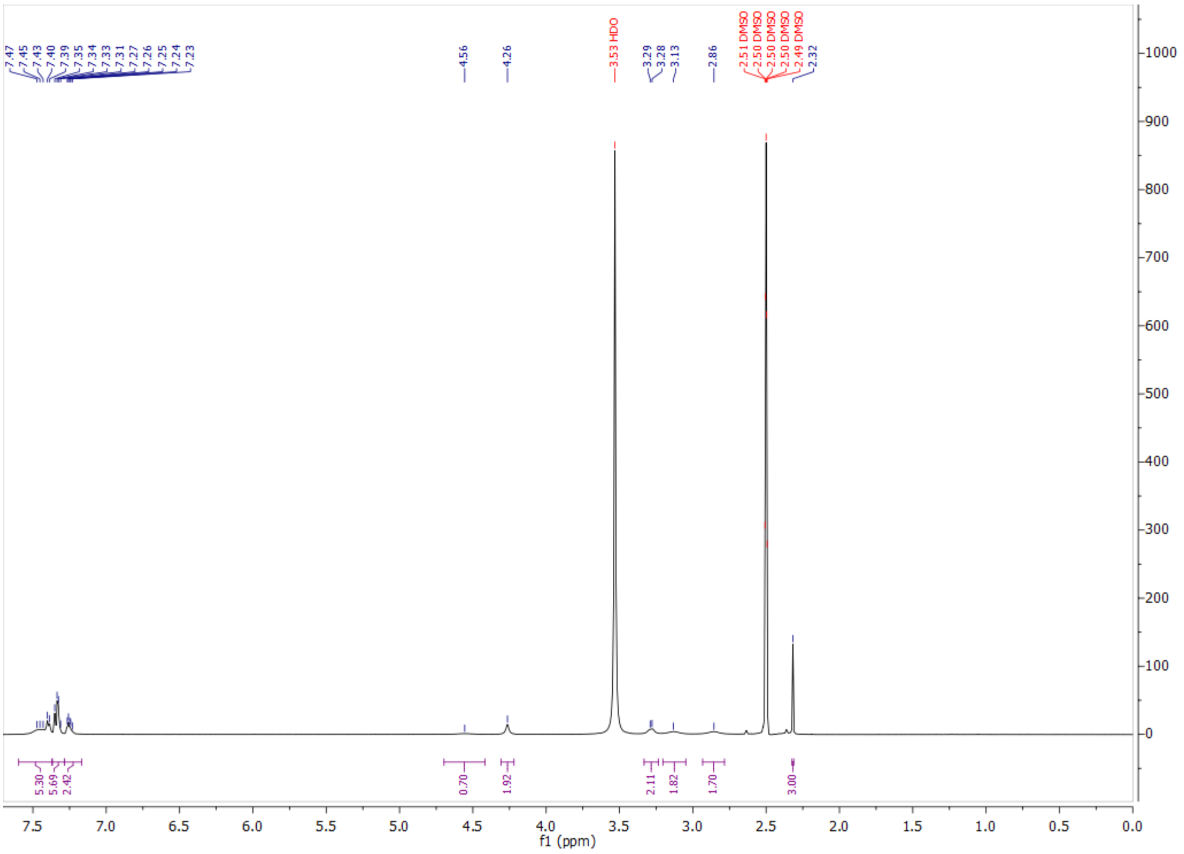

^{13}C NMR

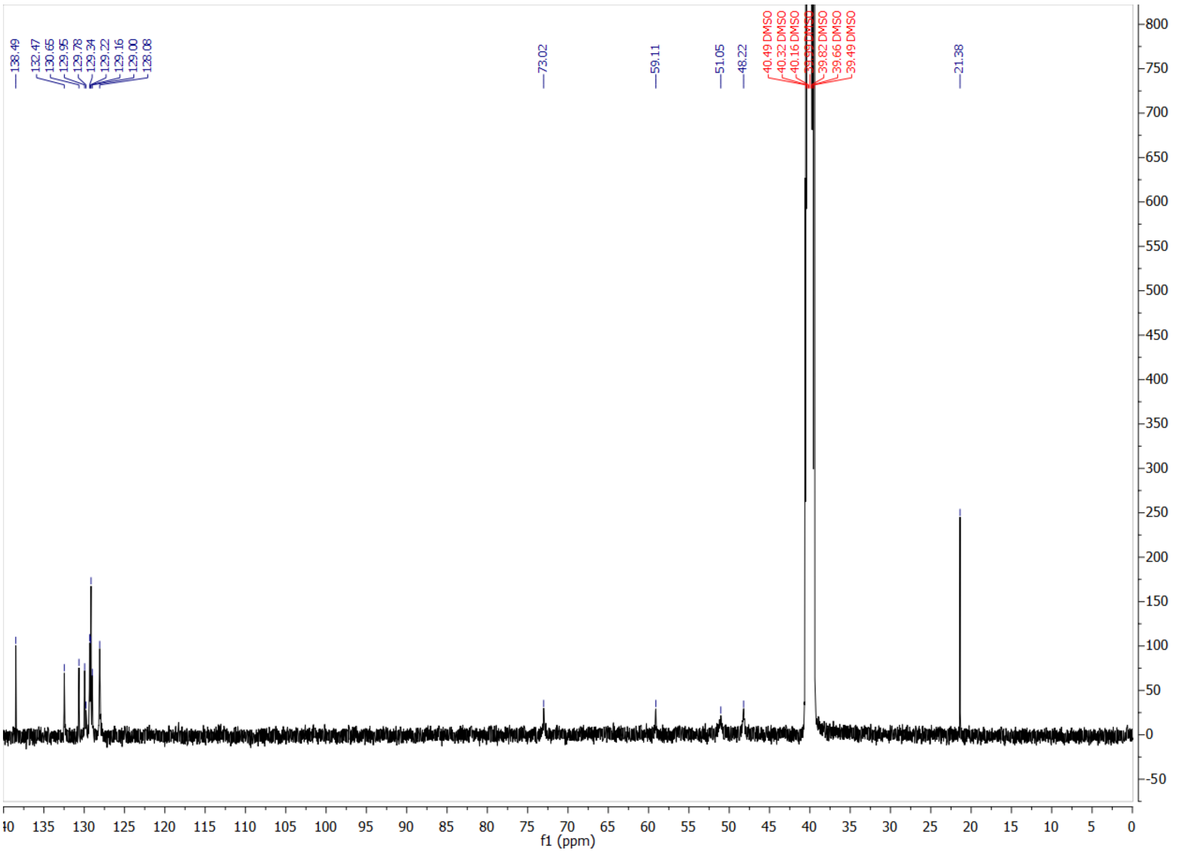

COMPOUND INFORMATION

Purity

```
Data File W:\analyti...condPass 2021-03-23 18-53-53\026-D2F-G7-meclizine dihydrochloride.D
Sample Name: meclizine dihydrochloride

    =====================================================================
    Acq. Operator   : SYSTEM                        Seq. Line :  26
    Sample Operator : SYSTEM
    Acq. Instrument : LCMS test                      Location :   D2F-G7
    Injection Date  : 3/23/2021 11:34:52 PM               Inj :   1
                                                Inj Volume : Inj prog
    Sequence File   : W:\analytical_LCMS_DATA\EUbOPEN\CGC_ECHO1-2_SecondPass 2021-03-23 18-53-53
                      \CGC_ECHO1-2_SecondPass.S
    Method          : W:\analytical_LCMS_DATA\EUbOPEN\CGC_ECHO1-2_SecondPass 2021-03-23 18-53-53
                      \CGL_SECONDPASS_NONPOLCOMP_VIAL2+4_20210323.M (Sequence Method)
    Last changed    : 3/23/2021 6:50:05 PM by SYSTEM
    Method Info     : CGL wellplate, 0.5 uL of 10 mM DMSO. Dilution with MeCN only (9+9 uL)
```

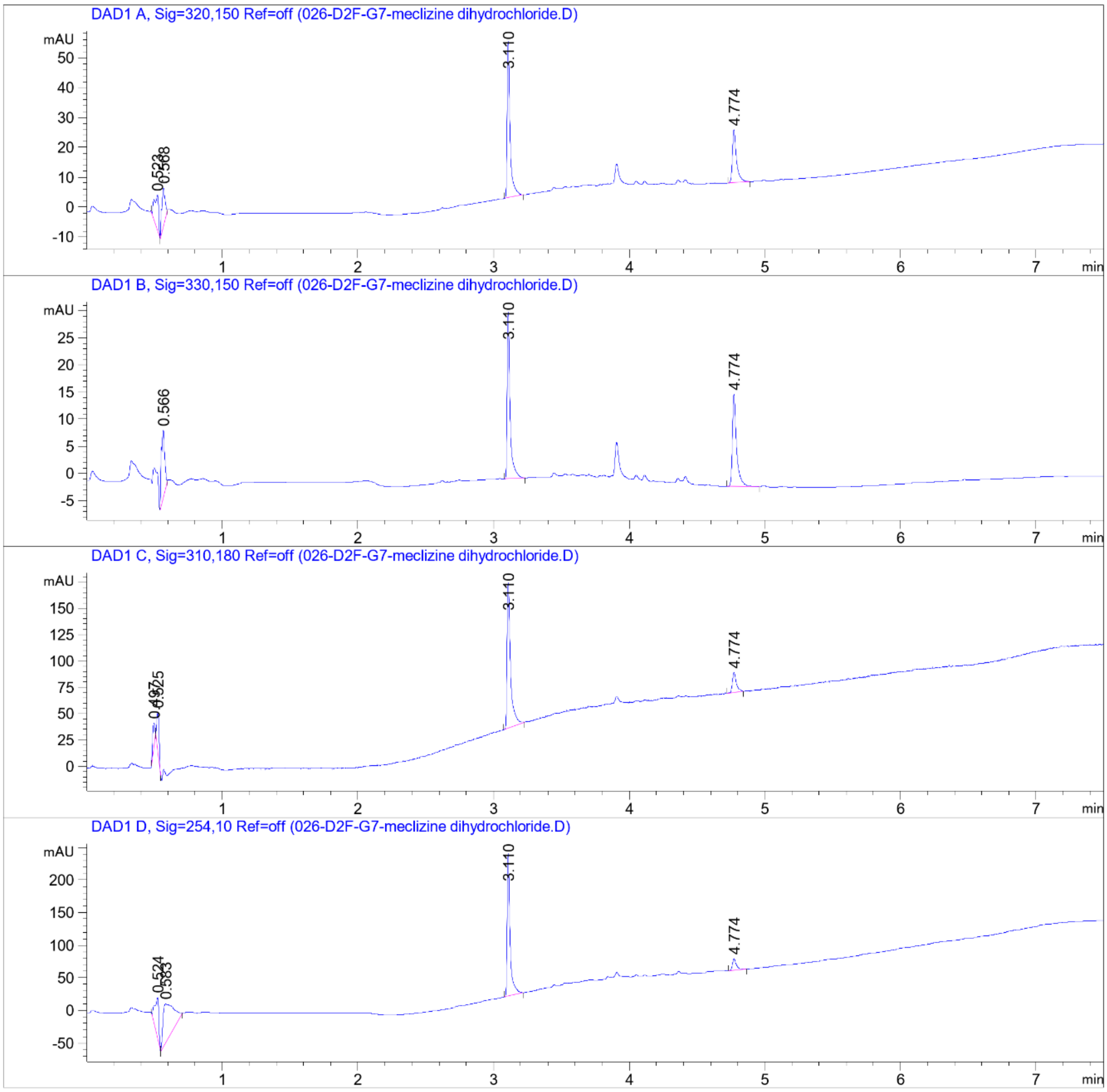

COMPOUND INFORMATION

Data File W:\analyti...condPass 2021-03-23 18-53-53\026-D2F-G7-meclizine dihydrochloride.D
Sample Name: meclizine dihydrochloride

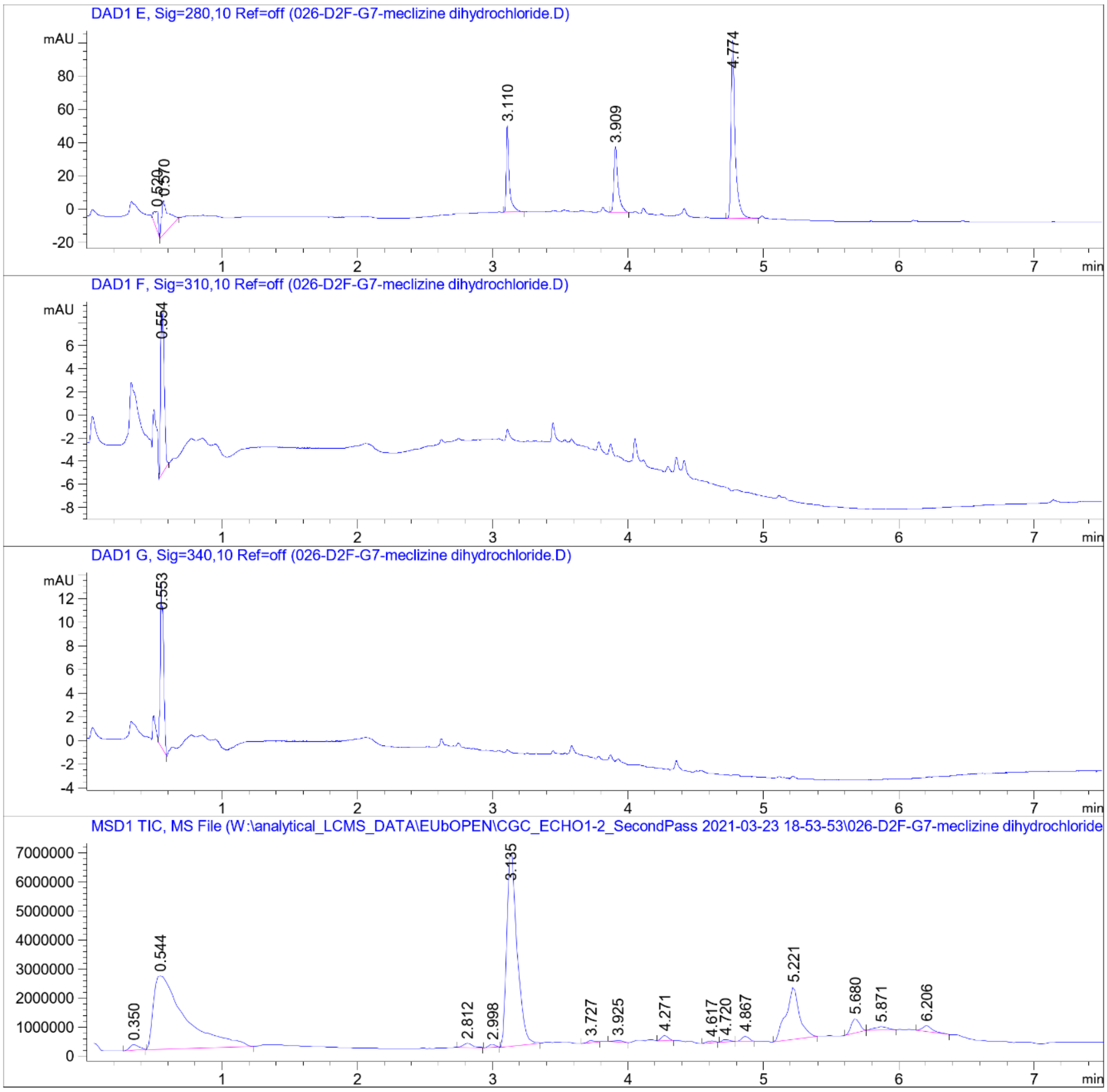

COMPOUND INFORMATION

Data File W:\analyti...condPass 2021-03-23 18-53-53\026-D2F-G7-meclizine dihydrochloride.D
Sample Name: meclizine dihydrochloride

MS Signal: MSD1 TIC, MS File, ES-API, Pos, Scan, Frag: 70, "POS Scan"
Spectra from peak tops.
Noise Cutoff: 1000 counts.
Reportable Ion Abundance: > 50%.
LC Signal: DAD1 A, Sig=320,150 Ref=off
Peak matching window: 0.1 min

Retention Time (LC)	LC Area	Retention Time (MS)	MS Area	Mol. Weight or Ion
-	-	0.350	866279	200.00 I<br>183.00 I<br>159.00 I
0.523	24	0.544	41320044	157.10 I
0.568	21	-	-	
-	-	2.812	693982	217.10 I
-	-	2.998	340473	274.20 I
3.110	79	3.135	35698364	391.20 I
-	-	3.727	276232	391.20 I<br>214.10 I
-	-	3.925	275017	512.50 I
-	-	4.271	645780	296.20 I
-	-	4.617	221542	228.20 I
4.774	37	4.720	291088	254.20 I
-	-	4.867	644958	280.30 I
-	-	5.221	12461739	282.30 I
-	-	5.680	2044310	359.30 I<br>284.30 I<br>282.30 I
-	-	5.871	762513	400.30 I<br>282.30 I
-	-	6.206	988690	338.30 I<br>282.20 I

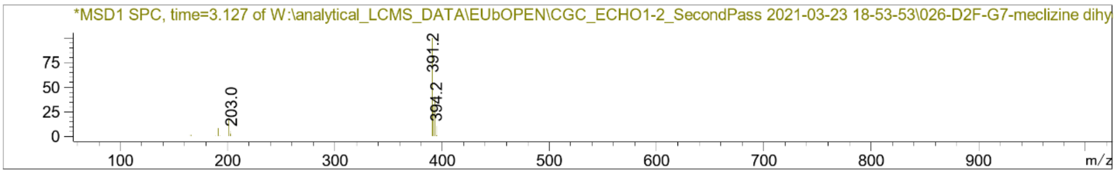

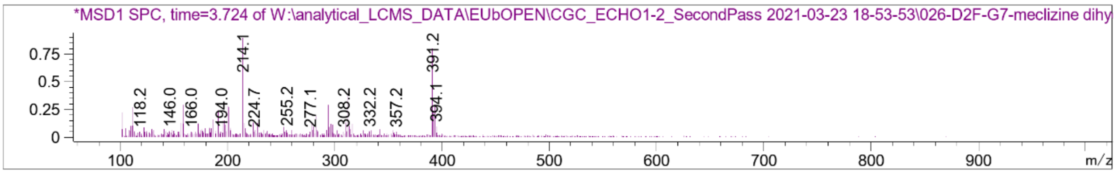

Supplement: Supplementary file 4 — Supplementary Data 1 [file 41467_2024_49493_MOESM4_ESM.zip › Meclizine.pdf]
