# Supplementary material for: Chemogenomics for NR1 nuclear hormone receptors
Source: Nat Commun. 2024 Jun 18;15:5201. doi: 10.1038/s41467-024-49493-6 (PMC11189487; doi:10.1038/s41467-024-49493-6)

COMPOUND INFORMATION

ML-209

**CAS Registry No.:**	1334526-14-5
**Formal Name:**	3-(benzo[d][1,3]dioxol-5-yl)-1-((3S,5R)-3,5-dimethylpiperidin-1-yl)-3-(2-hydroxy-4,6-dimethoxyphenyl)propan-1-one
**EUBOPEN ID:**	EUB0001161a
**Molecular Formula:**	$C_{25}H_{31}NO_6$
**Molecular Weight:**	441.52 g/mol
**Smiles:**	COC1=CC(OC)=C(C(CC(N2C[C@@H](C)C[C@@H](C)C2)=O)C3=CC=C4OCOC4=C3)C(O)=C1
**Recommended concentration:**	1 & 10 µM

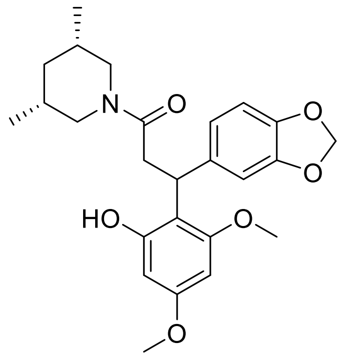

Biological activity

		Type	IC_{50}/EC_{50} [µM]	Reference
Main NR target:	NR1F2 (RORβ)	inv. Agonist	1.3	inhouse
	NR1F3 (RORγ)	inv. Agonist	0.20	inhouse
NR off-target @ 10 µM:	NR1F1 (RORα)	inv. Agonist	84% rem. act.	inhouse

COMPOUND INFORMATION

Identity

^{1}H NMR

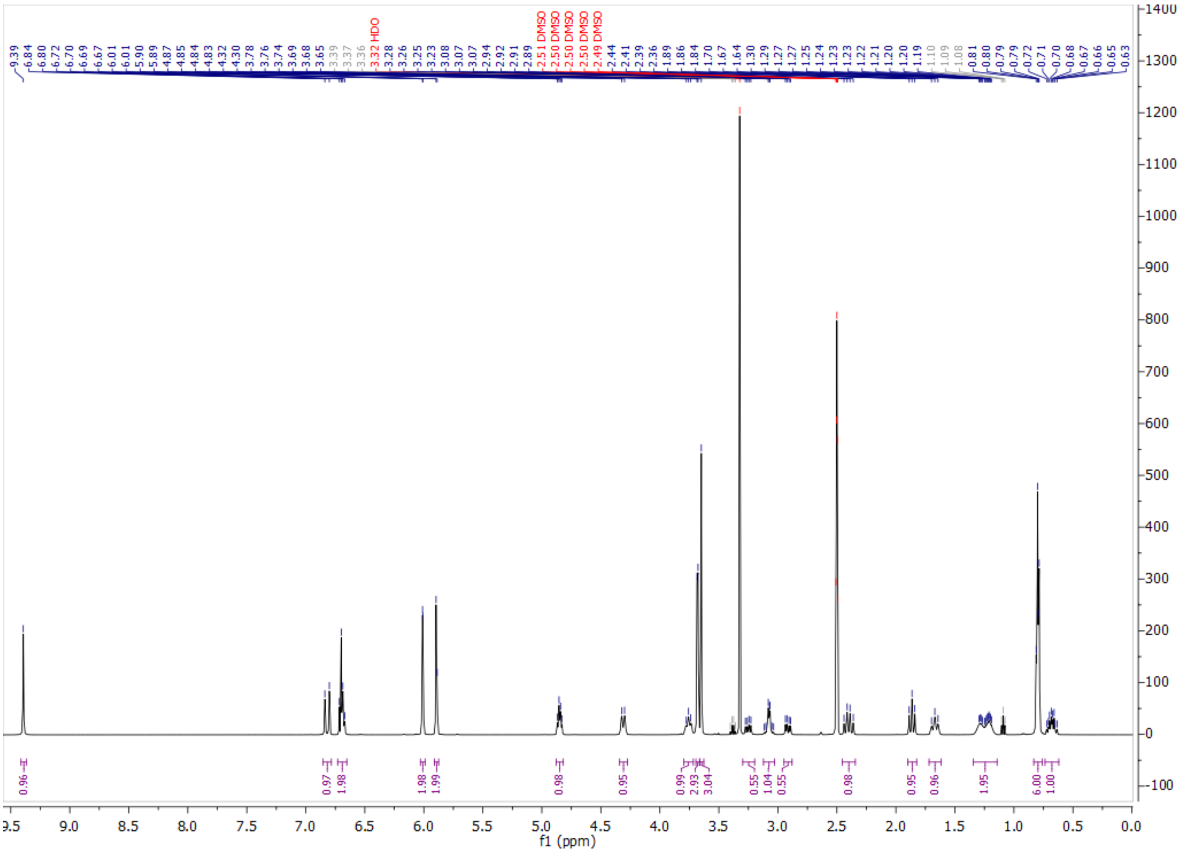

^{13}C NMR

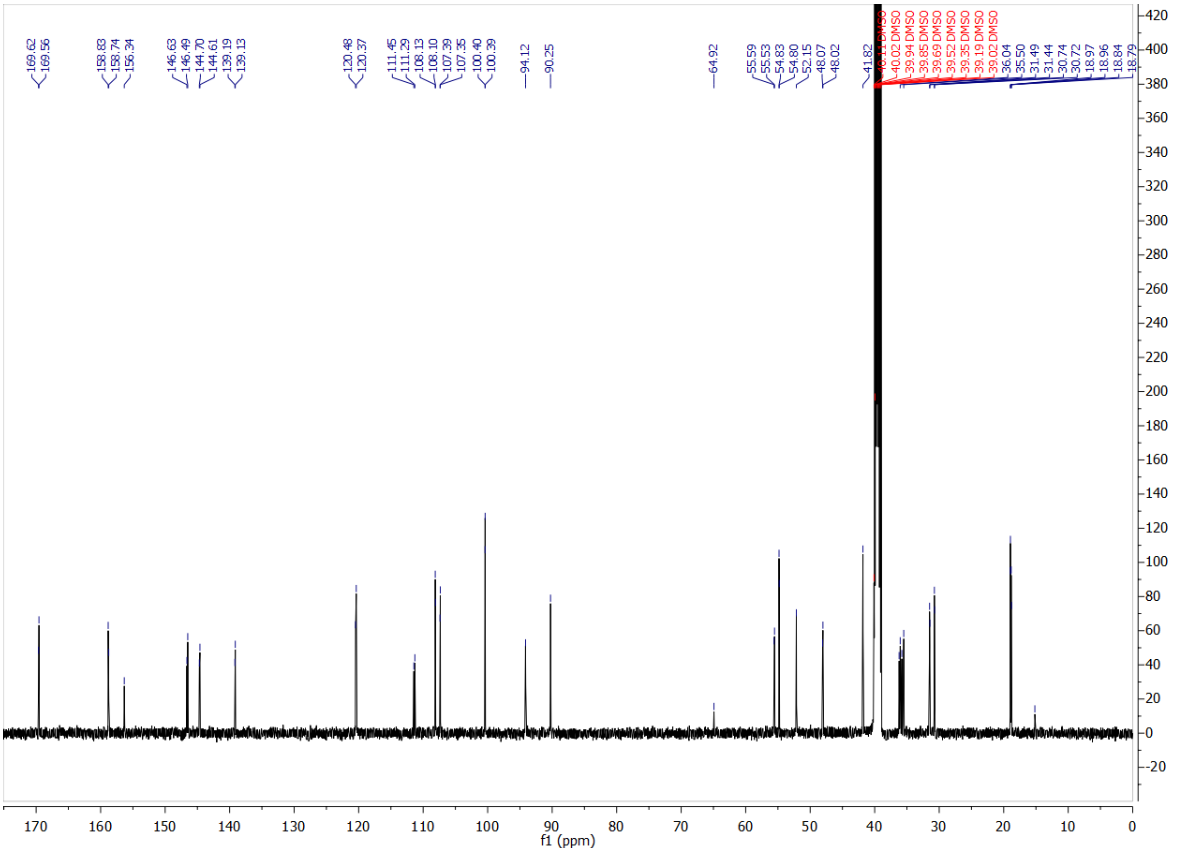

COMPOUND INFORMATION

Purity

Data File W:\analyti...OPEN\CGC_wave3_1_FirstPassB 2023-01-04 18-28-02\082-D2F-G7-ML-209.D
Sample Name: ML-209

```
=====================================================================
Acq. Operator   : SYSTEM                          Seq. Line :  82
Sample Operator : SYSTEM
Acq. Instrument : LCMS test                        Location :   D2F-G7
Injection Date  : 1/5/2023 9:26:32 AM                   Inj :   1
                                                 Inj Volume : Inj prog
Sequence File   : W:\analytical_LCMS_DATA\EUbOPEN\CGC_wave3_1_FirstPassB 2023-01-04 18-28-02
                  \CGC_wave3_1_FirstPassB.S
Method          : W:\analytical_LCMS_DATA\EUbOPEN\CGC_wave3_1_FirstPassB 2023-01-04 18-28-02
                  \CGL_FIRSTPASS_GENERALMETHOD_VIAL1+2_20210319.M (Sequence Method)
Last changed    : 1/25/2022 4:36:18 PM by SYSTEM
Method Info     : CGL wellplate, 0.5 uL of 10 mM DMSO, general method
```

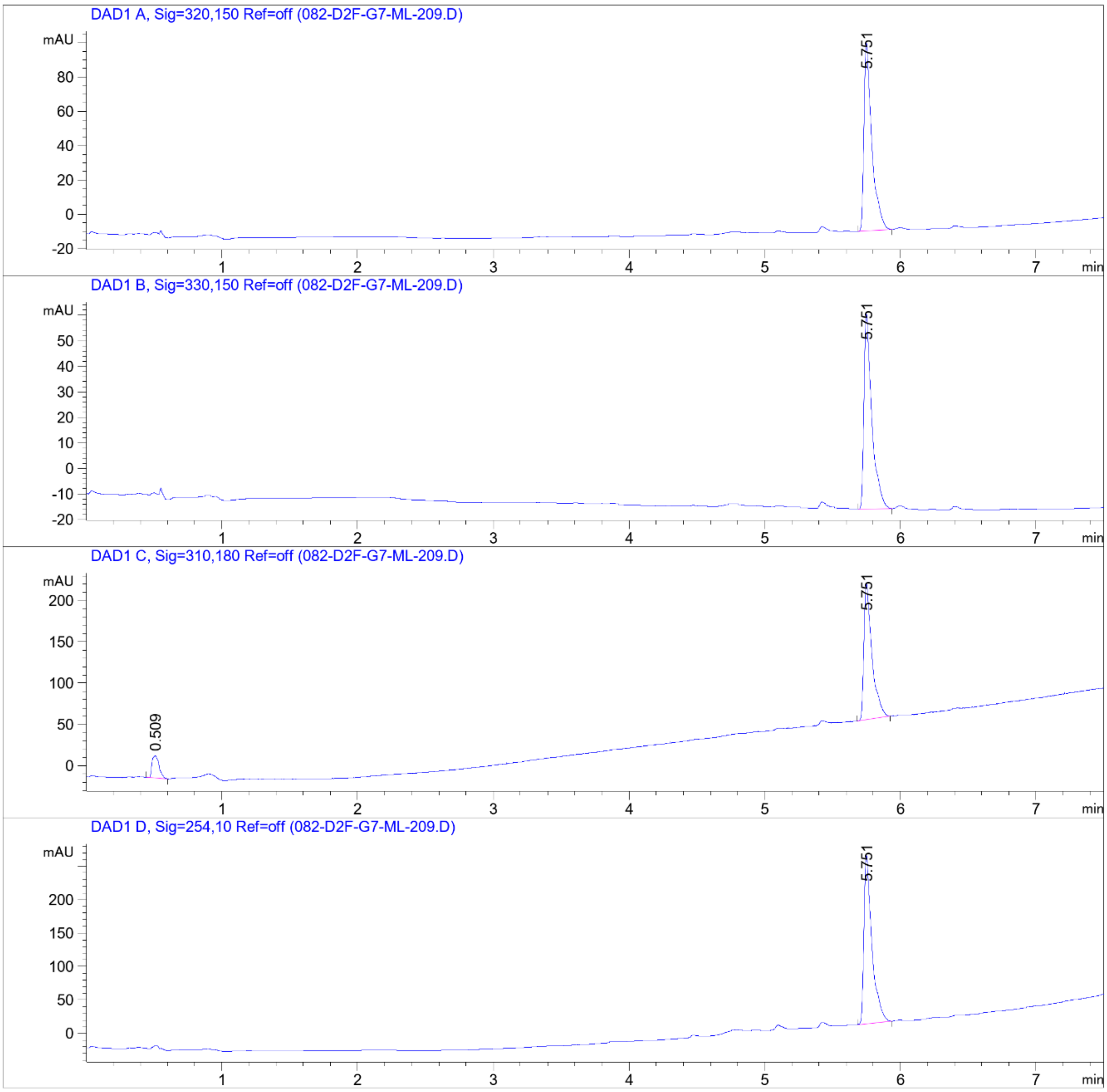

COMPOUND INFORMATION

Data File W:\analyti...OPEN\CGC_wave3_1_FirstPassB 2023-01-04 18-28-02\082-D2F-G7-ML-209.D
Sample Name: ML-209

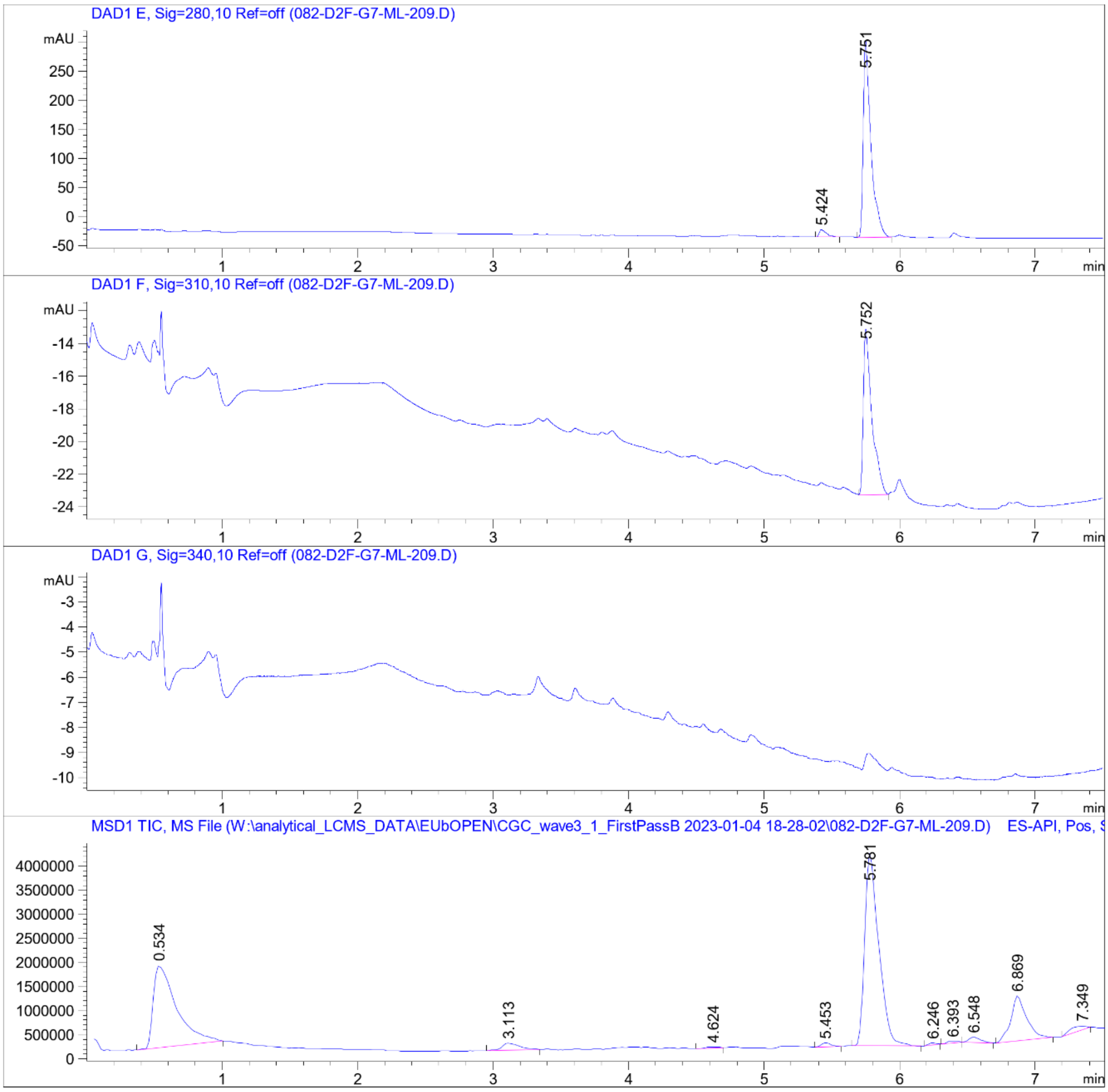

COMPOUND INFORMATION

Data File W:\analyti...OPEN\CGC_wave3_1_FirstPassB 2023-01-04 18-28-02\082-D2F-G7-ML-209.D
Sample Name: ML-209

MS Signal: MSD1 TIC, MS File, ES-API, Pos, Scan, Frag: 70, "POS Scan"
Spectra from peak tops.
Noise Cutoff: 1000 counts.
Reportable Ion Abundance: > 50%.
LC Signal: DAD1 A, Sig=320,150 Ref=off
Peak matching window: 0.1 min

Retention Time (LC)	LC Area	Retention Time (MS)	MS Area	Mol. Weight or Ion
-	-	0.534	20281808	157.00 I
-	-	3.113	1294489	239.10 I
				217.10 I
-	-	4.624	134280	510.30 I
				170.90 I
				158.10 I
				137.10 I
-	-	5.453	424767	510.30 I
				351.00 I
				329.10 I
				207.00 I
				137.10 I
5.751	441	5.781	28893416	442.20 I
-	-	6.246	143603	228.20 I
				137.10 I
-	-	6.393	195335	254.20 I
-	-	6.548	657064	507.30 I
				280.20 I
-	-	6.869	7581119	282.30 I
-	-	7.349	862882	400.30 I
				282.20 I

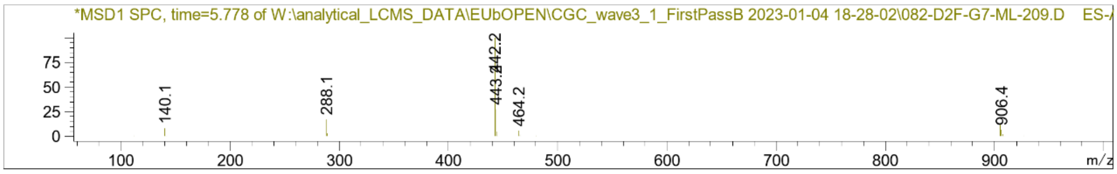

COMPOUND INFORMATION

Biological activity

ML-209
RORβ - IC_{50} 1.3 ± 0.1 µM
0.12 ± 0.06 rem. activity

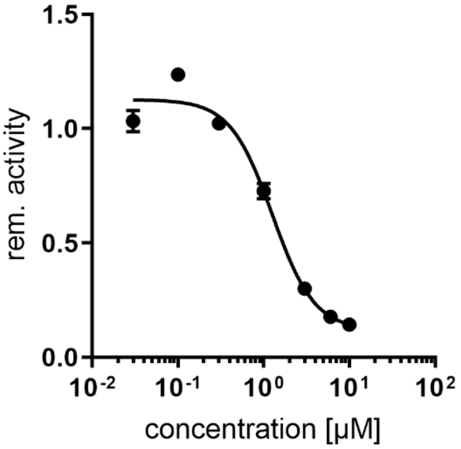

ML-209
RORγ - IC_{50} 0.20 ± 0.02 µM
0.00 ± 0.03 rem. activity

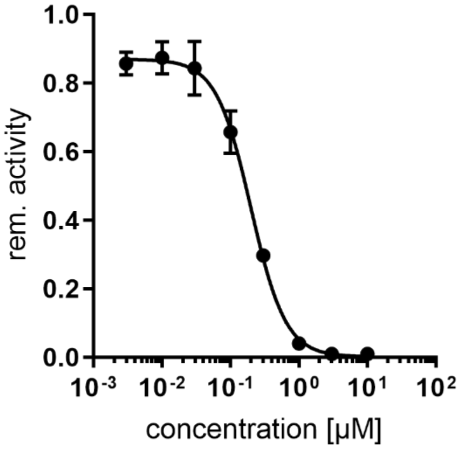

Supplement: Supplementary file 4 — Supplementary Data 1 [file 41467_2024_49493_MOESM4_ESM.zip › ML-209.pdf]
