# Supplementary material for: Chemogenomics for NR1 nuclear hormone receptors
Source: Nat Commun. 2024 Jun 18;15:5201. doi: 10.1038/s41467-024-49493-6 (PMC11189487; doi:10.1038/s41467-024-49493-6)

COMPOUND INFORMATION

Muraglitazar

**CAS Registry No.:**	331741-94-7
**Formal Name:**	N-((4-methoxyphenoxy)carbonyl)-N-(4-(2-(5-methyl-2-phenyloxazol-4-yl)ethoxy)benzyl)glycine
**EUbOPEN ID:**	EUB0001140a
**Molecular Formula:**	$C_{29}H_{28}N_2O_7$
**Molecular Weight:**	516.55 g/mol
**Smiles:**	CC1=C(N=C(O1)C2=CC=CC=C2)CCOC3=CC=C(C=C3)CN(CC(=O)O)C(=O)OC4=CC=C(C=C4)OC
**Recommended concentration:**	1 µM

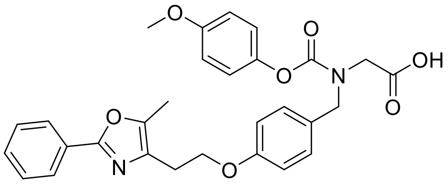

Biological activity

		Type	IC_{50}/EC_{50} [µM]	Reference
Main NR target:	NR1C1 (PPARα)	Agonist	0.05	inhouse
	NR1C3 (PPARγ)	Agonist	0.016	
NR off-target:	NR1C2 (PPARδ)	Agonist	7-fold act.	inhouse

COMPOUND INFORMATION

Identity

^{1}H NMR

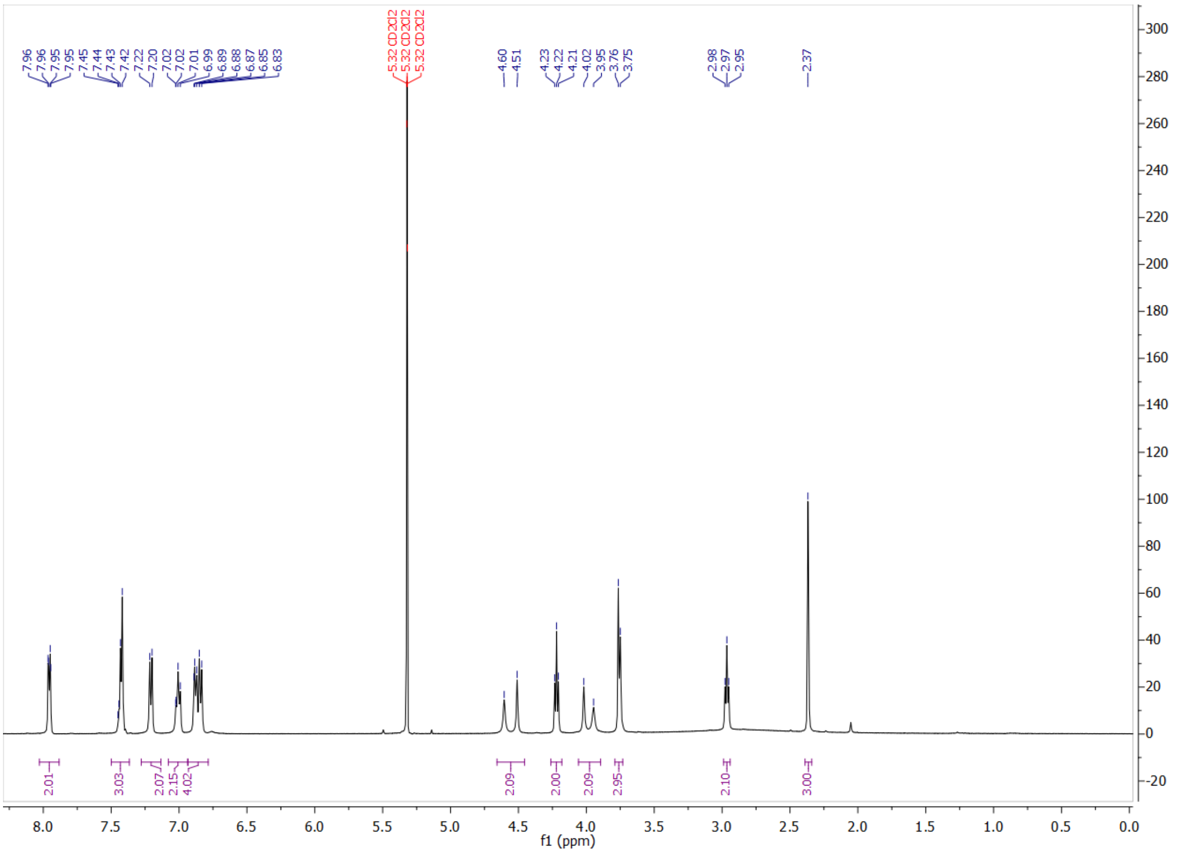

^{13}C NMR

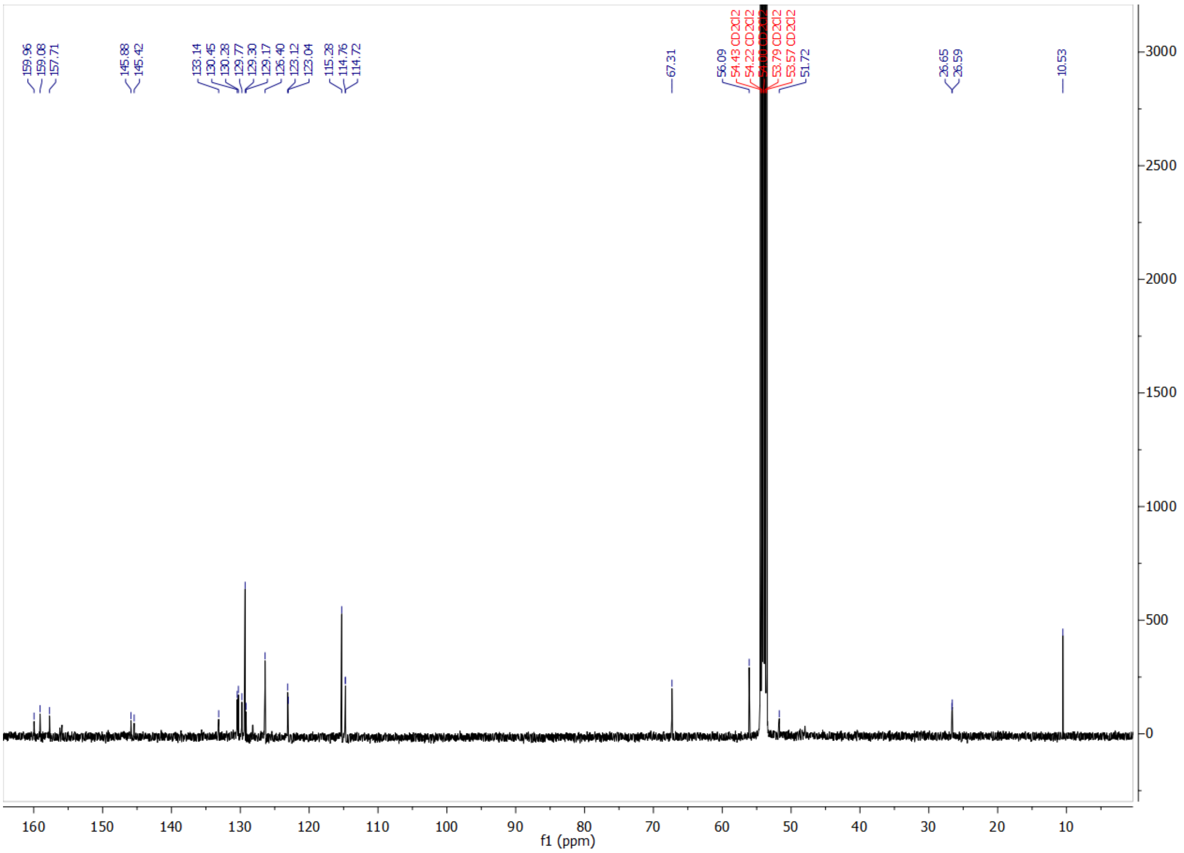

COMPOUND INFORMATION

Purity

M_r 516.55

MS: ESI-positive, *m/z* 517/186 (blue), *m/z* 517/292 (red)

LC: 0.1% HCOOH/ACN (20/80)

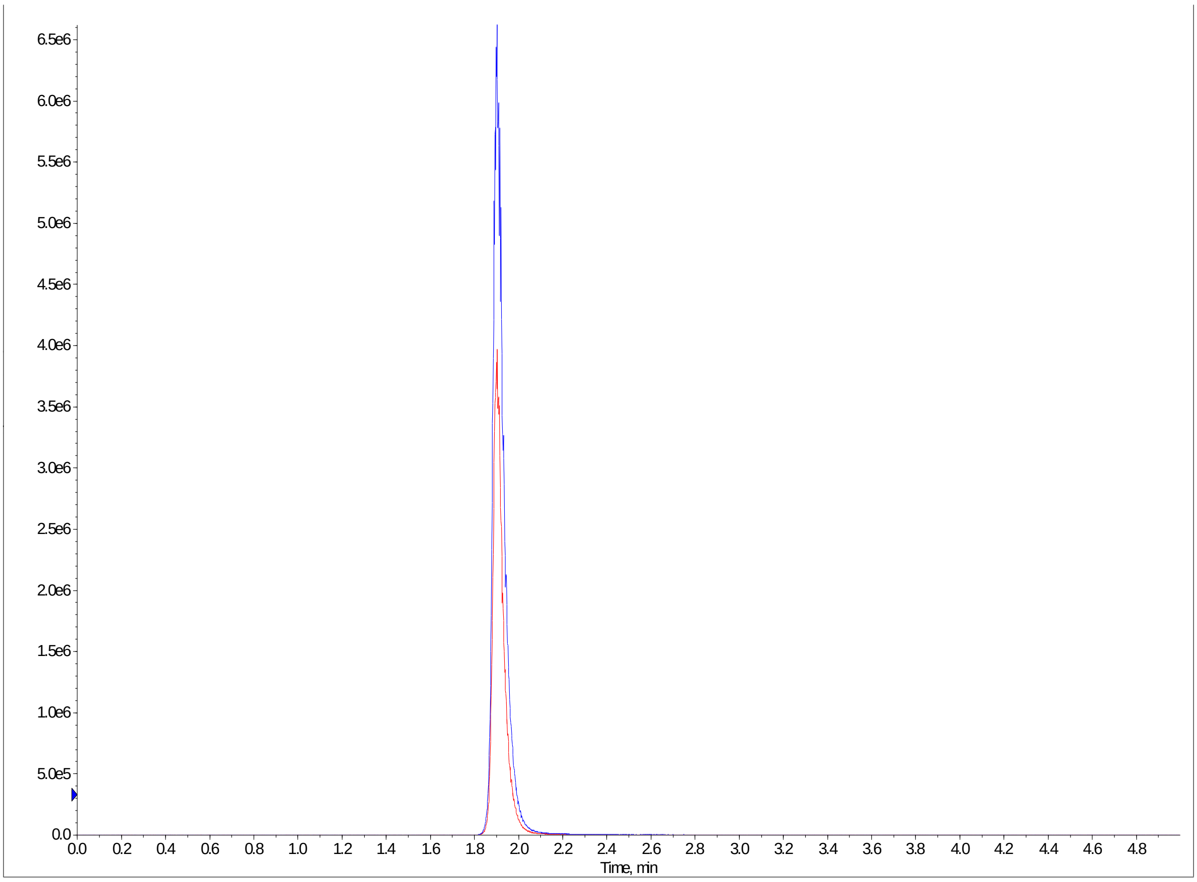

COMPOUND INFORMATION

LC-UV

LC: 0.1% HCOOH/ACN (40/60)

DAD: 210, 230, 240, 254 (XWC), 280 nm

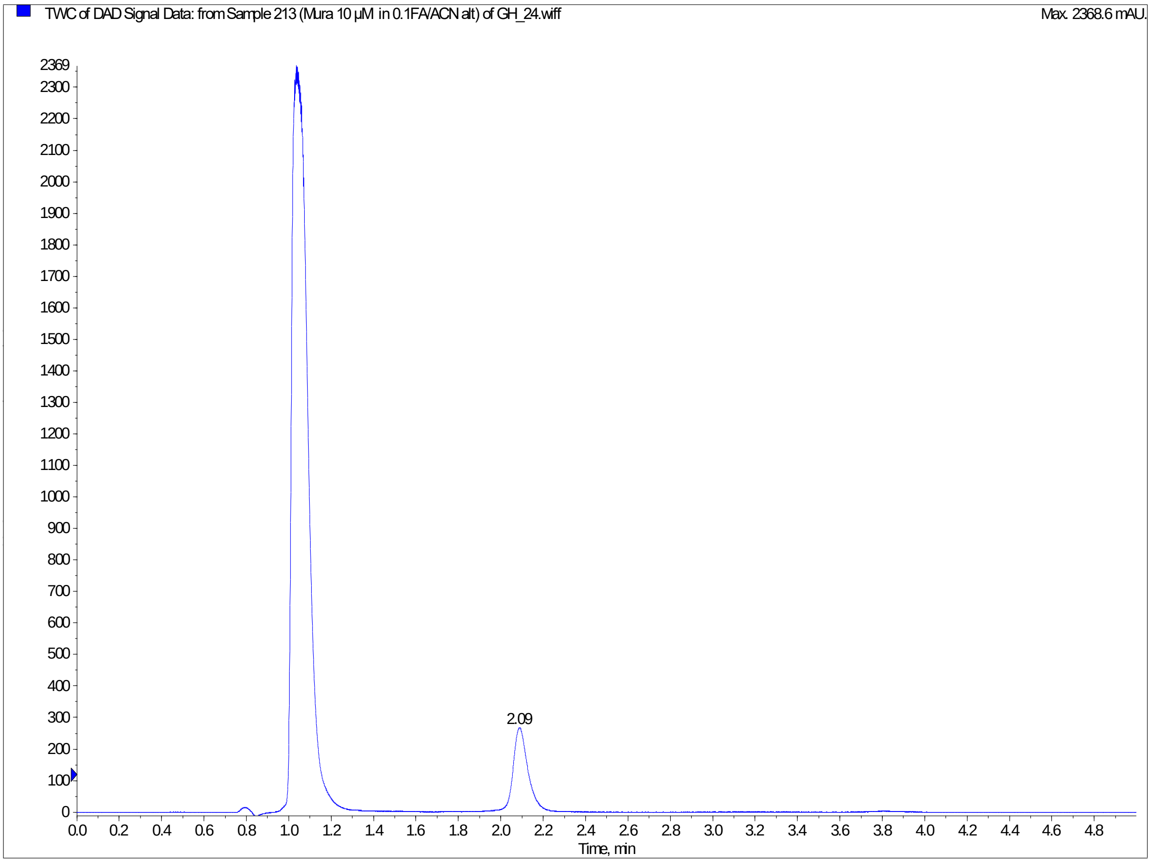

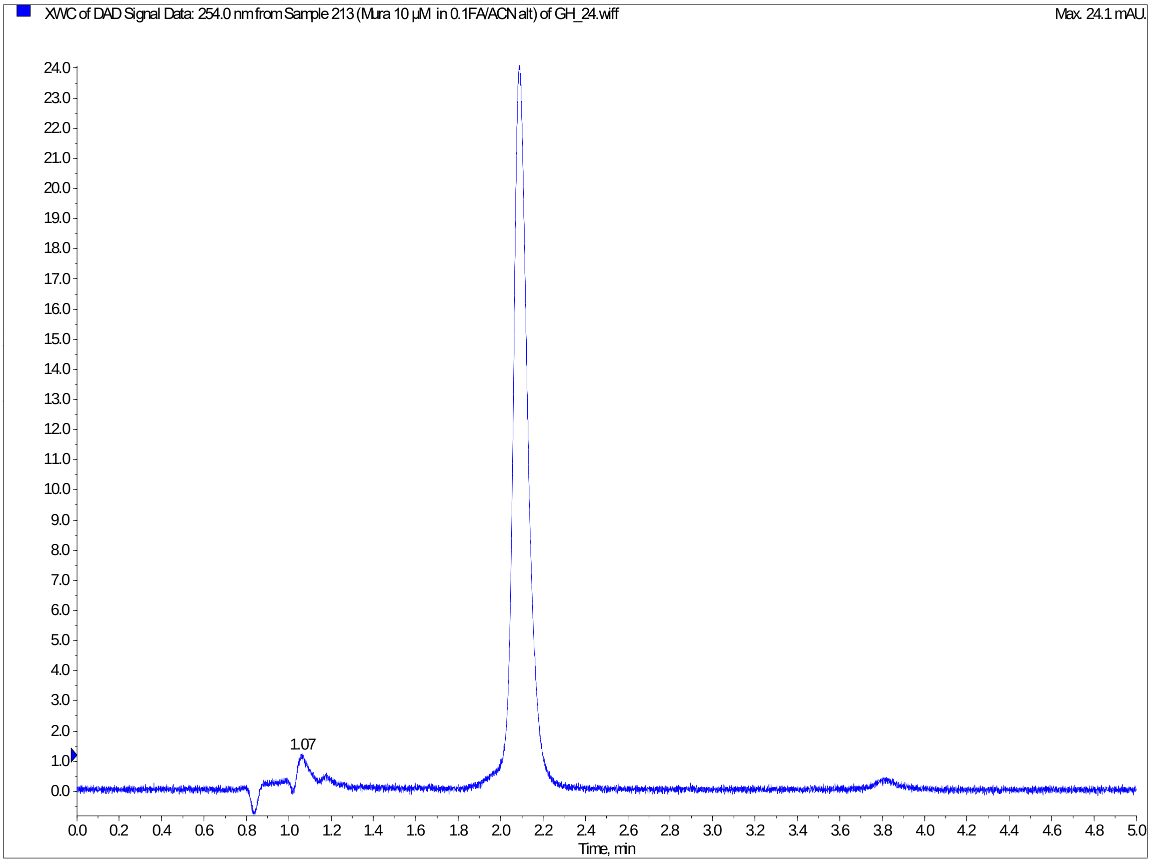

COMPOUND INFORMATION

Biological activity

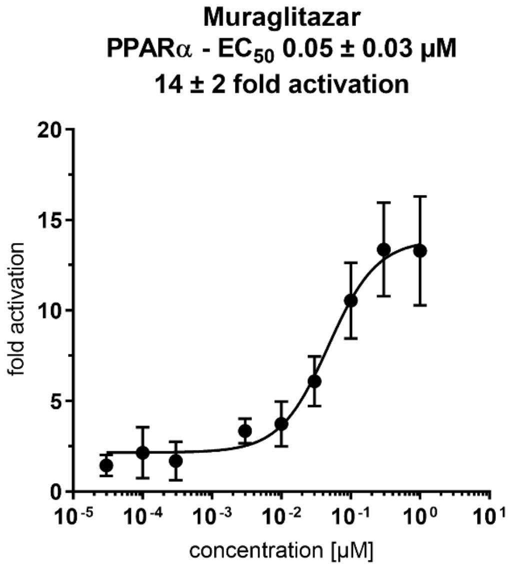

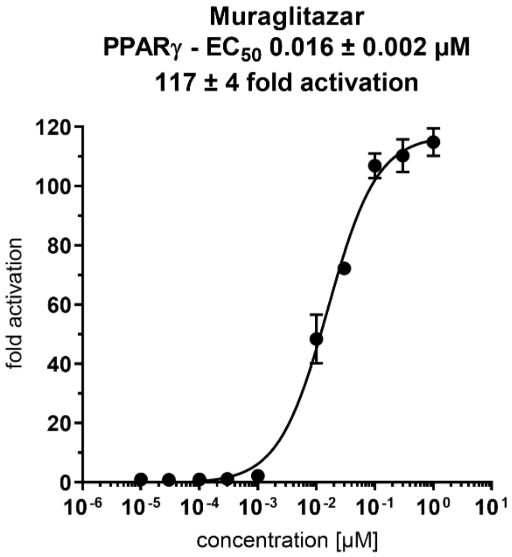

Supplement: Supplementary file 4 — Supplementary Data 1 [file 41467_2024_49493_MOESM4_ESM.zip › Muraglitazar.pdf]
