# Supplementary material for: Chemogenomics for NR1 nuclear hormone receptors
Source: Nat Commun. 2024 Jun 18;15:5201. doi: 10.1038/s41467-024-49493-6 (PMC11189487; doi:10.1038/s41467-024-49493-6)

COMPOUND INFORMATION

Oxatomide

**CAS Registry No.:**	60607-34-3
**Formal Name:**	1-(3-(4-benzhydrylpiperazin-1-yl)propyl)-1,3-dihydro-2H-benzo[d]imidazol-2-one
**EUbOPEN ID:**	EUB0002358a
**Molecular Formula:**	$C_{27}H_{30}N_4O$
**Molecular Weight:**	426.56 g/mol
**Smiles:**	O=c1[nH]c2ccccc2n1CCCN1CCN(C(c2ccccc2)c2ccccc2)CC1
**Recommended concentration:**	10 µM

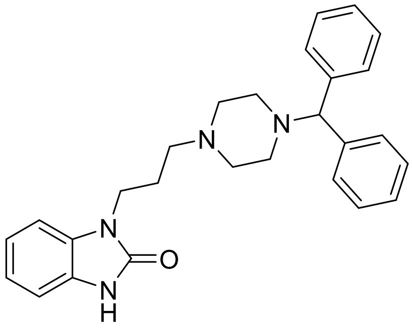

Biological activity

		Type	IC_{50}/EC_{50} [µM]	Reference
Main NR target:	NR1I2 (PXR)	Agonist	6	inhouse
NR off-target:				

COMPOUND INFORMATION

Identity

^{1}H NMR

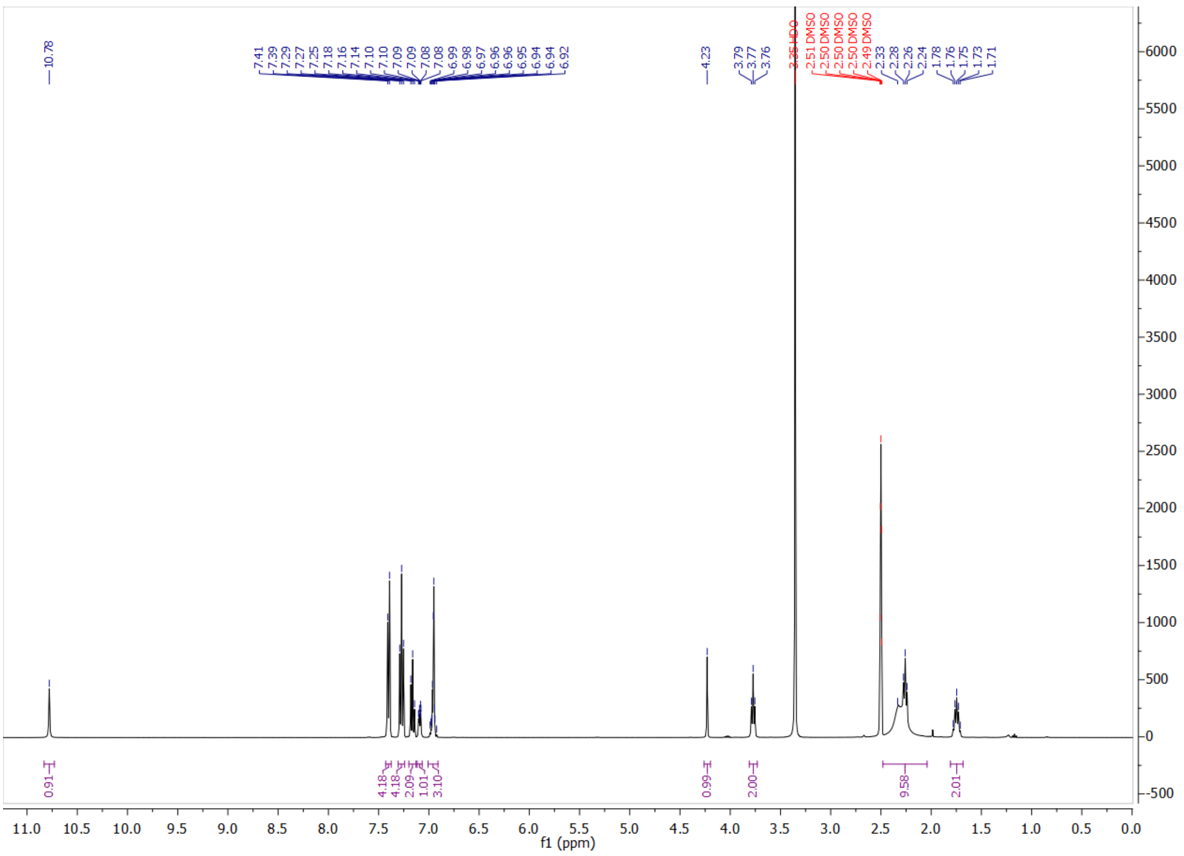

^{13}C NMR

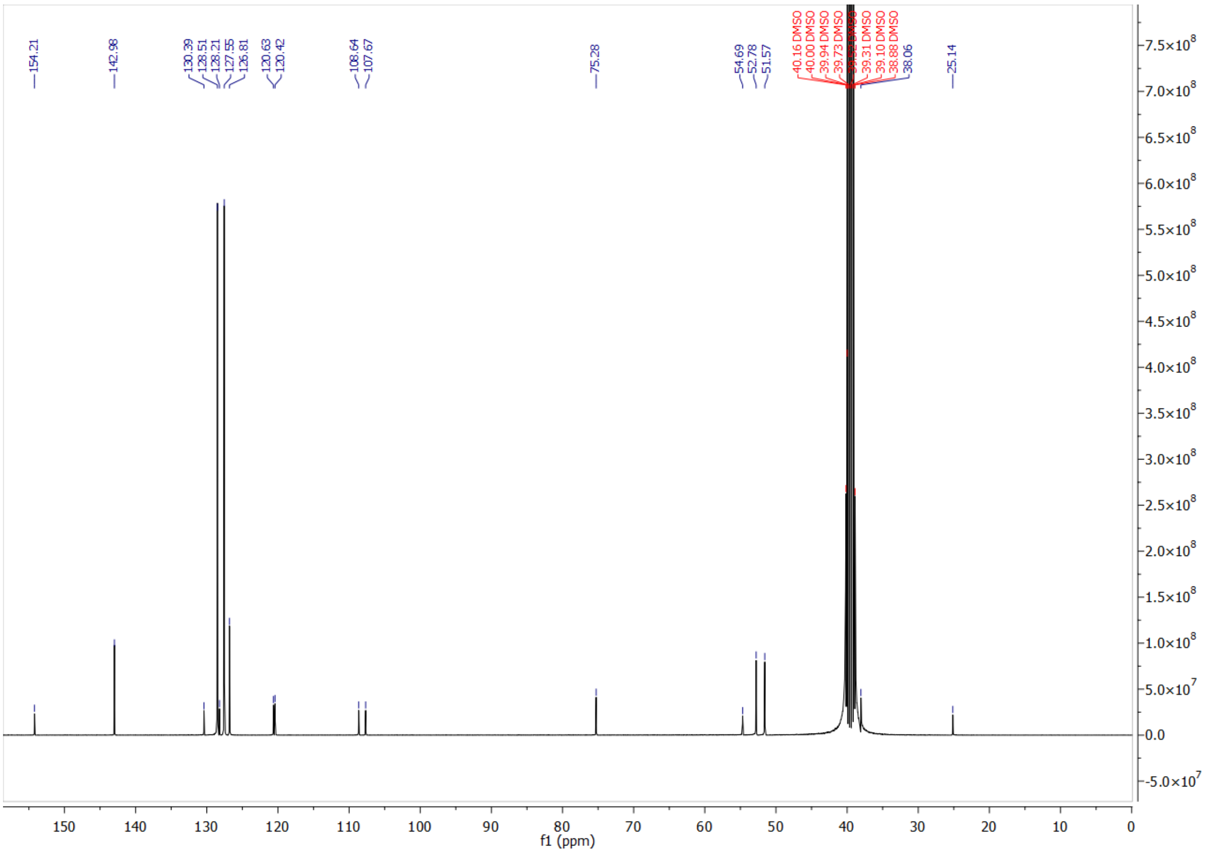

COMPOUND INFORMATION

Purity

**Data file:**	W:\analytical_LCMS_DATA\Sandra\Oxatomide (1) 2024-02-14 16-07-32.D		
**Sample name:**	Oxatomide		
**Description:**			
**Sample amount:**	0.000	**Sample type:**	Sample
		**Location:**	D2F-D3
**Instrument:**	LCMS test	**Injection:**	1 of 1
**Injection date:**	2024-02-14 16:44:24+01:00	**Injection volume:**	8.000
**Acq. method:**	FAST_Nonpolar_General_Method_MS-PosScan-100-1000.M		
**Analysis method:**	SR_03_25_RP-0,5ml_7.5µL_GRAD - 7min_284_280 nm_INJPROG_low.M		

**Module**	**Type**	**Part. No.**	**Serial No.**	**Firmware**
Agilent G6125B MSD	Detector	G6125B	<undef>	3.02.50
Quat. Pump	Pump	G7104C	DEAGZ01778	B.07.35 [0002]
Column Comp.	Column compartment	G7116A	DEAEM06531	D.07.35 [0002]
DAD	Detector	G7117C	DEAEK07274	D.07.35 [0002]
Multisampler	Auto sampler	G7167A	DEAGY01736	D.07.38 [0003]

Results

Sample Name Oxatomide

Chromatograms

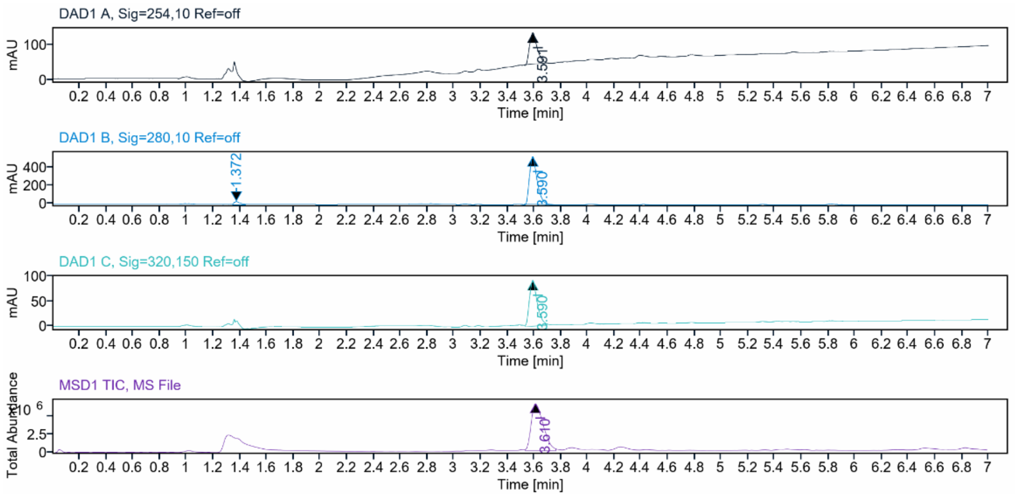

Sample Purity

Signal Description DAD1 A, Sig=254,10 Ref=off

Sample Name	Name	RT	Width	Area	Area%	Height
Oxatomide		3.591	0.061	353.3834	100.00	90.8205

Max Area% 100.000

UV Signal Purity>95% Pass

Signal Description DAD1 B, Sig=280,10 Ref=off

Sample Name	Name	RT	Width	Area	Area%	Height
Oxatomide		1.372	0.049	112.9369	5.26	36.4522
Oxatomide		3.590	0.060	2034.6965	94.74	518.3704

Max Area% 94.741

UV Signal Purity>95% Fail

COMPOUND INFORMATION

Signal Description	DAD1 C, Sig=320,150 Ref=off

Sample Name	Name	RT	Width	Area	Area%	Height
Oxatomide		3.590	0.063	376.0159	100.00	92.4773

Max Area%	100.000
UV Signal Purity>95%	Pass

UV Apex Spectra

RT:

3.591

Sample Name:

Oxatomide

Signal Name:

DAD1A

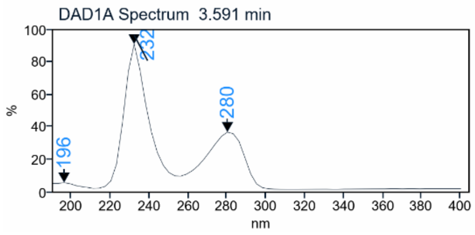

MSD Apex Spectra

RT:

3.610

Sample Name:

Oxatomide

Signal Name:

MSD1TIC

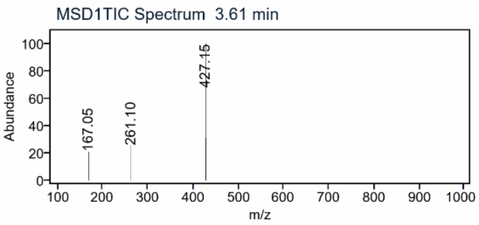

Biological activity

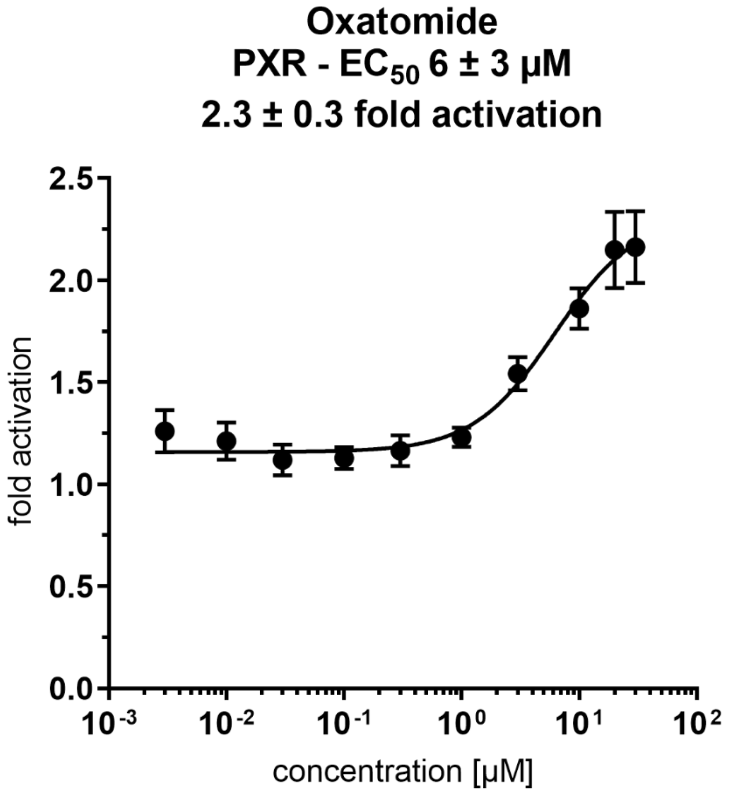

Supplement: Supplementary file 4 — Supplementary Data 1 [file 41467_2024_49493_MOESM4_ESM.zip › Oxatomide.pdf]
