# Supplementary material for: Chemogenomics for NR1 nuclear hormone receptors
Source: Nat Commun. 2024 Jun 18;15:5201. doi: 10.1038/s41467-024-49493-6 (PMC11189487; doi:10.1038/s41467-024-49493-6)

COMPOUND INFORMATION

PF-06747711

**CAS Registry No.:**	1892576-58-7
**Formal Name:**	3-cyano-N-(3-(1-isobutyrylpiperidin-4-yl)-1-methyl-4-(trifluoromethyl)-1H-pyrrolo[2,3-b]pyridin-5-yl)benzamide
**EUBOPEN ID:**	EUB0001170a
**Molecular Formula:**	$C_{26}H_{26}F_3N_5O_2$
**Molecular Weight:**	497.52 g/mol
**Smiles:**	FC(C1=C(NC(C2=CC(C#N)=CC=C2)=O)C=NC3=C1C(C4CCN(C(C(C)C)=O)CC4)=CN3C)(F)F
**Recommended concentration:**	1 µM

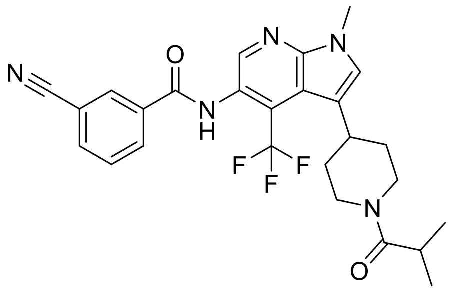

Biological activity

		Type	IC_{50}/EC_{50} [µM]	Reference
Main NR target:	NR1F3 (RORγ)	inv. Agonist	0.004	https://doi.org/10.1021/acs.jmedchem.8b00392
NR off-target:				

COMPOUND INFORMATION

Identity

^{1}H NMR

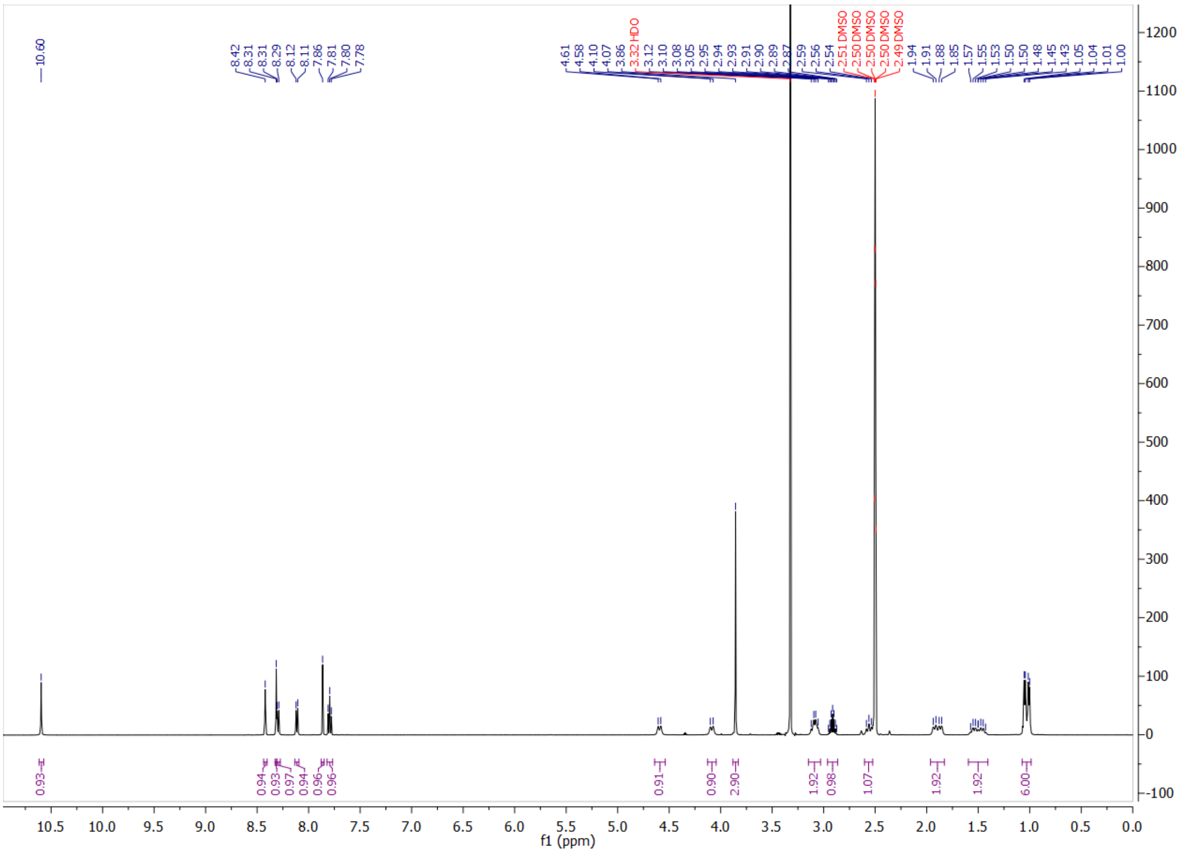

^{13}C NMR

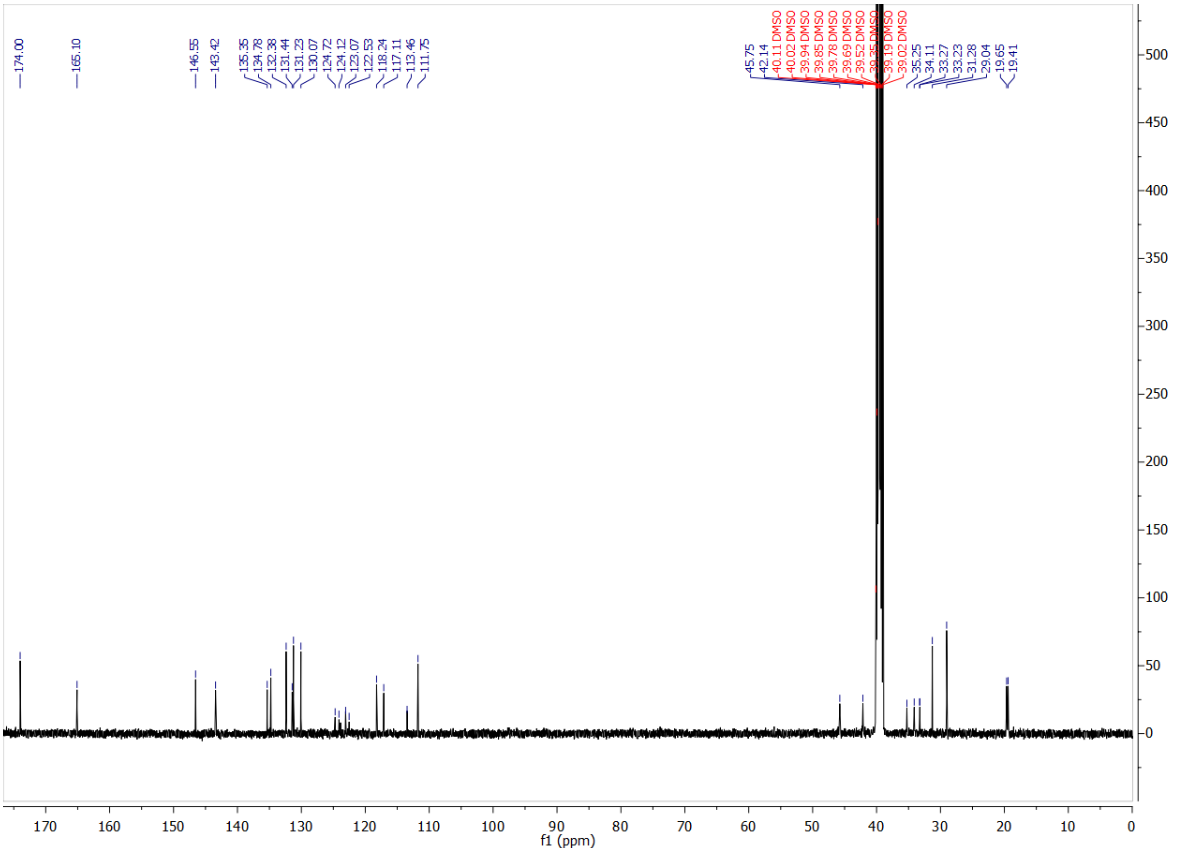

COMPOUND INFORMATION

Purity

Data File W:\analyti...CGC_wave3_1_FirstPassB 2023-01-04 18-28-02\093-D2F-H6-PF-06747711.D
Sample Name: PF-06747711

```
=====================================================================
Acq. Operator   : SYSTEM                          Seq. Line :  93
Sample Operator : SYSTEM
Acq. Instrument : LCMS test                        Location :   D2F-H6
Injection Date  : 1/5/2023 11:28:13 AM                  Inj :   1
                                                 Inj Volume : Inj prog
Sequence File   : W:\analytical_LCMS_DATA\EUbOPEN\CGC_wave3_1_FirstPassB 2023-01-04 18-28-02
                  \CGC_wave3_1_FirstPassB.S
Method          : W:\analytical_LCMS_DATA\EUbOPEN\CGC_wave3_1_FirstPassB 2023-01-04 18-28-02
                  \CGL_FIRSTPASS_GENERALMETHOD_VIAL1+2_20210319.M (Sequence Method)
Last changed    : 1/25/2022 4:36:18 PM by SYSTEM
Method Info     : CGL wellplate, 0.5 uL of 10 mM DMSO, general method
```

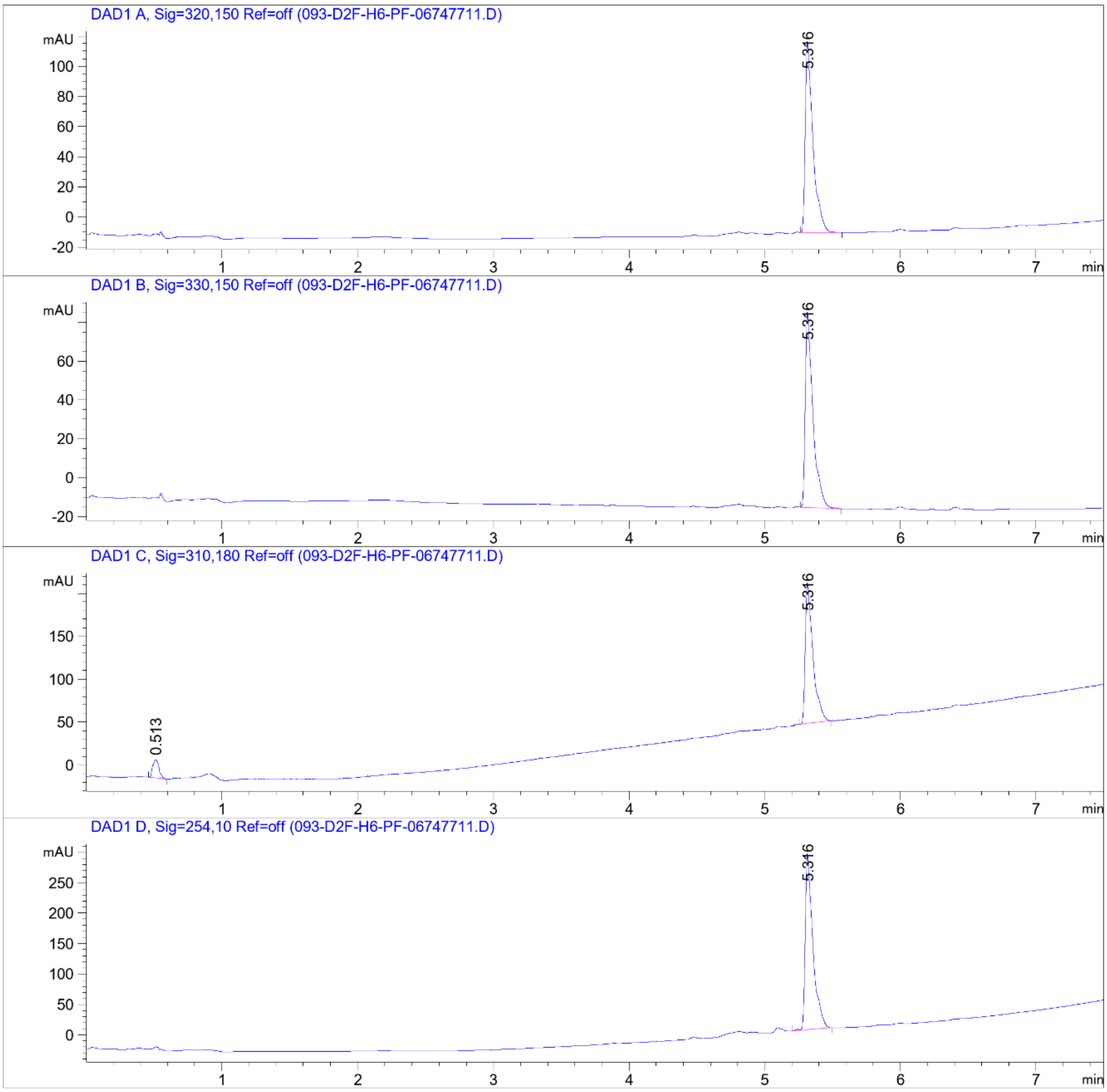

COMPOUND INFORMATION

Data File W:\analyti...CGC_wave3_1_FirstPassB 2023-01-04 18-28-02\093-D2F-H6-PF-06747711.D
Sample Name: PF-06747711

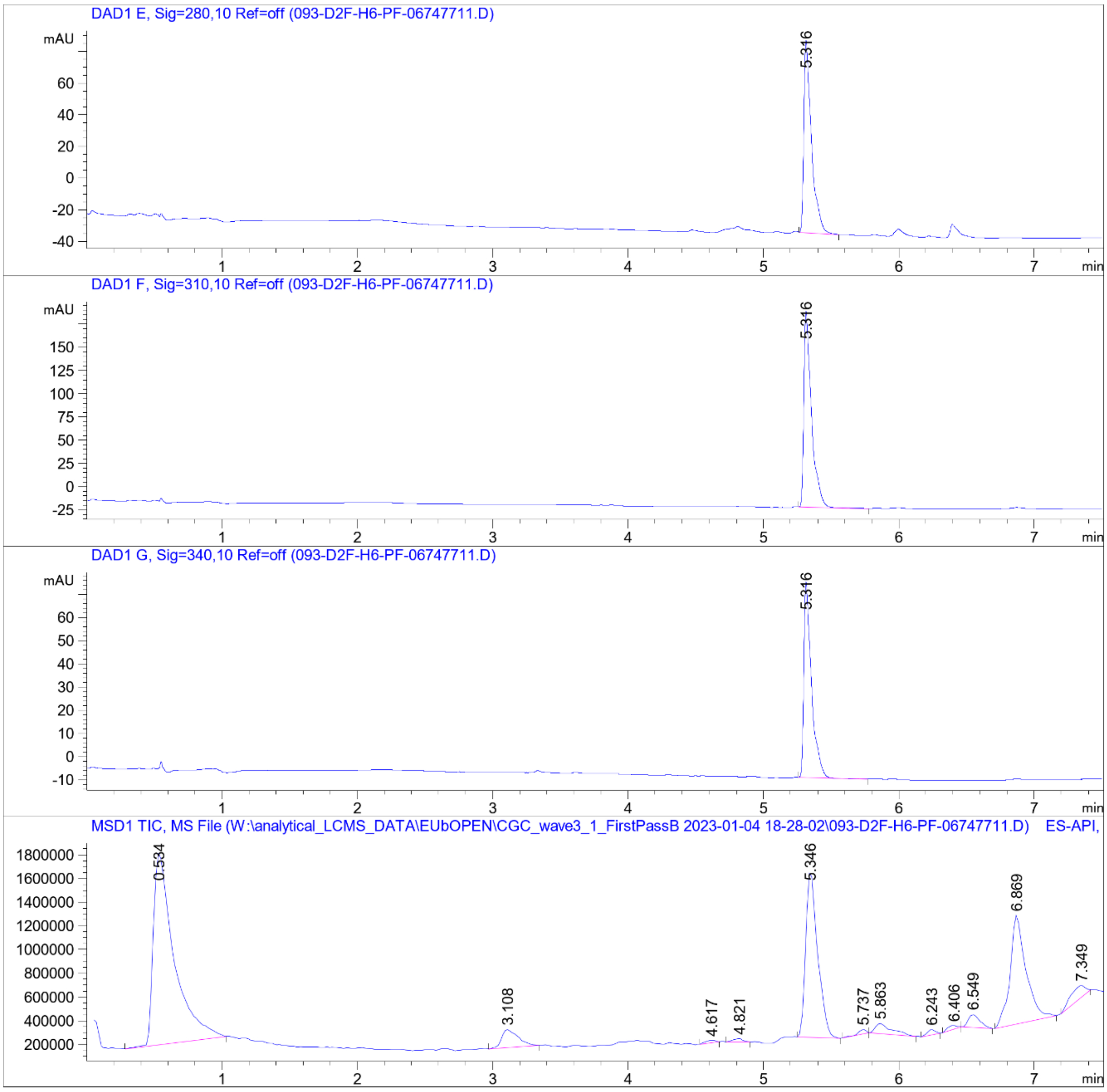

COMPOUND INFORMATION

Data File W:\analyti...CGC_wave3_1_FirstPassB 2023-01-04 18-28-02\093-D2F-H6-PF-06747711.D
Sample Name: PF-06747711

MS Signal: MSD1 TIC, MS File, ES-API, Pos, Scan, Frag: 70, "POS Scan"
Spectra from peak tops.
Noise Cutoff: 1000 counts.
Reportable Ion Abundance: > 50%.
LC Signal: DAD1 A, Sig=320,150 Ref=off
Peak matching window: 0.1 min

Retention Time (LC)	LC Area	Retention Time (MS)	MS Area	Mol. Weight or Ion
-	-	0.534	17254568	157.00 I
-	-	3.108	1309271	239.00 I
				217.10 I
-	-	4.617	104959	170.80 I
				158.20 I
				137.20 I
-	-	4.821	148624	510.40 I
				279.10 I
				170.80 I
				137.10 I
5.316	495	5.346	8638452	498.20 I
-	-	5.737	158727	280.20 I
-	-	5.863	709822	318.20 I
				296.20 I
-	-	6.243	180327	228.20 I
				137.10 I
-	-	6.406	162484	282.30 I
				254.20 I
-	-	6.549	646716	507.20 I
				485.30 I
				280.20 I
-	-	6.869	7524214	282.20 I
-	-	7.349	871242	400.30 I
				282.20 I

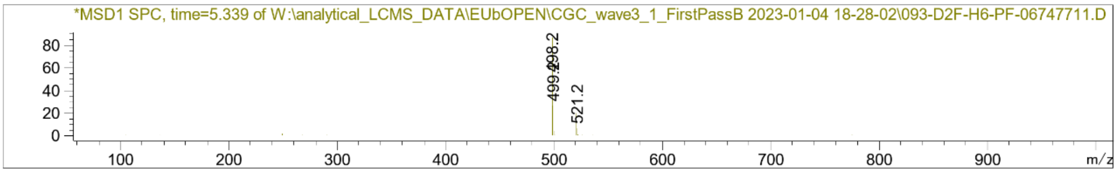

Supplement: Supplementary file 4 — Supplementary Data 1 [file 41467_2024_49493_MOESM4_ESM.zip › PF-06747711.pdf]
