# Supplementary material for: Chemogenomics for NR1 nuclear hormone receptors
Source: Nat Commun. 2024 Jun 18;15:5201. doi: 10.1038/s41467-024-49493-6 (PMC11189487; doi:10.1038/s41467-024-49493-6)

COMPOUND INFORMATION

Resmetirom (MGL-3196)

**CAS Registry No.:**	920509-32-6
**Formal Name:**	2-(3,5-dichloro-4-((5-isopropyl-6-oxo-1,6-dihydropyridazin-3-yl)oxy)phenyl)-3,5-dioxo-2,3,4,5-tetrahydro-1,2,4-triazine-6-carbonitrile
**EUbOPEN ID:**	EUB0001081a
**Molecular Formula:**	$C_{17}H_{12}Cl_2N_6O_4$
**Molecular Weight:**	435.22 g/mol
**Smiles:**	CC(C)C1=CC(=NNC1=O)OC2=C(C=C(C=C2Cl)N3C(=O)NC(=O)C(=N3)C#N)Cl
**Recommended concentration:**	10 µM

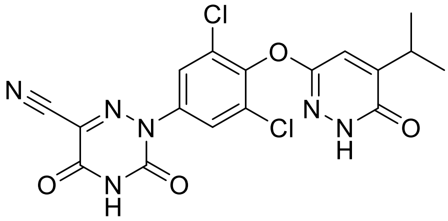

Biological activity

		Type	IC_{50}/EC_{50} [µM]	Reference
Main NR target:	NR1B2 (THRβ)	Agonist	0.2	https://doi.org/10.1021/jm4019299
NR off-target:				

COMPOUND INFORMATION

Identity

¹H NMR

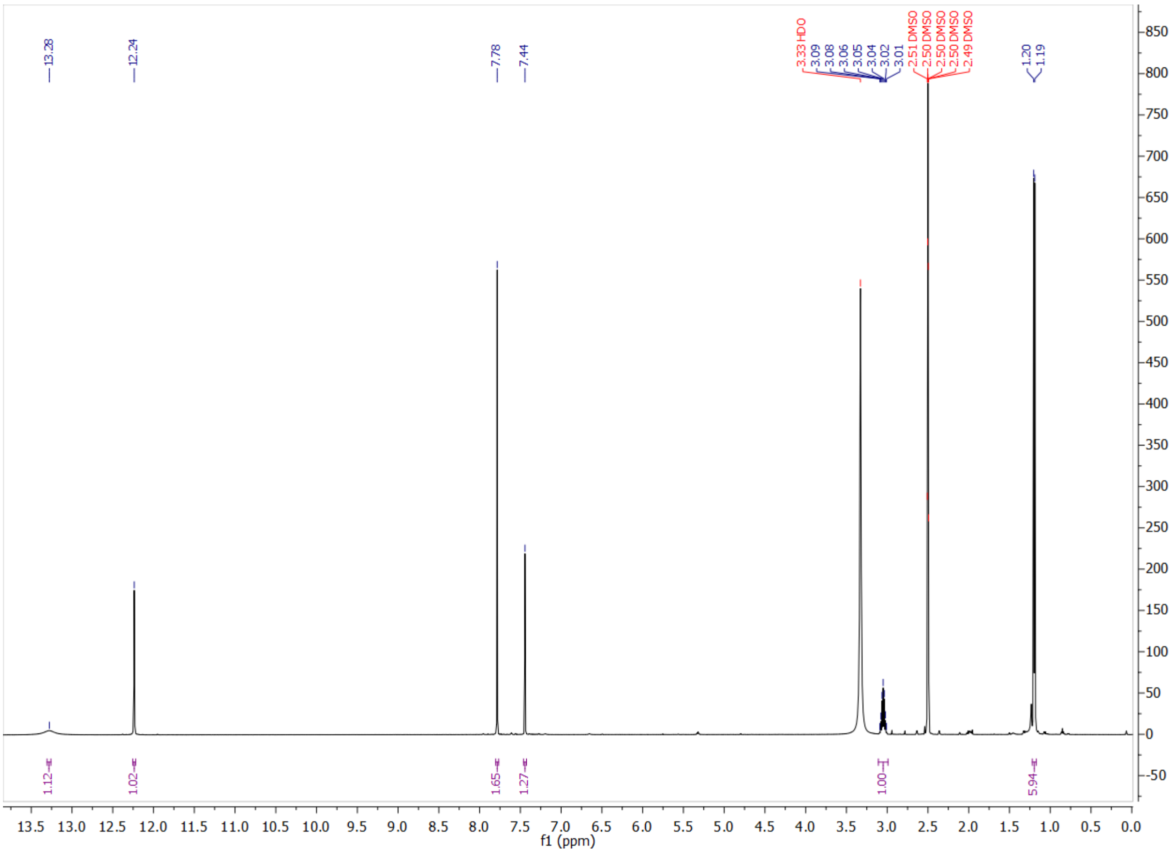

¹³C NMR

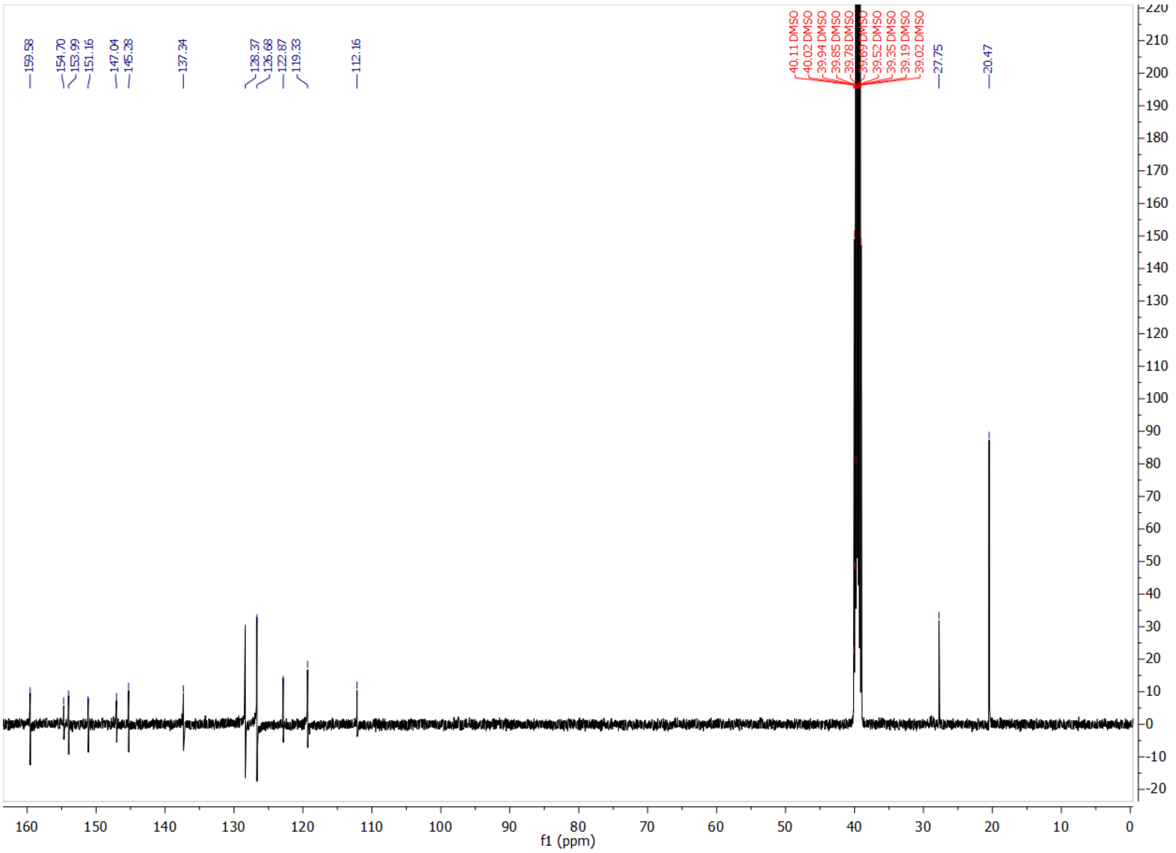

COMPOUND INFORMATION

Purity

```
Data File W:\analyti...irstPassB 2022-02-18 17-58-18\016-D2F-B4-resmetirom (or MGL-3196).D
Sample Name: resmetirom (or MGL-3196)

     =====================================================================
     Acq. Operator   : SYSTEM                         Seq. Line :  16
     Sample Operator : SYSTEM
     Acq. Instrument : LCMS test                       Location :   D2F-B4
     Injection Date  : 2/18/2022 8:47:01 PM                 Inj :   1
                                                 Inj Volume : Inj prog
     Sequence File   : W:\analytical_LCMS_DATA\EUbOPEN\CGC_wave2_4_FirstPassB 2022-02-18 17-58-18
                       \CGC_wave2_4_FirstPassB.S
     Method          : W:\analytical_LCMS_DATA\EUbOPEN\CGC_wave2_4_FirstPassB 2022-02-18 17-58-18
                       \CGL_FIRSTPASS_GENERALMETHOD_VIAL1+2_20210319.M (Sequence Method)
     Last changed    : 1/25/2022 4:36:18 PM by SYSTEM
     Method Info     : CGL wellplate, 0.5 uL of 10 mM DMSO, general method
```

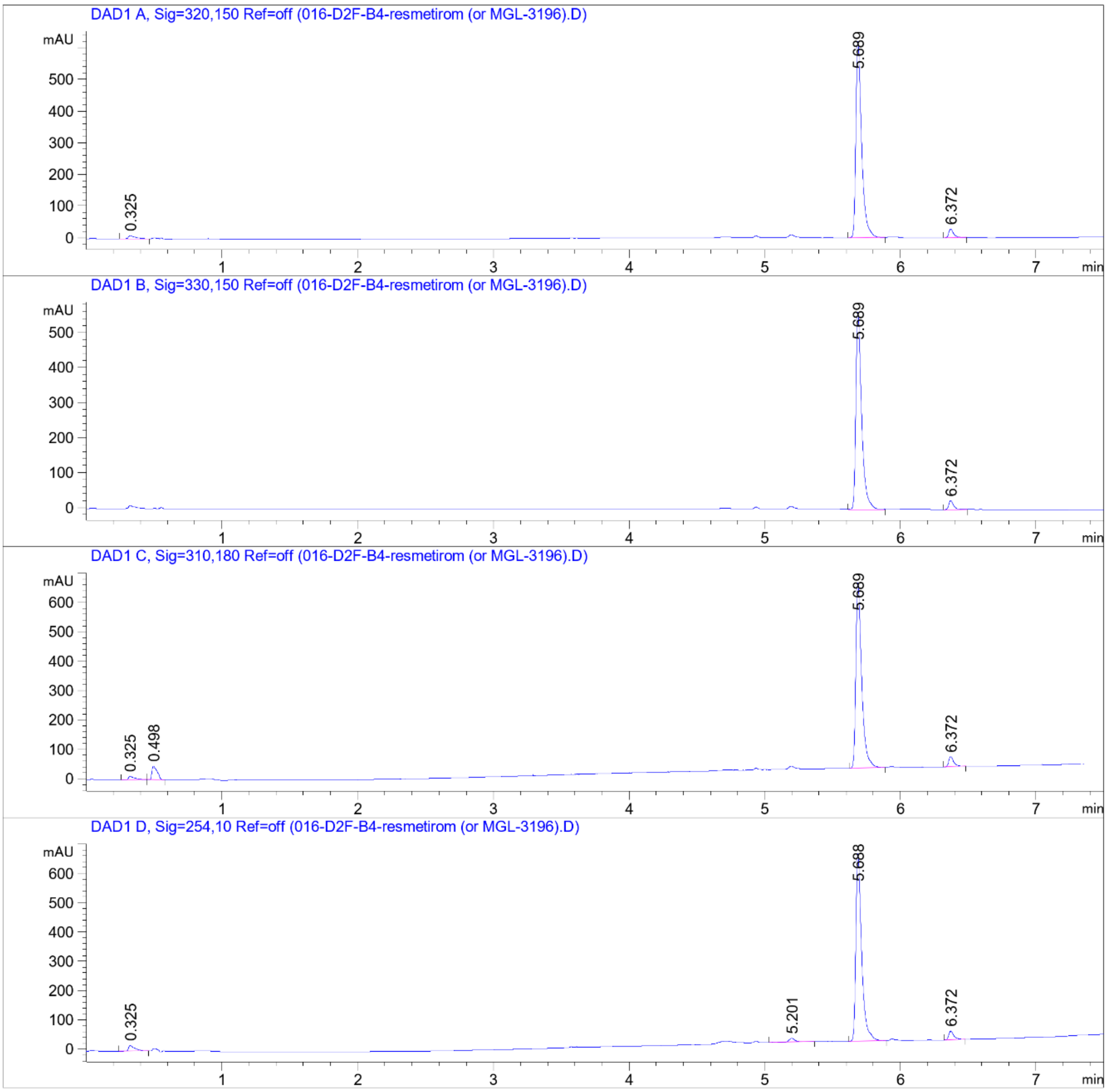

COMPOUND INFORMATION

Data File W:\analyti...irstPassB 2022-02-18 17-58-18\016-D2F-B4-resmetirom (or MGL-3196).D
Sample Name: resmetirom (or MGL-3196)

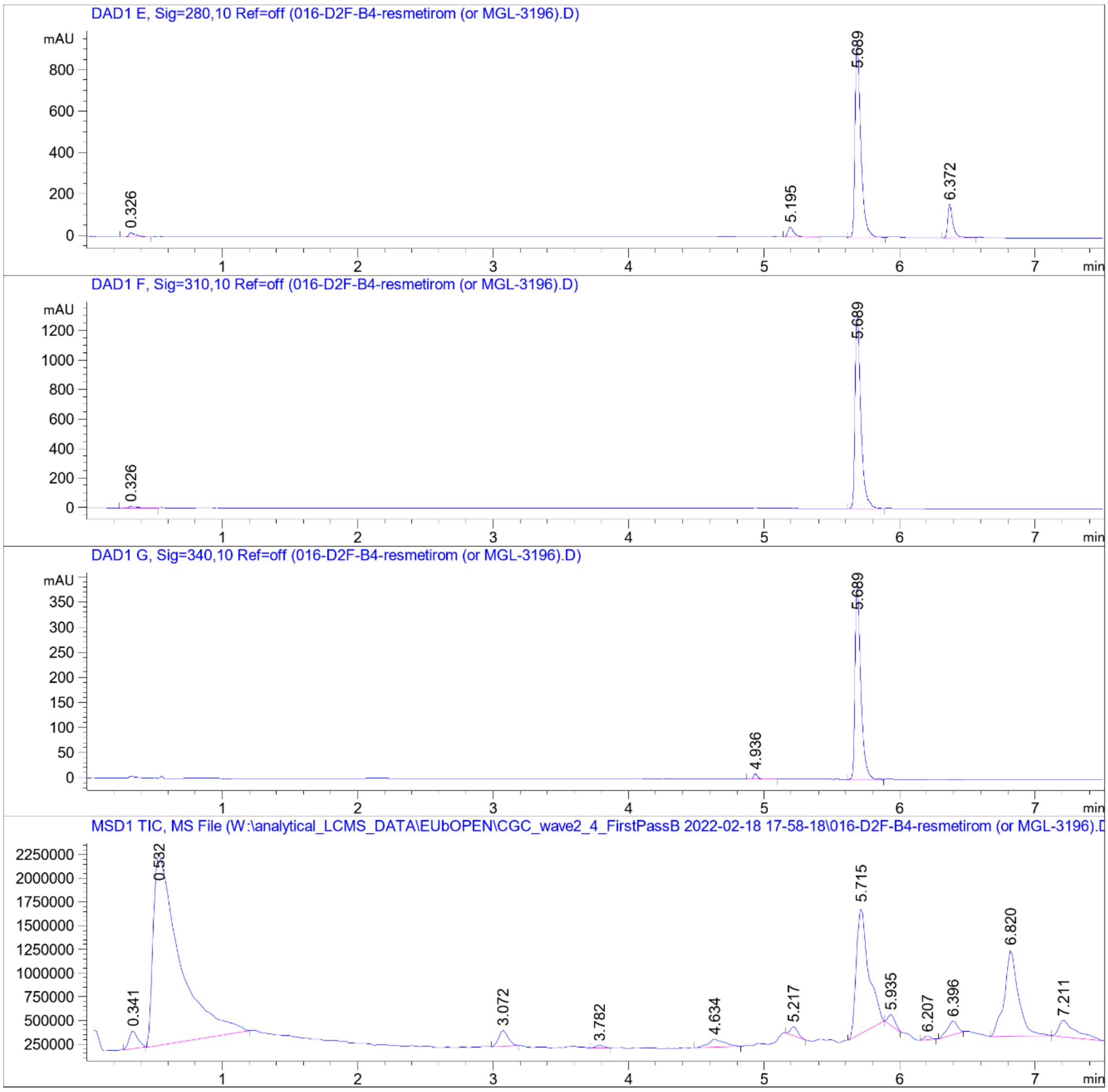

COMPOUND INFORMATION

Data File W:\analyti...irstPassB 2022-02-18 17-58-18\016-D2F-B4-resmetirom (or MGL-3196).D
Sample Name: resmetirom (or MGL-3196)

MS Signal: MSD1 TIC, MS File, ES-API, Pos, Scan, Frag: 70, "POS Scan"
Spectra from peak tops.
Noise Cutoff: 1000 counts.
Reportable Ion Abundance: > 50%.
LC Signal: DAD1 A, Sig=320,150 Ref=off
Peak matching window: 0.1 min

Retention Time (LC)	LC Area	Retention Time (MS)	MS Area	Mol. Weight or Ion
0.325	37	0.341	822951	182.90 I
				158.90 I
				142.00 I
-	-	0.532	27432850	157.00 I
-	-	3.072	806655	239.10 I
				217.10 I
-	-	3.782	129627	274.30 I
-	-	4.634	673506	332.20 I
-	-	5.217	370917	338.20 I
				316.30 I
				298.30 I
5.689	1835	5.715	8266391	437.00 I
				435.00 I
-	-	5.935	415266	318.20 I
				296.20 I
				282.20 I
				280.20 I
-	-	6.207	124596	228.20 I
6.372	71	6.396	713739	350.20 I
				282.30 I
				254.20 I
				243.10 I
-	-	6.820	6471295	282.30 I
-	-	7.211	1498464	284.20 I
				282.30 I

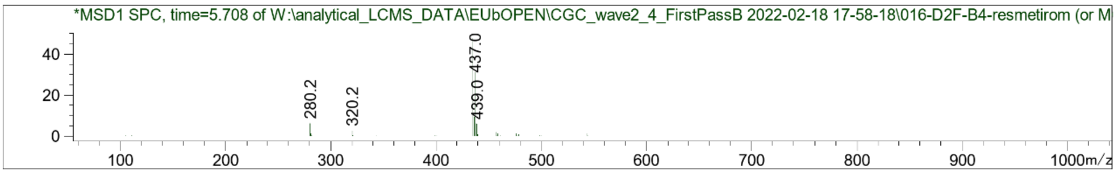

Supplement: Supplementary file 4 — Supplementary Data 1 [file 41467_2024_49493_MOESM4_ESM.zip › Resmetirom.pdf]
