# Supplementary material for: Chemogenomics for NR1 nuclear hormone receptors
Source: Nat Commun. 2024 Jun 18;15:5201. doi: 10.1038/s41467-024-49493-6 (PMC11189487; doi:10.1038/s41467-024-49493-6)

COMPOUND INFORMATION

Retinoic acid (Tretinoin)

**CAS Registry No.:**	302-79-4
**Formal Name:**	(2E,4E,6E,8E)-3,7-dimethyl-9-(2,6,6-trimethylcyclohex-1-en-1-yl)nona-2,4,6,8-tetraenoic acid
**EUbOPEN ID:**	EUB0001136a
**Molecular Formula:**	$C_{20}H_{28}O_2$
**Molecular Weight:**	300.44 g/mol
**Smiles:**	CC1=C(C(CCC1)(C)C)/C=C/C(=C/C=C/C(=C/C(=O)O)/C)/C
**Recommended concentration:**	1 µM

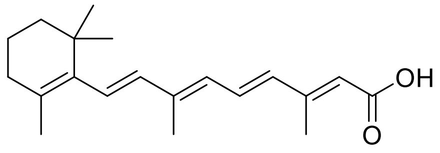

Biological activity

		Type	IC_{50}/EC_{50} [µM]	Reference
Main NR target:	NR1B1 (RARα)	Agonist	0.4	inhouse
	NR1B2 (RARβ)	Agonist	0.36	
	NR1B3 (RARγ)	Agonist	0.34	
NR off-target:	NR1F1 (RORα)	Inverse agonist	0.2	https://doi.org/10.1016/j.bmcl.2014.09.053
	NR1F2 (RORβ)	Inverse agonist	0.1	
	NR1F3 (RORγ)	Inverse agonist	0.2	
	NR2B1 (RXRα)	Agonist	41-fold act.	inhouse

COMPOUND INFORMATION

Identity

¹H NMR

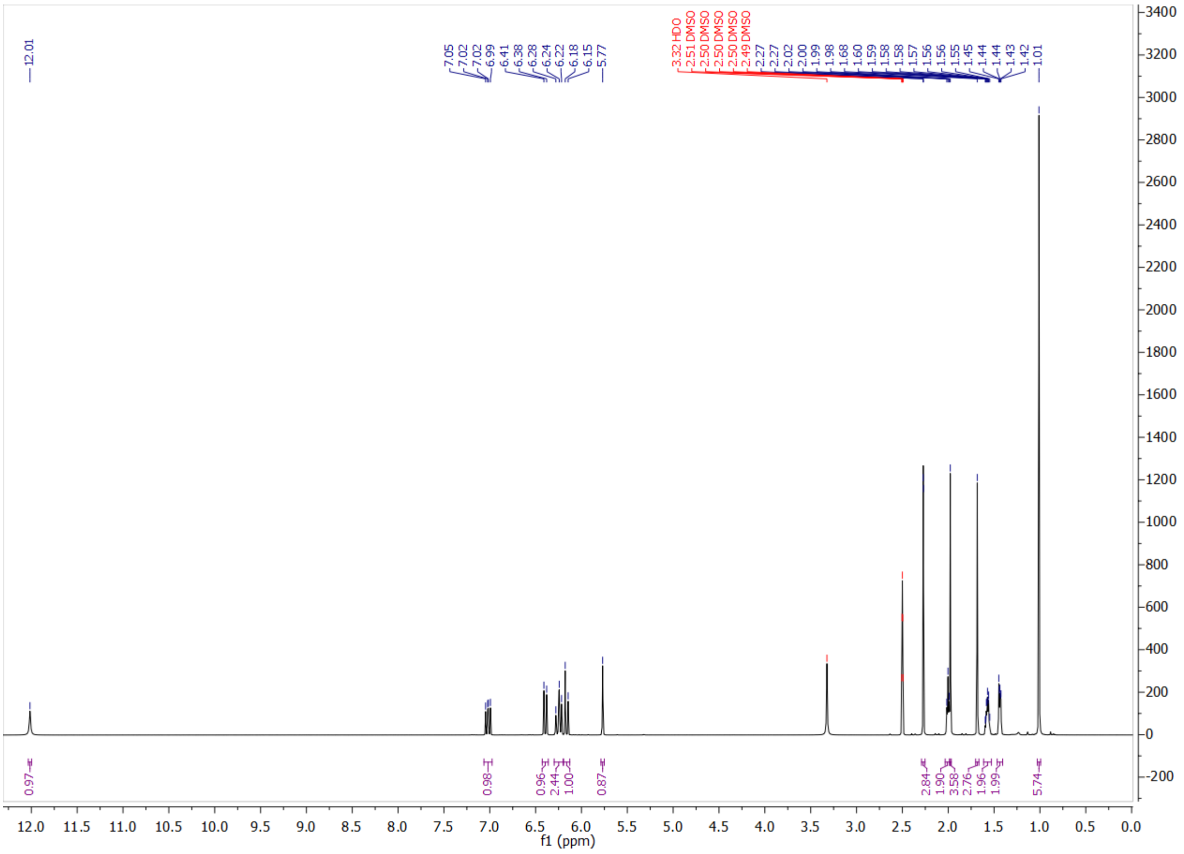

¹³C NMR

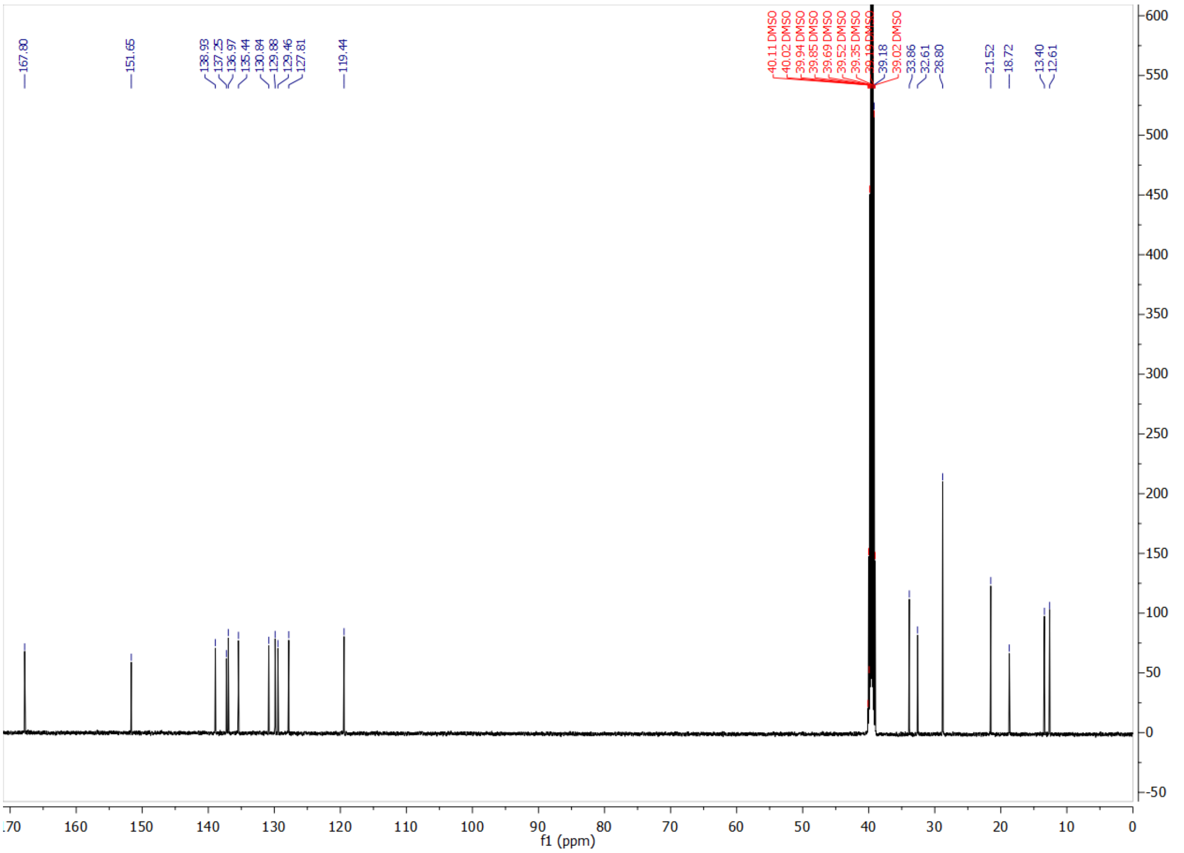

COMPOUND INFORMATION

Purity

M_r 300.44

MS: ESI-positive, *m/z* 301/123

LC: 0.1% HCOOH/ACN (30/70)

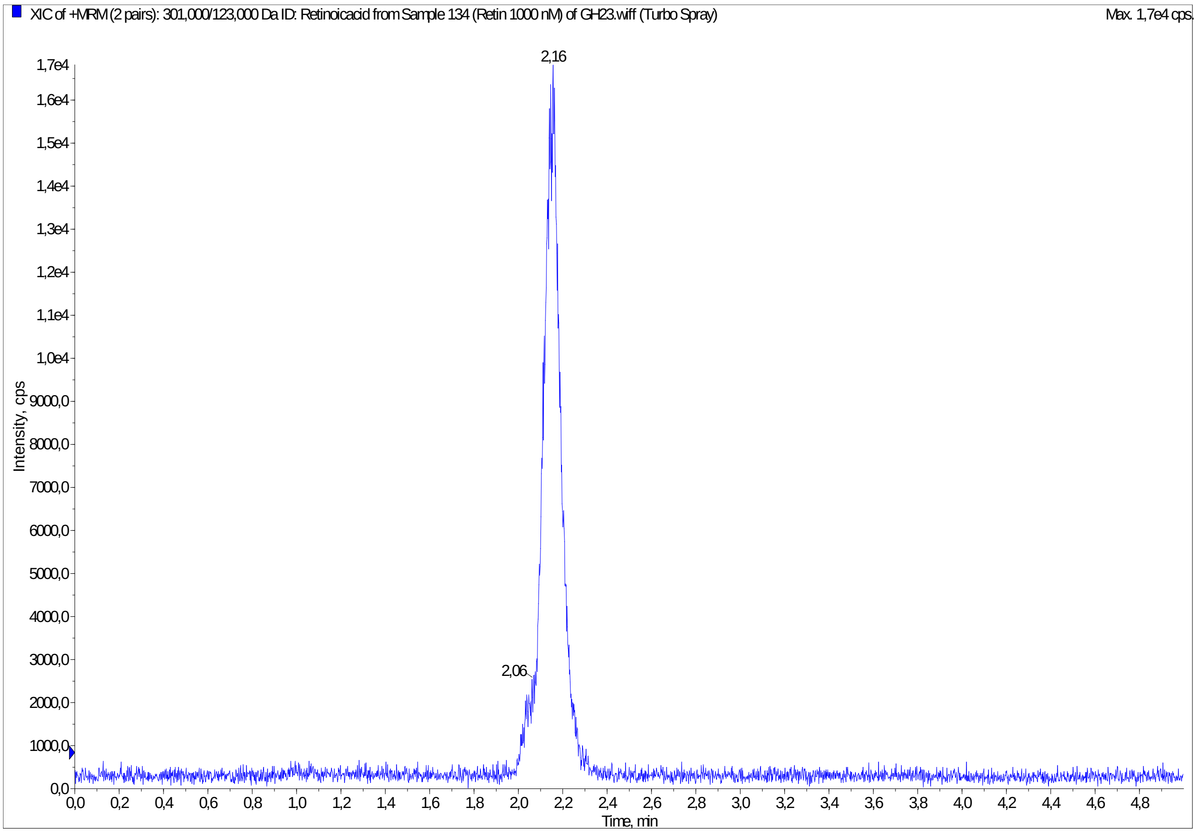

COMPOUND INFORMATION

LC-UV

LC: 0.1% HCOOH/ACN (30/70)

DAD: 230, 250, 238, 270, 325 (XWC) nm

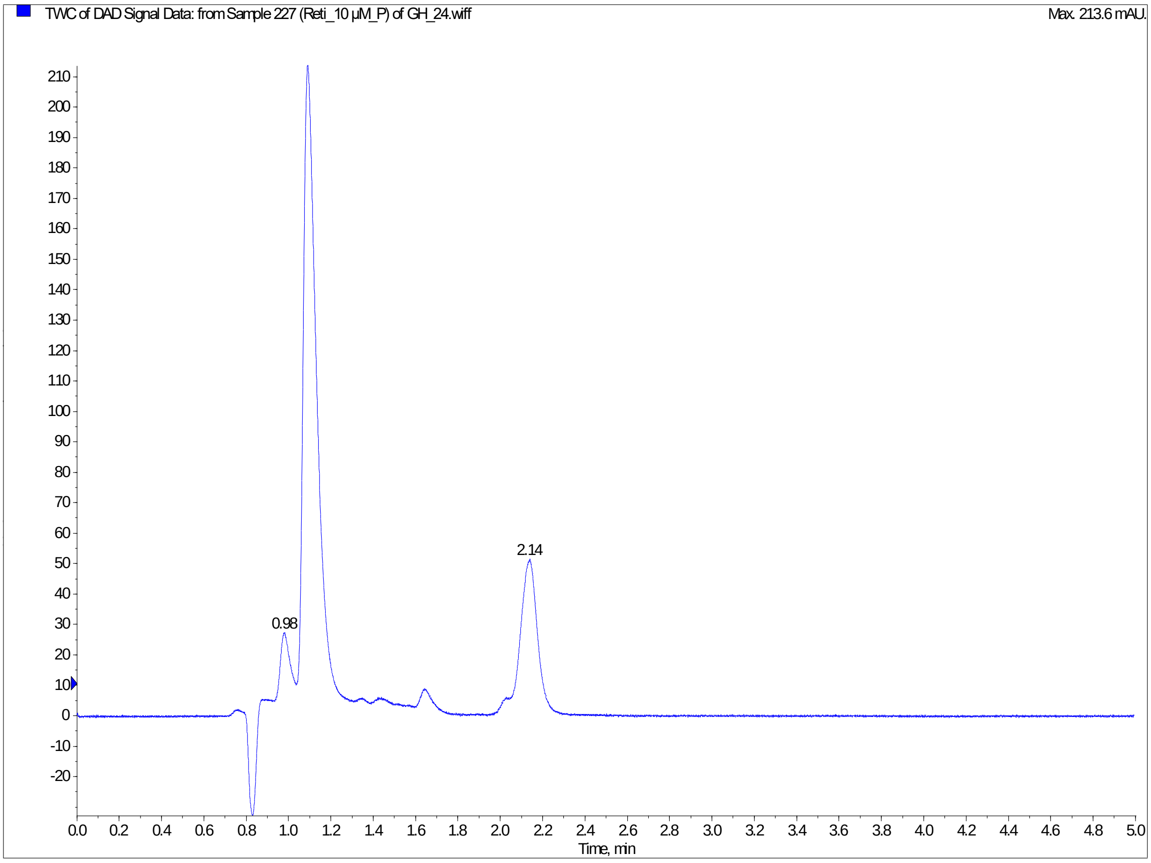

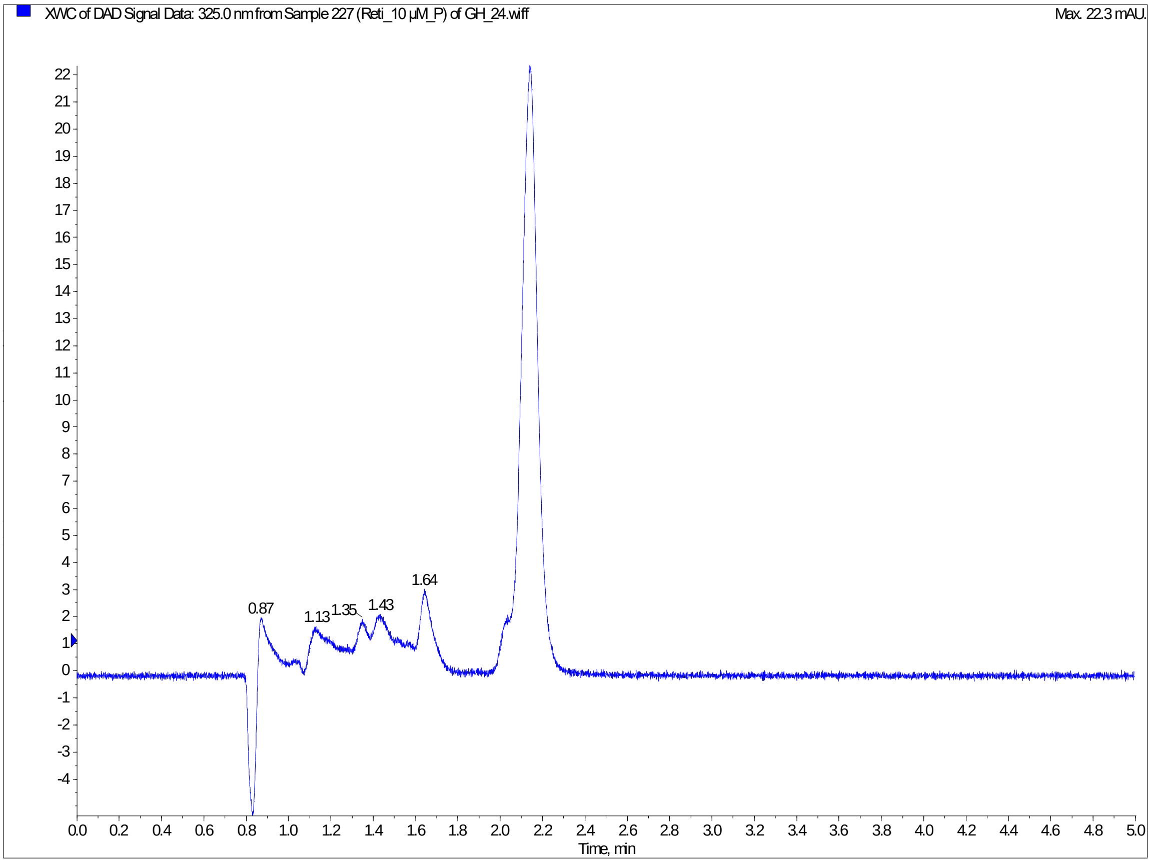

COMPOUND INFORMATION

Biological activity

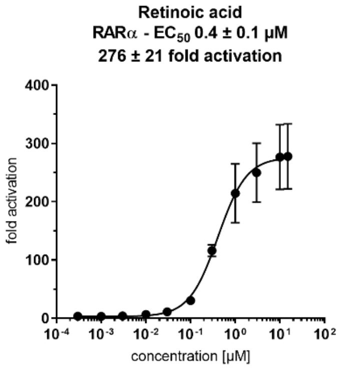

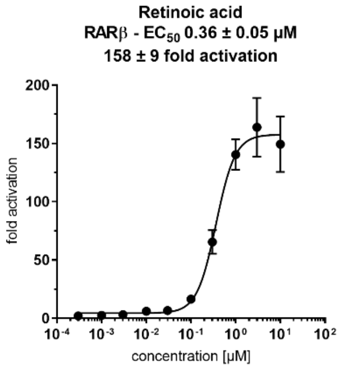

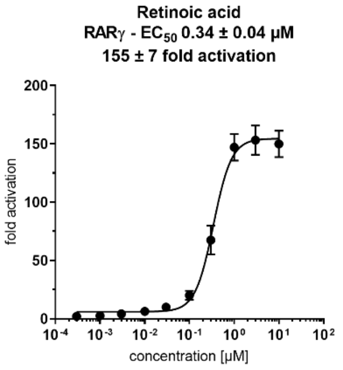

Supplement: Supplementary file 4 — Supplementary Data 1 [file 41467_2024_49493_MOESM4_ESM.zip › Retinoic acid.pdf]
