# Supplementary material for: Chemogenomics for NR1 nuclear hormone receptors
Source: Nat Commun. 2024 Jun 18;15:5201. doi: 10.1038/s41467-024-49493-6 (PMC11189487; doi:10.1038/s41467-024-49493-6)

COMPOUND INFORMATION

RGX-104 (hydrochloride)

**CAS Registry No.:**	610318-03-1
**Formal Name:**	2-(3-(3-((2-chloro-3-(trifluoromethyl)benzyl)(2,2-diphenylethyl)amino)butoxy)phenyl) acetic acid hydrochloride
**EUBOPEN ID:**	EUB0001482aCl
**Molecular Formula:**	$C_{34}H_{34}Cl_2F_3NO_3$
**Molecular Weight:**	632.55 g/mol
**Smiles:**	CC(N(CC(C1=CC=CC=C1)C2=CC=CC=C2)CC3=C(Cl)C(C(F)(F)F)=CC=C3)CCOC4=CC=CC(CC(O)=O)=C4.Cl
**Recommended concentration:**	10 µM

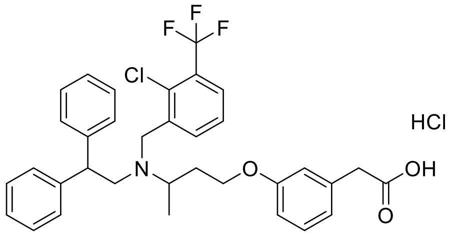

Biological activity

		Type	IC_{50}/EC_{50} [µM]	Reference
Main NR target:	NR1H3 (LXRα)	Agonist	3.3	inhouse
	NR1H2 (LXRβ)	Agonist	2.9	inhouse
NR off-target:				

COMPOUND INFORMATION

Identity

^{1}H NMR

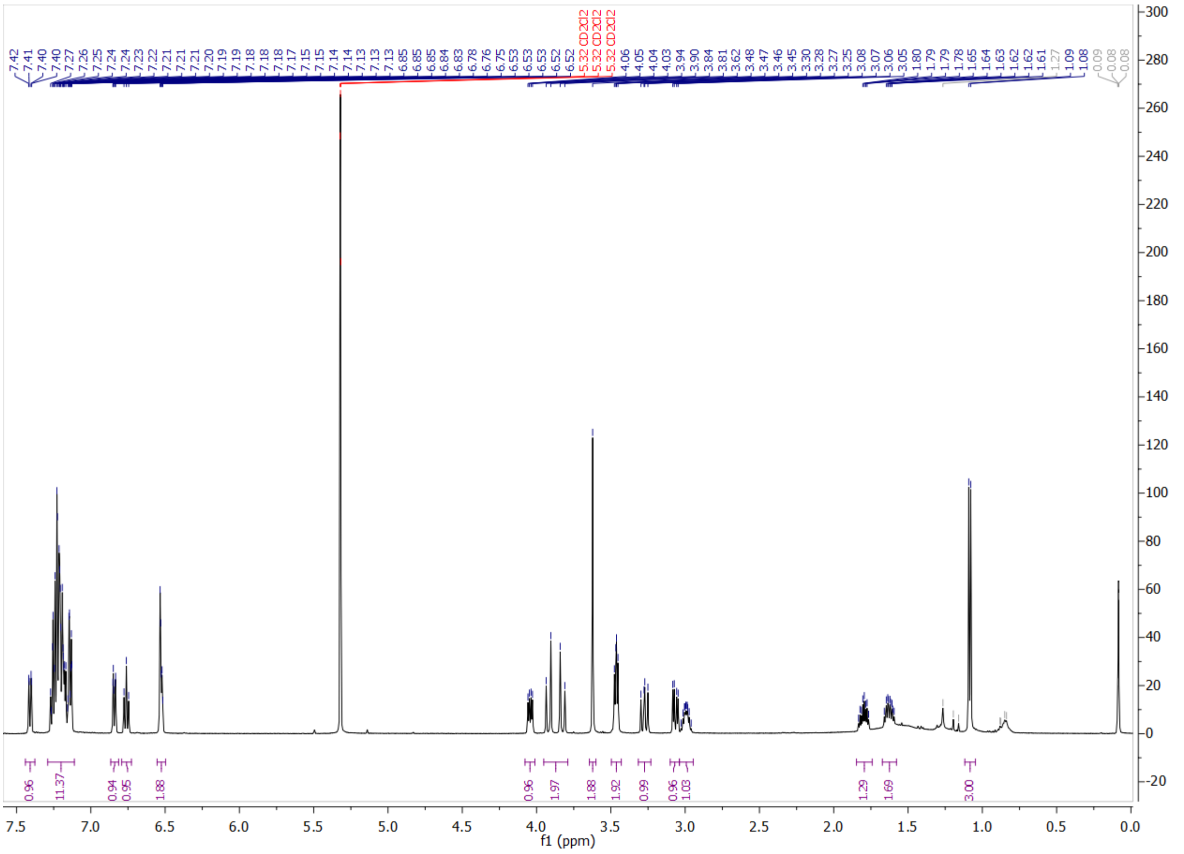

^{13}C NMR

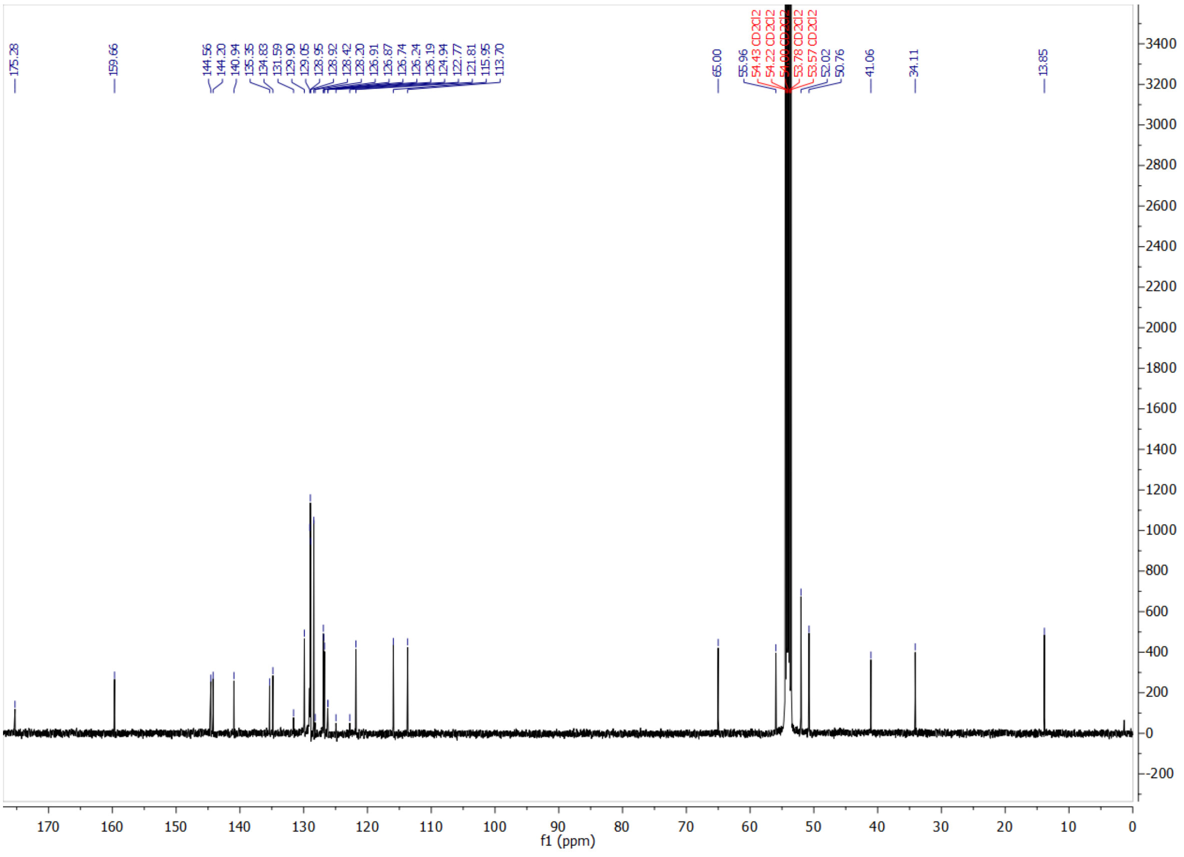

COMPOUND INFORMATION

Purity

LCMS

*M*r 596.08

MS: ESI-negative, m/z 594/179 (blue), m/z 594/141 (red)

LC: 0.1% HCOOH/ACN (30/70)

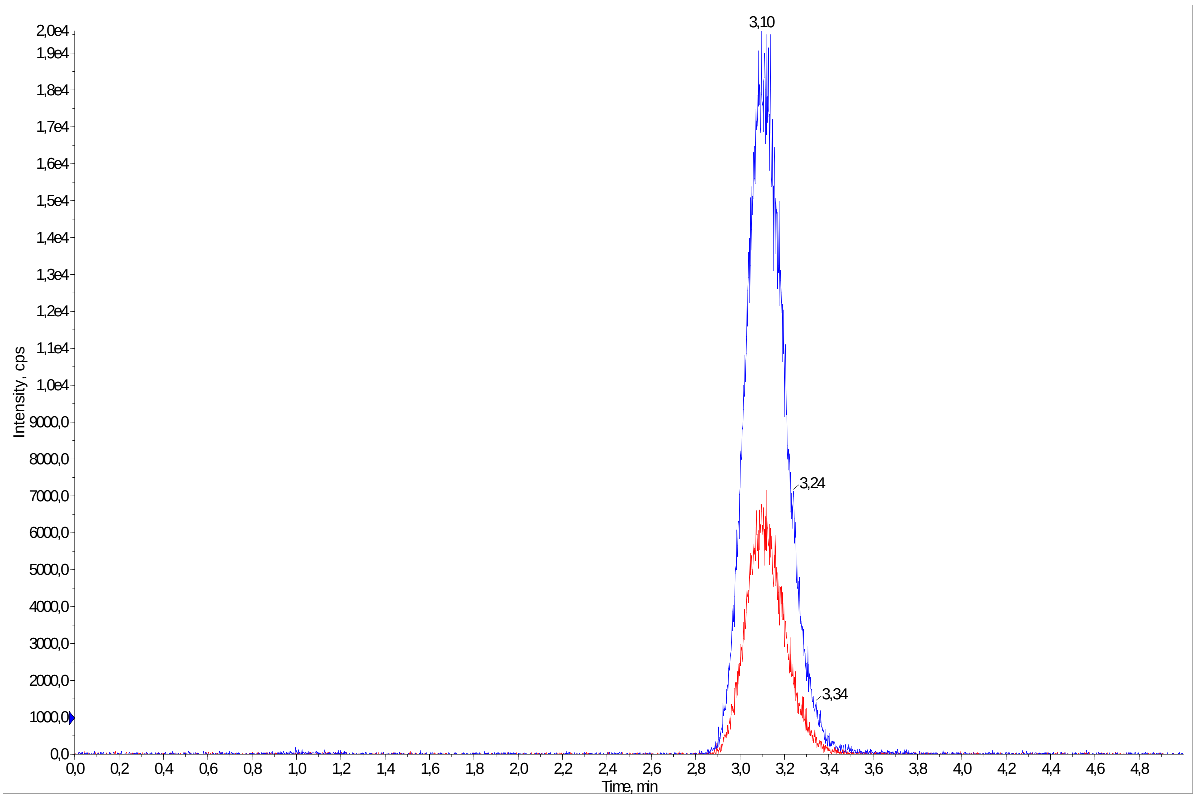

COMPOUND INFORMATION

LC-UV

LC: 0.1% HCOOH/ACN (15/85)

DAD: 230, 254, 275 (XWC), 290, 300 nm

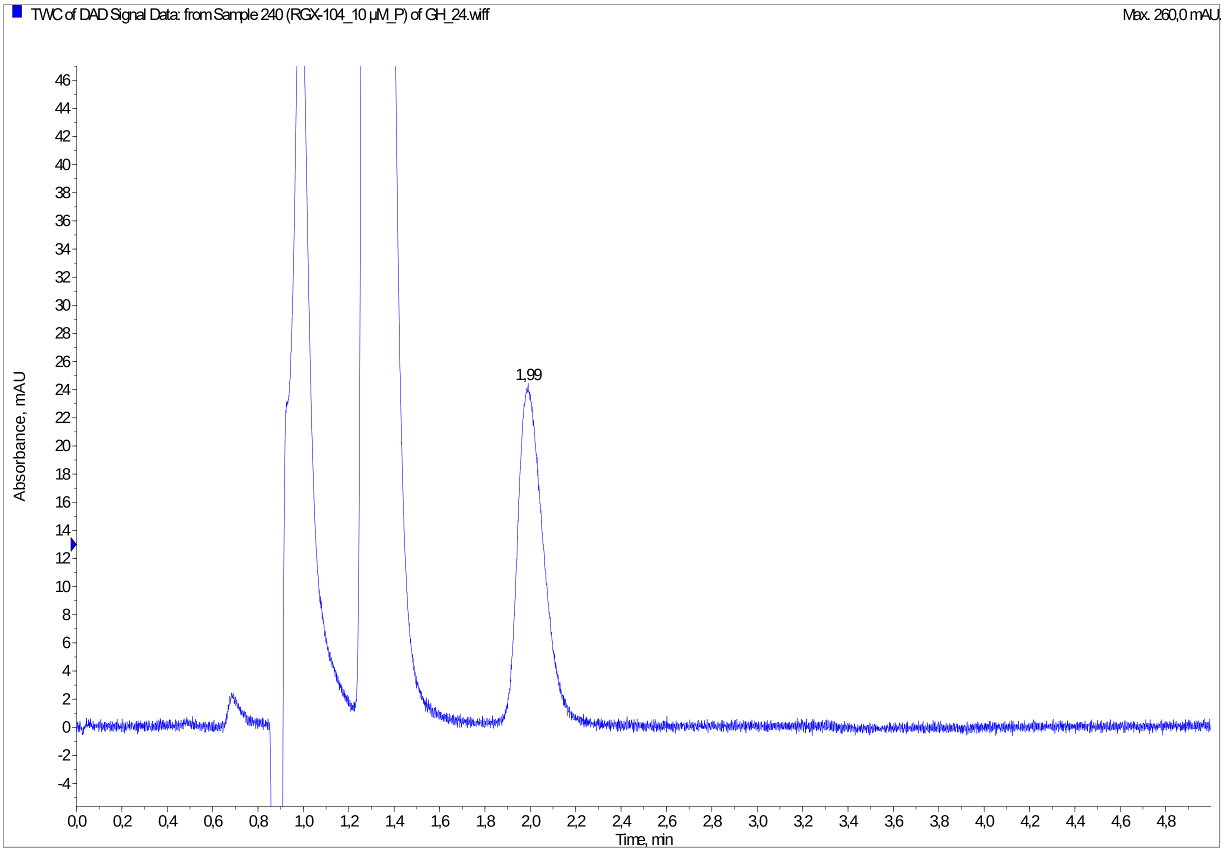

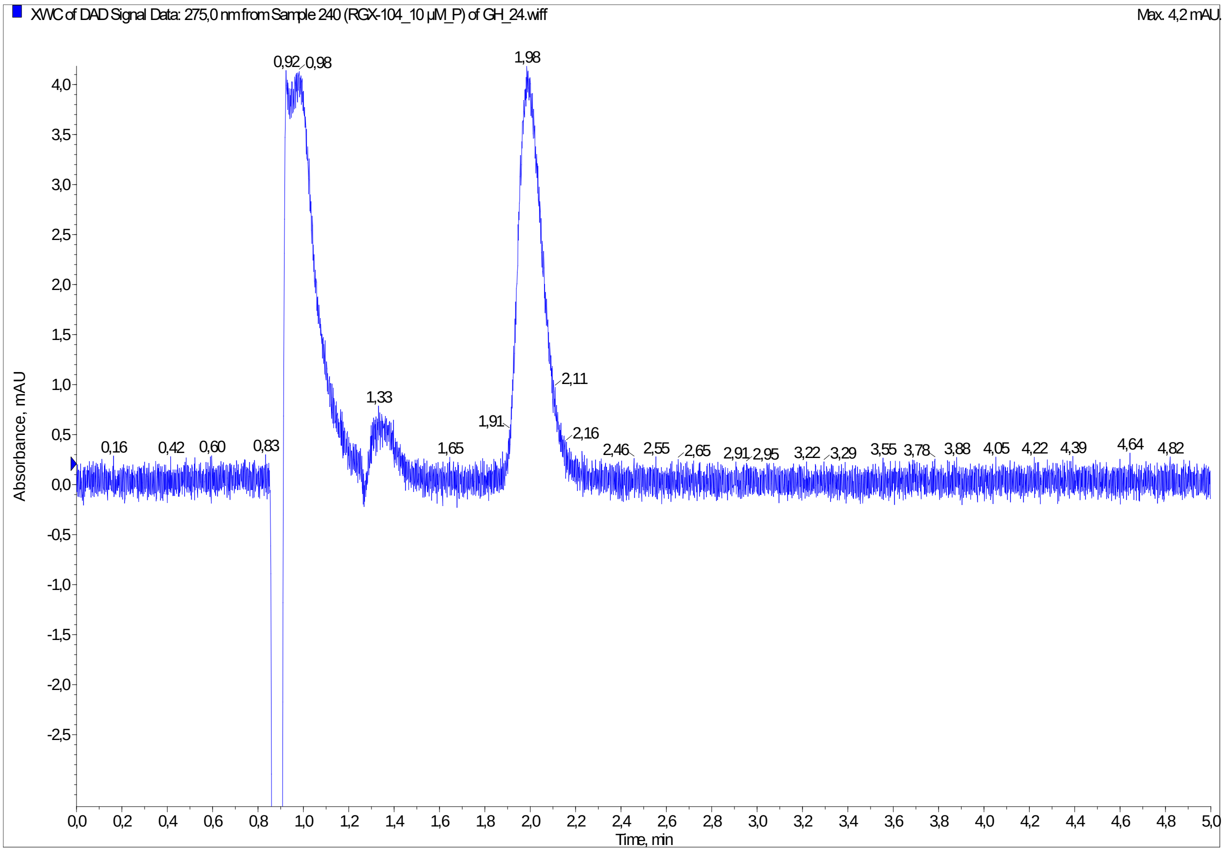

COMPOUND INFORMATION

Biological activity

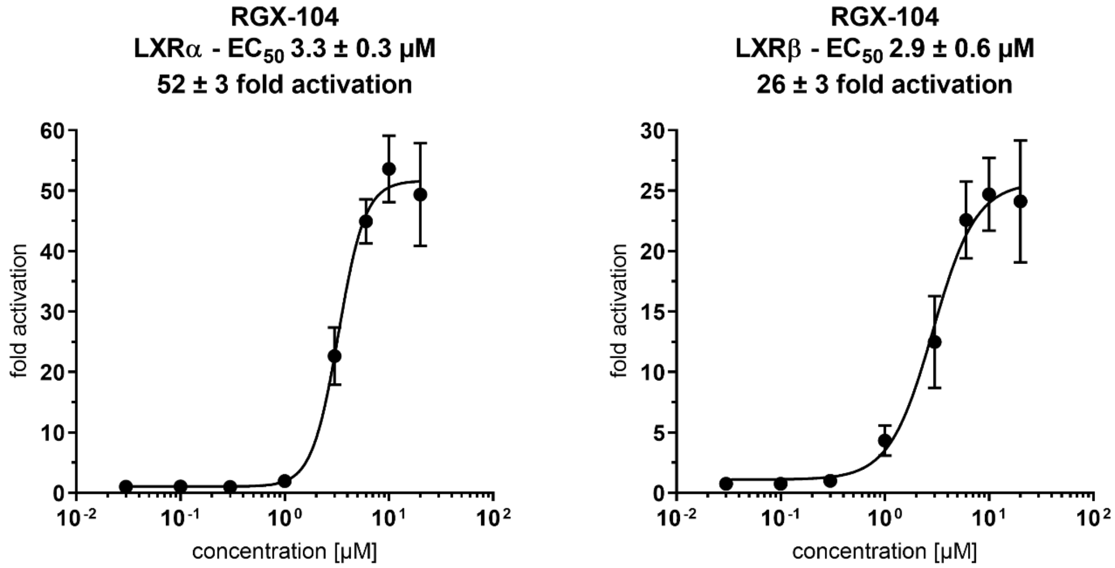

Supplement: Supplementary file 4 — Supplementary Data 1 [file 41467_2024_49493_MOESM4_ESM.zip › RGX-104.pdf]
