# Supplementary material for: Chemogenomics for NR1 nuclear hormone receptors
Source: Nat Commun. 2024 Jun 18;15:5201. doi: 10.1038/s41467-024-49493-6 (PMC11189487; doi:10.1038/s41467-024-49493-6)

COMPOUND INFORMATION

Rifampicin

CAS Registry No.: 13292-46-1

Formal Name: [2,15,17,27,29-pentahydroxy-11-methoxy-3,7,12,14,16,18,22-heptamethyl-26-[(E)-(4-methylpiperazin-1-yl)iminomethyl]-6,23-dioxo-8,30-dioxa-24-azatetracyclo[23.3.1.14,7.05,28]triaconta-1(29),2,4,9,19,21,25,27-octaen-13-yl] acetate

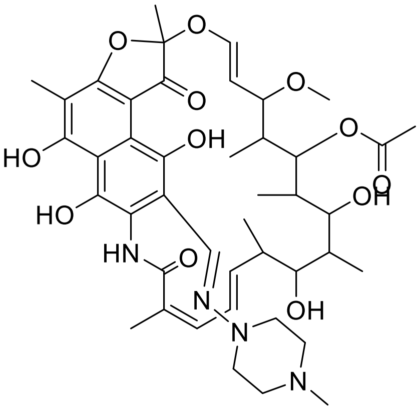

EUBOPEN ID: EUB0000574a

Molecular Formula: $C_{43}H_{58}N_4O_{12}$

Molecular Weight: 822.95 g/mol

Smiles: CC1/C=C/C=C(C)\C(NC2=C(/C=N/N3CCN(C)CC3)C(O)=C4C(C(O)=C(C)C5=C4C(C(O/C=C/C(OC)C(C)C(OC(C)=O)C(C)C(O)C(C)C1O)(C)O5)=O)=C2O)=O

Recommended concentration: 10 µM

Biological activity

		Type	IC_{50}/EC_{50} [µM]	Reference
Main NR target:	NR1I2 (PXR)	Agonist	1	https://doi.org/10.1016/j.bmcl.2016.02.031
NR off-target:				

COMPOUND INFORMATION

Identity

^{1}H NMR

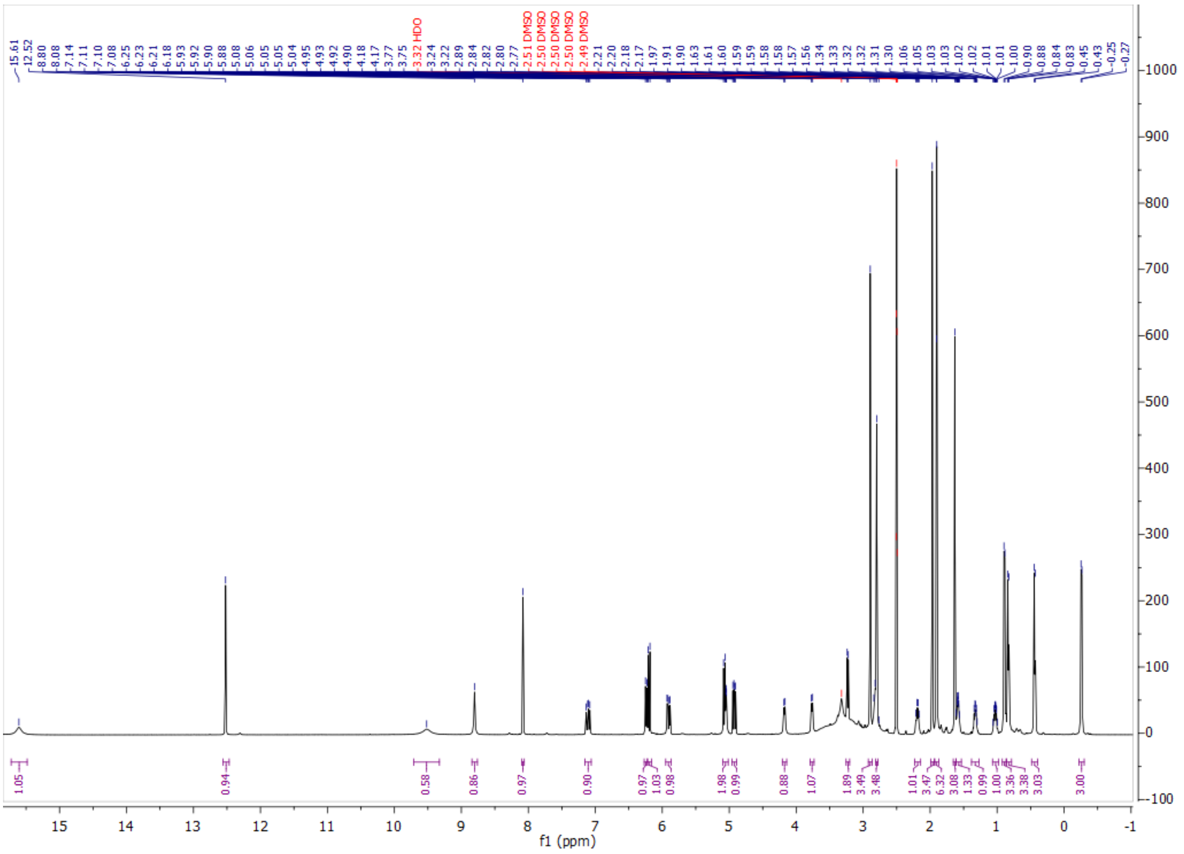

^{13}C NMR

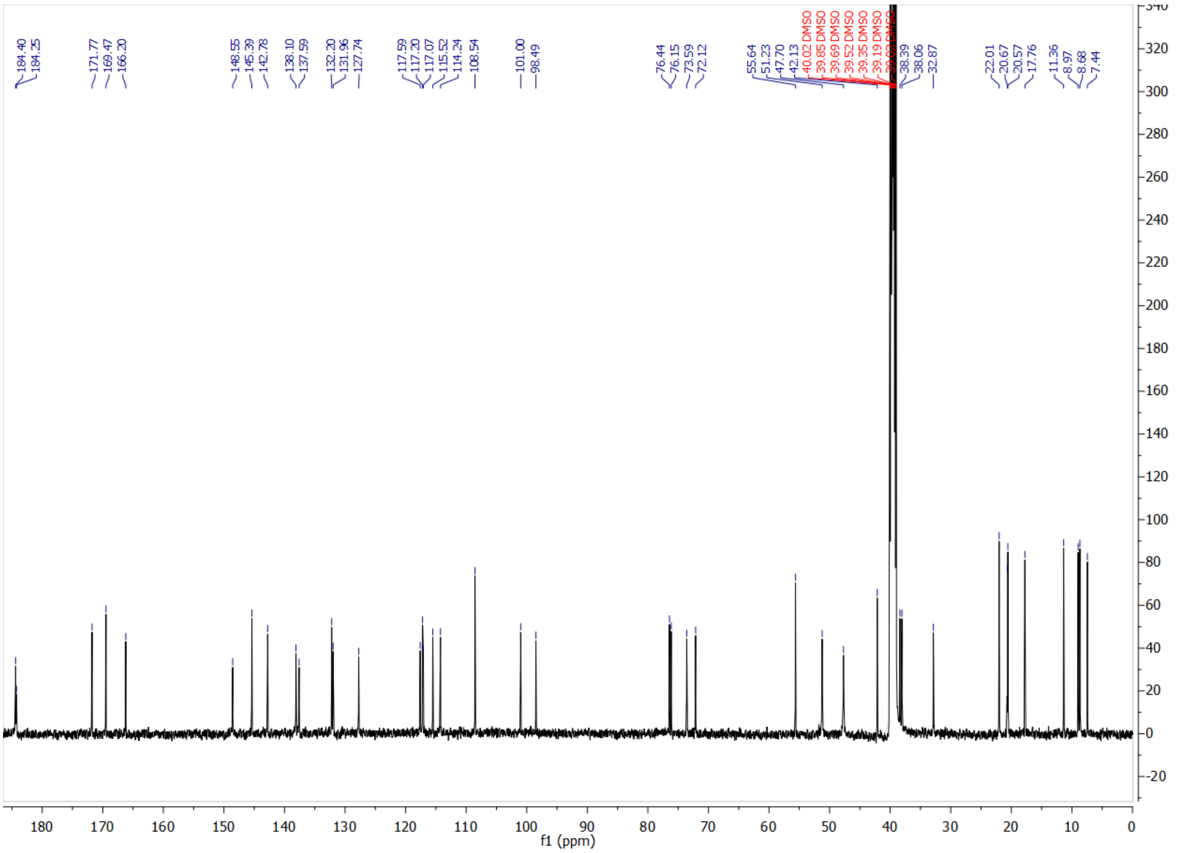

COMPOUND INFORMATION

Purity

```
Data File W:\analyti...\CGC_ECHO1-3_FirstPass 2021-03-20 13-21-54\040-D2B-D12-rifampicin.D
Sample Name: rifampicin

    =====================================================================
    Acq. Operator   : SYSTEM                        Seq. Line :  40
    Sample Operator : SYSTEM
    Acq. Instrument : LCMS test                      Location :   D2B-D12
    Injection Date  : 3/20/2021 8:37:50 PM                Inj :   1
                                                   Inj Volume : Inj prog
    Sequence File   : W:\analytical_LCMS_DATA\EUbOPEN\CGC_ECHO1-3_FirstPass 2021-03-20 13-21-54
                      \CGC_ECHO1-3_FirstPass.S
    Method          : W:\analytical_LCMS_DATA\EUbOPEN\CGC_ECHO1-3_FirstPass 2021-03-20 13-21-54
                      \CGL_FIRSTPASS_GENERALMETHOD_VIAL3+4_20210319.M (Sequence Method)
    Last changed    : 3/19/2021 5:35:24 PM by SYSTEM
    Method Info     : CGL wellplate, 0.5 uL of 10 mM DMSO, general method
```

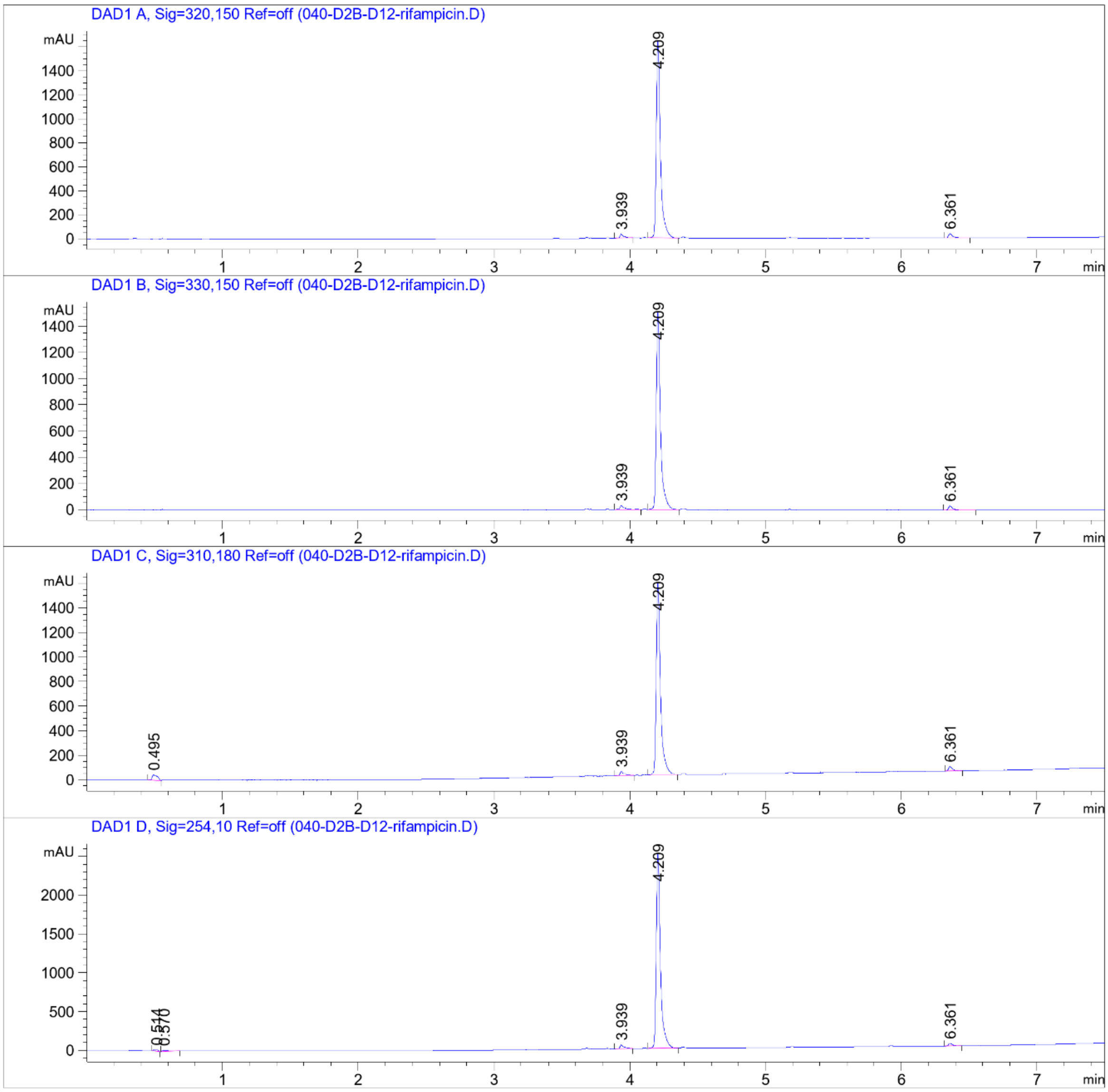

COMPOUND INFORMATION

Data File W:\analyti...\CGC_ECHO1-3_FirstPass 2021-03-20 13-21-54\040-D2B-D12-rifampicin.D
Sample Name: rifampicin

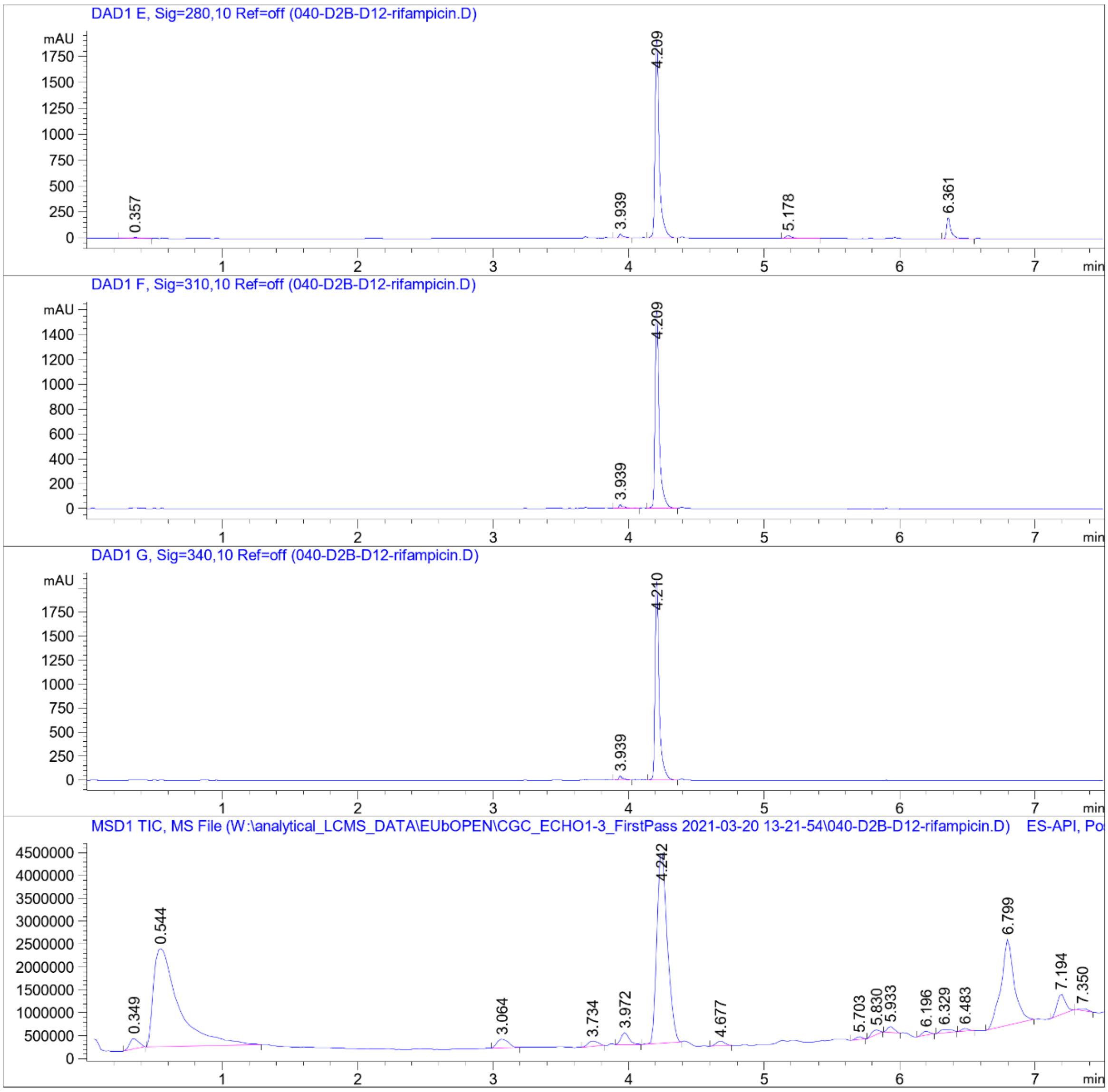

COMPOUND INFORMATION

Data File W:\analyti...\CGC_ECHO1-3_FirstPass 2021-03-20 13-21-54\040-D2B-D12-rifampicin.D
Sample Name: rifampicin

MS Signal: MSD1 TIC, MS File, ES-API, Pos, Scan, Frag: 70, "POS Scan"
Spectra from peak tops.
Noise Cutoff: 1000 counts.
Reportable Ion Abundance: > 50%.
LC Signal: DAD1 A, Sig=320,150 Ref=off
Peak matching window: 0.1 min

Retention Time (LC)	LC Area	Retention Time (MS)	MS Area	Mol. Weight or Ion
-	-	0.349	1036918	158.00 I
-	-	0.544	27798364	157.00 I
-	-	3.064	1111412	217.10 I
-	-	3.734	599228	274.20 I
3.939	69	3.972	1075451	808.40 I<br>807.40 I<br>749.40 I
4.209	3479	4.242	22688316	824.50 I<br>823.50 I
-	-	4.677	400338	326.30 I
-	-	5.703	170653	280.20 I
-	-	5.830	416009	296.20 I
-	-	5.933	439643	296.20 I<br>294.20 I<br>280.20 I
-	-	6.196	334373	280.20 I<br>228.20 I
6.361	73	6.329	427603	254.20 I
-	-	6.483	190447	280.20 I
-	-	6.799	12604060	282.20 I
-	-	7.194	1871416	284.20 I<br>282.20 I
-	-	7.350	228446	400.30 I<br>282.20 I

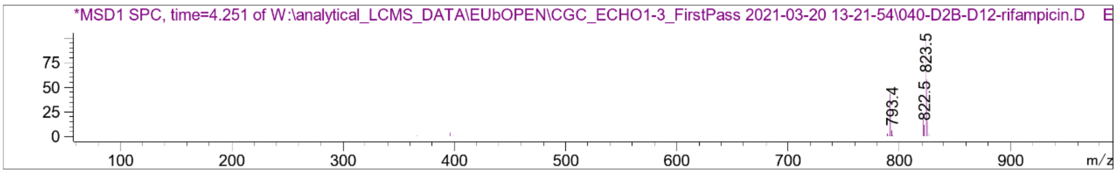

Supplement: Supplementary file 4 — Supplementary Data 1 [file 41467_2024_49493_MOESM4_ESM.zip › Rifampicin.pdf]
