# Supplementary material for: Chemogenomics for NR1 nuclear hormone receptors
Source: Nat Commun. 2024 Jun 18;15:5201. doi: 10.1038/s41467-024-49493-6 (PMC11189487; doi:10.1038/s41467-024-49493-6)

COMPOUND INFORMATION

Ro40-6055 (AM580)

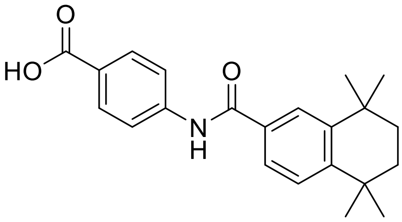

**CAS Registry No.:**	102121-60-8
**Formal Name:**	4-(5,5,8,8-tetramethyl-5,6,7,8-tetrahydronaphthalene-2-carboxamido)benzoic acid
**EUbOPEN ID:**	EUB0000567a
**Molecular Formula:**	$C_{22}H_{25}NO_3$
**Molecular Weight:**	351.45 g/mol
**Smiles:**	CC1(CCC(C2=C1C=CC(=C2)C(=O)NC3=CC=C(C=C3)C(=O)O)(C)C)C
**Recommended concentration:**	1 µM

Biological activity

		Type	IC_{50}/EC_{50} [µM]	Reference
Main NR target:	NR1B1 (RARα)	Agonist	0.0003	https://doi.org/10.1016/j.bmcl.2008.11.040
	NR1B2 (RARβ)	Agonist	0.009	
	NR1B3 (RARγ)	Agonist	0.01	
NR off-target:				

COMPOUND INFORMATION

Identity

1H NMR

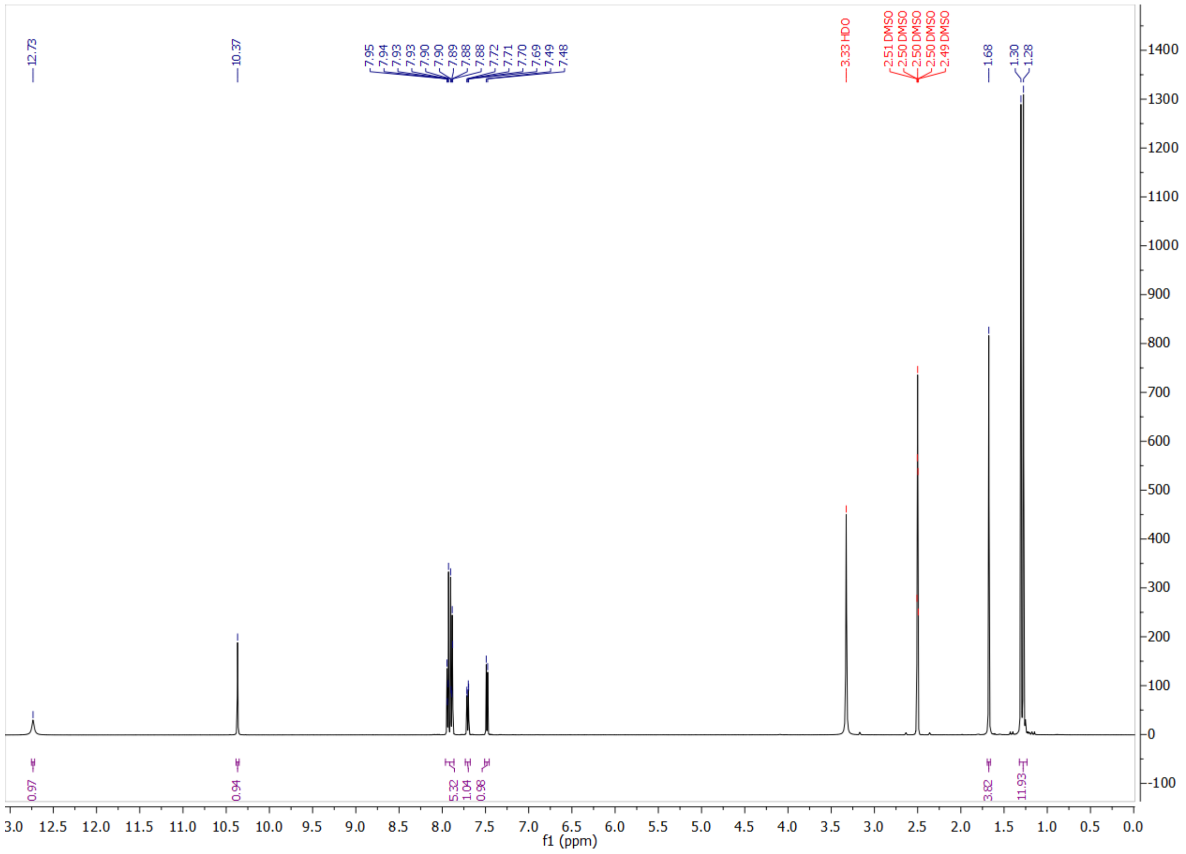

13C NMR

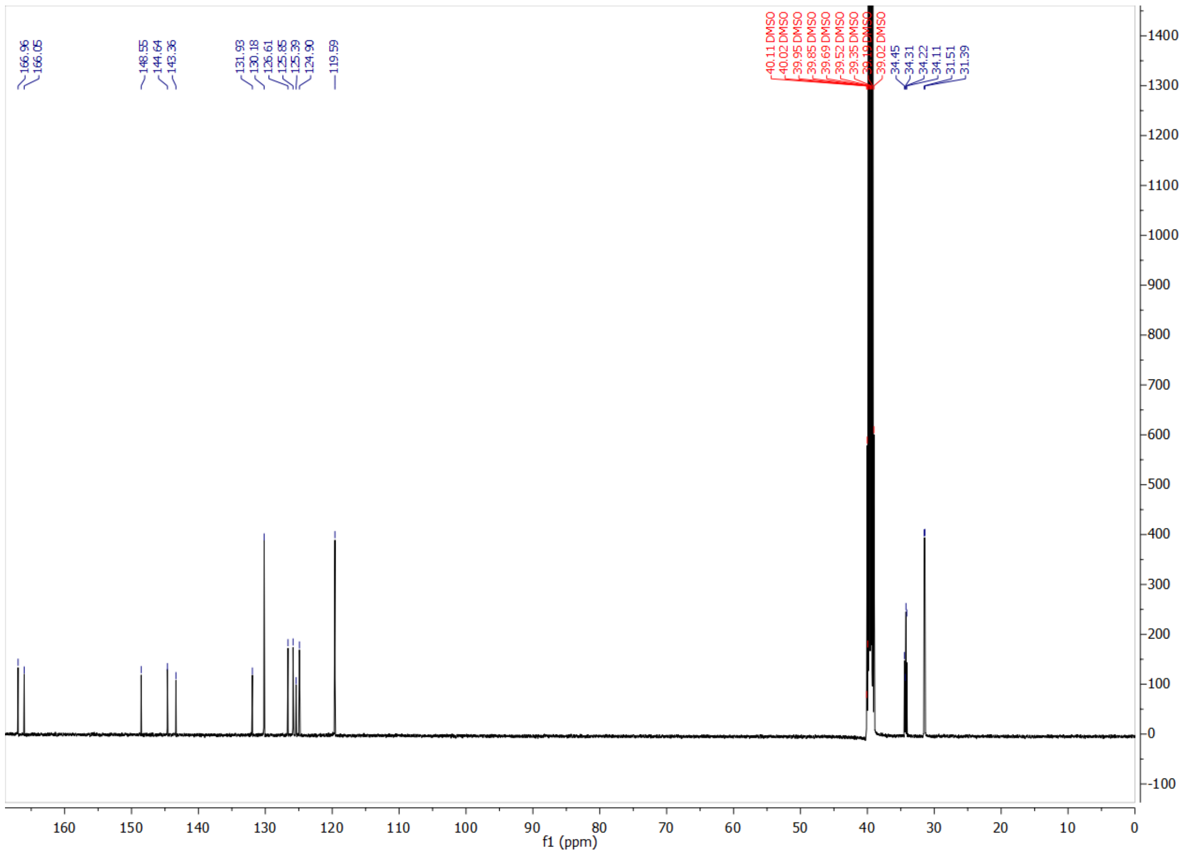

COMPOUND INFORMATION

Purity

Data File W:\analyti...\CGC_ECHO1-3_SecondPass 2021-03-25 18-09-21\022-D2F-B2-Ro 40-6055.D
Sample Name: Ro 40-6055

```
=====================================================================
Acq. Operator   : SYSTEM                          Seq. Line :  22
Sample Operator : SYSTEM
Acq. Instrument : LCMS test                        Location :   D2F-B2
Injection Date  : 3/25/2021 10:03:50 PM                 Inj :    1
                                                 Inj Volume : Inj prog
Sequence File   : W:\analytical_LCMS_DATA\EUbOPEN\CGC_ECHO1-3_SecondPass 2021-03-25 18-09-21
                  \CGC_ECHO1-3_SecondPass.S
Method          : W:\analytical_LCMS_DATA\EUbOPEN\CGC_ECHO1-3_SecondPass 2021-03-25 18-09-21
                  \CGL_SECONDPASS_NONPOLCOMP_VIAL2+4_20210323.M (Sequence Method)
Last changed    : 3/25/2021 4:32:02 PM by SYSTEM
Method Info     : CGL wellplate, 0.5 uL of 10 mM DMSO. Dilution with MeCN only (9+9 uL)
```

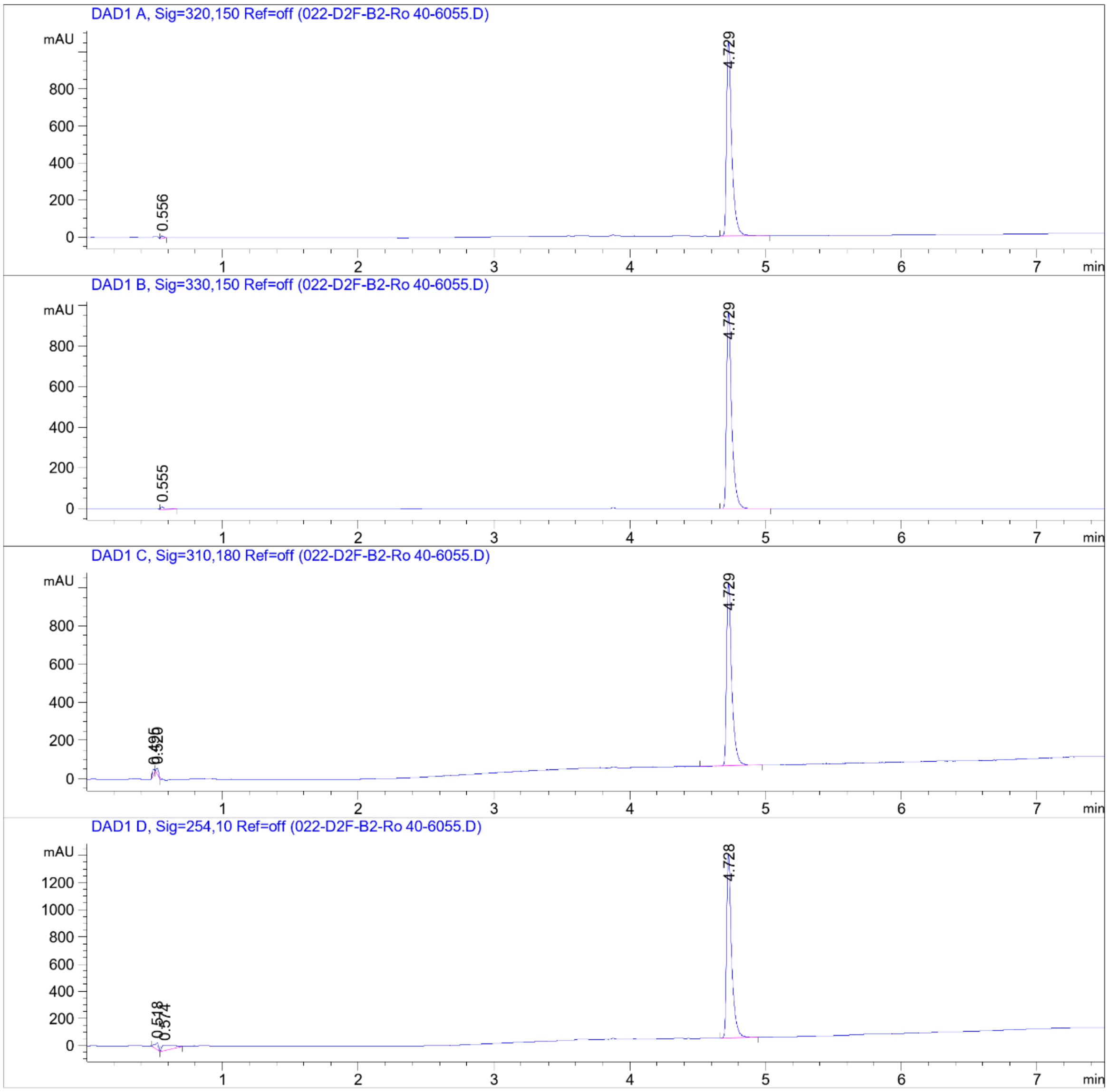

COMPOUND INFORMATION

Data File W:\analyti...\CGC_ECHO1-3_SecondPass 2021-03-25 18-09-21\022-D2F-B2-Ro 40-6055.D
Sample Name: Ro 40-6055

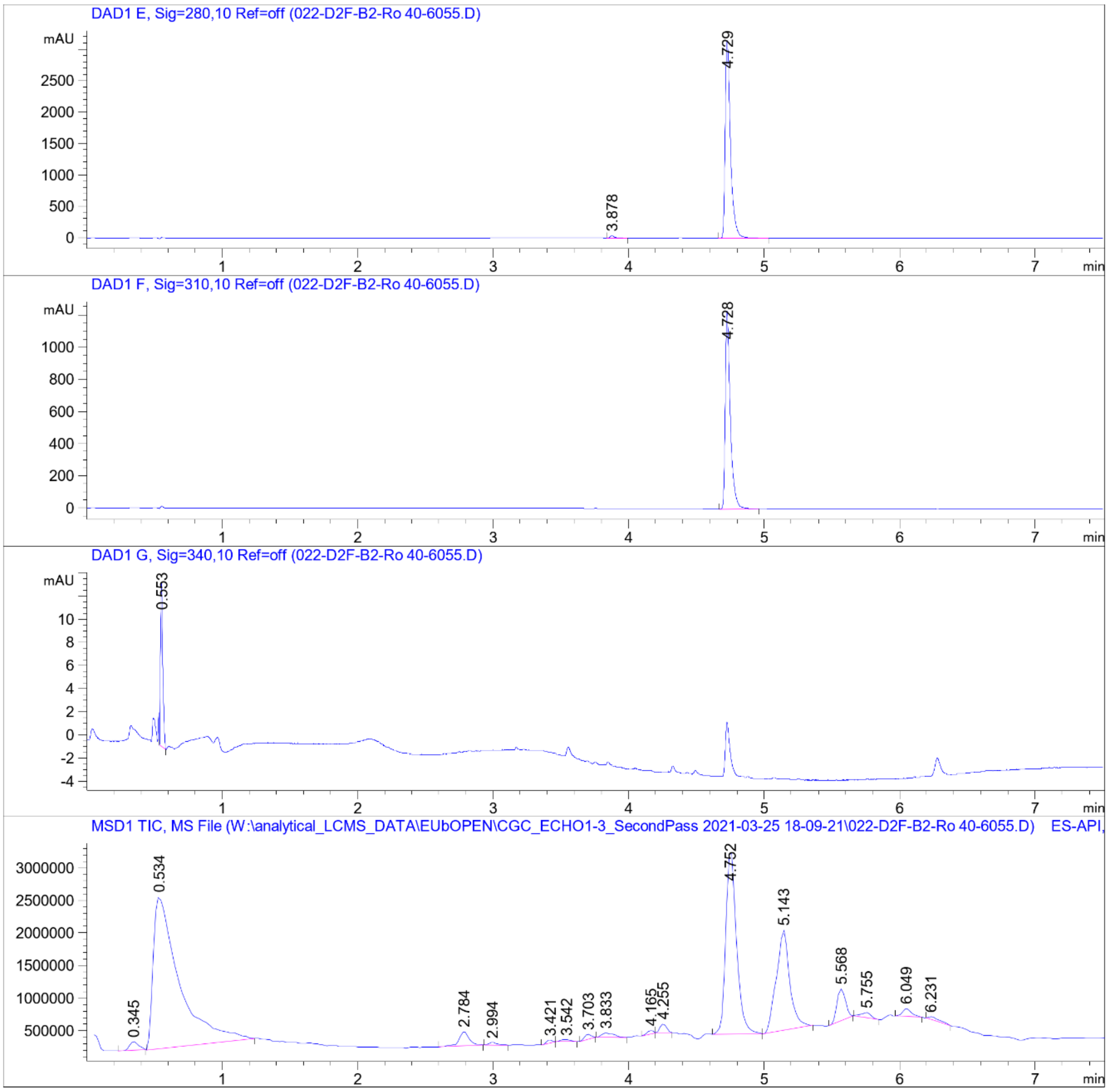

COMPOUND INFORMATION

Data File W:\analyti...\CGC_ECHO1-3_SecondPass 2021-03-25 18-09-21\022-D2F-B2-Ro 40-6055.D
Sample Name: Ro 40-6055

MS Signal: MSD1 TIC, MS File, ES-API, Pos, Scan, Frag: 70, "POS Scan"
Spectra from peak tops.
Noise Cutoff: 1000 counts.
Reportable Ion Abundance: > 50%.
LC Signal: DAD1 A, Sig=320,150 Ref=off
Peak matching window: 0.1 min

Retention Time (LC)	LC Area	Retention Time (MS)	MS Area	Mol. Weight or Ion
-	-	0.345	598066	200.00 I
				183.00 I
				159.00 I
				142.00 I
				110.10 I
0.556	20	0.534	30935600	157.00 I
-	-	2.784	1077283	217.10 I
-	-	2.994	160001	274.30 I
-	-	3.421	138476	326.40 I
-	-	3.542	163486	332.30 I
				326.30 I
				296.30 I
				282.30 I
				200.00 I
				159.00 I
				158.20 I
				151.20 I
				111.10 I
				110.10 I
				102.20 I
-	-	3.703	279175	214.10 I
-	-	3.833	447341	316.20 I
				298.30 I
-	-	4.165	160749	280.30 I
-	-	4.255	460486	296.30 I
4.729	2788	4.752	15417600	352.20 I
-	-	5.143	10493235	282.30 I
-	-	5.568	2079847	381.30 I
				359.30 I
				341.30 I
				284.30 I
				282.30 I
-	-	5.755	410310	400.40 I
				282.30 I
-	-	6.049	580429	338.30 I
				282.30 I
-	-	6.231	248440	282.20 I

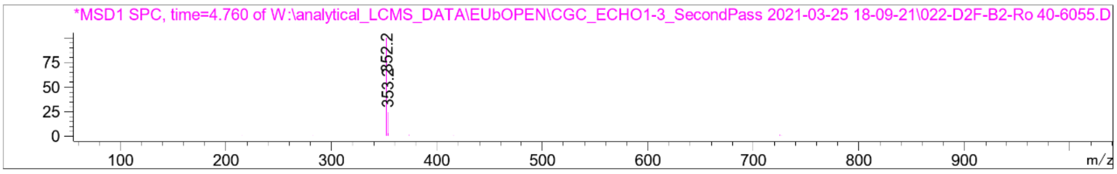

Supplement: Supplementary file 4 — Supplementary Data 1 [file 41467_2024_49493_MOESM4_ESM.zip › Ro40-6055.pdf]
