# Supplementary material for: Chemogenomics for NR1 nuclear hormone receptors
Source: Nat Commun. 2024 Jun 18;15:5201. doi: 10.1038/s41467-024-49493-6 (PMC11189487; doi:10.1038/s41467-024-49493-6)

COMPOUND INFORMATION

Rosiglitazone

**CAS Registry No.:**	122320-73-4
**Formal Name:**	5-(4-(2-(methyl(pyridin-2-yl)amino)ethoxy)benzyl)thiazolidine-2,4-dione
**EUbOPEN ID:**	EUB0000190b
**Molecular Formula:**	$C_{18}H_{19}N_3O_3S$
**Molecular Weight:**	357.43 g/mol
**Smiles:**	CN(CCOC1=CC=C(C=C1)CC2C(=O)NC(=O)S2)C3=CC=CC=N3
**Recommended concentration:**	1 µM

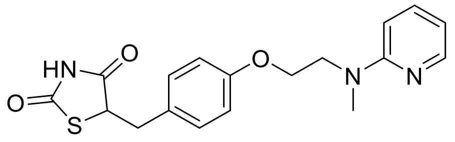

Biological activity

		Type	IC_{50}/EC_{50} [µM]	Reference
Main NR target:	NR1C3 (PPARγ)	Agonist	0.035	https://doi.org/10.1016/j.bmcl.2010.02.071
NR off-target:				

COMPOUND INFORMATION

EUbOPEN
Enabling & Unlocking Biology in the OPEN

Identity

^{1}H NMR

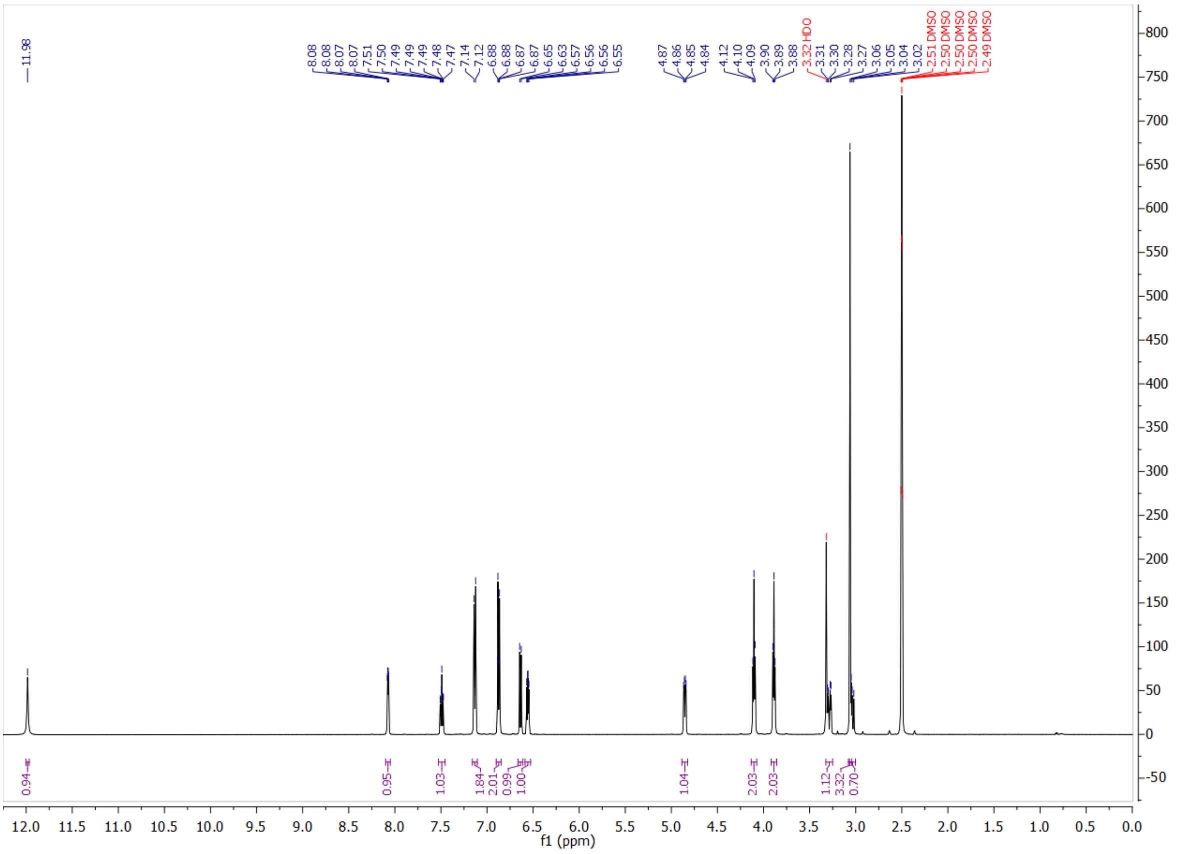

^{13}C NMR

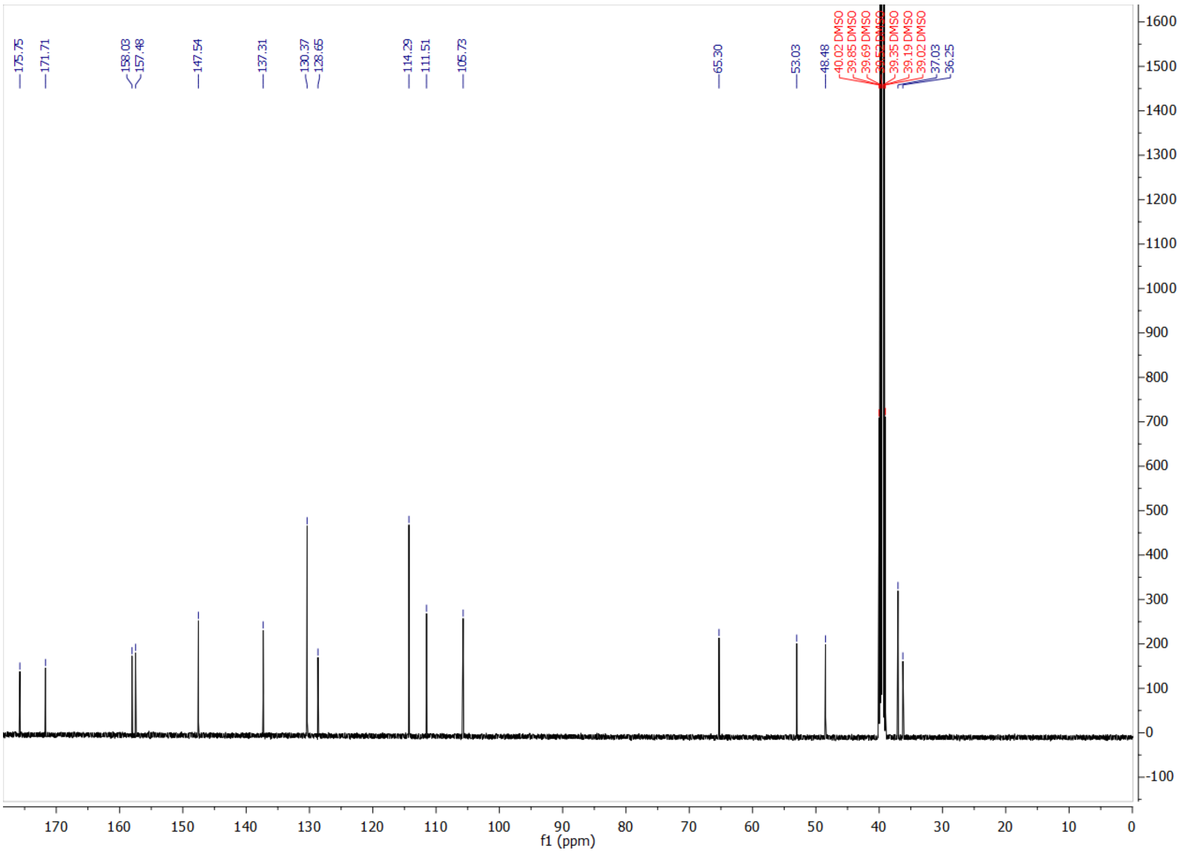

Purity

```
Data File W:\analyti...C_ECHO1-2_SecondPass 2021-03-23 18-53-53\006-D2F-A7-rosiglitazone.D
Sample Name: rosiglitazone

    =====================================================================
    Acq. Operator   : SYSTEM                          Seq. Line :    6
    Sample Operator : SYSTEM
    Acq. Instrument : LCMS test                        Location :   D2F-A7
    Injection Date  : 3/23/2021 7:52:59 PM                  Inj :    1
                                                     Inj Volume : Inj prog
    Sequence File   : W:\analytical_LCMS_DATA\EUbOPEN\CGC_ECHO1-2_SecondPass 2021-03-23 18-53-53
                      \CGC_ECHO1-2_SecondPass.S
    Method          : W:\analytical_LCMS_DATA\EUbOPEN\CGC_ECHO1-2_SecondPass 2021-03-23 18-53-53
                      \CGL_SECONDPASS_NONPOLCOMP_VIAL2+4_20210323.M (Sequence Method)
    Last changed    : 3/23/2021 6:50:05 PM by SYSTEM
    Method Info     : CGL wellplate, 0.5 uL of 10 mM DMSO. Dilution with MeCN only (9+9 uL)
```

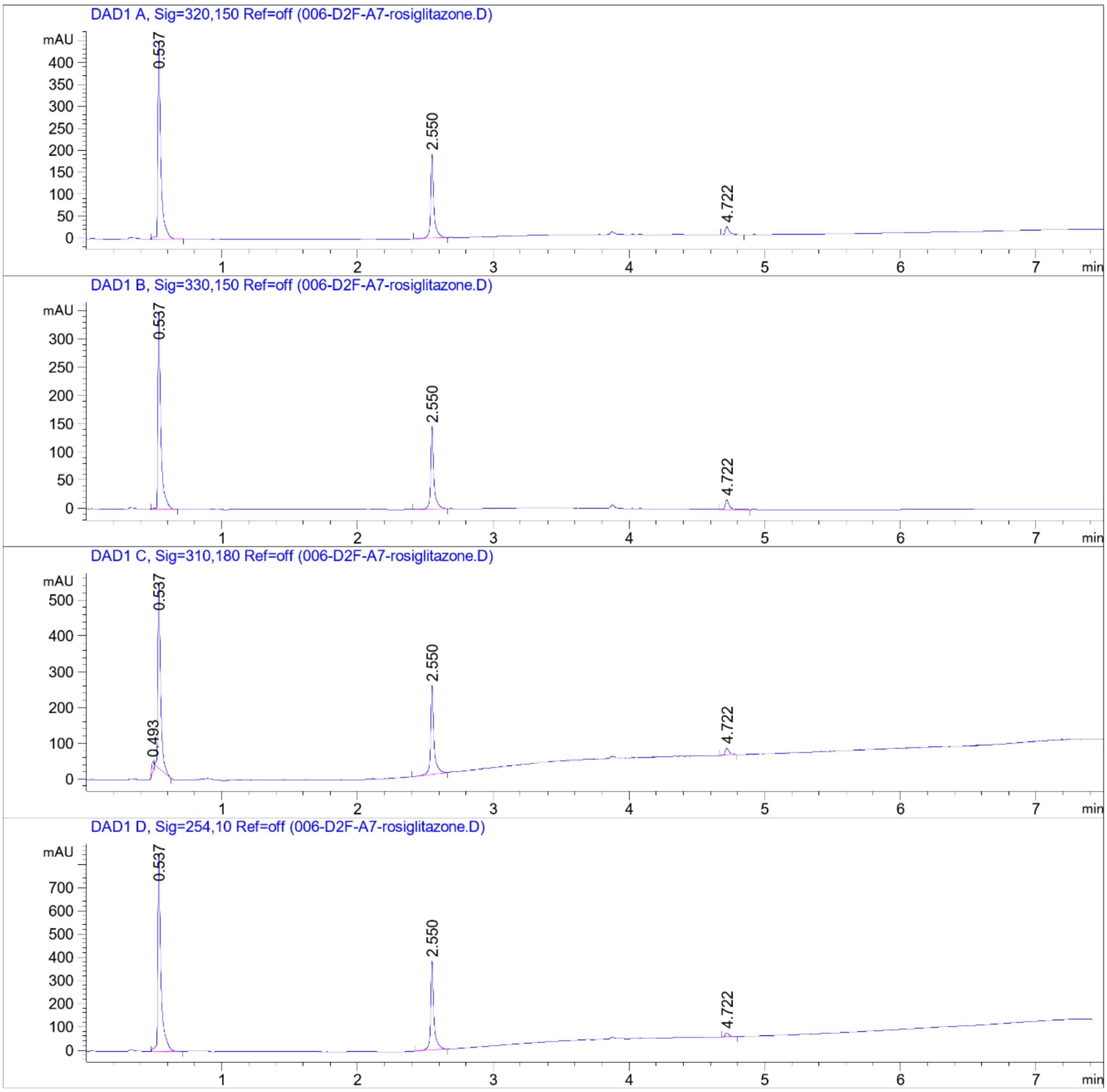

COMPOUND INFORMATION

Data File W:\analyti...C_ECHO1-2_SecondPass 2021-03-23 18-53-53\006-D2F-A7-rosiglitazone.D
Sample Name: rosiglitazone

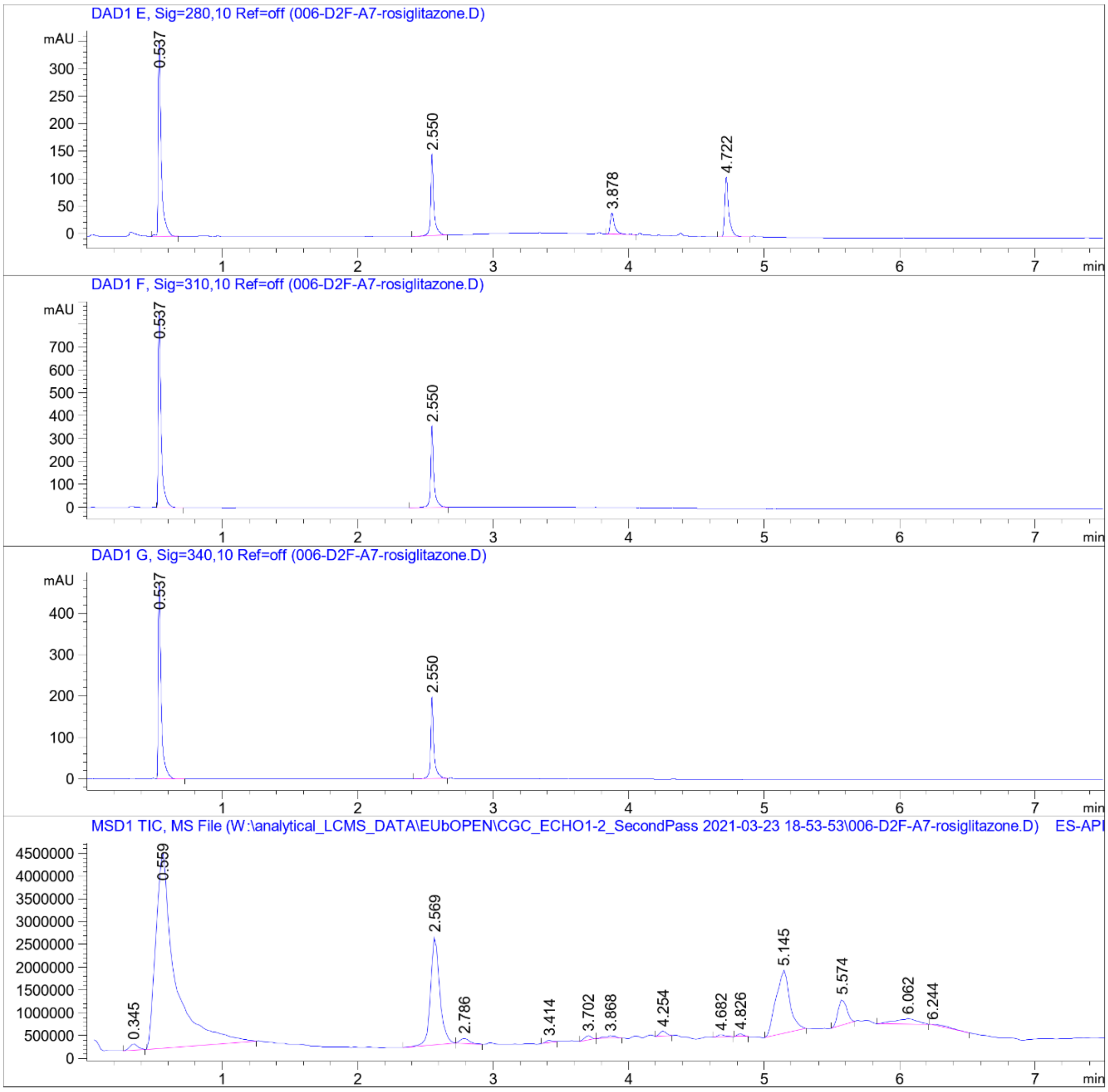

COMPOUND INFORMATION

Data File W:\analyti...C_ECHO1-2_SecondPass 2021-03-23 18-53-53\006-D2F-A7-rosiglitazone.D
Sample Name: rosiglitazone

MS Signal: MSD1 TIC, MS File, ES-API, Pos, Scan, Frag: 70, "POS Scan"
Spectra from peak tops.
Noise Cutoff: 1000 counts.
Reportable Ion Abundance: > 50%.
LC Signal: DAD1 A, Sig=320,150 Ref=off
Peak matching window: 0.1 min

Retention Time (LC)	LC Area	Retention Time (MS)	MS Area	Mol. Weight or Ion
-	-	0.345	574970	200.00 I
				183.00 I
				159.00 I
				142.00 I
				110.00 I
0.537	710	0.559	42584952	358.10 I
2.550	317	2.569	11259365	358.10 I
-	-	2.786	521762	217.10 I
-	-	3.414	182448	326.40 I
-	-	3.702	315982	312.20 I
				214.10 I
-	-	3.868	324485	316.30 I
				296.30 I
				288.20 I
				282.30 I
				268.20 I
				225.10 I
				200.00 I
				158.90 I
				111.10 I
				102.20 I
-	-	4.254	369752	296.30 I
4.722	37	4.682	192697	254.20 I
-	-	4.826	178529	280.30 I
-	-	5.145	9663870	282.30 I
-	-	5.574	2464099	359.30 I
				341.30 I
				284.30 I
				282.30 I
-	-	6.062	1318681	282.20 I
-	-	6.244	278382	282.30 I

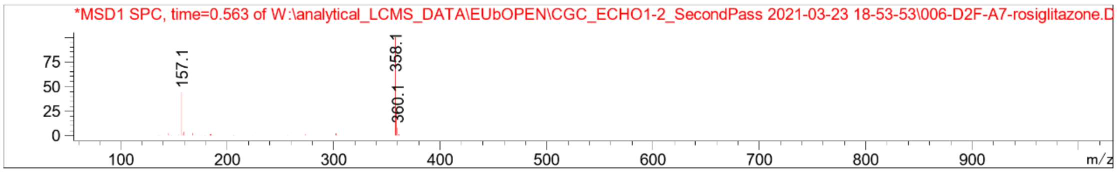

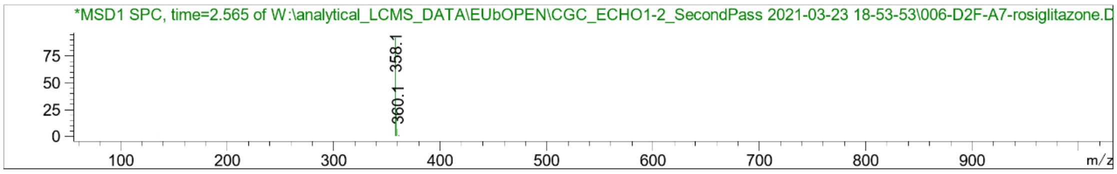

Supplement: Supplementary file 4 — Supplementary Data 1 [file 41467_2024_49493_MOESM4_ESM.zip › Rosiglitazone.pdf]
