# Supplementary material for: Chemogenomics for NR1 nuclear hormone receptors
Source: Nat Commun. 2024 Jun 18;15:5201. doi: 10.1038/s41467-024-49493-6 (PMC11189487; doi:10.1038/s41467-024-49493-6)

# COMPOUND INFORMATION

# S26948

| | | |
|---|---|---|
| **CAS Registry No.:** | 353280-43-0 | |
| **Formal Name:** | Dimethyl 2-(4-(2-(6-benzoyl-2-oxobenzo[d]thiazol-3(2H)-yl)ethoxy)benzyl)malonate | |
| **EUbOPEN ID:** | EUB0000571a | |
| **Molecular Formula:** | $C_{28}H_{25}NO_7S$ | |
| **Molecular Weight:** | 519.57 g/mol | |
| **Smiles:** | COC(=O)C(CC1=CC=C(C=C1)OCCN2C3=C(C=C(C=C3)C(=O)C4=CC=CC=C4)SC2=O)C(=O)OC | |
| **Recommended concentration:** | 1 µM | |

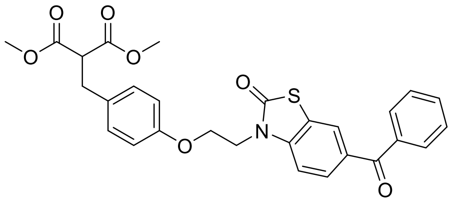

**Biological activity**

| | | Type | $IC_{50}/EC_{50}$ [µM] | Reference |
|---|---|---|---|---|
| Main NR target: | NR1C3 (PPARγ) | Agonist | 0.042 | inhouse |
| NR off-target: | | | | |

# **COMPOUND** INFORMATION

## Identity

### $^{1}$H NMR

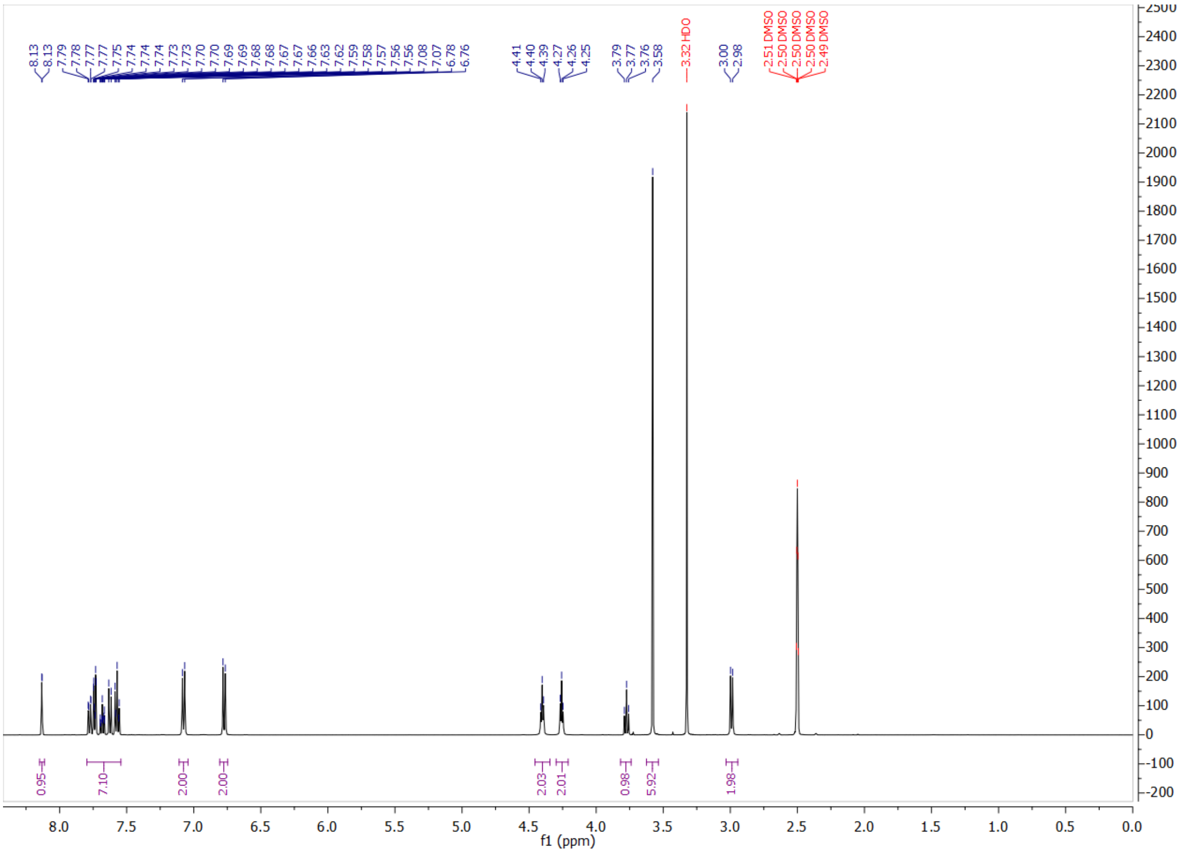


### $^{13}$C NMR

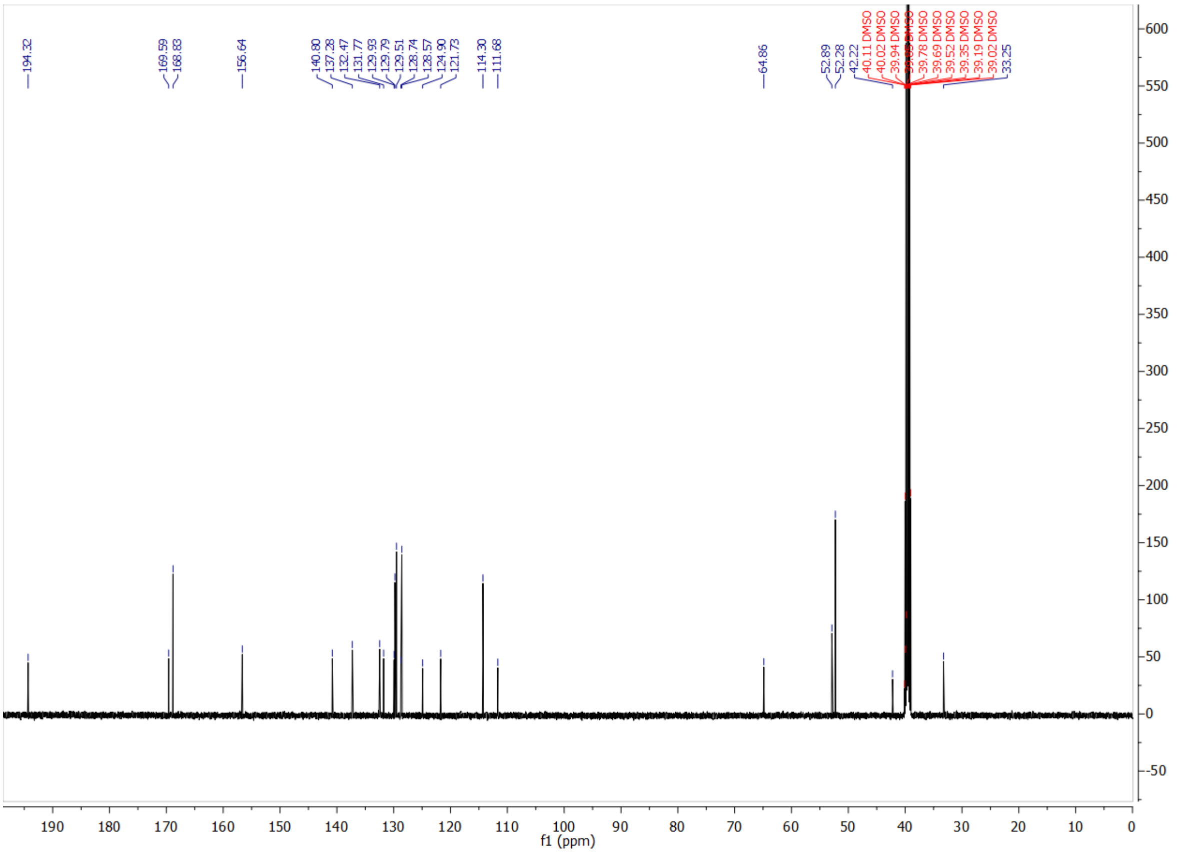

# **COMPOUND** INFORMATION

## Purity

```
Data File W:\analyti...bOPEN\CGC_ECHO1-3_FirstPass 2021-03-20 13-21-54\057-D2B-E6-S26948.D
Sample Name: S26948

    =====================================================================
    Acq. Operator   : SYSTEM                           Seq. Line :   57
    Sample Operator : SYSTEM
    Acq. Instrument : LCMS test                         Location :   D2B-E6
    Injection Date  : 3/20/2021 11:46:12 PM                  Inj :   1
                                                      Inj Volume : Inj prog
    Sequence File   : W:\analytical_LCMS_DATA\EUbOPEN\CGC_ECHO1-3_FirstPass 2021-03-20 13-21-54
                      \CGC_ECHO1-3_FirstPass.S
    Method          : W:\analytical_LCMS_DATA\EUbOPEN\CGC_ECHO1-3_FirstPass 2021-03-20 13-21-54
                      \CGL_FIRSTPASS_GENERALMETHOD_VIAL3+4_20210319.M (Sequence Method)
    Last changed    : 3/19/2021 5:35:24 PM by SYSTEM
    Method Info     : CGL wellplate, 0.5 uL of 10 mM DMSO, general method
```

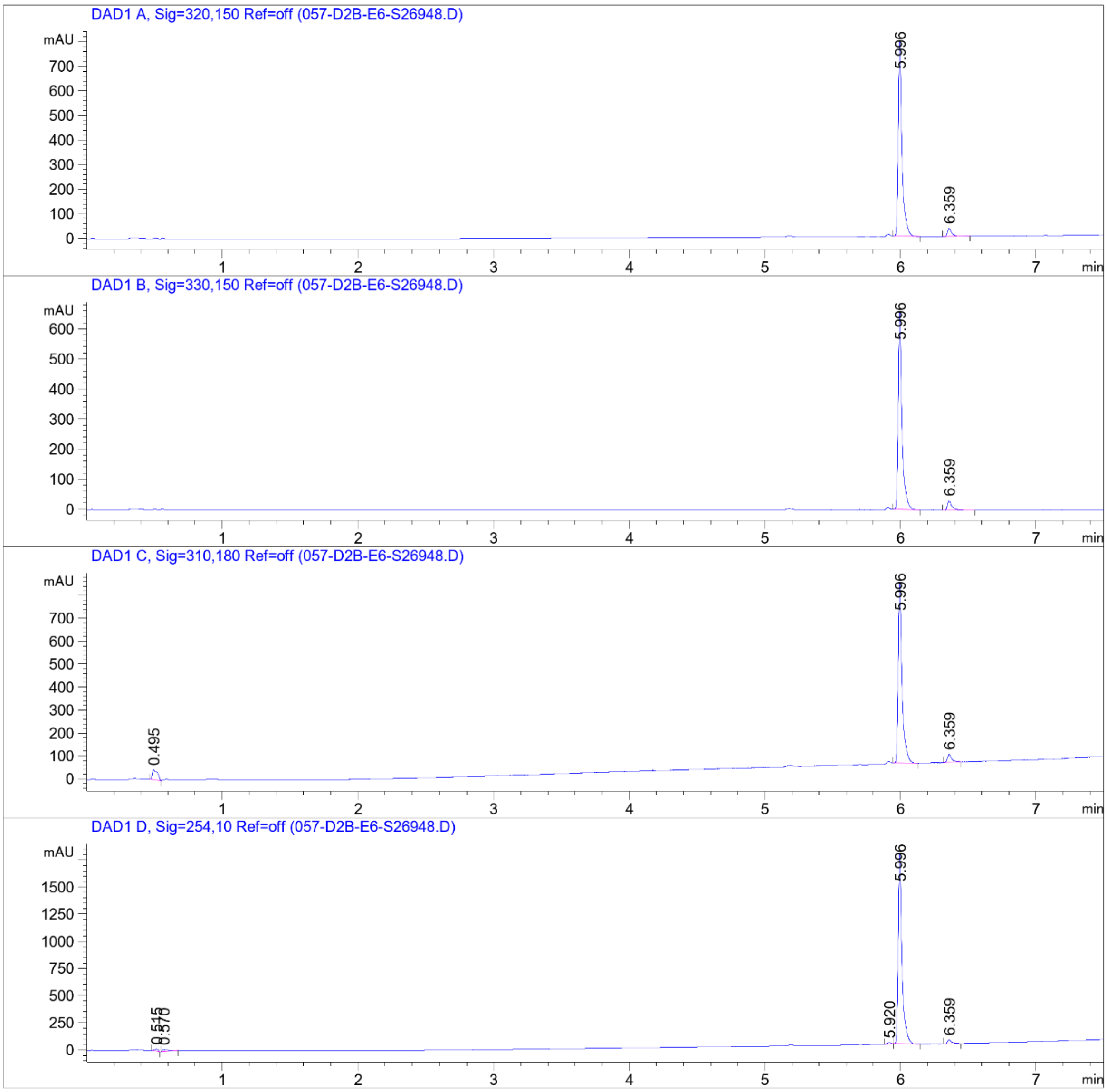

# **COMPOUND** INFORMATION

Data File W:\analyti...bOPEN\CGC_ECHO1-3_FirstPass 2021-03-20 13-21-54\057-D2B-E6-S26948.D
Sample Name: S26948

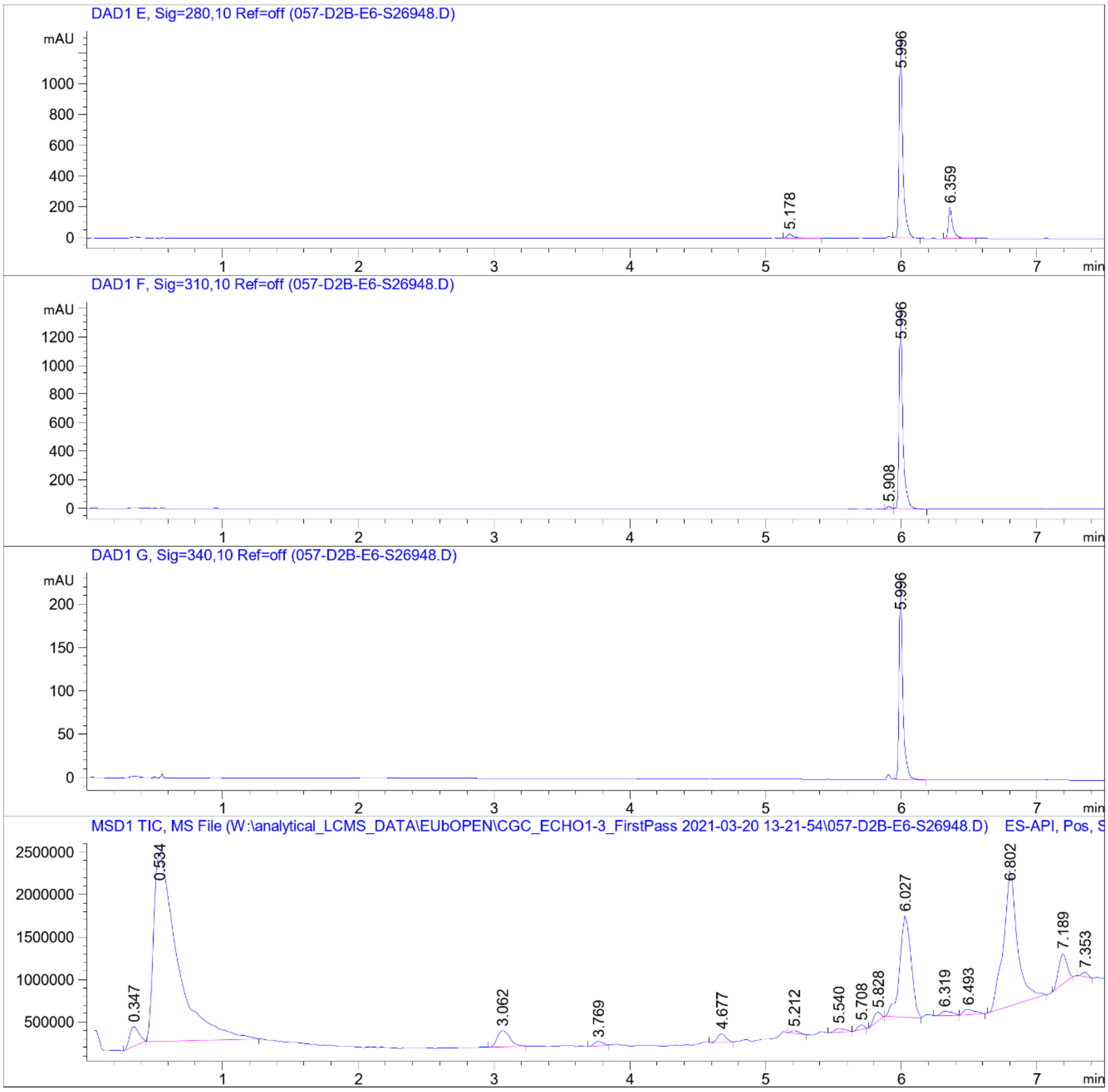

# **COMPOUND** INFORMATION

Data File W:\analyti...bOPEN\CGC_ECHO1-3_FirstPass 2021-03-20 13-21-54\057-D2B-E6-S26948.D
Sample Name: S26948

MS Signal: MSD1 TIC, MS File, ES-API, Pos, Scan, Frag: 70, "POS Scan"
Spectra from peak tops.
Noise Cutoff: 1000 counts.
Reportable Ion Abundance: > 50%.
LC Signal: DAD1 A, Sig=320,150 Ref=off
Peak matching window: 0.1 min

| Retention Time (LC) | LC Area | Retention Time (MS) | MS Area | Mol. Weight or Ion |
|---|---|---|---|---|
| - | - | 0.347 | 1083867 | 158.00 I |
| - | - | 0.534 | 27272738 | 157.00 I |
| - | - | 3.062 | 1219014 | 217.00 I |
| - | - | 3.769 | 229397 | 274.20 I |
| - | - | 4.677 | 447964 | 326.30 I |
| - | - | 5.212 | 120461 | 316.20 I |
| | | | | 102.10 I |
| - | - | 5.540 | 237806 | 326.20 I |
| | | | | 295.10 I |
| | | | | 282.20 I |
| | | | | 280.20 I |
| | | | | 102.20 I |
| - | - | 5.708 | 171409 | 280.20 I |
| - | - | 5.828 | 395846 | 296.20 I |
| 5.996 | 1514 | 6.027 | 7025183 | 520.10 I |
| 6.359 | 74 | 6.319 | 304671 | 254.20 I |
| - | - | 6.493 | 372301 | 280.20 I |
| - | - | 6.802 | 11450855 | 282.20 I |
| - | - | 7.189 | 1519516 | 284.30 I |
| | | | | 282.20 I |
| - | - | 7.353 | 156871 | 400.30 I |
| | | | | 282.20 I |

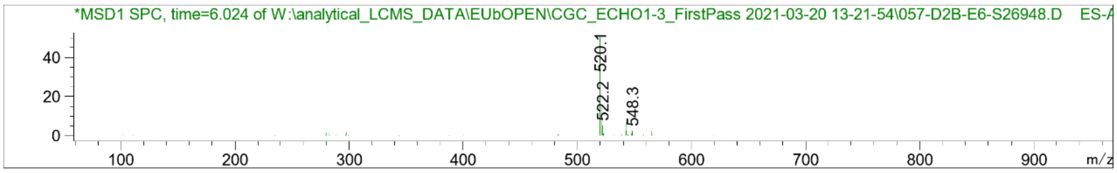

# **COMPOUND** INFORMATION

## Biological activity

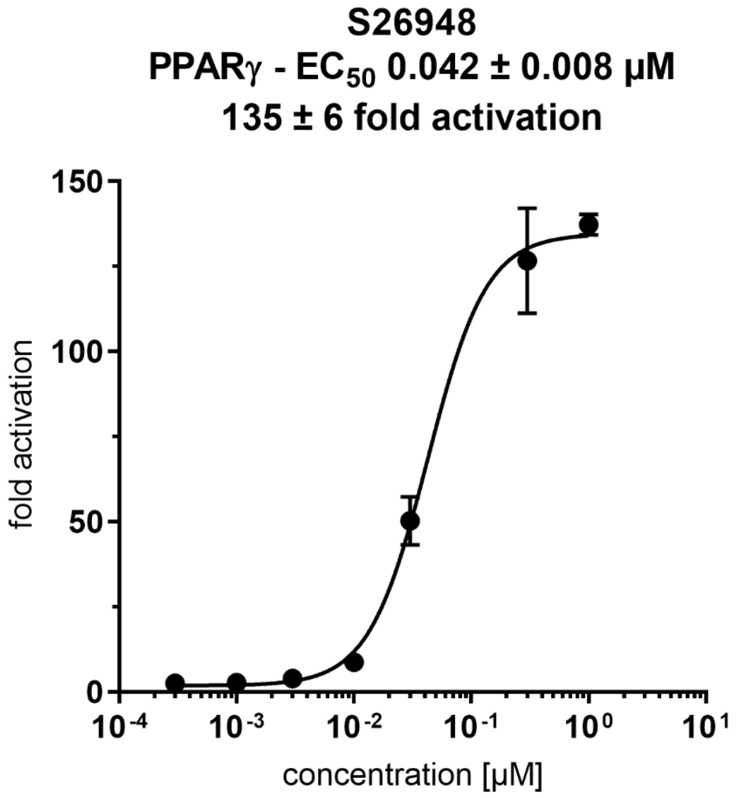

Supplement: Supplementary file 4 — Supplementary Data 1 [file 41467_2024_49493_MOESM4_ESM.zip › S26948.pdf]
