# Supplementary material for: Chemogenomics for NR1 nuclear hormone receptors
Source: Nat Commun. 2024 Jun 18;15:5201. doi: 10.1038/s41467-024-49493-6 (PMC11189487; doi:10.1038/s41467-024-49493-6)

COMPOUND INFORMATION

Seocalcitol

**CAS Registry No.:**	134404-52-7
**Formal Name:**	(1R,3S,Z)-5-(2-((1R,3aS,7aR,E)-1-((R,3E,5E)-7-ethyl-7-hydroxynona-3,5-dien-2-yl)-7a-methyloctahydro-4H-inden-4-ylidene)ethylidene)-4-methylenecyclohexane-1,3-diol
**EUBOPEN ID:**	EUB0000582a
**Molecular Formula:**	$C_{30}H_{46}O_3$
**Molecular Weight:**	454.70 g/mol
**Smiles:**	CCC(/C=C/C=C/[C@H]([C@H]1CC[C@@H]2[C@]1(C)CCC/C2=C\C=C3C[C@@H](O)C[C@H](O)C\3=C)C)(O)CC
**Recommended concentration:**	1 µM

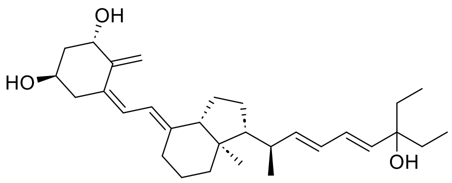

Biological activity

		Type	IC_{50}/EC_{50} [µM]	Reference
Main NR target:	NR1I1 (VDR)	Agonist	0.11	inhouse
NR off-target:				

COMPOUND INFORMATION

Identity

^{1}H NMR

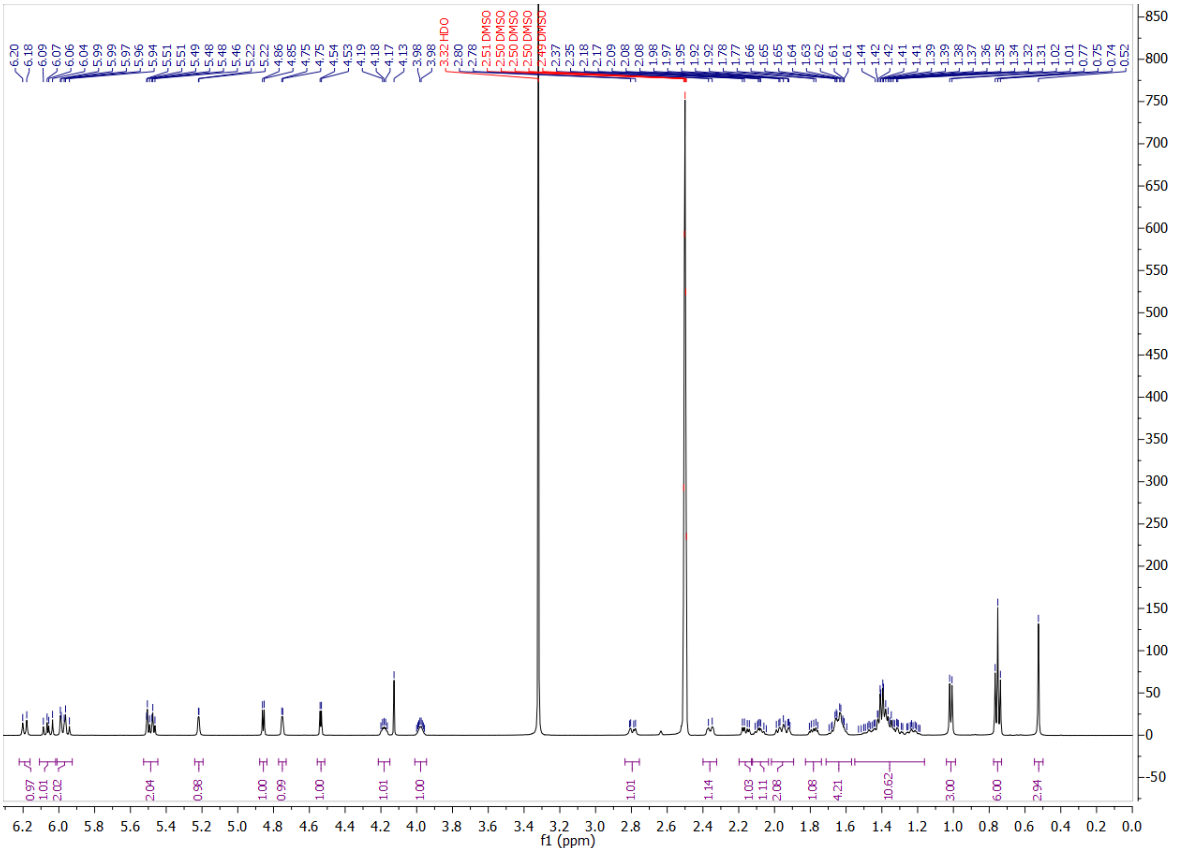

^{13}C NMR

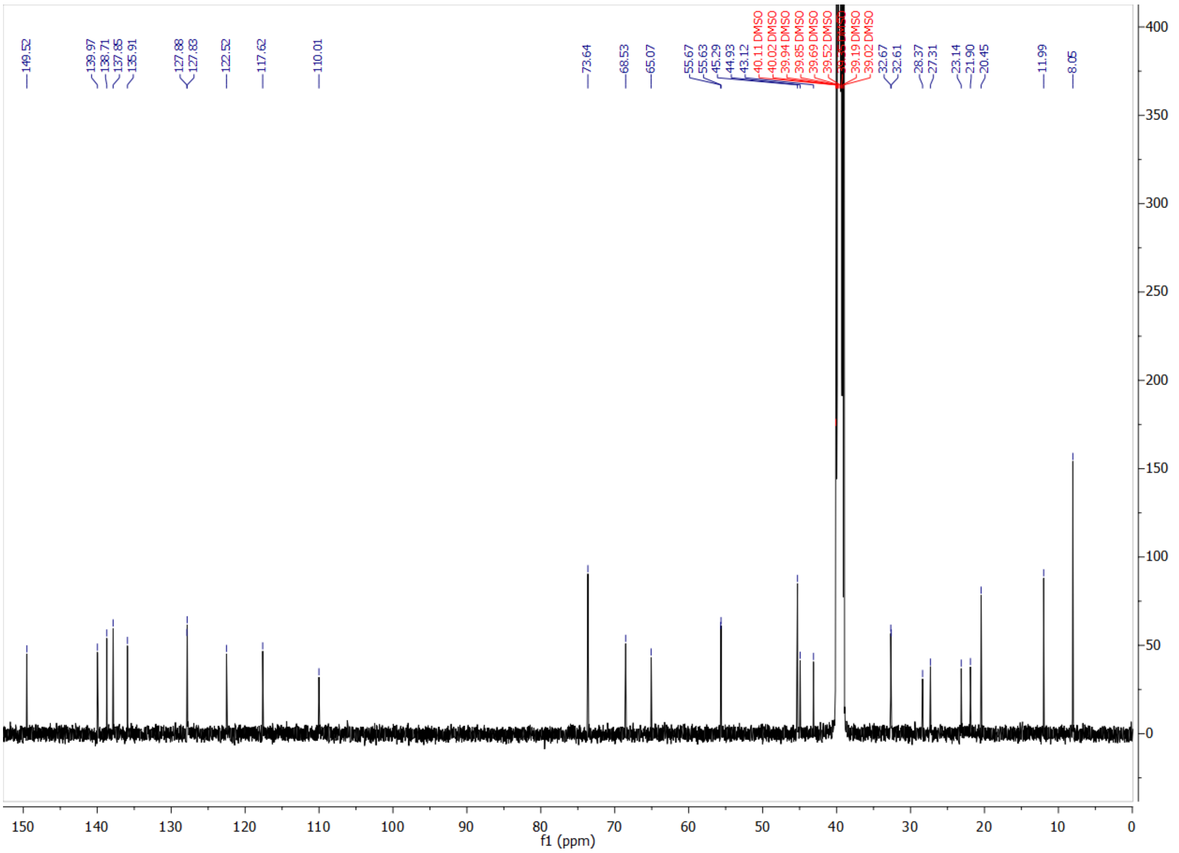

COMPOUND INFORMATION

Purity

LC-MS

M$_r$ 454.70

MS: ESI-positive, m/z 437/419 (blue), m/z 437/133 (red), loss of H2O under these conditions (see e.g. D.A. Vollmer et al., Mass Spectrom. Rev. 2-23, 34, 2015)

LC: 0.1% HCOOH/ACN (20/80)

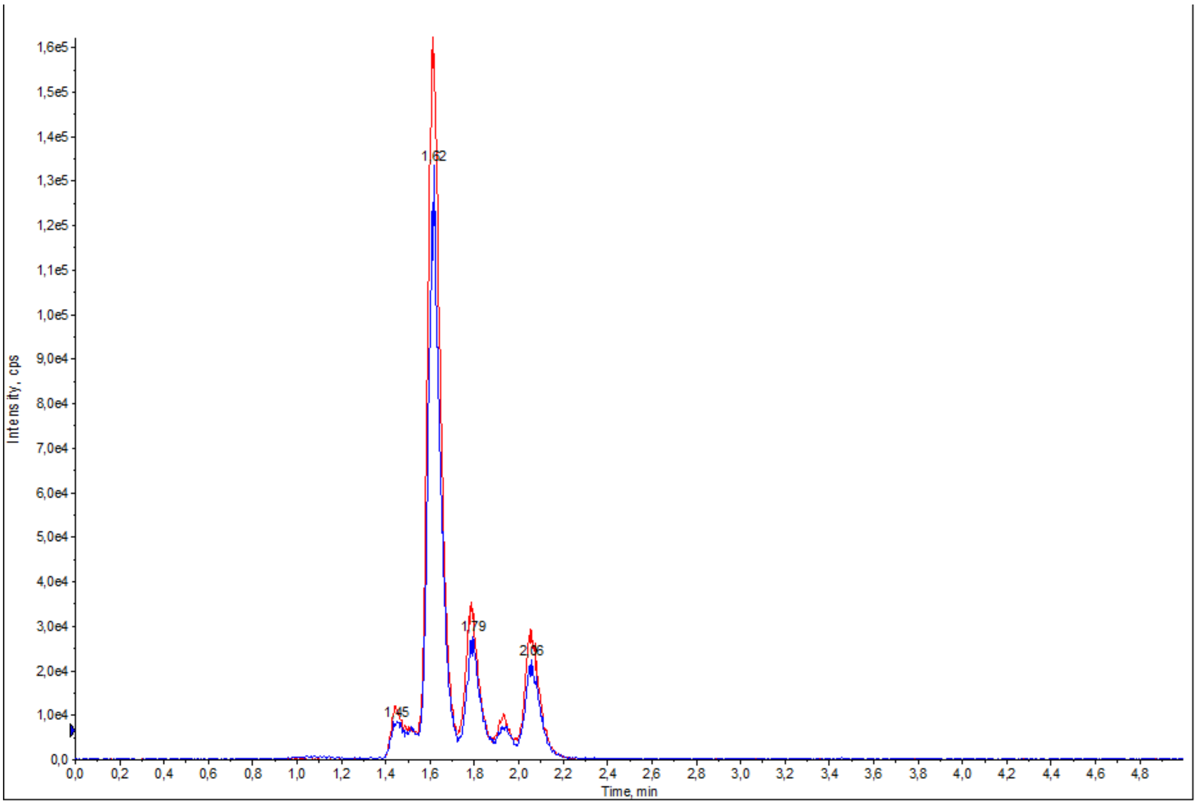

COMPOUND INFORMATION

LC-UV

LC: 0.1% HCOOH/ACN (30/70)

DAD: 240, 250, 265 (XWC), 280, 300 nm

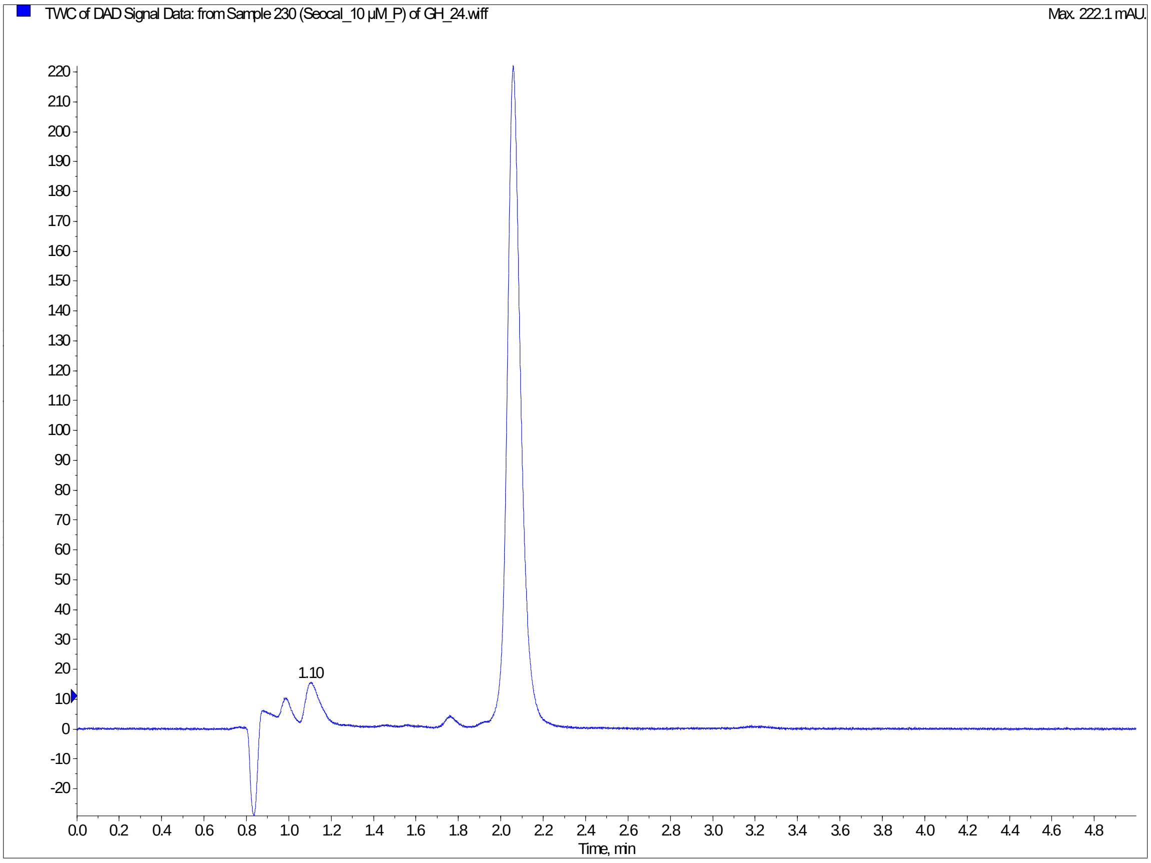

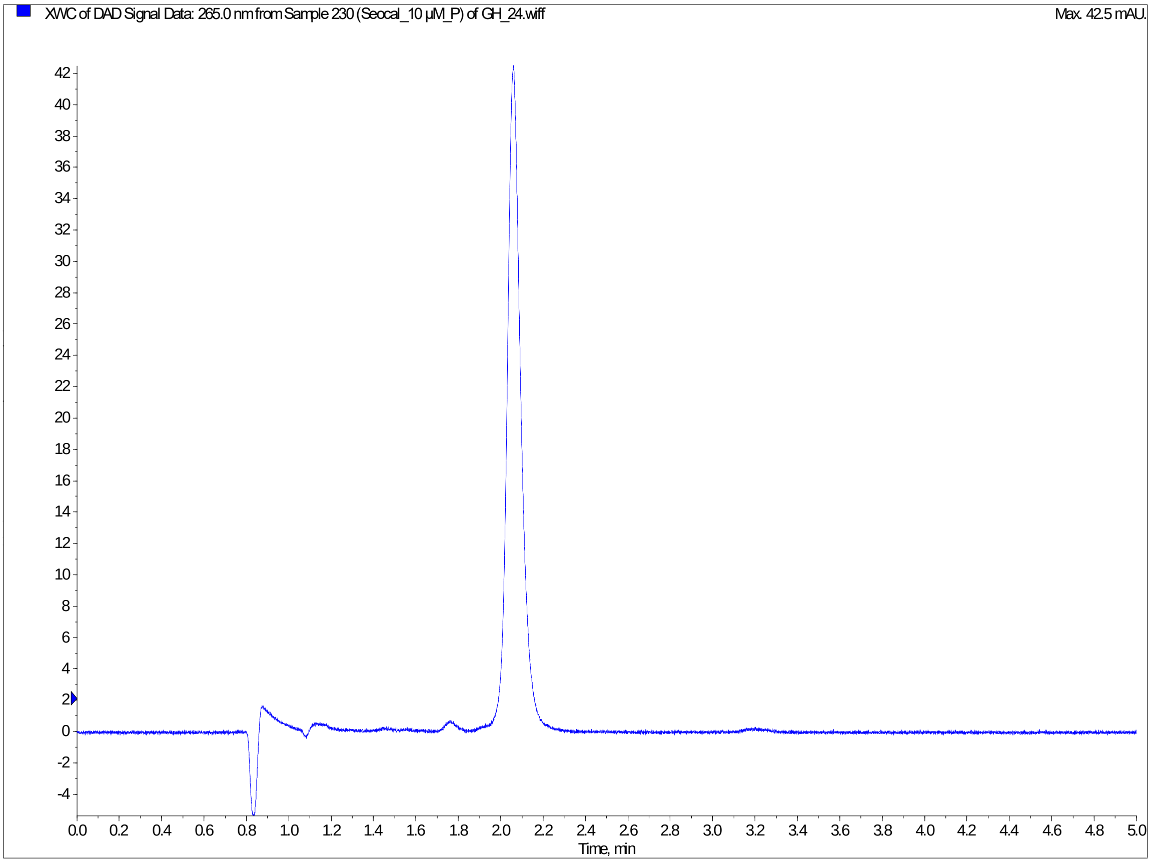

COMPOUND INFORMATION

Biological activity

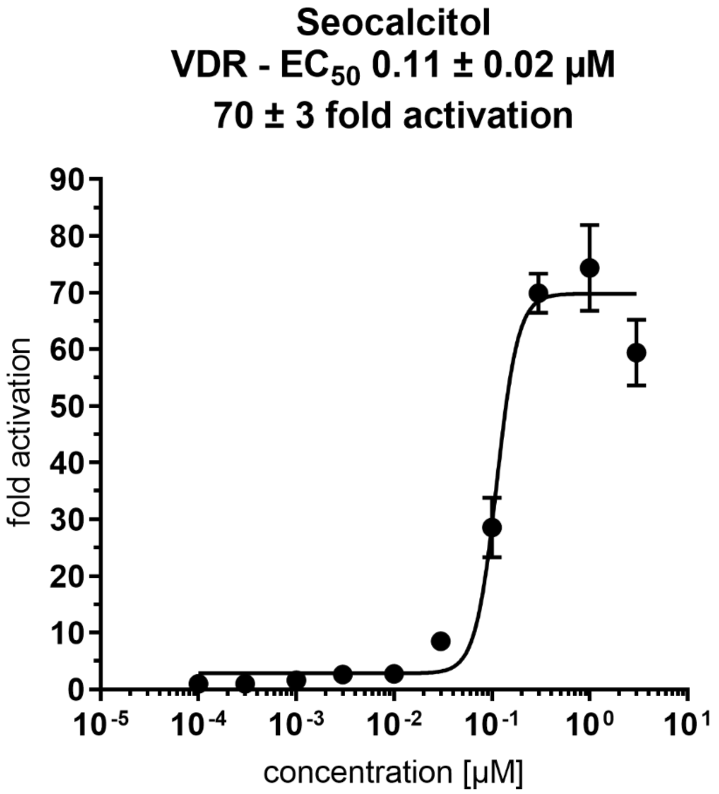

Supplement: Supplementary file 4 — Supplementary Data 1 [file 41467_2024_49493_MOESM4_ESM.zip › Seocalcitol.pdf]
