# Supplementary material for: Chemogenomics for NR1 nuclear hormone receptors
Source: Nat Commun. 2024 Jun 18;15:5201. doi: 10.1038/s41467-024-49493-6 (PMC11189487; doi:10.1038/s41467-024-49493-6)

COMPOUND INFORMATION

Sobetirome

**CAS Registry No.:**	211110-63-3
**Formal Name:**	2-(4-(4-hydroxy-3-isopropylbenzyl)-3,5-dimethylphenoxy)acetic acid
**EUbOPEN ID:**	EUB0000585a
**Molecular Formula:**	$C_{20}H_{24}O_4$
**Molecular Weight:**	328.41 g/mol
**Smiles:**	CC1=CC(=CC(=C1CC2=CC(=C(C=C2)O)C(C)C)C)OCC(=O)O
**Recommended concentration:**	1 µM

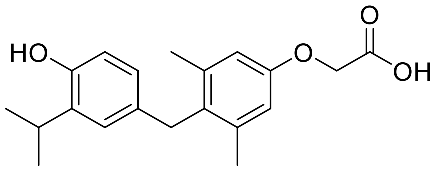

Biological activity

		Type	IC_{50}/EC_{50} [µM]	Reference
Main NR target:	NR1A1 (THRα)	Agonist	0.05	https://doi.org/10.1021/jm0201013
	NR1A2 (THRβ)	Agonist	0.007	
NR off-target:				

COMPOUND INFORMATION

Identity

^{1}H NMR

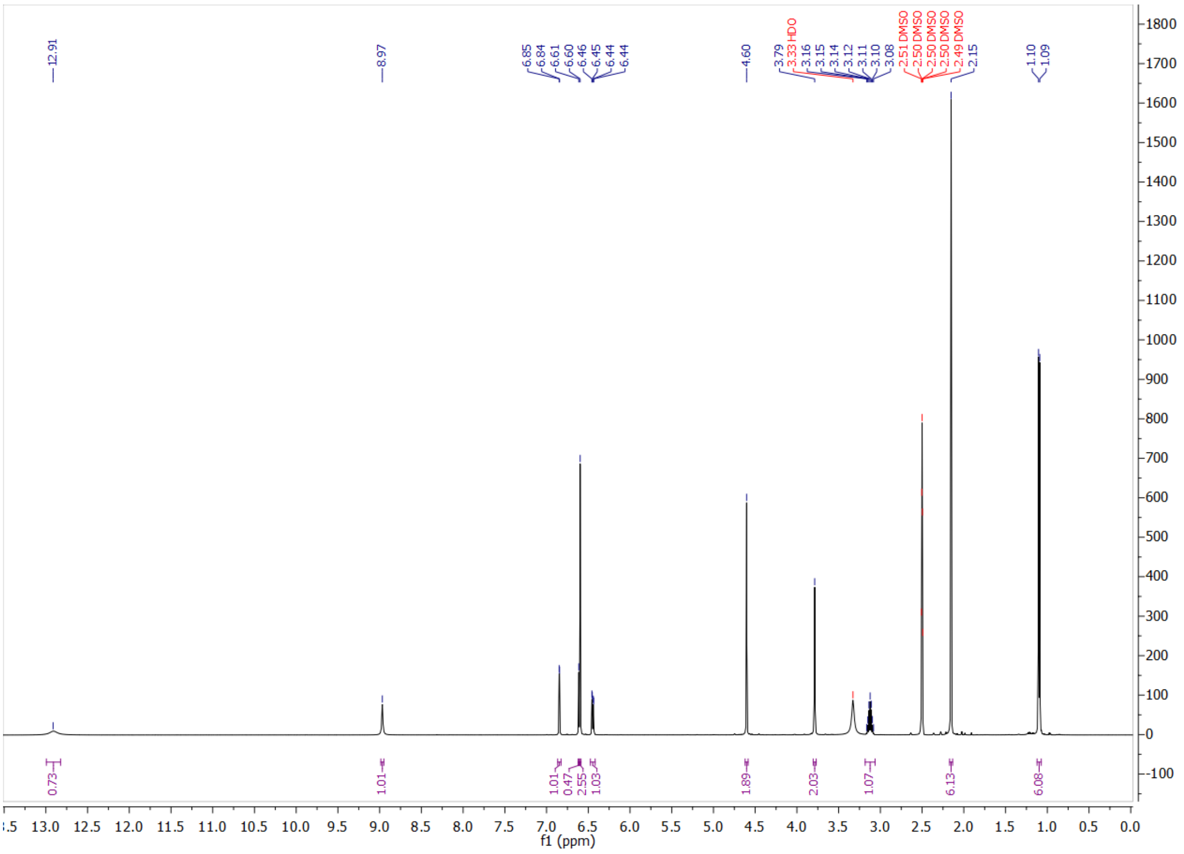

^{13}C NMR

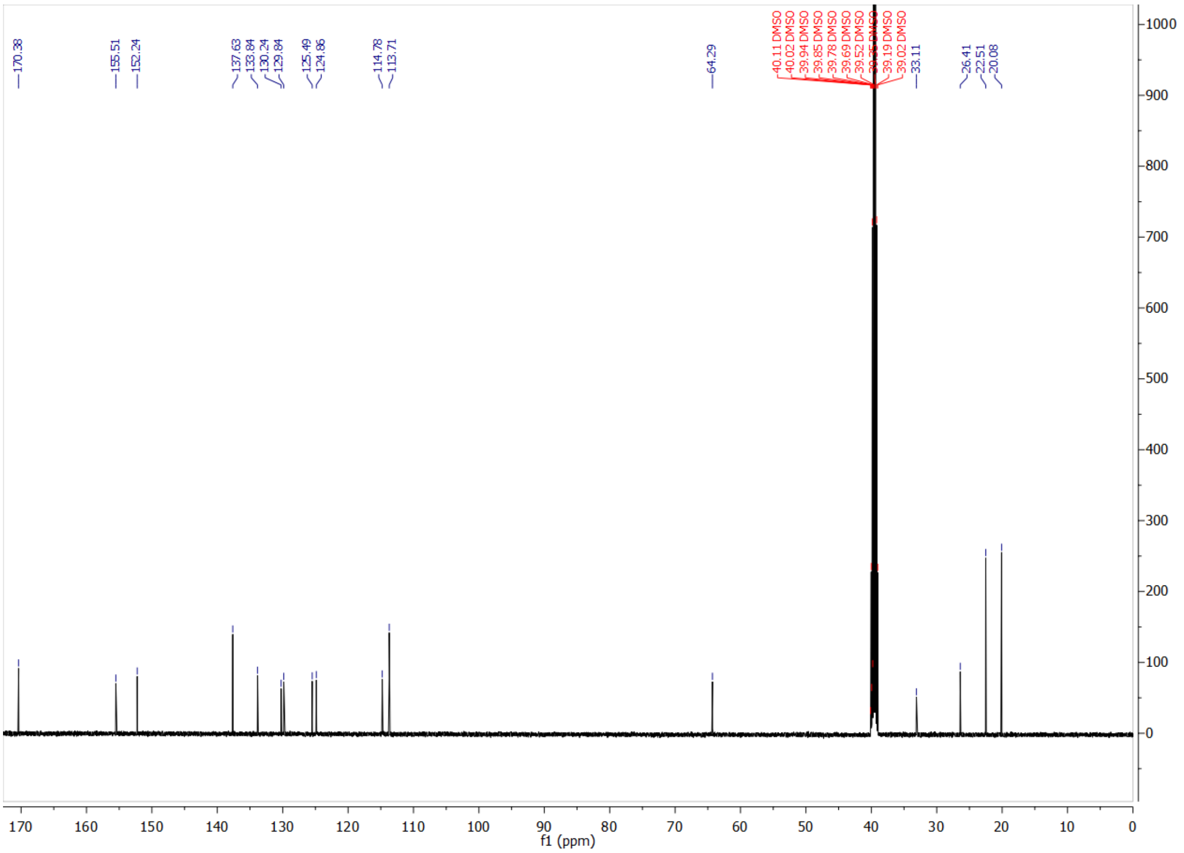

COMPOUND INFORMATION

Purity

M_r 328.41

MS: ESI-negative, *m/z* 327/269 (blue), *m/z* 327/135 (red)

LC: 0.1% HCOOH/ACN (40/60)

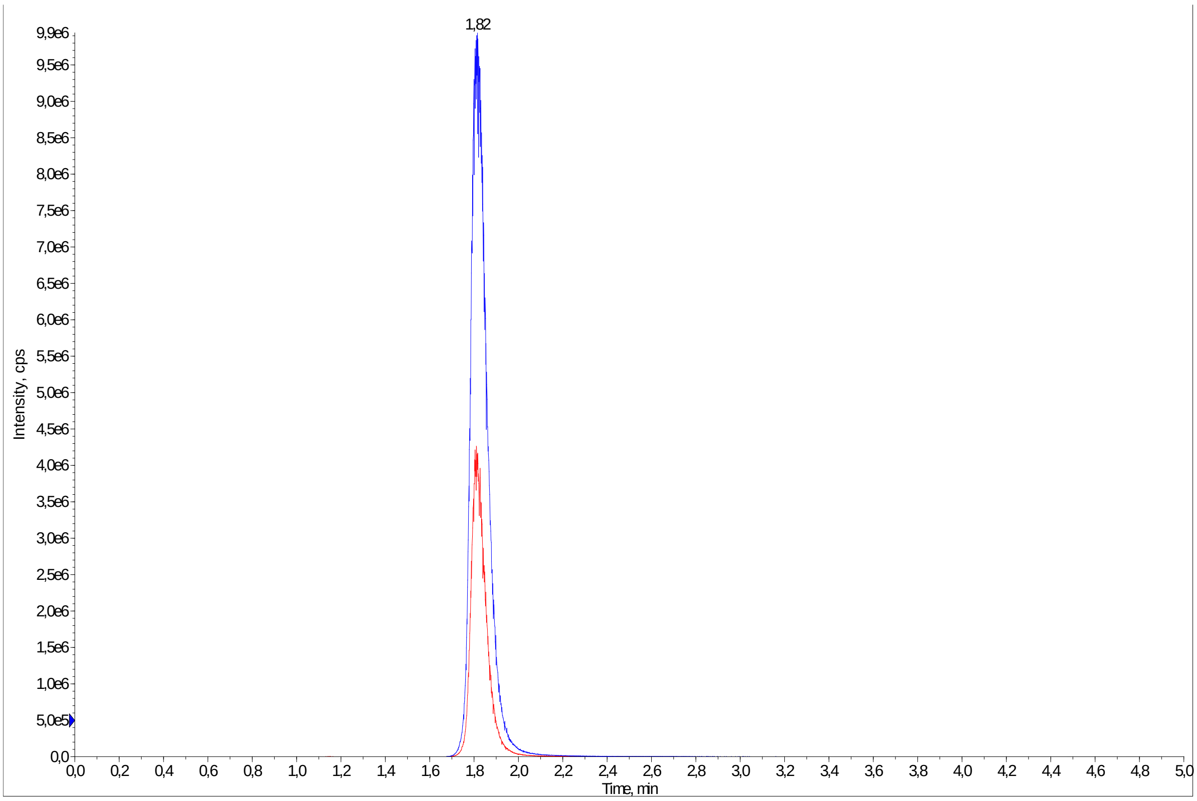

COMPOUND INFORMATION

LC-UV

LC: 0.1% HCOOH/ACN (40/60)

DAD: 210, 230 (XWC), 240, 254, 280 nm

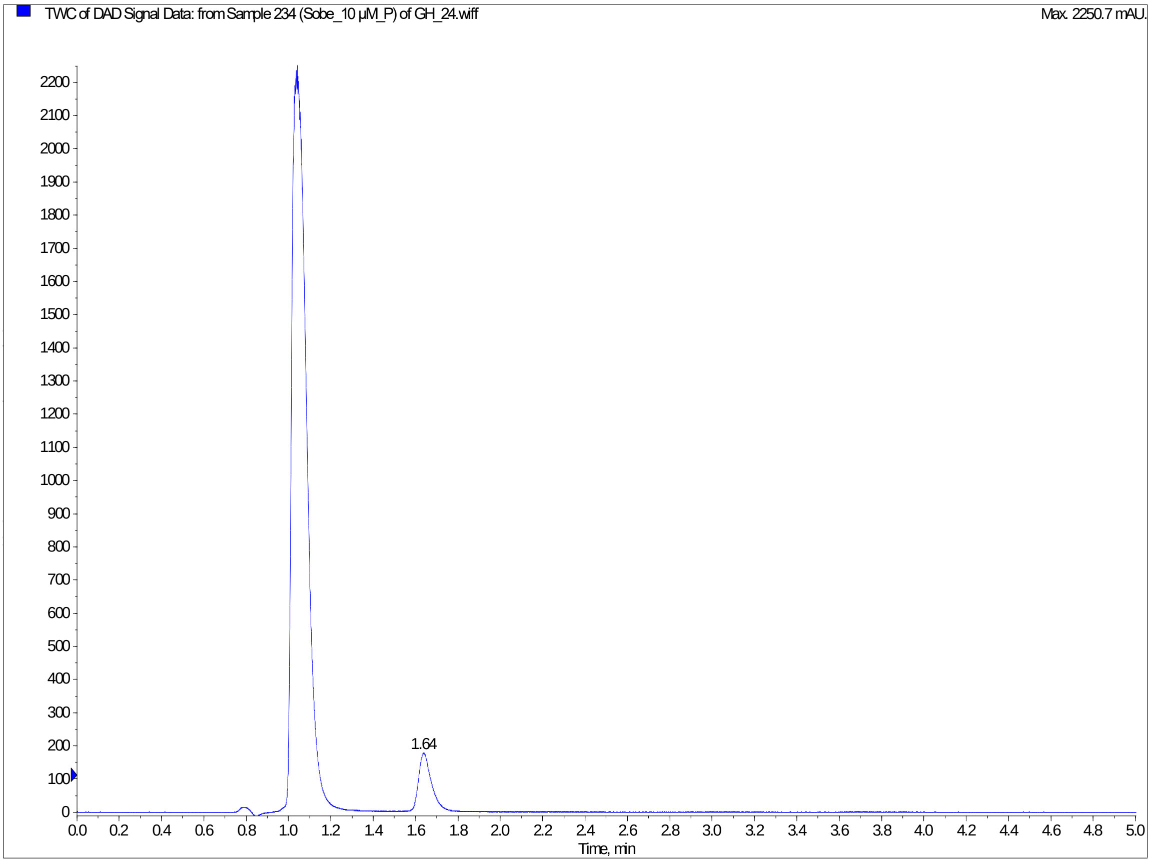

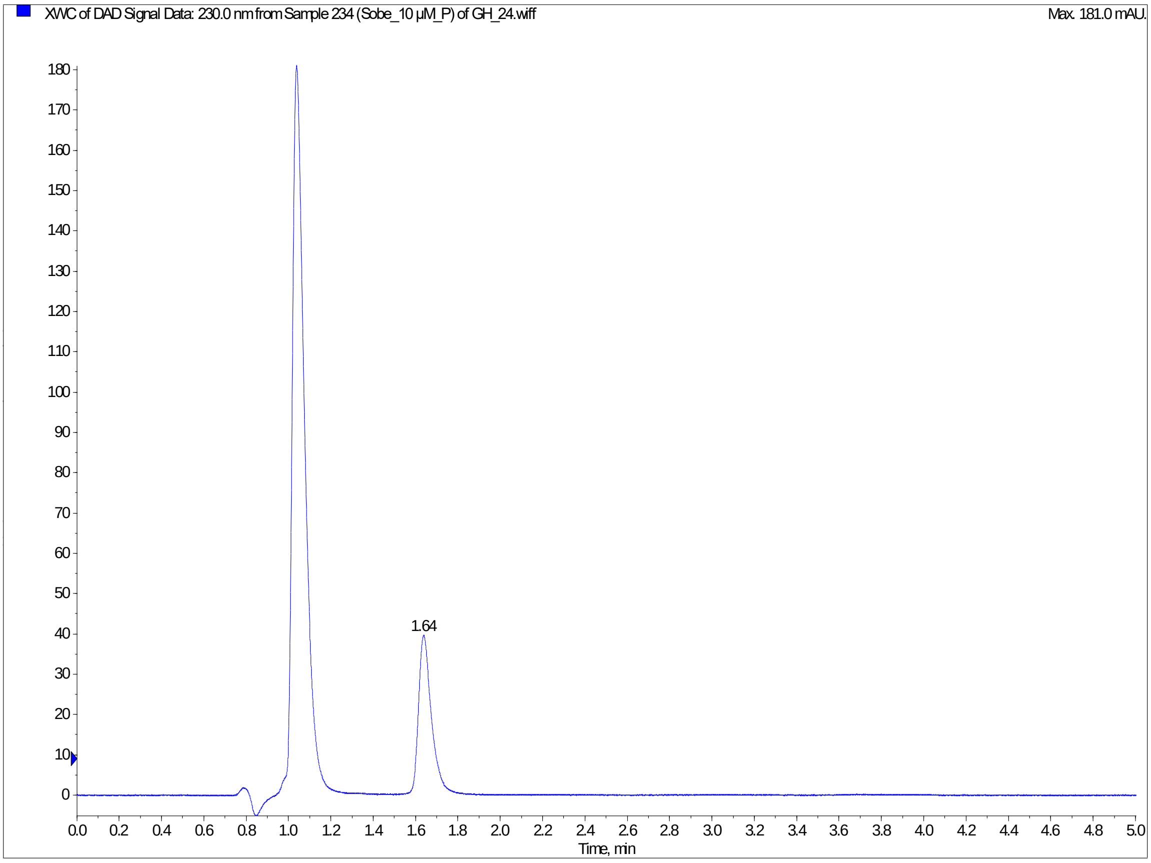

Supplement: Supplementary file 4 — Supplementary Data 1 [file 41467_2024_49493_MOESM4_ESM.zip › Sobetirome.pdf]
