# Supplementary material for: Chemogenomics for NR1 nuclear hormone receptors
Source: Nat Commun. 2024 Jun 18;15:5201. doi: 10.1038/s41467-024-49493-6 (PMC11189487; doi:10.1038/s41467-024-49493-6)

COMPOUND INFORMATION

SR1001

**CAS Registry No.:**	1335106-03-0
**Formal Name:**	N-(5-(N-(4-(1,1,1,3,3,3-hexafluoro-2-hydroxypropan-2-yl)phenyl)sulfamoyl)-4-methylthiazol-2-yl)acetamide
**EUBOPEN ID:**	EUB0001158a
**Molecular Formula:**	$C_{15}H_{13}F_6N_3O_4S_2$
**Molecular Weight:**	477.40 g/mol
**Smiles:**	CC(NC1=NC(C)=C(S1)S(NC2=CC=C(C=C2)C(C(F)(F)F)(C(F)(F)F)O)(=O)=O)=O
**Recommended concentration:**	1 µM

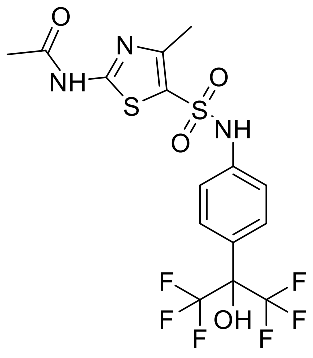

Biological activity

		Type	IC_{50}/EC_{50} [µM]	Reference
Main NR target:	NR1F1 (RORα)	inv. Agonist	0.9	inhouse
	NR1F2 (RORβ)	inv. Agonist	1.1	inhouse
	NR1F3 (RORγ)	inv. Agonist	0.11	inhouse
NR off-target:				

COMPOUND INFORMATION

Identity

^{1}H NMR

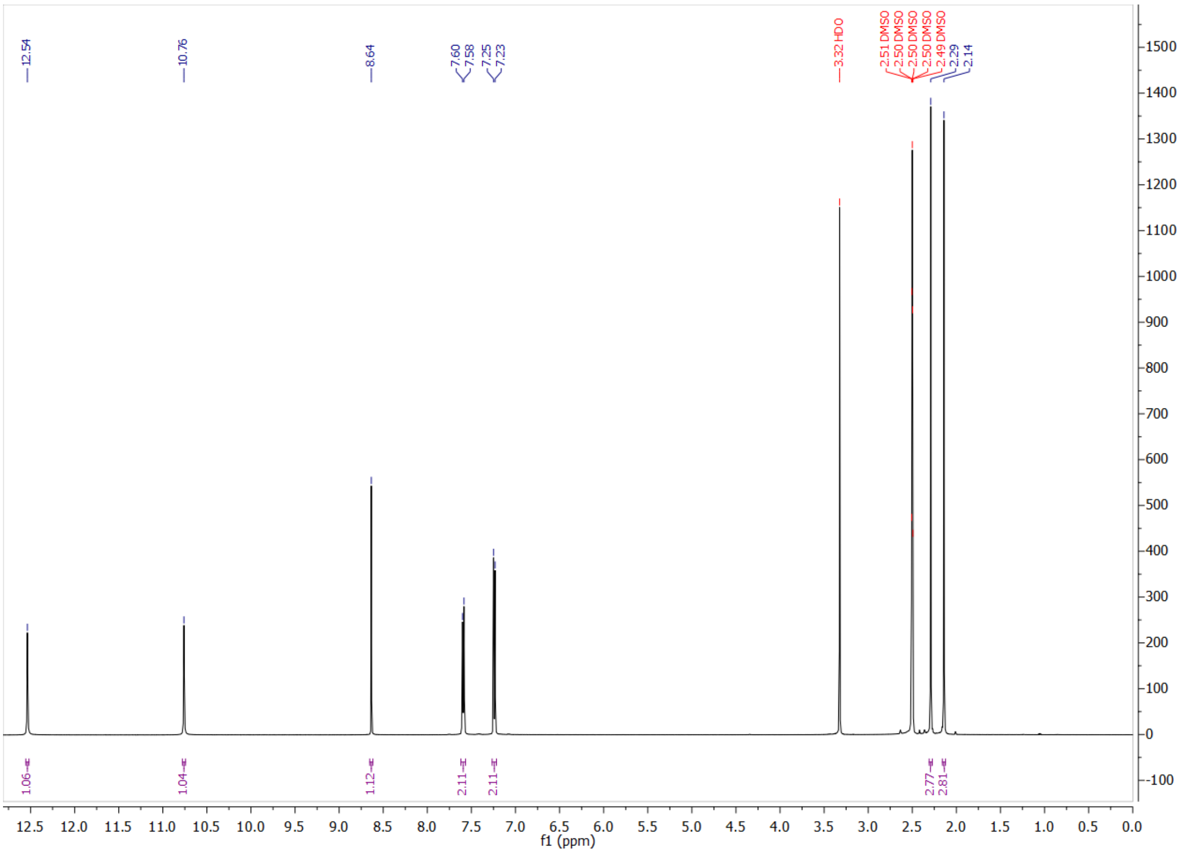

^{13}C NMR

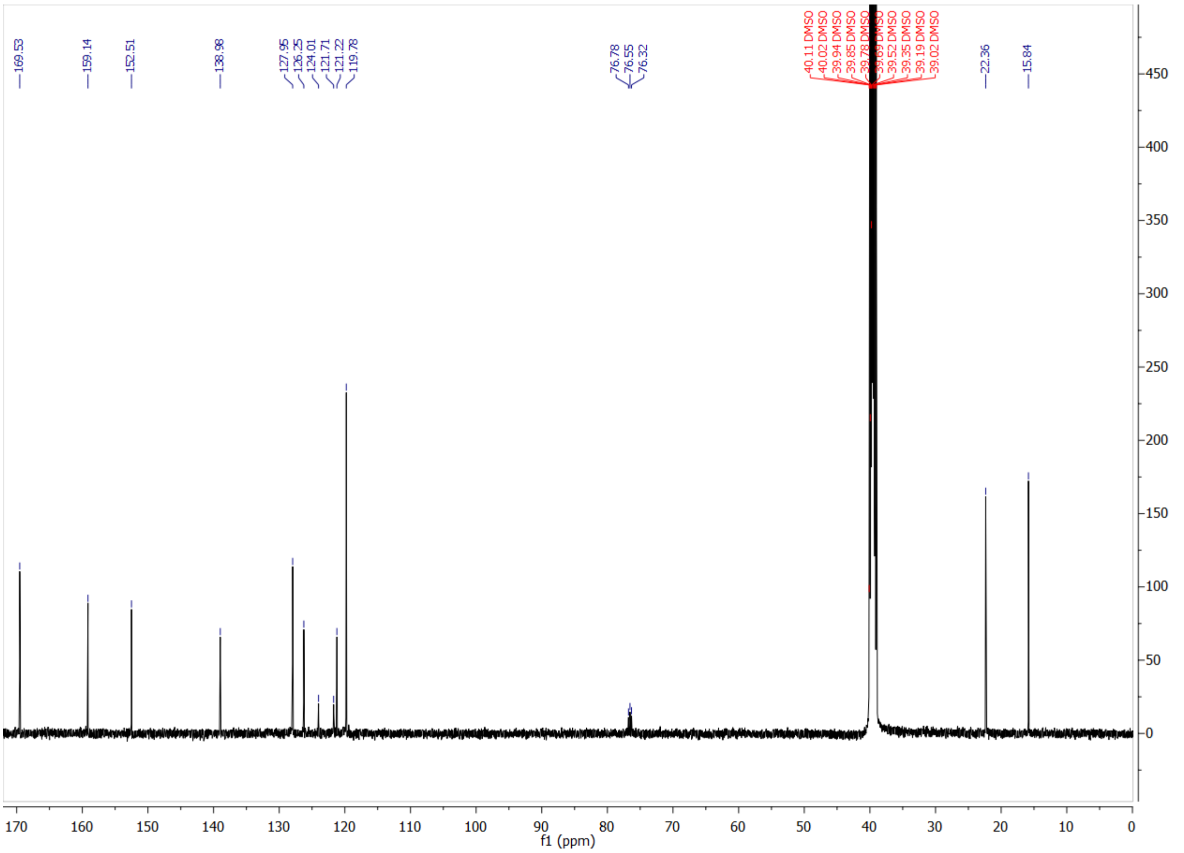

COMPOUND INFORMATION

Purity

```
Data File W:\analyti...OPEN\CGC_wave3_1_FirstPassB 2023-01-04 18-28-02\079-D2F-G4-SR1001.D
Sample Name: SR1001

    =====================================================================
    Acq. Operator   : SYSTEM                          Seq. Line :  79
    Sample Operator : SYSTEM
    Acq. Instrument : LCMS test                        Location :   D2F-G4
    Injection Date  : 1/5/2023 8:53:21 AM                   Inj :   1
                                                     Inj Volume : Inj prog
    Sequence File   : W:\analytical_LCMS_DATA\EUbOPEN\CGC_wave3_1_FirstPassB 2023-01-04 18-28-02
                      \CGC_wave3_1_FirstPassB.S
    Method          : W:\analytical_LCMS_DATA\EUbOPEN\CGC_wave3_1_FirstPassB 2023-01-04 18-28-02
                      \CGL_FIRSTPASS_GENERALMETHOD_VIAL1+2_20210319.M (Sequence Method)
    Last changed    : 1/25/2022 4:36:18 PM by SYSTEM
    Method Info     : CGL wellplate, 0.5 uL of 10 mM DMSO, general method
```

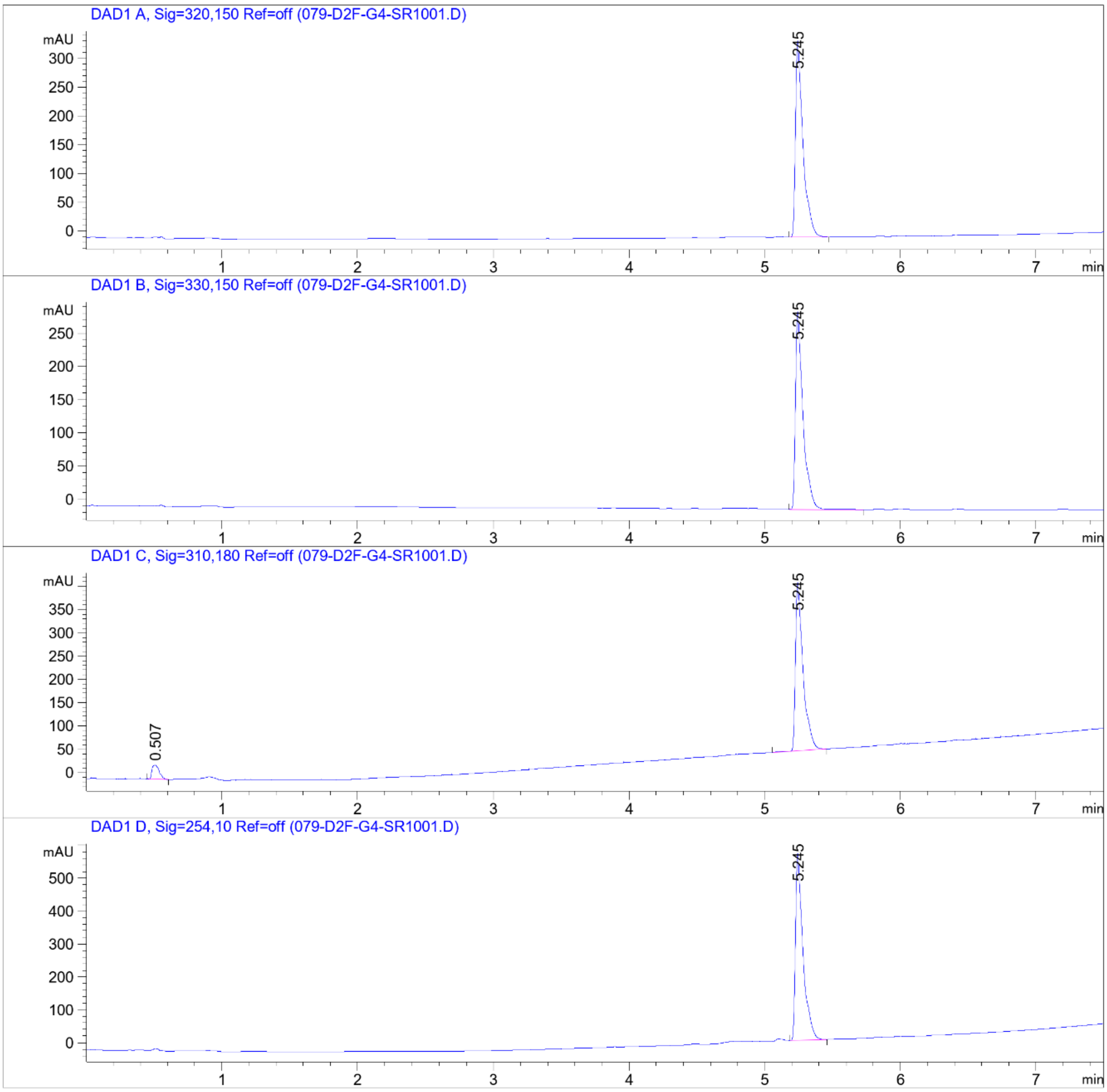

COMPOUND INFORMATION

Data File W:\analyti...OPEN\CGC_wave3_1_FirstPassB 2023-01-04 18-28-02\079-D2F-G4-SR1001.D
Sample Name: SR1001

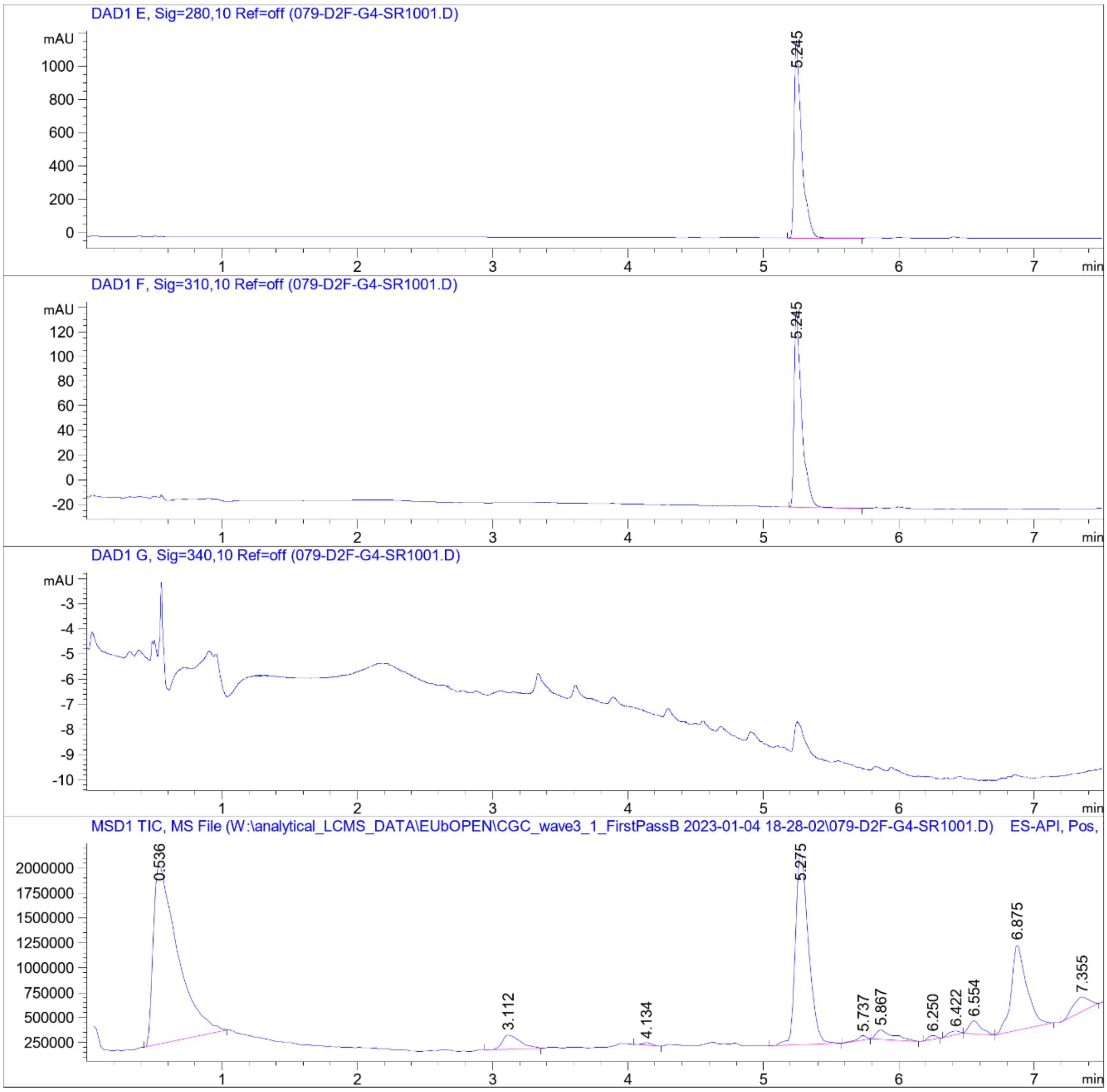

COMPOUND INFORMATION

Data File W:\analyti...OPEN\CGC_wave3_1_FirstPassB 2023-01-04 18-28-02\079-D2F-G4-SR1001.D
Sample Name: SR1001

MS Signal: MSD1 TIC, MS File, ES-API, Pos, Scan, Frag: 70, "POS Scan"
Spectra from peak tops.
Noise Cutoff: 1000 counts.
Reportable Ion Abundance: > 50%.
LC Signal: DAD1 A, Sig=320,150 Ref=off
Peak matching window: 0.1 min

Retention Time (LC)	LC Area	Retention Time (MS)	MS Area	Mol. Weight or Ion
-	-	0.536	22368776	157.10 I
-	-	3.112	1362420	239.00 I
				217.10 I
-	-	4.134	111615	510.40 I
				258.10 I
				170.80 I
5.245	1299	5.275	12884487	478.00 I
-	-	5.737	173390	280.20 I
-	-	5.867	851386	318.20 I
				296.20 I
-	-	6.250	161489	228.20 I
				137.20 I
-	-	6.422	219314	350.20 I
				282.20 I
				254.20 I
				137.10 I
-	-	6.554	787709	507.30 I
				485.30 I
				280.20 I
-	-	6.875	6840838	282.20 I
-	-	7.355	1244306	400.30 I
				282.20 I

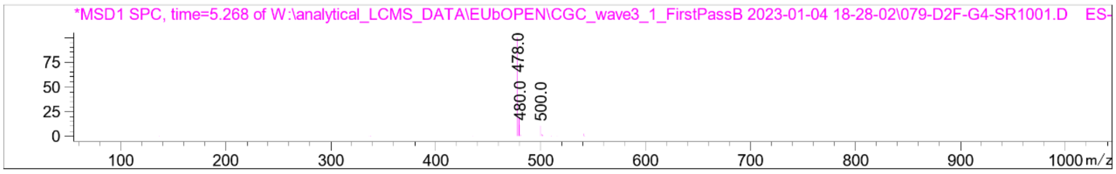

COMPOUND INFORMATION

Biological activity

SR1001
RORα - IC_{50} 0.9 ± 0.2 µM
0.26 ± 0.05 rem. activity

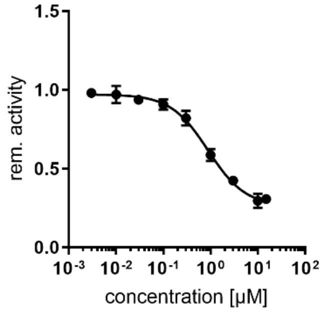

SR1001
RORβ - IC_{50} 1.1 ± 0.5 µM
0.1 ± 0.2 rem. activity

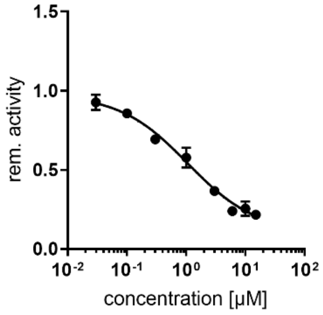

SR1001
RORγ - IC_{50} 0.11 ± 0.02 µM
0.00 ± 0.04 rem. activity

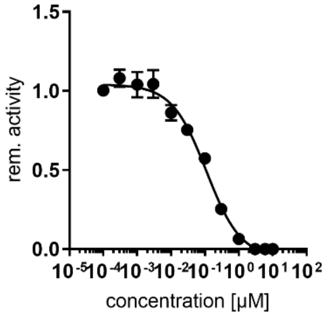

Supplement: Supplementary file 4 — Supplementary Data 1 [file 41467_2024_49493_MOESM4_ESM.zip › SR1001.pdf]
