# Supplementary material for: Chemogenomics for NR1 nuclear hormone receptors
Source: Nat Commun. 2024 Jun 18;15:5201. doi: 10.1038/s41467-024-49493-6 (PMC11189487; doi:10.1038/s41467-024-49493-6)

COMPOUND INFORMATION

SR1078

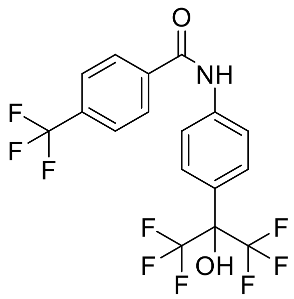

**CAS Registry No.:**	1246525-60-9
**Formal Name:**	N-(4-(1,1,1,3,3,3-hexafluoro-2-hydroxypropan-2-yl)phenyl)-4-(trifluoromethyl)benzamide
**EUBOPEN ID:**	EUB0001156a
**Molecular Formula:**	$C_{17}H_{10}F_9NO_2$
**Molecular Weight:**	431.26 g/mol
**Smiles:**	O=C(C1=CC=C(C=C1)C(F)(F)F)NC2=CC=C(C=C2)C(C(F)(F)F)(C(F)(F)F)O
**Recommended concentration:**	1 µM

Biological activity

		Type	IC_{50}/EC_{50} [µM]	Reference
Main NR target:	NR1F3 (RORγ)	inv. Agonist	2	https://doi.org/10.1021/cb100223d
NR off-target:				

COMPOUND INFORMATION

Identity

^{1}H NMR

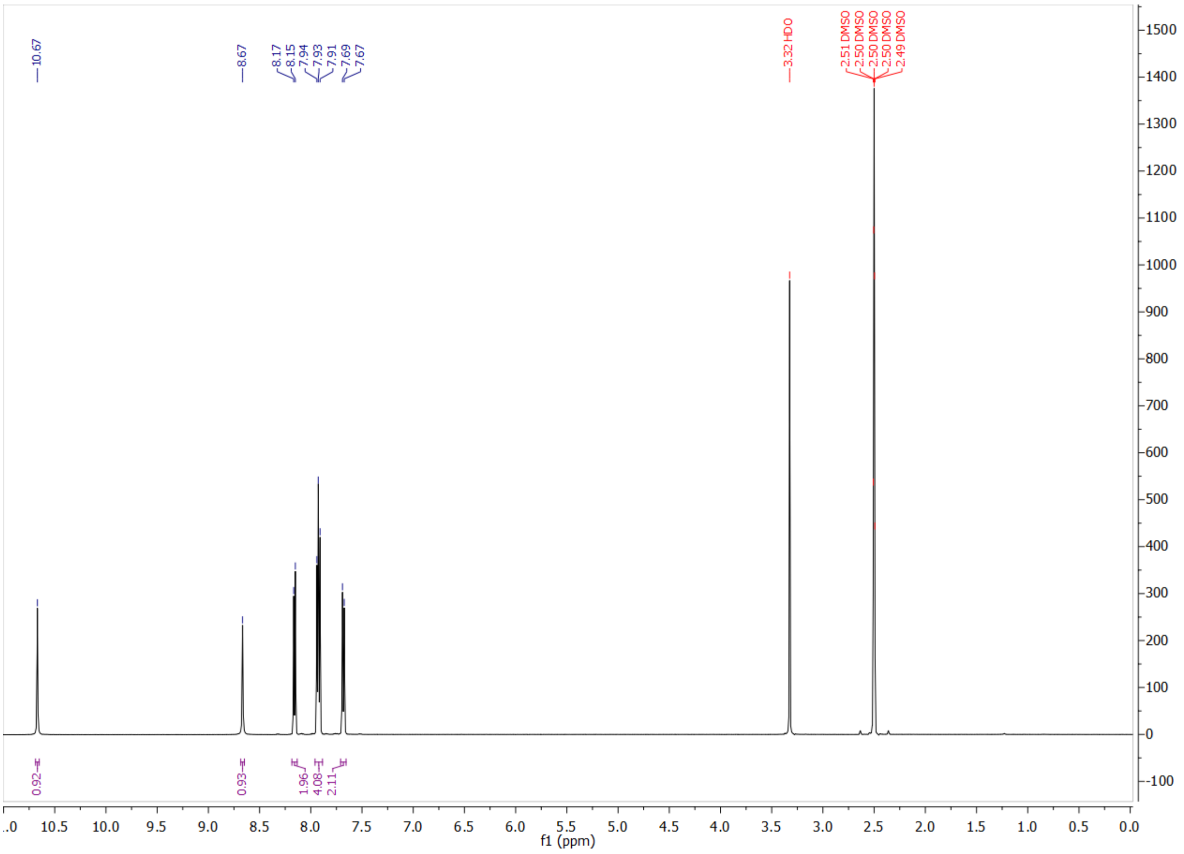

^{13}C NMR

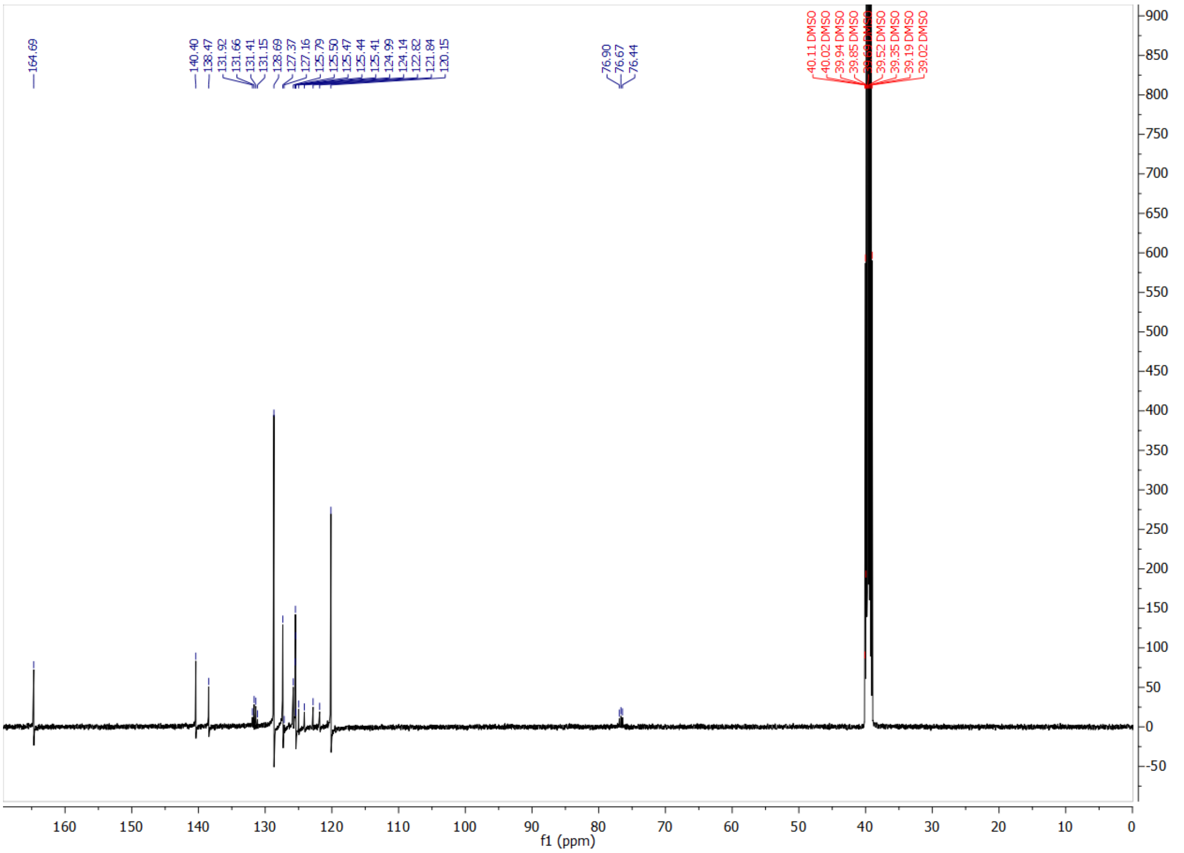

COMPOUND INFORMATION

Purity

```
Data File W:\analyti...OPEN\CGC_wave3_1_FirstPassB 2023-01-04 18-28-02\077-D2F-G2-SR1078.D
Sample Name: SR1078

    =====================================================================
    Acq. Operator   : SYSTEM                        Seq. Line :  77
    Sample Operator : SYSTEM
    Acq. Instrument : LCMS test                      Location :  D2F-G2
    Injection Date  : 1/5/2023 8:31:14 AM                 Inj :  1
                                                   Inj Volume : Inj prog
    Sequence File   : W:\analytical_LCMS_DATA\EUbOPEN\CGC_wave3_1_FirstPassB 2023-01-04 18-28-02
                      \CGC_wave3_1_FirstPassB.S
    Method          : W:\analytical_LCMS_DATA\EUbOPEN\CGC_wave3_1_FirstPassB 2023-01-04 18-28-02
                      \CGL_FIRSTPASS_GENERALMETHOD_VIAL1+2_20210319.M (Sequence Method)
    Last changed    : 1/25/2022 4:36:18 PM by SYSTEM
    Method Info     : CGL wellplate, 0.5 uL of 10 mM DMSO, general method
```

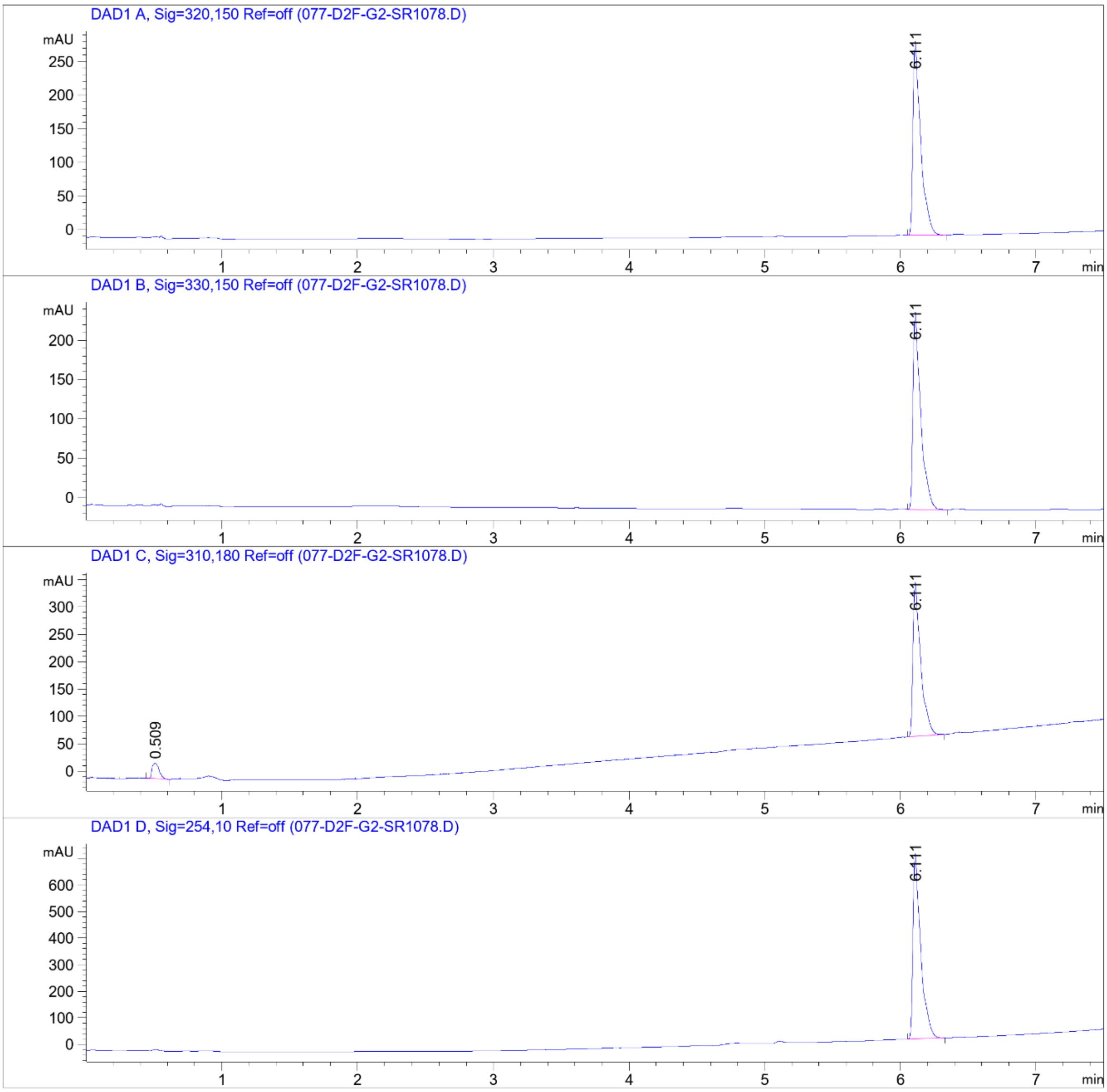

COMPOUND INFORMATION

Data File W:\analyti...OPEN\CGC_wave3_1_FirstPassB 2023-01-04 18-28-02\077-D2F-G2-SR1078.D
Sample Name: SR1078

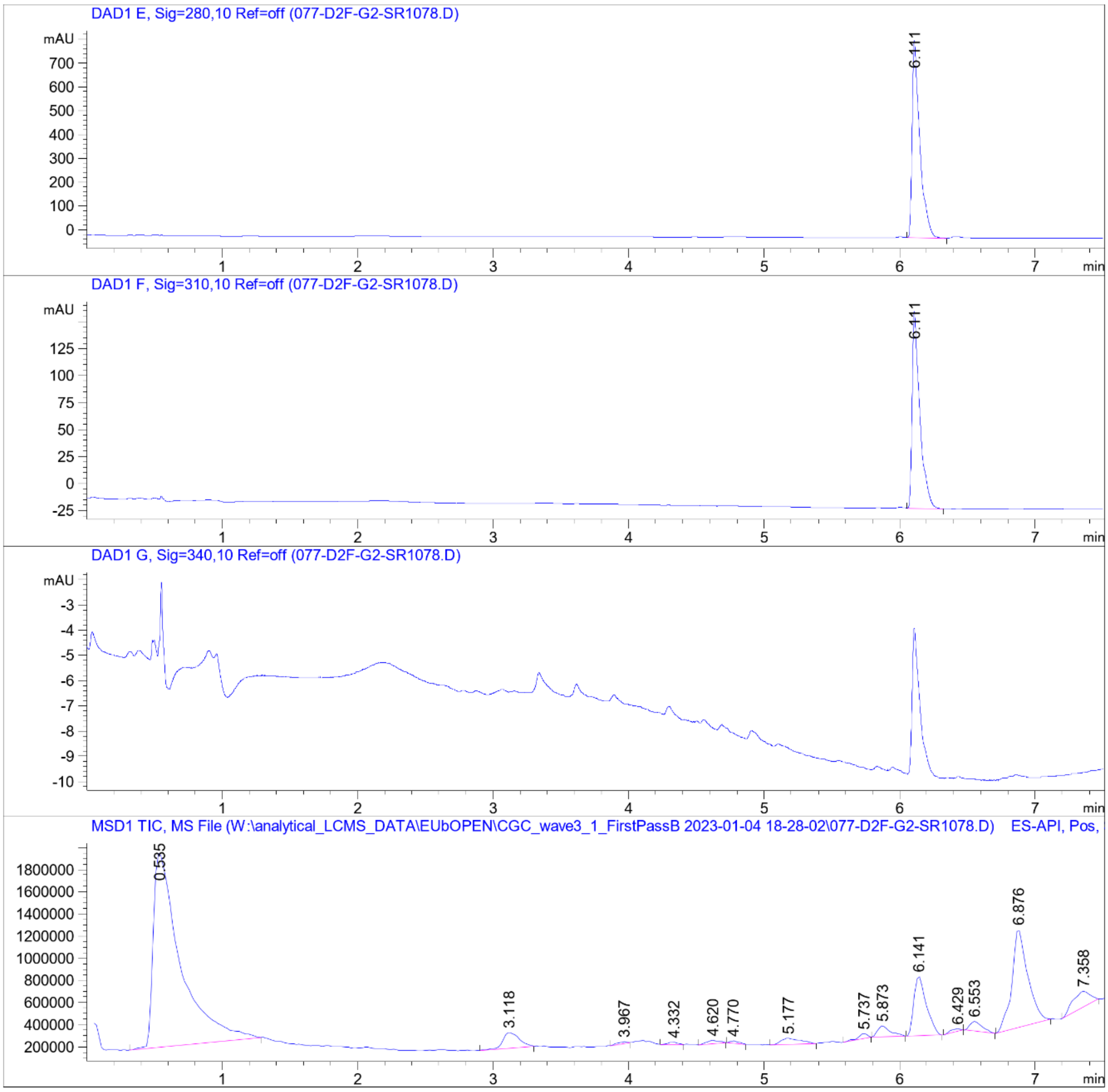

COMPOUND INFORMATION

Data File W:\analyti...OPEN\CGC_wave3_1_FirstPassB 2023-01-04 18-28-02\077-D2F-G2-SR1078.D
Sample Name: SR1078

MS Signal: MSD1 TIC, MS File, ES-API, Pos, Scan, Frag: 70, "POS Scan"
Spectra from peak tops.
Noise Cutoff: 1000 counts.
Reportable Ion Abundance: > 50%.
LC Signal: DAD1 A, Sig=320,150 Ref=off
Peak matching window: 0.1 min

Retention Time (LC)	LC Area	Retention Time (MS)	MS Area	Mol. Weight or Ion
-	-	0.535	24433608	157.00 I
-	-	3.118	1196035	239.00 I
				217.10 I
-	-	3.967	94859	216.10 I
				200.00 I
				170.90 I
-	-	4.332	98138	510.40 I
				170.90 I
-	-	4.620	184336	510.30 I
				170.90 I
				158.20 I
				137.10 I
-	-	4.770	102449	510.30 I
				279.10 I
				170.80 I
				137.10 I
-	-	5.177	552506	510.30 I
				338.20 I
				170.80 I
				137.10 I
-	-	5.737	179543	280.20 I
-	-	5.873	625801	318.20 I
				296.20 I
6.111	1138	6.141	3386224	432.00 I
-	-	6.429	134421	350.20 I
				282.20 I
				254.20 I
				137.20 I
-	-	6.553	539122	507.30 I
				280.20 I
-	-	6.876	7225335	282.30 I
-	-	7.358	1309224	400.30 I
				282.20 I

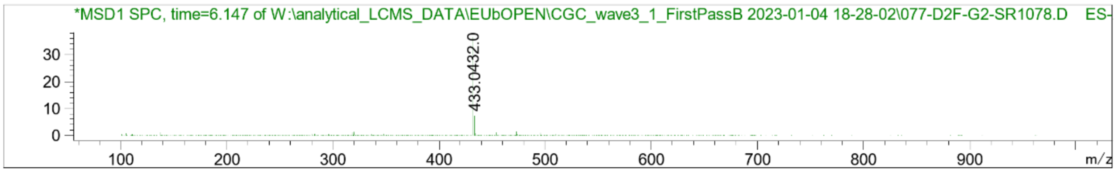

Supplement: Supplementary file 4 — Supplementary Data 1 [file 41467_2024_49493_MOESM4_ESM.zip › SR1078.pdf]
