# Supplementary material for: Chemogenomics for NR1 nuclear hormone receptors
Source: Nat Commun. 2024 Jun 18;15:5201. doi: 10.1038/s41467-024-49493-6 (PMC11189487; doi:10.1038/s41467-024-49493-6)

COMPOUND INFORMATION

SR12813

**CAS Registry No.:**	126411-39-0
**Formal Name:**	Tetraethyl (2-(3,5-di-tert-butyl-4-hydroxyphenyl)ethene-1,1-diyl)bis(phosphonate)
**EUBOPEN ID:**	EUB0000576a
**Molecular Formula:**	$C_{24}H_{42}O_7P_2$
**Molecular Weight:**	504.54 g/mol
**Smiles:**	CCOP(/C(P(OCC)(OCC)=O)=C/C1=CC(C(C)(C)C)=C(O)C(C(C)(C)C)=C1)(OCC)=O
**Recommended concentration:**	1 µM

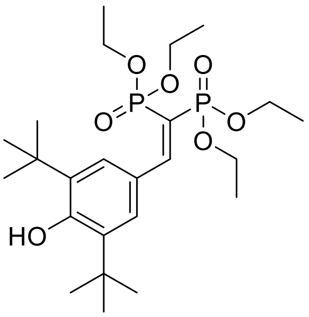

Biological activity

		Type	IC_{50}/EC_{50} [µM]	Reference
Main NR target:	NR1I2 (PXR)	Agonist	0.14	https://doi.org/10.1124/mol.106.033415
NR off-target:				

COMPOUND INFORMATION

Identity

^{1}H NMR

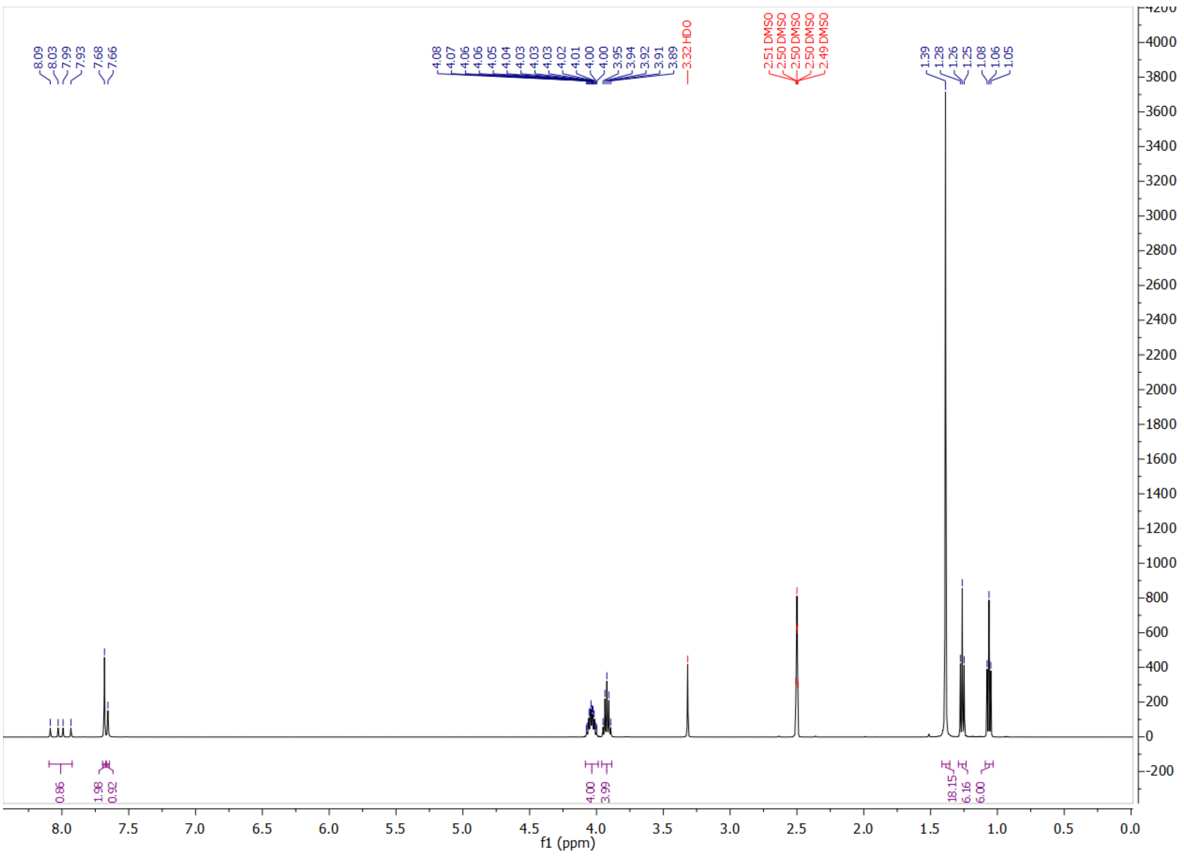

^{13}C NMR

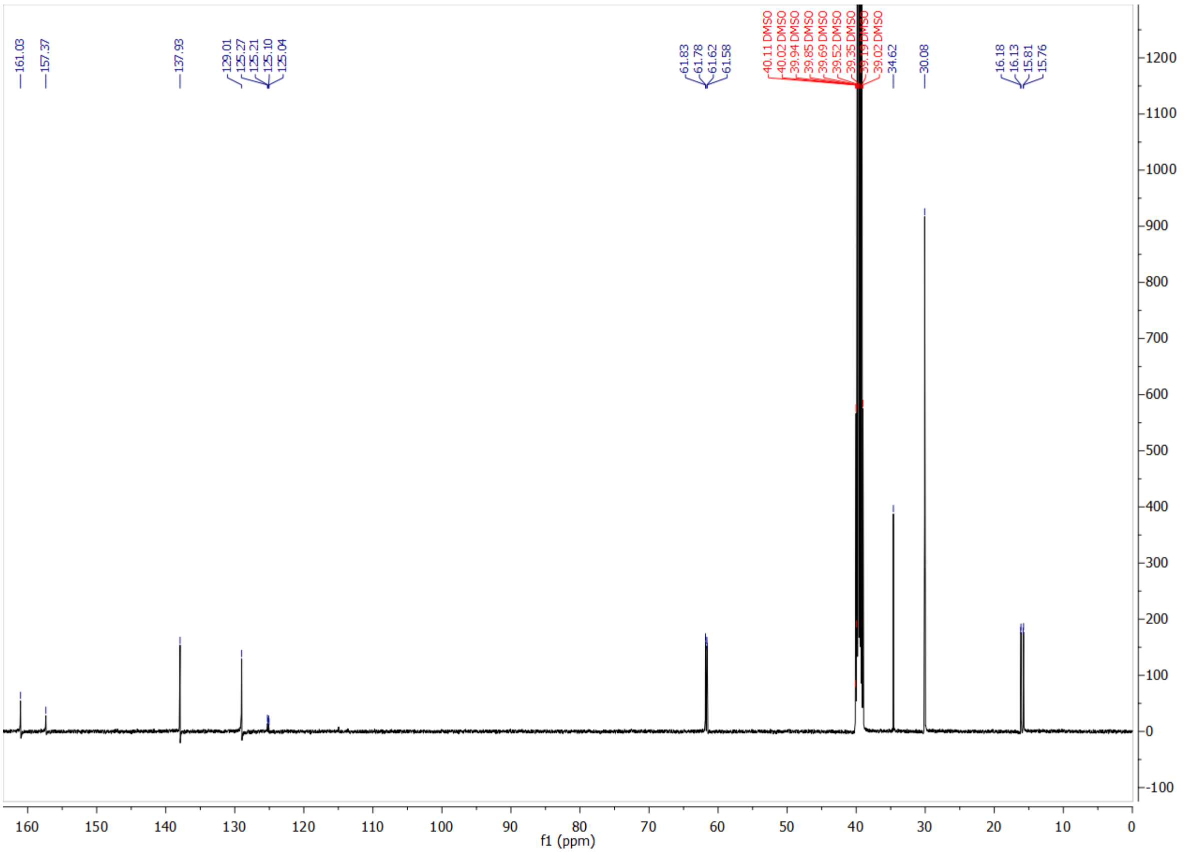

COMPOUND INFORMATION

Purity

Data File W:\analyti...PEN\CGC_wave3_2_FirstPass 2023-01-05 22-10-41\035-D2F-C7-SR-12813.D
Sample Name: SR-12813

```
=====================================================================
Acq. Operator   : SYSTEM                          Seq. Line :  35
Sample Operator : SYSTEM
Acq. Instrument : LCMS test                        Location :  D2F-C7
Injection Date  : 1/6/2023 4:29:43 AM                   Inj :  1
                                               Inj Volume : Inj prog
Sequence File   : W:\analytical_LCMS_DATA\EUbOPEN\CGC_wave3_2_FirstPass 2023-01-05 22-10-41
                  \CGC_wave3_2_FirstPass.S
Method          : W:\analytical_LCMS_DATA\EUbOPEN\CGC_wave3_2_FirstPass 2023-01-05 22-10-41
                  \CGL_FIRSTPASS_GENERALMETHOD_VIAL1+2_20210319.M (Sequence Method)
Last changed    : 1/25/2022 4:36:18 PM by SYSTEM
Method Info     : CGL wellplate, 0.5 uL of 10 mM DMSO, general method
```

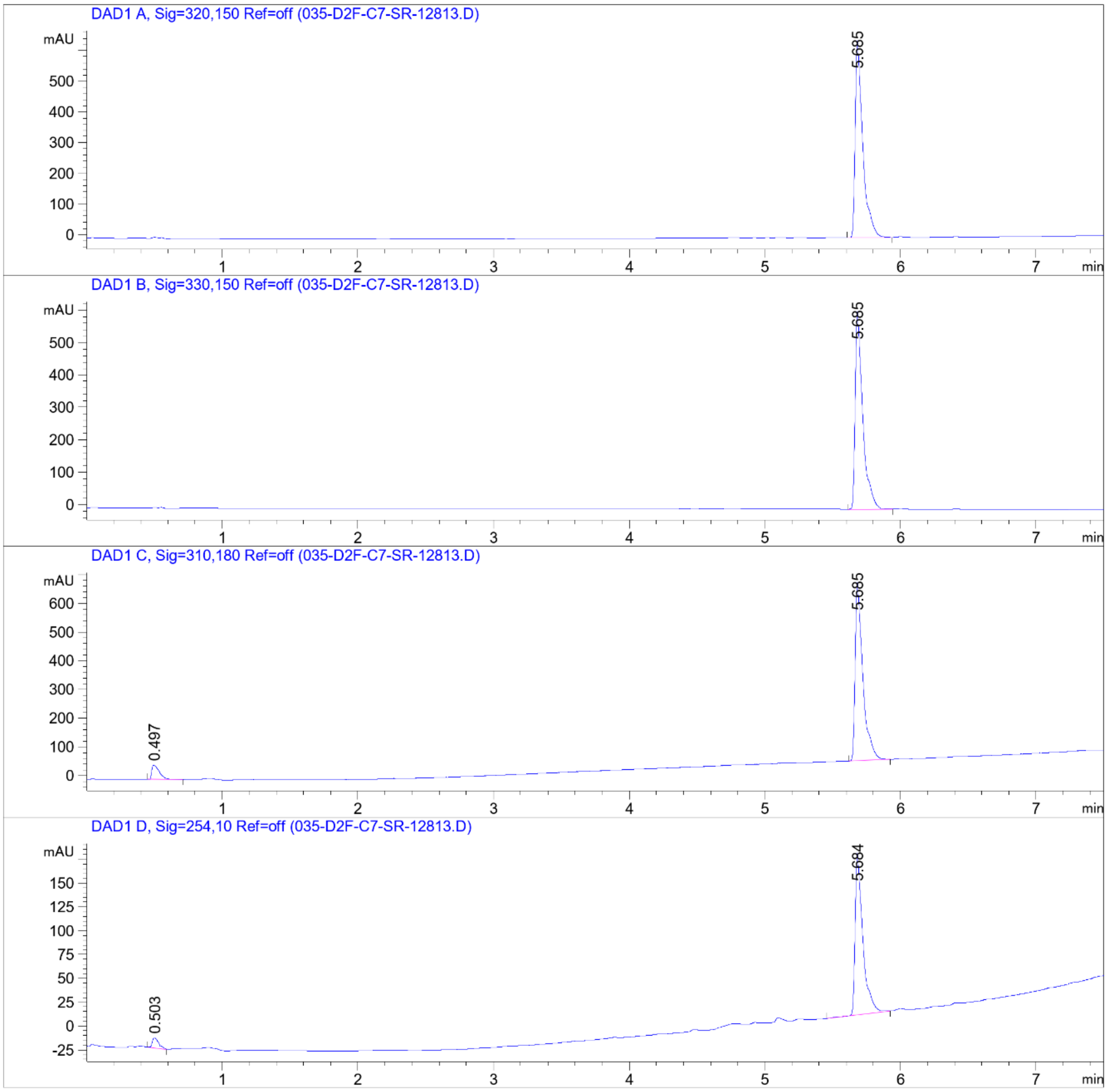

COMPOUND INFORMATION

Data File W:\analyti...PEN\CGC_wave3_2_FirstPass 2023-01-05 22-10-41\035-D2F-C7-SR-12813.D
Sample Name: SR-12813

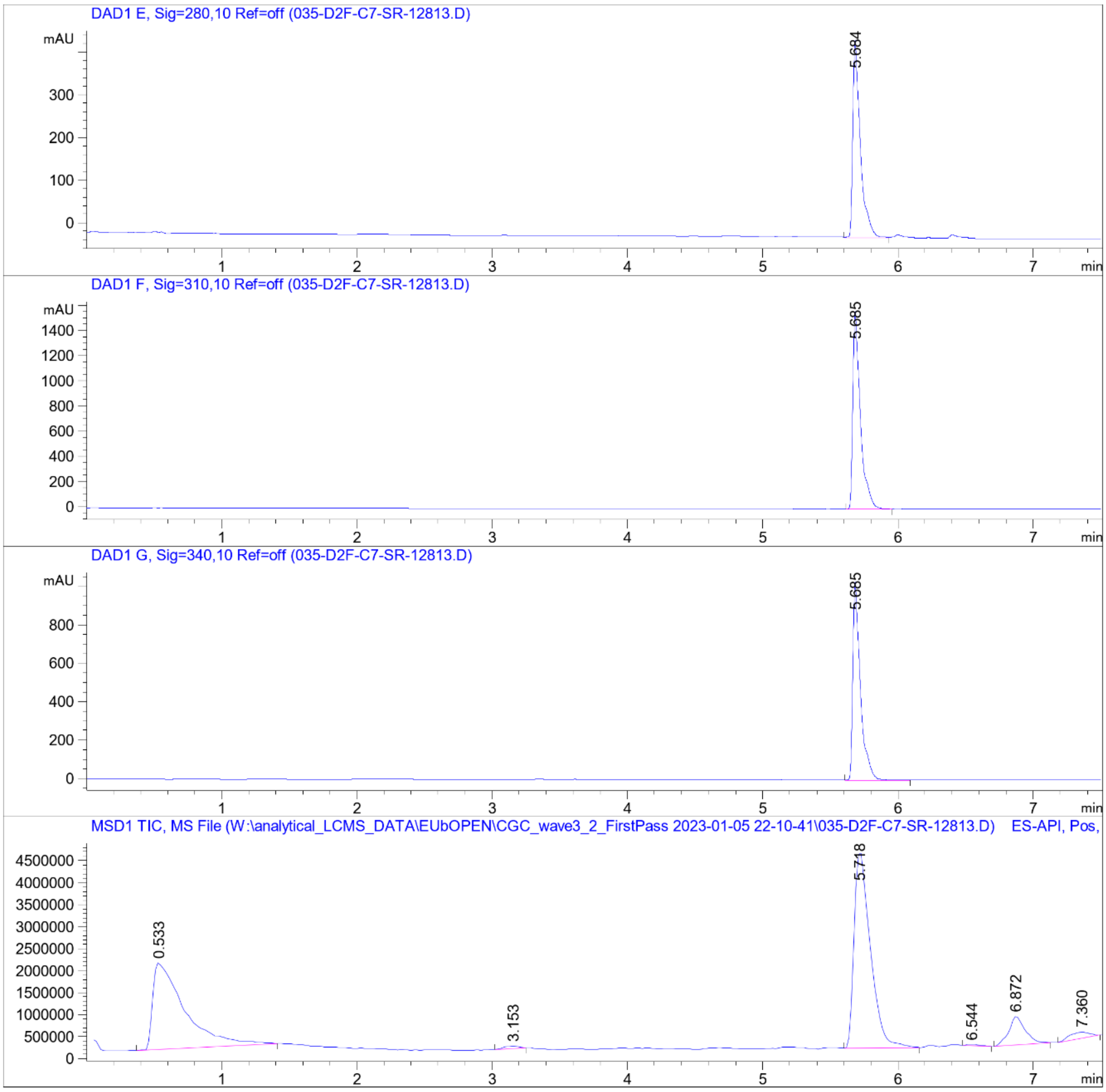

COMPOUND INFORMATION

Data File W:\analyti...PEN\CGC_wave3_2_FirstPass 2023-01-05 22-10-41\035-D2F-C7-SR-12813.D
Sample Name: SR-12813

MS Signal: MSD1 TIC, MS File, ES-API, Pos, Scan, Frag: 70, "POS Scan"
Spectra from peak tops.
Noise Cutoff: 1000 counts.
Reportable Ion Abundance: > 50%.
LC Signal: DAD1 A, Sig=320,150 Ref=off
Peak matching window: 0.1 min

Retention Time (LC)	LC Area	Retention Time (MS)	MS Area	Mol. Weight or Ion
-	-	0.533	32099910	157.00 I
-	-	3.153	457253	188.10 I<br>170.10 I
5.685	2483	5.718	37039984	505.30 I
-	-	6.544	209209	507.30 I<br>280.20 I
-	-	6.872	5459114	282.20 I
-	-	7.360	1514067	400.30 I<br>282.20 I

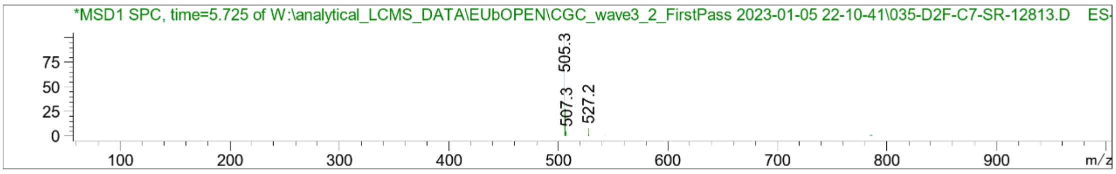

Supplement: Supplementary file 4 — Supplementary Data 1 [file 41467_2024_49493_MOESM4_ESM.zip › SR12813.pdf]
