# Supplementary material for: Chemogenomics for NR1 nuclear hormone receptors
Source: Nat Commun. 2024 Jun 18;15:5201. doi: 10.1038/s41467-024-49493-6 (PMC11189487; doi:10.1038/s41467-024-49493-6)

COMPOUND INFORMATION

SR2211

**CAS Registry No.:**	1359164-11-6
**Formal Name:**	1,1,1,3,3,3-hexafluoro-2-(2-fluoro-4'-((4-(pyridin-4-ylmethyl)piperazin-1-yl)methyl)-[1,1'-biphenyl]-4-yl)propan-2-ol
**EUBOPEN ID:**	EUB0001160a
**Molecular Formula:**	$C_{26}H_{24}F_7N_3O$
**Molecular Weight:**	527.49 g/mol
**Smiles:**	FC1=C(C2=CC=C(CN3CCN(CC4=CC=NC=C4)CC3)C=C2)C=CC(C(C(F)(F)F)(O)C(F)(F)F)=C1
**Recommended concentration:**	1 µM

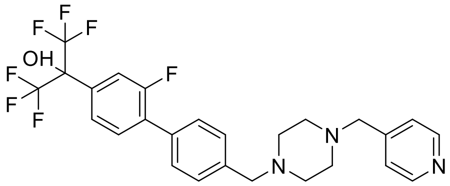

Biological activity

		Type	IC_{50}/EC_{50} [µM]	Reference
Main NR target:	NR1F3 (RORγ)	inv. Agonist	0.3	https://doi.org/10.1021/cb200496y
NR off-target:				

COMPOUND INFORMATION

Identity

^{1}H NMR

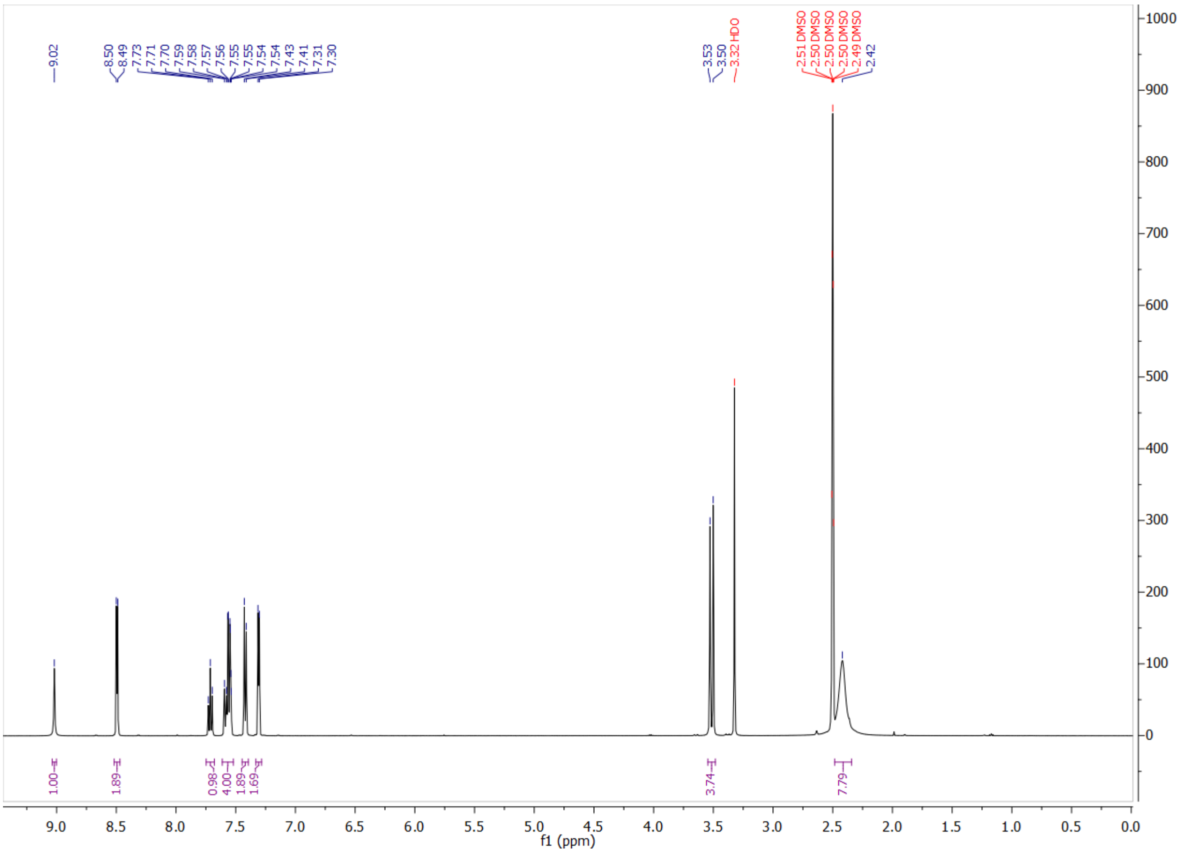

^{13}C NMR

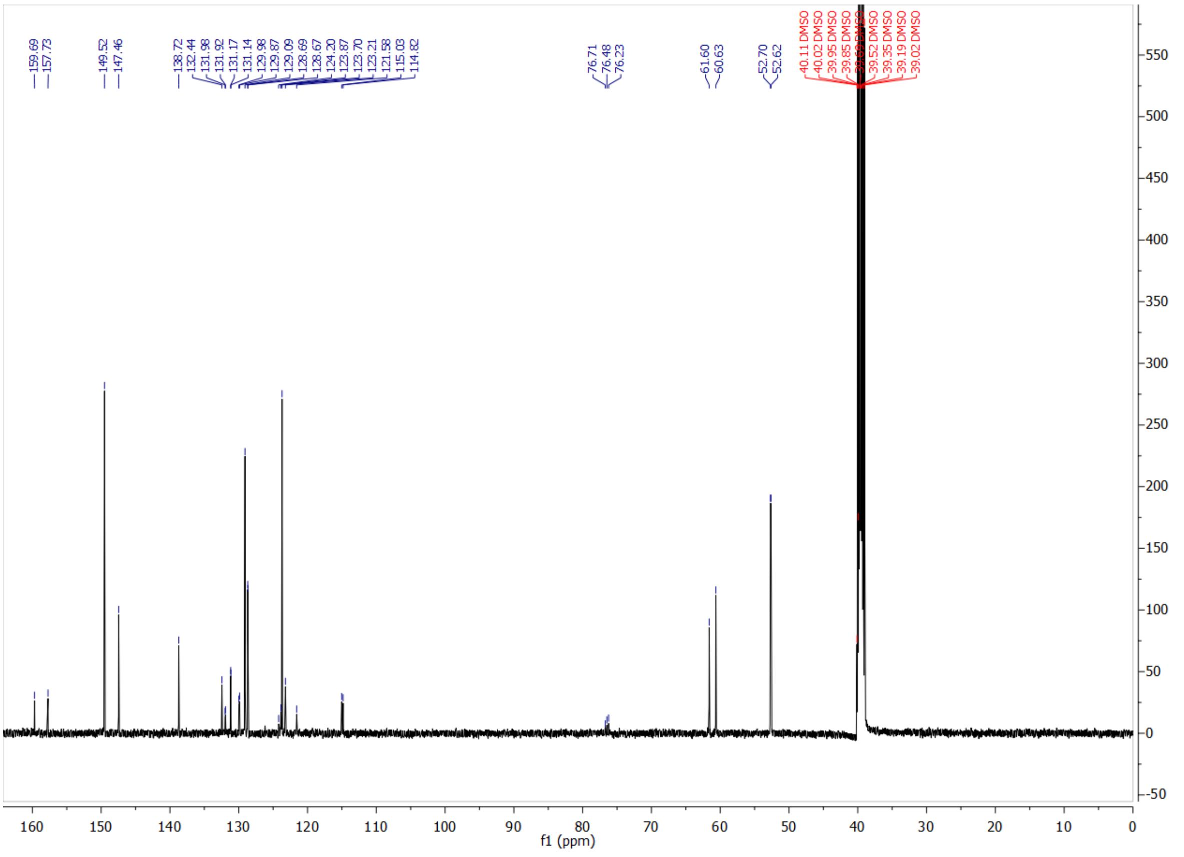

COMPOUND INFORMATION

Purity

Data File W:\analyti...OPEN\CGC_wave3_3rdrun_vials 2023-04-14 23-47-17\004-D1F-B4-SR2211.D
Sample Name: SR2211

```
=====================================================================
Acq. Operator   : SYSTEM                        Seq. Line :    4
Sample Operator : SYSTEM
Acq. Instrument : LCMS test                      Location :   D1F-B4
Injection Date  : 4/15/2023 12:24:54 AM               Inj :    1
                                               Inj Volume : Inj prog
Sequence File   : W:\analytical_LCMS_DATA\EUbOPEN\CGC_wave3_3rdrun_vials 2023-04-14 23-47-17
                  \CGC_wave3_3rdrun_vials.S
Method          : W:\analytical_LCMS_DATA\EUbOPEN\CGC_wave3_3rdrun_vials 2023-04-14 23-47-17
                  \CGL_FIRSTPASS_GENERALMETHOD_VIAL1+2_VIAL_20220414.M (Sequence Method)
Last changed    : 4/14/2023 5:08:13 PM by SYSTEM
Method Info     : CGL wellplate, 0.5 uL of 10 mM DMSO, general method
```

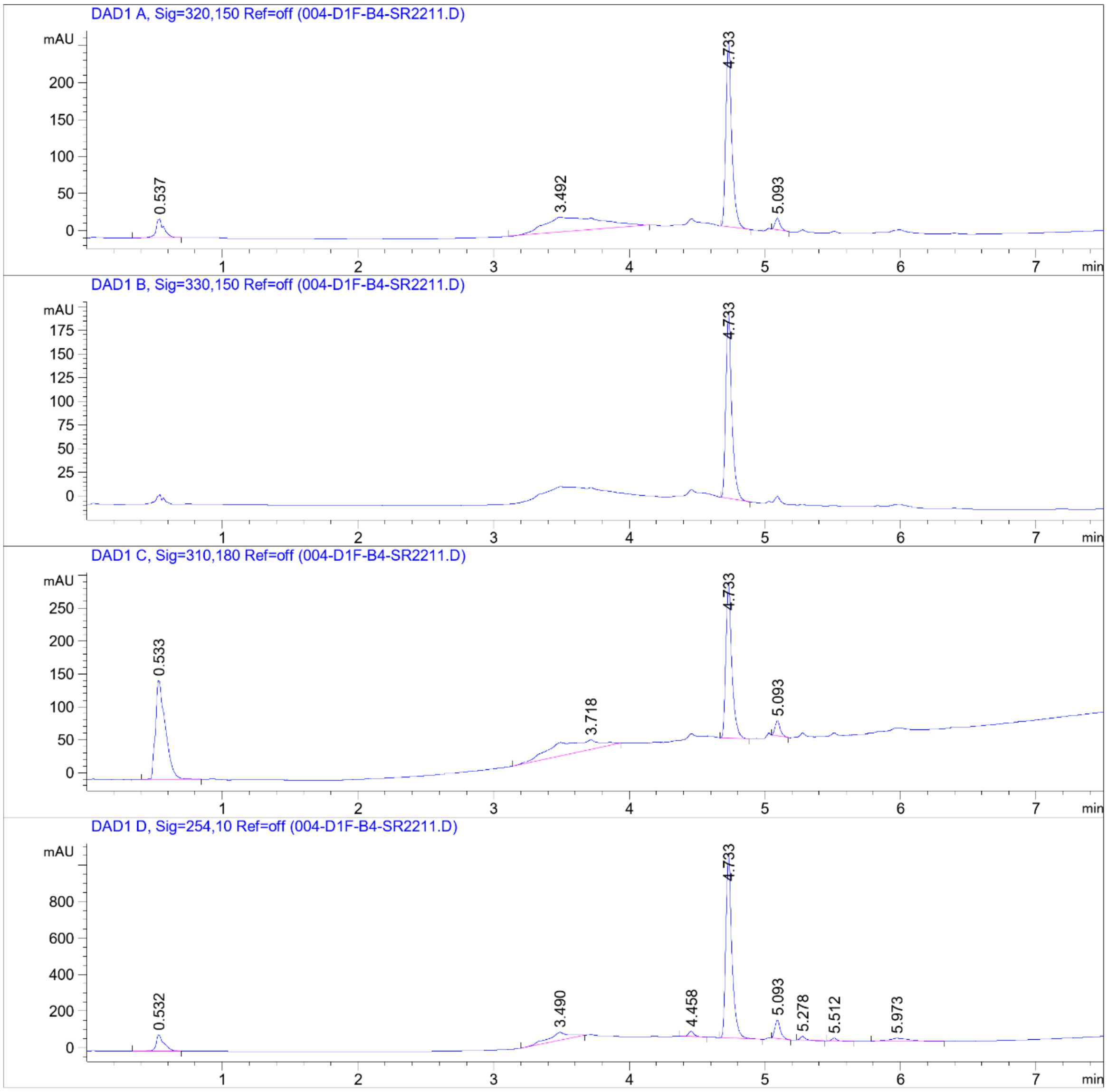

COMPOUND INFORMATION

Data File W:\analyti...OPEN\CGC_wave3_3rdrun_vials 2023-04-14 23-47-17\004-D1F-B4-SR2211.D
Sample Name: SR2211

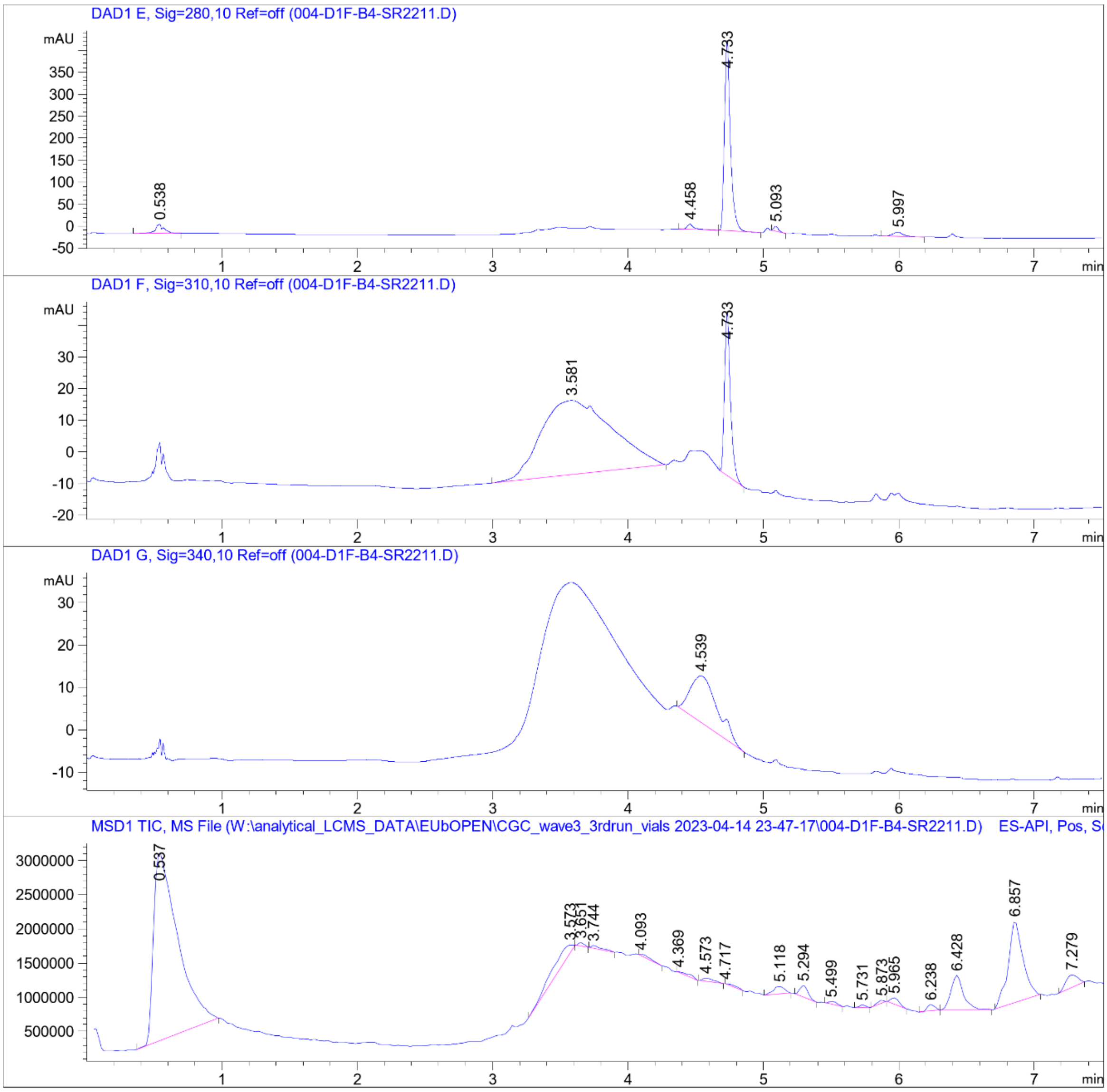

COMPOUND INFORMATION

Data File W:\analyti...OPEN\CGC_wave3_3rdrun_vials 2023-04-14 23-47-17\004-D1F-B4-SR2211.D
Sample Name: SR2211

MS Signal: MSD1 TIC, MS File, ES-API, Pos, Scan, Frag: 70, "POS Scan"
Spectra from peak tops.
Noise Cutoff: 1000 counts.
Reportable Ion Abundance: > 50%.
LC Signal: DAD1 A, Sig=320,150 Ref=off
Peak matching window: 0.1 min

Retention Time (LC)	LC Area	Retention Time (MS)	MS Area	Mol. Weight or Ion
0.537	104	0.537	33914356	157.00 I
3.492	593	-	-	
-	-	3.573	2327656	170.80 I
-	-	3.651	162640	170.80 I
-	-	3.744	235333	170.80 I
-	-	4.093	197062	170.80 I
-	-	4.369	213335	170.80 I
-	-	4.573	273748	528.20 I<br>351.00 I<br>264.60 I<br>178.10 I<br>170.80 I
4.733	763	4.717	127689	528.20 I<br>351.00 I<br>264.60 I<br>178.10 I<br>170.80 I
5.093	43	5.118	606200	528.20 I<br>351.00 I<br>264.60 I<br>178.10 I
-	-	5.294	698966	528.20 I<br>398.20 I<br>376.20 I<br>351.00 I<br>264.60 I<br>178.10 I
-	-	5.499	181000	528.20 I<br>351.00 I<br>264.60 I<br>178.10 I
-	-	5.731	112807	528.10 I<br>351.00 I<br>280.20 I<br>264.60 I<br>178.10 I
-	-	5.873	152922	318.20 I<br>296.20 I
-	-	5.965	390011	528.20 I<br>282.20 I
-	-	6.238	359780	280.20 I<br>228.20 I

COMPOUND INFORMATION

-	-	6.428	3248994	350.20 I<br>282.20 I
-	-	6.857	9042897	282.20 I
-	-	7.279	1142228	400.30 I<br>284.30 I<br>282.20 I

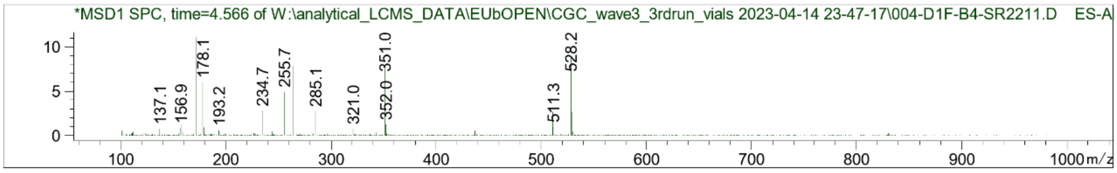

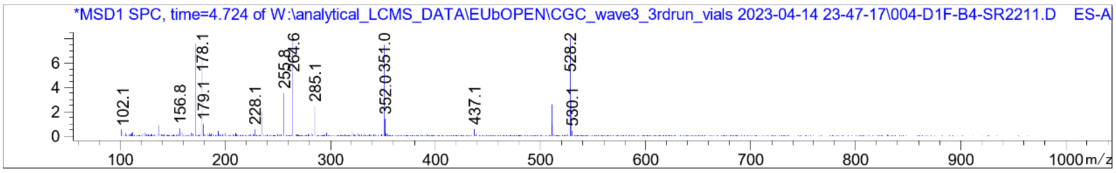

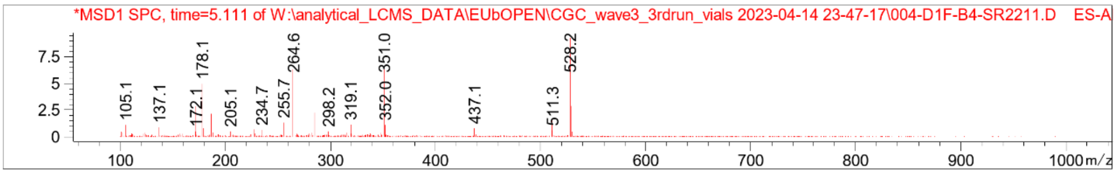

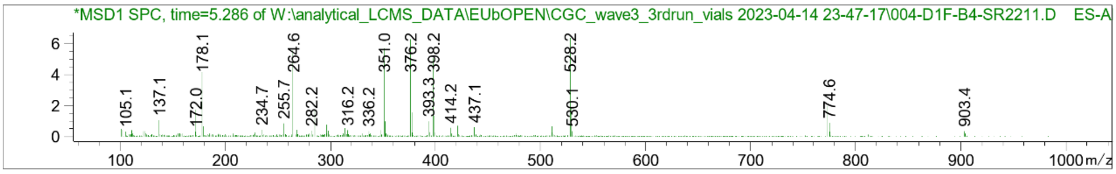

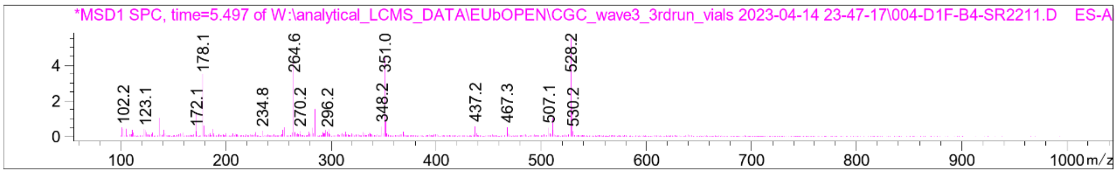

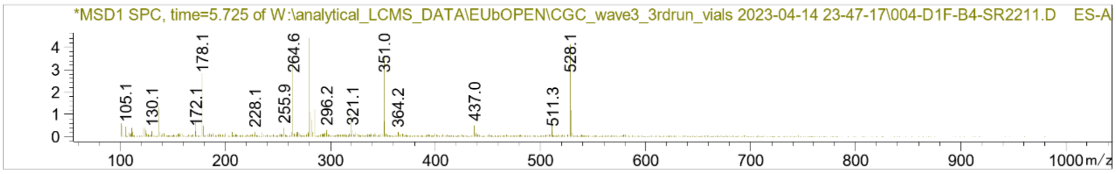

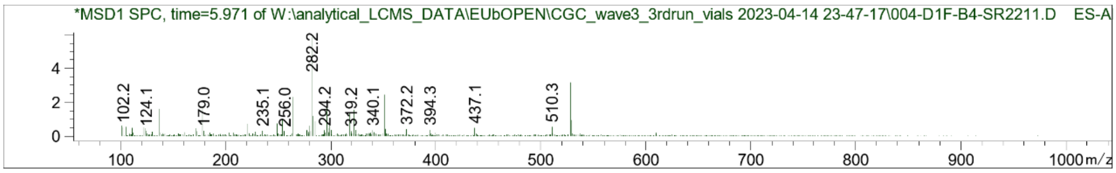

Supplement: Supplementary file 4 — Supplementary Data 1 [file 41467_2024_49493_MOESM4_ESM.zip › SR2211.pdf]
