# Supplementary material for: Chemogenomics for NR1 nuclear hormone receptors
Source: Nat Commun. 2024 Jun 18;15:5201. doi: 10.1038/s41467-024-49493-6 (PMC11189487; doi:10.1038/s41467-024-49493-6)

COMPOUND INFORMATION

SR3335

**CAS Registry No.:**	293753-05-6
**Formal Name:**	N-(4-(1,1,1,3,3,3-hexafluoro-2-hydroxypropan-2-yl)phenyl)thiophene-2-sulfonamide
**EUBOPEN ID:**	EUB0001157a
**Molecular Formula:**	$C_{13}H_9F_6NO_3S_2$
**Molecular Weight:**	405.33 g/mol
**Smiles:**	OC(C(F)(F)F)(C1=CC=C(C=C1)NS(C2=CC=CS2)(=O)=O)C(F)(F)F
**Recommended concentration:**	10 µM

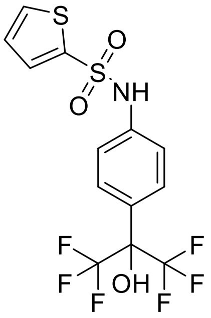

Biological activity

		Type	IC_{50}/EC_{50} [µM]	Reference
Main NR target:	NR1F1 (RORα)	inv. Agonist	0.5	https://doi.org/10.1021/cb1002762
	NR1F2 (RORβ)	inv. Agonist	1.5	inhouse
	NR1F3 (RORγ)	inv. Agonist	3.2	inhouse
NR off-target:				

COMPOUND INFORMATION

Identity

^{1}H NMR

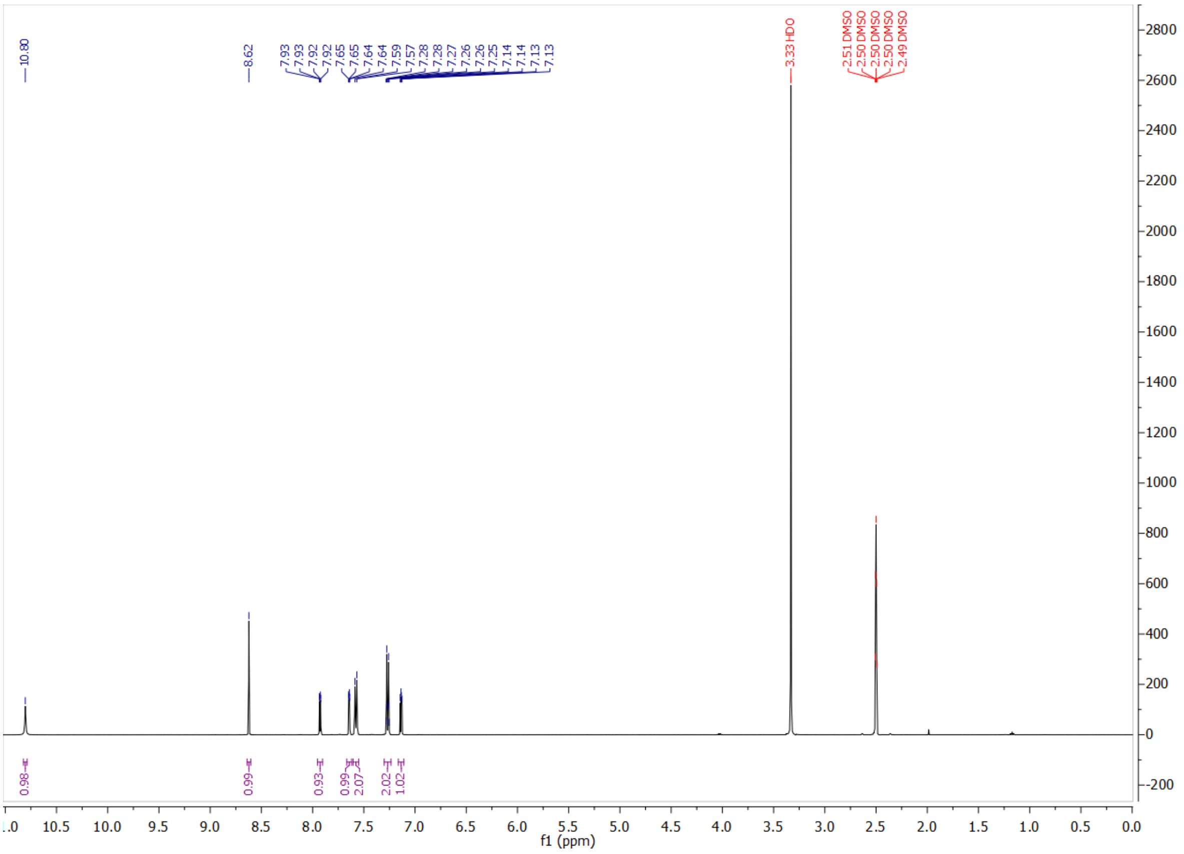

^{13}C NMR

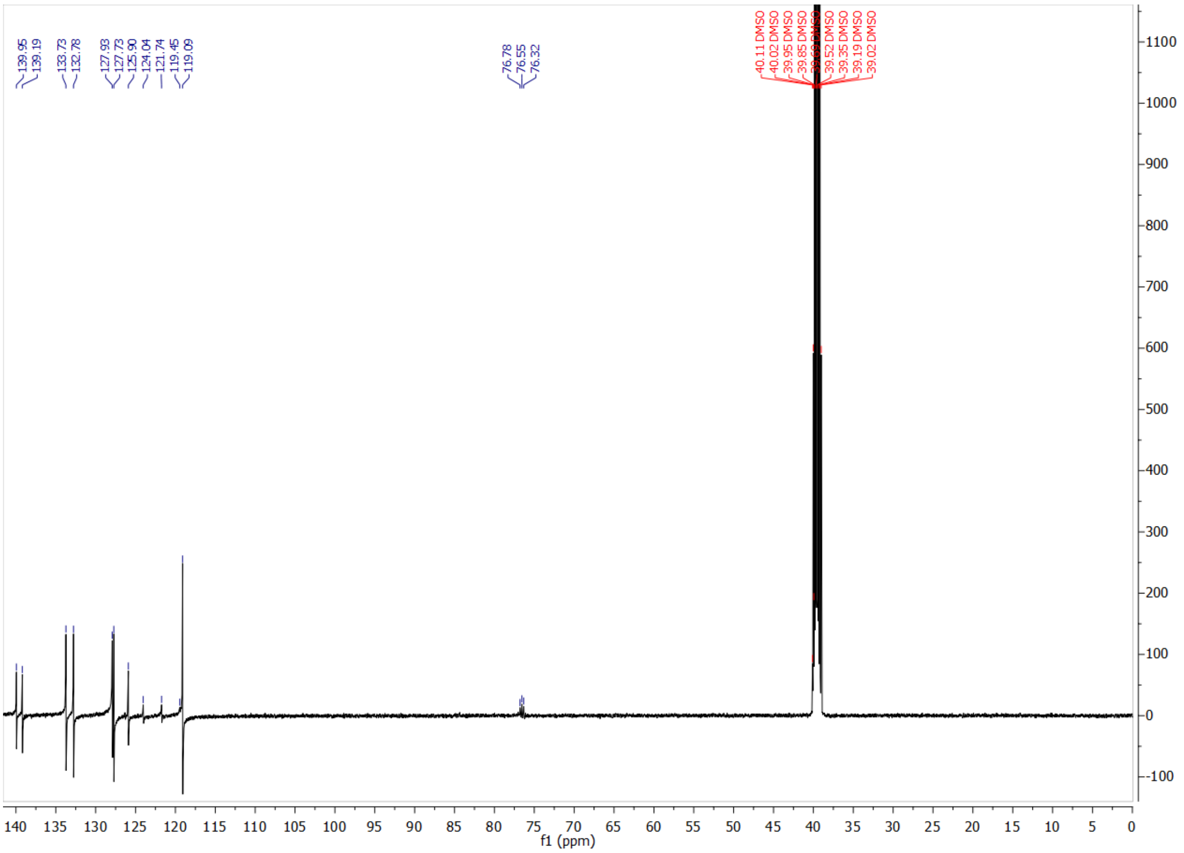

COMPOUND INFORMATION

Purity

```
Data File W:\analyti...OPEN\CGC_wave3_1_FirstPassB 2023-01-04 18-28-02\078-D2F-G3-SR3335.D
Sample Name: SR3335

    =====================================================================
    Acq. Operator   : SYSTEM                        Seq. Line :   78
    Sample Operator : SYSTEM
    Acq. Instrument : LCMS test                      Location :   D2F-G3
    Injection Date  : 1/5/2023 8:42:18 AM                 Inj :    1
                                                   Inj Volume : Inj prog
    Sequence File   : W:\analytical_LCMS_DATA\EUbOPEN\CGC_wave3_1_FirstPassB 2023-01-04 18-28-02
                      \CGC_wave3_1_FirstPassB.S
    Method          : W:\analytical_LCMS_DATA\EUbOPEN\CGC_wave3_1_FirstPassB 2023-01-04 18-28-02
                      \CGL_FIRSTPASS_GENERALMETHOD_VIAL1+2_20210319.M (Sequence Method)
    Last changed    : 1/25/2022 4:36:18 PM by SYSTEM
    Method Info     : CGL wellplate, 0.5 uL of 10 mM DMSO, general method
```

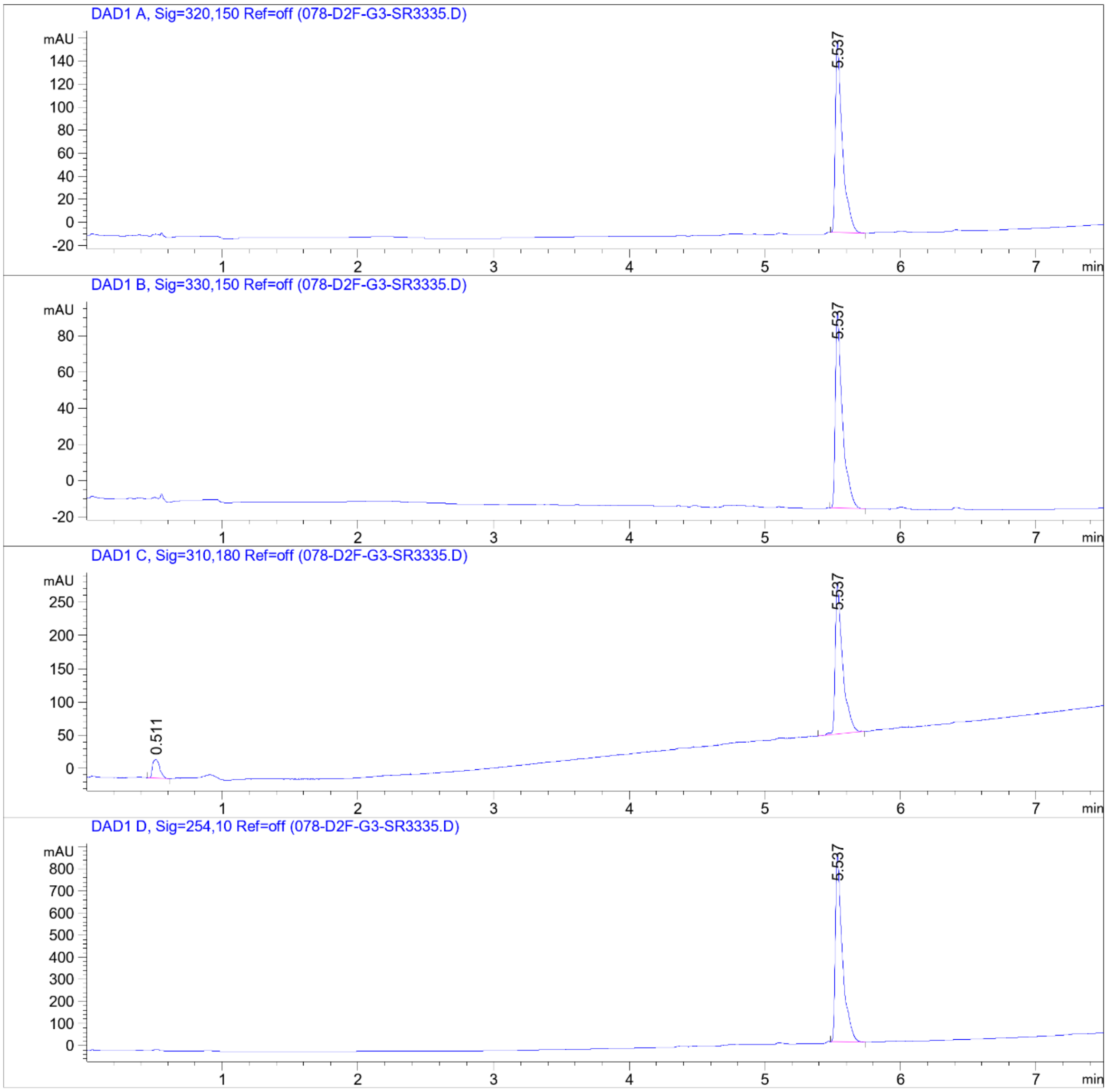

COMPOUND INFORMATION

Data File W:\analyti...OPEN\CGC_wave3_1_FirstPassB 2023-01-04 18-28-02\078-D2F-G3-SR3335.D
Sample Name: SR3335

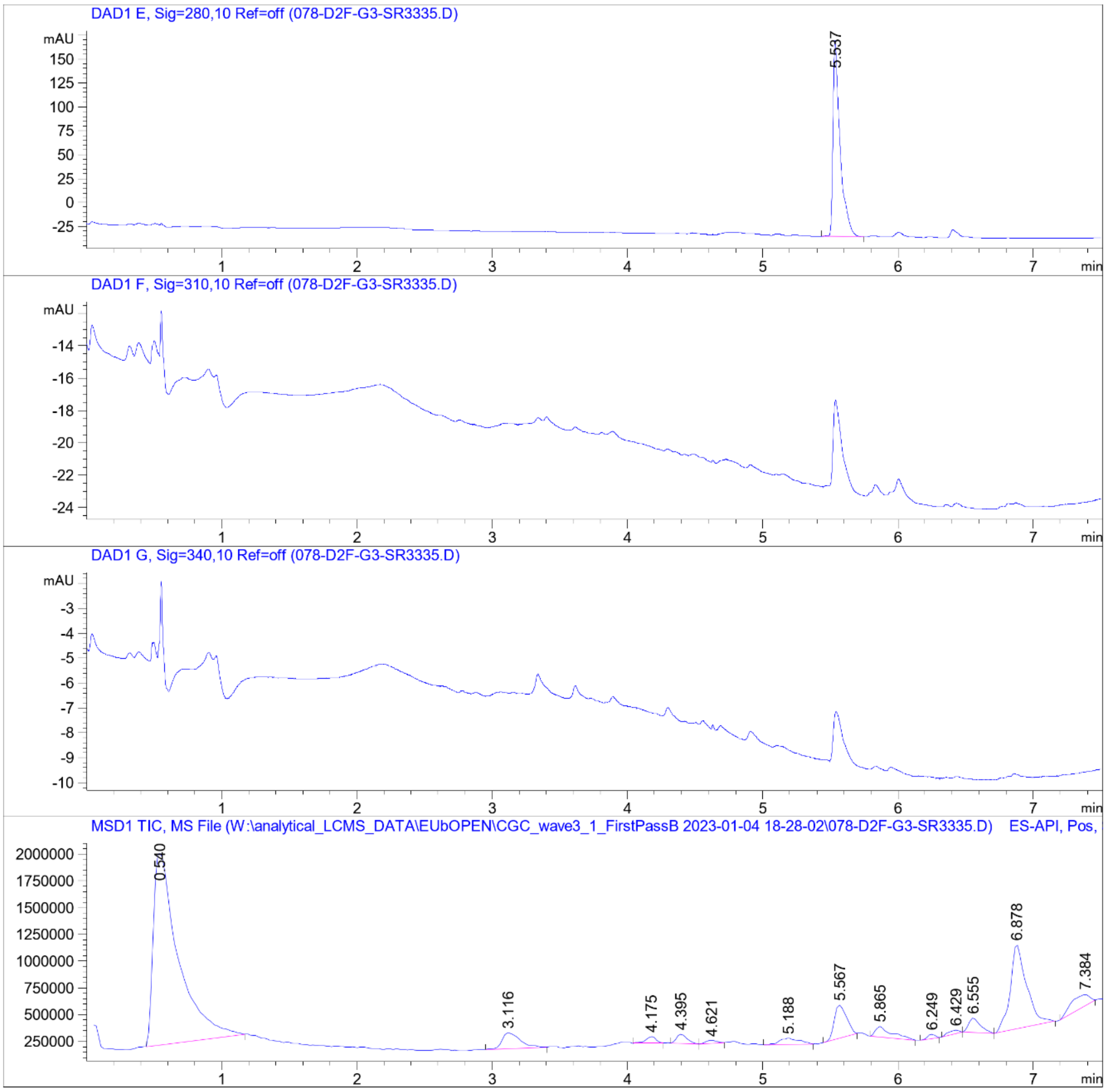

COMPOUND INFORMATION

Data File W:\analyti...OPEN\CGC_wave3_1_FirstPassB 2023-01-04 18-28-02\078-D2F-G3-SR3335.D
Sample Name: SR3335

MS Signal: MSD1 TIC, MS File, ES-API, Pos, Scan, Frag: 70, "POS Scan"
Spectra from peak tops.
Noise Cutoff: 1000 counts.
Reportable Ion Abundance: > 50%.
LC Signal: DAD1 A, Sig=320,150 Ref=off
Peak matching window: 0.1 min

Retention Time (LC)	LC Area	Retention Time (MS)	MS Area	Mol. Weight or Ion
-	-	0.540	23020338	157.00 I
-	-	3.116	1403902	239.00 I
				217.10 I
-	-	4.175	328456	358.10 I
-	-	4.395	454467	260.00 I
-	-	4.621	138582	510.40 I
				170.90 I
				158.10 I
				137.10 I
-	-	5.188	586426	510.40 I
				338.20 I
				298.30 I
				170.90 I
				137.10 I
				105.10 I
5.537	599	5.567	1937924	405.90 I
-	-	5.865	809492	318.20 I
				296.20 I
-	-	6.249	160544	228.20 I
				137.10 I
-	-	6.429	206571	350.20 I
				282.30 I
				254.20 I
				137.10 I
-	-	6.555	823420	507.30 I
				485.30 I
				280.20 I
-	-	6.878	6638833	282.20 I
-	-	7.384	1146268	400.30 I
				282.20 I

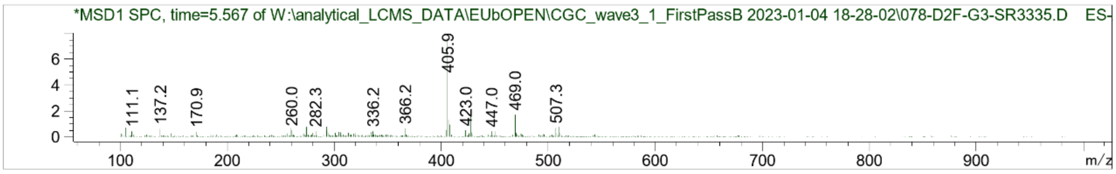

COMPOUND INFORMATION

Biological activity

SR3335
RORβ - IC_{50} 1.5 ± 0.3 µM
0.19 ± 0.06 rem. activity

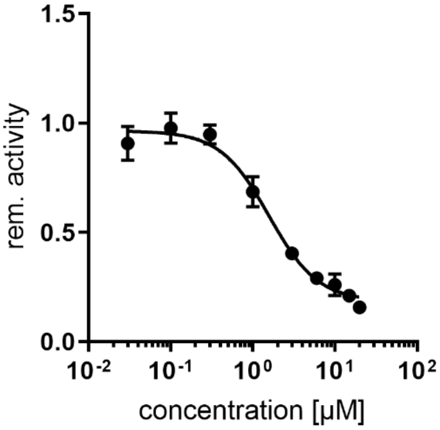

SR3335
RORγ - IC_{50} 3.2 ± 0.6 µM
0.11 ± 0.08 rem. activity

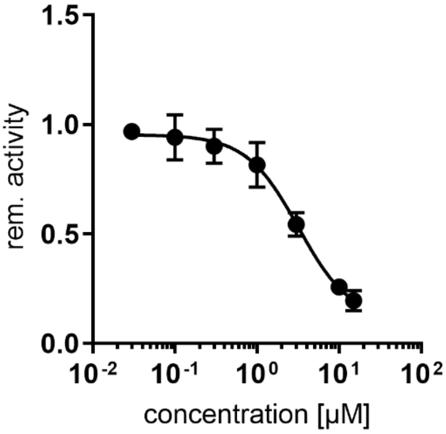

Supplement: Supplementary file 4 — Supplementary Data 1 [file 41467_2024_49493_MOESM4_ESM.zip › SR3335.pdf]
