# Supplementary material for: Chemogenomics for NR1 nuclear hormone receptors
Source: Nat Commun. 2024 Jun 18;15:5201. doi: 10.1038/s41467-024-49493-6 (PMC11189487; doi:10.1038/s41467-024-49493-6)

COMPOUND INFORMATION

SR8278

**CAS Registry No.:**	1254944-66-5
**Formal Name:**	Ethyl 2-(5-(methylthio)thiophene-2-carbonyl)-1,2,3,4-tetrahydroisoquinoline-3-carboxylate
**EUbOPEN ID:**	EUB0001153a
**Molecular Formula:**	$C_{18}H_{19}NO_3S_2$
**Molecular Weight:**	361.47 g/mol
**Smiles:**	CCOC(=O)C1CC2=CC=CC=C2CN1C(=O)C3=CC=C(S3)SC
**Recommended concentration:**	10 µM

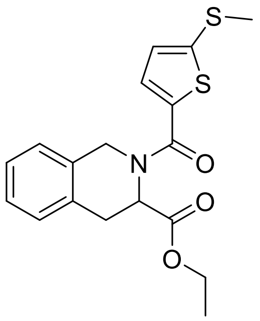

Biological activity

		Type	IC_{50}/EC_{50} [µM]	Reference
Main NR target:	NR1D1 (revERBα)	Antagonist	0.5	https://doi.org/10.1021/cb1002575
NR off-target:				

COMPOUND INFORMATION

Identity

^{1}H NMR

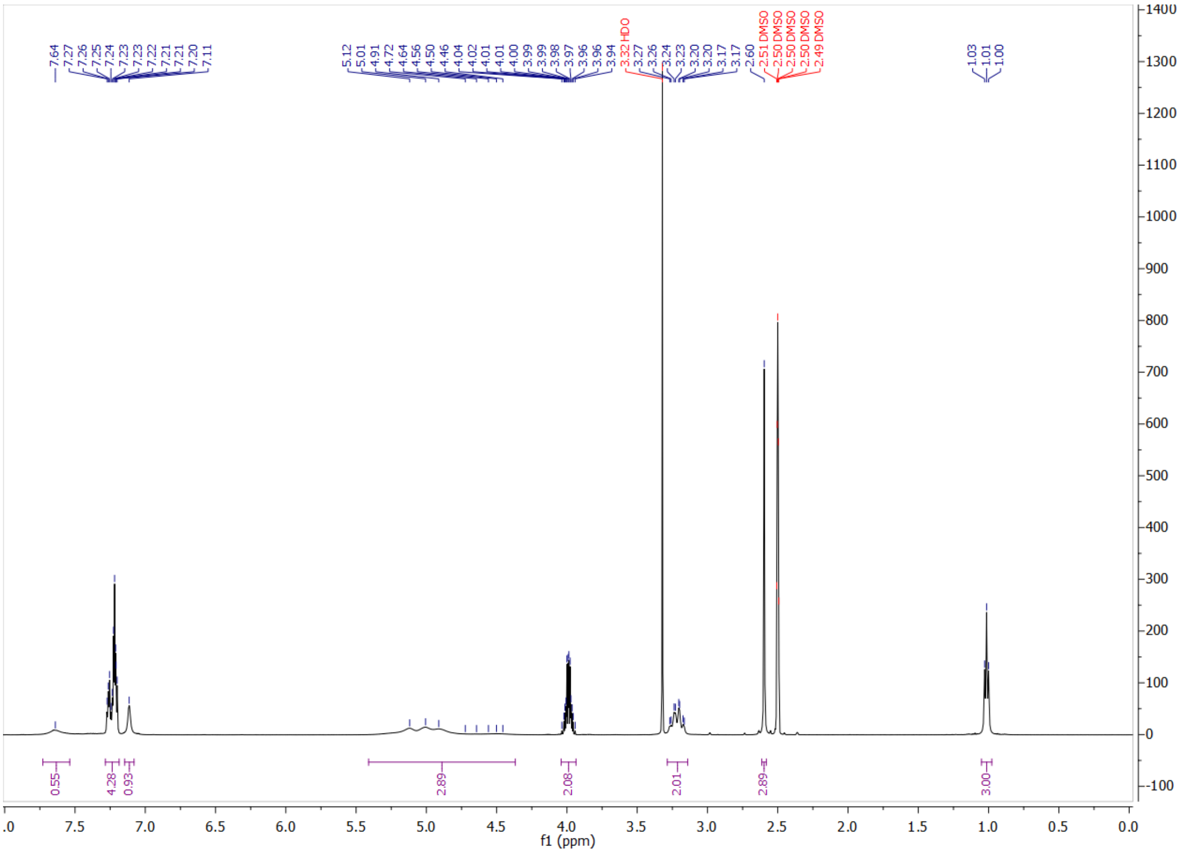

^{13}C NMR

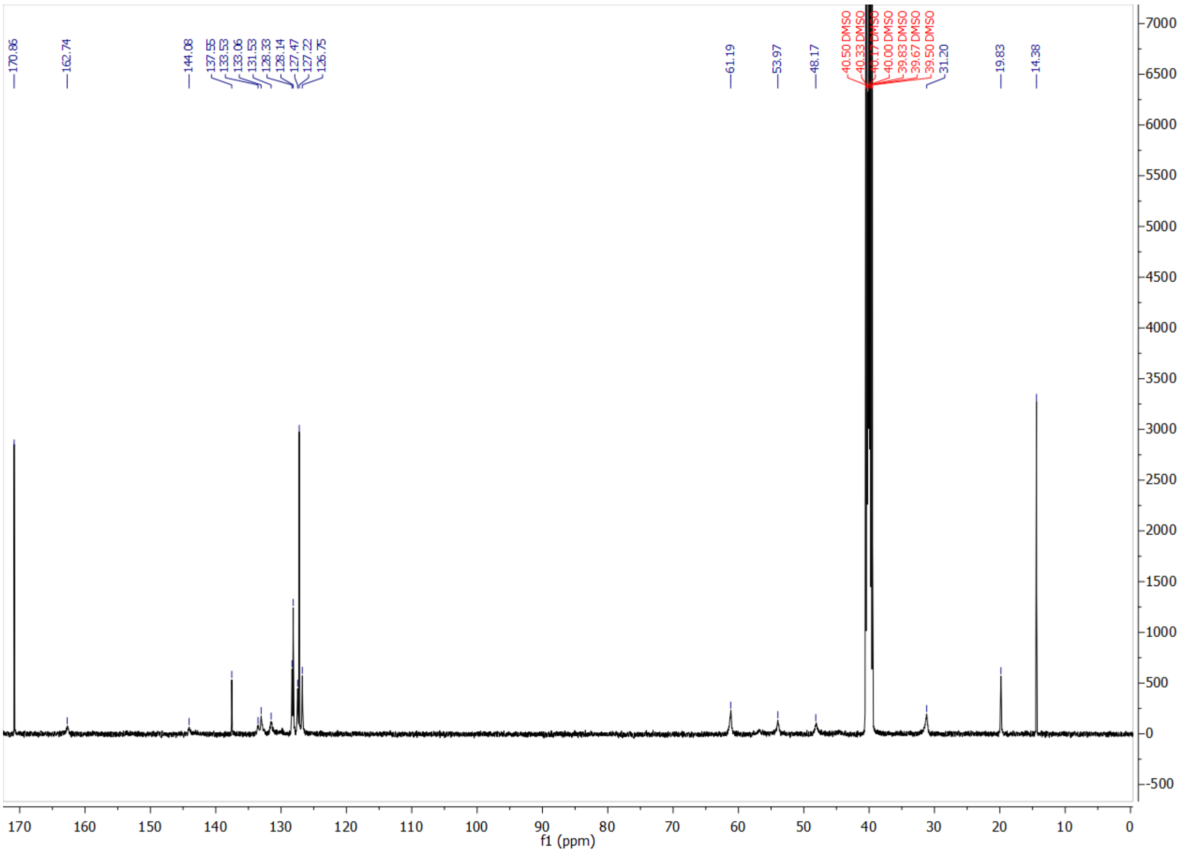

COMPOUND INFORMATION

Purity

Data File W:\analyti...PEN\CGC_wave3_1_FirstPassB 2023-01-04 18-28-02\063-D2F-F11-SR8278.D
Sample Name: SR8278

===

Acq. Operator : SYSTEM Seq. Line : 63
Sample Operator : SYSTEM
Acq. Instrument : LCMS test Location : D2F-F11
Injection Date : 1/5/2023 5:56:25 AM Inj : 1
Inj Volume : Inj prog
Sequence File : W:\analytical_LCMS_DATA\EUbOPEN\CGC_wave3_1_FirstPassB 2023-01-04 18-28-02\CGC_wave3_1_FirstPassB.S
Method : W:\analytical_LCMS_DATA\EUbOPEN\CGC_wave3_1_FirstPassB 2023-01-04 18-28-02\CGL_FIRSTPASS_GENERALMETHOD_VIAL1+2_20210319.M (Sequence Method)
Last changed : 1/25/2022 4:36:18 PM by SYSTEM
Method Info : CGL wellplate, 0.5 uL of 10 mM DMSO, general method

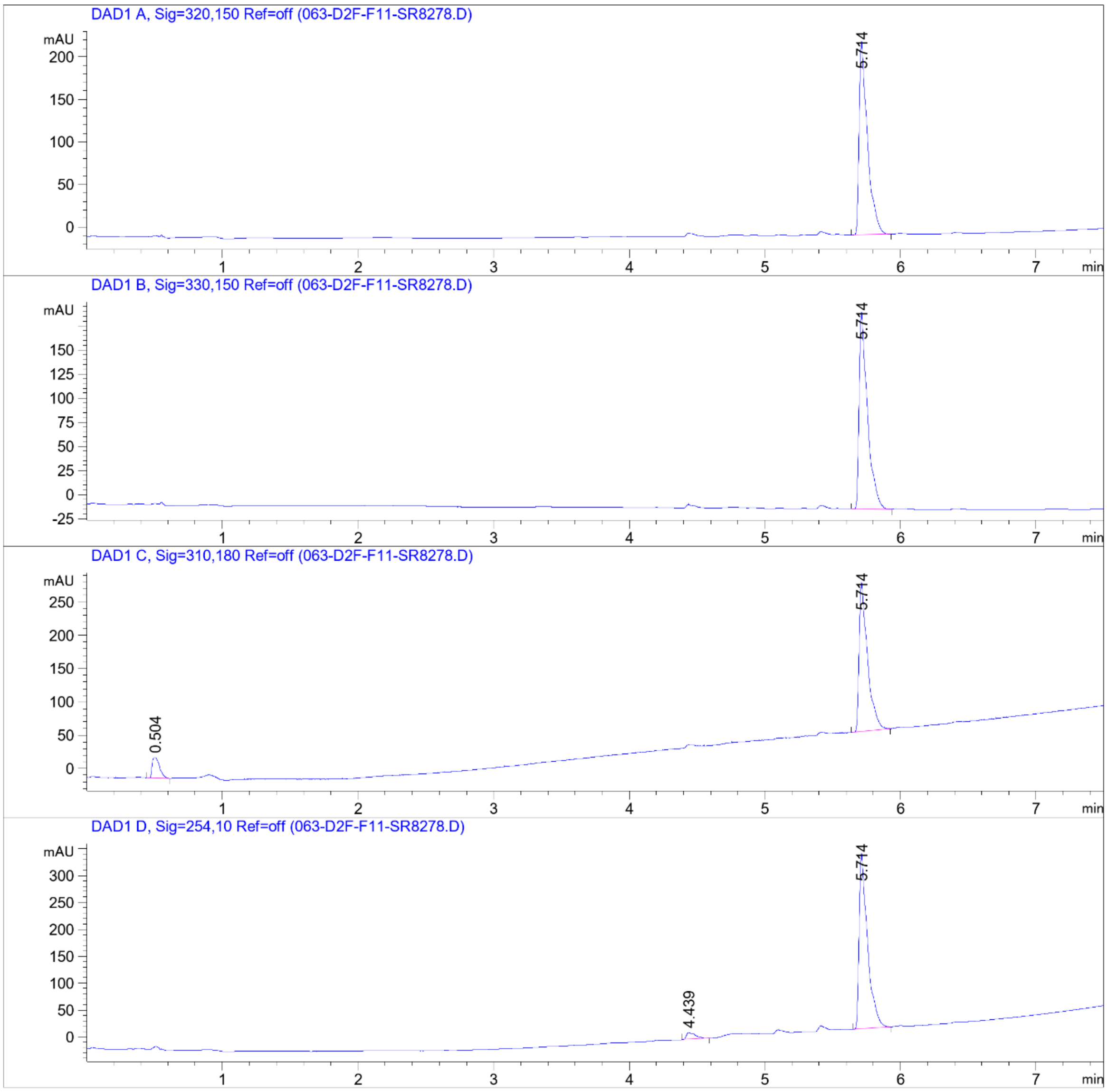

COMPOUND INFORMATION

Data File W:\analyti...PEN\CGC_wave3_1_FirstPassB 2023-01-04 18-28-02\063-D2F-F11-SR8278.D
Sample Name: SR8278

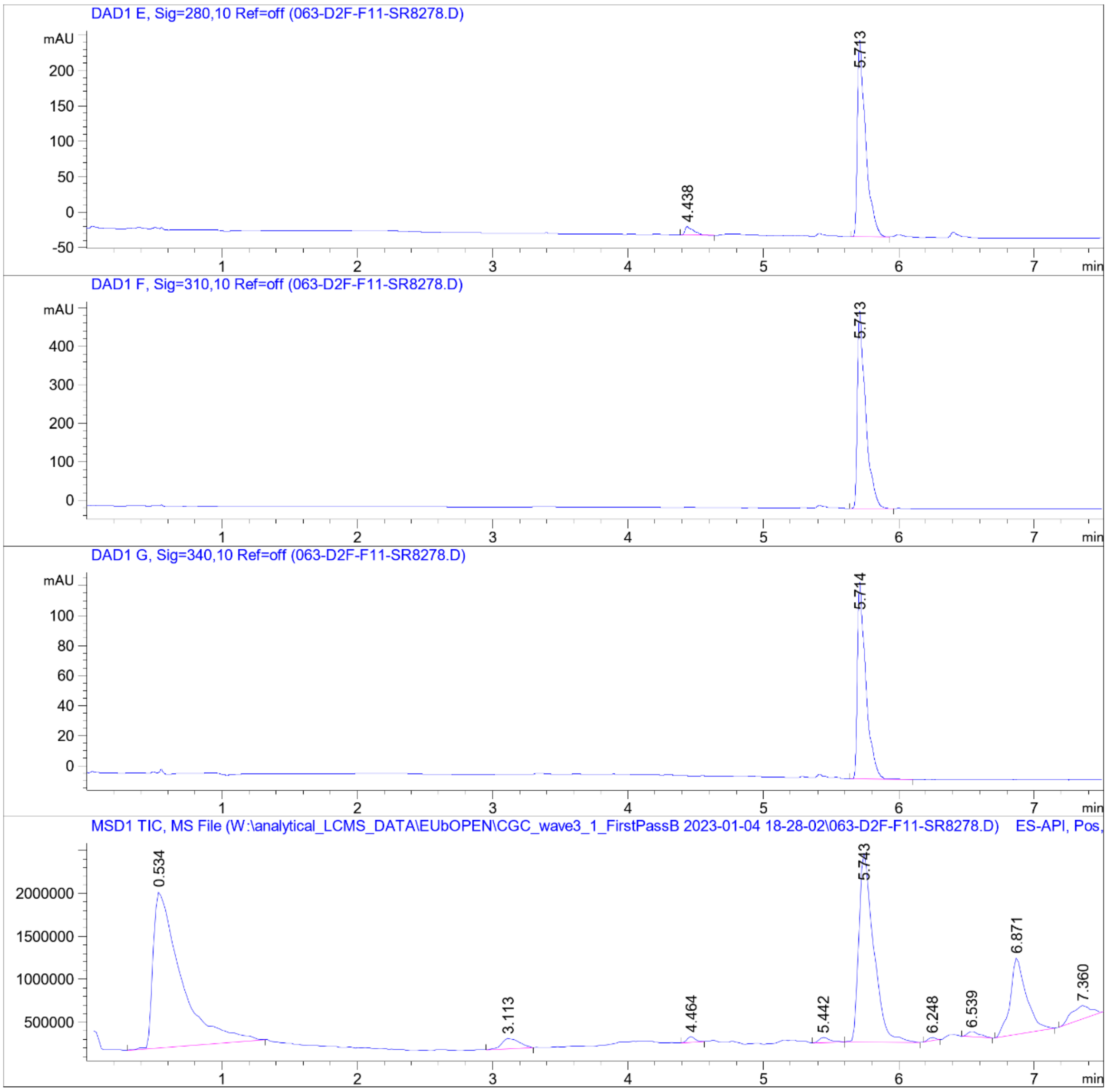

COMPOUND INFORMATION

Data File W:\analyti...PEN\CGC_wave3_1_FirstPassB 2023-01-04 18-28-02\063-D2F-F11-SR8278.D
Sample Name: SR8278

MS Signal: MSD1 TIC, MS File, ES-API, Pos, Scan, Frag: 70, "POS Scan"
Spectra from peak tops.
Noise Cutoff: 1000 counts.
Reportable Ion Abundance: > 50%.
LC Signal: DAD1 A, Sig=320,150 Ref=off
Peak matching window: 0.1 min

Retention Time (LC)	LC Area	Retention Time (MS)	MS Area	Mol. Weight or Ion
-	-	0.534	25976634	157.00 I
-	-	3.113	1076920	239.00 I
				217.10 I
-	-	4.464	283100	510.30 I
				378.00 I
				170.90 I
-	-	5.442	356940	400.00 I
				378.10 I
5.714	966	5.743	16697945	362.00 I
-	-	6.248	151152	228.20 I
				137.10 I
-	-	6.539	391411	280.20 I
-	-	6.871	7415539	282.20 I
-	-	7.360	1630597	400.30 I
				282.30 I

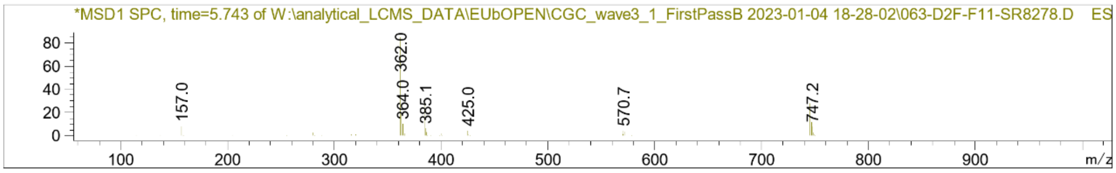

Supplement: Supplementary file 4 — Supplementary Data 1 [file 41467_2024_49493_MOESM4_ESM.zip › SR8278.pdf]
