# Supplementary material for: Chemogenomics for NR1 nuclear hormone receptors
Source: Nat Commun. 2024 Jun 18;15:5201. doi: 10.1038/s41467-024-49493-6 (PMC11189487; doi:10.1038/s41467-024-49493-6)

COMPOUND INFORMATION

SR9009

**CAS Registry No.:**	1379686-30-2
**Formal Name:**	Ethyl 3-(((4-chlorobenzyl)((5-nitrothiophen-2-yl)methyl)amino)methyl)pyrrolidine-1-carboxylate
**EUbOPEN ID:**	EUB0001152a
**Molecular Formula:**	$C_{20}H_{24}ClN_3O_4S$
**Molecular Weight:**	437.94 g/mol
**Smiles:**	CCOC(=O)N1CCC(C1)CN(CC2=CC=C(C=C2)Cl)CC3=CC=C(S3)[N+](=O)[O-]
**Recommended concentration:**	10 µM

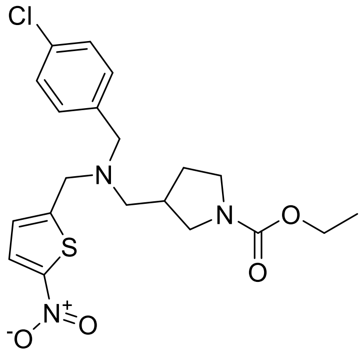

Biological activity

		Type	IC_{50}/EC_{50} [µM]	Reference
Main NR target:	NR1D1 (revERBα)	Agonist	6.0	inhouse
	NR1D2 (revERBβ)	Agonist	11	
NR off-target:				

COMPOUND INFORMATION

Identity

^{1}H NMR

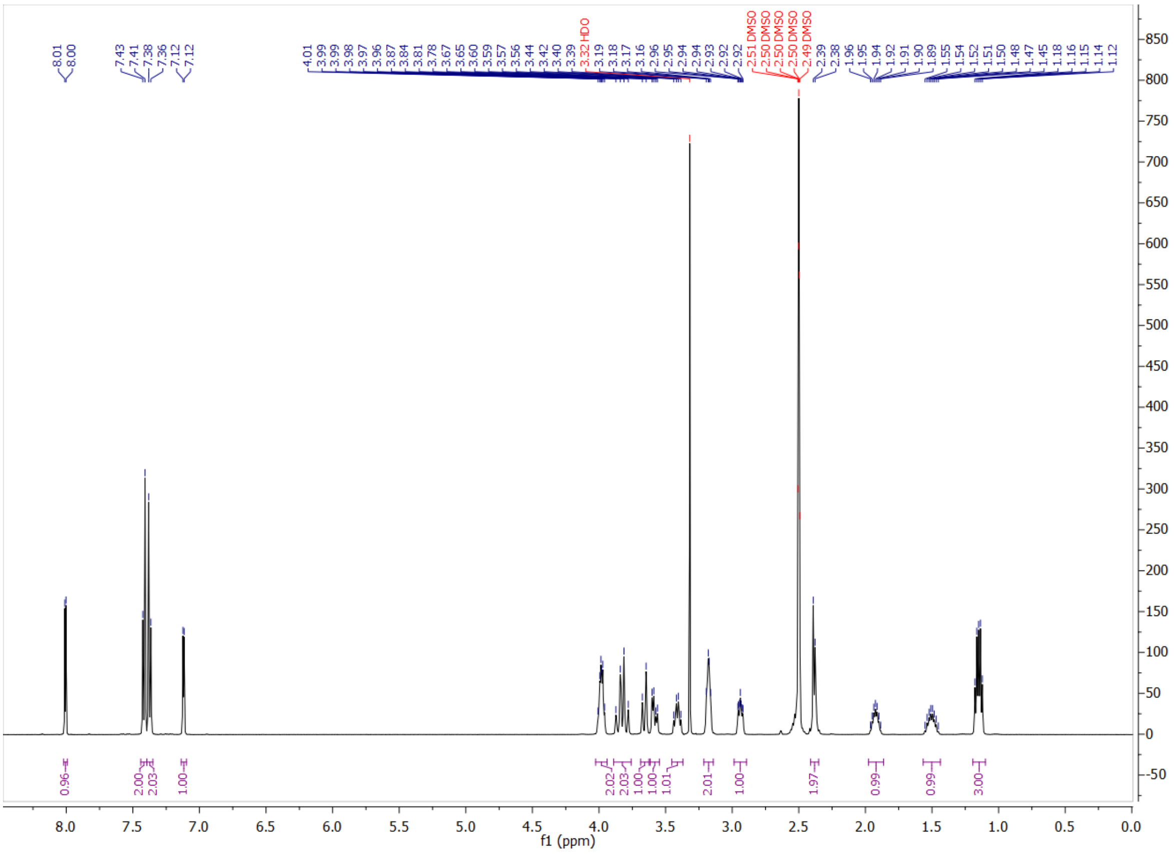

^{13}C NMR

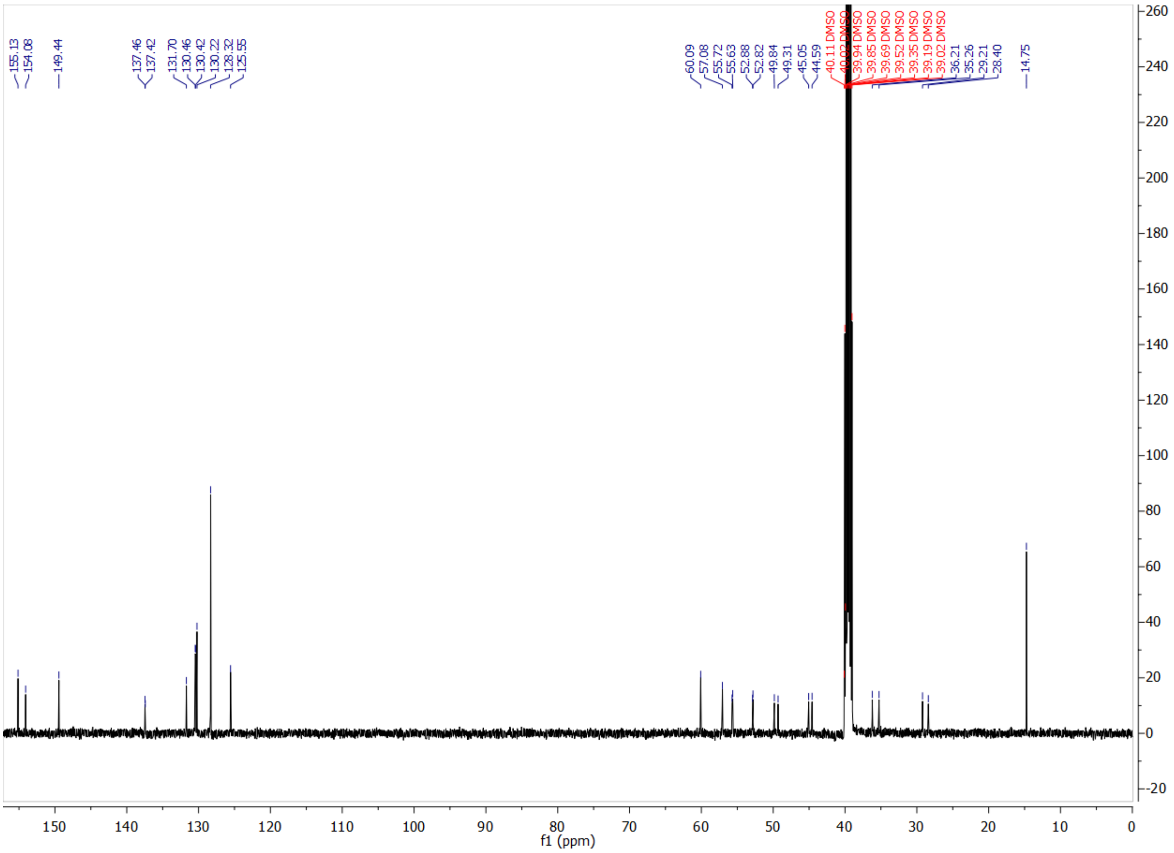

COMPOUND INFORMATION

Purity

Data File W:\analyti...PEN\CGC_wave3_1_FirstPassB 2023-01-04 18-28-02\062-D2F-F10-SR9009.D
Sample Name: SR9009

```
=====================================================================
Acq. Operator   : SYSTEM                          Seq. Line :   62
Sample Operator : SYSTEM
Acq. Instrument : LCMS test                        Location :   D2F-F10
Injection Date  : 1/5/2023 5:45:22 AM                   Inj :   1
                                                Inj Volume : Inj prog
Sequence File   : W:\analytical_LCMS_DATA\EUbOPEN\CGC_wave3_1_FirstPassB 2023-01-04 18-28-02
                  \CGC_wave3_1_FirstPassB.S
Method          : W:\analytical_LCMS_DATA\EUbOPEN\CGC_wave3_1_FirstPassB 2023-01-04 18-28-02
                  \CGL_FIRSTPASS_GENERALMETHOD_VIAL1+2_20210319.M (Sequence Method)
Last changed    : 1/25/2022 4:36:18 PM by SYSTEM
Method Info     : CGL wellplate, 0.5 uL of 10 mM DMSO, general method
```

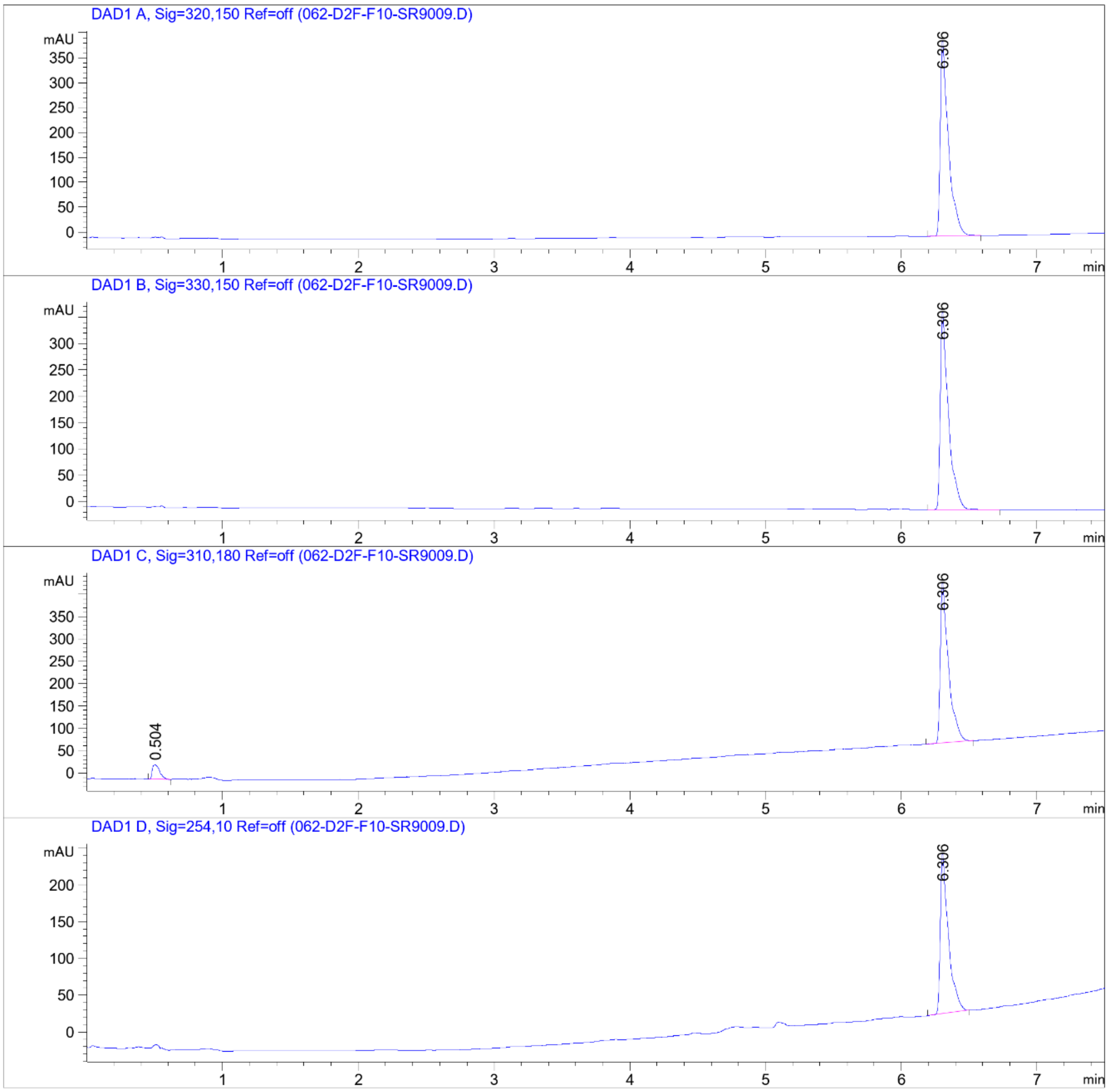

COMPOUND INFORMATION

Data File W:\analyti...PEN\CGC_wave3_1_FirstPassB 2023-01-04 18-28-02\062-D2F-F10-SR9009.D
Sample Name: SR9009

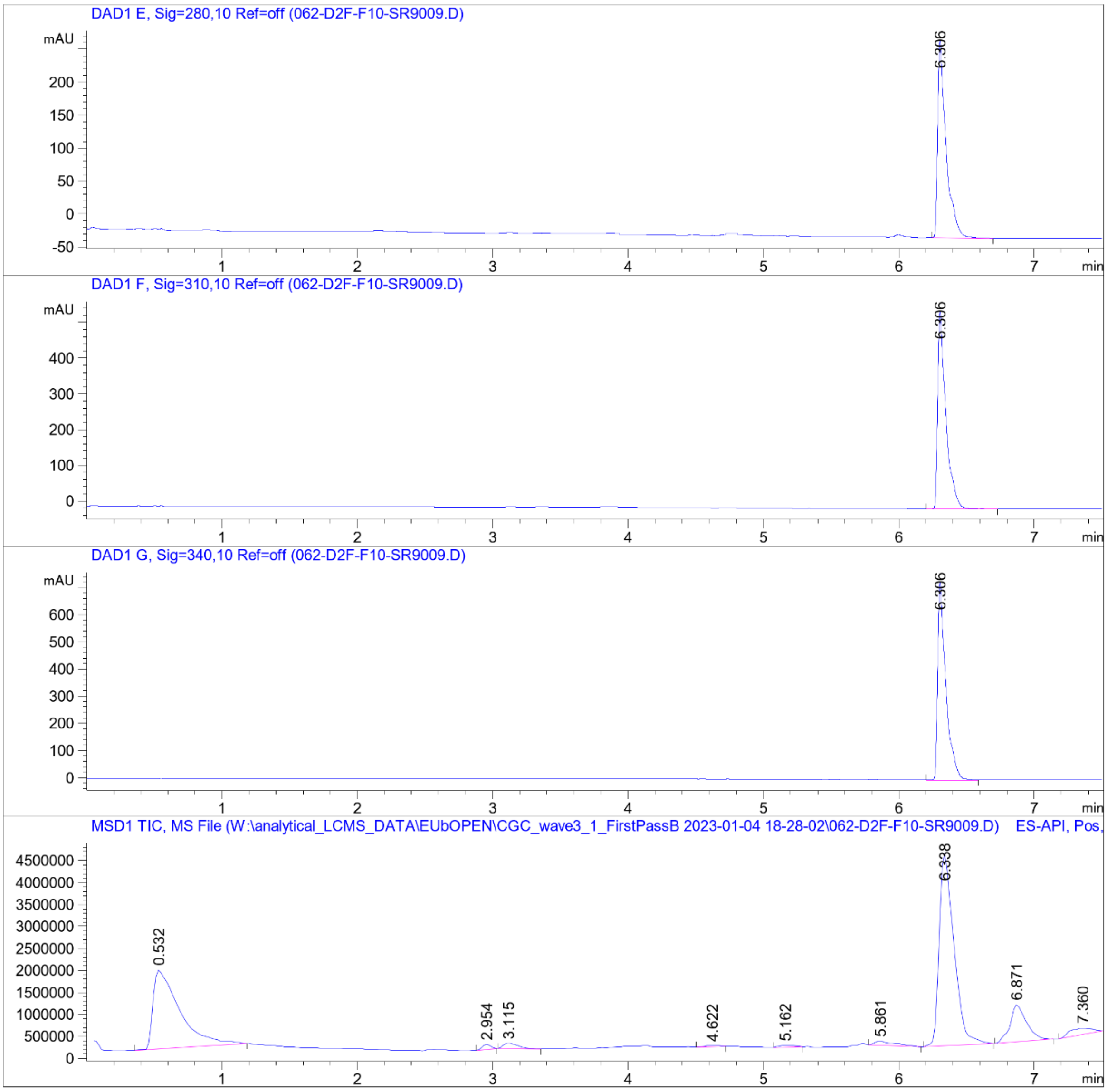

COMPOUND INFORMATION

Data File W:\analyti...PEN\CGC_wave3_1_FirstPassB 2023-01-04 18-28-02\062-D2F-F10-SR9009.D
Sample Name: SR9009

```
MS Signal: MSD1 TIC, MS File,  ES-API, Pos, Scan, Frag: 70, "POS Scan"
    Spectra from peak tops.
    Noise Cutoff: 1000 counts.
    Reportable Ion Abundance: > 50%.
LC Signal: DAD1 A, Sig=320,150 Ref=off
    Peak matching window: 0.1  min
```

Retention Time (LC)	LC Area	Retention Time (MS)	MS Area	Mol. Weight or Ion
-	-	0.532	24555990	157.00 I
-	-	2.954	504443	297.10 I
-	-	3.115	951776	239.00 I<br>217.10 I
-	-	4.622	206301	510.40 I<br>170.80 I
-	-	5.162	321651	510.40 I<br>170.90 I<br>137.20 I
-	-	5.861	886477	318.20 I<br>296.20 I
6.306	1631	6.338	33107130	438.10 I
-	-	6.871	7148443	282.20 I
-	-	7.360	1752307	400.40 I<br>282.30 I

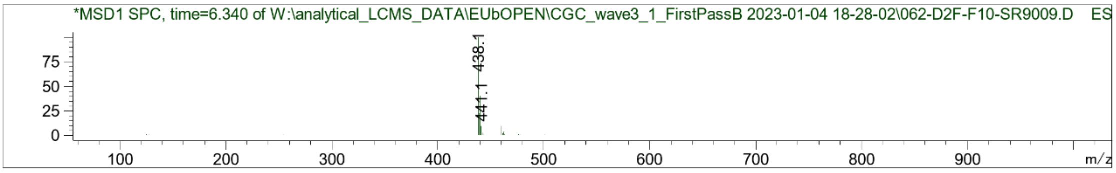

Biological activity

SR9009
revERBα - IC_{50} 6.0 ± 0.8 µM
0.12 ± 0.07 rem. activity

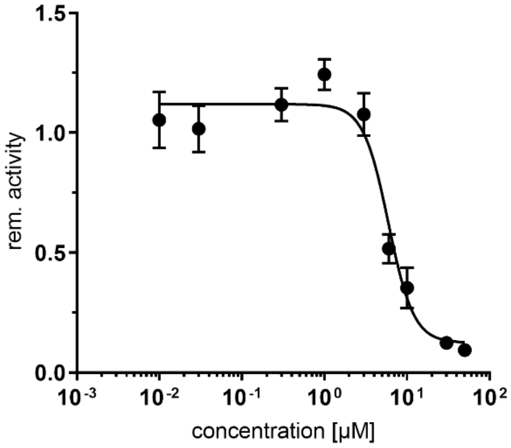

SR9009
revERBβ - IC_{50} 11 ± 2 µM
0.3 ± 0.1 rem. activity

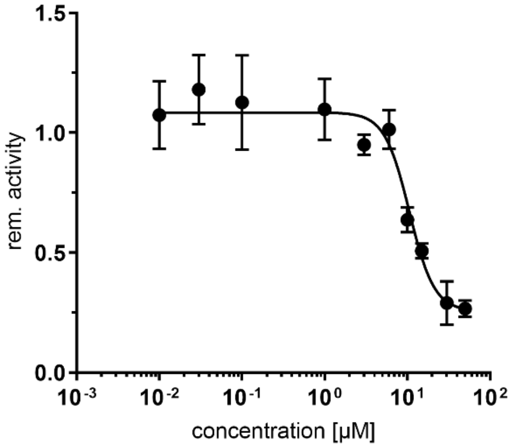

Supplement: Supplementary file 4 — Supplementary Data 1 [file 41467_2024_49493_MOESM4_ESM.zip › SR9009.pdf]
