# Supplementary material for: Chemogenomics for NR1 nuclear hormone receptors
Source: Nat Commun. 2024 Jun 18;15:5201. doi: 10.1038/s41467-024-49493-6 (PMC11189487; doi:10.1038/s41467-024-49493-6)

COMPOUND INFORMATION

SR9011

**CAS Registry No.:**	1379686-29-9
**Formal Name:**	3-(((4-chlorobenzyl)((5-nitrothiophen-2-yl)methyl)amino)methyl)-N-pentylpyrrolidine-1-carboxamide
**EUBOPEN ID:**	EUB0001155a
**Molecular Formula:**	$C_{23}H_{31}ClN_4O_3S$
**Molecular Weight:**	479.04 g/mol
**Smiles:**	ClC1=CC=C(CN(CC2=CC=C(S2)[N+]([O])=O)CC3CCN(C3)C(NCCCCC)=O)C=C1
**Recommended concentration:**	10 µM

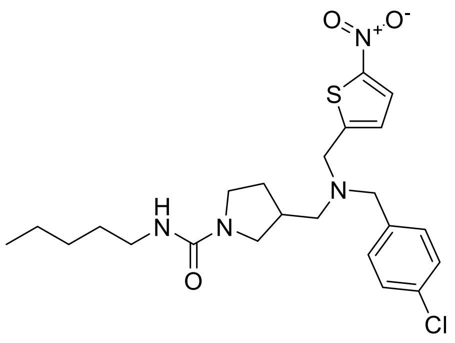

Biological activity

		Type	IC_{50}/EC_{50} [µM]	Reference
Main NR target:	NR1D1 (revERBα)	Agonist	2	inhouse
	NR1D2 (revERBβ)	Agonist	3.1	inhouse
NR off-target:				

COMPOUND INFORMATION

Identity

¹H NMR

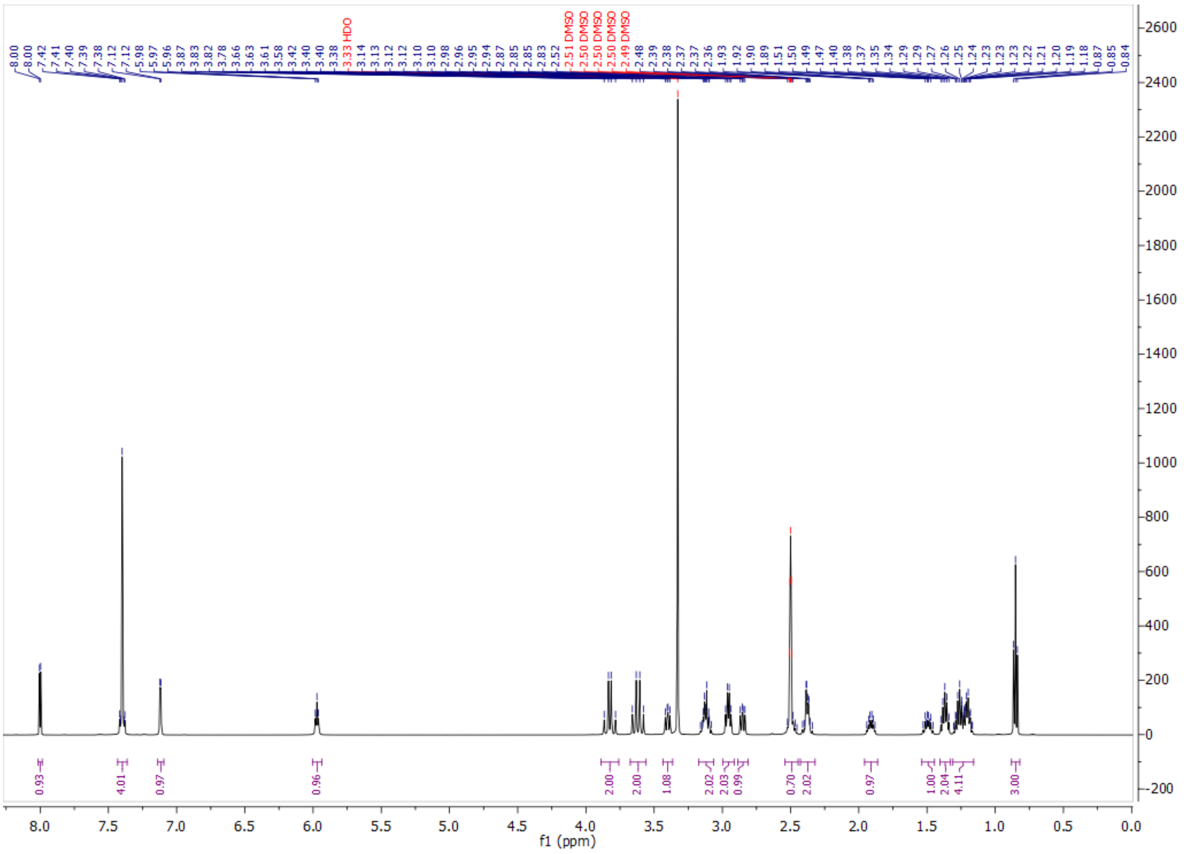

¹³C NMR

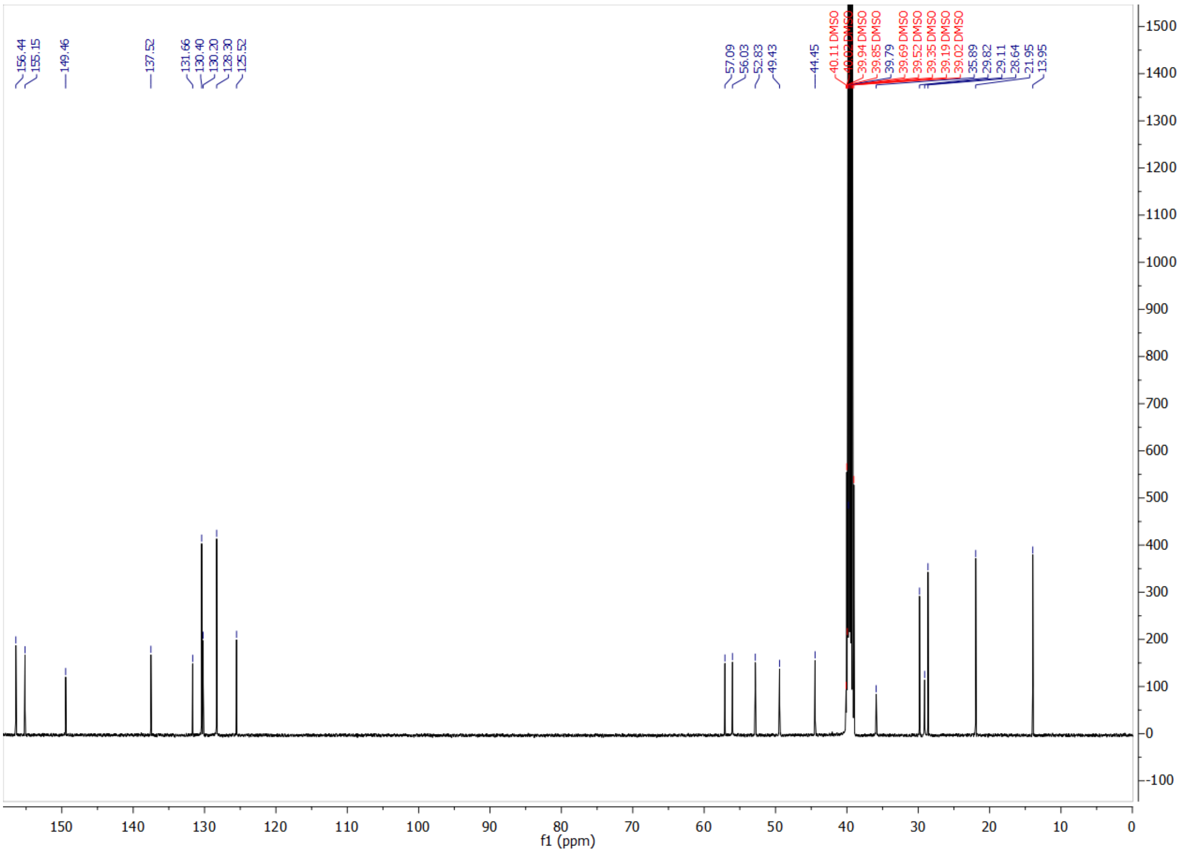

COMPOUND INFORMATION

EUbOPEN
Enabling & Unlocking Biology in the OPEN

Purity

```
Data File W:\analyti...OPEN\CGC_wave3_1_FirstPassB 2023-01-04 18-28-02\073-D2F-G1-SR9011.D
Sample Name: SR9011

    =====================================================================
    Acq. Operator   : SYSTEM                         Seq. Line :   73
    Sample Operator : SYSTEM
    Acq. Instrument : LCMS test                       Location :   D2F-G1
    Injection Date  : 1/5/2023 7:46:59 AM                  Inj :   1
                                                Inj Volume : Inj prog
    Sequence File   : W:\analytical_LCMS_DATA\EUbOPEN\CGC_wave3_1_FirstPassB 2023-01-04 18-28-02
                      \CGC_wave3_1_FirstPassB.S
    Method          : W:\analytical_LCMS_DATA\EUbOPEN\CGC_wave3_1_FirstPassB 2023-01-04 18-28-02
                      \CGL_FIRSTPASS_GENERALMETHOD_VIAL1+2_20210319.M (Sequence Method)
    Last changed    : 1/25/2022 4:36:18 PM by SYSTEM
    Method Info     : CGL wellplate, 0.5 uL of 10 mM DMSO, general method
```

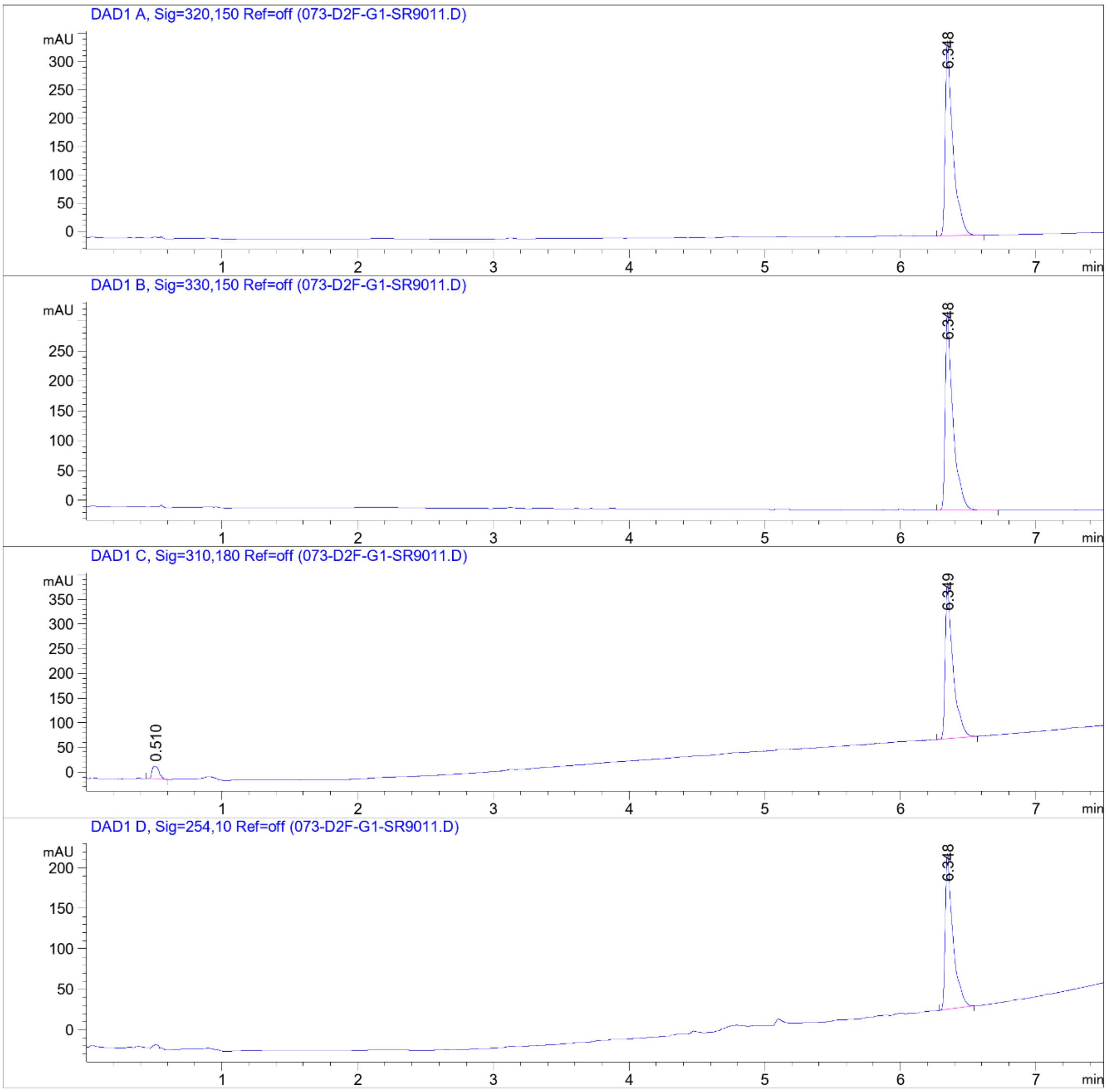

COMPOUND INFORMATION

Data File W:\analyti...OPEN\CGC_wave3_1_FirstPassB 2023-01-04 18-28-02\073-D2F-G1-SR9011.D
Sample Name: SR9011

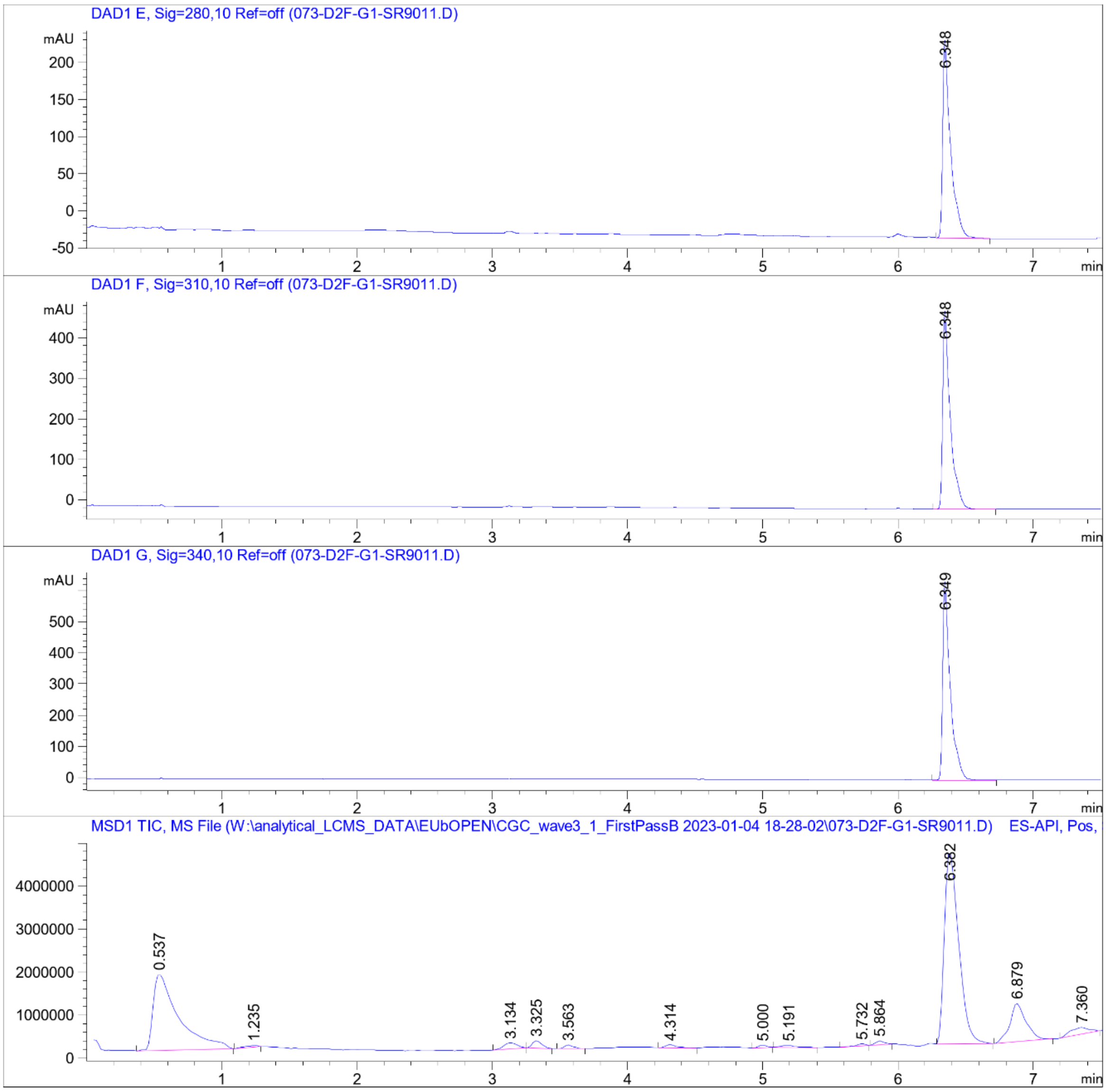

COMPOUND INFORMATION

Data File W:\analyti...OPEN\CGC_wave3_1_FirstPassB 2023-01-04 18-28-02\073-D2F-G1-SR9011.D
Sample Name: SR9011

MS Signal: MSD1 TIC, MS File, ES-API, Pos, Scan, Frag: 70, "POS Scan"
Spectra from peak tops.
Noise Cutoff: 1000 counts.
Reportable Ion Abundance: > 50%.
LC Signal: DAD1 A, Sig=320,150 Ref=off
Peak matching window: 0.1 min

Retention Time (LC)	LC Area	Retention Time (MS)	MS Area	Mol. Weight or Ion
-	-	0.537	23147890	157.00 I
-	-	1.235	239192	157.00 I
-	-	3.134	963696	239.10 I
				217.10 I
				188.10 I
-	-	3.325	810373	338.20 I
-	-	3.563	437907	215.10 I
-	-	4.314	405057	257.10 I
-	-	5.000	242234	271.20 I
-	-	5.191	471579	510.40 I
				170.90 I
				137.10 I
-	-	5.732	190906	280.20 I
-	-	5.864	390894	318.20 I
				296.20 I
6.348	1346	6.382	32871608	479.20 I
-	-	6.879	7439279	282.20 I
-	-	7.360	1563593	400.30 I
				282.20 I

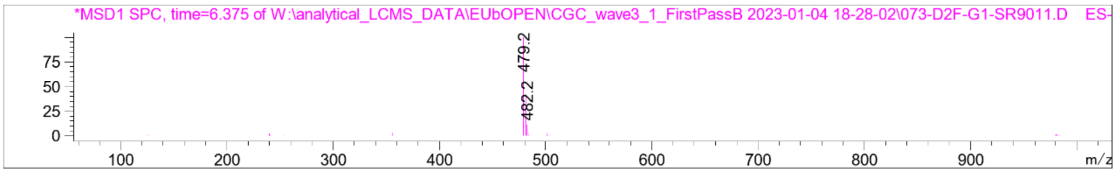

COMPOUND INFORMATION

Biological activity

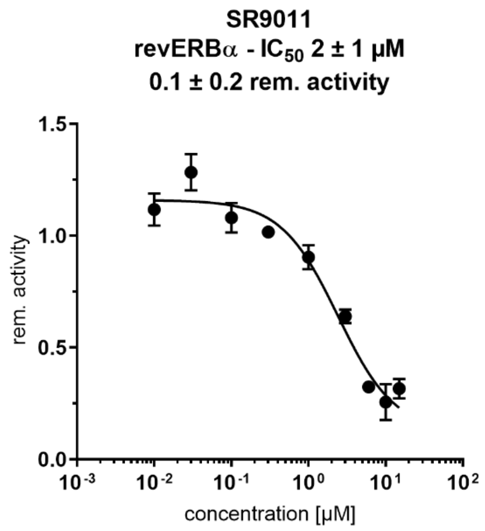

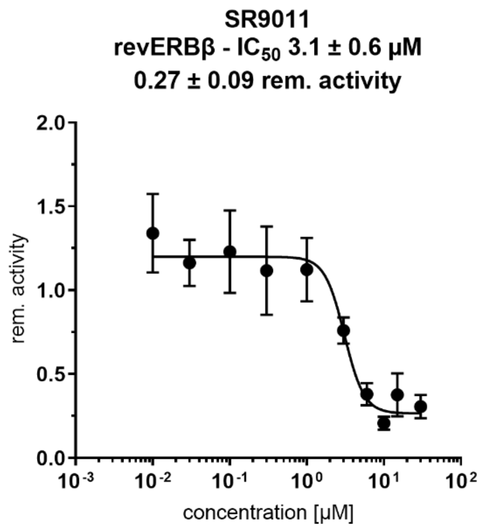

Supplement: Supplementary file 4 — Supplementary Data 1 [file 41467_2024_49493_MOESM4_ESM.zip › SR9011.pdf]
