# Supplementary material for: Chemogenomics for NR1 nuclear hormone receptors
Source: Nat Commun. 2024 Jun 18;15:5201. doi: 10.1038/s41467-024-49493-6 (PMC11189487; doi:10.1038/s41467-024-49493-6)

COMPOUND INFORMATION

SR9238

**CAS Registry No.:**	1416153-62-2
**Formal Name:**	Ethyl 5-(((2,4,6-trimethyl-N-((3'-(methylsulfonyl)-[1,1'-biphenyl]-4-yl)methyl)phenyl)sulfonamido)methyl)furan-2-carboxylate
**EUBOPEN ID:**	EUB0000082b
**Molecular Formula:**	$C_{31}H_{33}NO_7S_2$
**Molecular Weight:**	595.73 g/mol
**Smiles:**	CS(C1=CC=CC(C2=CC=C(C=C2)CN(S(C3=C(C=C(C=C3C)C)C)(=O)=O)CC4=CC=C(O4)C(OCC)=O)=C1)(=O)=O
**Recommended concentration:**	1 µM

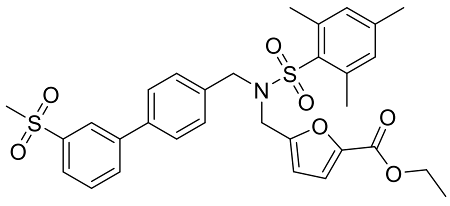

Biological activity

		Type	IC_{50}/EC_{50} [µM]	Reference
Main NR target:	NR1H3 (LXRα)	Antagonist	0.2	https://doi.org/10.1021/cb300541g
	NR1H2 (LXRβ)	Antagonist	0.04	
NR off-target:				

COMPOUND INFORMATION

EUbOPEN
Enabling & Unlocking Biology in the OPEN

Identity

^{1}H NMR

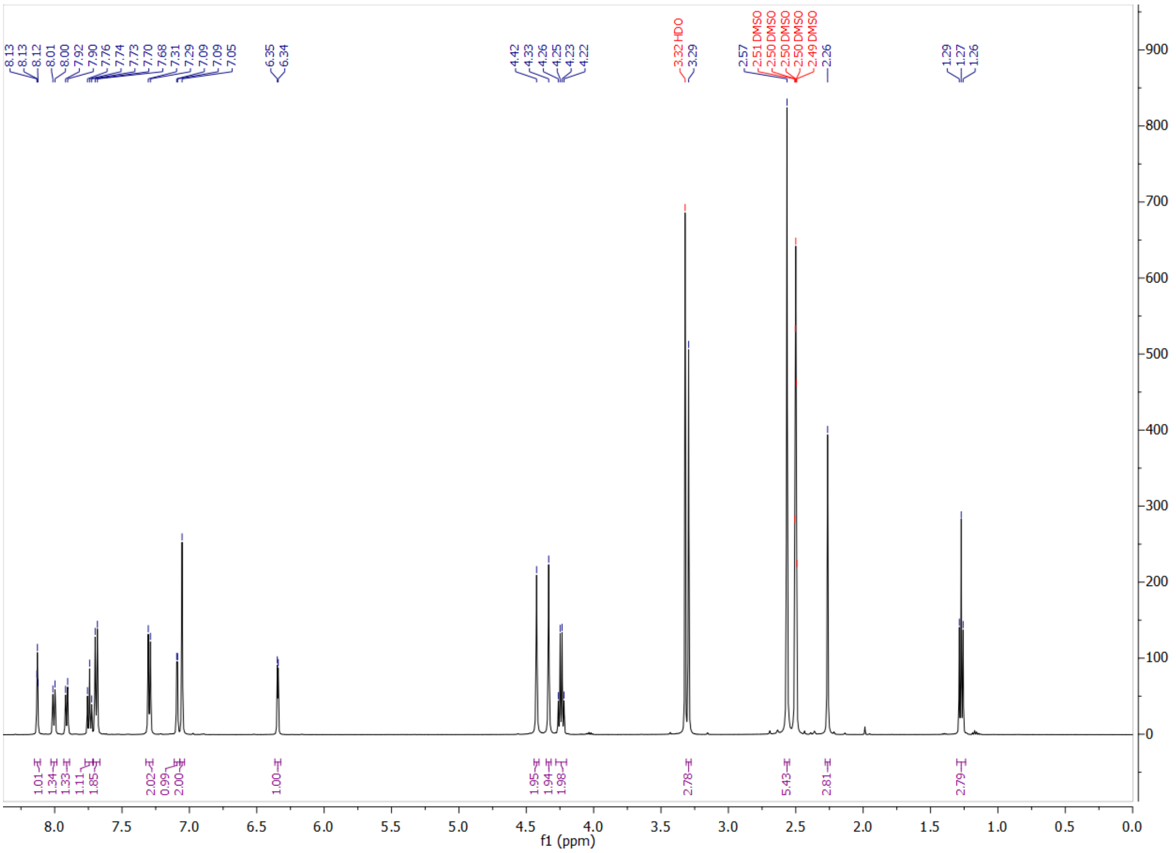

^{13}C NMR

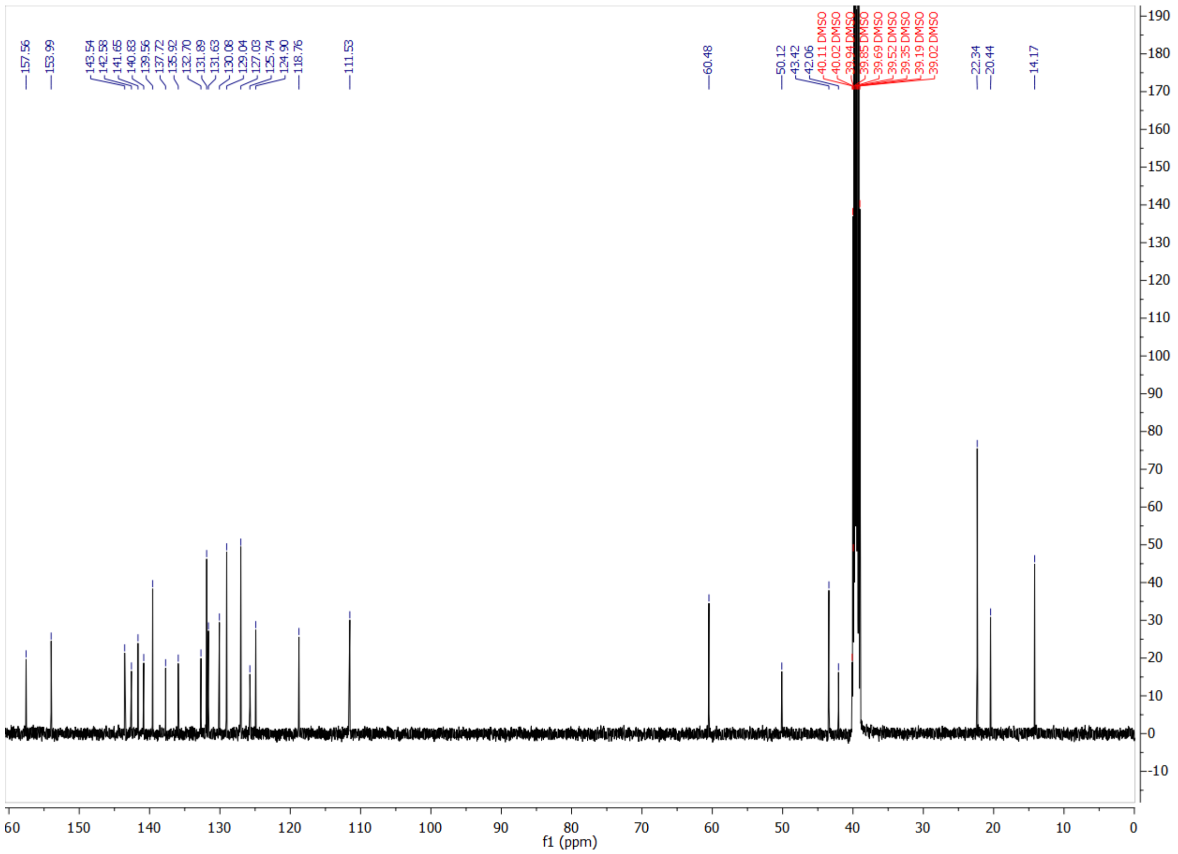

COMPOUND INFORMATION

Purity

Data File W:\analyti...PEN\CGC_wave3_1_FirstPassB 2023-01-04 18-28-02\074-D2F-G10-SR9238.D
Sample Name: SR9238

```
=====================================================================
Acq. Operator   : SYSTEM                         Seq. Line :  74
Sample Operator : SYSTEM
Acq. Instrument : LCMS test                       Location :  D2F-G10
Injection Date  : 1/5/2023 7:58:03 AM                  Inj :  1
                                                Inj Volume : Inj prog
Sequence File   : W:\analytical_LCMS_DATA\EUbOPEN\CGC_wave3_1_FirstPassB 2023-01-04 18-28-02
                  \CGC_wave3_1_FirstPassB.S
Method          : W:\analytical_LCMS_DATA\EUbOPEN\CGC_wave3_1_FirstPassB 2023-01-04 18-28-02
                  \CGL_FIRSTPASS_GENERALMETHOD_VIAL1+2_20210319.M (Sequence Method)
Last changed    : 1/25/2022 4:36:18 PM by SYSTEM
Method Info     : CGL wellplate, 0.5 uL of 10 mM DMSO, general method
```

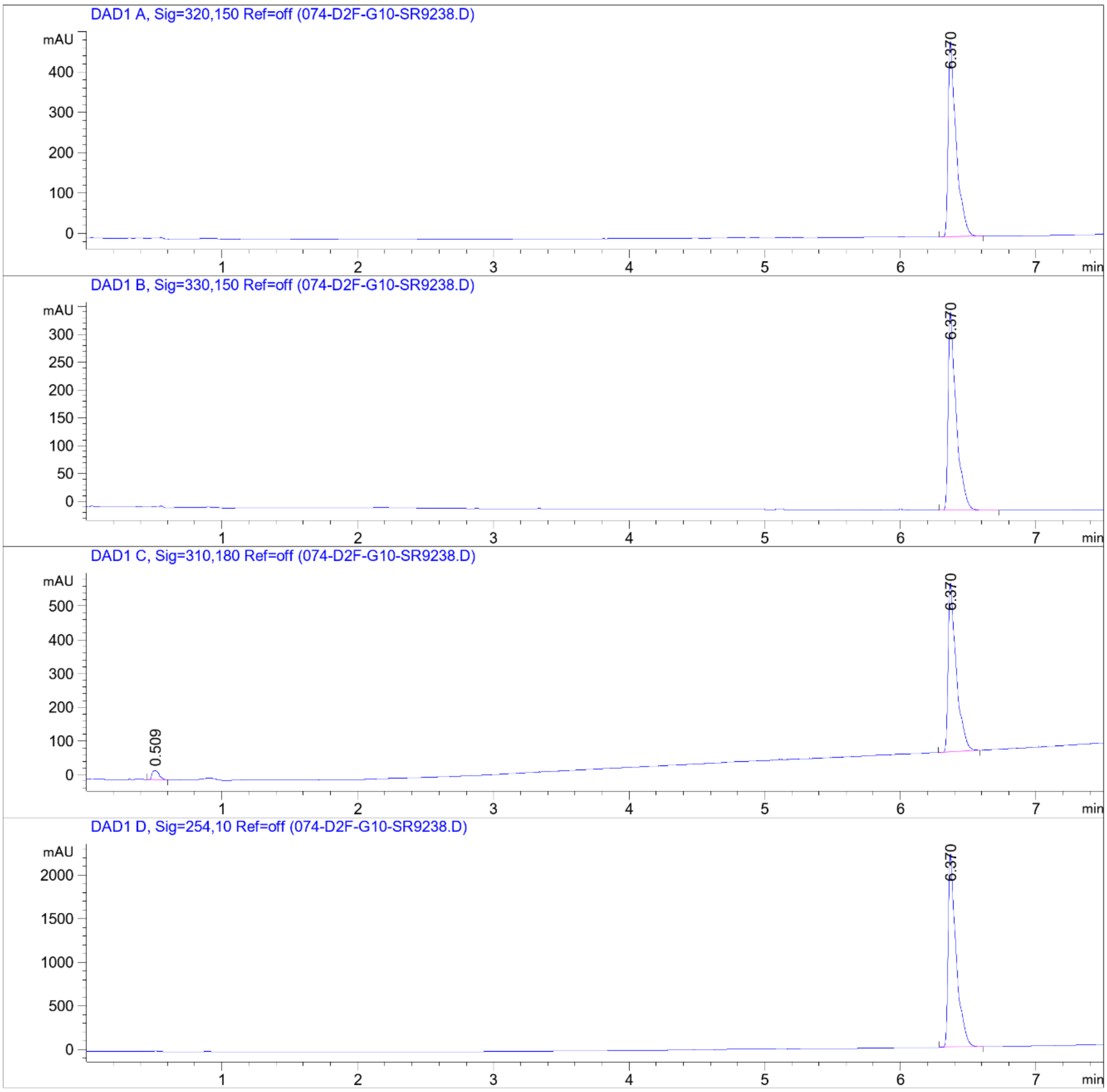

COMPOUND INFORMATION

Data File W:\analyti...PEN\CGC_wave3_1_FirstPassB 2023-01-04 18-28-02\074-D2F-G10-SR9238.D
Sample Name: SR9238

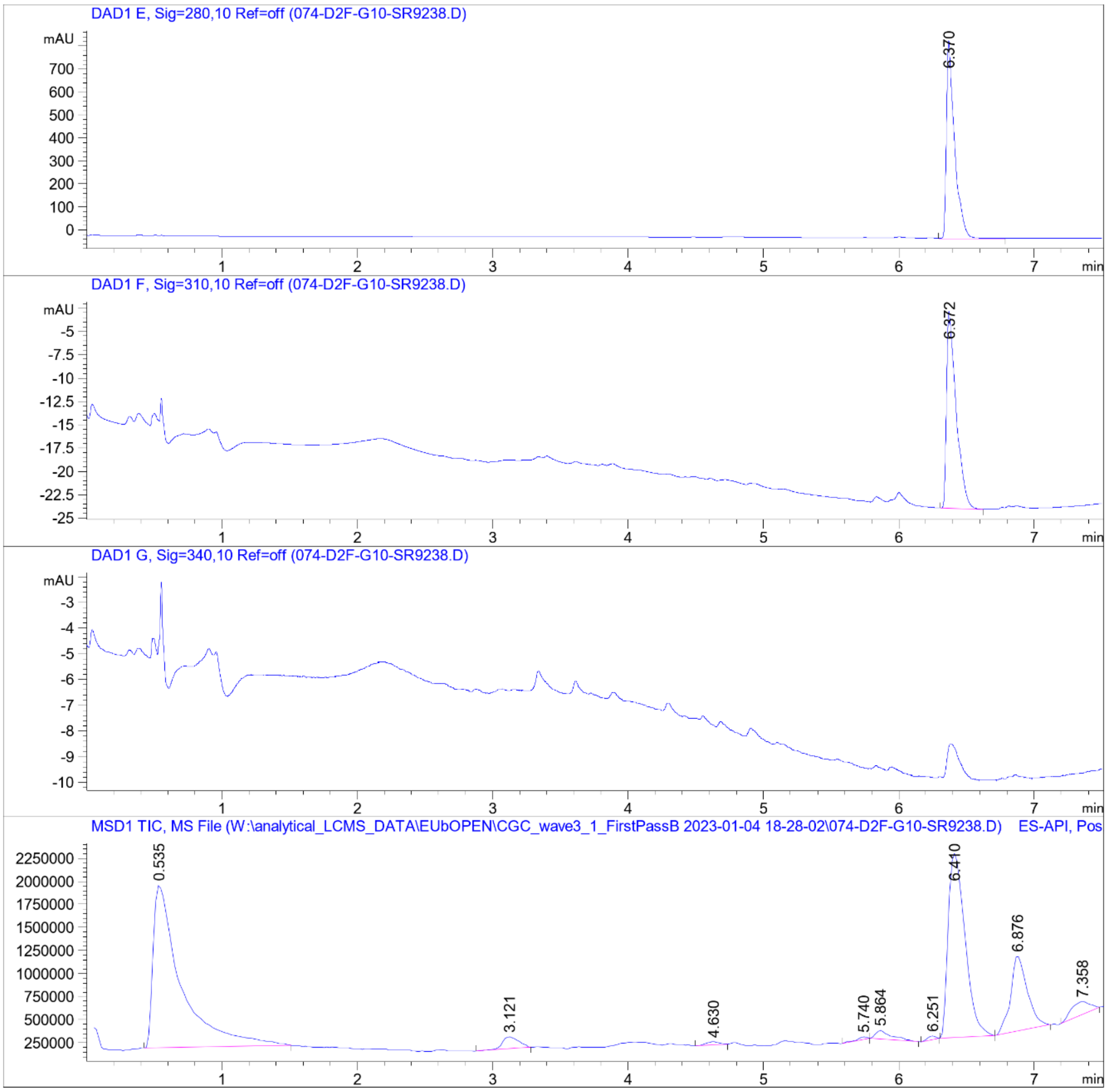

COMPOUND INFORMATION

Data File W:\analyti...PEN\CGC_wave3_1_FirstPassB 2023-01-04 18-28-02\074-D2F-G10-SR9238.D
Sample Name: SR9238

MS Signal: MSD1 TIC, MS File, ES-API, Pos, Scan, Frag: 70, "POS Scan"
Spectra from peak tops.
Noise Cutoff: 1000 counts.
Reportable Ion Abundance: > 50%.
LC Signal: DAD1 A, Sig=320,150 Ref=off
Peak matching window: 0.1 min

Retention Time (LC)	LC Area	Retention Time (MS)	MS Area	Mol. Weight or Ion
-	-	0.535	25013830	157.00 I
-	-	3.121	1090441	239.10 I
				217.10 I
-	-	4.630	197414	510.40 I
				170.90 I
				158.20 I
-	-	5.740	127908	280.20 I
-	-	5.864	766346	318.20 I
				296.20 I
-	-	6.251	149562	228.20 I
				137.10 I
6.370	1972	6.410	17302958	618.20 I
				596.20 I
-	-	6.876	6988443	282.20 I
-	-	7.358	1392870	400.30 I
				282.20 I

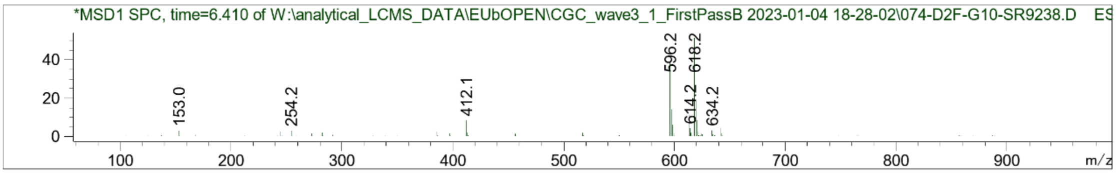

Supplement: Supplementary file 4 — Supplementary Data 1 [file 41467_2024_49493_MOESM4_ESM.zip › SR9238.pdf]
