# Supplementary material for: Chemogenomics for NR1 nuclear hormone receptors
Source: Nat Commun. 2024 Jun 18;15:5201. doi: 10.1038/s41467-024-49493-6 (PMC11189487; doi:10.1038/s41467-024-49493-6)

COMPOUND INFORMATION

EUbOPEN
Enabling & Unlocking Biology in the OPEN

T0901317

**CAS Registry No.:**	293754-55-9
**Formal Name:**	N-(4-(1,1,1,3,3,3-hexafluoro-2-hydroxypropan-2-yl)phenyl)-N-(2,2,2-trifluoroethyl)benzenesulfonamide
**EUbOPEN ID:**	EUB0000040b
**Molecular Formula:**	$C_{17}H_{12}F_9NO_3S$
**Molecular Weight:**	481.33 g/mol
**Smiles:**	O=S(C1=CC=CC=C1)(N(C2=CC=C(C(C(F)(F)F)(O)C(F)(F)F)C=C2)CC(F)(F)F)=O
**Recommended concentration:**	1 µM

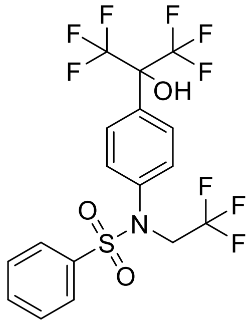

Biological activity

		Type	IC_{50}/EC_{50} [µM]	Reference
Main NR target:	NR1F3 (RORγ)	inv. Agonist	0.5	https://doi.org/10.1021/jm401901d
	NR1H3 (LXRα)	Agonist	0.4	
	NR1H2 (LXRβ)	Agonist	0.2	
	NR1I2 (PXR)	Agonist	0.04	
NR off-target:	NR1H4 (FXR)	Agonism	1.3	https://doi.org/10.1021/jm401901d

COMPOUND INFORMATION

Identity

^{1}H NMR

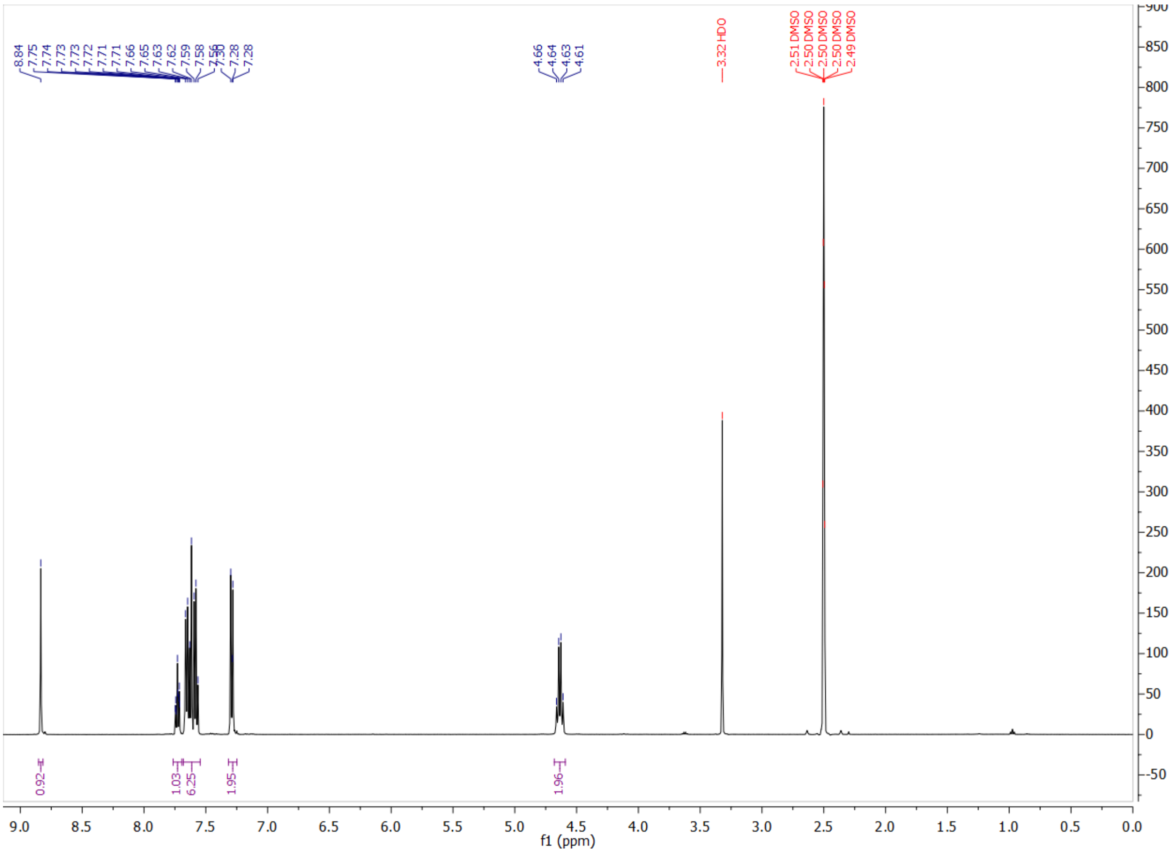

^{13}C NMR

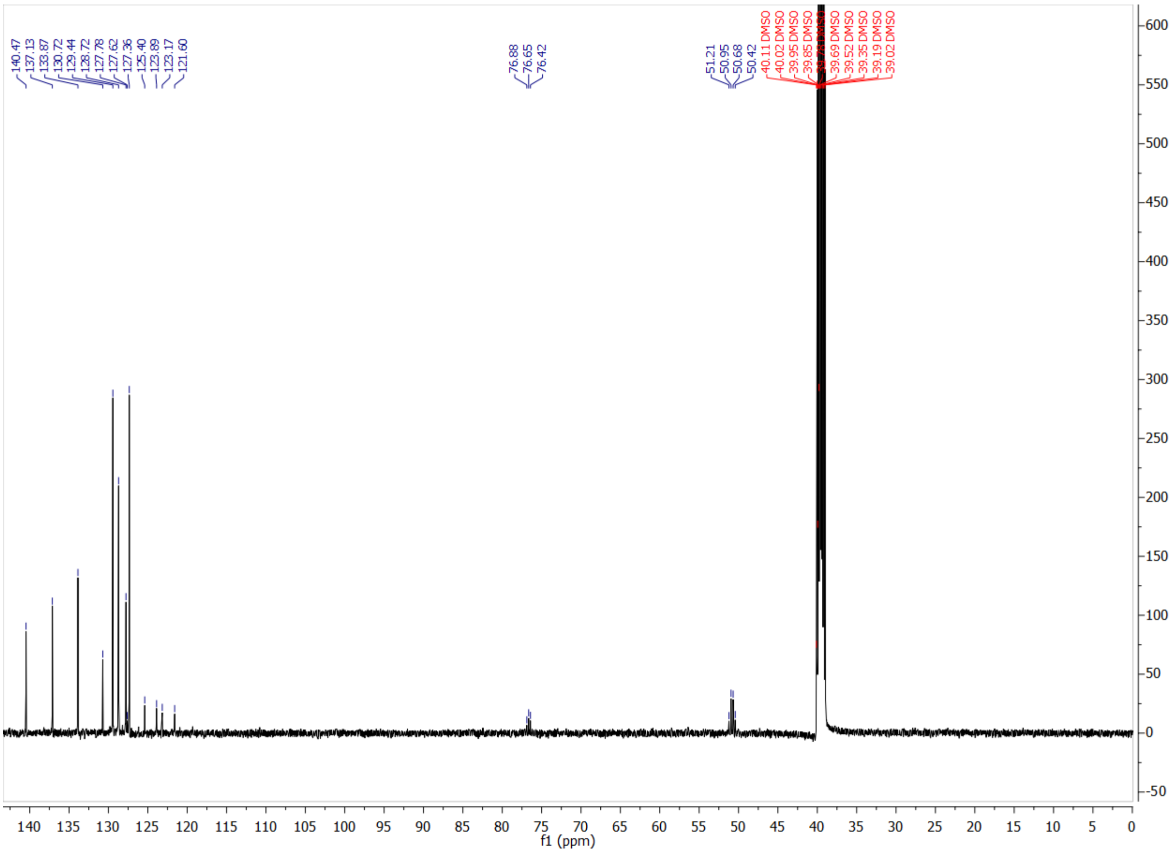

COMPOUND INFORMATION

Purity

Data File W:\analyti...N\CGC_wave2_4_FirstPassB 2022-02-18 17-58-18\082-D2F-G11-T0901317.D
Sample Name: T0901317

```
=====================================================================
Acq. Operator   : SYSTEM                          Seq. Line :  82
Sample Operator : SYSTEM
Acq. Instrument : LCMS test                        Location :   D2F-G11
Injection Date  : 2/19/2022 8:56:50 AM                  Inj :   1
                                                Inj Volume : Inj prog
Sequence File   : W:\analytical_LCMS_DATA\EUbOPEN\CGC_wave2_4_FirstPassB 2022-02-18 17-58-18
                  \CGC_wave2_4_FirstPassB.S
Method          : W:\analytical_LCMS_DATA\EUbOPEN\CGC_wave2_4_FirstPassB 2022-02-18 17-58-18
                  \CGL_FIRSTPASS_GENERALMETHOD_VIAL1+2_20210319.M (Sequence Method)
Last changed    : 1/25/2022 4:36:18 PM by SYSTEM
Method Info     : CGL wellplate, 0.5 uL of 10 mM DMSO, general method
```

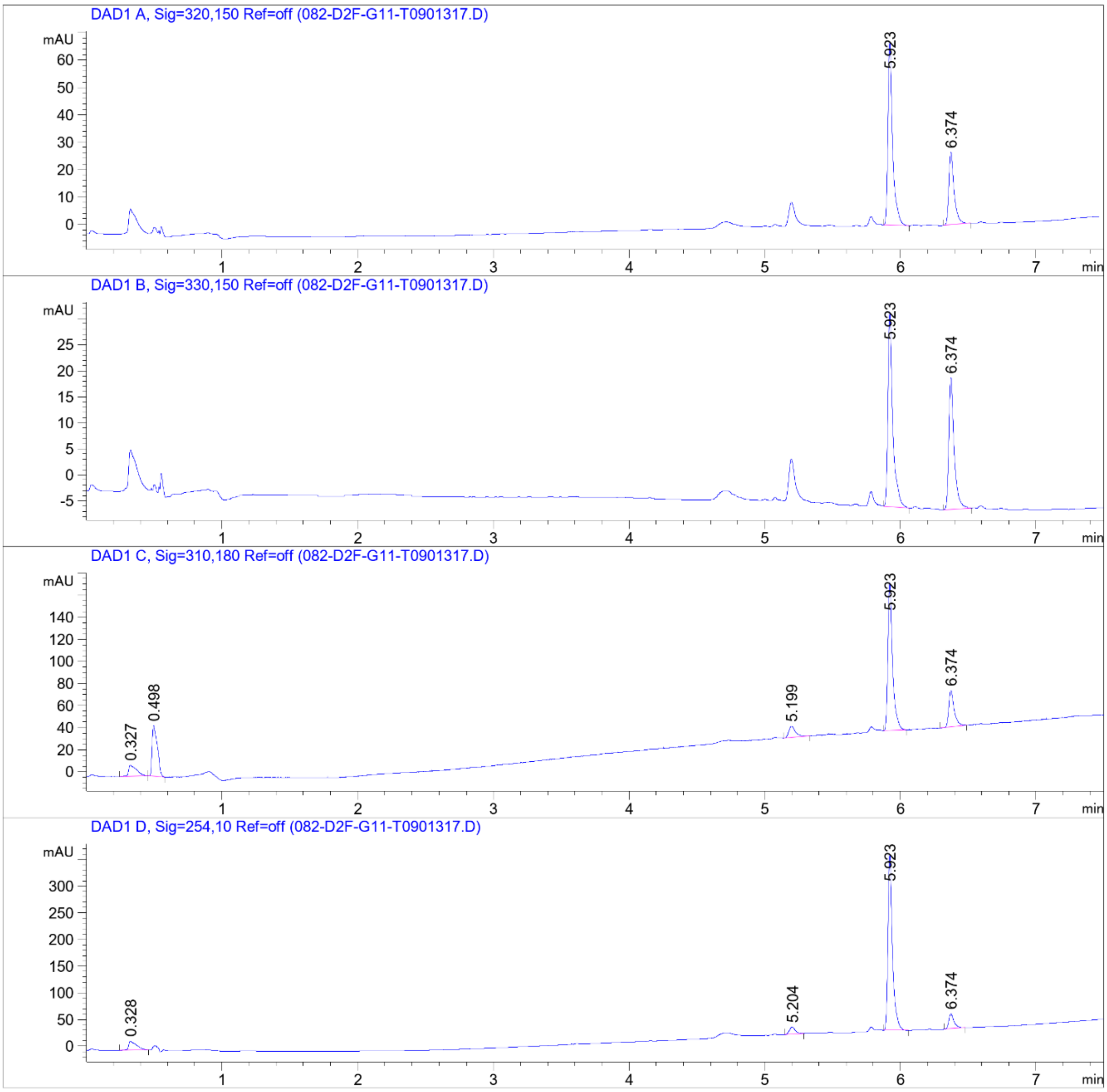

COMPOUND INFORMATION

Data File W:\analyti...N\CGC_wave2_4_FirstPassB 2022-02-18 17-58-18\082-D2F-G11-T0901317.D
Sample Name: T0901317

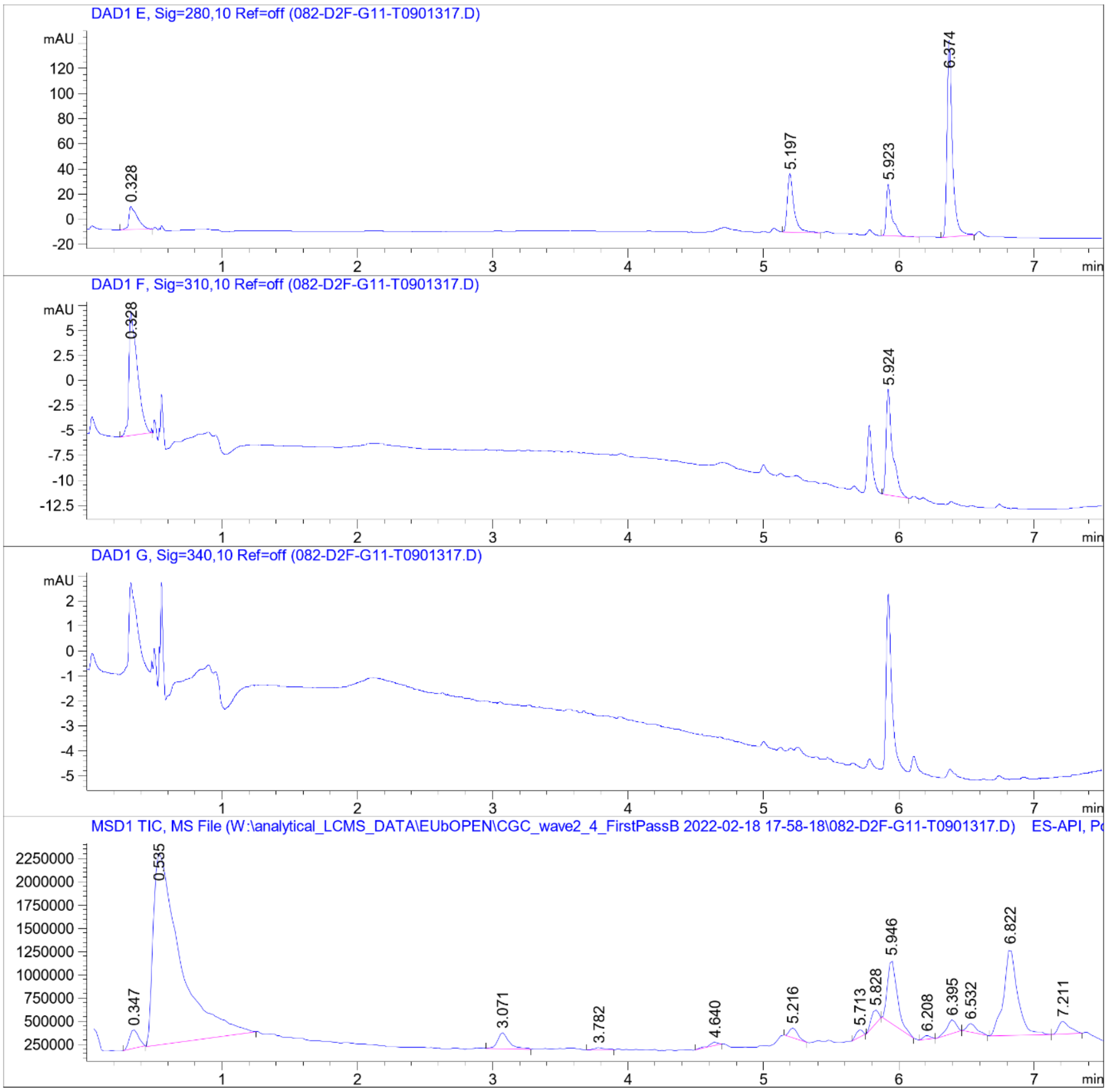

COMPOUND INFORMATION

Data File W:\analyti...N\CGC_wave2_4_FirstPassB 2022-02-18 17-58-18\082-D2F-G11-T0901317.D
Sample Name: T0901317

MS Signal: MSD1 TIC, MS File, ES-API, Pos, Scan, Frag: 70, "POS Scan"
Spectra from peak tops.
Noise Cutoff: 1000 counts.
Reportable Ion Abundance: > 50%.
LC Signal: DAD1 A, Sig=320,150 Ref=off
Peak matching window: 0.1 min

Retention Time (LC)	LC Area	Retention Time (MS)	MS Area	Mol. Weight or Ion
-	-	0.347	917991	199.90 I
				182.90 I
				158.90 I
				158.00 I
				141.90 I
				101.00 I
-	-	0.535	28780390	157.00 I
-	-	3.071	989882	239.00 I
				217.00 I
-	-	3.782	116894	274.20 I
-	-	4.640	176651	332.20 I
-	-	5.216	454801	338.20 I
				316.20 I
-	-	5.713	251208	280.20 I
-	-	5.828	547018	318.20 I
				296.20 I
5.923	161	5.946	3399445	482.00 I
-	-	6.208	146868	228.10 I
6.374	72	6.395	700252	350.30 I
				282.20 I
				254.20 I
				243.00 I
-	-	6.532	449043	507.20 I
				485.20 I
				280.20 I
-	-	6.822	6657310	282.20 I
-	-	7.211	806416	284.20 I
				282.20 I

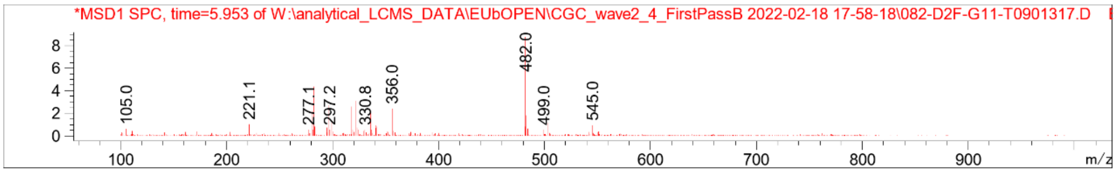

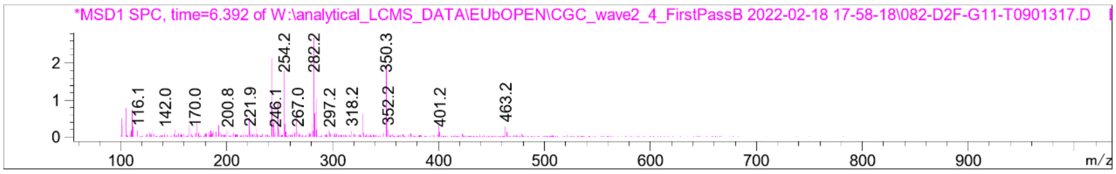

Supplement: Supplementary file 4 — Supplementary Data 1 [file 41467_2024_49493_MOESM4_ESM.zip › T0901317.pdf]
