# Supplementary material for: Chemogenomics for NR1 nuclear hormone receptors
Source: Nat Commun. 2024 Jun 18;15:5201. doi: 10.1038/s41467-024-49493-6 (PMC11189487; doi:10.1038/s41467-024-49493-6)

COMPOUND INFORMATION

T3 (3,3´,5-Triiodo-L-thyronine)

**CAS Registry No.:**	6893-02-3
**Formal Name:**	(S)-2-amino-3-(4-(4-hydroxy-3-iodophenoxy)-3,5-diiodophenyl)propanoic acid
**EUbOPEN ID:**	EUB0000555a
**Molecular Formula:**	$C_{15}H_{12}I_3NO_4$
**Molecular Weight:**	650.98 g/mol
**Smiles:**	C1=CC(=C(C=C1OC2=C(C=C(C=C2I)C[C@@H](C(=O)O)N)I)I)O
**Recommended concentration:**	1 µM

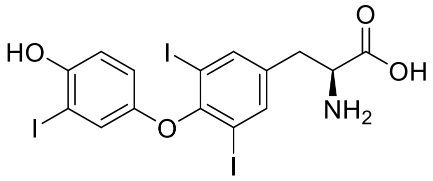

Biological activity

		Type	IC_{50}/EC_{50} [µM]	Reference
Main NR target:	NR1A1 (THRα)	Agonist	0.002	https://doi.org/10.1016/j.bmc.2007.10.040, https://doi.org/10.1021/jm0201013
	NR1A2 (THRβ)	Agonist	0.002	
NR off-target:				

COMPOUND INFORMATION

Identity

^{1}H NMR

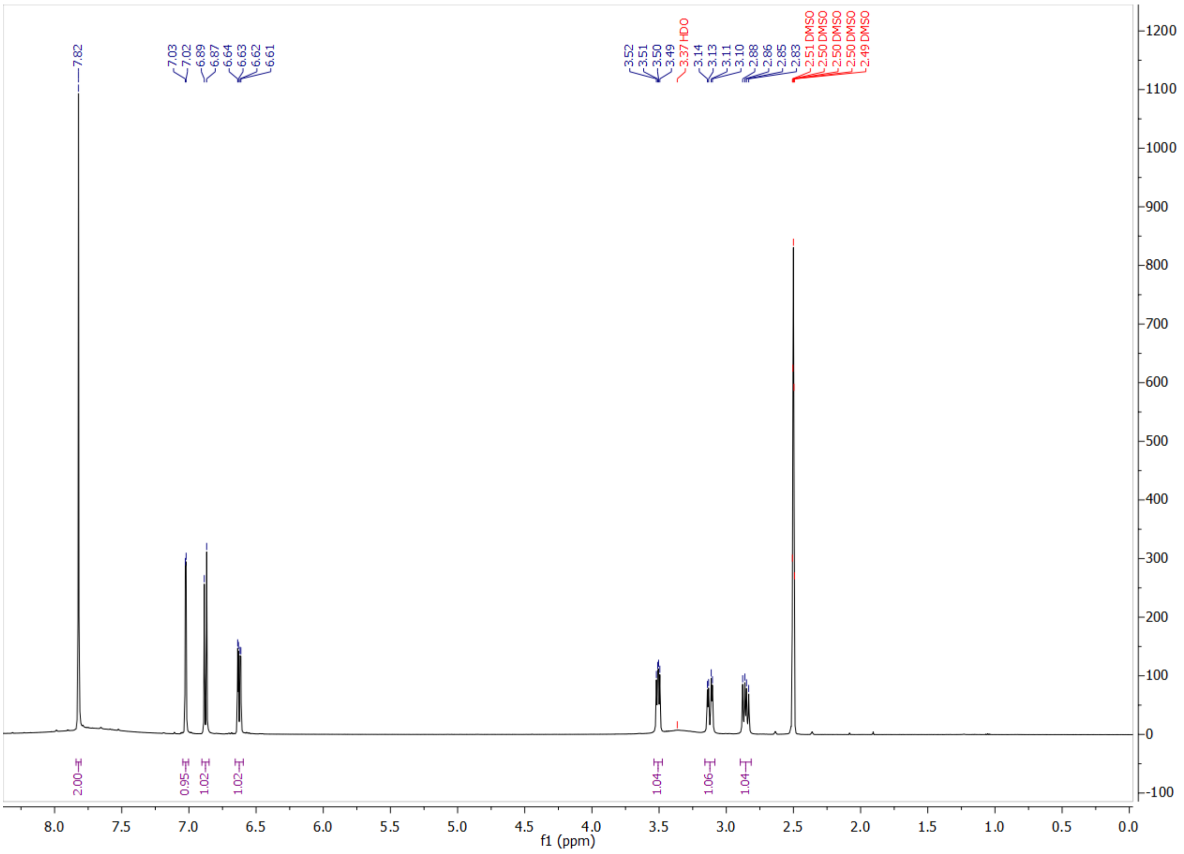

^{13}C NMR

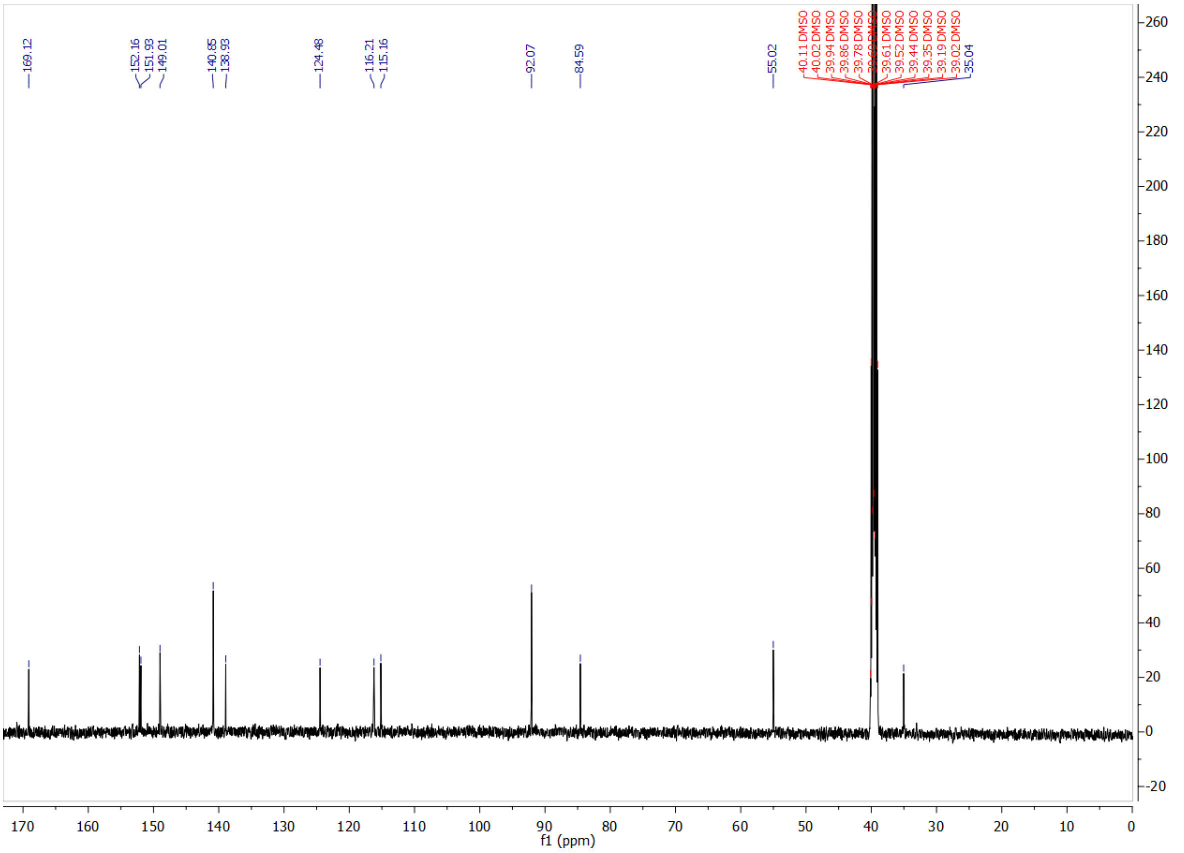

COMPOUND INFORMATION

Purity

Data File W:\analyti...\EUbOPEN\CGC_ECHO1-2_SecondPass 2021-03-23 18-53-53\023-D2F-G1-T3.D
Sample Name: T3

===
Acq. Operator : SYSTEM Seq. Line : 23
Sample Operator : SYSTEM
Acq. Instrument : LCMS test Location : D2F-G1
Injection Date : 3/23/2021 11:01:33 PM Inj : 1
Inj Volume : Inj prog
Sequence File : W:\analytical_LCMS_DATA\EUbOPEN\CGC_ECHO1-2_SecondPass 2021-03-23 18-53-53\CGC_ECHO1-2_SecondPass.S
Method : W:\analytical_LCMS_DATA\EUbOPEN\CGC_ECHO1-2_SecondPass 2021-03-23 18-53-53\CGL_SECONDPASS_NONPOLCOMP_VIAL2+4_20210323.M (Sequence Method)
Last changed : 3/23/2021 6:50:05 PM by SYSTEM
Method Info : CGL wellplate, 0.5 uL of 10 mM DMSO. Dilution with MeCN only (9+9 uL)

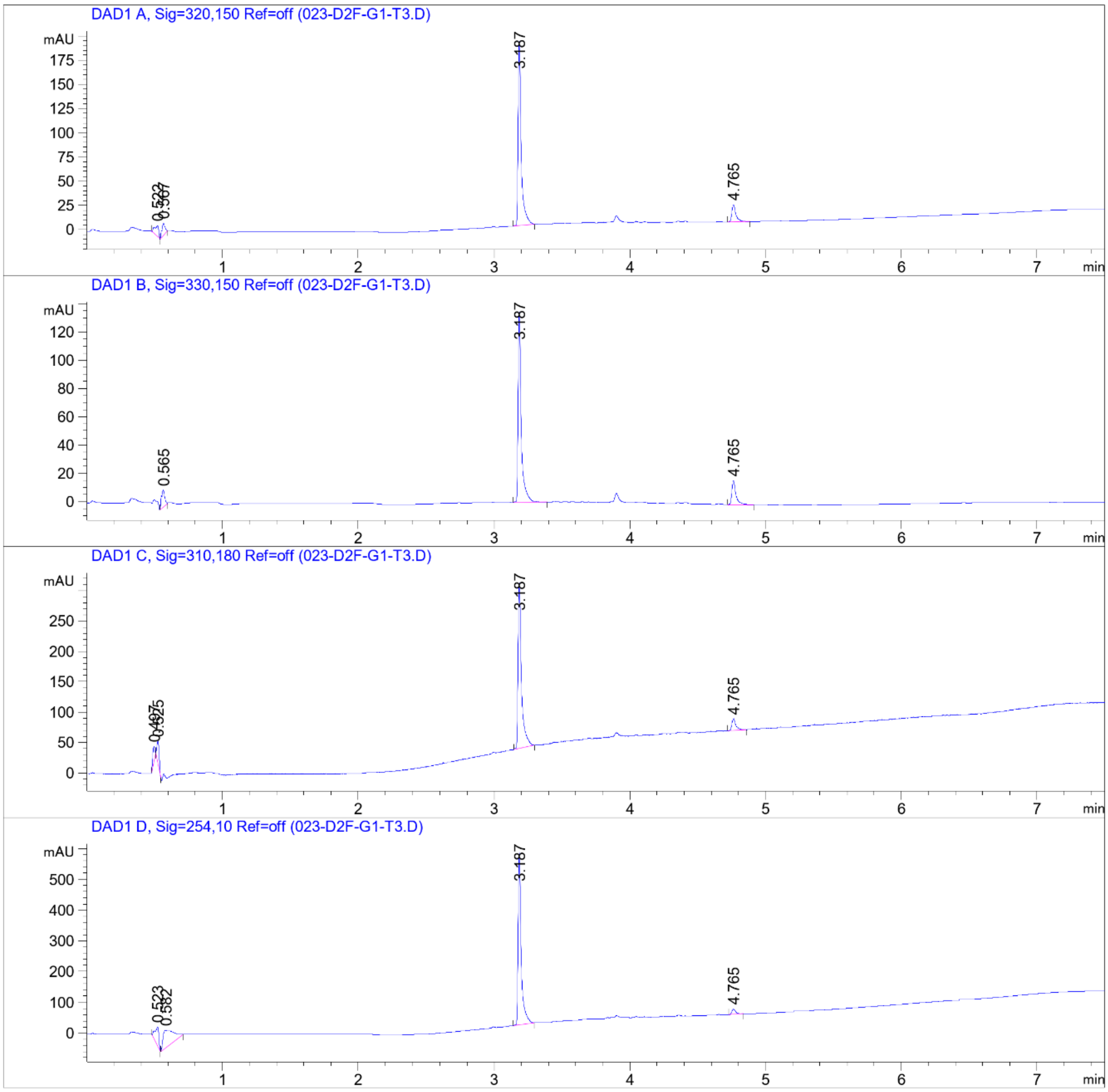

COMPOUND INFORMATION

Data File W:\analyti...\EUbOPEN\CGC_ECHO1-2_SecondPass 2021-03-23 18-53-53\023-D2F-G1-T3.D
Sample Name: T3

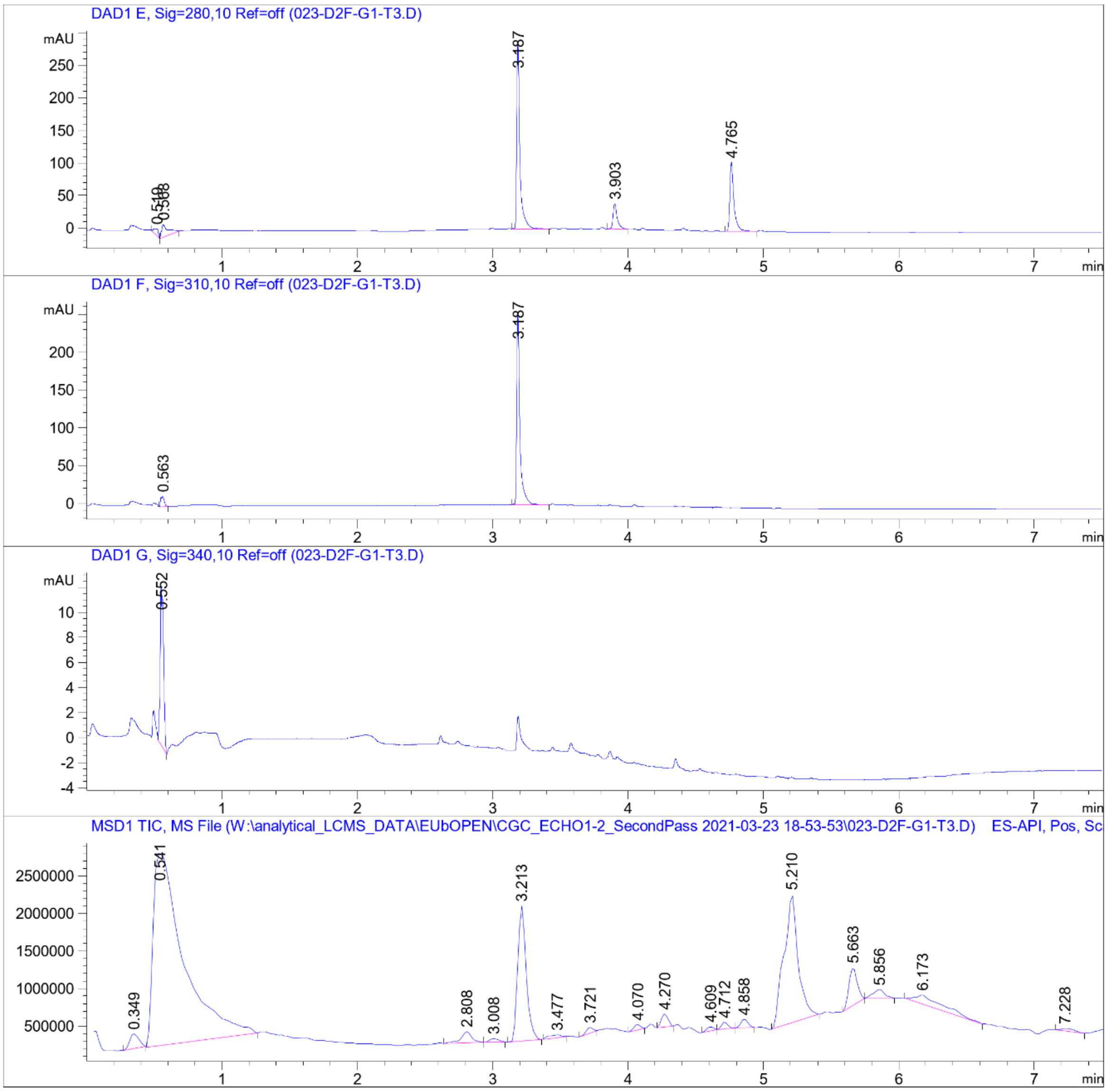

COMPOUND INFORMATION

Data File W:\analyti...\EUbOPEN\CGC_ECHO1-2_SecondPass 2021-03-23 18-53-53\023-D2F-G1-T3.D
Sample Name: T3

MS Signal: MSD1 TIC, MS File, ES-API, Pos, Scan, Frag: 70, "POS Scan"
Spectra from peak tops.
Noise Cutoff: 1000 counts.
Reportable Ion Abundance: > 50%.
LC Signal: DAD1 A, Sig=320,150 Ref=off
Peak matching window: 0.1 min

Retention Time (LC)	LC Area	Retention Time (MS)	MS Area	Mol. Weight or Ion
-	-	0.349	920387	200.00 I
				183.10 I
				159.00 I
				142.00 I
				111.10 I
				110.10 I
0.522	23	0.541	39819696	157.10 I
0.567	20	-	-	
-	-	2.808	787438	217.10 I
-	-	3.008	213253	525.90 I
				274.30 I
				200.00 I
				158.90 I
				157.10 I
3.187	303	3.213	7789105	651.90 I
-	-	3.477	261675	232.10 I
-	-	3.721	263599	214.10 I
-	-	4.070	235858	216.10 I
-	-	4.270	594019	296.30 I
-	-	4.609	200004	280.30 I
				228.20 I
4.765	37	4.712	282520	254.30 I
-	-	4.858	393240	280.30 I
-	-	5.210	12456643	282.30 I
-	-	5.663	2142663	359.30 I
				341.30 I
				284.30 I
				282.20 I
-	-	5.856	623589	400.30 I
				282.30 I
-	-	6.173	1855474	282.30 I
-	-	7.228	249470	102.20 I

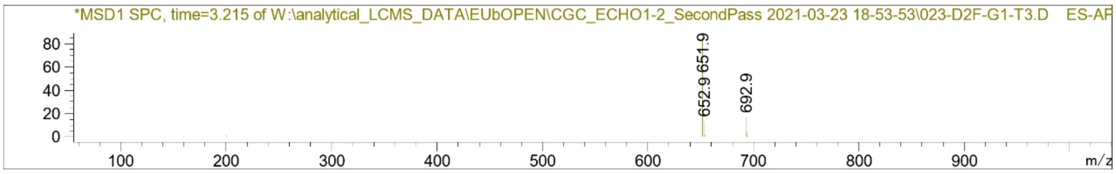

Supplement: Supplementary file 4 — Supplementary Data 1 [file 41467_2024_49493_MOESM4_ESM.zip › T3.pdf]
