# Supplementary material for: Chemogenomics for NR1 nuclear hormone receptors
Source: Nat Commun. 2024 Jun 18;15:5201. doi: 10.1038/s41467-024-49493-6 (PMC11189487; doi:10.1038/s41467-024-49493-6)

COMPOUND INFORMATION

Tamibarotene (AM80)

**CAS Registry No.:**	94497-51-5
**Formal Name:**	4-((5,5,8,8-tetramethyl-5,6,7,8-tetrahydronaphthalen-2-yl)carbamoyl)benzoic acid
**EUbOPEN ID:**	EUB0000566a
**Molecular Formula:**	$C_{22}H_{25}NO_3$
**Molecular Weight:**	351.45 g/mol
**Smiles:**	CC1(CCC(C2=C1C=CC(=C2)NC(=O)C3=CC=C(C=C3)C(=O)O)(C)C)C
**Recommended concentration:**	1 µM

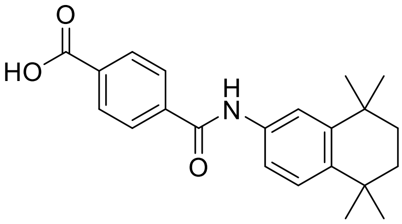

Biological activity

		Type	IC_{50}/EC_{50} [µM]	Reference
Main NR target:	NR1B1 (RARα)	Agonist	0.05	https://doi.org/10.1021/jm100189a
	NR1B2 (RARβ)	Agonist	0.2	
	NR1B3 (RARγ)	Agonist	0.6	
NR off-target:				

COMPOUND INFORMATION

Identity

^{1}H NMR

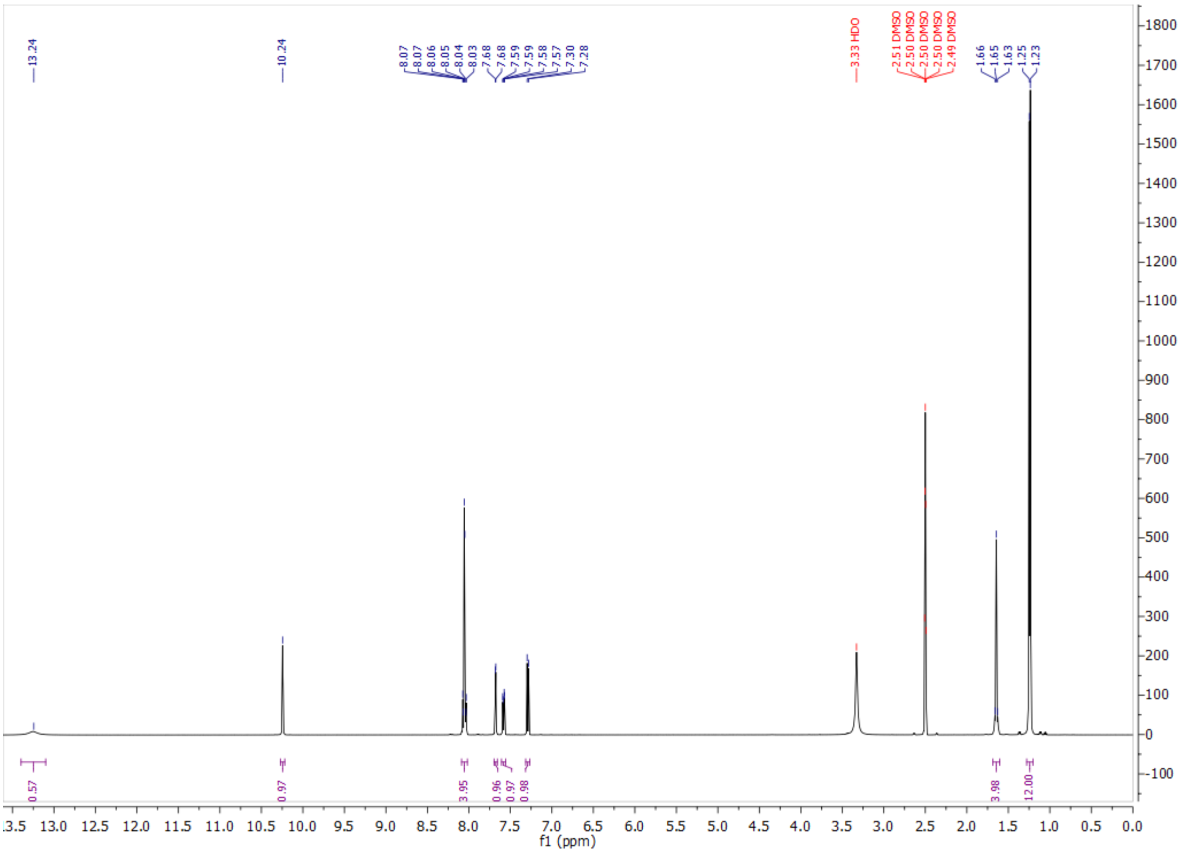

^{13}C NMR

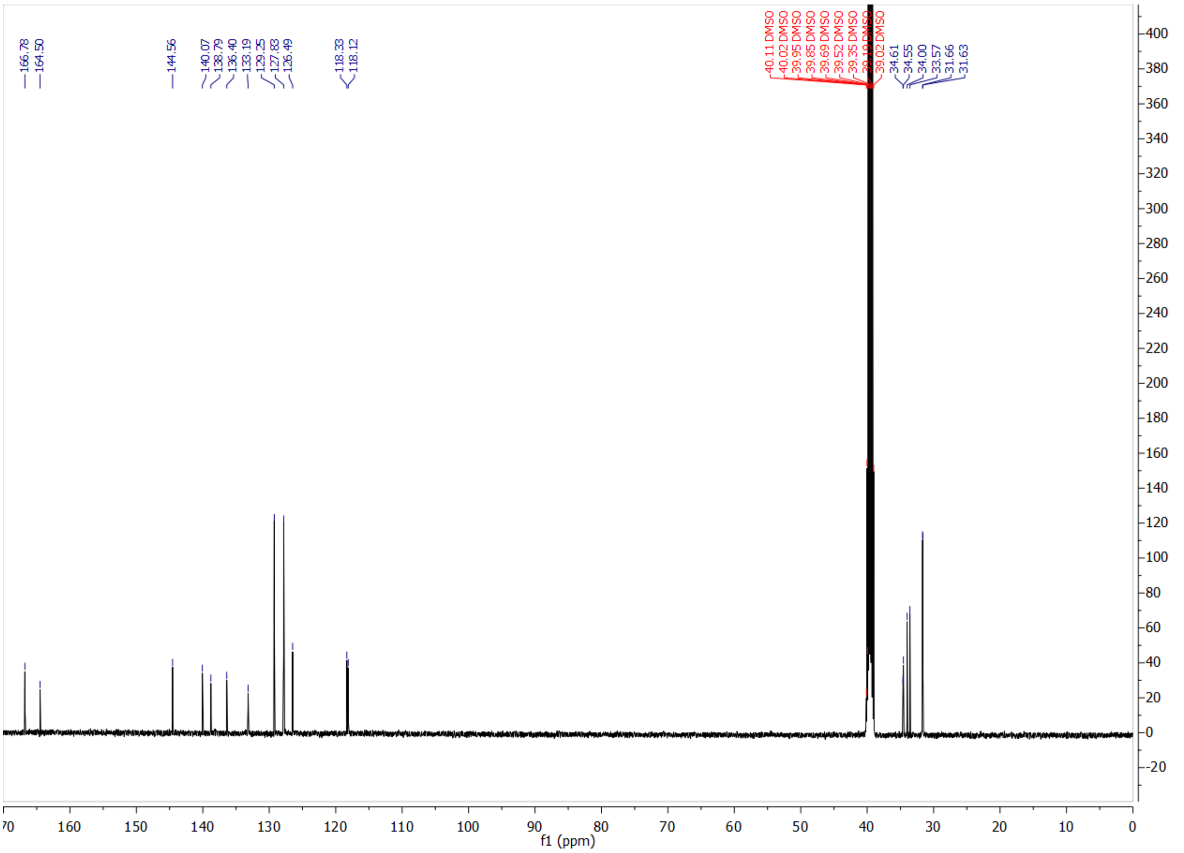

COMPOUND INFORMATION

Purity

Data File W:\analyti...CGC_ECHO1-3_FirstPass 2021-03-20 13-21-54\013-D2B-B1-tamibarotene.D
Sample Name: tamibarotene

```
=====================================================================
Acq. Operator   : SYSTEM                          Seq. Line :  13
Sample Operator : SYSTEM
Acq. Instrument : LCMS test                        Location :   D2B-B1
Injection Date  : 3/20/2021 3:38:37 PM                  Inj :   1
                                                 Inj Volume : Inj prog
Sequence File   : W:\analytical_LCMS_DATA\EUbOPEN\CGC_ECHO1-3_FirstPass 2021-03-20 13-21-54
                  \CGC_ECHO1-3_FirstPass.S
Method          : W:\analytical_LCMS_DATA\EUbOPEN\CGC_ECHO1-3_FirstPass 2021-03-20 13-21-54
                  \CGL_FIRSTPASS_GENERALMETHOD_VIAL3+4_20210319.M (Sequence Method)
Last changed    : 3/19/2021 5:35:24 PM by SYSTEM
Method Info     : CGL wellplate, 0.5 uL of 10 mM DMSO, general method
```

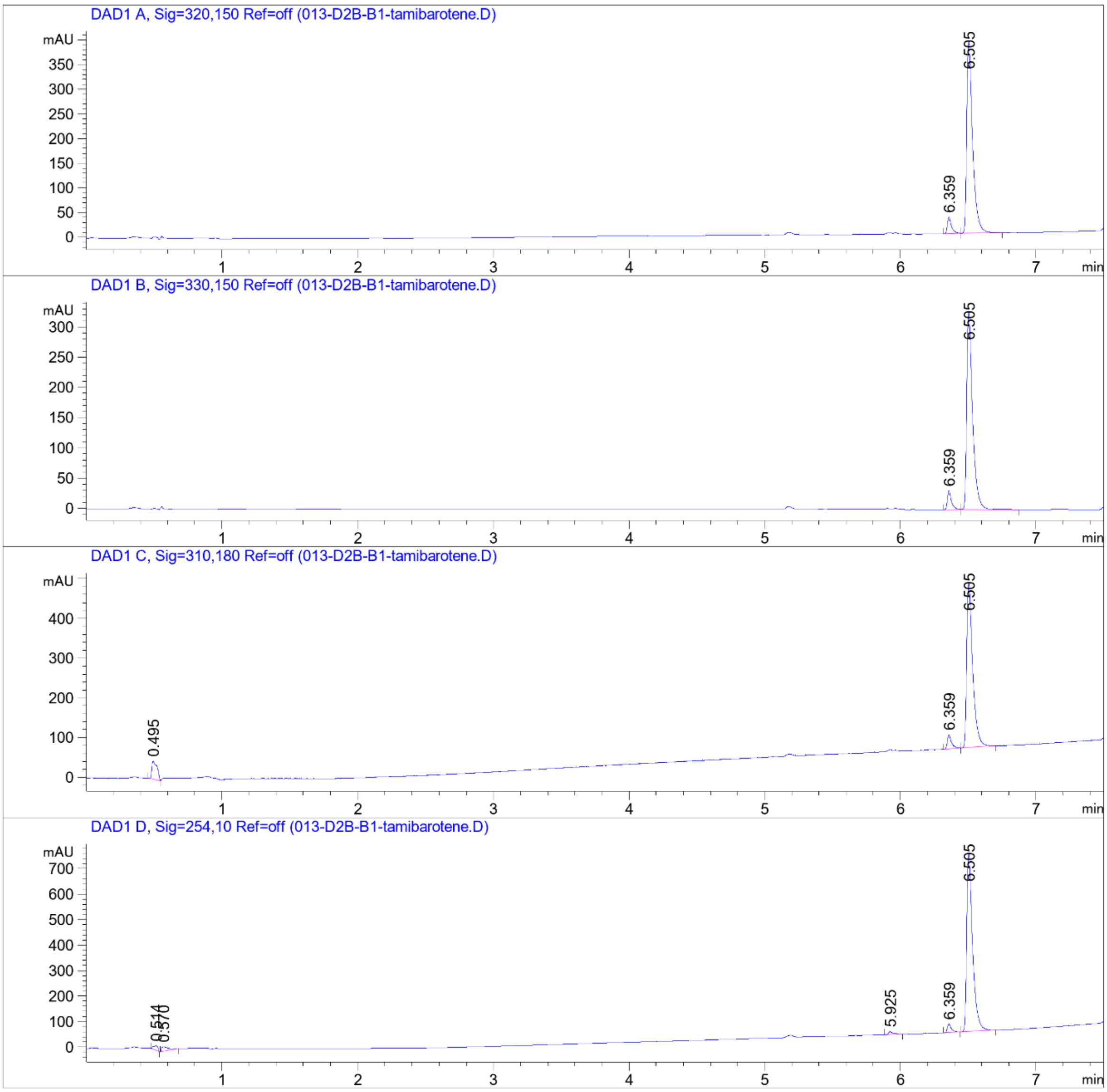

COMPOUND INFORMATION

Data File W:\analyti...CGC_ECHO1-3_FirstPass 2021-03-20 13-21-54\013-D2B-B1-tamibarotene.D
Sample Name: tamibarotene

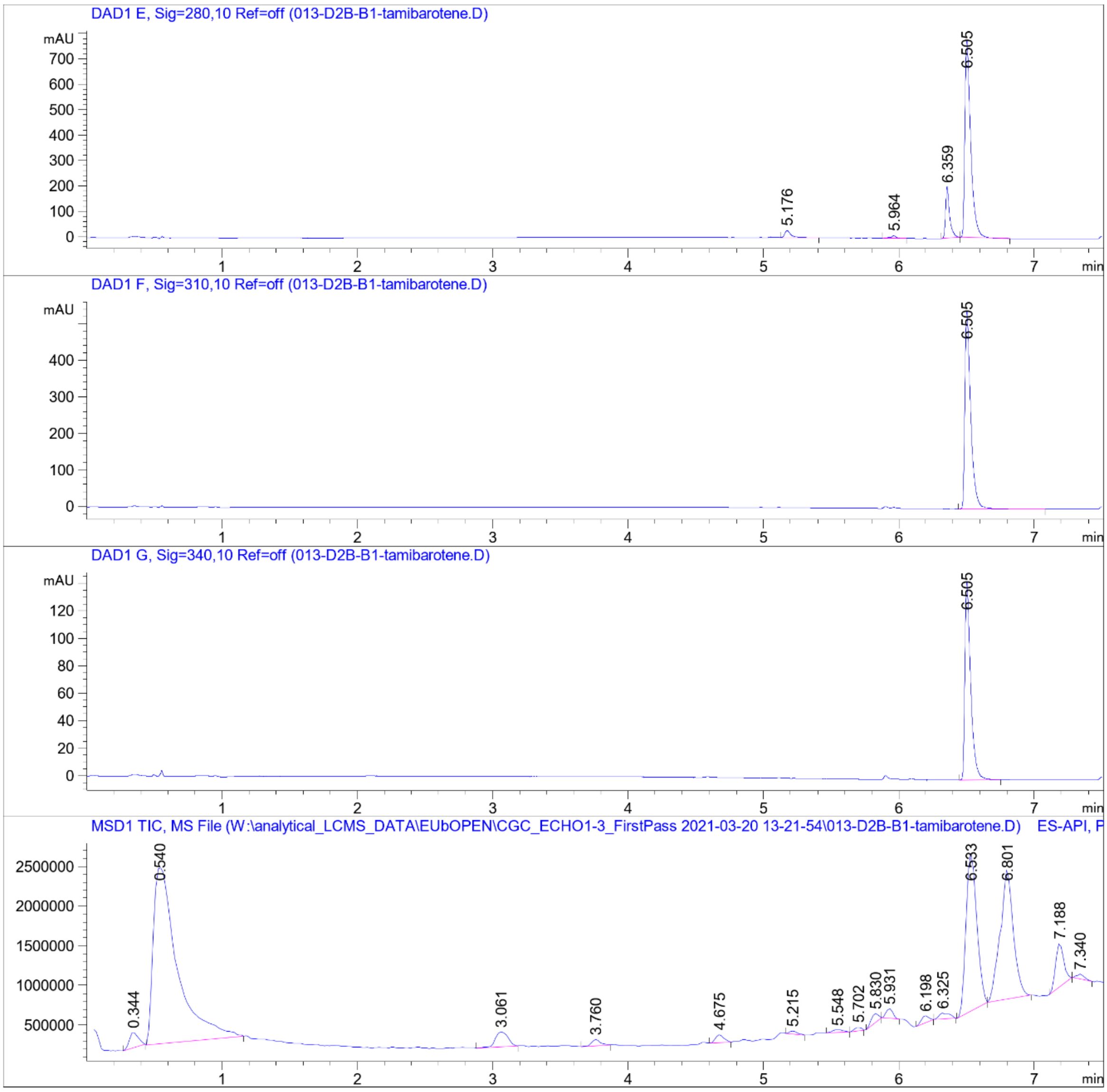

COMPOUND INFORMATION

Data File W:\analyti...CGC_ECHO1-3_FirstPass 2021-03-20 13-21-54\013-D2B-B1-tamibarotene.D
Sample Name: tamibarotene

MS Signal: MSD1 TIC, MS File, ES-API, Pos, Scan, Frag: 70, "POS Scan"
Spectra from peak tops.
Noise Cutoff: 1000 counts.
Reportable Ion Abundance: > 50%.
LC Signal: DAD1 A, Sig=320,150 Ref=off
Peak matching window: 0.1 min

Retention Time (LC)	LC Area	Retention Time (MS)	MS Area	Mol. Weight or Ion
-	-	0.344	900849	158.00 I
-	-	0.540	27320490	157.00 I
-	-	3.061	1157963	217.00 I
-	-	3.760	370769	274.20 I
-	-	4.675	441213	326.30 I
-	-	5.215	170311	316.30 I
				298.20 I
				296.20 I
				200.00 I
				158.90 I
				111.00 I
				102.10 I
-	-	5.548	168250	326.20 I
				280.20 I
				102.20 I
-	-	5.702	168069	280.20 I
-	-	5.830	419190	296.20 I
-	-	5.931	414708	296.20 I
				294.20 I
				280.20 I
-	-	6.198	336896	280.20 I
				228.20 I
6.359	69	6.325	405567	254.20 I
6.505	1163	6.533	10910815	352.10 I
-	-	6.801	10984832	282.30 I
-	-	7.188	2311659	284.30 I
				282.20 I
-	-	7.340	244243	400.30 I
				282.20 I

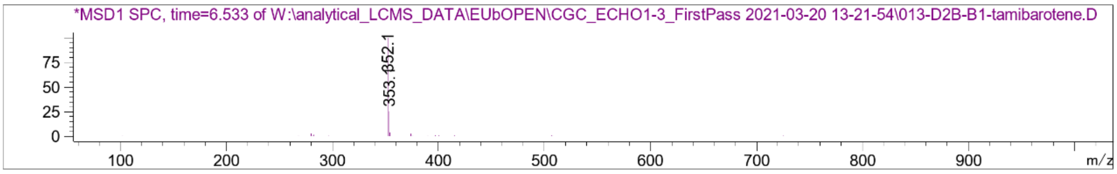

Supplement: Supplementary file 4 — Supplementary Data 1 [file 41467_2024_49493_MOESM4_ESM.zip › Tamibarotene.pdf]
