# Supplementary material for: Chemogenomics for NR1 nuclear hormone receptors
Source: Nat Commun. 2024 Jun 18;15:5201. doi: 10.1038/s41467-024-49493-6 (PMC11189487; doi:10.1038/s41467-024-49493-6)

COMPOUND INFORMATION

Tazarotene

**CAS Registry No.:**	118292-40-3
**Formal Name:**	Ethyl 6-((4,4-dimethylthiochroman-6-yl)ethynyl)nicotinate
**EUbOPEN ID:**	EUB0000563a
**Molecular Formula:**	$C_{21}H_{21}NO_2S$
**Molecular Weight:**	351.46 g/mol
**Smiles:**	CCOC(=O)C1=CN=C(C=C1)C#CC2=CC3=C(C=C2)SCCC3(C)C
**Recommended concentration:**	1 µM

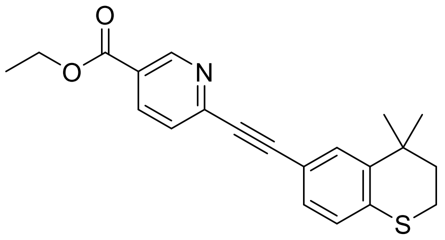

Biological activity

		Type	IC_{50}/EC_{50} [µM]	Reference
Main NR target:	NR1B1 (RARα)	Agonist	0.06	https://doi.org/10.1016/j.bmcl.2008.11.040
	NR1B2 (RARβ)	Agonist	0.0008	
	NR1B3 (RARγ)	Agonist	0.04	
NR off-target:				

COMPOUND INFORMATION

Identity

^{1}H NMR

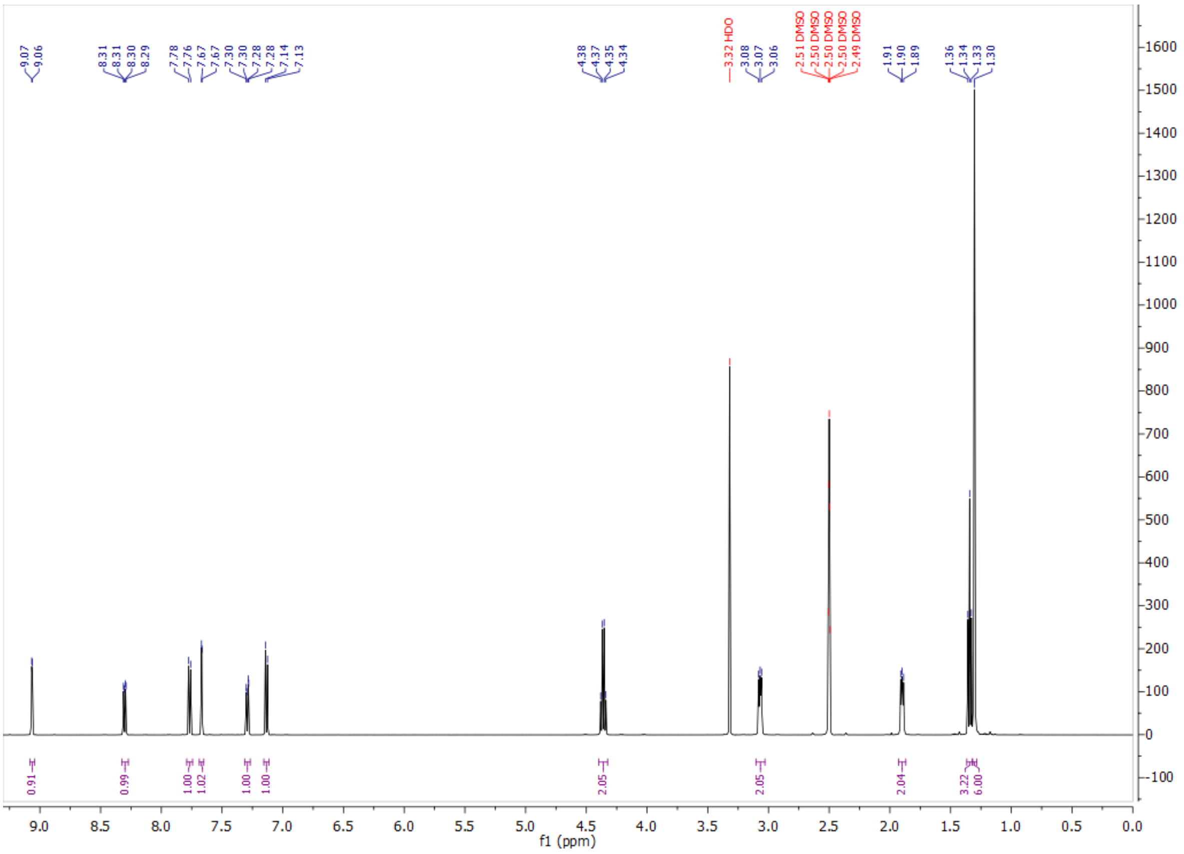

^{13}C NMR

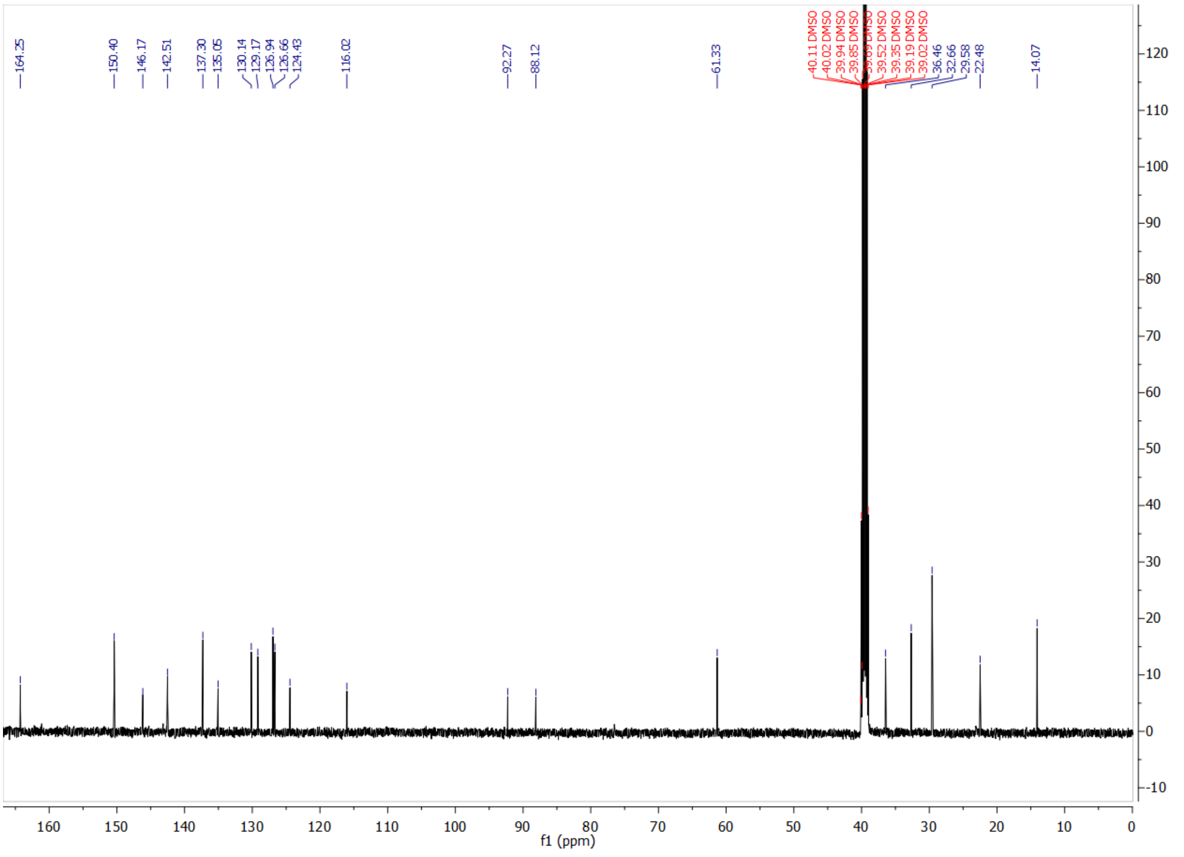

COMPOUND INFORMATION

Purity

Data File W:\analyti...N\CGC_ECHO1-3_FirstPass 2021-03-20 13-21-54\024-D2B-B9-tazarotene.D
Sample Name: tazarotene

```
=====================================================================
Acq. Operator   : SYSTEM                          Seq. Line :  24
Sample Operator : SYSTEM
Acq. Instrument : LCMS test                        Location :   D2B-B9
Injection Date  : 3/20/2021 5:40:31 PM                  Inj :   1
                                                Inj Volume : Inj prog
Sequence File   : W:\analytical_LCMS_DATA\EUbOPEN\CGC_ECHO1-3_FirstPass 2021-03-20 13-21-54
                  \CGC_ECHO1-3_FirstPass.S
Method          : W:\analytical_LCMS_DATA\EUbOPEN\CGC_ECHO1-3_FirstPass 2021-03-20 13-21-54
                  \CGL_FIRSTPASS_GENERALMETHOD_VIAL3+4_20210319.M (Sequence Method)
Last changed    : 3/19/2021 5:35:24 PM by SYSTEM
Method Info     : CGL wellplate, 0.5 uL of 10 mM DMSO, general method
```

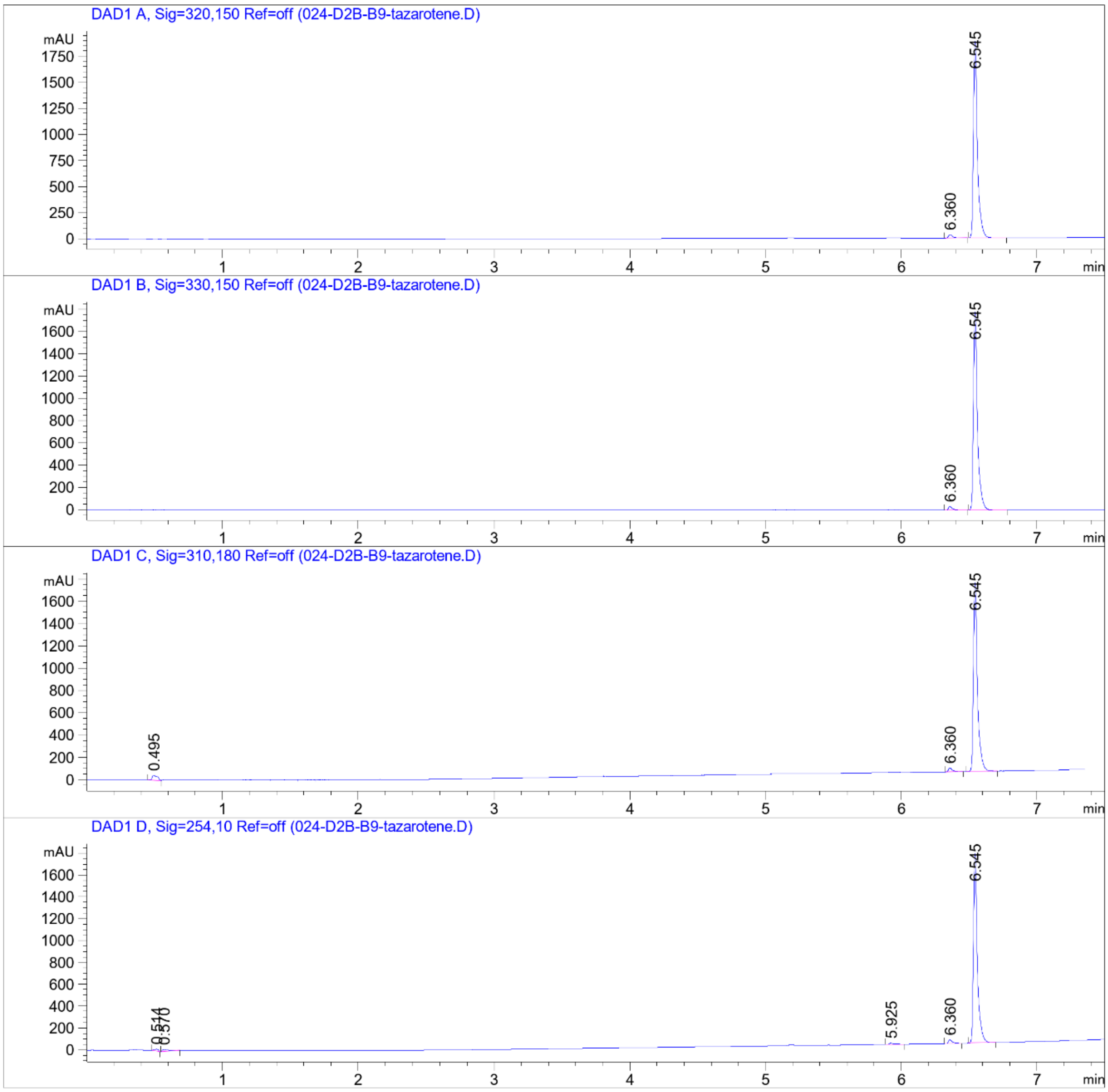

COMPOUND INFORMATION

Data File W:\analyti...N\CGC_ECHO1-3_FirstPass 2021-03-20 13-21-54\024-D2B-B9-tazarotene.D
Sample Name: tazarotene

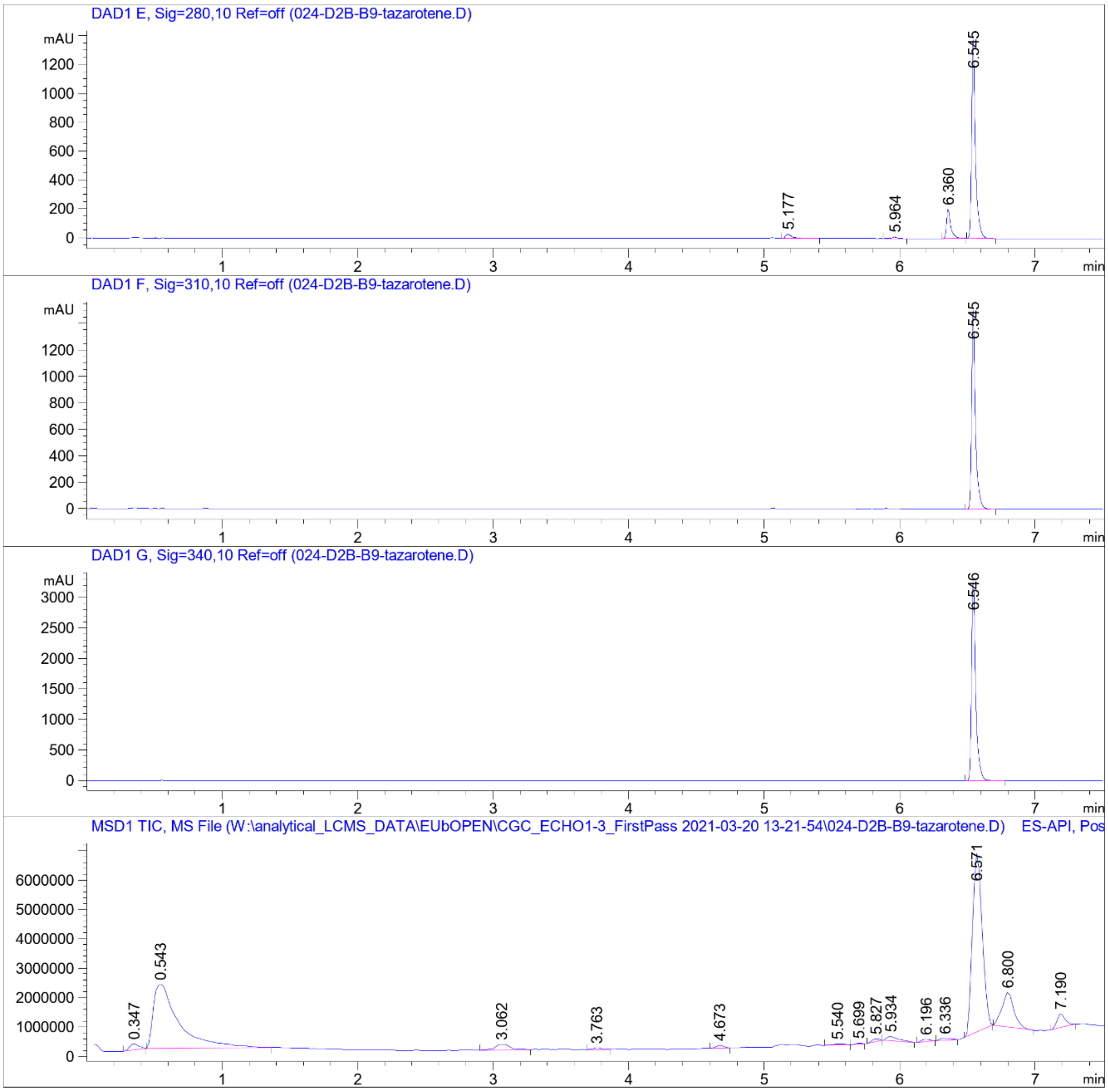

COMPOUND INFORMATION

Data File W:\analyti...N\CGC_ECHO1-3_FirstPass 2021-03-20 13-21-54\024-D2B-B9-tazarotene.D
Sample Name: tazarotene

MS Signal: MSD1 TIC, MS File, ES-API, Pos, Scan, Frag: 70, "POS Scan"
Spectra from peak tops.
Noise Cutoff: 1000 counts.
Reportable Ion Abundance: > 50%.
LC Signal: DAD1 A, Sig=320,150 Ref=off
Peak matching window: 0.1 min

Retention Time (LC)	LC Area	Retention Time (MS)	MS Area	Mol. Weight or Ion
-	-	0.347	937717	157.90 I
-	-	0.543	28776806	157.00 I
-	-	3.062	1403390	217.10 I
-	-	3.763	213235	274.20 I
-	-	4.673	390833	326.30 I
-	-	5.540	297630	326.30 I<br>295.20 I<br>282.30 I<br>280.20 I<br>102.10 I
-	-	5.699	152586	280.20 I
-	-	5.827	346908	296.20 I
-	-	5.934	843527	296.20 I<br>294.20 I<br>280.20 I
-	-	6.196	324168	280.20 I<br>228.20 I
6.360	71	6.336	379048	254.20 I
6.545	3847	6.571	30548758	352.10 I
-	-	6.800	7177743	282.20 I
-	-	7.190	1956745	284.20 I<br>282.20 I

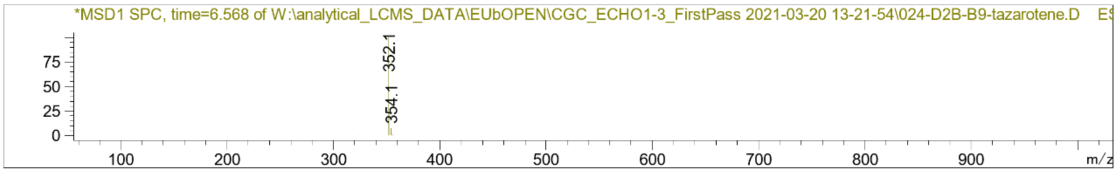

Supplement: Supplementary file 4 — Supplementary Data 1 [file 41467_2024_49493_MOESM4_ESM.zip › Tazarotene.pdf]
