# Supplementary material for: Chemogenomics for NR1 nuclear hormone receptors
Source: Nat Commun. 2024 Jun 18;15:5201. doi: 10.1038/s41467-024-49493-6 (PMC11189487; doi:10.1038/s41467-024-49493-6)

COMPOUND INFORMATION

Tesaglitazar

**CAS Registry No.:**	251565-85-2
**Formal Name:**	(S)-2-ethoxy-3-(4-(4-((methylsulfonyl)oxy)phenethoxy)phenyl)propanoic acid
**EUbOPEN ID:**	EUB0001144a
**Molecular Formula:**	$C_{20}H_{24}O_7S$
**Molecular Weight:**	408.47 g/mol
**Smiles:**	CCO[C@H](C(O)=O)CC1=CC=C(OCCC2=CC=C(OS(=O)(C)=O)C=C2)C=C1
**Recommended concentration:**	1 µM

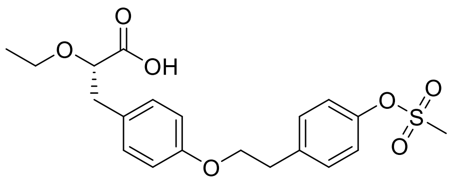

Biological activity

		Type	IC_{50}/EC_{50} [µM]	Reference
Main NR target:	NR1C1 (PPARα)	Agonist	0.29	inhouse
	NR1C2 (PPARγ)	Agonist	0.127	
NR off-target:				

COMPOUND INFORMATION

Identity

^{1}H NMR

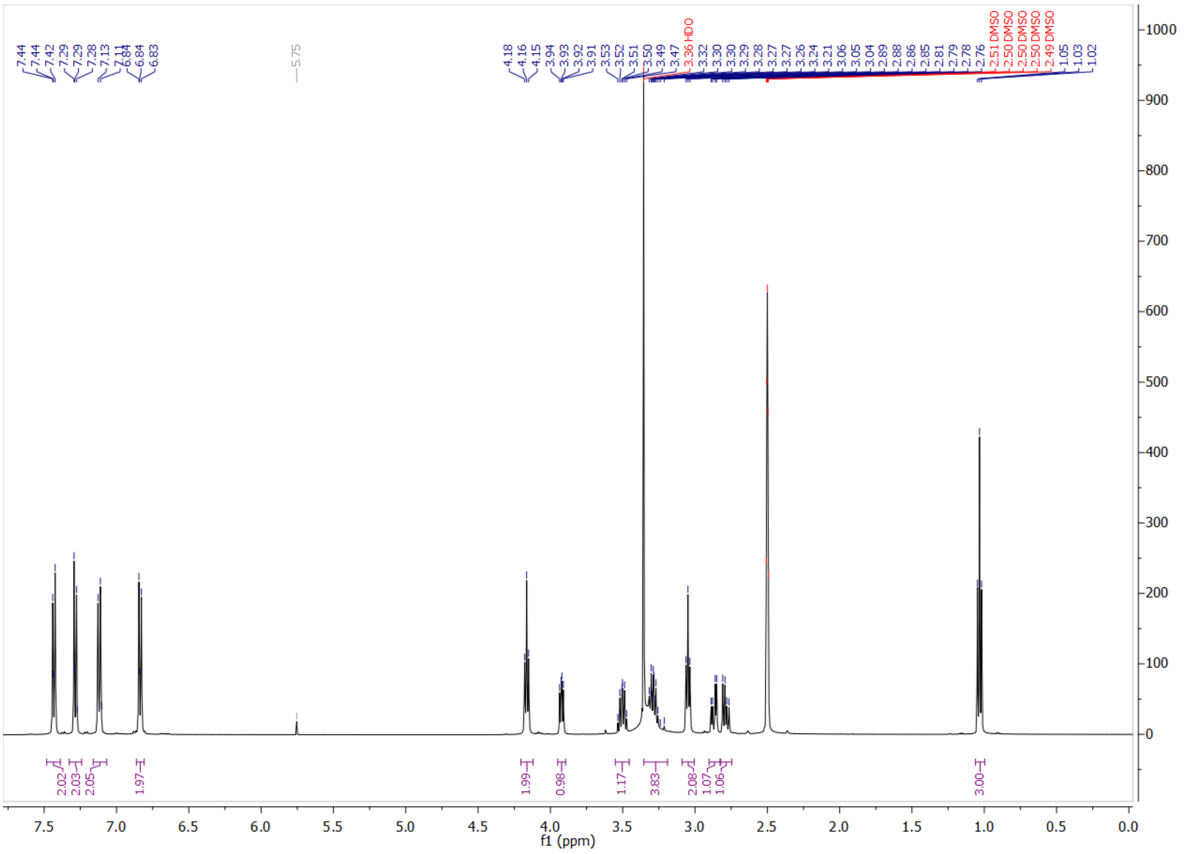

^{13}C NMR

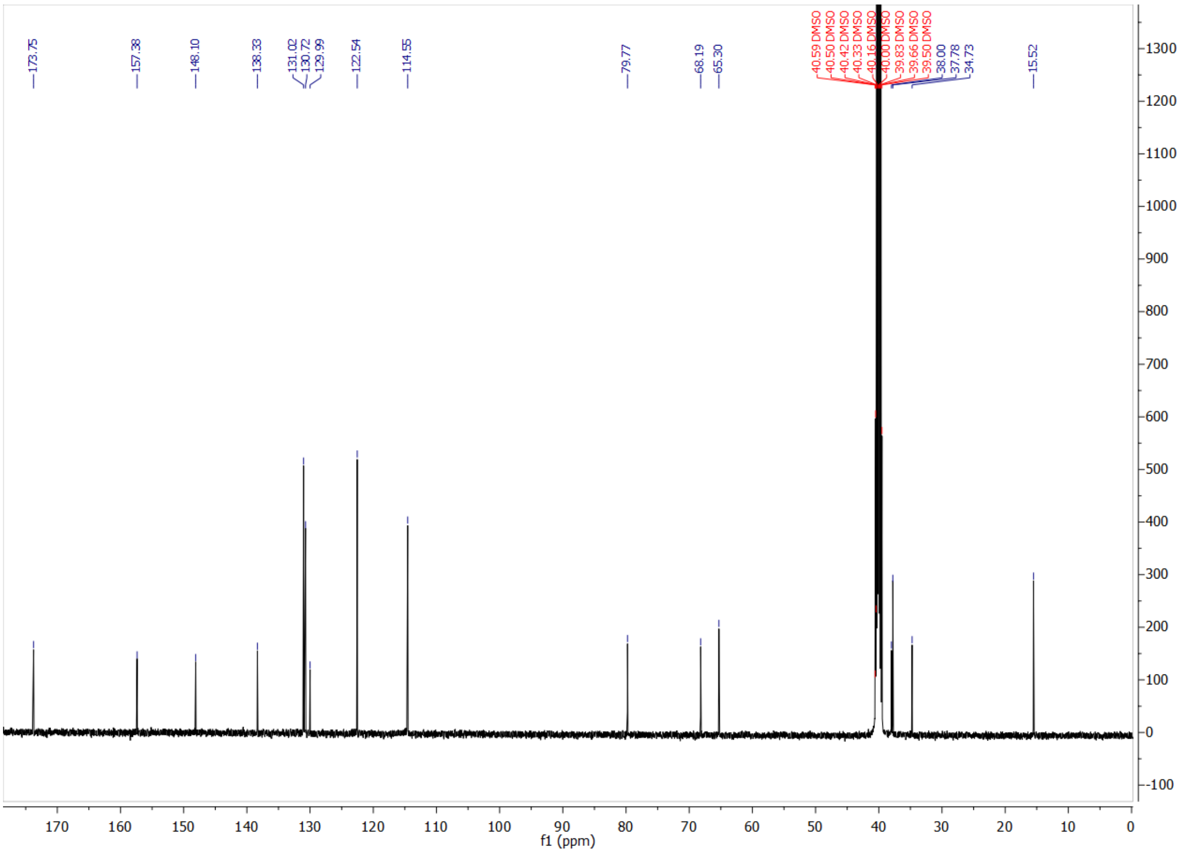

COMPOUND INFORMATION

Purity

M_r 408.47

MS: ESI-positive, *m/z* 409/199 (blue), *m/z* 409/121 (red)

LC: 0.1% HCOOH/ACN (40/60)

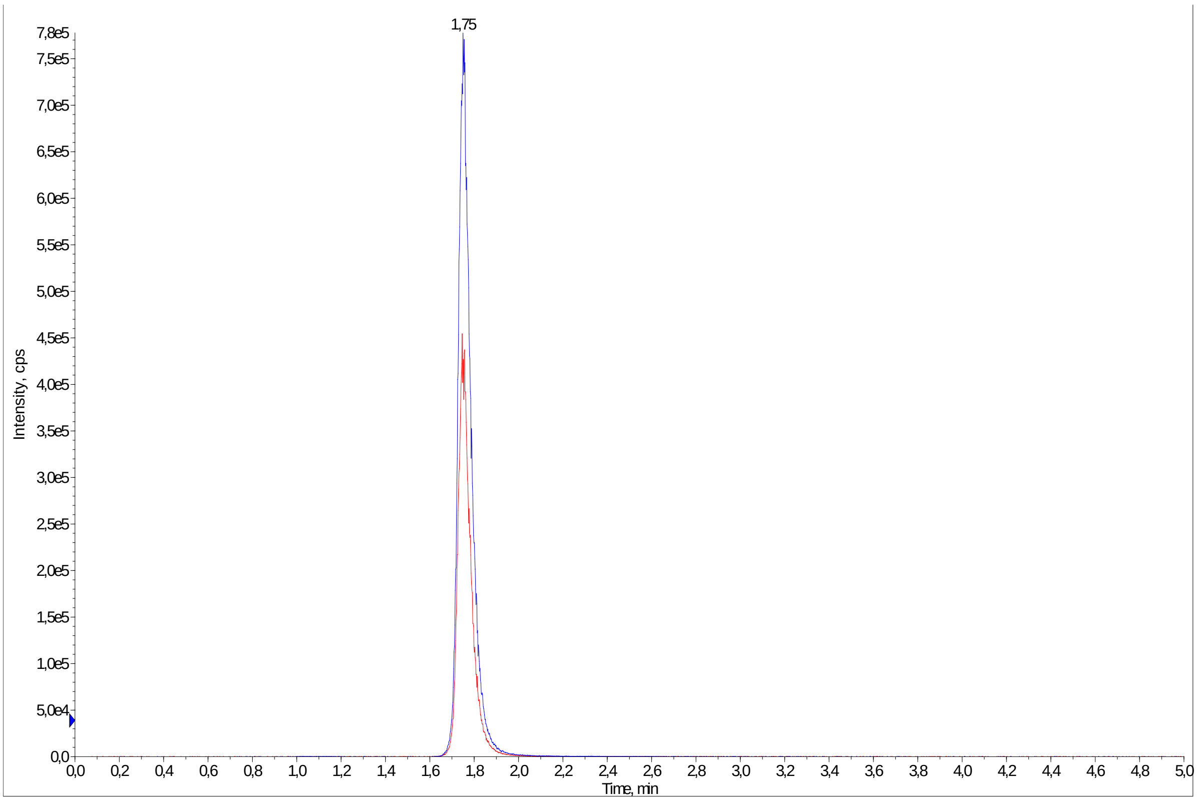

COMPOUND INFORMATION

LC-UV

LC: 0.1% HCOOH/ACN (40/60)

DAD: 210, 230 (XWC), 240, 254, 280 nm

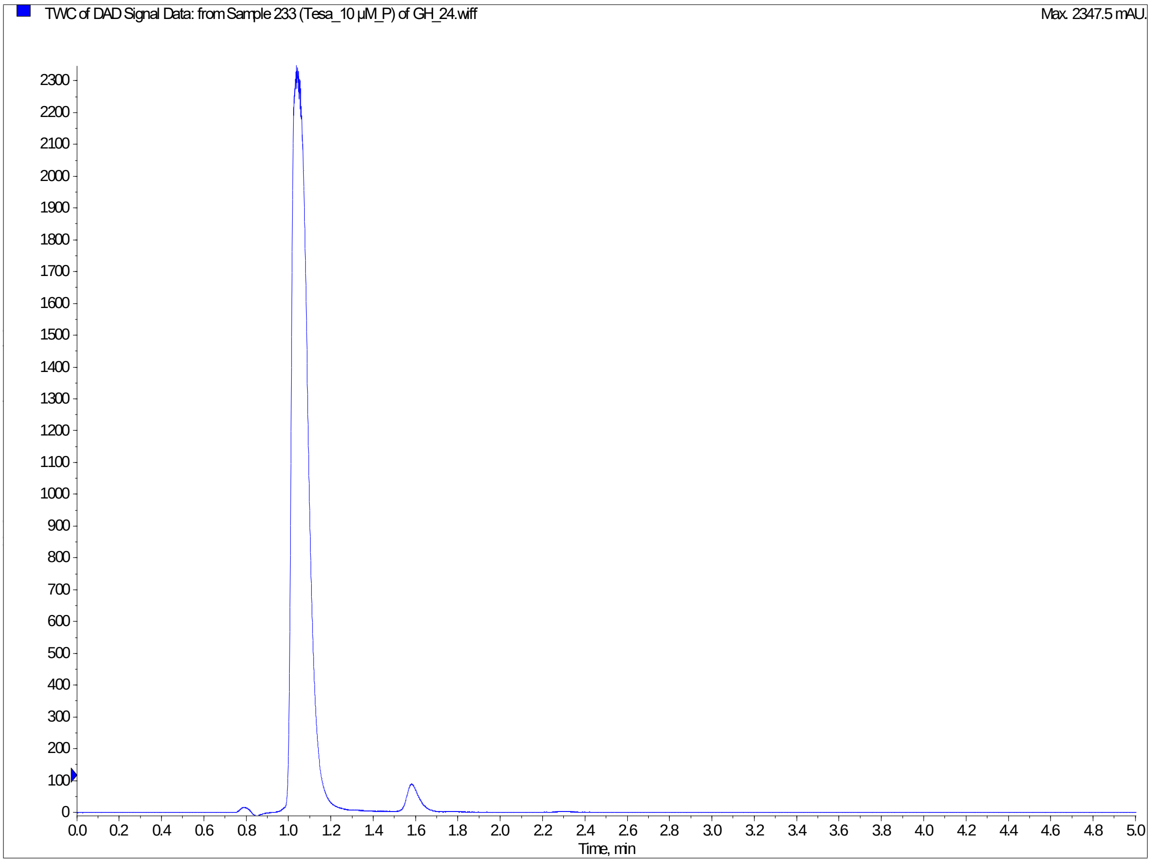

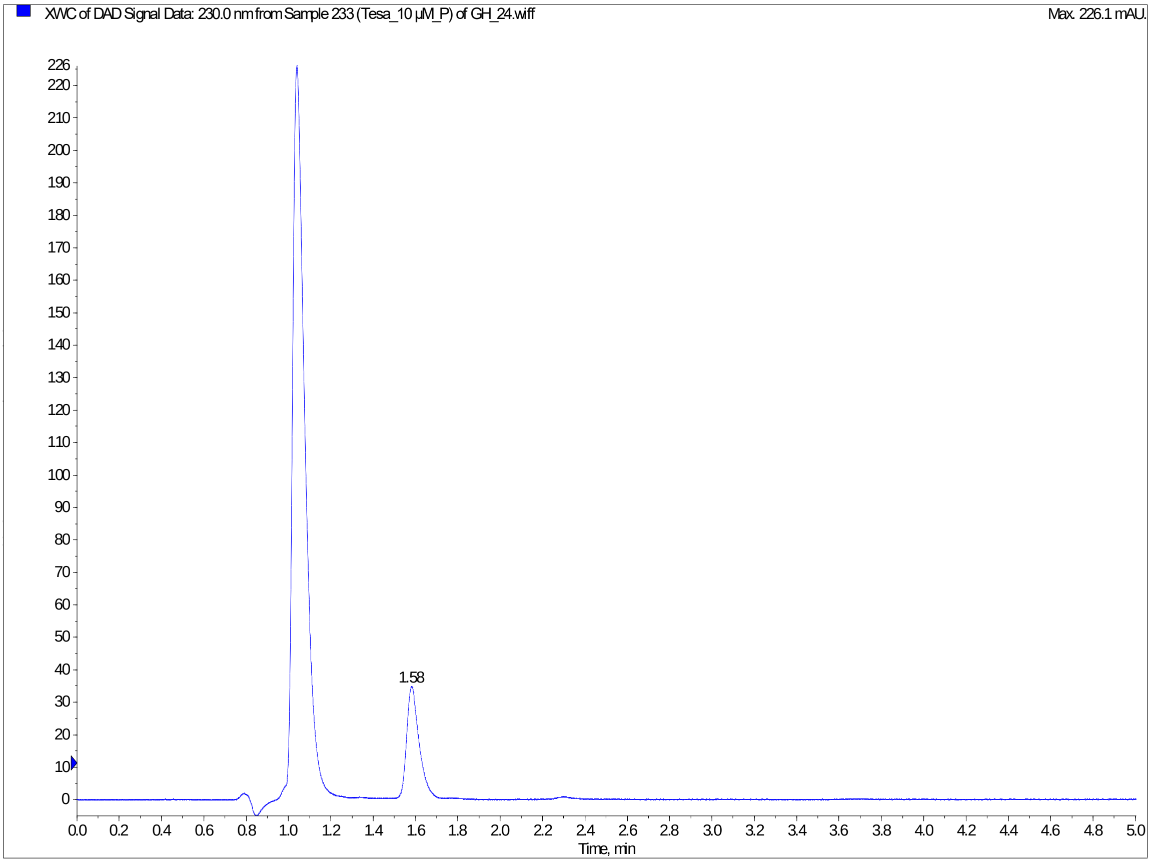

COMPOUND INFORMATION

Biological activity

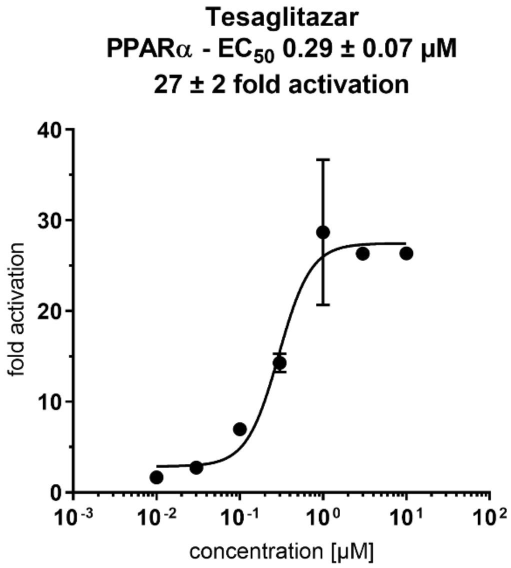

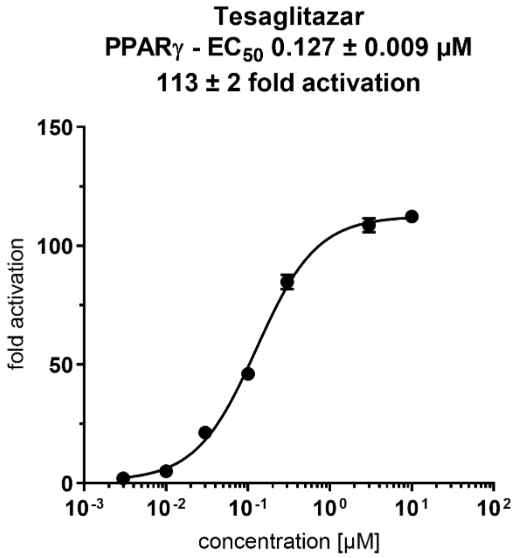

Supplement: Supplementary file 4 — Supplementary Data 1 [file 41467_2024_49493_MOESM4_ESM.zip › Tesaglitazar.pdf]
