# Supplementary material for: Chemogenomics for NR1 nuclear hormone receptors
Source: Nat Commun. 2024 Jun 18;15:5201. doi: 10.1038/s41467-024-49493-6 (PMC11189487; doi:10.1038/s41467-024-49493-6)

# **COMPOUND** INFORMATION

# Tiratricol

| | |
|---|---|
| **CAS Registry No.:** | 51-24-1 |
| **Formal Name:** | 2-(4-(4-hydroxy-3-iodophenoxy)-3,5-diiodophenyl)acetic acid |
| **EUbOPEN ID:** | EUB0001139a |
| **Molecular Formula:** | $C_{14}H_9I_3O_4$ |
| **Molecular Weight:** | 621,94 g/mol |
| **Smiles:** | C1=CC(=C(C=C1OC2=C(C=C(C=C2I)CC(=O)O)I)I)O |
| **Recommended concentration:** | 1 µM |

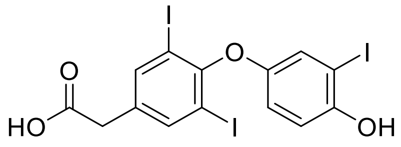


### Biological activity

| | | Type | $IC_{50}/EC_{50}$ [µM] | Reference |
|---|---|---|---|---|
| Main NR target: | NR1A1 (THRα) | Antagonist | 0.002 | https://doi.org/10.1002/jat.2825 |
| | NR1A2 (THRβ) | Antagonist | 0.004 | |
| NR off-target: | | | | |

# **COMPOUND** INFORMATION

# Identity

### $^{1}$H NMR

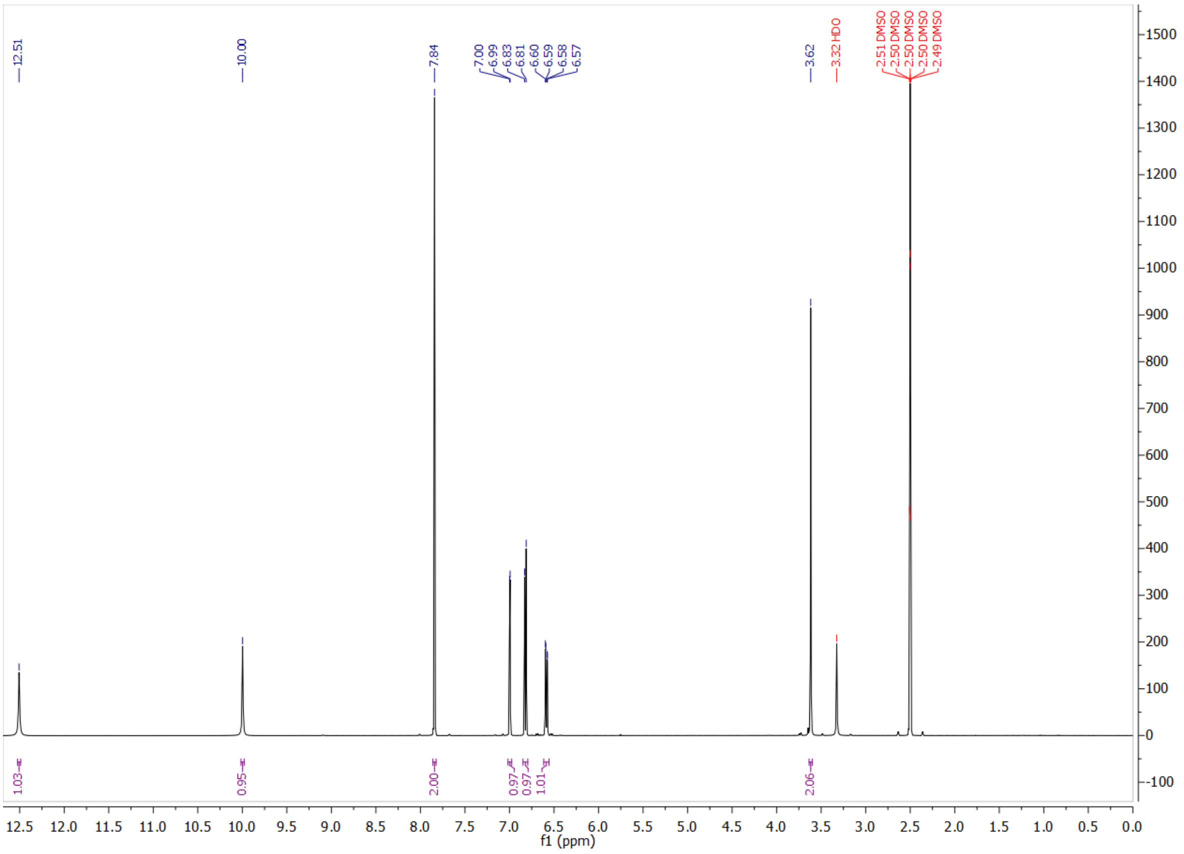

### $^{13}$C NMR

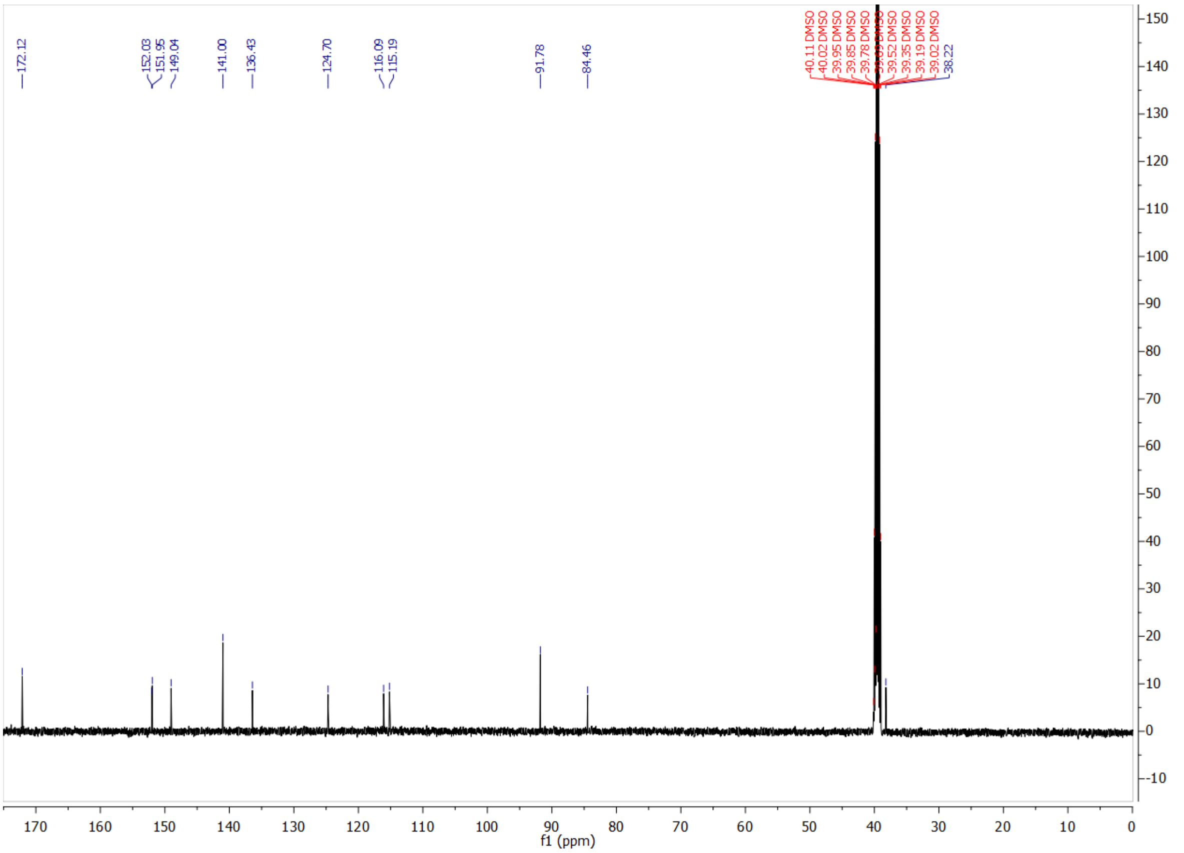

# **COMPOUND** INFORMATION

## Purity

$M_r$ 621.94

MS: ESI-negative, *m/z* 621/127 (red), *m/z* 577/127 (blue, see R.M.S. Jongejan et al., Clin. Chem. 556-566, 66, 2020)

LC: 0.1% HCOOH/ACN (40/60)

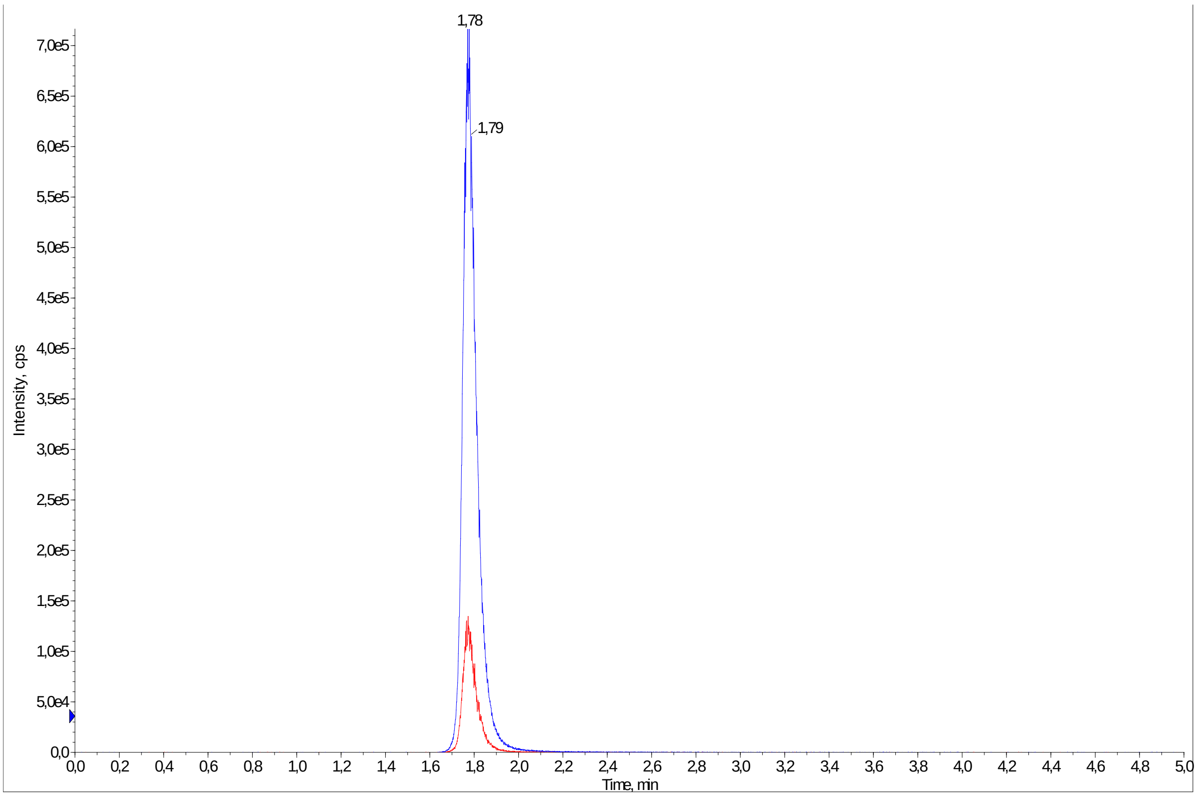

# **COMPOUND** INFORMATION

**LC-UV**

LC: 0.1% HCOOH/ACN (40/60)

DAD: 210, 230, 240, 254 (XWC), 280 nm

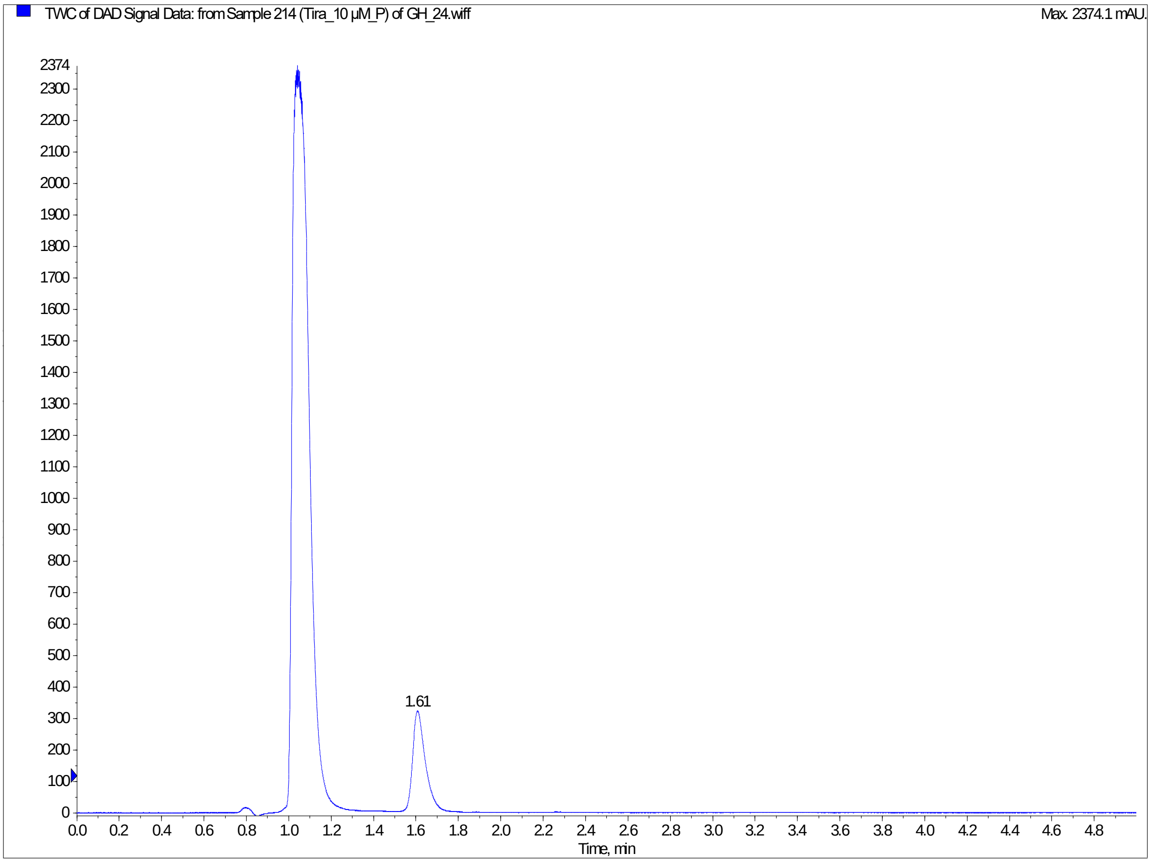


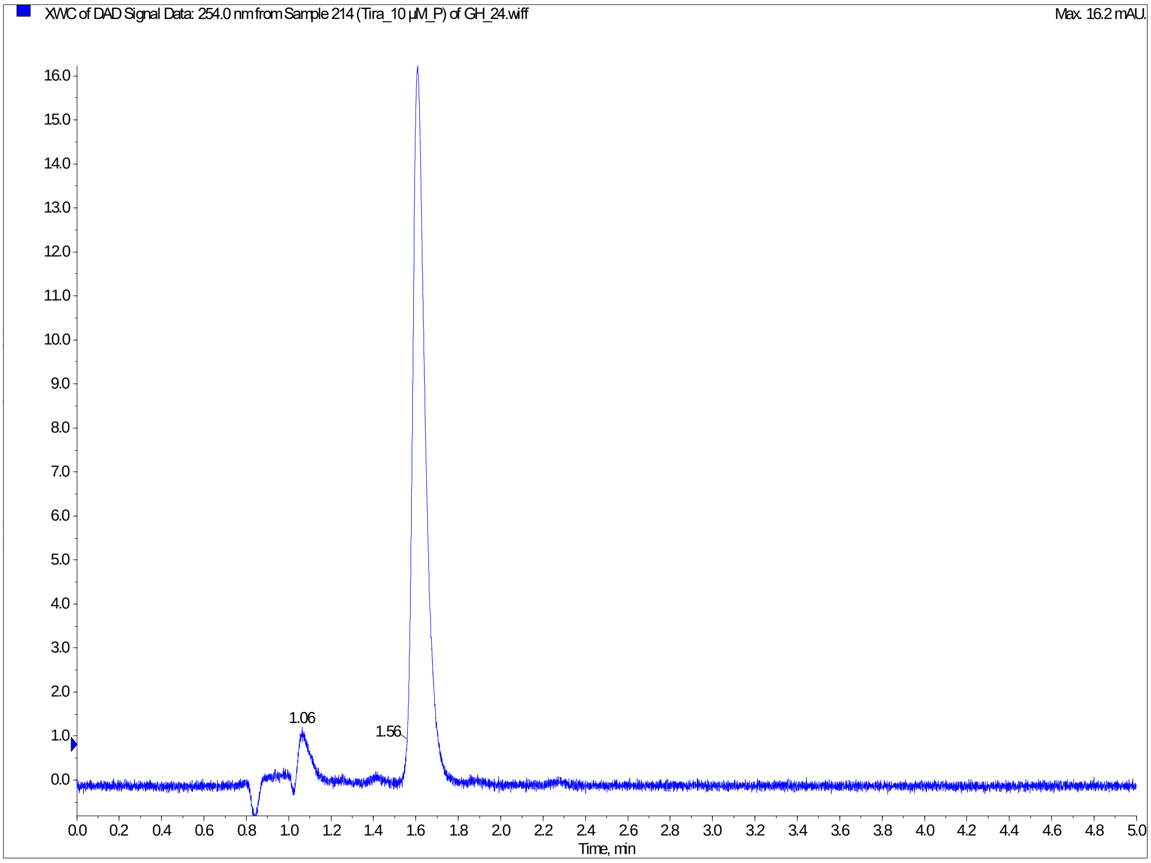

Supplement: Supplementary file 4 — Supplementary Data 1 [file 41467_2024_49493_MOESM4_ESM.zip › Tiratricol.pdf]
