# Supplementary material for: Chemogenomics for NR1 nuclear hormone receptors
Source: Nat Commun. 2024 Jun 18;15:5201. doi: 10.1038/s41467-024-49493-6 (PMC11189487; doi:10.1038/s41467-024-49493-6)

COMPOUND INFORMATION

Tropifexor

**CAS Registry No.:**	1383816-29-2
**Formal Name:**	2-(3-((5-cyclopropyl-3-(2-(trifluoromethoxy)phenyl)isoxazol-4-yl)methoxy)-8-azabicyclo[3.2.1]octan-8-yl)-4-fluorobenzo[d]thiazole-6-carboxylic acid
**EUBOPEN ID:**	EUB0001169a
**Molecular Formula:**	$C_{29}H_{25}F_4N_3O_5S$
**Molecular Weight:**	603.59 g/mol
**Smiles:**	O=C(C1=CC(F)=C2N=C(N3C4CC(OCC5=C(C6CC6)ON=C5C7=CC=CC=C7OC(F)(F)F)CC3CC4)SC2=C1)O
**Recommended concentration:**	1 µM

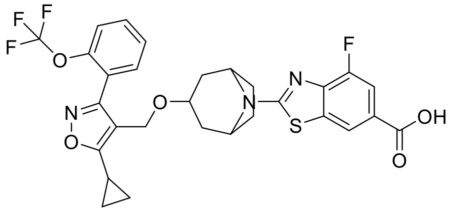

Biological activity

		Type	IC_{50}/EC_{50} [µM]	Reference
Main NR target:	NR1H4 (FXR)	Agonist	0.0003	https://doi.org/10.1021/acs.jmedchem.7b00907
NR off-target:				

COMPOUND INFORMATION

Identity

^{1}H NMR

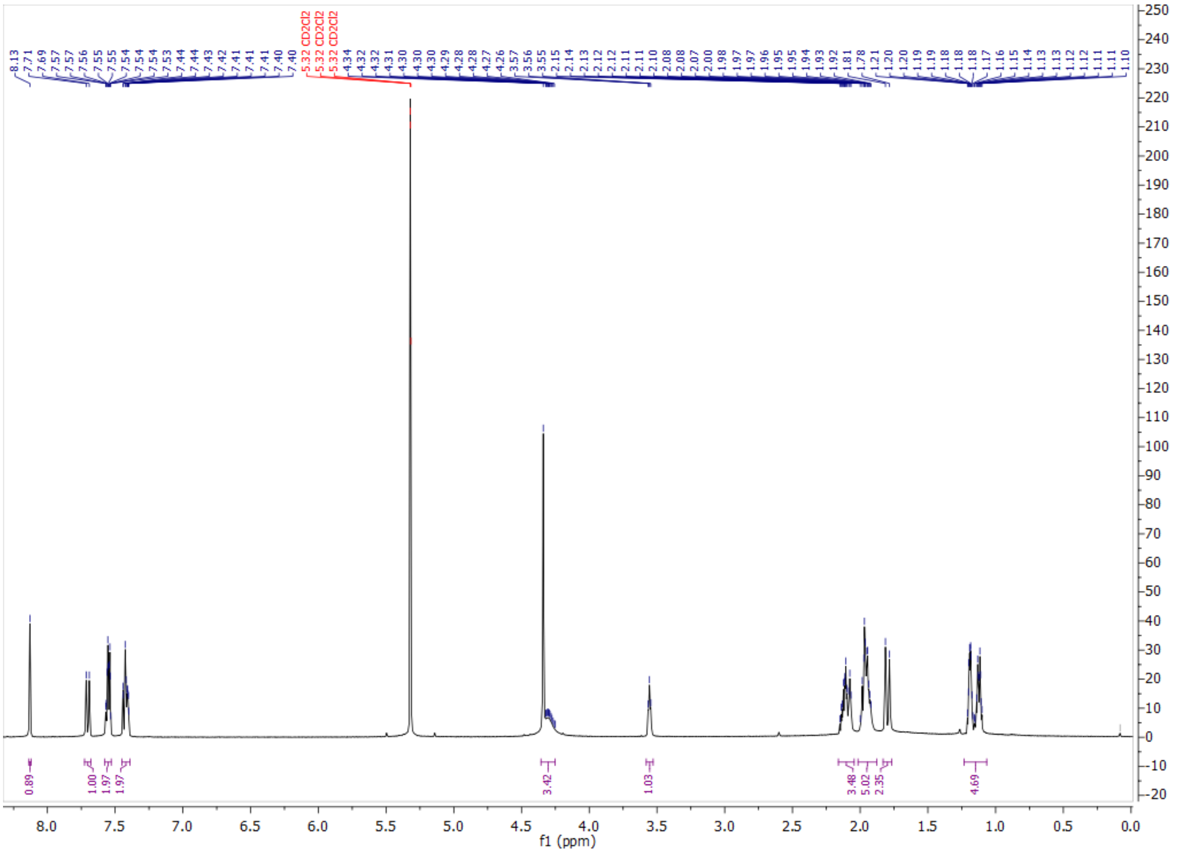

^{13}C NMR

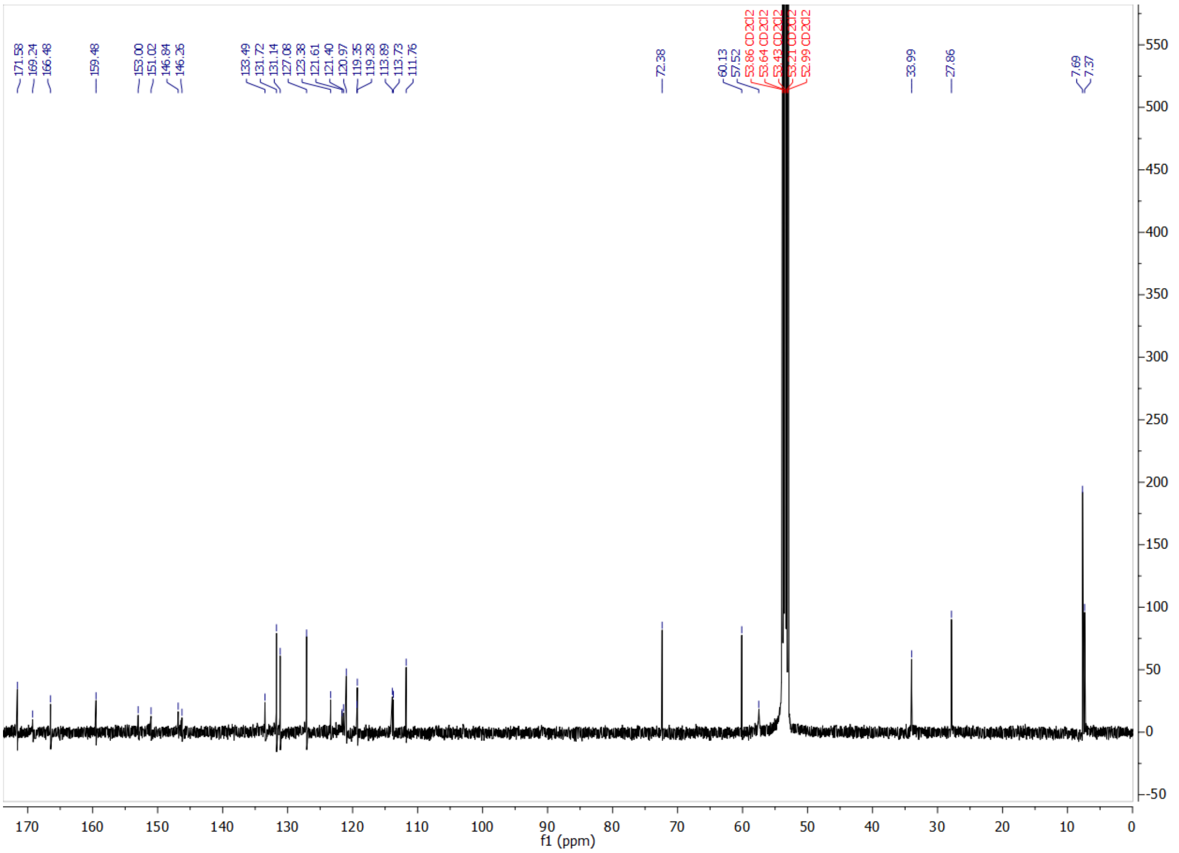

COMPOUND INFORMATION

Purity

```
Data File W:\analyti...\CGC_wave3_1_FirstPassB 2023-01-04 18-28-02\092-D2F-H5-Tropifexor.D
Sample Name: Tropifexor

    =====================================================================
    Acq. Operator   : SYSTEM                          Seq. Line :   92
    Sample Operator : SYSTEM
    Acq. Instrument : LCMS test                        Location :   D2F-H5
    Injection Date  : 1/5/2023 11:17:09 AM                  Inj :   1
                                                    Inj Volume : Inj prog
    Sequence File   : W:\analytical_LCMS_DATA\EUbOPEN\CGC_wave3_1_FirstPassB 2023-01-04 18-28-02
                      \CGC_wave3_1_FirstPassB.S
    Method          : W:\analytical_LCMS_DATA\EUbOPEN\CGC_wave3_1_FirstPassB 2023-01-04 18-28-02
                      \CGL_FIRSTPASS_GENERALMETHOD_VIAL1+2_20210319.M (Sequence Method)
    Last changed    : 1/25/2022 4:36:18 PM by SYSTEM
    Method Info     : CGL wellplate, 0.5 uL of 10 mM DMSO, general method
```

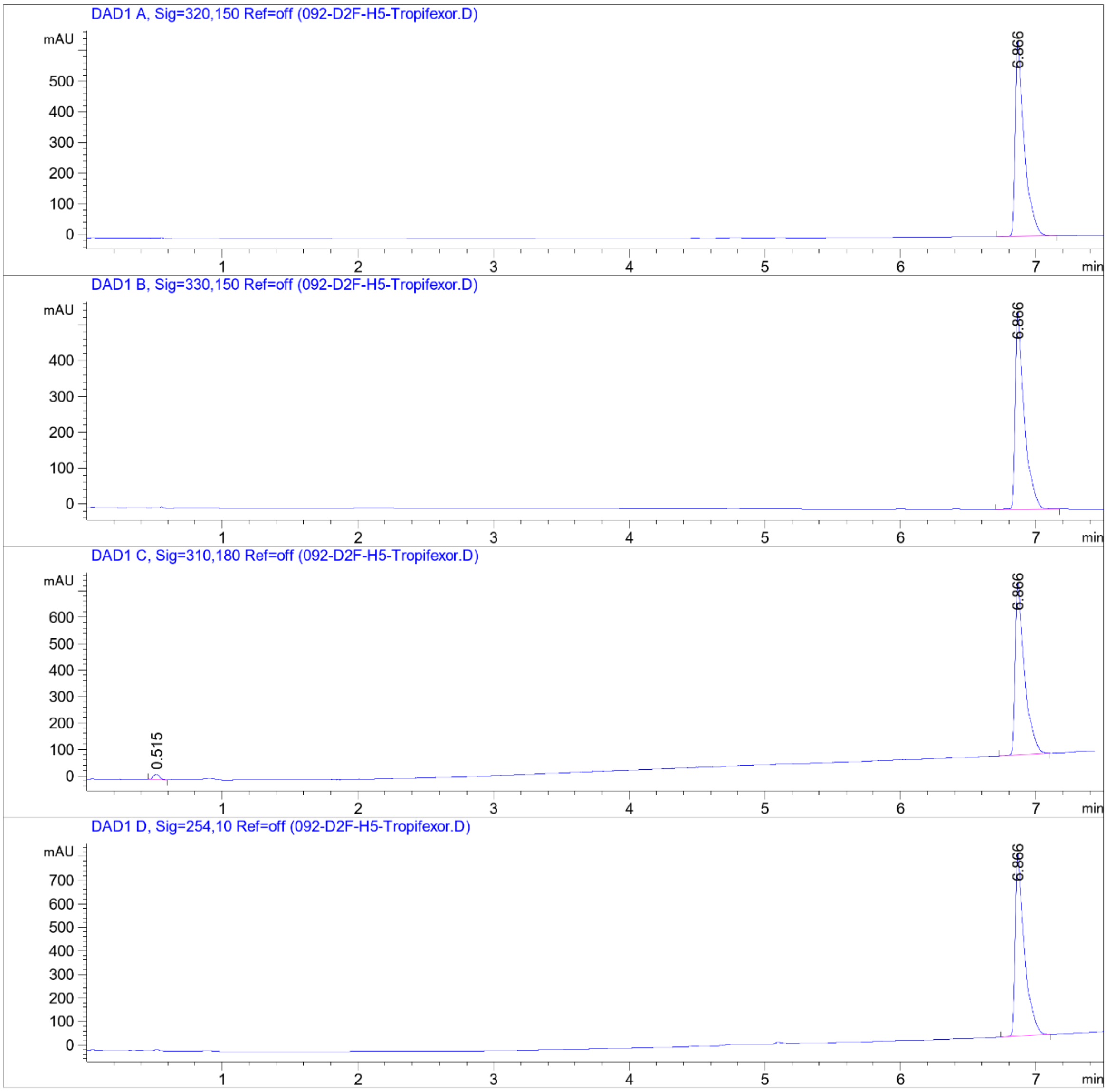

COMPOUND INFORMATION

Data File W:\analyti...\CGC_wave3_1_FirstPassB 2023-01-04 18-28-02\092-D2F-H5-Tropifexor.D
Sample Name: Tropifexor

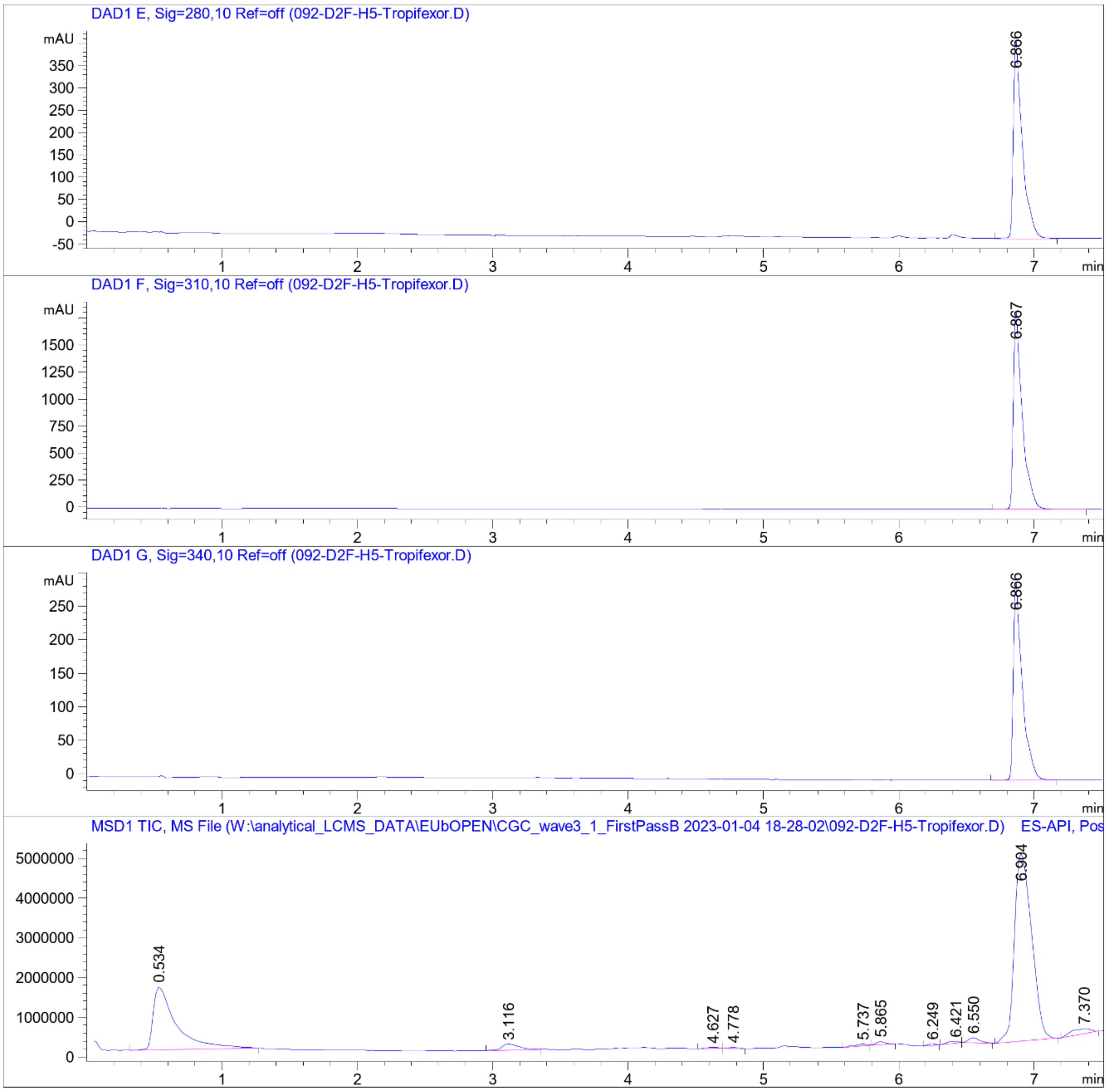

COMPOUND INFORMATION

Data File W:\analyti...\CGC_wave3_1_FirstPassB 2023-01-04 18-28-02\092-D2F-H5-Tropifexor.D
Sample Name: Tropifexor

MS Signal: MSD1 TIC, MS File, ES-API, Pos, Scan, Frag: 70, "POS Scan"
Spectra from peak tops.
Noise Cutoff: 1000 counts.
Reportable Ion Abundance: > 50%.
LC Signal: DAD1 A, Sig=320,150 Ref=off
Peak matching window: 0.1 min

Retention Time (LC)	LC Area	Retention Time (MS)	MS Area	Mol. Weight or Ion
-	-	0.534	18485472	157.00 I
-	-	3.116	1355728	239.00 I
				217.10 I
-	-	4.627	161282	510.30 I
				170.90 I
				158.20 I
				137.10 I
-	-	4.778	134497	279.10 I
				137.10 I
-	-	5.737	206667	280.20 I
-	-	5.865	370628	318.20 I
				296.20 I
-	-	6.249	167731	228.20 I
				137.10 I
-	-	6.421	234804	350.20 I
				282.20 I
				254.20 I
				137.10 I
-	-	6.550	724366	507.30 I
				485.30 I
				280.20 I
6.866	2926	6.904	41956712	604.20 I
-	-	7.370	1335602	400.30 I
				282.20 I

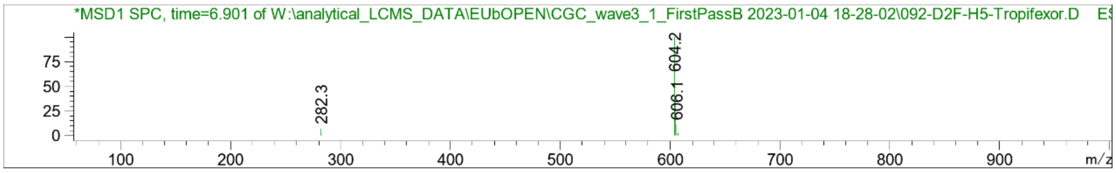

Supplement: Supplementary file 4 — Supplementary Data 1 [file 41467_2024_49493_MOESM4_ESM.zip › Tropifexor.pdf]
