# Supplementary material for: Chemogenomics for NR1 nuclear hormone receptors
Source: Nat Commun. 2024 Jun 18;15:5201. doi: 10.1038/s41467-024-49493-6 (PMC11189487; doi:10.1038/s41467-024-49493-6)

COMPOUND INFORMATION

XL335

**CAS Registry No.:**	629664-81-9
**Formal Name:**	Isopropyl 3-(3,4-difluorobenzoyl)-1,1-dimethyl-1,2,3,6-tetrahydroazepino[4,5-b]indole-5-carboxylate
**EUBOPEN ID:**	EUB0000573a
**Molecular Formula:**	$C_{25}H_{24}F_2N_2O_3$
**Molecular Weight:**	438.47 g/mol
**Smiles:**	CC(OC(C1=CN(C(C2=CC(F)=C(F)C=C2)=O)CC(C)(C)C3=C1NC4=CC=CC=C43)=O)C
**Recommended concentration:**	1 µM

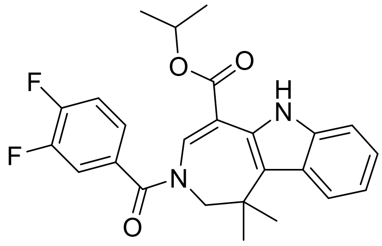

Biological activity

		Type	IC_{50}/EC_{50} [µM]	Reference
Main NR target:	NR1H4 (FXR)	Agonist	0.099	inhouse
NR off-target:				

COMPOUND INFORMATION

Identity

^{1}H NMR

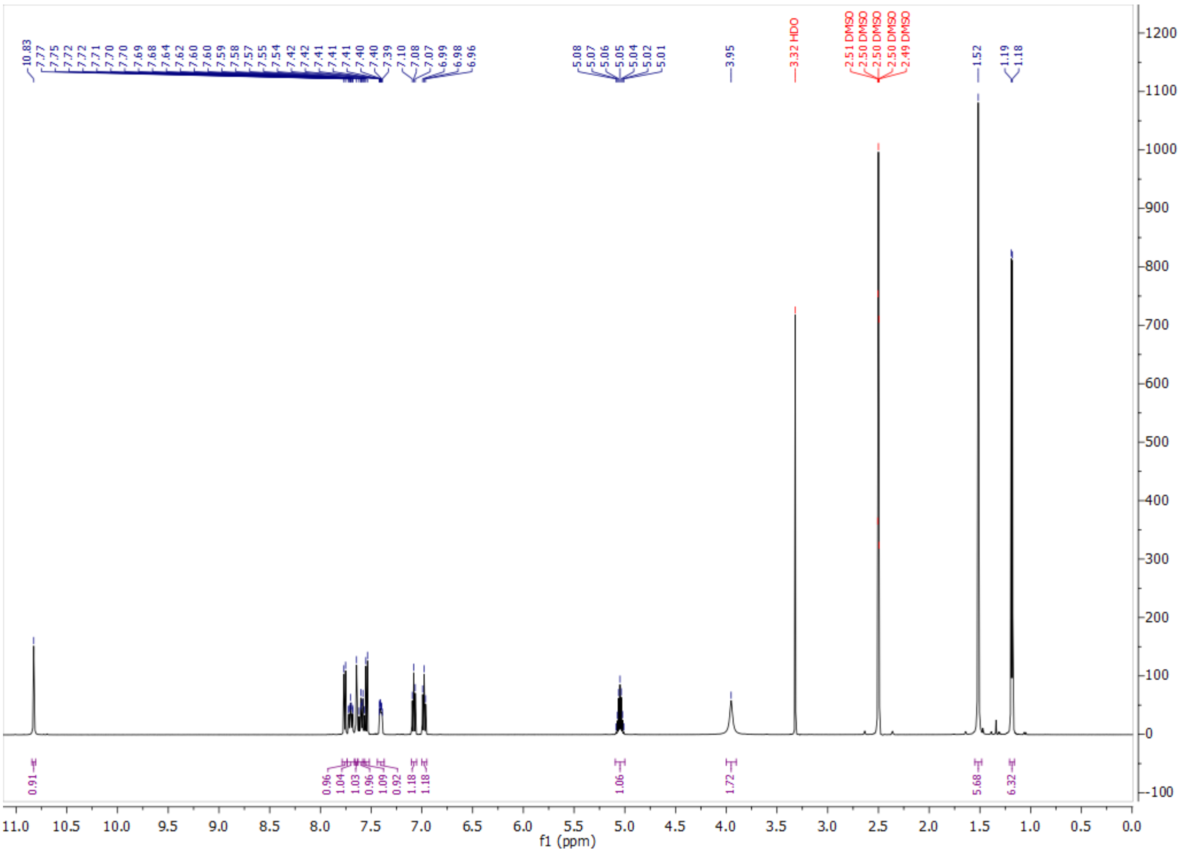

^{13}C NMR

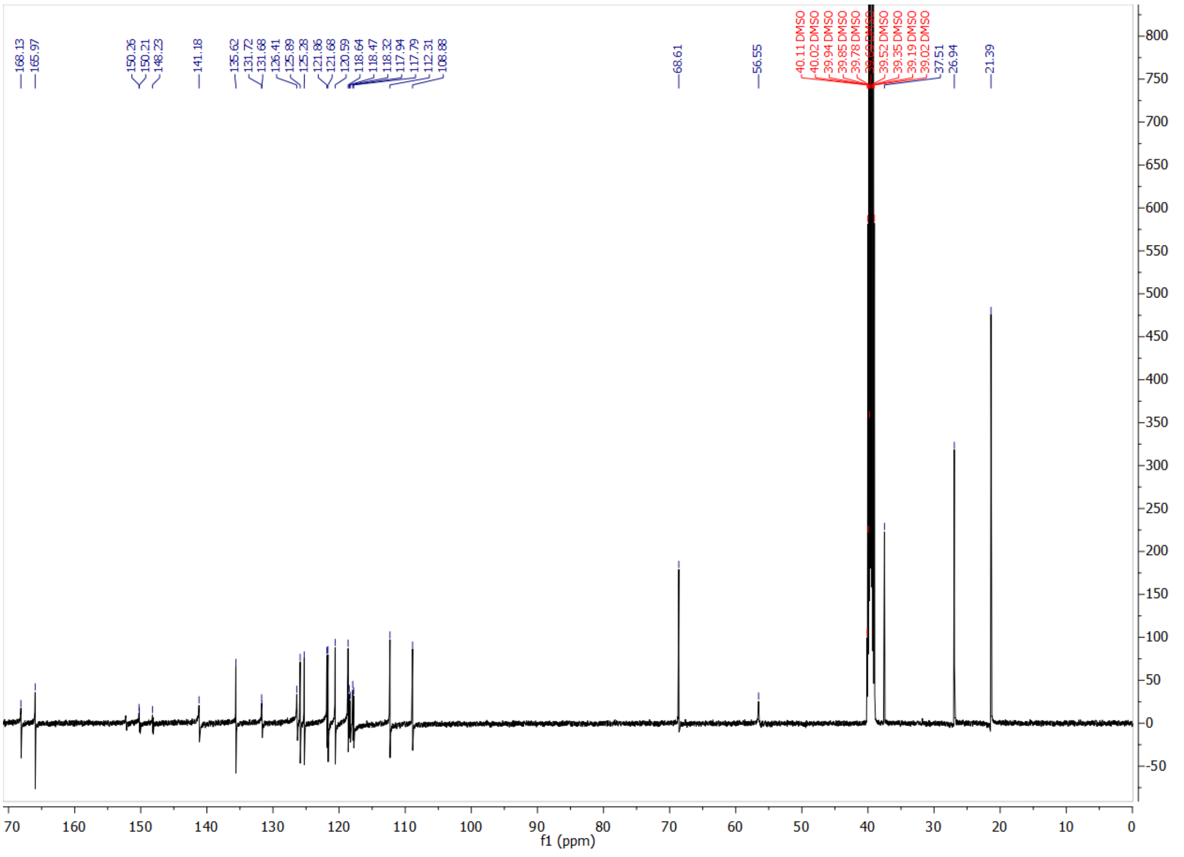

COMPOUND INFORMATION

Purity

```
Data File W:\analyti...bOPEN\CGC_ECHO1-3_FirstPass 2021-03-20 13-21-54\039-D2B-D11-xl335.D
Sample Name: xl335

    =====================================================================
    Acq. Operator   : SYSTEM                          Seq. Line :  39
    Sample Operator : SYSTEM
    Acq. Instrument : LCMS test                        Location :   D2B-D11
    Injection Date  : 3/20/2021 8:26:46 PM                  Inj :   1
                                                    Inj Volume : Inj prog
    Sequence File   : W:\analytical_LCMS_DATA\EUbOPEN\CGC_ECHO1-3_FirstPass 2021-03-20 13-21-54
                      \CGC_ECHO1-3_FirstPass.S
    Method          : W:\analytical_LCMS_DATA\EUbOPEN\CGC_ECHO1-3_FirstPass 2021-03-20 13-21-54
                      \CGL_FIRSTPASS_GENERALMETHOD_VIAL3+4_20210319.M (Sequence Method)
    Last changed    : 3/19/2021 5:35:24 PM by SYSTEM
    Method Info     : CGL wellplate, 0.5 uL of 10 mM DMSO, general method
```

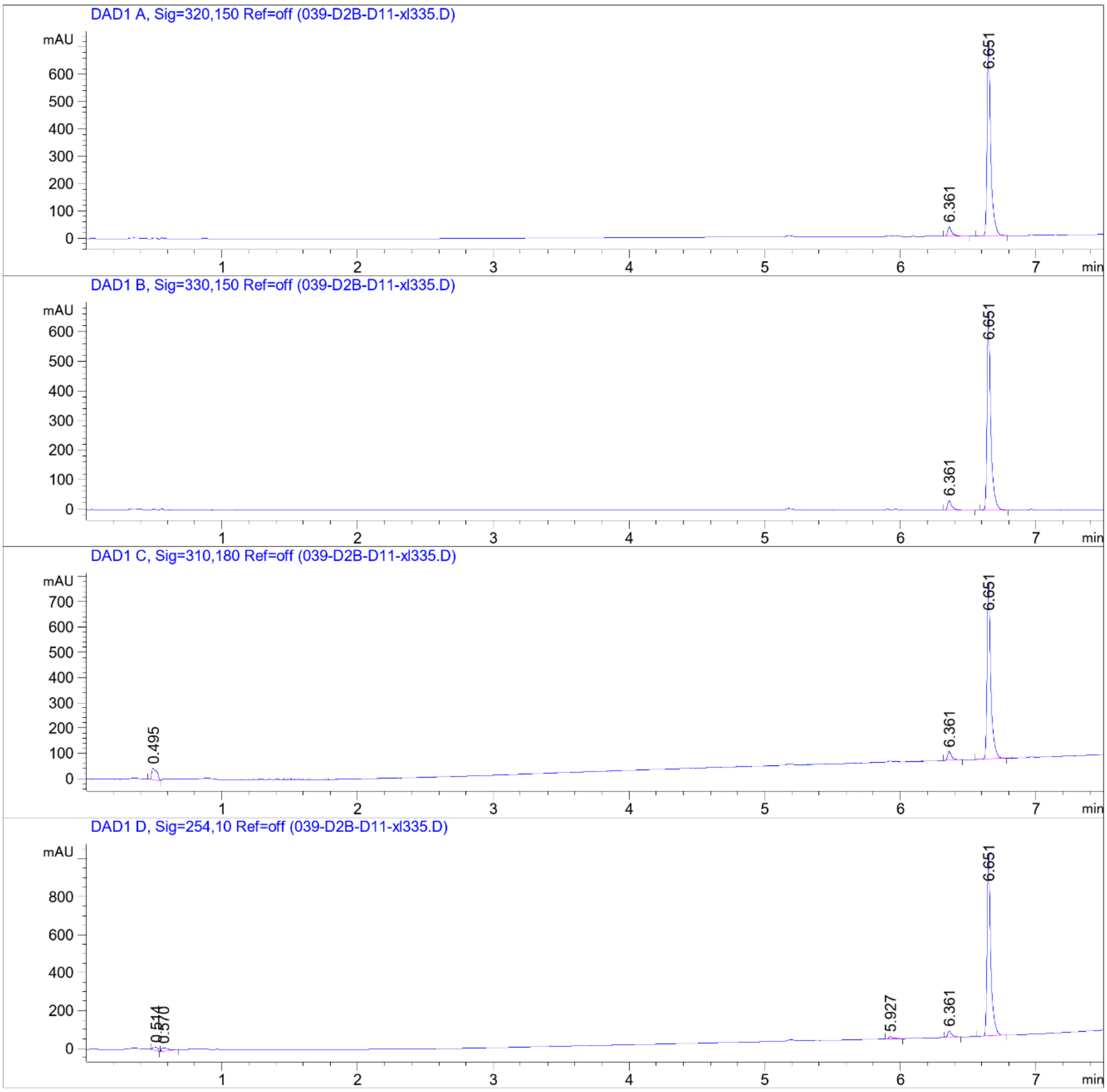

COMPOUND INFORMATION

Data File W:\analyti...bOPEN\CGC_ECHO1-3_FirstPass 2021-03-20 13-21-54\039-D2B-D11-xl335.D
Sample Name: xl335

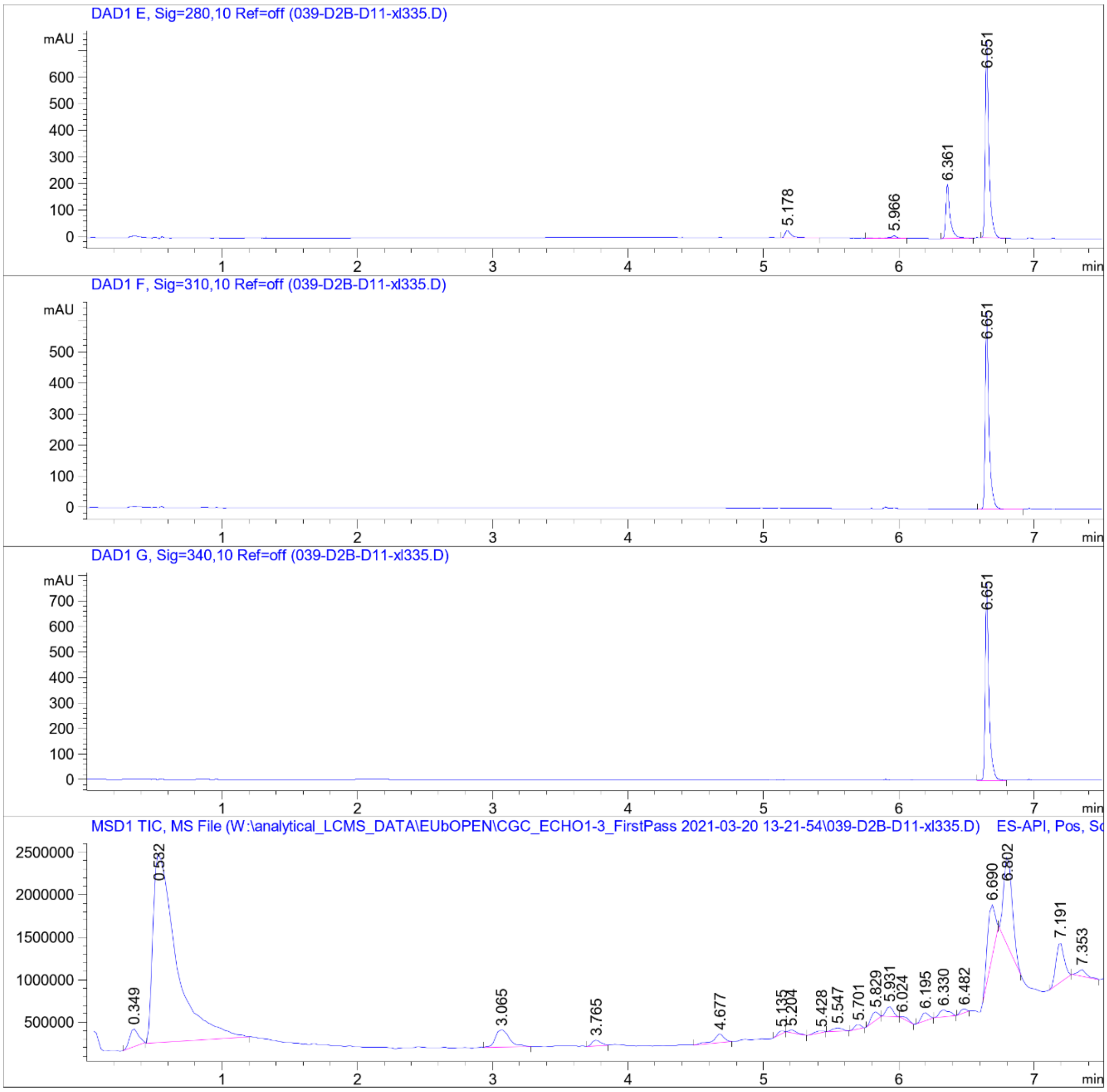

COMPOUND INFORMATION

Data File W:\analyti...bOPEN\CGC_ECHO1-3_FirstPass 2021-03-20 13-21-54\039-D2B-D11-xl335.D
Sample Name: xl335

MS Signal: MSD1 TIC, MS File, ES-API, Pos, Scan, Frag: 70, "POS Scan"
Spectra from peak tops.
Noise Cutoff: 1000 counts.
Reportable Ion Abundance: > 50%.
LC Signal: DAD1 A, Sig=320,150 Ref=off
Peak matching window: 0.1 min

Retention Time (LC)	LC Area	Retention Time (MS)	MS Area	Mol. Weight or Ion
-	-	0.349	975039	158.00 I
				130.00 I
-	-	0.532	26800514	157.00 I
-	-	3.065	1428043	217.00 I
-	-	3.765	289226	274.20 I
-	-	4.677	549076	326.40 I
-	-	5.135	98579	298.20 I
				295.10 I
				225.10 I
				102.10 I
-	-	5.204	126551	316.20 I
				111.00 I
				102.10 I
-	-	5.428	100580	310.20 I
				111.10 I
				102.10 I
-	-	5.547	208128	326.20 I
				282.20 I
				280.20 I
				102.10 I
-	-	5.701	172443	280.20 I
-	-	5.829	362638	296.20 I
-	-	5.931	418153	296.20 I
				280.20 I
-	-	6.024	111913	298.20 I
				296.30 I
				280.20 I
				102.20 I
-	-	6.195	360092	280.20 I
				228.20 I
6.361	73	6.330	428097	254.20 I
-	-	6.482	144000	280.20 I
6.651	1333	6.690	2542721	439.10 I
-	-	6.802	4482313	282.20 I
-	-	7.191	1959236	284.20 I
				282.20 I
-	-	7.353	344395	400.30 I
				282.20 I

COMPOUND INFORMATION

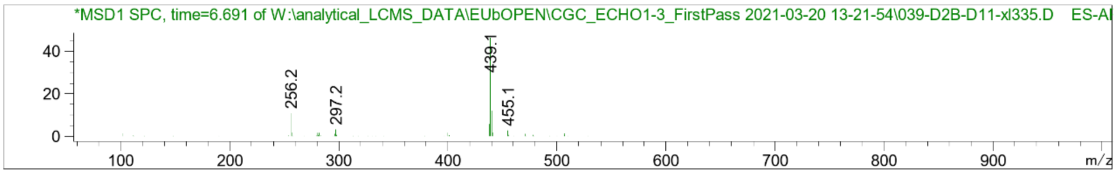

Biological activity

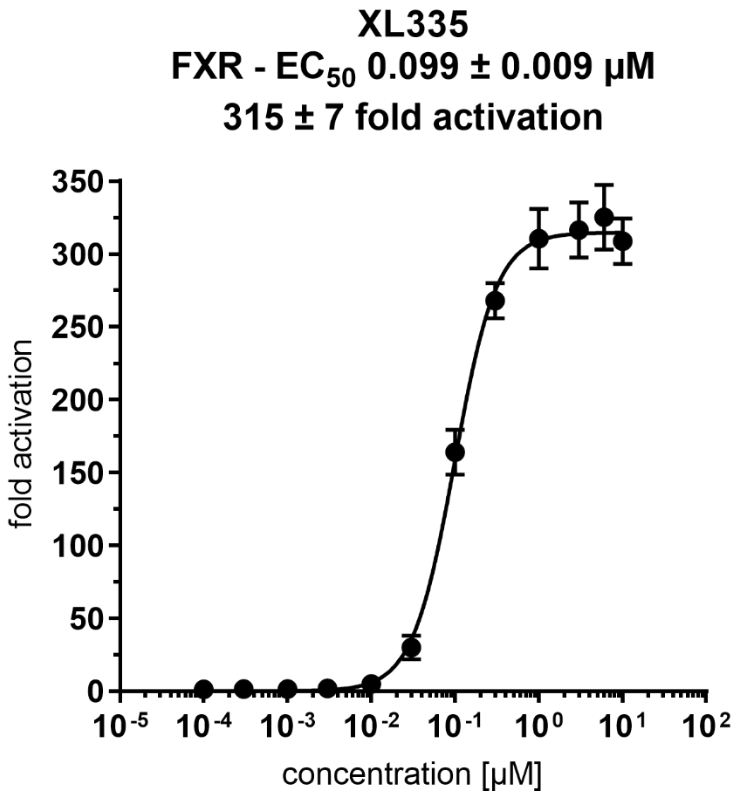

Supplement: Supplementary file 4 — Supplementary Data 1 [file 41467_2024_49493_MOESM4_ESM.zip › XL335.pdf]
